# Silicon functionalization expands the repertoire of Si-rhodamine fluorescent probes†

**DOI:** 10.1039/d2sc01821g

**Published:** 2022-05-02

**Authors:** Desaboini Nageswara Rao, Xincai Ji, Stephen C. Miller

**Affiliations:** Department of Biochemistry and Molecular Biotechnology, University of Massachusetts Chan Medical School Worcester Massachusetts 01605 USA Stephen.Miller@umassmed.edu

## Abstract

Fluorescent dyes such as rhodamines are widely used to assay the activity and image the location of otherwise invisible molecules. Si-rhodamines, in which the bridging oxygen of rhodamines is replaced with a dimethyl silyl group, are increasingly the dye scaffold of choice for biological applications, as fluorescence is shifted into the near-infrared while maintaining high brightness. Despite intense interest in Si-rhodamines, there has been no exploration of the scope of silicon functionalization in these dyes, a potential site of modification that does not exist in conventional rhodamines. Here we report a broad range of silyl modifications that enable brighter dyes, further red-shifting, new ways to modulate fluorescence, and the introduction of handles for dye attachment, including fluorogenic labeling agents for nuclear DNA, SNAP-tag and HaloTag labeling. Modifications to the bridging silicon are therefore of broad utility to improve and expand the applications of all Si-dyes.

## Abstract 2

Functionalization of the bridging silicon atom of Si-rhodamine dyes allows tuning of dye performance, the attachment of sensors, and the addition of biomolecular targeting ligands useful for the construction of live cell imaging probes.

## Graphical abstract

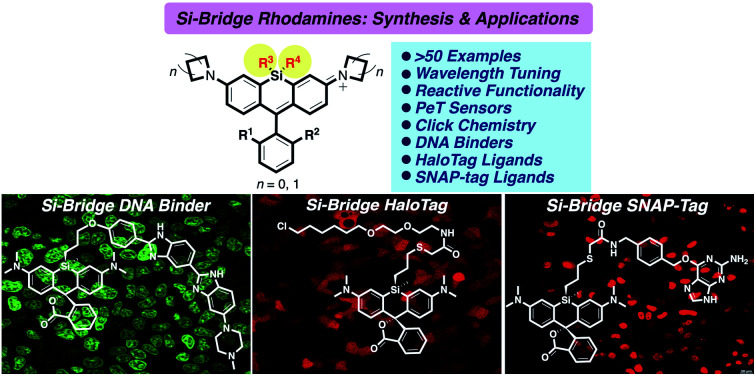

## Introduction

Siloles have long been known to exhibit red-shifted fluorescence properties compared to their analogous silicon-free heterocycles due to conjugation between the Si σ* orbitals and the π* orbitals of the chromophore.^1^ However, it wasn't until 2008 that this LUMO-lowering effect was shown to apply to long-wavelength xanthene dyes by replacing the bridging oxygen atom with a dimethylsilyl group.^2^ Si-rhodamine dyes have been widely studied and optimized ever since,^3–15^ and the effect of this modification has recently been shown to extend to other classes of near-IR dye scaffolds.^16,17^ Despite this intense interest, and the extension of the bridge-substitution strategy to other elements,^3,18–22^ virtually all Si-dyes reported thus far have been confined to dimethylsilyl substitution.^19,23,24^ Belying the nearly exclusive reliance on dimethylsilyl bridging groups amongst long-wavelength Si-dyes, a number of silyl substitutions are known within silole optical materials.^1,25–27^

**Fig. 1:**
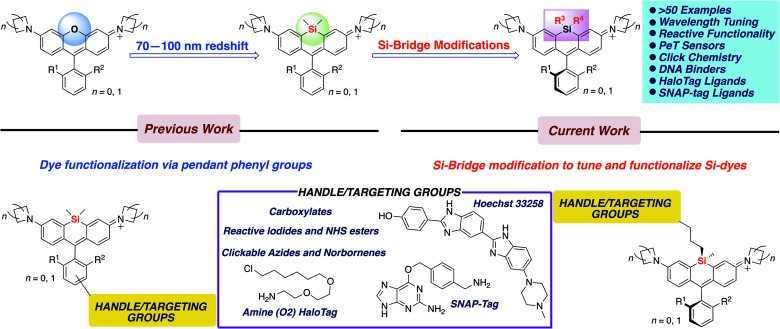
Scope of work.

The dimethylsilyl group has been ubiquitous among the Si-rhodamines reported to date presumably because of its small size and familiarity. However, more extensive modification of the silyl group – something that is not possible with oxygen-bridged rhodamines – represents a missed opportunity, as it holds great promise for tuning dye behavior and tethering of Si-dyes to sensors or biomolecules. Here we show that a wide variety of silyl modifications can be productively incorporated into Si-rhodamines. Diphenyl and divinylsilyl dyes are further red-shifted by 10–15 nm compared to the standard dimethylsilyl dyes. Chloropropyl silanes include functional handles that can be used for further elaboration into iodides, clickable azides and functionalized thioethers. Finally, we show that molecular sensors and biomolecular targeting groups can be directly incorporated into the silyl bridge (Fig. 1), enabling new ways of modulating fluorescence and applications such as no-wash labeling of the nucleus and targeted fusion proteins.

## Results and discussion

### Initial synthesis of new Si-bridge rhodamines

**Scheme 1:**
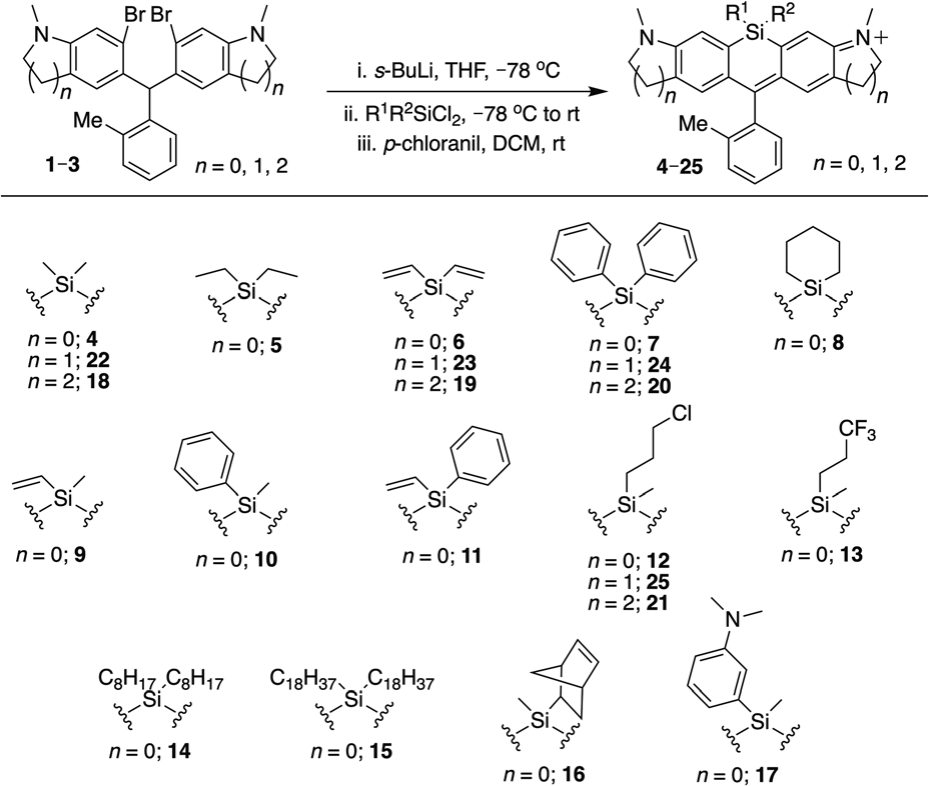
Synthesis of Si-bridge rhodamines.

To allow rapid access to novel Si-substituted dyes, we began with dibromo scaffold 1 (Scheme 1).^18^ Lithium–halogen exchange chemistry followed by reaction with different commercially available and synthesized dichlorosilanes afforded Si-leuco dyes, which were directly oxidized with *p*-chloranil to yield the desired Si-rhodamines 4–17, purified as the TFA salt (Scheme 1). Gratifyingly, most dichlorosilanes yielded the expected Si-rhodamine dyes, importantly including chloropropyl silanes with a potential handle for subsequent modification. Cyclobutyl and cyclopentyl dichlorosilanes, which form strained and likely unstable reaction products, were the exceptions.^27–29^ Asymmetrically substituted Si-dyes led to two isomers that were evident by NMR but not separated by chromatography.

### Brighter, red-shifted Si-dyes

We examined the photophysical properties of the new Si-rhodamines in aqueous buffer (Table 1). As reference, we compared these properties to the known dimethylsilyl-bridged dye SiR (4).^3,30^ We found that the quantum yield of the new dyes was largely unperturbed by Si-modification, and most of the dyes were as bright or brighter than the parent dimethylsilyl dye SiR. For example, the diethylsilyl dye 5 is 30% brighter, primarily due to a larger extinction coefficient. Whereas simple alkyl substitution on silicon did not appreciably affect the fluorescence wavelength, the introduction of vinyl and phenyl groups caused additive red-shifts in excitation and emission (Table 1). The diphenyldisilyl dye 7 is the most red-shifted, while divinylsilyl dye 6 is both red-shifted and brighter than 4 due to its higher extinction coefficient. Mixed Si-substitution with phenyl, vinyl, and methyl groups gave intermediate effects (9–11). The excitation and emission wavelengths of phenylvinylsilyl dye 11 roughly split the difference between dyes 6 and 7, and is brighter than 9, 10, and 7, but dimmer than 6. Chloropropyl silane 12 is not red-shifted, but is brighter than 4 owing to its larger extinction coefficient (Table 1). The basis for this increase is unclear, but changes of similar magnitude are known in Si-rhodamines with different amine donors and pendant phenyl groups.^9^ On the other hand, incorporation of a trifluoropropyl group into the Si-bridge (13) did not red-shift the dye and lowered brightness. Dioctylsilyl dye 14 is considerably more lipophilic than the other Si-rhodamines, exhibiting weakened fluorescence in PBS (*Φ* = 0.18), a low extinction coefficient that did not follow Beer's law, and a pronounced Rayleigh scatter peak. When the solvent was switched to EtOH, we observed bright fluorescence and no scatter peak (Table 1). Dioctadecylsilyl dye 15 was poorly soluble, and its fluorescence properties were not further characterized. Potentially, however, such long-chain aliphatic groups could be used to recruit Si-dyes to membranes.

**Table 1:** Photophysical properties of Si-bridge rhodamines

Dye	*λ*_ex_ (nm)	*λ*_em_ (nm)	*ϕ*	*ε* (M^−1^ cm^−1^)	Brightness (M^−1^ cm^−1^)
4 (SiR)	648	663	0.34	126 400	43 102
5	648	664	0.36	164 900	58 540
6	657	672	0.36	158 300	56 671
7	663	677	0.35	120 900	41 952
8	650	666	0.33	170 000	56 610
9	651	668	0.35	141 400	48 924
10	657	670	0.35	99 140	34 203
11	658	676	0.36	150 700	53 499
12	650	665	0.36	180 600	64 113
13	650	666	0.35	112 900	39 854
14	651	666	0.57^a^	156 300^a^	89 091^a^
16	649	666	0.38	160 000	60 800
17	651	668	0.02, 0.31^b^	154 200	3700
18 (SiR680)	674	692	0.31	135 000	41 850
19	683	702	0.32	234 700	75 104
20	690	710	0.28	148 300	41 524
21	677	694	0.33	125 000	41 250
22 (SiR700)	690	715	0.17	100 000	17 000
23	702	728	0.13	136 100	17 693
24	710	737	0.11	117 000	12 870
25	693	717	0.17	129 100	21 947
38	649	663	0.47	118 000	55 460
39	658	673	0.44	109 700	48 268
40	663	680	0.41	154 200	63 222
41	651	668	0.49	166 700	81 683
42	653	671	0.35	158 000	54 826
43	655	673	0.38	200 000	76 000
44	653	670	0.48	180 000	86 400
51	655	673	0.40	152 100	60 840
54	655	673	0.37	171 000	63 270

a Photophysical properties were measured in PBS except as noted: ethanol.

b Photophysical properties were measured in PBS except as noted: 100 mM sodium acetate buffer, pH 3.

### Si-substitution effects extend generally to different Si-rhodamine scaffolds

**Scheme 2:**
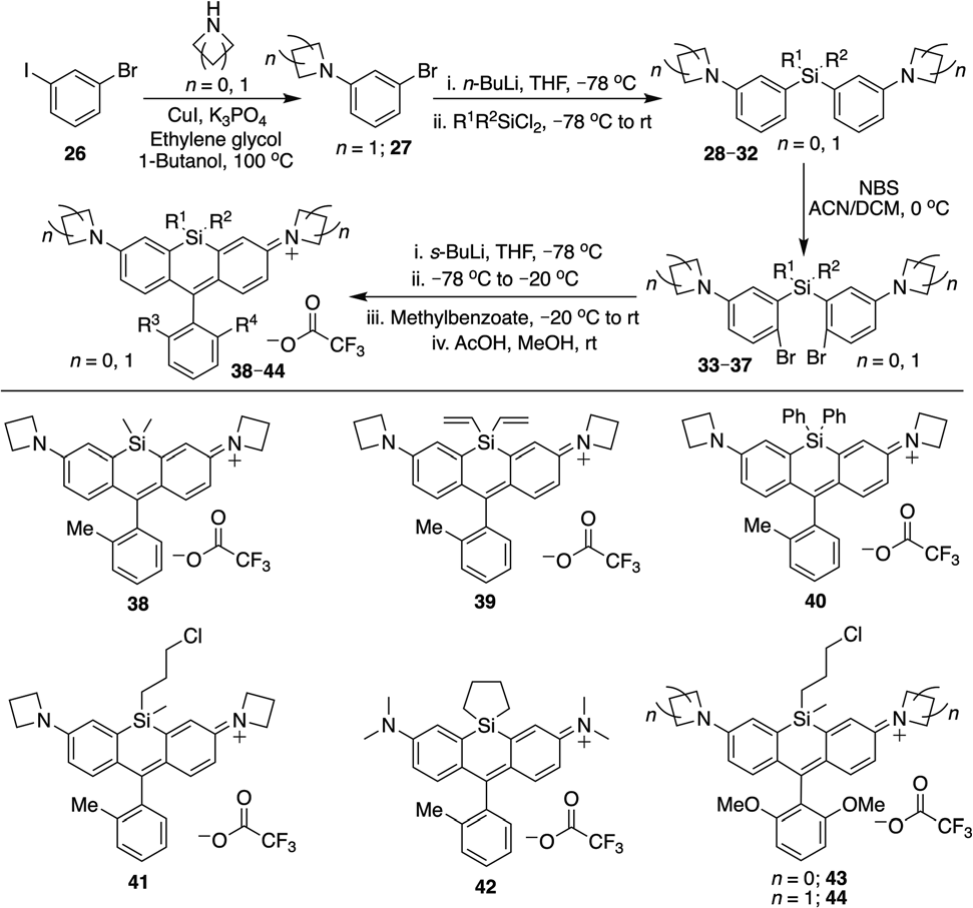
Alternative synthesis of Si-bridge rhodamines.

We next evaluated the effect of modified silyl groups in a broader range of Si-rhodamine dyes with different amine donors (Schemes 1 and 2). The previously reported dimethylsilyl dyes SiR680 (18) and SiR700 (22) incorporate rigid tetrahydroquinolines and indolines respectively, which red-shifts their fluorescence properties compared to 4.^30^ Notably, we found that the red-shifting effects of Si-modification are additive within these scaffolds. Divinylsilyl (19) and diphenylsilyl (20) SiR680 dyes are 9–18 nm further red-shifted from SiR680 (18) (Table 1). Similarly, the divinylsilyl (23) and diphenylsilyl (24) indoline SiR700 dyes are red-shifted by 12–22 nm, and 23 is brighter than the dimethylsilyl dye SiR700 (22).^30^ Among these dyes, 19 is a particular standout, as it is redshifted and 60% brighter than 18 (Table 1).

Azetidine donors are known to improve the quantum yield of rhodamines compared to dimethylamine groups,^9^*e.g.*, compare 38 (*Φ* = 0.47; Table 1) to 4 (*Φ* = 0.34; Table 1). Si-modification was well tolerated within this scaffold as well, as we found that the diphenylsilyl dye 40 and the chloropropylsilyl dye 41 are brighter than the dimethylsilyl dye 38 (Table 1). Furthermore, dyes 39 and 40 are both red-shifted compared to 38 (Table 1). We expect that other amine donors that improve quantum yields, such as thiomorpholine dioxide,^31,32^ will be similarly compatible.

### Cyclic silanes

Strain-promoted lowering of the Si σ* energy in cyclic silanes is an approach to red-shift emission that has been applied to dithienosiloles.^27^ Incorporation of spirocyclic Si-modifications within rhodamines was therefore anticipated to red-shift dye fluorescence. Treatment of 1 with cyclohexyl dichlorosilane yielded the expected Si-rhodamine dye 8 (Scheme 1). However, the reactions with the corresponding five and four-membered ring analogs yielded many side products. We were able to obtain the cyclopentyl analog 42 in good yield through an alternate synthetic pathway (Scheme 2), but the four-membered ring analog could not be isolated by either route. The silacyclopentyl analog 42 is red-shifted compared to 4 and 8, consistent with a strain-induced LUMO-lowering effect (Table 1). However, we found that 42 is chemically unstable in solution, degrading from a blue-colored near-IR dye to a red-colored red fluorescent dye along with other side-products. LC/MS and HRMS indicated an increase in mass from 425 to 457, consistent with the net addition of CH_3_OH (Fig. S1†). Our operating hypothesis is that this involves ring opening of the silacyclopentane (Fig. S1†). Chemical instability has previously been noted for spirocyclic siloles,^28^ due in part to this same strain-induced LUMO-lowering effect,^27^ particularly for the four-membered rings.^29^

### Introduction of sensors and functional handles

A general strategy used to develop fluorescent sensors is photo-induced electron transfer (PeT).^3,33^ Such sensors have typically been incorporated into the pendant phenyl group of rhodamines (Fig. 1). We investigated whether a PeT sensor could be introduced directly onto the Si atom of a Si-rhodamine. Dye 17 (Scheme 1) was anticipated to exhibit quenched dye fluorescence at physiological pH, but become brightly fluorescent at acidic pH when the aryl amine becomes protonated. The quantum yield in PBS at pH 7.4 is <0.02 (Table 1), consistent with PeT quenching when the aryl amine is not protonated. Conversely, the quantum yield in pH 3 acetate buffer is 0.31, consistent with relief of PeT quenching when the aryl amine is protonated (Table 1).

Silyl modification could also be used to introduce handles for attachment of dyes to sensors or biomolecules. A commercially-available dichlorosilane includes a norbornene group, which could potentially be used for Inverse-Electron Demand Diels–Alder (IEDDA) click chemistry with tetrazines.^34^ We synthesized norbornylsilyl dye 16 as a mixture of four isomers (exo/endo norbornene and two atropisomers), and found that the norbornene dye isomers react with methyl tetrazine NHS ester under mild conditions (Scheme S1†). Although the NMR was difficult to interpret due to the presence of multiple isomers, HRMS indicated that the expected cycloaddition products are formed, consistent with the oxidized aromatic pyridazine product rather than the dihydropyridazine (Scheme S1†).

**Scheme 3:**
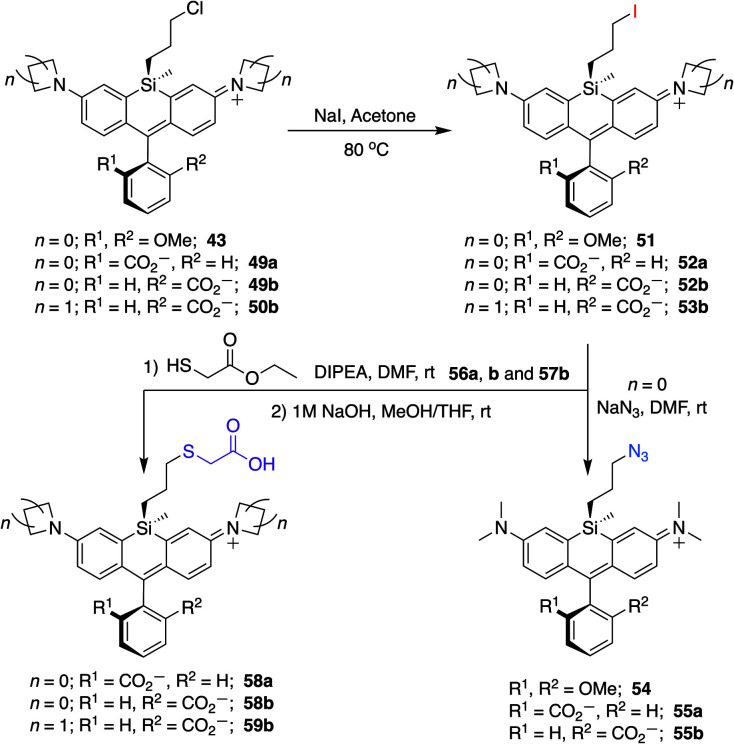
Conversion of chloropropylsilyl Si-bridge dyes into iodo, azido, and thiol-substituted Si-rhodamines.

To further explore the scope of diverse functional attachment *via* the bridging silane, we substituted the chloride in 12 with iodide to make the corresponding iodopropyl dye (12-I, Scheme S2†). This electrophilic dye could potentially be used in subsequent reactions with nucleophiles. However, we found that treatment of 12-I with the small nucleophile azide resulted in both iodide displacement and reaction at the central carbon of the rhodamine scaffold, a known site of nucleophilic attack.^35^ We therefore synthesized chloropropylsilyl dyes 43 and 44 in a 2,6-dimethoxy scaffold (Scheme 2), as symmetrical substitution of rhodamines at both *ortho* positions of the pendant phenyl sterically shields nucleophilic attack at the central carbon.^20,35^ Displacement of the chloride in 43 with iodide formed iodopropylsilyl dye 51, which could be further elaborated to the azide 54 (Scheme 3). Interestingly, however, the azetidine dye 44 was labile to excess iodide, which resulted in tri-iodination *via* displacement of the chloro group and ring-opening of both azetidines (Fig. S2†).

### Si-modifications in spirolactone dyes

Si-rhodamine dyes that can spirolactonize are valuable for live cell imaging, as the spirolactone form is nonfluorescent and cell permeable, whereas the zwitterionic form is brightly fluorescent and can selectively form when bound to particular target biomolecules (Fig. S3†). Notable examples include SiTMR (45)^5^ and JF646 (46).^9^ These dyes exist primarily in the spirolactone form in aqueous buffer, with *K*_L–Z_ lactone–zwitterion equilibrium values^36^ of 0.0034 and 0.002. Estimation of the maximal extinction coefficient when ring-opened has been reported using EtOH/0.1% TFA (Table 2).^9^

**Table 2:** Photophysical properties of spirolactones

Dye	*λ*_ex_ (nm)	*λ*_em_ (nm)	*ϕ*	*ε*_max_ a (M^−1^ cm^−1^)
45 (SiTMR)^b^	643	662	0.41	141 000
46 (JF646)^b^	646	664	0.52	152 000
47	656^c^	675^c^	ND	80 000
48	664^c^	681^c^	ND	80 000
49a	645	665	0.40	206 900
49b	645	665	0.40	200 200
50a	648	666	ND	183 000
50b	648	666	0.53	213 200
52a	645	665	0.43	115 000
52b	645	665	0.45	144 000
55a	645	666	0.43	206 700
55b	645	665	0.44	218 400
56a	645	665	0.45	145 800
56b	646	665	0.45	150 700
58a	645	665	0.45	180 600
58b	645	665	0.45	160 400

a Photophysical properties were measured in PBS except as noted: 95%EtOH/0.1%TFA.

b Photophysical properties were measured in PBS except as noted: values from ref. 9.

c Photophysical properties were measured in PBS except as noted: 0.1% SDS/PBS.

**Scheme 4:**
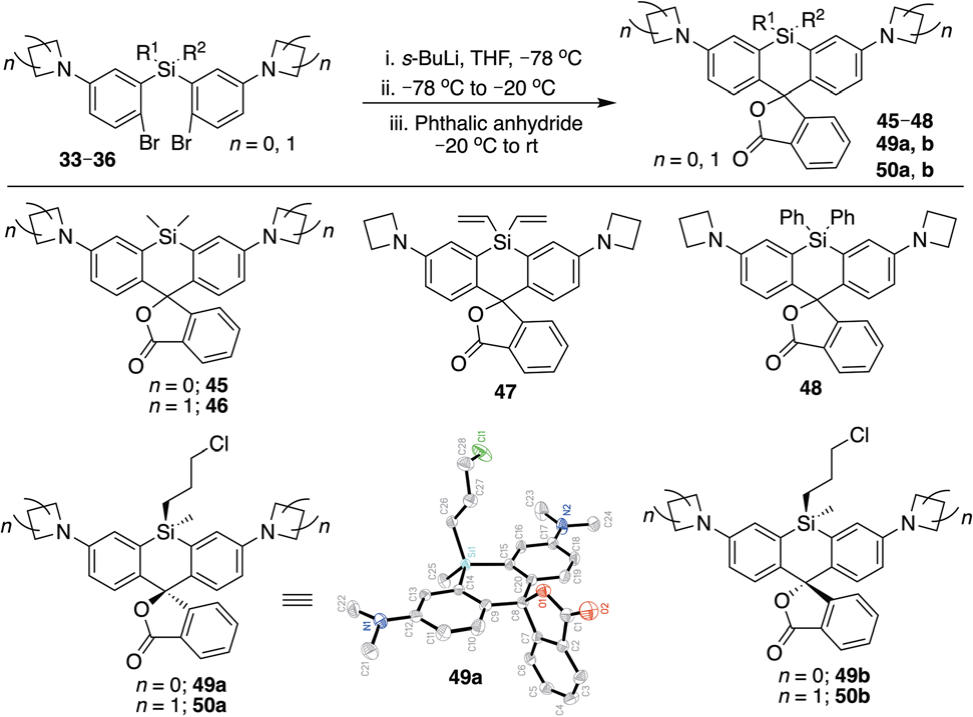
Synthesis of Si-bridge spirolactones. Small molecule X-ray crystal structure of 49a reveals that it is the “s,s” isomer, where the silyl chloropropyl group and the phenyl of the spirolactone are on opposite faces of the planar chromophore.

We synthesized Si-rhodamine spirolactones with diphenyl, divinyl, and chloropropyl silyl bridging groups for comparison to the respective dimethylsilyl spirolactones (Scheme 4). Divinylsilyl and diphenylsilyl spirolactones in the azetidine series (47, 48) are red-shifted but give relatively low extinction coefficient maximum values in 95% EtOH/0.1% TFA of ∼80 000 M^−1^ cm^−1^ (Table 2) which may indicate that the dyes strongly favor the spirolactone form. Using anhydrous EtOH/0.1% TFA increased these values to ∼124 000 and 144 000, respectively. We did not attempt to measure aqueous quantum yields as these spirolactones did not appreciably absorb in PBS alone, and the *K*_L–Z_ lactone–zwitterion equilibrium value for 47 is 0.00125. Because dimethylamine-donor dyes such as SiTMR (45) are known to adopt the open zwitterionic form more than azetidine-donor spirolactones like JF646 (46) (Scheme 4 and Table 2),^9^ and the azetidine in dye 44 is labile to iodide, we chose to evaluate the chloropropylsilyl modification with dimethylamine donors.

The synthesis of chloropropylsilyl spirolactones yielded two separable isomeric products (49a and 49b, Scheme 4). Both have a quantum yield of 0.40 in PBS, the extinction coefficient in 95% EtOH/0.1%TFA for both is ∼200 000 M^−1^ cm^−1^ (Table 2), and the *K*_L–Z_ lactone–zwitterion equilibrium value for 49b is 0.0045. We therefore anticipate that the open-closed equilibrium of spirolactones like 47 and 48 can be further tuned and optimized by modification of the amine donor and/or the acidity of the spirolactone leaving group.^9,37^

### Structure and synthetic elaboration of Si-bridge rhodamine spirolactones

Unlike asymmetric Si-substituted cationic Si-rhodamines such as 9–13 (Scheme 1), we found that asymmetric Si-substituted spirolactones (Scheme 4) were readily separable by flash chromatography. Small molecule X-ray crystallography of 49a revealed that it is the “s,s” isomer, where the chloropropyl group and the phenyl of the spirolactone are on opposite faces of the planar dye (Scheme 4). We were unable to obtain an X-ray structure of 49b, but the HRMS and NMR are consistent with the presentation of the silyl substituents as the “r,r” isomer.

**Fig. 2:**
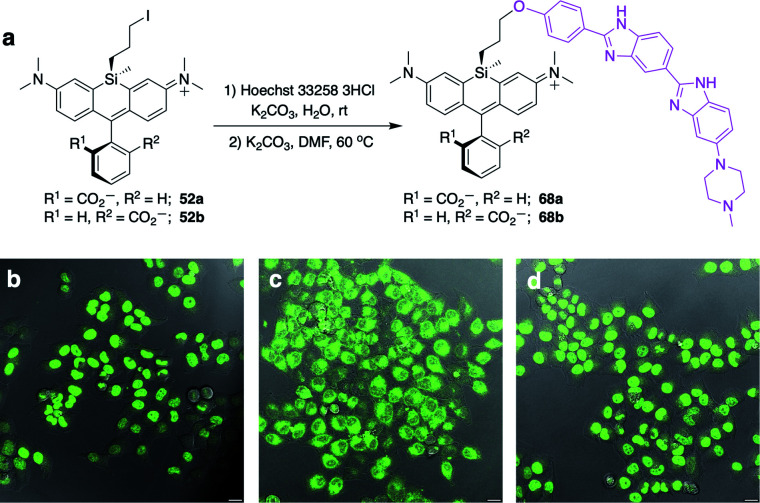
Synthesis and no-wash live cell imaging with Si-bridge dyes 68a–b. (a) Synthesis of 68a–b from Si-iodopropyl dyes and Hoechst 33258; (b–d) Overlay of fluorescence and DIC in live HeLa cells treated with 1 μM (b) SiR-DNA; (c) 68a; (d) 68b. Scale bar is 20 μm, fluorescence pseudo-colored green.

**Fig. 3:**
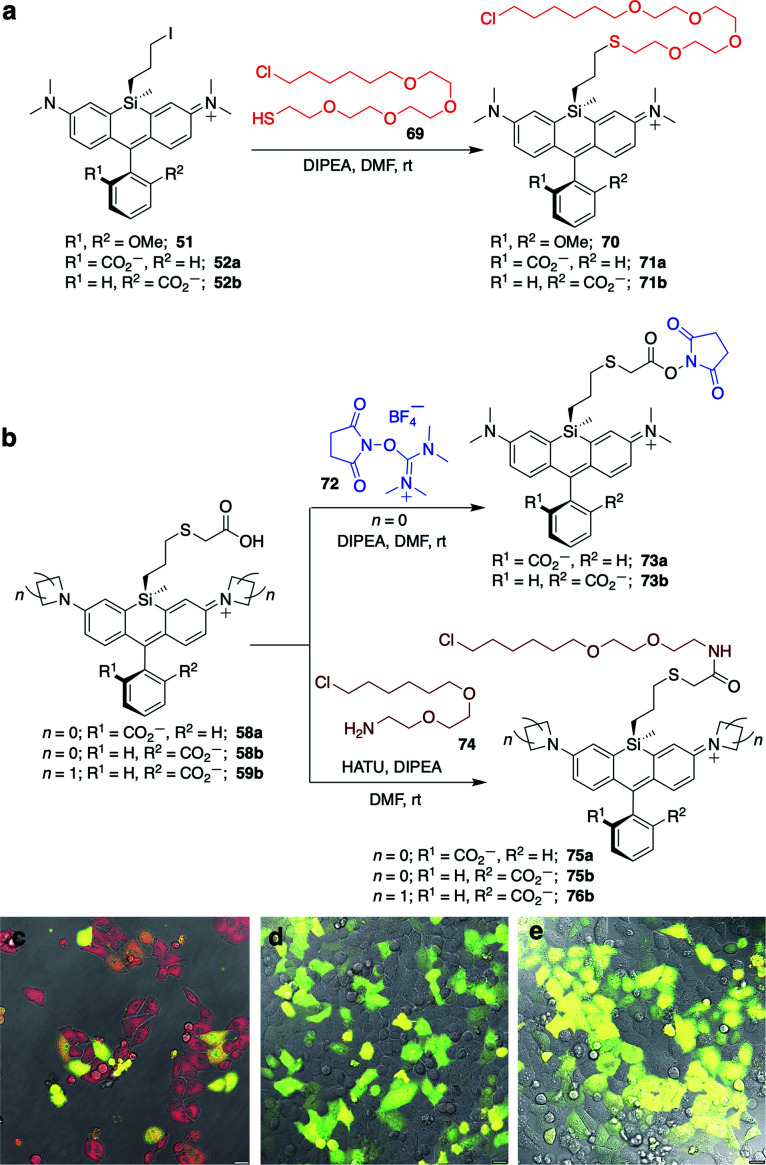
Synthesis of Si-bridge NHS esters, HaloTag-ligand dyes, and no-wash live cell imaging in HeLa cells transfected with pHaloTag-EGFP. (a) Thio (O4) HaloTag ligand dyes 70–71b; (b) Si-bridge NHS esters 73a–b and amine (O2) HaloTag ligand dyes 75a–76b; (c–e) Overlay of GFP, DIC, and dye fluorescence in cells treated with 200 nM (c) 75a; (d) 75b; (e) 76b. Scale bar is 20 μm.

**Fig. 4:**
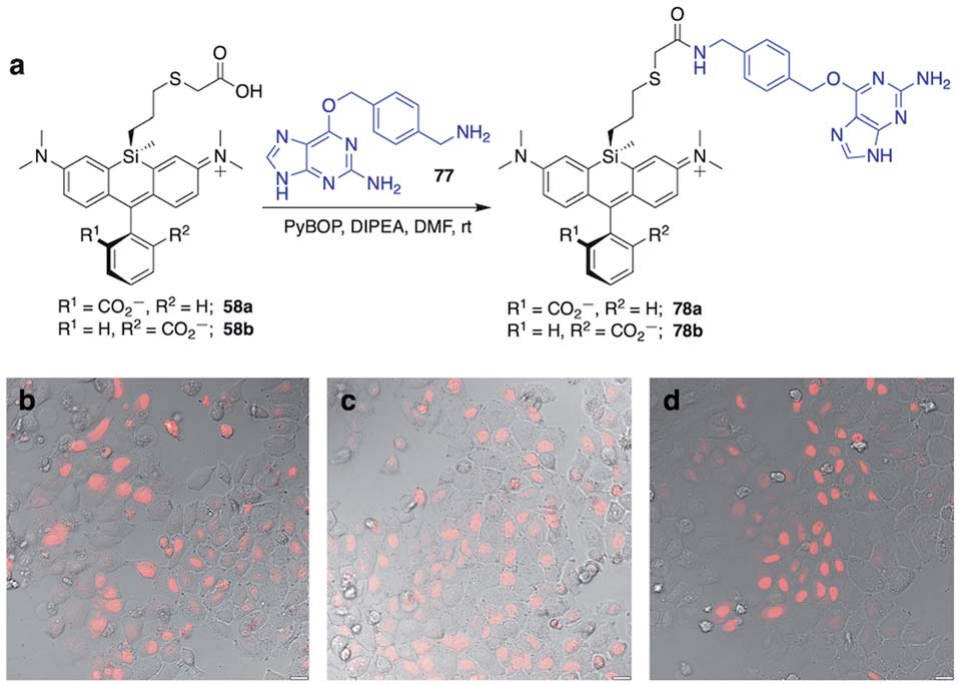
Synthesis of Si-bridge dyes 78a–b and their use for live-cell imaging of HeLa cells transfected with pSNAPf-H2B. (a) Synthesis of 78a–b by the reaction of 58a–b with O^6^-benzylguanidine coupling partner 77; (b–d) Overlay of dye fluorescence and DIC for cells treated with 3 μM (b) SNAP-Cell 647-SiR, (c) 78a, or (d) 78b. Scale bar is 20 μm.

Displacement of the chloro group in 49a and 49b with iodide afforded the corresponding isomeric iodopropylsilyl dyes 52a and 52b (Scheme 3), which could be further elaborated by reaction with a wide variety of nucleophiles. Azide-functionalized dyes 55a and 55b were readily synthesized (Scheme 3), and reacted rapidly with strained dibenzocyclooctyne (DBCO) reagents in copper-free click chemistry reactions (Scheme S1†).^38,39^ Reaction of 52a–b with the phenol of Hoechst 33258 allowed direct incorporation of this DNA-binding dye (68a–b, Fig. 2). Additional Si-bridge elaboration allowed introduction of an NHS ester (Fig. 3), chloroalkane ligands for the protein HaloTag^40^ (Fig. 3), and O^6^-benzylguanine ligands for SNAP-tag^41^ (Fig. 4). These reagents all hold considerable potential for labelling of biomolecules with bright, photostable near-IR dyes, and we anticipate that Si-iodopropylsilyl dyes can be readily elaborated with many other nucleophiles.

Chloropropylsilyl spirolactones in the azetidine series also yielded two separable isomers (50a–b, Scheme 4). In contrast to cationic azetidine dye 44, we found that displacement of the chloro group in the azetidine dye 50b with iodide was facile, yielding 53b (Scheme 3) and allowing entry to Si-bridge spirolactone dyes with azetidine donors. We presume that azetidine ring-opening by iodide observed for 44 is facilitated by the partial positive charge on the azetidines in the cationic dye form, but not in the neutral spirolactone 50b.

### No-wash live cell imaging of the nucleus with a Si-bridge dye

Fluorogenic spirolactonizable Si-rhodamines are particularly valuable for no-wash live cell imaging, as the neutral cell-permeable form can convert to a highly fluorescent form when bound to particular targets, such as DNA^42^ or the protein HaloTag.^5^ This fluorogenic response occurs when the nonfluorescent spirolactone ring opens, generating a zwitterionic dye. To date, all examples of fluorogenic Si-rhodamine dyes have been modified with targeting groups directly on the pendant phenyl ring that forms the spirolactone (Fig. 1). *A priori*, it was not obvious whether this same fluorogenic behavior would also occur with dyes that are modified with targeting groups on the more distal silyl group.

SiR-DNA is a commercially-available Si-rhodamine dye with the DNA-targeting ligand Hoechst 33258 attached to the pendant phenyl ring, allowing specific labeling of the nucleus in live cells (Fig. S4†).^42^ We synthesized two Hoechst 33258-modified Si-bridge isomers *via* direct reaction of the phenol of Hoechst 33258 with the isomeric Si-iodopropyl dyes 52a and 52b, and then assessed their ability to label the nucleus in live HeLa cells compared to SiR-DNA (Fig. 2 and S5†).

We found that 68b is highly fluorogenic, labelling only the nucleus in live cells (Fig. 2d). This importantly demonstrates that the valued fluorogenic effect seen in traditional Si-rhodamine dyes modified on the pendant phenyl ring can also translate to Si-rhodamine dyes modified on the silyl group. Interestingly, however, the isomeric compound 68a labelled internal membranes and yielded no labelling of the nucleus (Fig. 2c). To gain more insight into the molecular basis for this difference, we evaluated the photophysical properties of 68a–b*in vitro* in the presence and absence of a hairpin DNA oligonucleotide (hpDNA).^43^ Like SiR-DNA, these dyes are quenched and virtually nonfluorescent in PBS, with quantum yields <0.02 and brightness of <130 M^−1^ cm^−1^ (ESI†). In the presence of 30 μM hpDNA, the extinction coefficient of 68b increases to 26 000 M^−1^ cm^−1^ and the quantum yield to 0.40, yielding a brightness of 10 400 M^−1^ cm^−1^ compared to 8518 M^−1^ cm^−1^ for SiR-DNA, whereas 68a exhibits little turn-on response (550 M^−1^ cm^−1^). Isomeric dyes modified at different ring positions on the pendant phenyl have also been shown to differ in their behaviour toward biomolecules, including their interaction with DNA,^43^ HaloTag and SNAP-tag.^44^ Investigation of the reasons for this difference in Si-bridge dyes, where there is control over which “face” of the dye is directed toward the target and/or sensor, could enable a more detailed understanding of the underlying principles for specific fluorogenic probe design.

### No-wash live cell imaging of HaloTag-expressing cells with a Si-bridge dye

The fluorogenic behavior of 68b for DNA labeling suggested that fluorogenic probes for other valuable classes of live cell targets could be developed. HaloTag presents an anionic surface that is known to favor spirolactone dye ring-opening.^44^ Our operating hypothesis is that favorable biomolecular interaction with the ring-opened cationic dye will occur only when the anionic carboxylate is facing away from the protein surface (Fig. S6†). Thus, we predicted that fluorogenic Si-bridge dyes targeting HaloTag will also favor r,r isomer b, with the Si-tether and the carboxylate on opposite faces of the dye. To test this supposition, we transiently transfected HeLa cells with pHaloTag-EGFP^45^ and treated the cells with 200 nM of six Si-bridge dyes modified with chloroalkane ligands for HaloTag (70, 71a, 71b, 75a, 75b, and 76b in Fig. 3). As anticipated, the r,r isomer 75b and the corresponding azetidine analog 76b are fluorogenic and labeling correlates with GFP expression (Fig. 3 and S7†). Dye 71b also labeled HaloTag-EGFP-expressing cells to some extent but had significant background (Fig. S8†), suggesting that nonspecific interactions can also shift its spirolactone equilibrium to the open form. On the other hand, the s,s isomers 75a and 71a did not label HaloTag-EGFP-expressing cells, and the symmetric cationic dye 70 only stained mitochondria, suggesting that sequestration by the negatively polarized mitochondrial membrane dominates its behavior in cells (Fig. S8†).

### Labeling of SNAP-tag-expressing cells with Si-bridge dyes

SNAP-tag is another popular system for labeling fusion proteins.^41^ SNAP-tag is more promiscuous towards its substrates than HaloTag,^44^ and thus we anticipated that the relative fluorogenic behavior for each Si-bridge dye isomer would differ from the stark facial selectivity results seen above with Hoechst probes and HaloTag. Two Si-bridge dye isomers with an O^6^-benzylguanine SNAP-tag ligand attached to either face of the dye (78a–b, Fig. 4) were compared to the commercial dye SNAP-Cell 647-SiR, which has a SNAP-tag ligand attached to the pendant phenyl (Fig. S4†). All three dyes labeled the nucleus in HeLa cells transfected with pSNAPf-H2B (Fig. 4 and S9†). Thus SNAP-tag labeling is tolerant of attachment on either face of the dye *via* the Si-bridge as well as conventional positioning *via* the pendant phenyl.

## Conclusion

Replacement of the bridging oxygen atom in xanthene dyes with a dimethylsilyl group has had a tremendous effect on biological imaging methods since its first report over 12 years ago. Yet, more extensive modification of the silyl group has thus far remained untapped. Here we have synthesized a broad range of novel Si-modified Si-rhodamine dyes that are often brighter and/or more red-shifted than the corresponding conventional dimethylsilyl dyes. Diverse functionality can be incorporated at the bridging silicon, allowing elaboration into a wealth of dyes derivatized through the Si-bridge, including molecular sensors, thiol-reactive iodides, clickable azides, amine-reactive NHS esters, and dyes with specific protein-binding or DNA-targeting ligands. We demonstrate that the fluorogenic behavior of spirolactonizable Si-rhodamine dyes can be retained in Si-bridge dyes and utilized for applications such as no-wash live cell imaging. Moreover, the ability to add sensors and targeting groups to the Si-bridge of Si-rhodamines in addition to conventional modification of the pendant phenyl opens up new possibilities in sensor design, including dual sensors, probes with both sensor and targeting groups, and different facial display of these functionalities. Modification of the bridging group as described herein is not possible with conventional oxygen-bridged xanthene dyes, but could be similarly applied to dyes with bridging elements such as carbon, germanium and phosphorus.^46^ We anticipate that Si-modification will have broad applications within Si-xanthenes and spur the development of other classes of related dyes and functionalized materials.

## Data availability

All of the experimental data is in the ESI.† The X-ray data has been deposited in the appropriate repository.

## Author contributions

D. N. R. synthesized all compounds. S. C. M. measured photophysical properties. X. J. performed cell imaging. D. N. R. and S. C. M. wrote the manuscript. S. C. M. conceived and supervised the study.

## Conflicts of interest

The authors declare the following competing financial interest: D. N. R and S. C. M. have filed a patent application on Si-bridge dyes.

## Supplementary Material

SC-013-D2SC01821G-s001

SC-013-D2SC01821G-s002

## References

[cit1] Yamaguchi S., Jin R.-Z., Tamao K. (1998). J. Organomet. Chem..

[cit2] Fu M., Xiao Y., Qian X., Zhao D., Xu Y. (2008). Chem. Commun..

[cit3] Koide Y., Urano Y., Hanaoka K., Terai T., Nagano T. (2011). ACS Chem. Biol..

[cit4] Wang T., Zhao Q.-J., Hu H.-G., Yu S.-C., Liu X., Liu L., Wu Q.-Y. (2012). Chem. Commun..

[cit5] Lukinavičius G., Umezawa K., Olivier N., Honigmann A., Yang G., Plass T., Mueller V., Reymond L., Corrêa Jr I. R., Luo Z.-G., Schultz C., Lemke E. A., Heppenstall P., Eggeling C., Manley S., Johnsson K. (2013). Nat. Chem..

[cit6] Myochin T., Hanaoka K., Iwaki S., Ueno T., Komatsu T., Terai T., Nagano T., Urano Y. (2015). J. Am. Chem. Soc..

[cit7] Ikeno T., Nagano T., Hanaoka K. (2017). Chem.–Asian J..

[cit8] Kolmakov K., Hebisch E., Wolfram T., Nordwig L. A., Wurm C. A., Ta H., Westphal V., Belov V. N., Hell S. W. (2015). Chem. –Eur. J..

[cit9] Grimm J. B., Brown T. A., Tkachuk A. N., Lavis L. D. (2017). ACS Cent. Sci..

[cit10] Wirth R., Gao P., Nienhaus G. U., Sunbul M., Jäschke A. (2019). J. Am. Chem. Soc..

[cit11] Tyson J., Hu K., Zheng S., Kidd P., Dadina N., Chu L., Toomre D., Bewersdorf J., Schepartz A. (2021). ACS Cent. Sci..

[cit12] Uno S., Kamiya M., Yoshihara T., Sugawara K., Okabe K., Tarhan M. C., Fujita H., Funatsu T., Okada Y., Tobita S., Urano Y. (2014). Nat. Chem..

[cit13] Xiao Y., Qian X. (2020). Coord. Chem. Rev..

[cit14] Huang Y.-L., Walker A. S., Miller E. W. (2015). J. Am. Chem. Soc..

[cit15] Fischer C., Sparr C. (2018). Angew. Chem., Int. Ed..

[cit16] Choi A., Miller S. C. (2018). Org. Lett..

[cit17] Pengshung M., Neal P., Atallah T. L., Kwon J., Caram J. R., Lopez S. A., Sletten E. M. (2020). Chem. Commun..

[cit18] Zhou X., Lai R., Beck J. R., Li H., Stains C. I. (2016). Chem. Commun..

[cit19] Zhou X., Lesiak L., Lai R., Beck J. R., Zhao J., Elowsky C. G., Li H., Stains C. I. (2017). Angew. Chem., Int. Ed. Engl..

[cit20] Grzybowski M., Taki M., Senda K., Sato Y., Ariyoshi T., Okada Y., Kawakami R., Imamura T., Yamaguchi S. (2018). Angew. Chem., Int. Ed..

[cit21] Liu J., Sun Y.-Q., Zhang H., Shi H., Shi Y., Guo W. (2016). ACS Appl. Mater. Interfaces.

[cit22] Chai X., Cui X., Wang B., Yang F., Cai Y., Wu Q., Wang T. (2015). Chemistry.

[cit23] Nie H., Qiao L., Yang W., Guo B., Xin F., Jing J., Zhang X. (2016). J. Mater. Chem. B.

[cit24] Liu J., Liu M., Zhang H., Guo W. (2021). Angew. Chem., Int. Ed..

[cit25] Cai Y., Qin A., Tang B. Z. (2017). J. Mater. Chem. C.

[cit26] Chen J., Law C. C. W., Lam J. W. Y., Dong Y., Lo S. M. F., Williams I. D., Zhu D., Tang B. Z. (2003). Chem. Mater..

[cit27] Son H.-J., Han W.-S., Chun J.-Y., Lee C.-J., Han J.-I., Ko J., Kang S. O. (2007). Organometallics.

[cit28] Son H.-J., Han W.-S., Chun J.-Y., Kwon S.-N., Ko J., Kang S. O. (2008). Organometallics.

[cit29] Huang H., Youn J., Ponce Ortiz R., Zheng Y., Facchetti A., Marks T. (2011). Chem. Mater..

[cit30] Koide Y., Urano Y., Hanaoka K., Piao W., Kusakabe M., Saito N., Terai T., Okabe T., Nagano T. (2012). J. Am. Chem. Soc..

[cit31] Sharma D. K., Adams S. T., Liebmann K. L., Miller S. C. (2017). Org. Lett..

[cit32] Lv X., Gao C., Han T., Shi H., Guo W. (2020). Chem. Commun..

[cit33] Miura T., Urano Y., Tanaka K., Nagano T., Ohkubo K., Fukuzumi S. (2003). J. Am. Chem. Soc..

[cit34] Devaraj N. K., Weissleder R., Hilderbrand S. A. (2008). Bioconjugate Chem..

[cit35] Kryman M. W., Schamerhorn G. A., Yung K., Sathyamoorthy B., Sukumaran D. K., Ohulchanskyy T. Y., Benedict J. B., Detty M. R. (2013). Organometallics.

[cit36] Zheng Q., Ayala A. X., Chung I., Weigel A. V., Ranjan A., Falco N., Grimm J. B., Tkachuk A. N., Wu C., Lippincott-Schwartz J., Singer R. H., Lavis L. D. (2019). ACS Cent. Sci..

[cit37] Wang L., Tran M., D'Este E., Roberti J., Koch B., Xue L., Johnsson K. (2020). Nat. Chem..

[cit38] Agard N. J., Prescher J. A., Bertozzi C. R. (2004). J. Am. Chem. Soc..

[cit39] Debets M. F., van Berkel S. S., Schoffelen S., Rutjes F. P. J. T., van Hest J. C. M., van Delft F. L. (2010). Chem. Commun..

[cit40] Los G. V., Encell L. P., McDougall M. G., Hartzell D. D., Karassina N., Zimprich C., Wood M. G., Learish R., Ohana R. F., Urh M., Simpson D., Mendez J., Zimmerman K., Otto P., Vidugiris G., Zhu J., Darzins A., Klaubert D. H., Bulleit R. F., Wood K. V. (2008). ACS Chem. Biol..

[cit41] Keppler A., Gendreizig S., Gronemeyer T., Pick H., Vogel H., Johnsson K. (2003). Nat. Biotechnol..

[cit42] Lukinavičius G., Blaukopf C., Pershagen E., Schena A., Reymond L., Derivery E., Gonzalez-Gaitan M., D'Este E., Hell S. W., Wolfram Gerlich D., Johnsson K. (2015). Nat. Commun..

[cit43] Bucevičius J., Keller-Findeisen J., Gilat T., Hell S. W., Lukinavičius G. (2019). Chem. Sci..

[cit44] Wilhelm J., Kühn S., Tarnawski M., Gotthard G., Tünnermann J., Tänzer T., Karpenko J., Mertes N., Xue L., Uhrig U., Reinstein J., Hiblot J., Johnsson K. (2021). Biochemistry.

[cit45] Ebner M., Lučić I., Leonard T. A., Yudushkin I. (2017). Mol. Cell.

[cit46] Ogasawara H., Tanaka Y., Taki M., Yamaguchi S. (2021). Chem. Sci..

