# Supplementary material for: Silicon functionalization expands the repertoire of Si-rhodamine fluorescent probes
Source: Chem Sci. 2022 May 2;13(20):6081–8. doi: 10.1039/d2sc01821g (PMC9132037; doi:10.1039/d2sc01821g)
Supplement: SC-013-D2SC01821G-s001 [file SC-013-D2SC01821G-s001.pdf]

Electronic Supplementary Material (ESI) for Chemical Science.
This journal is © The Royal Society of Chemistry 2022

Supporting Information

Silicon Substitution Expands the Repertoire of Si-Rhodamine Fluorescent Probes

Desaboini Nageswara Rao, Xincai Ji, and Stephen C. Miller*

Department of Biochemistry and Molecular Biotechnology, University of Massachusetts Chan Medical School, Worcester, Massachusetts 01605, United States

*Corresponding author e-mail: stephen.miller@umassmed.edu

Contents:

1) Supplementary Schemes S1-S2 and Figures S1-S9	S3–S11
2) Crystal data and structure refinement for **49a**	S12
3) Photophysical properties of Si-Bridge dyes	S13–S19
4) General experimental information	S19–S22
5) Synthesis of intermediates	S23–S26
6) Synthesis of Si-Bridge rhodamines	S26–S44
7) Alternative synthesis of Si-Bridge rhodamines	S45–S57
8) Synthesis of Si-Bridge spirolactones	S58–S63
9) Unexpected tri-iodination of **44**	S63
10) Introduction of handles	S64–S73
11) Applications of clickable dyes with tetrazene and DBCO reagents	S73–S79
12) Synthesis of Si-Bridge Hoechst dyes	S80–S82
13) Synthesis of thiol (O4) HaloTag ligand	S82–S85
14) Synthesis of Si-Bridge thiol (O4) HaloTags	S85–S87
15) Synthesis of Si-Bridge NHS esters	S88–S89
16) Synthesis of amine (O2) HaloTag ligand	S89–S91
17) Synthesis of Si-Bridge amine (O2) HaloTags	S91–S93
18) Synthesis of Si-Bridge SNAP-Tags	S94–S95
19) References	S96

Supplementary Schemes and Figures

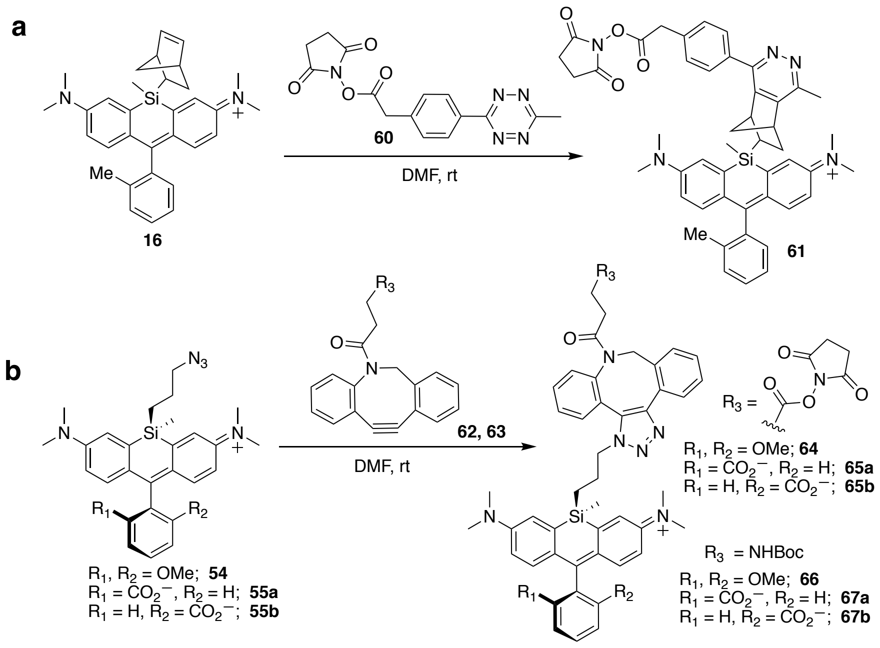

Scheme S1. Applications of clickable dyes with methyltetrazine NHS ester and DBCO NHS ester/NHBoc reagents.

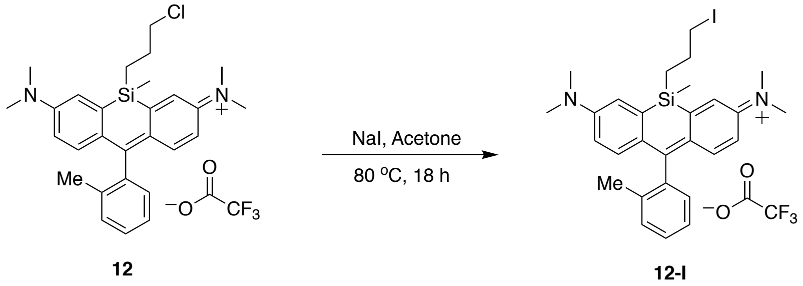

Scheme S2. Synthesis of **12-I** from corresponding chloropropyl dye **12** with NaI.

a

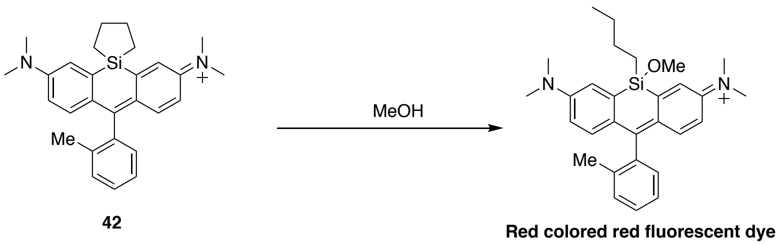

b

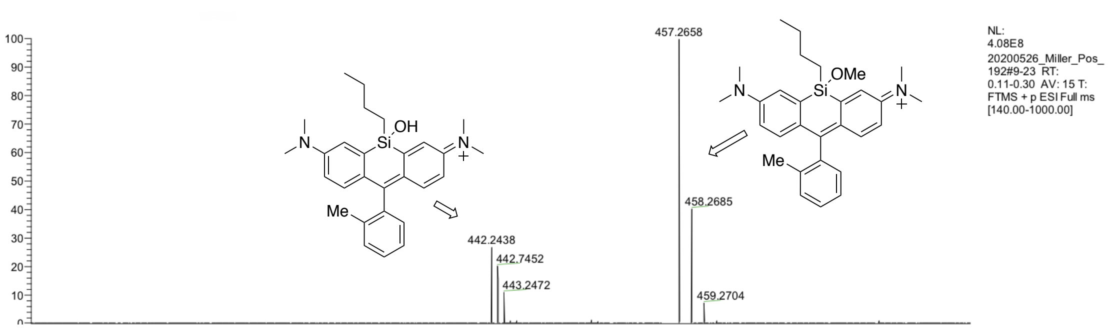

Figure S1. Instability of silacyclopentyl dye **42** in solution. (a) Proposed ring-opened product in methanol (the solvent used for HRMS analysis). (b) HRMS is consistent with ring-opening by methanol and water.

a

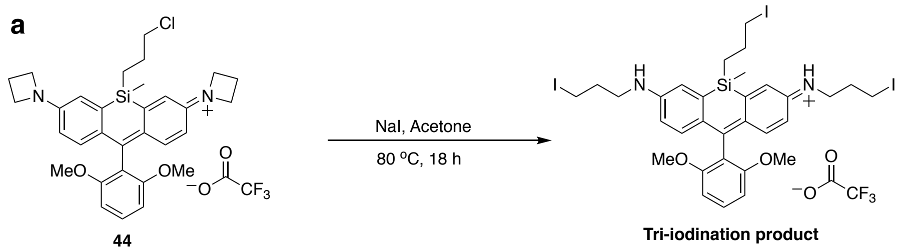

b

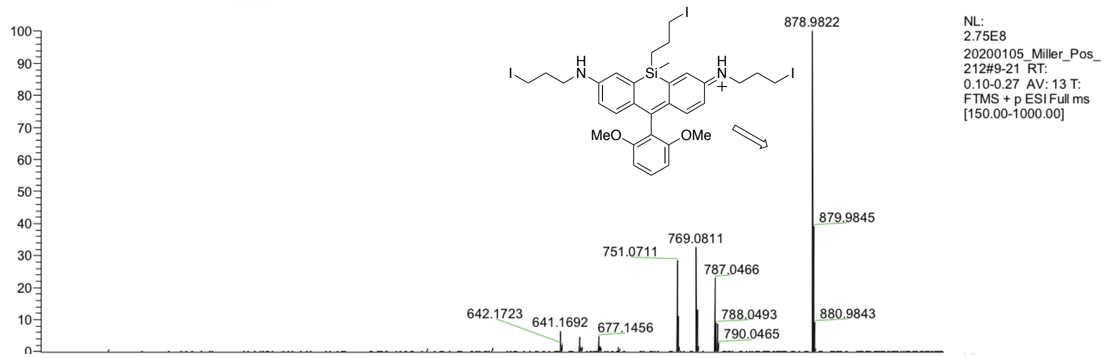

c

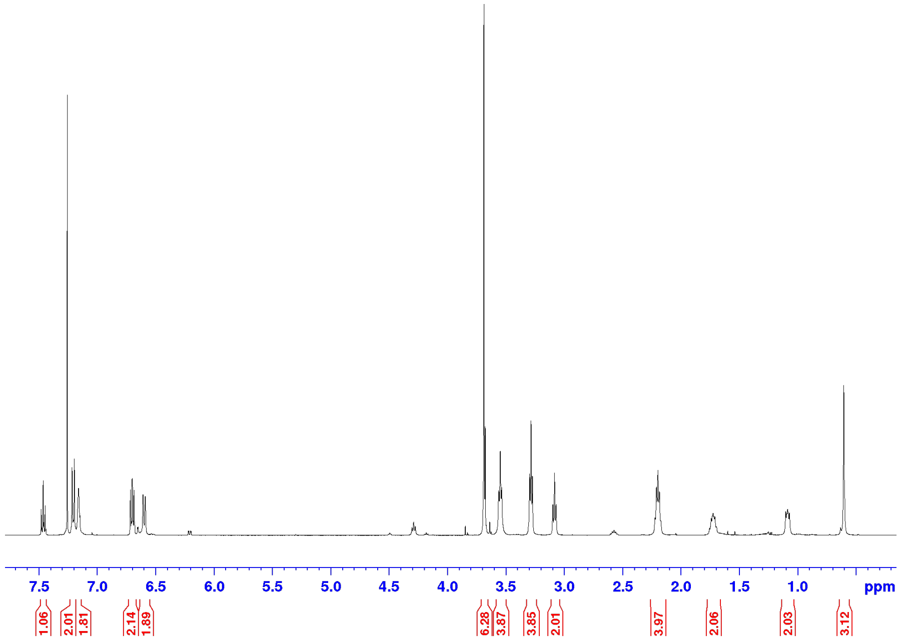

Figure S2. Unexpected tri-iodination of **44** via displacement of the chloro group and ring-opening of the azetidine rings with iodide. (a) Formation of tri-iodinated product by the treatment of **44** with excess NaI; (b) HRMS of the tri-iodination product; (c) ^{1}H-NMR of the tri-iodination product.

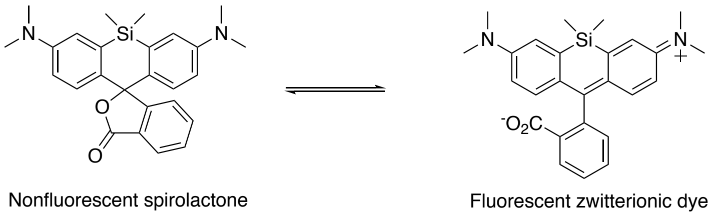

Figure S3. Spirolactone-zwitterion equilibrium.

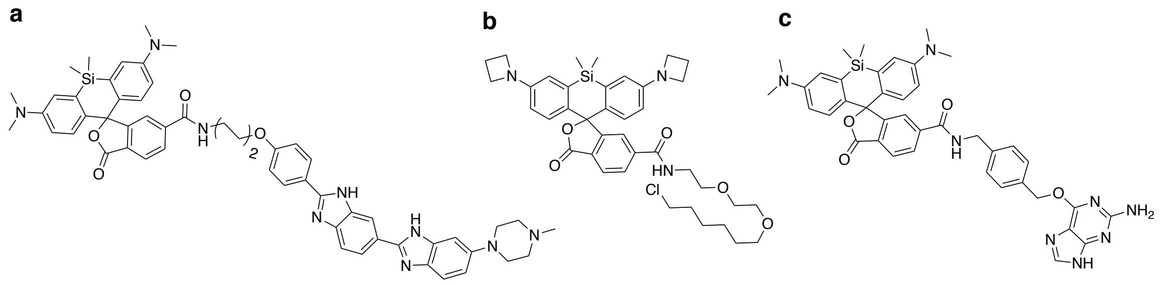

Figure S4. Structures of commercially available fluorogenic Si-rhodamine dyes modified with targeting groups on the pendant phenyl ring. (a) SiR-DNA; (b) JF646-HaloTag ligand; (c) SNAP-Cell 647-SiR.

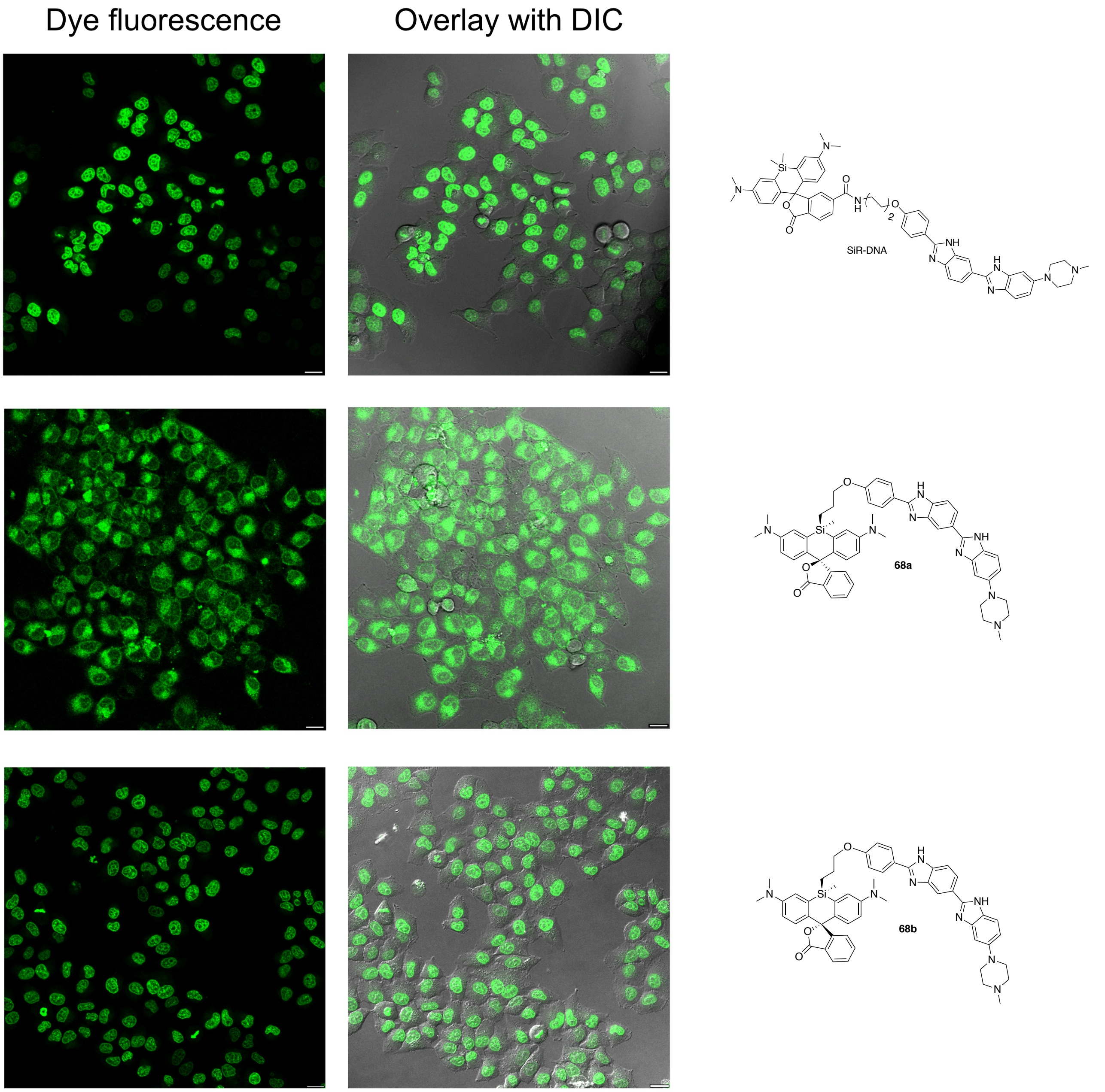

Figure S5. No-wash imaging with SiR-DNA, **68a**, and **68b**. Scale bar is 20 μm, fluorescence is pseudo-colored green.

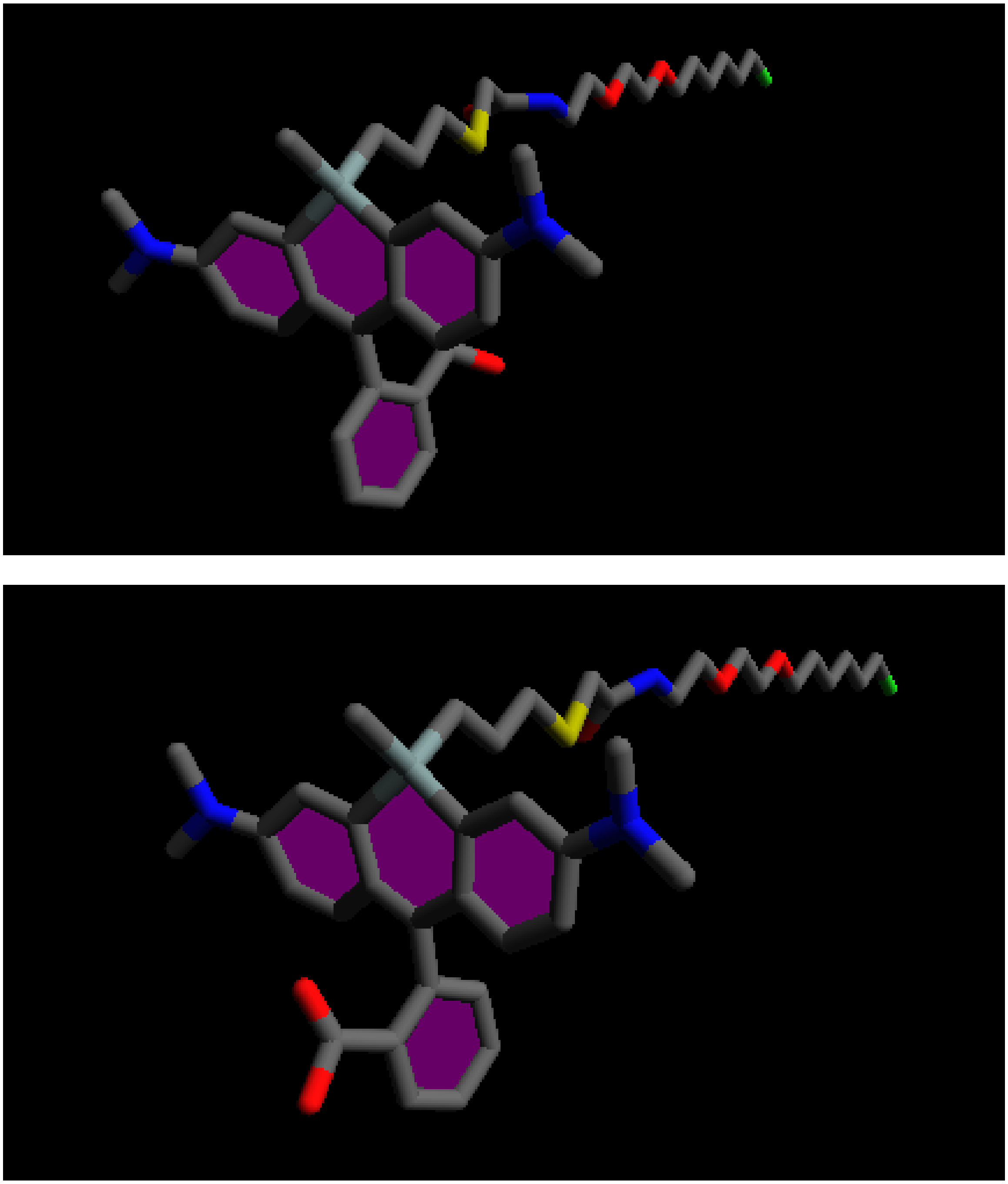

Figure S6. Comparison of **75a** (top) and **75b** (bottom) isomers in their zwitterionic form. We postulate that the preferred orientation for fluorogenic labeling has the carboxylate facing away from the HaloTag ligand and hence the protein surface (**75b**, bottom). Molecules were drawn in Avogadro 1.2.0.[1]

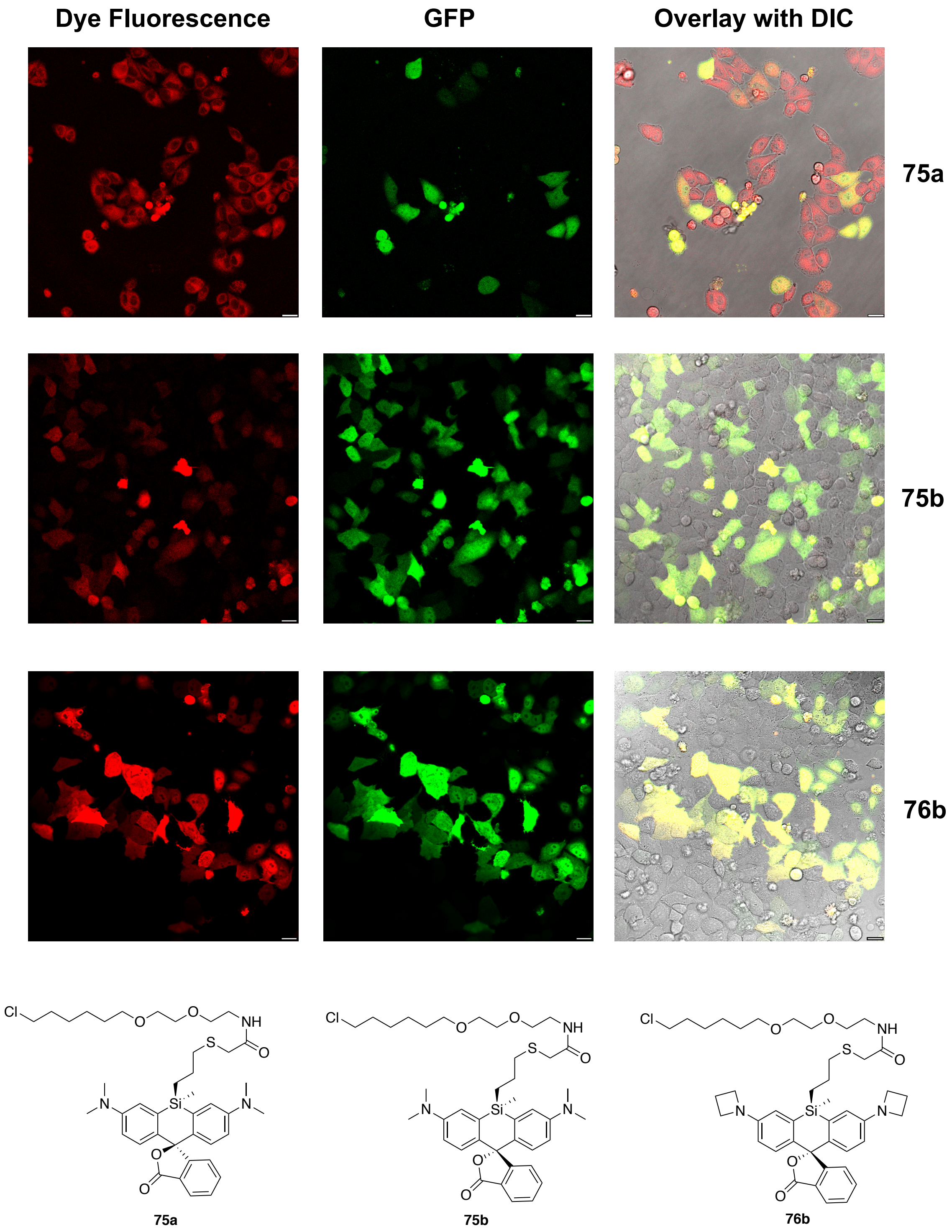

Figure S7. No-wash live cell imaging of HeLa cells transfected with pHaloTag-EGFP with 200 nM dyes **75a**, **75b**, and **76b**.

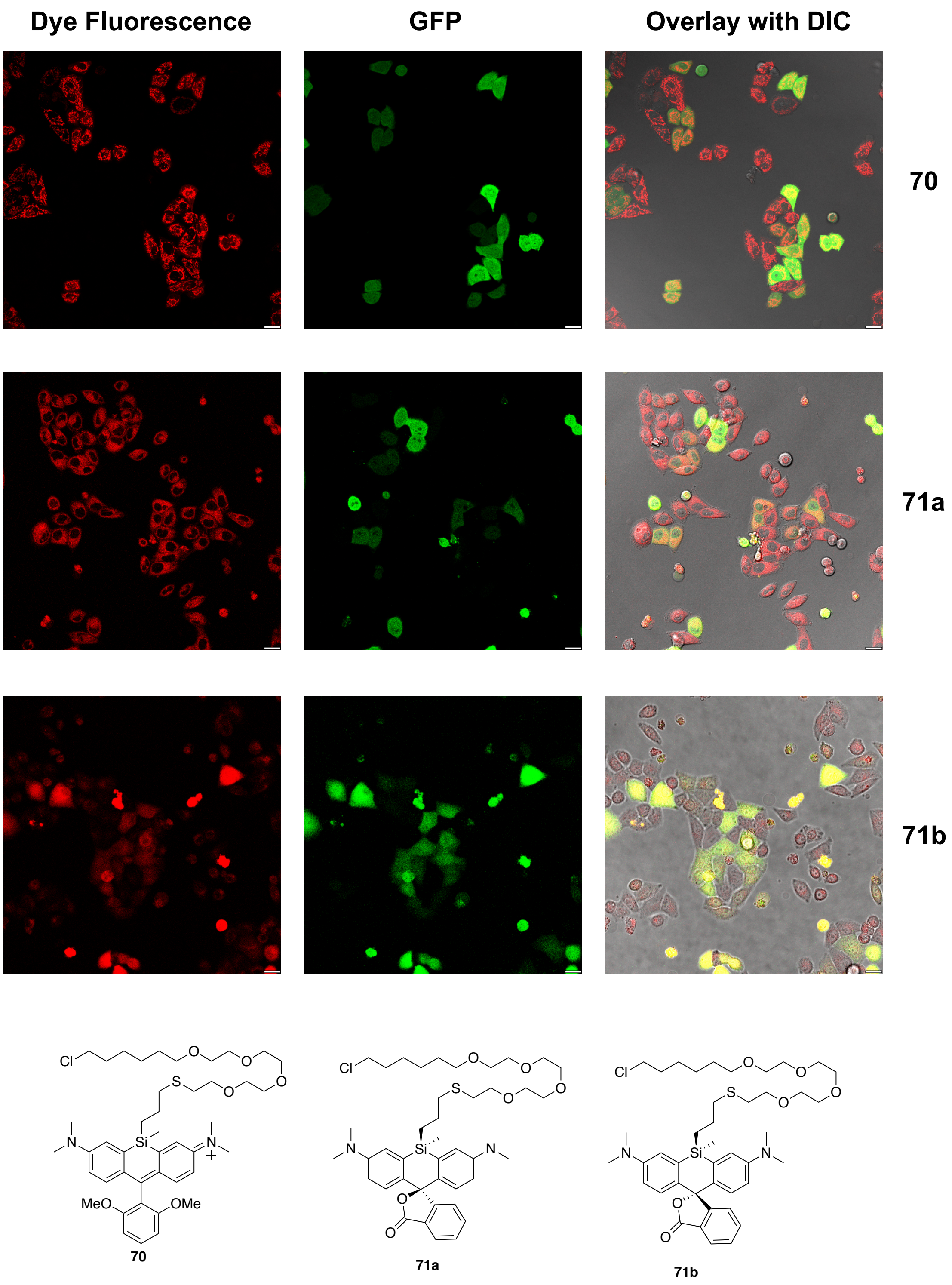

Figure S8. No-wash live cell imaging of transfected with pHaloTag-EGFP with 200 nM dyes **70**, **71a**, and **71b**.

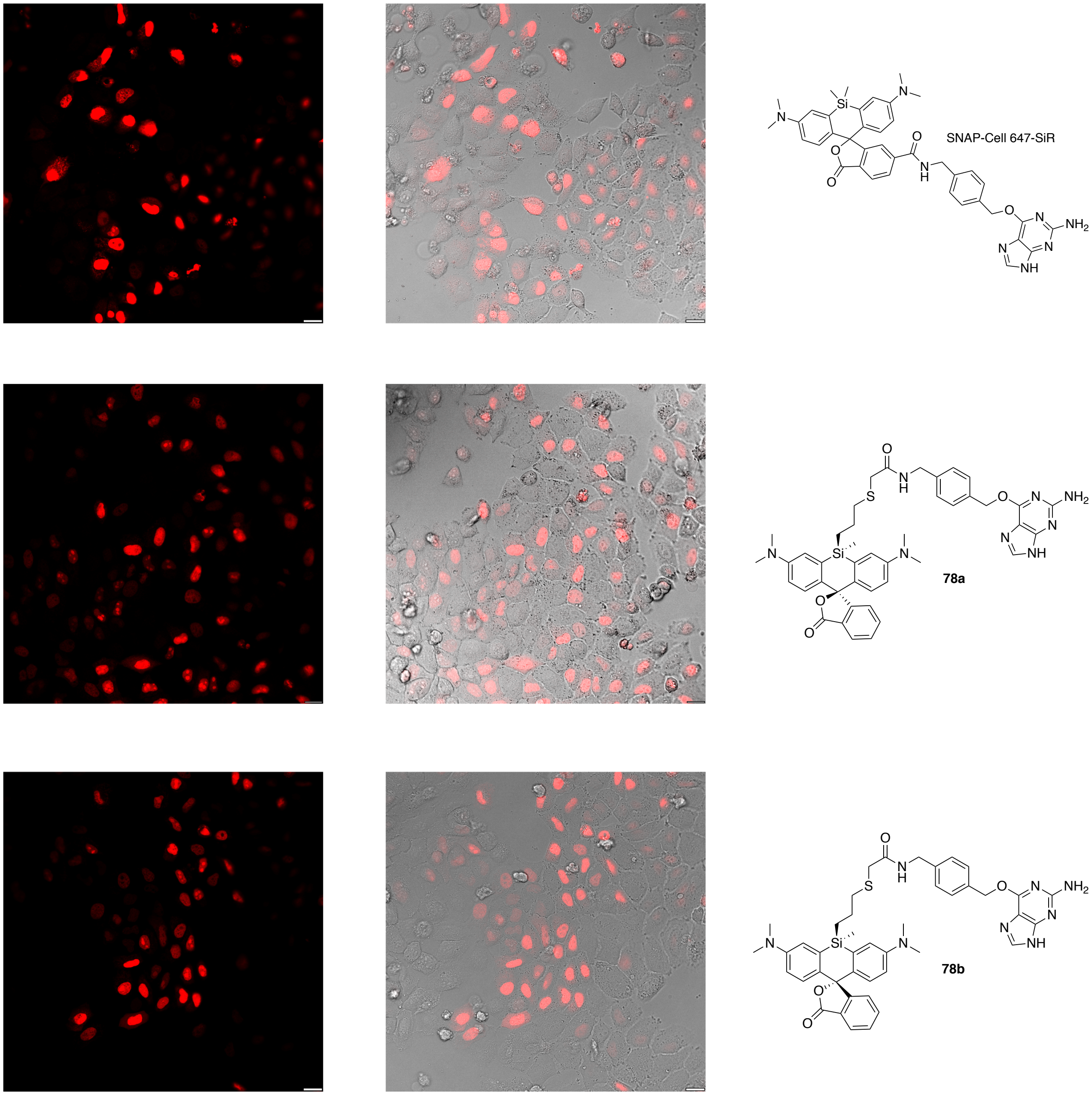

Figure S9. Imaging of HeLa cells transfected with pSNAPf-H2B.

Table S1. Crystal data and structure refinement for **49a**.

Identification code	UMD2365j_a (NM02-54-1)
Empirical formula	C28 H31 Cl N2 O2 Si
Formula weight	491.09
Temperature	297(2) K
Wavelength	0.71073 Å
Crystal system	Triclinic
Space group	P-1
Unit cell dimensions	a = 8.2938(5) Å α= 74.549(2)°.
	b = 10.0055(7) Å β= 88.400(2)°.
	c = 17.6368(11) Å γ = 68.207(2)°.
Volume	1305.73(15) $Å^3$
Z	2
Density (calculated)	1.249 Mg/m^3
Absorption coefficient	0.220 mm^{-1}
F(000)	520
Crystal size	0.300 x 0.240 x 0.120 mm^3
Crystal color / habit	colorless / plate
Theta range for data collection	2.653 to 26.399°.
Index ranges	-10<=h<=10, -12<=k<=12, -22<=l<=22
Reflections collected	49921
Independent reflections	5289 [R(int) = 0.0284]
Completeness to theta = 25.242°	98.6 %
Absorption correction	multi-scan / sadabs
Refinement method	Full-matrix least-squares on F^2
Data / restraints / parameters	5289 / 0 / 312
Goodness-of-fit on F^2	1.063
Final R indices [I>2sigma(I)]	R1 = 0.0404, wR2 = 0.1086
R indices (all data)	R1 = 0.0487, wR2 = 0.1170
Extinction coefficient	n/a
Largest diff. peak and hole	0.262 and -0.292 e.$Å^{-3}$

Table S2. Photophysical properties of all Si-Bridge dyes

Structure	Dye	λ_{ex} (nm)	λ_{em} (nm)	Φ	ε ($M^{-1}cm^{-1}$)	Brightness (Φ x ε) ($M^{-1}cm^{-1}$)
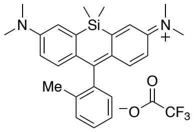	**4 (SiR)**	648	663	0.34	126,400	43,102
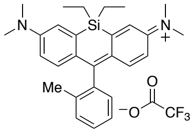	**5**	648	664	0.36	164,900	58,540
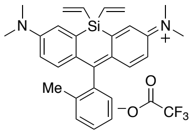	**6**	657	672	0.36	158,300	56,671
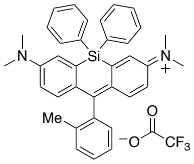	**7**	663	677	0.35	120,900	41,952
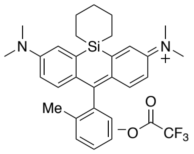	**8**	650	666	0.33	170,000	56,610
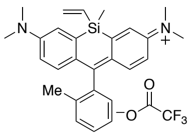	**9**	651	668	0.35	141,400	48,924
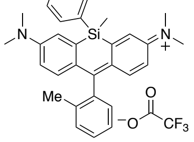	**10**	657	670	0.35	99,140	34,203
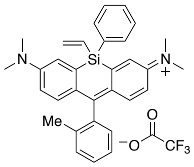	**11**	658	676	0.36	150,700	53,499
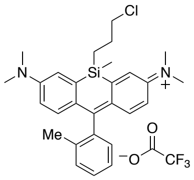	**12**	650	665	0.36	180,600	64,113

All photophysical properties were measured in PBS pH 7.4.

Structure	Dye	λ_{ex} (nm)	λ_{em} (nm)	Φ	ε ($M^{-1}cm^{-1}$)	Brightness ($\Phi \times \varepsilon$) ($M^{-1}cm^{-1}$)
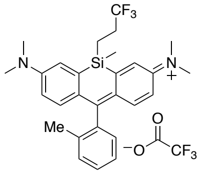	**13**	650	666	0.35	112,900	39,854
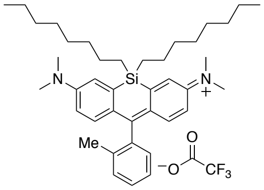	**14**	651	666	0.57[a]	156,300[a]	89,091
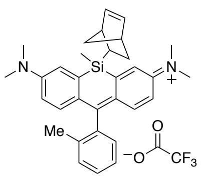	**16**	649	666	0.38	160,000	60,800
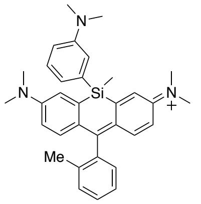	**17**	651	668	0.02<br>0.31[b]	154,200	3700
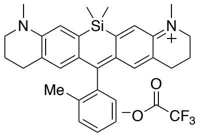	**18 (SiR680)**	674	692	0.31	135,000	41,850
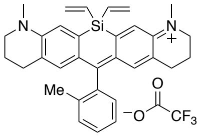	**19**	683	702	0.32	234,700	75,104
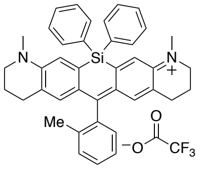	**20**	690	710	0.28	148,300	41,524
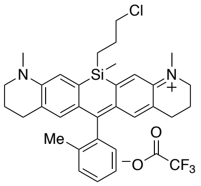	**21**	677	694	0.33	125,000	41,250

Photophysical properties were measured in PBS pH 7.4 except as noted: [a]EtOH; [b]100 mM sodium acetate buffer, pH 3.

Structure	Dye	λ_{ex} (nm)	λ_{em} (nm)	Φ	ε ($M^{-1}cm^{-1}$)	Brightness ($\Phi \times \varepsilon$) ($M^{-1}cm^{-1}$)
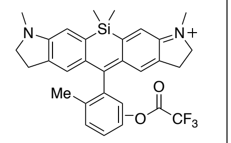	**22 (SiR700)**	690	715	0.17	100,000	17,000
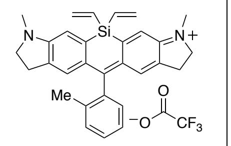	**23**	702	728	0.13	136,100	17,693
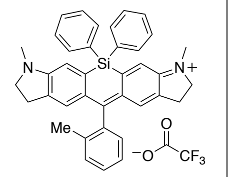	**24**	710	737	0.11	117,000	12,870
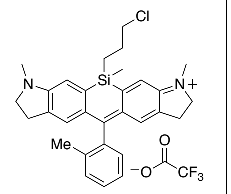	**25**	693	717	0.17	129,100	21,947
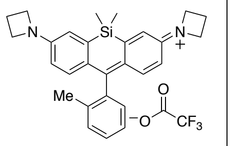	**38**	649	663	0.47	118,000	55,460
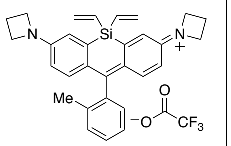	**39**	658	673	0.44	109,700	48,268
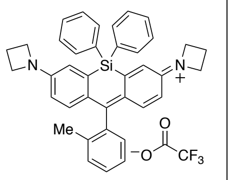	**40**	663	680	0.41	154,200	63,222
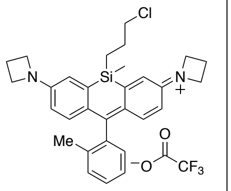	**41**	651	668	0.49	166,700	81,683
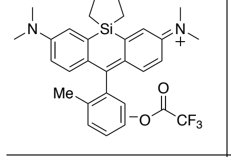	**42**	653	671	0.35	158,000	54,826

All photophysical properties were measured in PBS pH 7.4.

Structure	Dye	λ_{ex} (nm)	λ_{em} (nm)	Φ	ε ($M^{-1}cm^{-1}$)	Brightness ($\Phi \times \varepsilon$) ($M^{-1}cm^{-1}$)
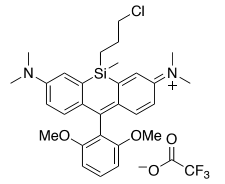	**43**	655	673	0.38	200,000	76,000
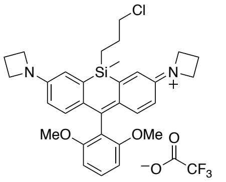	**44**	653	670	0.48	180,000	86,400
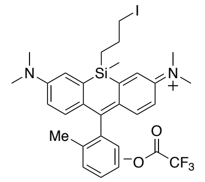	**12-I**	650	666	0.38	140,000	53,200
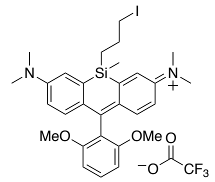	**51**	655	673	0.40	152,100	60,480
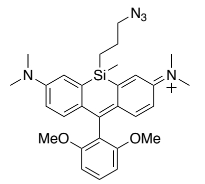	**54**	655	673	0.37	171,000	63,270

All photophysical properties were measured in PBS pH 7.4.

Structure	Dye	λ_{ex} (nm)	λ_{em} (nm)	Φ	ε_{max} ($M^{-1}cm^{-1}$)[a]
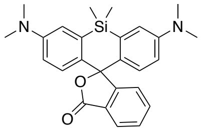	**45 (SiTMR)**	648	667	0.38	130,000
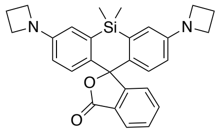	**46 (JF646)**	646	664	0.45	156,000
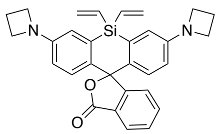	**47**	656[b]	675[b]	ND	80,000
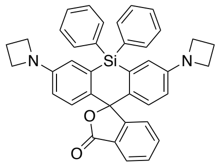	**48**	664[b]	681[b]	ND	80,000
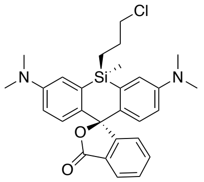	**49a**	645	665	0.40	206,900
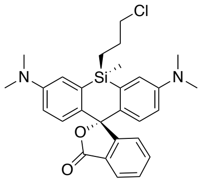	**49b**	645	665	0.40	200,200
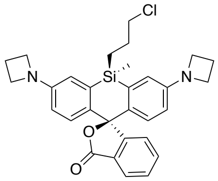	**50a**	648	666	ND	183,000
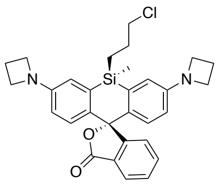	**50b**	648	666	0.53	213,200

Photophysical properties were measured in PBS pH 7.4 except as noted: [a]95%EtOH/ 0.1%TFA; [b]0.1%SDS/PBS.

Structure	Dye	λ_{ex} (nm)	λ_{em} (nm)	Φ	ε_{max} ($M^{-1}cm^{-1}$)[a]
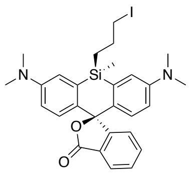	**52a**	645	665	0.43	115,000
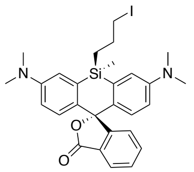	**52b**	645	665	0.45	144,000
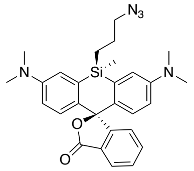	**55a**	645	666	0.43	206,700
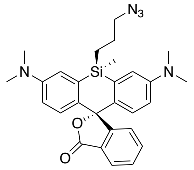	**55b**	645	665	0.44	218,400
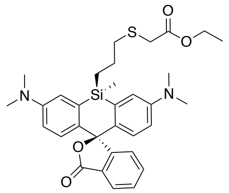	**56a**	645	665	0.45	145,800
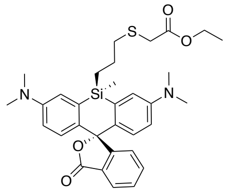	**56b**	646	665	0.45	150,700
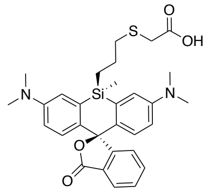	**58a**	645	665	0.45	180,600
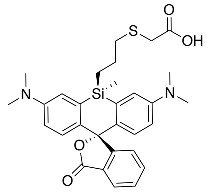	**58b**	645	665	0.45	160,400

Photophysical properties were measured in PBS pH 7.4 except as noted: [a]95%EtOH/ 0.1%TFA.

Table S3. Photophysical properties of 68a and 68b compared to SiR-DNA

Hoechst Probe (PBS)	λ_{ex} (nm)	λ_{em} (nm)	ε ($M^{-1}cm^{-1}$)	ϕ	Brightness (ε x ϕ)
SiR-DNA (ref 2)	667	673	14,500	0.003	43.5
**68a**	660	-	6,500	0.02	130
**68b**	663	-	10,000	0.009	90
+ 30 μM hpDNA					
SiR-DNA (ref 2)	654	677	54,600	0.156	8,518
**68a**	658	672	5,500	0.10	550
**68b**	653	670	26,000	0.40	10,400
0.1% SDS/PBS					
SiR-DNA (ref 2)	652	676	90,000	0.524	47,160
**68a**	650	668	25,500	0.43	10,965
**68b**	649	670	127,000	0.51	64,770

Supplementary Methods

General experimental information

All reactions were performed in oven-dried round bottomed flasks fitted with rubber septa under argon atmosphere, unless otherwise noted. All reagents and solvents, including anhydrous solvents, were purchased from commercial sources and used as received. DBCO and tetrazine reagents were purchased from Click Chemistry Tools. Flash column chromatography was performed on an ISCO CombiFlash Rf+ instrument using RediSep Gold, Silicycle, or Biotage columns. Preparative HPLC was performed on a Varian ProStar equipped with an Agilent 10-Prep C18 21.2 x 250 mm column. Thin-layer chromatography (TLC) was performed using silica gel (60 F-254) coated aluminum plates (EMD Millipore), and spots were visualized by exposure to ultraviolet light (UV), exposure to iodine adsorbed on silica gel, and/or exposure to an acidic solution of phosphomolybdic acid (PMA) followed by brief heating. ^{1}H NMR and ^{13}C NMR spectra were acquired on a Bruker Avance III HD 500 MHz NMR instrument. Chemical shifts are reported in ppm (δ scale) with the residual solvent signal used as reference and coupling constant (J) values

are reported in hertz (Hz). Data are presented as follows: chemical shift, multiplicity (s = singlet, d = doublet, dd = doublet of doublet, td = triplet of doublet, dt = doublet of triplet, t = triplet, q = quartet, p = pentet, m = multiplet, br s = broad singlet), coupling constant in Hz, and integration. High-resolution mass spectra (HRMS) were recorded in the UMass Medical School Mass Spectrometry Facility on a Thermo Scientific Orbitrap Velos Pro mass spectrometer coupled with a Thermo Scientific Accela 1250 UPLC and an autosampler using electrospray ionization (ESI) in the positive mode. Small molecule X-ray crystallography was performed at the South Coast Single Crystal X-Ray Diffraction Facility, UMass Dartmouth. Cell imaging was performed on a Leica SP-8 microscope (SCOPE core facility, UMass Medical School) using a 40X1.30 oil objective. Dyes were excited with the HeNe (633 nm) laser, and EGFP was excited with the Argon (488 nm) laser. Image analysis was performed using Leica LAS X SP8 software.

Dye characterization

Absorption and fluorescence spectra were measured on a Horiba Duetta fluorescence and absorbance spectrometer in quartz cuvettes (Starna Cells, catalog # 3-Q-10). Extinction coefficients were calculated from plots of absorption versus concentration. Quantum yields were measured on a Hamamatsu Quantaurus QY C-11347-11 absolute quantum yield integrating sphere spectrometer at absorption values of <0.1 in side-arm quartz cuvettes (Hamamatsu cat # A10095-02). All measurements were performed in PBS (9.0 g/l NaCl, 0.795 g/l Na_2HPO_4, 0.144 g/l KH_2PO_4, pH 7.4, Corning cat #21-040-CV), ethanol, or 0.1%TFA/ethanol and prepared from stock solutions of dyes in DMSO, with final DMSO <1%. The photophysical properties of **68a** and **68b** were also measured after a 2h incubation in 0.1% SDS/PBS and after 2h treatment with 30 μM hairpin DNA (hpDNA).[2] Selected $K_{L\text{-}Z}$ values were determined in 1:1 v/v dioxane:water as previously described.[3]

Small molecule x-ray crystallography

A colorless crystal of **49a**, recrystallized from 1:1 DCM/EtOAc, was mounted on a Cryoloop with oil. Data were collected at 24°C on a Bruker D8 Venture X-ray single crystal instrument using Mo K alpha radiation and data were corrected for absorption with SADAS. The structure was solved by direct methods (intrinsic phasing), and all non-hydrogen atoms were refined by full matrix least squares on F^2. All hydrogen atoms were placed in calculate positions with appropriate riding parameters.

Highest peak 0.26 at 0.8598 0.3853 0.3207 [0.83 A from C15]
Deepest hole -0.29 at 0.7797 0.8952 0.4871 [0.87 A from CL1]

Plasmids

pSNAPf-H2B Control Plasmid was a gift from New England Biolabs & Ana Egana (Addgene plasmid # 101124 ; http://n2t.net/addgene:101124 ; RRID:Addgene_101124)

pHaloTag-EGFP was a gift from Thomas Leonard & Ivan Yudushkin (Addgene plasmid # 86629 ; http://n2t.net/addgene:86629 ; RRID:Addgene_86629)

No-wash imaging of the nucleus with Si-rhodamine Hoechst dyes

HeLa cells were cultured in Dulbecco's modified Eagle medium (DMEM, from GIBCO, catalog no. 11995065) supplemented with 10% fetal bovine serum (FBS) (GIBCO, catalog no. 10437028) and 1% penicillin-streptomycin (Sigma) at 37°C in a 5% CO_2 incubator. For imaging, cells were seeded in 35 mm glass bottom dishes (Cellvis, catalog no. D35-28-0-N). Labeling of the nucleus in live cells using SiR-DNA (Spirochrome, Cytoskeleton cat no. CY-SC007) and compounds **68a** and **68b** was performed following the manufacturer's instructions for SiR-DNA.

No-wash imaging of HaloTag-expressing cells

HeLa cells were seeded in 35 mm glass bottom dishes (Cellvis, catalog no. D35-28-0-N) and transfected with pHaloTag®-EGFP (Addgene #86629). Transient transfections were performed using Lipofectamine 2000 (Invitrogen, catalog no.1168019) following the manufacturer's instructions. HeLa cell labeling and confocal imaging were performed 24 hr after transfection. Labeling with dyes containing HaloTag® ligands

(Promega) was performed following the manufacturer's instructions. Briefly, cells were incubated with 200 nM dye in DMEM for 15 min at 37 °C and then imaged.

Imaging of SNAP-tag-expressing cells

HeLa cells were seeded in 35 mm glass bottom dishes (Cellvis, catalog no. D35-28-0-N) and transfected with pSNAPf-H2B control plasmid (Addgene #101124). Transient transfections were performed using Lipofectamine 2000 (Invitrogen, catalog no.1168019) following the manufacturer's instructions. HeLa cell labeling and confocal imaging were performed 24 hr after transfection. Labeling with SNAP-cell® 647 SiR (New England Biolabs, catalog no. S9102S) and dyes **78a** and **78b** containing O^6-benzylguanine SNAP-tag ligands was performed following the manufacturer's instructions. Briefly, cells were incubated with 3 μM dye in cell culture medium for 30 min at 37 °C. The cells were then washed three times with tissue culture medium and incubated in fresh medium for 30 minutes. The medium was replaced one more time to remove unreacted SNAP-tag before imaging.

Chemical Synthesis

4,4'-(*o*-Tolylmethylene)bis(3-bromo-*N*,*N*-dimethylaniline) (1)[4].

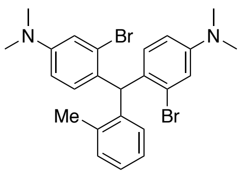

A solution of 3-bromo-*N*,*N*-dimethylaniline (8.0 g, 40.0 mmol) in anhydrous toluene (20.0 mL) was treated with 2-methylbenzaldehyde (1.16 mL, 10.0 mmol) and *p*-toluenesulfonic acid monohydrate (1.90 g, 10.0 mmol) at room temperature and then the mixture solution was refluxed in a Dean-Stark apparatus. After 3 h, another 20 mL of anhydrous toluene was added into the reaction and the reaction mixture was refluxed overnight, then cooled to room temperature. The solvent was evaporated under reduced pressure, and the residue was dried under high vacuum for an hour. The resulting oil was then dissolved in dichloromethane (200 mL), washed with saturated aqueous $NaHCO_3$ (100 mL), dried (Na_2SO_4), filtered, and evaporated under reduced pressure. The residue was purified by flash column chromatography (RediSep Gold column, 80 g, 0–60% DCM/hexanes, linear gradient for 20 min) to provide **1** (2.60 g, 52%) as a white foamy solid. ^{1}H NMR (500 MHz, $CDCl_3$) δ 7.17–7.11 (m, 2 H), 7.07 (td, J = 7.5, 2.5 Hz, 1 H), 6.94 (d, J = 2.5 Hz, 2 H), 6.72 (d, J = 7.5 Hz, 1 H), 6.62 (d, J = 9.0 Hz, 2 H), 6.53 (dd, J = 8.5, 2.5 Hz, 2 H), 5.96 (s, 1 H), 2.91 (s, 12 H), 2.20 (s, 3 H) ppm; ^{13}C NMR (125 MHz, $CDCl_3$) δ 150.04, 141.75, 137.12, 131.09, 130.37, 129.63, 128.85, 126.65, 126.35, 125.72, 116.60, 111.34, 51.52, 40.53, 19.67 ppm; HRMS (ESI) *m/z*: $[M + H]^+$ calcd for $C_{24}H_{27}Br_2N_2$, 503.0515; found 503.0509.

7-Bromo-1-methyl-1,2,3,4-tetrahydroquinoline[5].

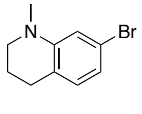

A solution of 7-bromoquinoline (5.0 g, 24.0 mmol) in acetic acid (80.0 mL) was treated with paraformaldehyde (7.21 g, 240 mmol) under an argon atmosphere was cooled to 0 °C in an ice-water bath. After 10 min, $NaBH_3CN$ (3.77 g, 60.0 mmol) was added in small portions. The resulting reaction mixture was warmed to room temperature and stirred for

4 h. The reaction mixture was cooled to 0 °C in an ice-water bath and neutralized with 2M NaOH solution (100 mL). After extraction with DCM (2 x 150 mL), the combined extracts were washed with saturated NaCl solution (150 mL), dried (Na_2SO_4), filtered, and evaporated under reduced pressure. The resulting residue was purified by flash column chromatography (Silicycle column, 50 g, 0–10% EtOAc/Hexanes, linear gradient for 20 min) to provide 7-bromo-1-methyl-1,2,3,4-tetrahydroquinoline (2.90 g, 53%) as a colorless liquid. ^{1}H NMR (500 MHz, $CDCl_3$) δ 6.78 (d, J = 8.0 Hz, 1 H), 6.69 (dd, J = 8.0, 2.0 Hz, 1 H), 6.66 (d, J = 1.5 Hz, 1 H), 3.22 (t, J = 6.0 Hz, 2 H), 2.87 (s, 3 H), 2.69 (t, J = 6.5 Hz, 2 H), 1.95 (p, J = 6.5 Hz, 2 H) ppm; ^{13}C NMR (125 MHz, $CDCl_3$) δ 147.84, 129.95, 121.62, 120.74, 118.60, 113.37, 51.00, 39.04, 27.50, 22.21 ppm; HRMS (ESI) *m/z*: $[M + H]^+$ calcd for $C_{10}H_{13}BrN$, 226.0226; found 226.0221.

6-Bromo-1-methylindoline.

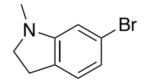

The same procedure was used as described above for compound 7-bromo-1-methyl-1,2,3,4-tetrahydroquinoline. A mixture of 6-bromoindole (6.0 g, 30.6 mmol) and paraformaldehyde (9.19 g, 306 mmol) in AcOH (80.0 mL) was treated with $NaBH_3CN$ (4.80 g, 76.5 mmol) to provide 6-bromo-1-methylindoline (3.80 g, 58%) as a colorless liquid. ^{1}H NMR (500 MHz, $CDCl_3$) δ 6.89 (d, J = 8.0 Hz, 1 H), 6.75 (dd, J = 7.5, 1.5 Hz, 1 H), 6.55 (d, J = 2.0 Hz, 1 H), 3.33 (t, J = 8.0 Hz, 2 H), 2.89 (t, J = 8.0 Hz, 2 H), 2.74 (s, 3 H) ppm; ^{13}C NMR (125 MHz, $CDCl_3$) δ 154.92, 129.39, 125.37, 121.17, 120.17, 110.13, 56.25, 35.79, 28.36 ppm; HRMS (ESI) *m/z*: $[M + H]^+$ calcd for $C_9H_{11}BrN$, 212.0069; found 212.0067.

7-Bromo-6-((5-bromo-1-methyl-1,2,3,4-tetrahydroquinolin-6-yl)(*o*-tolyl)methyl)-1-methyl-1,2,3,4-tetrahydroquinoline (2)[5].

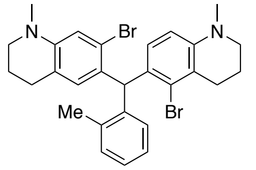

A solution of 7-bromo-1-methyl-1,2,3,4-tetrahydroquinoline (1.38 g, 6.10 mmol) in 2N HCl (50.0 mL) was treated with 2-methylbenzaldehyde (0.36 mL, 3.05 mmol) under argon atmosphere was refluxed for overnight. After cooling to room temperature, the reaction mixture was cooled to 0 °C in an ice-water bath and neutralized with saturated $NaHCO_3$ solution (100 mL) and extraction with DCM (2 x 100 mL), the combined organic extracts were washed with saturated NaCl solution (100 mL), dried (Na_2SO_4), filtered, and evaporated under reduced pressure. The resulting residue was purified by flash column chromatography (Silicycle column, 50 g, 0–60% DCM/Hexanes, linear gradient for 20 min) to provide **2** (1.30 g, 77%) as a white foamy solid. ^{1}H NMR (500 MHz, $CDCl_3$) δ 7.16–7.09 (m, 2 H), 7.08–7.04 (m, 1 H), 6.78 (s, 2 H), 6.73 (d, J = 7.5 Hz, 1 H), 6.34 (s, 2 H), 5.88 (s, 1 H), 3.20 (t, J = 6.0 Hz, 4 H), 2.86 (s, 6 H), 2.62–2.51 (m, 4 H), 2.20 (s, 3 H), 1.91 (p, J = 6.0 Hz, 4 H) ppm; ^{13}C NMR (125 MHz, $CDCl_3$) δ 141.93, 137.15, 130.73, 130.26, 128.91, 126.19, 125.64, 123.92, 122.02, 114.97, 51.54, 51.06, 39.29, 27.57, 22.15, 19.75 ppm; HRMS (ESI) *m/z*: $[M + H]^+$ calcd for $C_{28}H_{31}Br_2N_2$, 553.0849; found 553.0839.

6-Bromo-5-((4-bromo-1-methylindolin-5-yl)(*o*-tolyl)methyl)-1-methylindoline (3).

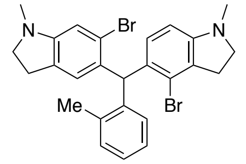

The same procedure was used as described above for compound **2**. A solution of 6-bromo-1-methylindoline (1.35 g, 6.36 mmol) in 2N HCl (50.0 mL) was treated with 2-methylbenzaldehyde (0.37 mL, 3.19 mmol) to provide **3** (1.0 g, 60%) as a white foaming solid. ^{1}H NMR (500 MHz, $CDCl_3$) δ 7.18–7.11 (m, 2 H), 7.10–7.05 (m, 1 H), 6.73 (d, J = 7.5 Hz, 1 H), 6.68 (s, 2 H), 6.49 (s, 2 H), 5.96 (s, 1 H), 3.32 (t, J = 6.5 Hz, 4 H), 2.81 (t, J

= 6.5 Hz, 4 H), 2.74 (s, 6 H), 2.19 (s, 3 H) ppm; ^{13}C NMR (125 MHz, $CDCl_3$) δ 141.84, 137.23, 130.38, 129.92, 128.90, 126.42, 126.37, 125.73, 124.44, 113.96, 56.40, 52.33, 36.42, 28.57, 19.71 ppm; HRMS (ESI) *m/z*: $[M + H]^+$ calcd for $C_{26}H_{27}Br_2N_2$, 525.0536; found 525.0528.

Synthesis of Si-Bridge rhodamines

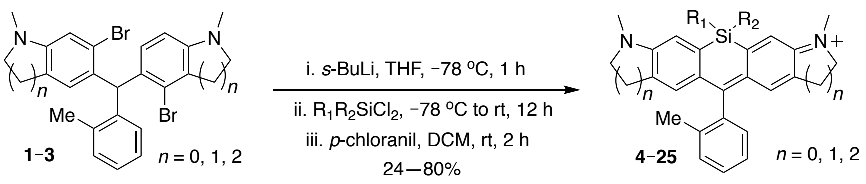

***N*-(7-(Dimethylamino)-5,5-dimethyl-10-(*o*-tolyl)dibenzo[*b*,*e*]silin-3(5*H*)-ylidene)-*N*-methylmethanaminium (4).**

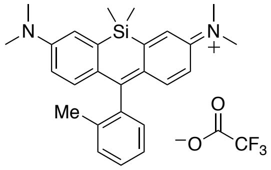

A degassed solution of 4,4'-(*o*-tolylmethylene)bis(3-bromo-*N*,*N*-dimethylaniline) **1** (0.10 g, 0.20 mmol) in anhydrous THF (5.0 mL) under argon atmosphere was cooled to −78 °C in an acetone/dry ice bath. After 15 min, *s*-BuLi (1.4M in cyclohexane) (0.32 mL, 0.44 mmol) was added dropwise over 10 min. The resulting reaction mixture was stirred at −78 °C for additional one hour. At the same temperature, dichlorodimethylsilane (40.0 µL, 0.30 mmol) dissolved in anhydrous THF (5.0 mL) was added dropwise over 10 min. The reaction mixture was then slowly warmed to room temperature and stirred overnight. The reaction mixture was then cooled to ~ 5 °C and quenched by addition of 2N HCl (1.0 mL) and stirred at room temperature for 10 min. $NaHCO_3$ (10.0 mL) was added and then extracted with dichloromethane (25.0 mL), which was dried (Na_2SO_4), filtered, and evaporated under reduced pressure. The residue was re-dissolved in anhydrous DCM (10.0 mL) and treated with *p*-chloranil (0.10 g, 0.40 mmol) at room temperature, and then the

mixture solution was stirred for 2 h. The solvent was then evaporated under reduced pressure, and the residue was purified by flash column chromatography (Silicycle column, 12 g, 0–15% MeOH in 1% v/v TFA/DCM, linear gradient for 20 min) to yield (60.0 mg, 68%) of the trifluoroacetate salt of **4** as a dark blue color solid. An analytically pure sample was obtained through further purification by reverse-phase HPLC (30–95% MeCN/H_2O, linear gradient, with constant 0.1% v/v TFA additive). ^1H NMR (500 MHz, $CDCl_3$) δ 7.45–7.41 (m, 1 H), 7.36–7.32 (m, 2 H), 7.18 (d, J = 2.5 Hz, 2 H), 7.10–7.07 (m, 3 H), 6.59 (dd, J = 10.0, 3.0 Hz, 2 H), 3.43 (s, 12 H), 2.03 (s, 3 H), 0.60 (s, 3 H), 0.58 (s, 3 H) ppm; ^{13}C NMR (125 MHz, $CDCl_3$) δ 170.44, 154.29, 148.83, 141.77, 138.54, 135.83, 130.41, 129.08, 129.02, 127.79, 125.78, 120.88, 114.03, 40.97, 19.50, −0.84, −1.17 ppm; ^{19}F NMR (470 MHz, $CDCl_3$) δ −75.65 ppm; HRMS (ESI) *m/z*: $[M]^+$ calcd for $C_{26}H_{31}N_2Si$, 399.2251; found 399.2243.

***N*-(7-(Dimethylamino)-5,5-diethyl-10-(*o*-tolyl)dibenzo[*b*,*e*]silin-3(5*H*)-ylidene)-*N*-methylmethanaminium (5).**

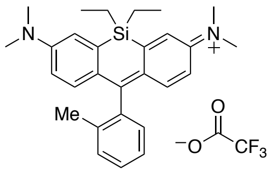

The same procedure was used as described above for compound **4**. 4,4'-(*o*-tolylmethylene)bis(3-bromo-*N*,*N*-dimethylaniline) **1** (0.15 g, 0.30 mmol) in anhydrous THF (8.0 mL) was treated with *s*-BuLi (1.4M in cyclohexane) (0.47 mL, 0.66 mmol) and dichlorodiethylsilane (70.0 µL, 0.45 mmol). The resulting residue was re-dissolved in DCM (10.0 mL), followed by treatment with *p*-chloranil (0.15 g, 0.60 mmol). The residue was purified by flash column chromatography (Silicycle column, 12 g, 0–5% MeOH in 1% v/v TFA/DCM for 10 min, hold at 5% MeOH isocratic for 5 min, and then increase to 15% gradient over 5 min) to provide **5** (90.0 mg, 71%) as a dark blue color solid. ^1H NMR (500 MHz, $CDCl_3$) δ 7.46–7.40 (m, 1 H), 7.38–7.30 (m, 2 H), 7.13 (d, J = 2.0 Hz, 2 H), 7.12–7.06 (m, 3 H), 6.62 (dd, J = 9.5, 2.5 Hz, 2 H), 3.34 (s, 12 H), 2.01 (s, 3 H), 1.14–1.03 (m, 4 H), 1.00 (t, J = 8.0 Hz, 3 H), 0.93 (t, J = 7.5 Hz, 3 H) ppm; ^{13}C NMR (125 MHz, $CDCl_3$)

δ 170.99, 154.12, 146.90, 141.90, 138.53, 135.73, 130.43, 129.11, 129.01, 128.58, 125.78, 120.61, 114.13, 40.93, 19.43, 7.31, 7.21, 6.32, 5.78 ppm; ^{19}F NMR (470 MHz, $CDCl_3$) δ −75.72 ppm; HRMS (ESI) *m/z*: $[M]^+$ calcd for $C_{28}H_{35}N_2Si$, 427.2564; found 427.2557.

***N*-(7-(Dimethylamino)-10-(*o*-tolyl)-5,5-divinyldibenzo[*b*,*e*]silin-3(5*H*)-ylidene)-*N*-methylmethanaminium (6).**

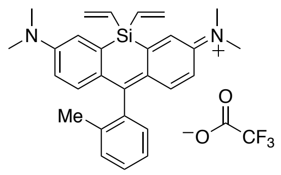

The same procedure was used as described above for compound **4**. 4,4'-(*o*-tolylmethylene)bis(3-bromo-*N*,*N*-dimethylaniline) **1** (0.15 g, 0.30 mmol) in anhydrous THF (8.0 mL) was treated with *s*-BuLi (1.4M in cyclohexane) (0.47 mL, 0.66 mmol) and dichlorodivinylsilane (70.0 μL, 0.45 mmol). The resulting residue was re-dissolved in DCM (10.0 mL), followed by treatment with *p*-chloranil (0.15 g, 0.60 mmol). The residue was purified by flash column chromatography (Silicycle column, 12 g, 0–15% MeOH in 1% v/v TFA/DCM, linear gradient for 20 min) to yield (70.0 mg, 52%) of the trifluoroacetate salt of **6** as a dark blue color solid. An analytically pure sample was obtained through further purification by reverse-phase HPLC (30–95% MeCN/H_2O, linear gradient, with constant 0.1% v/v TFA additive). ^{1}H NMR (500 MHz, $CDCl_3$) δ 7.46–7.41 (m, 1 H), 7.36–7.32 (m, 2 H), 7.12–7.07 (m, 5 H), 6.62 (dd, J = 9.5, 3.0 Hz, 2 H), 6.44–6.28 (m, 4 H), 6.02 (dd, J = 19.5, 3.5 Hz, 1 H), 5.94 (dd, J = 18.0, 5.0 Hz, 1 H), 3.33 (s, 12 H), 2.07 (s, 3 H) ppm; ^{13}C NMR (125 MHz, $CDCl_3$) δ 170.39, 154.18, 144.32, 142.00, 139.66, 139.25, 138.22, 135.83, 131.05, 130.68, 130.47, 129.19, 129.04, 128.13, 125.80, 122.23, 114.32, 40.98, 19.48 ppm; ^{19}F NMR (470 MHz, $CDCl_3$) δ −75.64 ppm; HRMS (ESI) *m/z*: $[M]^+$ calcd for $C_{28}H_{31}N_2Si$, 423.2251; found 423.2244.

N-(7-(Dimethylamino)-5,5-diphenyl-10-(o-tolyl)dibenzo[b,e]silin-3(5H)-ylidene)-N-methylmethanaminium (7).

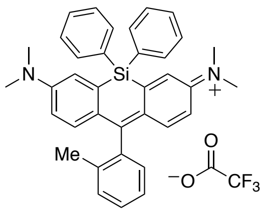

The same procedure was used as described above for compound **4**. 4,4'-(o-tolylmethylene)bis(3-bromo-N,N-dimethylaniline) **1** (0.15 g, 0.30 mmol) in anhydrous THF (8.0 mL) was treated with s-BuLi (1.4M in cyclohexane) (0.47 mL, 0.66 mmol) and dichlorodiphenylsilane (94.0 μL, 0.45 mmol). The resulting residue was re-dissolved in DCM (10.0 mL), followed by treatment with p-chloranil (0.15 g, 0.60 mmol). The residue was purified by flash column chromatography (Silicycle column, 12 g, 0–15% MeOH in 1% v/v TFA/DCM, linear gradient for 20 min) to yield (80.0 mg, 51%) of the trifluoroacetate salt of **7** as a dark blue color solid. An analytically pure sample was obtained through further purification by reverse-phase HPLC (30–95% MeCN/H_2O, linear gradient, with constant 0.1% v/v TFA additive). ^{1}H NMR (500 MHz, $CDCl_3$) δ 7.64 (dd, J = 8.0, 1.0 Hz, 2 H), 7.58 (dd, J = 8.0, 1.0 Hz, 2 H), 7.56–7.51 (m, 2 H), 7.50–7.43 (m, 5 H), 7.37–7.33 (m, 2 H), 7.15 (d, J = 9.5 Hz, 2 H), 7.13–7.10 (m, 3 H), 6.65 (dd, J = 10.0, 3.0 Hz, 2 H), 3.25 (s, 12 H), 2.09 (s, 3 H) ppm; ^{13}C NMR (125 MHz, $CDCl_3$) δ 170.46, 154.15, 145.16, 142.09, 138.14, 135.98, 135.82, 131.44, 131.39, 131.32, 130.89, 130.54, 129.28, 129.06, 129.03, 129.01, 128.43, 125.85, 122.74, 114.49, 40.96, 19.48 ppm; ^{19}F NMR (470 MHz, $CDCl_3$) δ −75.66 ppm; HRMS (ESI) m/z: $[M]^+$ calcd for $C_{36}H_{35}N_2Si$, 523.2564; found 523.2552.

***N*-(7-(Dimethylamino)-10-(*o*-tolyl)-3*H*-spiro[dibenzo[*b*,*e*]siline-5,1'-silinan]-3-ylidene)-*N*-methylmethanaminium (8).**

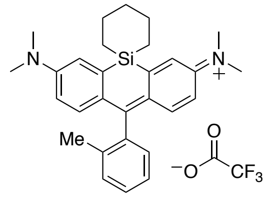

The same procedure was used as described above for compound **4**. 4,4'-(*o*-tolylmethylene)bis(3-bromo-*N*,*N*-dimethylaniline) **1** (0.15 g, 0.30 mmol) in anhydrous THF (8.0 mL) was treated with *s*-BuLi (1.4M in cyclohexane) (0.47 mL, 0.66 mmol) and dichlorocyclohexylsilane (80.0 μL, 0.45 mmol). The resulting residue was re-dissolved in DCM (10.0 mL), followed by treatment with *p*-chloranil (0.15 g, 0.60 mmol). The residue was purified by flash column chromatography (Silicycle column, 12 g, 0–5% MeOH in 1% v/v TFA/DCM for 10 min, hold at 5% MeOH isocratic for 5 min, and then increase to 15% gradient over 5 min) to provide **8** (50.0 mg, 39%) as a dark blue color solid. ^{1}H NMR (500 MHz, $CDCl_3$) δ 7.45–7.40 (m, 1 H), 7.36–7.30 (m, 4 H), 7.11–7.04 (m, 3 H), 6.67 (dd, J = 9.5, 3.0 Hz, 2 H), 3.42 (s, 12 H), 2.08–2.02 (m, 4 H), 2.01 (s, 3 H), 1.83–1.76 (m, 2 H), 1.17–1.09 (m, 4 H) ppm; ^{13}C NMR (125 MHz, $CDCl_3$) δ 170.04, 153.97, 148.13, 141.90, 138.56, 135.78, 130.43, 129.08, 129.02, 128.02, 125.81, 121.23, 114.29, 41.28, 29.12, 24.38, 24.30, 19.59, 13.07, 12.87 ppm; HRMS (ESI) *m/z*: $[M]^+$ calcd for $C_{29}H_{35}N_2Si$, 439.2564; found 439.2556.

***N*-(7-(Dimethylamino)-5-methyl-10-(*o*-tolyl)-5-vinyldibenzo[*b*,*e*]silin-3(5*H*)-ylidene)-*N*-methylmethanaminium (9).**

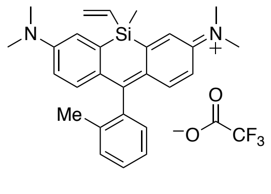

The same procedure was used as described above for compound **4**. 4,4'-(*o*-tolylmethylene)bis(3-bromo-*N*,*N*-dimethylaniline) **1** (0.15 g, 0.30 mmol) in anhydrous THF (8.0 mL) was treated with *s*-BuLi (1.4M in cyclohexane) (0.47 mL, 0.66 mmol) and

dichloro(methyl)(vinyl)silane (63.0 μL, 0.45 mmol). The resulting residue was re-dissolved in DCM (10.0 mL), followed by treatment with *p*-chloranil (0.15 g, 0.60 mmol). The residue was purified by flash column chromatography (Silicycle column, 12 g, 0–5% MeOH in 1% v/v TFA/DCM for 10 min, hold at 5% MeOH isocratic for 5 min, and then increase to 15% gradient over 5 min) to provide **9** as an inseparable mixture of isomers (72.0 mg, 58%) in dark blue color solid. ^{1}H NMR (500 MHz, $CDCl_3$) δ 7.46–7.41 (m, 1 H), 7.37–7.31 (m, 2 H), 7.13–7.06 (m, 5 H), 6.61 (dd, J = 9.5, 3.0 Hz, 2 H), 6.37–6.21 (m, 2 H), 6.00 (dd, J = 19.5, 3.5 Hz, 1 H), 3.33 (s, 12 H), 2.04 and 2.01 (2 x s, 3 H), 0.68 and 0.65 (2 x s, 3 H) ppm; ^{13}C NMR (125 MHz, $CDCl_3$) δ 170.55, 154.25, 146.46, 141.89, 138.35, 138.01, 137.69, 135.81 (2 signals), 133.06, 132.72, 130.45 (2 signals), 129.15, 129.05, 128.97, 127.95, 125.80 (2 signals), 121.53 (2 signals), 114.18, 40.94, 19.49 (2 signals), –3.26 (2 signals) ppm; ^{19}F NMR (470 MHz, $CDCl_3$) δ −75.63 ppm; HRMS (ESI) *m/z*: $[M]^+$ calcd for $C_{27}H_{31}N_2Si$, 411.2251; found 411.2244.

N-(7-(Dimethylamino)-5-methyl-5-phenyl-10-(*o*-tolyl)dibenzo[*b*,*e*]silin-3(5*H*)-ylidene)-*N*-methylmethanaminium (10).

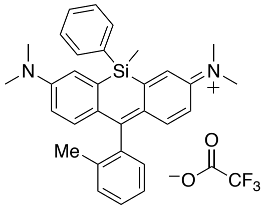

The same procedure was used as described above for compound **4**. 4,4'-(*o*-tolylmethylene)bis(3-bromo-*N*,*N*-dimethylaniline) **1** (0.15 g, 0.30 mmol) in anhydrous THF (8.0 mL) was treated with *s*-BuLi (1.4M in cyclohexane) (0.47 mL, 0.66 mmol) and dichloro(methyl)(phenyl)silane (85.0 μL, 0.45 mmol). The resulting residue was re-dissolved in DCM (10.0 mL), followed by treatment with *p*-chloranil (0.15 g, 0.60 mmol). The residue was purified by flash column chromatography (Silicycle column, 12 g, 0–5% MeOH in 1% v/v TFA/DCM for 10 min, hold at 5% MeOH isocratic for 5 min, and then increase to 15% gradient over 5 min) to provide **10** as an inseparable mixture of isomers (0.11 g, 76%) in dark blue color solid. ^{1}H NMR (500 MHz, $CDCl_3$) δ 7.59–7.54 (m, 2 H), 7.51–7.40 (m, 4 H), 7.39–7.32 (m, 2 H), 7.15–7.09 (m, 3 H), 7.08–7.05 (m, 2 H), 6.61 (dd,

J = 9.5, 2.5 Hz, 2 H), 3.26 (s, 12 H), 2.07 and 2.05 (2 x s, 3 H), 0.93 and 0.90 (2 x s, 3 H) ppm; ^{13}C NMR (125 MHz, $CDCl_3$) δ 170.73, 154.29 (2 signals), 146.83, 141.90 (2 signals), 138.34, 135.91, 134.66, 134.53, 133.34, 131.00, 130.50, 129.22, 128.94, 128.89, 128.10 (2 signals), 125.84 (2 signals), 121.71, 114.25, 40.89, 19.50 (2 signals), –3.25 (2 signals) ppm; ^{19}F NMR (470 MHz, $CDCl_3$) δ −75.85 ppm; HRMS (ESI) *m/z*: $[M]^+$ calcd for $C_{31}H_{33}N_2Si$, 461.2408; found 461.2401.

_N_-(7-(Dimethylamino)-5-phenyl-10-(_o_-tolyl)-5-vinyldibenzo[_b_,_e_]silin-3(5_H_)-ylidene)-_N_-methylmethanaminium (11).

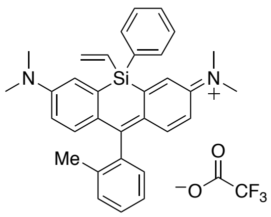

The same procedure was used as described above for compound **4**. 4,4'-(*o*-tolylmethylene)bis(3-bromo-*N*,*N*-dimethylaniline) **1** (0.15 g, 0.30 mmol) in anhydrous THF (8.0 mL) was treated with *s*-BuLi (1.4M in cyclohexane) (0.47 mL, 0.66 mmol) and dichloro(phenyl)(vinyl)silane (92.0 μL, 0.45 mmol). The resulting residue was re-dissolved in DCM (10.0 mL), followed by treatment with *p*-chloranil (0.15 g, 0.60 mmol). The residue was purified by flash column chromatography (Silicycle column, 12 g, 0–5% MeOH in 1% v/v TFA/DCM for 10 min, hold at 5% MeOH isocratic for 5 min, and then increase to 15% gradient over 5 min) to provide **11** as an inseparable mixture of isomers (0.12 g, 80%) in dark blue color solid. ^{1}H NMR (500 MHz, $CDCl_3$) δ 7.62–7.48 (m, 3 H), 7.47–7.41 (m, 3 H), 7.38–7.31 (m, 2 H), 7.16–7.05 (m, 5 H), 6.63 (dd, J = 10.0, 3.0 Hz, 2 H), 6.58–6.42 (m, 2 H), 5.95 (dd, J = 19.5, 3.5 Hz, 1 H), 3.28 (s, 12 H), 2.04 and 2.01 (2 x s, 3 H) ppm; ^{13}C NMR (125 MHz, $CDCl_3$) δ 170.59, 154.17 (2 signals), 144.72 (2 signals), 142.08, 139.97, 138.16, 135.88, 135.41, 135.23, 131.25, 131.10, 131.00, 130.50, 129.24, 128.96, 128.24, 125.81 (2 signals), 122.45, 114.36, 40.91, 19.45 (2 signals) ppm; ^{19}F NMR (470 MHz, $CDCl_3$) δ −75.77 ppm; HRMS (ESI) *m/z*: $[M]^+$ calcd for $C_{32}H_{33}N_2Si$, 473.2408; found 473.2401.

***N*-(5-(3-Chloropropyl)-7-(dimethylamino)-5-methyl-10-(*o*-tolyl)dibenzo[*b*,*e*]silin-3(5*H*)-ylidene)-*N*-methylmethanaminium (12).**

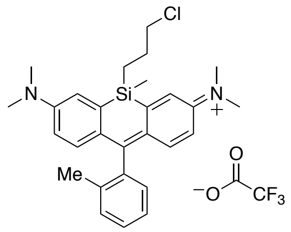

The same procedure was used as described above for compound **4**. 4,4'-(*o*-tolylmethylene)bis(3-bromo-*N*,*N*-dimethylaniline) **1** (0.15 g, 0.30 mmol) in anhydrous THF (8.0 mL) was treated with *s*-BuLi (1.4M in cyclohexane) (0.47 mL, 0.66 mmol) and dichloro(3-chloropropyl)(methyl)silane (70.0 μL, 0.45 mmol). The resulting residue was re-dissolved in DCM (10.0 mL), followed by treatment with *p*-chloranil (0.15 g, 0.60 mmol). The residue was purified by flash column chromatography (Silicycle column, 12 g, 0–15% MeOH in 1% v/v TFA/DCM, linear gradient for 20 min) to yield (70.0 mg, 50%) of the trifluoroacetate salt of **12** as an inseparable mixture of isomers in dark blue color solid. An analytically pure sample was obtained through further purification by reverse-phase HPLC (30–95% MeCN/H_2O, linear gradient, with constant 0.1% v/v TFA additive). ^{1}H NMR (500 MHz, $CDCl_3$) δ 7.46–7.41 (m, 1 H), 7.37–7.32 (m, 2 H), 7.19 (t, J = 3.0 Hz, 2 H), 7.11–7.06 (m, 3 H), 6.60 (dd, J = 9.5, 2.5 Hz, 2 H), 3.47 and 3.43 (2 x t, J = 7.0 Hz, 2 H), 3.35 (s, 12 H), 2.03 and 2.02 (2 x s, 3 H), 1.77–1.63 (m, 2 H), 1.22–1.16 (m, 2 H), 0.67 and 0.65 (2 x s, 3 H) ppm; ^{13}C NMR (125 MHz, $CDCl_3$) δ 170.47 (2 signals), 154.24 (2 signals), 147.14 (2 signals), 141.80 (2 signals), 138.46 (2 signals), 135.92, 135.66, 130.44 (2 signals), 129.18, 129.13, 129.12, 128.12, 128.02, 125.83 (2 signals), 121.06 (2 signals), 114.17, 47.39 (2 signals), 41.01, 26.99 (2 signals), 19.52, 13.68 (2 signals), −3.37 (2 signals) ppm; ^{19}F NMR (470 MHz, $CDCl_3$) δ −75.67 ppm; HRMS (ESI) *m/z*: $[M]^+$ calcd for $C_{28}H_{34}ClN_2Si$, 461.2174; found 461.2168.

***N*-(7-(Dimethylamino)-5-methyl-10-(*o*-tolyl)-5-(3,3,3-trifluoropropyl)dibenzo[*b*,*e*]silin-3(5*H*)-ylidene)-*N*-methylmethanaminium (13).**

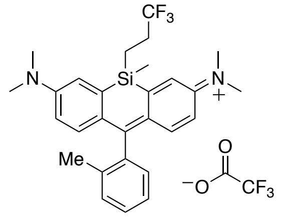

The same procedure was used as described above for compound **4**. 4,4'-(*o*-tolylmethylene)bis(3-bromo-*N*,*N*-dimethylaniline) **1** (0.15 g, 0.30 mmol) in anhydrous THF (8.0 mL) was treated with *s*-BuLi (1.4M in cyclohexane) (0.47 mL, 0.66 mmol) and dichloro(3,3,3-trifluoropropyl)(methyl)silane (95.0 μL, 0.45 mmol). The resulting residue was re-dissolved in DCM (10.0 mL), followed by treatment with *p*-chloranil (0.15 g, 0.60 mmol). The residue was purified by flash column chromatography (Silicycle column, 12 g, 0–5% MeOH in 1% v/v TFA/DCM for 10 min, hold at 5% MeOH isocratic for 5 min, and then increase to 15% gradient over 5 min) to provide **13** as an inseparable mixture of isomers (90.0 mg, 63%) in dark blue color solid. ^{1}H NMR (500 MHz, $CDCl_3$) δ 7.45–7.39 (m, 1 H), 7.37–7.29 (m, 2 H), 7.20–7.12 (m, 2 H), 7.10–7.02 (m, 3 H), 6.62 (dd, J = 9.5, 2.5 Hz, 2 H), 3.33 (s, 12 H), 2.01 and 2.00 (2 x s, 3 H), 1.99–1.84 (m, 2 H), 1.30–1.17 (m, 2 H), 0.70 and 0.69 (2 x s, 3 H) ppm; ^{13}C NMR (125 MHz, $CDCl_3$) δ 170.43 (2 signals), 154.25 (2 signals), 145.58 (2 signals), 141.80 (2 signals), 140.15, 138.19 (2 signals), 135.87, 135.38, 130.45, 130.40, 129.15 (2 signals), 128.65, 127.91, 127.80, 127.25(q, J = 269.3 Hz), 125.81, 120.92 (2 signals), 114.34, 40.92 (2 signals), 28.14 (2 x q, J = 30.2 Hz), 19.36 (2 signals), 8.49 (2 signals), −3.92 (2 signals) ppm; ^{19}F NMR (470 MHz, $CDCl_3$) δ −68.14, −68.23, −75.65 ppm; HRMS (ESI) *m/z*: $[M]^+$ calcd for $C_{28}H_{32}F_3N_2Si$, 481.2281; found 481.2273.

_N_-(7-(Dimethylamino)-5,5-dioctyl-10-(_o_-tolyl)dibenzo[_b_,_e_]silin-3(5_H_)-ylidene)-_N_-methylmethanaminium (14).

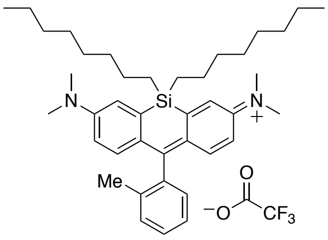

The same procedure was used as described above for compound **4**. 4,4'-(*o*-tolylmethylene)bis(3-bromo-*N*,*N*-dimethylaniline) **1** (0.15 g, 0.30 mmol) in anhydrous THF (8.0 mL) was treated with *s*-BuLi (1.4M in cyclohexane) (0.47 mL, 0.66 mmol) and dichlorodioctylsilane (0.16 mL, 0.45 mmol). The resulting residue was re-dissolved in DCM (10.0 mL), followed by treatment with *p*-chloranil (0.15 g, 0.60 mmol). The residue was purified by flash column chromatography (Silicycle column, 12 g, 0–15% MeOH in 1% v/v TFA/DCM, linear gradient for 20 min) to yield (0.10 g, 56%) of the trifluoroacetate salt of **14** as a dark blue color solid. An analytically pure sample was obtained through further purification by reverse-phase HPLC (30–95% MeCN/H_2O, linear gradient, with constant 0.1% v/v TFA additive). ^1H NMR (500 MHz, $CDCl_3$) δ 7.46–7.41 (m, 1 H), 7.37–7.31 (m, 2 H), 7.13–7.04 (m, 5 H), 6.62 (dd, J = 10.0, 3.0 Hz, 2 H), 3.34 (s, 12 H), 2.00 (s, 3 H), 1.29–1.12 (m, 24 H), 1.10–1.04 (m, 4 H), 0.87–0.81 (m, 6 H) ppm; ^{13}C NMR (125 MHz, $CDCl_3$) δ 170.96, 154.07, 147.58, 141.95, 138.51, 135.70, 130.46, 129.16, 128.97, 128.41, 125.81, 120.58, 114.16, 40.91, 33.18, 33.10, 31.94, 29.28, 29.22, 29.20, 23.65, 23.60, 22.76, 22.75, 19.43, 14.60, 14.20, 14.19, 14.16 ppm; ^{19}F NMR (470 MHz, $CDCl_3$) δ −75.72 ppm; HRMS (ESI) *m/z*: $[M]^+$ calcd for $C_{40}H_{59}N_2Si$, 595.4442; found 595.4430.

***N*-(7-(Dimethylamino)-5,5-dioctadecyl-10-(*o*-tolyl)dibenzo[*b*,*e*]silin-3(5*H*)-ylidene)-*N*-methylmethanaminium (15).**

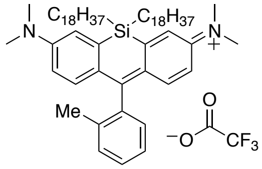

A degassed solution of tetrachlorosilane (1.48 g, 8.73 mmol) in anhydrous Et_2O (20.0 mL) under argon atmosphere was cooled to −78 °C in an acetone/dry ice bath. After 15 min, 0.5M solution of octadecylmagnesium chloride in THF (35.0 mL, 17.5 mmol) was added dropwise over 20 min. The resulting reaction mixture was then slowly warmed to room temperature and stirred for 2 h. the resulting reaction mixture was filtered through celite and concentrated under reduced pressure. The crude dichlorodioctadecylsilane was used without further purification to prepare **15**.

The same procedure was used as described above for compound **4**. 4,4'-(*o*-tolylmethylene)bis(3-bromo-*N*,*N*-dimethylaniline) **1** (0.15 g, 0.30 mmol) in anhydrous THF (8.0 mL) was treated with *s*-BuLi (1.4M in cyclohexane) (0.47 mL, 0.66 mmol) and dichlorodioctadecylsilane (0.27 mL, 0.45 mmol). The resulting residue was re-dissolved in DCM (10.0 mL), followed by treatment with *p*-chloranil (0.15 g, 0.60 mmol). The residue was purified by flash column chromatography (Silicycle column, 12 g, 0–5% MeOH in 1% v/v TFA/DCM for 10 min, hold at 5% MeOH isocratic for 5 min, and then increase to 15% gradient over 5 min) to provide **15** (26.0 mg, 10%) as a dark blue color solid. ^{1}H NMR (500 MHz, $CDCl_3$) δ 7.47–7.42 (m, 1 H), 7.37–7.32 (m, 2 H), 7.14–7.04 (m, 5 H), 6.63 (dd, J = 10.0, 3.0 Hz, 2 H), 3.33 (s, 12 H), 2.00 (s, 3 H), 1.30–1.04 (m, 64 H), 0.90–0.85 (m, 10 H) ppm; ^{13}C NMR (125 MHz, $CDCl_3$) δ 171.03, 154.07, 147.59, 141.98, 138.49, 135.69, 132.65, 130.46, 129.18, 128.98, 128.43, 125.82, 120.57, 114.16, 40.93, 33.22, 33.16, 32.07, 29.89, 29.85, 29.80, 29.69, 29.64, 29.50, 29.31, 23.68, 23.64, 22.84, 19.44, 14.63, 14.26, 14.17 ppm; ^{19}F NMR (470 MHz, $CDCl_3$) δ −75.84 ppm; HRMS (ESI) *m/z*: $[M]^+$ calcd for $C_{60}H_{99}N_2Si$, 875.7572; found 875.7550.

***N*-(5-((2*S*)-Bicyclo[2.2.1]hept-5-en-2-yl)-7-(dimethylamino)-5-methyl-10-(*o*-tolyl)dibenzo[*b*,*e*]silin-3(5*H*)-ylidene)-*N*-methylmethanaminium (16).**

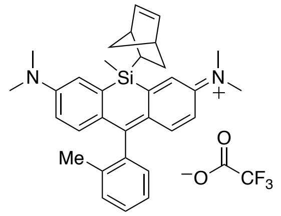

The same procedure was used as described above for compound **4**. 4,4'-(*o*-tolylmethylene)bis(3-bromo-*N*,*N*-dimethylaniline) **1** (0.15 g, 0.30 mmol) in anhydrous THF (8.0 mL) was treated with *s*-BuLi (1.4M in cyclohexane) (0.47 mL, 0.66 mmol) and dichloro(methyl)(5-bicyclo[2.2.1]hept-5-en-2-yl)silane (93.0 μL, 0.45 mmol). The resulting residue was re-dissolved in DCM (10.0 mL), followed by treatment with *p*-chloranil (0.15 g, 0.60 mmol). The residue was purified by flash column chromatography (Silicycle column, 12 g, 0–5% MeOH in 1% v/v TFA/DCM for 10 min, hold at 5% MeOH isocratic for 5 min, and then increase to 15% gradient over 5 min) to provide **16** as an inseparable mixture of endo and exo isomers (80.0 mg, 56%) in dark blue color solid. ^{1}H NMR (500 MHz, $CDCl_3$) (mixture of endo and exo isomers) δ 7.46–7.41 (m, 1 H), 7.37–7.31 (m, 2 H), 7.15–7.02 (m, 5 H), 6.64–6.57 (m, 2 H), 6.15–5.64 (m, 2 H), 3.34 (2 x s, 12 H), 2.99–2.67 (m, 2 H), 2.11–1.97 (m, 3 H), 1.61–1.02 (m, 4 H), 0.77–0.67 (m, 3 H), 0.65–0.55 (m, 1 H) ppm; ^{19}F NMR (470 MHz, $CDCl_3$) δ −75.81 ppm; HRMS (ESI) *m/z*: $[M]^+$ calcd for $C_{32}H_{37}N_2Si$, 477.2721; found 477.2714.

***N*-(7-(Dimethylamino)-5-(3-(dimethylamino)phenyl)-5-methyl-10-(*o*-tolyl)dibenzo[*b*,*e*]silin-3(5*H*)-ylidene)-*N*-methylmethanaminium (17).**

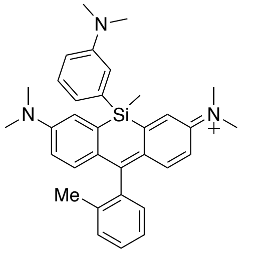

A degassed solution of 3-bromo-*N*,*N*-dimethylaniline (1.37 g, 6.82 mmol) in anhydrous Et_2O (20.0 mL) under argon atmosphere was cooled to −78 °C in an acetone/dry ice bath. After 15 min, *s*-BuLi (1.4M in cyclohexane) (5.35 mL, 7.16 mmol) was added dropwise over 10 min. The resulting reaction mixture was stirred at −78 °C for additional one hour and was then added dropwise to a solution of methyltrichlorosilane (4.02 mL, 34.1 mmol) in anhydrous Et_2O (20.0 mL) under argon atmosphere at −78 °C. The resulting reaction mixture was then slowly warmed to room temperature and stirred for 2 h. The resulting reaction mixture was filtered through Celite and concentrated under reduced pressure. The crude dichloro(methyl)(3-(dimethylamino)phenyl)silane was used without further purification to prepare **17**.

The same procedure was used as described above for compound **4**. 4,4'-(*o*-tolylmethylene)bis(3-bromo-*N*,*N*-dimethylaniline) **1** (0.15 g, 0.30 mmol) in anhydrous THF (8.0 mL) was treated with *s*-BuLi (1.4M in cyclohexane) (0.47 mL, 0.66 mmol) and dichloro(methyl)(3-(dimethylamino)phenyl)silane (0.11 g, 0.45 mmol). The resulting residue was re-dissolved in DCM (10.0 mL), followed by treatment with *p*-chloranil (0.15 g, 0.60 mmol). The residue was purified by flash column chromatography (Silicycle column, 12 g, 0–5% MeOH in DCM for 10 min, hold at 5% MeOH isocratic for 5 min, and then increase to 15% gradient over 5 min) to provide **17** as an inseparable mixture of isomers (50.0 mg, 34%) in dark blue color solid. ^{1}H NMR (500 MHz, $CDCl_3$) δ 7.45–7.40 (m, 1 H), 7.37–7.27 (m, 3 H), 7.14–7.06 (m, 5 H), 6.92–6.78 (m, 3 H), 6.66 (dd, J = 9.5, 3.0 Hz, 2 H), 3.34 (s, 12 H), 2.95 and 2.92 (2 x s, 6 H), 2.06 and 2.05 (2 x s, 3 H), 0.93 and 0.89 (2 x s, 3 H) ppm; ^{13}C NMR (125 MHz, $CDCl_3$) δ 170.03, 154.23 (2 signals), 147.01, 141.70 (2 signals), 138.46, 135.85, 133.62, 131.00, 130.46 (2 signals), 129.62, 129.12,

128.95, 128.04, 125.85 (2 signals), 122.41, 121.70, 117.79, 114.90, 114.39, 41.22, 40.63, 19.59 (2 signals), –2.79 (2 signals) ppm; HRMS (ESI) *m/z*: $[M]^+$ calcd for $C_{33}H_{38}N_3Si$, 504.2830; found 504.2824.

1,11,13,13-Tetramethyl-6-(*o*-tolyl)-2,3,4,8,9,10,11,13-octahydrosilino[3,2-*g*:5,6-*g′*]diquinolin-1-ium (18).

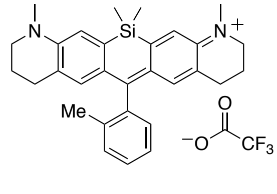

A degassed solution of **2** (0.20 g, 0.36 mmol) in anhydrous THF (10.0 mL) under argon atmosphere was cooled to −78 °C in an acetone/dry ice bath. After 15 min, *s*-BuLi (1.4M in cyclohexane) (0.57 mL, 0.79 mmol) was added dropwise over 5 min. The resulting reaction mixture was stirred at −78 °C for additional 30 min. At the same temperature, dichlorodimethylsilane (57.0 μL, 0.47 mmol) dissolved in anhydrous THF (10.0 mL) was added dropwise over 10 min. The reaction mixture was then slowly warmed to room temperature and stirred overnight. The reaction mixture was then cooled to ~ 5 °C and quenched by addition of 2N HCl (2.0 mL) and stirred at room temperature for 10 min. $NaHCO_3$ (25.0 mL) was added and then extracted with dichloromethane (50.0 mL), which was dried (Na_2SO_4), filtered, and evaporated under reduced pressure. The residue was re-dissolved in anhydrous DCM (20.0 mL) and treated with *p*-chloranil (0.18 g, 0.72 mmol) at room temperature, and then the mixture solution was stirred for 2 h. The solvent was then evaporated under reduced pressure, and the residue was purified by flash column chromatography (Silicycle column, 12 g, 0–5% MeOH in 1% v/v TFA/DCM for 10 min, hold at 5% MeOH isocratic for 5 min, and then increase to 15% gradient over 5 min) to yield (40.0 mg, 25%) of the trifluoroacetate salt of **18** as a dark blue-green solid. ^1H NMR (500 MHz, $CDCl_3$) δ 7.45–7.40 (m, 1 H), 7.37–7.30 (m, 2 H), 7.06 (d, J = 7.5 Hz, 1 H), 7.01 (s, 2 H), 6.66 (s, 2 H), 3.58 (t, J = 5.5 Hz, 4 H), 3.32 (s, 6 H), 2.50 (t, J = 6.0 Hz, 4 H), 2.20 (s, 3 H), 1.98–1.89 (m, 4 H), 0.55 (s, 3 H), 0.53 (s, 3 H) ppm; ^{13}C NMR (125 MHz, $CDCl_3$) δ 168.74, 152.05, 147.75, 138.83, 138.36, 135.70, 130.36, 129.06, 128.91, 127.98,

125.75, 124.68, 119.63, 52.70, 39.88, 27.51, 21.00, 19.61, −0.67, −1.11 ppm; ^{19}F NMR (470 MHz, $CDCl_3$) δ −75.85 ppm; HRMS (ESI) *m/z*: $[M]^+$ calcd for $C_{30}H_{35}N_2Si$, 451.2564; found 451.2559.

1,11-Dimethyl-6-(*o*-tolyl)-13,13-divinyl-2,3,4,8,9,10,11,13-octahydrosilino[3,2-*g*:5,6-*g′*]diquinolin-1-ium (19).

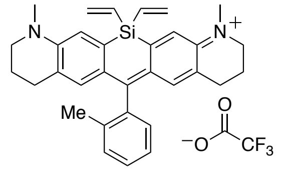

The same procedure was used as described above for compound **18**. A solution of **2** (0.20 g, 0.36 mmol) in anhydrous THF (10.0 mL) was treated with *s*-BuLi (1.4M in cyclohexane) (0.57 mL, 0.79 mmol) and dichlorodivinyllsilane (67.0 μL, 0.47 mmol). The resulting residue was re-dissolved in DCM (20.0 mL), followed by treatment with *p*-chloranil (0.18 g, 0.72 mmol) to provide **19** (45.0 mg, 26%) as a green solid. ^{1}H NMR (500 MHz, $CDCl_3$) δ 7.45–7.40 (m, 1 H), 7.36–7.30 (m, 2 H), 7.06 (d, J = 8.0 Hz, 1 H), 6.96 (s, 2 H), 6.67 (s, 2 H), 6.42–6.26 (m, 4 H), 6.01 (dd, J = 19.5, 3.5 Hz, 1 H), 5.93 (dd, J = 16.5, 6.0 Hz, 1 H), 3.60 (t, J = 5.5 Hz, 4 H), 3.30 (s, 6 H), 2.51 (t, J = 6.0 Hz, 4 H), 2.02 (s, 3 H), 1.99–1.90 (m, 4 H) ppm; ^{13}C NMR (125 MHz, $CDCl_3$) δ 168.48, 151.96, 143.18, 139.28, 138.83, 138.60, 138.53, 135.73, 131.44, 131.07, 130.40, 129.10, 128.98, 128.31, 125.75, 124.99, 121.09, 52.78, 39.89, 27.49, 20.94, 19.60 ppm; ^{19}F NMR (470 MHz, $CDCl_3$) δ −75.80 ppm; HRMS (ESI) *m/z*: $[M]^+$ calcd for $C_{32}H_{35}N_2Si$, 475.2564; found 475.2557.

1,11-Dimethyl-13,13-diphenyl-6-(*o*-tolyl)-2,3,4,8,9,10,11,13-octahydrosilino[3,2-*g*:5,6-*g′*]diquinolin-1-ium (20).

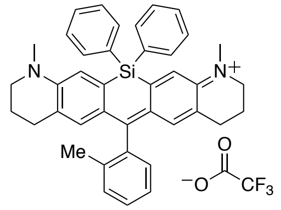

The same procedure was used as described above for compound **18**. A solution of **2** (0.20 g, 0.36 mmol) in anhydrous THF (10.0 mL) was treated with *s*-BuLi (1.4M in cyclohexane) (0.57 mL, 0.79 mmol) and dichlorodiphenylsilane (0.10 mL, 0.47 mmol). The resulting residue was re-dissolved in DCM (20.0 mL), followed by treatment with *p*-chloranil (0.18 g, 0.72 mmol) to provide **20** (50.0 mg, 24%) as a green solid. ^{1}H NMR (500 MHz, $CDCl_3$) δ 7.67–7.62 (m, 2 H), 7.61–7.57 (m, 2 H), 7.56–7.42 (m, 7 H), 7.38–7.32 (m, 2 H), 7.10 (d, J = 7.0 Hz, 1 H), 6.99 (s, 2 H), 6.71 (s, 2 H), 3.58 (t, J = 5.0 Hz, 4 H), 3.17 (s, 6 H), 2.52 (t, J = 5.5 Hz, 4 H), 2.03 (s, 3 H), 1.99–1.89 (m, 4 H) ppm; ^{13}C NMR (125 MHz, $CDCl_3$) δ 168.50, 151.96, 143.99, 138.56, 135.98, 135.80, 135.73, 131.95, 131.39, 131.23, 131.16, 130.47, 129.13, 129.06, 128.96, 128.94, 128.62, 125.81, 125.17, 121.61, 52.79, 39.85, 27.47, 20.89, 19.62 ppm; ^{19}F NMR (470 MHz, $CDCl_3$) δ −75.83 ppm; HRMS (ESI) *m/z*: $[M]^+$ calcd for $C_{40}H_{39}N_2Si$, 575.2877; found 575.2871.

13-(3-Chloropropyl)-1,11,13-trimethyl-6-(*o*-tolyl)-2,3,4,8,9,10,11,13-octahydrosilino[3,2-*g*:5,6-*g′*]diquinolin-1-ium (21).

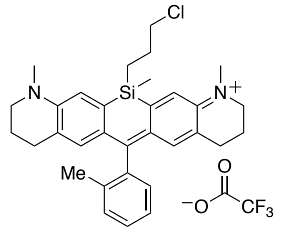

The same procedure was used as described above for compound **18**. A solution of **2** (0.20 g, 0.36 mmol) in anhydrous THF (10.0 mL) was treated with *s*-BuLi (1.4M in cyclohexane) (0.57 mL, 0.79 mmol) and dichloro(3-chloropropyl)(methyl)silane (73.0 µL, 0.47 mmol). The resulting residue was re-dissolved in DCM (20.0 mL), followed by treatment with *p*-

chloranil (0.18 g, 0.72 mmol) to provide **21** as an inseparable mixture of isomers (48.0 mg, 26%) in green color solid. ^{1}H NMR (500 MHz, $CDCl_3$) δ 7.46–7.40 (m, 1 H), 7.37–7.30 (m, 2 H), 7.06 (d, J = 8.0 Hz, 1 H), 7.04 (s, 2 H), 6.65 (s, 2 H), 3.59 (t, J = 5.5 Hz, 4 H), 3.51–3.41 (m, 2 H), 3.34 (s, 6 H), 2.51 (t, J = 6.0 Hz, 4 H), 2.03 and 2.02 (2 x s, 3 H), 1.99–1.88 (m, 4 H), 1.78–1.64 (m, 2 H), 1.21–1.10 (m, 2 H), 0.62 and 0.60 (2 x s, 3 H) ppm; ^{13}C NMR (125 MHz, $CDCl_3$) δ 168.54 (2 signals), 152.04 (2 signals), 146.01 (2 signals), 138.79 (2 signals), 138.33 (2 signals), 135.80, 135.57, 130.38 (2 signals), 129.23, 128.96, 128.94, 128.91, 128.32, 128.22, 125.78 (2 signals), 124.83, 119.91 (2 signals), 52.79, 47.57 (2 signals), 40.07, 27.51, 27.07 (2 signals), 20.96, 19.67, 13.87 (2 signals), −3.09 (2 signals) ppm; ^{19}F NMR (470 MHz, $CDCl_3$) δ −75.68 ppm; HRMS (ESI) *m/z*: $[M]^+$ calcd for $C_{32}H_{38}ClN_2Si$, 513.2487; found 513.2483.

1,9,11,11-Tetramethyl-5-(*o*-tolyl)-2,3,7,8,9,11-hexahydrosilino[3,2-*f*:5,6-*f'*]diindol-1-ium (22).

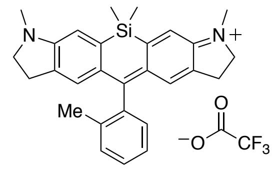

The same procedure was used as described above for compound **18**. A solution of **3** (0.20 g, 0.38 mmol) in anhydrous THF (10.0 mL) was treated with *s*-BuLi (1.4M in cyclohexane) (0.60 mL, 0.84 mmol) and dichlorodimethyllsilane (60.0 μL, 0.50 mmol). The resulting residue was re-dissolved in DCM (20.0 mL), followed by treatment with *p*-chloranil (0.19 g, 0.76 mmol) to provide **22** (60.0 mg, 37%) as a green solid. ^{1}H NMR (500 MHz, $CDCl_3$) δ 7.46–7.41 (m, 1 H), 7.38–7.31 (m, 2 H), 7.06 (d, J = 7.5 Hz, 1 H), 6.88 (s, 2 H), 6.67 (s, 2 H), 3.82 (t, J = 7.0 Hz, 4 H), 3.21 (s, 6 H), 2.95 (t, J = 7.5 Hz, 4 H), 2.03 (s, 3 H), 0.56 (s, 3 H), 0.53 (s, 3 H) ppm; ^{13}C NMR (125 MHz, $CDCl_3$) δ 166.99, 157.08, 151.09, 139.58, 135.70, 133.22, 132.95, 130.49, 128.96, 128.90, 128.79, 125.97, 114.44, 54.80, 33.87, 26.57, 19.50, −0.94, −1.36 ppm; ^{19}F NMR (470 MHz, $CDCl_3$) δ −75.85 ppm; HRMS (ESI) *m/z*: $[M]^+$ calcd for $C_{28}H_{31}N_2Si$, 423.2251; found 423.2246.

1,9-Dimethyl-5-(*o*-tolyl)-11,11-divinyl-2,3,7,8,9,11-hexahydrosilino[3,2-*f*:5,6-*f'*]diindol-1-ium (23).

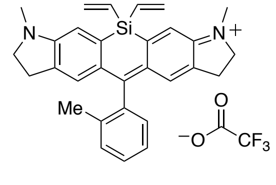

The same procedure was used as described above for compound **18**. A solution of **3** (0.20 g, 0.38 mmol) in anhydrous THF (10.0 mL) was treated with *s*-BuLi (1.4M in cyclohexane) (0.60 mL, 0.84 mmol) and dichlorodivinyllsilane (70.0 μL, 0.50 mmol). The resulting residue was re-dissolved in DCM (20.0 mL), followed by treatment with *p*-chloranil (0.19 g, 0.76 mmol) to provide **23** (80.0 mg, 47%) as a green solid. ^{1}H NMR (500 MHz, $CDCl_3$) δ 7.46–7.41 (m, 1 H), 7.38–7.32 (m, 2 H), 7.05 (d, J = 7.5 Hz, 1 H), 6.80 (s, 2 H), 6.68 (s, 2 H), 6.43–6.24 (m, 4 H), 6.02 (dd, J = 19.5, 3.0 Hz, 1 H), 5.93 (dd, J = 19.0, 4.0 Hz, 1 H), 3.85 (t, J = 8.0 Hz, 4 H), 3.20 (s, 6 H), 2.96 (t, J = 7.5 Hz, 4 H), 2.02 (s, 3 H) ppm; ^{13}C NMR (125 MHz, $CDCl_3$) δ 166.84, 157.00, 146.48, 139.54, 139.32, 139.06, 135.72, 133.40, 133.30, 131.04, 130.66, 130.54, 129.26, 128.98, 128.87, 125.97, 115.72, 54.83, 33.85, 26.56, 19.46 ppm; ^{19}F NMR (470 MHz, $CDCl_3$) δ −75.85 ppm; HRMS (ESI) *m/z*: $[M]^+$ calcd for $C_{30}H_{31}N_2Si$, 447.2251; found 447.2246.

1,9-Dimethyl-11,11-diphenyl-5-(*o*-tolyl)-2,3,7,8,9,11-hexahydrosilino[3,2-*f*:5,6-*f'*]diindol-1-ium (24).

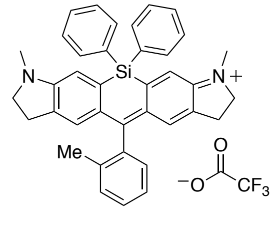

The same procedure was used as described above for compound **18**. A solution of **3** (0.20 g, 0.38 mmol) in anhydrous THF (10.0 mL) was treated with *s*-BuLi (1.4M in cyclohexane) (0.60 mL, 0.84 mmol) and dichlorodiphenyllsilane (0.11 mL, 0.50 mmol). The resulting residue was re-dissolved in DCM (20.0 mL), followed by treatment with *p*-chloranil (0.19 g, 0.76 mmol) to provide **24** (78.0 mg, 38%) as a green solid. ^{1}H NMR (500 MHz, $CDCl_3$)

δ 7.67–7.62 (m, 2 H), 7.61–7.56 (m, 2 H), 7.55–7.42 (m, 7 H), 7.39–7.33 (m, 2 H), 7.09 (d, *J* = 7.0 Hz, 1 H), 6.79 (s, 2 H), 6.73 (s, 2 H), 3.85 (t, *J* = 7.5 Hz, 4 H), 3.10 (s, 6 H), 2.97 (t, *J* = 7.5 Hz, 4 H), 2.02 (s, 3 H) ppm; ^{13}C NMR (125 MHz, $CDCl_3$) δ 166.87, 156.98, 147.42, 139.28, 136.12, 135.91, 135.70, 133.51, 133.44, 131.48, 131.30, 131.22, 130.91, 130.61, 129.57, 129.02, 128.98, 128.95, 126.03, 116.19, 54.93, 33.94, 26.59, 19.49 ppm; ^{19}F NMR (470 MHz, $CDCl_3$) δ −75.85 ppm; HRMS (ESI) *m/z*: $[M]^+$ calcd for $C_{38}H_{35}N_2Si$, 547.2564; found 547.2557.

11-(3-Chloropropyl)-1,9,11-trimethyl-5-(*o*-tolyl)-2,3,7,8,9,11-hexahydrosilino[3,2-*f*:5,6-*f'*]diindol-1-ium (25).

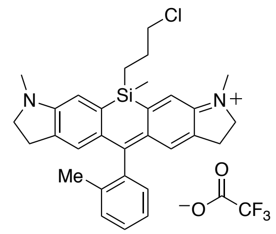

The same procedure was used as described above for compound **18**. A solution of **3** (0.20 g, 0.38 mmol) in anhydrous THF (10.0 mL) was treated with *s*-BuLi (1.4M in cyclohexane) (0.60 mL, 0.84 mmol) and dichloro(3-chloropropyl)(methyl)silane (77.0 µL, 0.50 mmol). The resulting residue was re-dissolved in DCM (20.0 mL), followed by treatment with *p*-chloranil (0.19 g, 0.76 mmol) to provide **25** as an inseparable mixture of isomers (75.0 mg, 41%) in green color solid. ^{1}H NMR (500 MHz, $CDCl_3$) δ 7.46–7.40 (m, 1 H), 7.38–7.32 (m, 2 H), 7.04 (t, *J* = 7.5 Hz, 1 H), 6.91 and 6.90 (2 x s, 2 H), 6.66 and 6.65 (2 x s, 2 H), 3.83 (t, *J* = 8.0 Hz, 4 H), 3.46 and 3.42 (2 x t, *J* = 7.0 Hz, 2 H), 3.23 (s, 6 H), 2.95 (t, *J* = 8.0 Hz, 4 H), 2.03 and 2.02 (2 x s, 3 H), 1.74–1.61 (m, 2 H), 1.18–1.10 (m, 2 H), 0.63 and 0.61 (2 x s, 3 H) ppm; ^{13}C NMR (125 MHz, $CDCl_3$) δ 166.91 (2 signals), 157.06 (2 signals), 149.35 (2 signals), 139.55 (2 signals), 135.81, 135.54, 133.21 (2 signals), 133.15, 130.55, 130.46, 129.26, 129.17, 129.12, 128.84, 128.81, 126.01 (2 signals), 114.59 (2 signals), 54.79, 47.37 (2 signals), 33.90, 26.98 (2 signals), 26.56, 19.58 (2 signals), 13.88 (2 signals), −3.59 (2 signals) ppm; ^{19}F NMR (470 MHz, $CDCl_3$) δ −75.81 ppm; HRMS (ESI) *m/z*: $[M]^+$ calcd for $C_{30}H_{34}ClN_2Si$, 485.2174; found 485.2170.

Alternative synthesis of Si-Bridge rhodamines

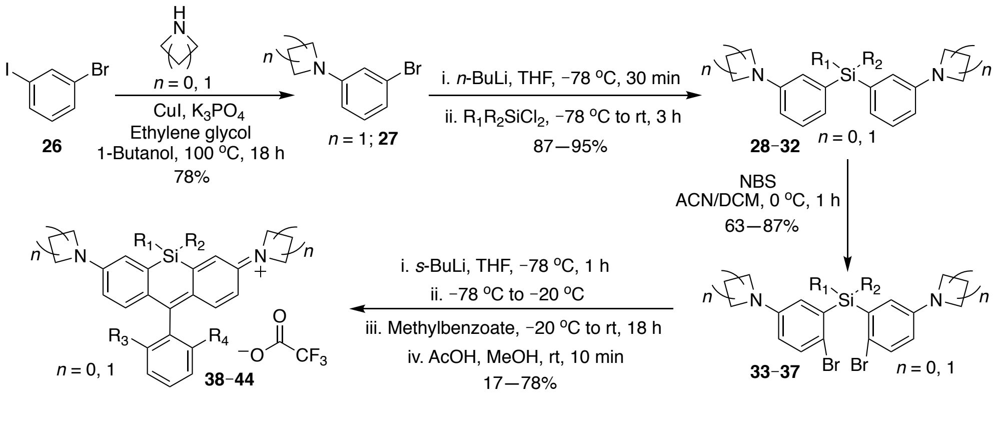

1-(3-Bromophenyl)azetidine (27)[6].

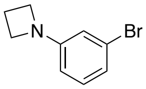

An oven-dried sealed tube was charged with CuI (0.41 g, 2.12 mmol) and K_3PO_4 (13.6 g, 63.9 mmol). The vial was capped with rubber septum and evacuated/backfilled with argon. Anhydrous 1-butanol (40.0 mL) was added, followed by ethylene glycol (2.89 mL, 51.1 mmol), 3-bromoiodobenzene **26** (2.71 mL, 21.3 mmol), and azetidine (1.72 mL, 25.6 mmol). The vial was sealed with teflon cap under argon and the reaction mixture was stirred at 100 °C for 18 h. The reaction mixture was cooled to room temperature and diluted with saturated NH_4Cl solution (100 mL). After extraction with EtOAc (2 x 150 mL), the combined extracts were washed with saturated NaCl solution (150 mL), dried (Na_2SO_4), filtered, and evaporated under reduced pressure. The resulting residue was purified by flash column chromatography (Silicycle column, 50 g, 0–10% EtOAc/Hexanes, linear gradient for 20 min) to provide **27** (3.50 g, 78%) as a colorless oil. ^{1}H NMR (500 MHz, $CDCl_3$) δ 7.04 (t, J = 8.0 Hz, 1 H), 6.84–6.80 (m, 1 H), 6.55 (t, J = 2.0 Hz, 1 H), 6.33 (dd, J = 8.0, 2.0 Hz, 1 H), 3.87 (t, J = 7.0 Hz, 4 H), 2.37 (p, J = 7.0 Hz, 2 H) ppm; ^{13}C NMR (125 MHz, $CDCl_3$) δ 153.34, 130.30, 123.16, 120.03, 114.20, 109.95, 52.43, 16.99 ppm; HRMS (ESI) *m/z*: $[M + H]^+$ calcd for $C_9H_{11}BrN$, 212.0069; found 212.0068.

Bis(3-(azetidin-1-yl)phenyl)dimethylsilane (28a)[6].

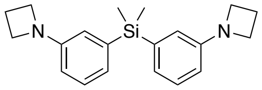

A degassed solution of **27** (1.20 g, 5.66 mmol) in anhydrous THF (25.0 mL) under argon atmosphere was cooled to −78 °C in an acetone/dry ice bath. After 15 min, *n*-BuLi (2.5M in hexanes) (2.26 mL, 5.66 mmol) was added dropwise over 10 min. The resulting reaction mixture was stirred at −78 °C for additional 30 min. At the same temperature, dichlorodimethylsilane (0.29 mL, 2.38 mmol) dissolved in anhydrous THF (5.0 mL) was added dropwise over 5 min. The dry ice bath was removed, and reaction mixture was stirred at room temperature for 3 h. It was subsequently quenched with saturated NH_4Cl (25.0 mL), diluted with water (25.0 mL), and then extracted with EtOAc (2 x 50.0 mL), the combined extracts were washed with saturated NaCl solution (50.0 mL), dried (Na_2SO_4), filtered, and evaporated under reduced pressure. The resulting residue was purified by flash column chromatography (Silicycle column, 50 g, 0–15% EtOAc/Hexanes, linear gradient for 20 min) to provide **28a** (0.72 g, 94%) as a colorless gummy solid. ^{1}H NMR (500 MHz, $CDCl_3$) δ 7.20 (t, J = 7.5 Hz, 2 H), 6.90 (dt, J = 7.0, 1.0 Hz, 2 H), 6.61 (d, J = 2.5 Hz, 2 H), 6.47 (dd, J = 8.0, 2.0 Hz, 2 H), 3.86 (t, J = 7.5 Hz, 8 H), 2.34 (p, J = 7.0 Hz, 4 H), 0.50 (s, 6 H) ppm; ^{13}C NMR (125 MHz, $CDCl_3$) δ 151.60, 138.94, 128.36, 123.48, 116.91, 112.31, 52.63, 17.16, −2.10 ppm; HRMS (ESI) *m/z*: $[M + H]^+$ calcd for $C_{20}H_{27}N_2Si$, 323.1938; found 323.1935.

3,3'-(Dimethylsilanediyl)bis(*N*,*N*-dimethylaniline) (28b)[6].

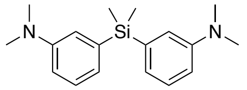

The same procedure was used as described above for compound **28a**. A solution of 3-bromo-*N*,*N*-dimethylaniline (2.50 g, 12.5 mmol) in anhydrous THF (30.0 mL) was treated with *n*-BuLi (2.5M in hexanes) (5.0 mL, 12.5 mmol) and dichlorodimethylsilane (0.63 mL, 5.25 mmol) to provide **28b** (1.50 g, 95%) as a colorless gummy solid. ^{1}H NMR (500 MHz, $CDCl_3$) δ 7.30–7.27 (m, 2 H), 6.98 (d, J = 2.5 Hz, 2 H), 6.95 (d, J = 7.5 Hz, 2 H), 6.80 (dd,

J = 8.0, 2.0 Hz, 2 H), 2.96 (s, 12 H), 0.57 (s, 6 H) ppm; ^{13}C NMR (125 MHz, $CDCl_3$) δ 150.06, 139.09, 128.61, 122.87, 118.48, 113.70, 40.83, −2.02 ppm; HRMS (ESI) *m/z*: [M + H]$^+$ calcd for $C_{18}H_{27}N_2Si$, 299.1938; found 299.1935.

Bis(3-(azetidin-1-yl)phenyl)divinylsilane (29).

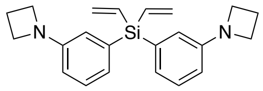

The same procedure was used as described above for compound **28a**. A solution of **27** (1.10 g, 5.19 mmol) in anhydrous THF (25.0 mL) was treated with *n*-BuLi (2.5M in hexanes) (2.10 mL, 5.19 mmol) and dichlorodivinylsilane (0.31 mL, 2.18 mmol) to provide **29** (0.70 g, 92%) as a colorless gummy solid. ^{1}H NMR (500 MHz, $CDCl_3$) δ 7.21 (t, J = 7.5 Hz, 2 H), 6.90 (d, J = 7.5 Hz, 2 H), 6.63 (d, J = 2.0 Hz, 2 H), 6.49 (dd, J = 8.5, 2.0 Hz, 2 H), 6.46 (dd, J = 20.0, 14.5 Hz, 2 H), 6.22 (dd, J = 14.5, 3.5 Hz, 2 H), 5.81 (dd, J = 20.0, 3.5 Hz, 2 H), 3.85 (t, J = 7.0 Hz, 8 H), 2.34 (p, J = 7.0 Hz, 4 H) ppm; ^{13}C NMR (125 MHz, $CDCl_3$) δ 151.66, 136.11, 134.84, 134.42, 128.38, 124.76, 118.21, 112.65, 52.61, 17.16 ppm; HRMS (ESI) *m/z*: [M + H]$^+$ calcd for $C_{22}H_{27}N_2Si$, 347.1938; found 347.1932.

Bis(3-(azetidin-1-yl)phenyl)diphenylsilane (30).

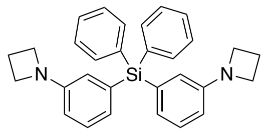

The same procedure was used as described above for compound **28a**. A solution of **27** (1.10 g, 5.19 mmol) in anhydrous THF (25.0 mL) was treated with *n*-BuLi (2.5M in hexanes) (2.10 mL, 5.19 mmol) and dichlorodiphenylsilane (0.46 mL, 2.18 mmol) to provide **30** (0.85 g, 87%) as a colorless gummy solid. ^{1}H NMR (500 MHz, $CDCl_3$) δ 7.61–7.56 (m, 4 H), 7.42–7.37 (m, 2 H), 7.36–7.32 (m, 4 H), 7.21 (t, J = 7.5 Hz, 2 H), 6.92 (d, J = 7.0 Hz, 2 H), 6.67 (d, J = 2.0 Hz, 2 H), 6.52 (dd, J = 8.0, 2.0 Hz, 2 H), 3.80 (t, J = 7.5 Hz, 8 H), 2.31 (p, J = 7.5 Hz, 4 H) ppm; ^{13}C NMR (125 MHz, $CDCl_3$) δ 151.59, 136.58, 135.72,

134.94, 134.72, 130.20, 129.46, 128.37, 127.79, 125.77, 119.29, 112.69, 52.58, 17.14 ppm; HRMS (ESI) *m/z*: $[M + H]^+$ calcd for $C_{30}H_{31}N_2Si$, 447.2251; found 447.2248.

1,1'-(((3-Chloropropyl)(methyl)silanediyl)bis(3,1-phenylene))bis(azetidine) (31a).

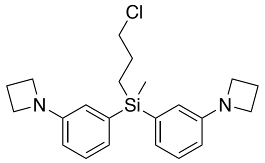

The same procedure was used as described above for compound **28a**. A solution of **27** (2.16 g, 10.2 mmol) in anhydrous THF (30.0 mL) was treated with *n*-BuLi (2.5M in hexanes) (4.07 mL, 10.2 mmol) and dichloro(3-chloropropyl)(methyl)silane (0.67 mL, 4.27 mmol) to provide **31a** (1.50 g, 91%) as a colorless gummy solid. ^{1}H NMR (500 MHz, $CDCl_3$) δ 7.20 (t, J = 7.5 Hz, 2 H), 6.88 (d, J = 7.5 Hz, 2 H), 6.60 (d, J = 2.0 Hz, 2 H), 6.48 (dd, J = 8.0, 2.0 Hz, 2 H), 3.87 (t, J = 7.0 Hz, 8 H), 3.50 (t, J = 7.0 Hz, 2 H), 2.35 (p, J = 7.5 Hz, 4 H), 1.87–1.79 (m, 2 H), 1.16–1.06 (m, 2 H), 0.51 (s, 3 H) ppm; ^{13}C NMR (125 MHz, $CDCl_3$) δ 151.58, 137.21, 128.46, 123.71, 117.13, 112.52, 52.63, 48.22, 27.78, 17.15, 12.19, −4.17 ppm; HRMS (ESI) *m/z*: $[M + H]^+$ calcd for $C_{22}H_{30}ClN_2Si$, 385.1861; found 385.1858.

3,3'-((3-Chloropropyl)(methyl)silanediyl)bis(*N*,*N*-dimethylaniline) (31b).

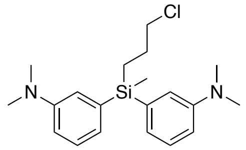

The same procedure was used as described above for compound **28a**. A solution of 3-bromo-*N*,*N*-dimethylaniline (5.0 g, 25.0 mmol) in anhydrous THF (50.0 mL) was treated with *n*-BuLi (2.5M in hexanes) (10.0 mL, 25.0 mmol) and dichloro(3-chloropropyl)(methyl)silane (1.64 mL, 10.5 mmol) to provide **31b** (3.60 g, 95%) as a colorless gummy solid. ^{1}H NMR (500 MHz, $CDCl_3$) δ 7.25 (t, J = 8.0 Hz, 2 H), 6.93 (d, J = 2.0 Hz, 2 H), 6.90 (d, J = 7.5 Hz, 2 H), 6.78 (dd, J = 8.0, 2.0 Hz, 2 H), 3.52 (t, J = 7.0

Hz, 2 H), 2.94 (s, 12 H), 1.92–1.83 (m, 2 H), 1.20–1.13 (m, 2 H), 0.55 (s, 3 H) ppm; ^{13}C NMR (125 MHz, $CDCl_3$) δ 150.07, 137.36, 128.71, 123.04, 118.64, 113.84, 48.25, 40.80, 27.85, 12.31, −4.09 ppm; HRMS (ESI) *m/z*: $[M + H]^+$ calcd for $C_{20}H_{30}ClN_2Si$, 361.1861; found 361.1855.

3,3'-(Silolane-1,1-diyl)bis(*N*,*N*-dimethylaniline) (32).

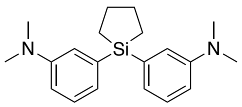

The same procedure was used as described above for compound **28a**. A solution of 3-bromo-*N*,*N*-dimethylaniline (2.0 g, 10.0 mmol) in anhydrous THF (30.0 mL) was treated with *n*-BuLi (2.5M in hexanes) (4.0 mL, 10.0 mmol) and cyclopentyldichlorosilane (0.65 g, 4.19 mmol) to provide **32** (1.20 g, 88%) as a colorless gummy solid. ^{1}H NMR (500 MHz, $CDCl_3$) δ 7.29–7.23 (m, 2 H), 6.97 (d, J = 2.5 Hz, 2 H), 6.75 (d, J = 7.0 Hz, 2 H), 6.79 (dd, J = 8.5, 3.0 Hz, 2 H), 2.94 (s, 12 H), 1.81 (p, J = 3.5 Hz, 4 H), 1.12 (t, J = 7.0 Hz, 4 H) ppm; ^{13}C NMR (125 MHz, $CDCl_3$) δ 150.06, 137.79, 128.65, 123.37, 118.97, 113.75, 40.79, 27.98, 12.52 ppm; HRMS (ESI) *m/z*: $[M + H]^+$ calcd for $C_{20}H_{29}N_2Si$, 325.2095; found 325.2089.

Bis(5-(azetidin-1-yl)-2-bromophenyl)dimethylsilane (33a).

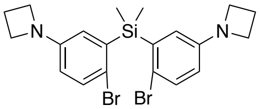

A solution of **28a** (0.70 g, 2.17 mmol) in a mixture of anhydrous ACN/DCM (2:1, 30.0 mL) under argon atmosphere was cooled to 0 °C in an ice-water bath. After 10 min, NBS (0.78 g, 4.38 mmol) was added in small portions over 10 min. The resulting reaction mixture was stirred at 0 °C for 1 h. Saturated $NaHCO_3$ solution (25.0 mL) was added to the reaction mixture. After extraction with DCM (3 x 25.0 mL), the combined extracts were washed with saturated NaCl solution (25.0 mL), dried (Na_2SO_4), filtered, and evaporated under reduced pressure. The resulting residue was purified by flash column

chromatography (Silicycle column, 25 g, 0–15% EtOAc/Hexanes, linear gradient for 20 min) to provide **33a** (0.65 g, 63%) as a white solid. ^{1}H NMR (500 MHz, $CDCl_3$) δ 7.31 (d, J = 8.5 Hz, 2 H), 6.51 (d, J = 2.5 Hz, 2 H), 6.31 (dd, J = 8.5, 3.0 Hz, 2 H), 3.81 (t, J = 7.5 Hz, 8 H), 2.33 (p, J = 7.5 Hz, 4 H), 0.71 (s, 6 H) ppm; ^{13}C NMR (125 MHz, $CDCl_3$) δ 150.59, 138.93, 132.95, 120.41, 117.55, 114.16, 52.59, 17.03, −0.90 ppm; HRMS (ESI) *m/z*: $[M + H]^+$ calcd for $C_{20}H_{25}Br_2N_2Si$, 479.0148; found 479.0150.

3,3'-(Dimethylsilanediyl)bis(4-bromo-*N*,*N*-dimethylaniline) (33b).

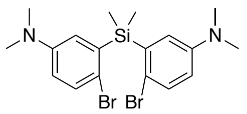

The same procedure was used as described above for compound **33a**. A solution of **28b** (1.50 g, 5.02 mmol) in a mixture of anhydrous ACN/DCM (2:1, 46.0 mL) was treated with NBS (1.81 g, 10.2 mmol) to provide **33b** (1.90 g, 83%) as a white solid. ^{1}H NMR (500 MHz, $CDCl_3$) δ 7.35 (d, J = 8.5 Hz, 2 H), 6.84 (d, J = 3.5 Hz, 2 H), 6.60 (dd, J = 8.5, 3.0 Hz, 2 H), 2.88 (s, 12 H), 0.76 (s, 6 H) ppm; ^{13}C NMR (125 MHz, $CDCl_3$) δ 149.04, 138.88, 133.11, 121.93, 116.94, 115.40, 40.71, −0.79 ppm; HRMS (ESI) *m/z*: $[M + H]^+$ calcd for $C_{18}H_{25}Br_2N_2Si$, 455.0148; found 455.0151.

Bis(5-(azetidin-1-yl)-2-bromophenyl)divinylsilane (34).

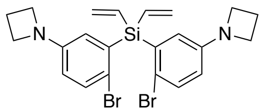

The same procedure was used as described above for compound **33a**. A solution of **29** (0.67 g, 1.93 mmol) in a mixture of anhydrous ACN/DCM (2:1, 24.0 mL) was treated with NBS (0.71 g, 3.96 mmol) to provide **34** (0.75 g, 77%) as a white solid. ^{1}H NMR (500 MHz, $CDCl_3$) δ 7.31 (d, J = 8.5 Hz, 2 H), 6.66 (dd, J = 20.0, 14.5 Hz, 2 H), 6.61 (d, J = 3.0 Hz, 2 H), 6.33 (dd, J = 8.5, 3.0 Hz, 2 H), 6.26 (dd, J = 14.5, 3.5 Hz, 2 H), 5.84 (dd, J = 20.5, 3.5 Hz, 2 H), 3.82 (t, J = 7.0 Hz, 8 H), 2.33 (p, J = 7.5 Hz, 4 H) ppm; ^{13}C NMR (125 MHz,

$CDCl_3$) δ 150.59, 136.40, 136.25, 133.51, 132.97, 121.62, 117.58, 114.44, 52.57, 17.02 ppm; HRMS (ESI) *m/z*: $[M + H]^+$ calcd for $C_{22}H_{25}Br_2N_2Si$, 503.0148; found 503.0151.

Bis(5-(azetidin-1-yl)-2-bromophenyl)diphenylsilane (35).

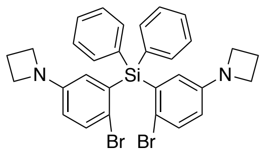

The same procedure was used as described above for compound **33a**. A solution of **30** (0.78 g, 1.75 mmol) in a mixture of anhydrous ACN/DCM (2:1, 30.0 mL) was treated with NBS (0.64 g, 3.58 mmol) to provide **35** (0.80 g, 76%) as a white solid. ^{1}H NMR (500 MHz, $CDCl_3$) δ 7.67–7.61 (m, 4 H), 7.42–7.31 (m, 8 H), 6.53 (d, J = 3.0 Hz, 2 H), 6.36 (dd, J = 8.5, 3.0 Hz, 2 H), 3.71 (t, J = 7.5 Hz, 8 H), 2.27 (p, J = 7.0 Hz, 4 H) ppm; ^{13}C NMR (125 MHz, $CDCl_3$) δ 150.47, 136.86, 135.81, 133.83, 133.41, 129.40, 127.65, 123.34, 118.34, 114.50, 52.35, 16.96 ppm; HRMS (ESI) *m/z*: $[M + H]^+$ calcd for $C_{30}H_{29}Br_2N_2Si$, 603.0461; found 603.0460.

1,1'-(((3-Chloropropyl)(methyl)silanediyl)bis(4-bromo-3,1-phenylene))bis(azetidine) (36a).

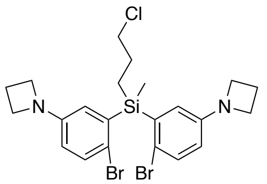

The same procedure was used as described above for compound **33a**. A solution of **31a** (1.0 g, 2.60 mmol) in a mixture of anhydrous ACN/DCM (2:1, 30.0 mL) was treated with NBS (0.94 g, 5.25 mmol) to provide **36a** (1.16 g, 82%) as a white gummy solid. ^{1}H NMR (500 MHz, $CDCl_3$) δ 7.31 (d, J = 8.5 Hz, 2 H), 6.49 (d, J = 2.5 Hz, 2 H), 6.33 (dd, J = 8.5, 2.0 Hz, 2 H), 3.82 (t, J = 7.5 Hz, 8 H), 3.54 (t, J = 7.0 Hz, 2 H), 2.34 (p, J = 7.5 Hz, 4 H), 1.83–1.74 (m, 2 H), 1.46–1.40 (m, 2 H), 0.71 (s, 3 H) ppm; ^{13}C NMR (125 MHz, $CDCl_3$)

δ 150.53, 137.58, 133.04, 120.62, 117.48, 114.32, 52.60, 48.26, 27.98, 17.01, 12.05, −2.64 ppm; HRMS (ESI) *m/z*: $[M + H]^+$ calcd for $C_{22}H_{28}Br_2ClN_2Si$, 541.0072; found 541.0076.

3,3'-((3-Chloropropyl)(methyl)silanediyl)bis(4-bromo-*N*,*N*-dimethylaniline) (36b).

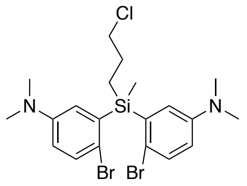

The same procedure was used as described above for compound **33a**. A solution of **31b** (3.60 g, 9.97 mmol) in a mixture of anhydrous ACN/DCM (2:1, 60.0 mL) was treated with NBS (3.58 g, 20.2 mmol) to provide **36b** (4.50 g, 87%) as a white gummy solid. ^{1}H NMR (500 MHz, $CDCl_3$) δ 7.35 (d, J = 9.0 Hz, 2 H), 6.80 (d, J = 2.5 Hz, 2 H), 6.60 (dd, J = 7.0, 2.0 Hz, 2 H), 3.55 (t, J = 6.5 Hz, 2 H), 2.88 (s, 12 H), 1.86–1.78 (m, 2 H), 1.51–1.45 (m, 2 H), 0.75 (s, 3 H) ppm; ^{13}C NMR (125 MHz, $CDCl_3$) δ 149.05, 137.53, 133.21, 122.04, 116.78, 115.48, 48.28, 40.70, 28.09, 12.26, −2.54 ppm; HRMS (ESI) *m/z*: $[M + H]^+$ calcd for $C_{20}H_{28}Br_2ClN_2Si$, 519.0051; found 519.0048.

3,3'-(Silolane-1,1-diyl)bis(4-bromo-*N*,*N*-dimethylaniline) (37).

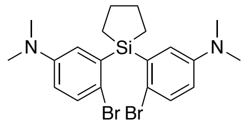

The same procedure was used as described above for compound **33a**. A solution of **32** (0.50 g, 1.54 mmol) in a mixture of anhydrous ACN/DCM (2:1, 21.0 mL) was treated with NBS (0.56 g, 3.11 mmol) to provide **37** (0.60 g, 81%) as a white solid. ^{1}H NMR (500 MHz, $CDCl_3$) δ 7.32 (d, J = 8.5 Hz, 2 H), 7.02 (d, J = 3.0 Hz, 2 H), 6.59 (dd, J = 8.5, 3.0 Hz, 2 H), 2.89 (s, 12 H), 1.80 (p, J = 3.5 Hz, 4 H), 1.34 (t, J = 7.0 Hz, 4 H) ppm; ^{13}C NMR (125 MHz, $CDCl_3$) δ 148.95, 137.76, 132.84, 122.33, 117.46, 115.54, 40.78, 27.21, 12.82 ppm; HRMS (ESI) *m/z*: $[M + H]^+$ calcd for $C_{20}H_{27}Br_2N_2Si$, 481.0305; found 481.0308.

1-(7-(Azetidin-1-yl)-5,5-dimethyl-10-(*o*-tolyl)dibenzo[*b*,*e*]silin-3(5*H*)-ylidene)azetidin-1-ium (38).

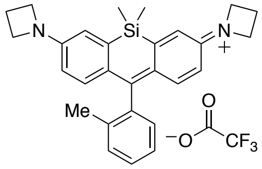

A degassed solution of **33a** (0.30 g, 0.63 mmol) in anhydrous THF (15.0 mL) under argon atmosphere was cooled to −78 °C in an acetone/dry ice bath. After 15 min, *s*-BuLi (1.4M in cyclohexane) (1.78 mL, 2.50 mmol) was added dropwise over 5 min. The resulting reaction mixture was stirred at −78 °C for additional 1 hour. It was then warmed to −20 °C, and a solution of methyl 2-methylbenzoate (0.19 mL, 1.37 mmol) in THF (10.0 mL) was added dropwise over 30 min. The reaction was allowed to warm to room temperature and stirred for overnight (18 h). It was subsequently quenched with saturated NH_4Cl (25.0 mL), diluted with water (25.0 mL), and then extracted with EtOAc (2 x 50.0 mL), the combined extracts were washed with saturated NaCl solution (25.0 mL), dried (Na_2SO_4), filtered, and evaporated under reduced pressure. The resulting residue was re-dissolved in anhydrous MeOH (15.0 mL), and treated with AcOH (150 μL) at room temperature (immediate dark blue color), and then the mixture solution was stirred for 10 min. The solvent was then evaporated under reduced pressure, and the residue was purified by flash column chromatography (Silicycle column, 12 g, 0–5% MeOH in 1% v/v TFA/DCM for 10 min, hold at 5% MeOH isocratic for 5 min, and then increase to 15% gradient over 5 min) to yield (0.11 g, 41%) of the trifluoroacetate salt of **38** as a dark blue solid. ^{1}H NMR (500 MHz, $CDCl_3$) δ 7.43–7.39 (m, 1 H), 7.35–7.29 (m, 2 H), 7.06 (d, J = 7.5 Hz, 1 H), 7.00 (d, J = 9.5 Hz, 2 H), 6.75 (d, J = 2.5 Hz, 2 H), 6.20 (dd, J = 9.5, 2.5 Hz, 2 H), 4.34 (t, J = 7.5 Hz, 8 H), 2,59 (p, J = 7.5 Hz, 4 H), 2.00 (s, 3 H), 0.55 (s, 3 H), 0.53 (s, 3 H) ppm; ^{13}C NMR (125 MHz, $CDCl_3$) δ 169.89, 153.03, 148.28, 141.39, 138.75, 135.78, 130.39, 128.98, 127.59, 125.78, 118.82, 117.09, 114.79, 111.90, 51.99, 19.40, 16.09, −1.01, −1.32 ppm; ^{19}F NMR (470 MHz, $CDCl_3$) δ −75.77 ppm; HRMS (ESI) *m/z*: $[M]^+$ calcd for $C_{28}H_{31}N_2Si$, 423.2251; found 423.2249.

1-(7-(Azetidin-1-yl)-10-(*o*-tolyl)-5,5-divinyldibenzo[*b*,*e*]silin-3(5*H*)-ylidene)azetidin-1-ium (39).

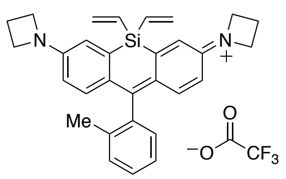

The same procedure was used as described above for compound **38**. A solution of **34** (0.10 g, 0.20 mmol) in anhydrous THF (5.0 mL) was treated with *s*-BuLi (1.4M in cyclohexane) (0.36 mL, 0.50 mmol) and methyl 2-methylbenzoate (61.0 μL, 0.44 mmol) in THF (5.0 mL). The resulting residue was re-dissolved in MeOH (5.0 mL), followed by treatment with AcOH (50.0 μL) to provide **39** (15.0 mg, 17%) as a dark blue solid. ^{1}H NMR (500 MHz, $CDCl_3$) δ 7.44–7.39 (m, 1 H), 7.35–7.29 (m, 2 H), 7.06 (d, J = 7.5 Hz, 1 H), 7.01 (d, J = 9.5 Hz, 2 H), 6.73–6.65 (m, 2 H), 6.43–6.25 (m, 4 H), 6.23 (dd, J = 9.5, 2.0 Hz, 2 H), 5.99 (dd, J = 20.0, 3.0 Hz, 1 H), 5.91 (dd, J = 19.0, 4.0 Hz, 1 H), 4.34 (t, J = 7.5 Hz, 8 H), 2.59 (p, J = 7.5 Hz, 4 H), 1.99 (s, 3 H) ppm; ^{13}C NMR (125 MHz, $CDCl_3$) δ 169.63, 152.94, 143.84, 141.55, 139.52, 139.13, 138.51, 135.83, 130.93, 130.56, 130.44, 129.05, 127.95, 127.31, 125.80, 120.28, 112.20, 52.09, 19.41, 16.10 ppm; ^{19}F NMR (470 MHz, $CDCl_3$) δ −75.62 ppm; HRMS (ESI) *m/z*: $[M]^+$ calcd for $C_{30}H_{31}N_2Si$, 447.2251; found 447.2250.

1-(7-(Azetidin-1-yl)-5,5-diphenyl-10-(*o*-tolyl)dibenzo[*b*,*e*]silin-3(5*H*)-ylidene)azetidin-1-ium (40).

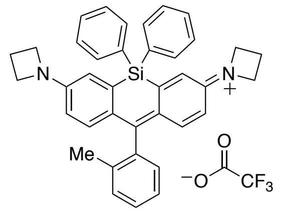

The same procedure was used as described above for compound **38**. A solution of **35** (0.15 g, 0.25 mmol) in anhydrous THF (10.0 mL) was treated with *s*-BuLi (1.4M in cyclohexane) (0.71 mL, 0.99 mmol) and methyl 2-methylbenzoate (76.0 μL, 0.55 mmol) in THF (10.0 mL). The resulting residue was re-dissolved in MeOH (10.0 mL), followed by treatment

with AcOH (100 µL) to provide **40** (50.0 mg, 37%) as a dark blue solid. ^{1}H NMR (500 MHz, $CDCl_3$) δ 7.68–7.39 (m, 11 H), 7.37–7.30 (m, 2 H), 7.13–7.01 (m, 3 H), 6.75–6.62 (m, 2 H), 6.26 (d, J = 8.5 Hz, 2 H), 4.26 (t, J = 7.5 Hz, 8 H), 2.54 (p, J = 7.5 Hz, 4 H), 2.00 (s, 3 H) ppm; ^{13}C NMR (125 MHz, $CDCl_3$) δ 169.83, 152.91, 144.69, 141.67, 138.40, 136.01, 135.85, 131.36, 131.32, 131.25, 130.81, 130.51, 129.15, 129.04, 128.96, 128.94, 128.21, 125.85, 120.70, 112.41, 52.10, 19.41, 16.04 ppm; ^{19}F NMR (470 MHz, $CDCl_3$) δ −75.79 ppm; HRMS (ESI) *m/z*: $[M]^+$ calcd for $C_{38}H_{35}N_2Si$, 547.2564; found 547.2558.

1-(7-(Azetidin-1-yl)-5-(3-chloropropyl)-5-methyl-10-(*o*-tolyl)dibenzo[*b*,*e*]silin-3(5*H*)-ylidene)azetidin-1-ium (41).

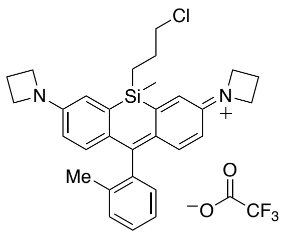

The same procedure was used as described above for compound **38**. A solution of **36a** (0.20 g, 0.37 mmol) in anhydrous THF (10.0 mL) was treated with *s*-BuLi (1.4M in cyclohexane) (0.92 mL, 1.29 mmol) and methyl 2-methylbenzoate (0.12 mL, 0.81 mmol) in THF (10.0 mL). The resulting residue was re-dissolved in MeOH (10.0 mL), followed by treatment with AcOH (100 µL) to provide **41** as an inseparable mixture of isomers (65.0 mg, 36%) in dark blue color solid. ^{1}H NMR (500 MHz, $CDCl_3$) δ 7.44–7.39 (m, 1 H), 7.36–7.29 (m, 2 H), 7.04 (d, J = 7.5 Hz, 1 H), 7.02–6.97 (m, 2 H), 6.85–6.80 (m, 2 H), 6.21 (dd, J = 9.5, 2.0 Hz, 2 H), 4.38 (t, J = 7.5 Hz, 8 H), 3.47 and 3.43 (2 x t, J = 6.5 Hz, 2 H), 2.59 (p, J = 7.5 Hz, 4 H), 2.01 and 2.00 (2 x s, 3 H), 1.76–1.61 (m, 2 H), 1.20–1.13 (m, 2 H), 0.64 and 0.62 (2 x s, 3 H) ppm; ^{13}C NMR (125 MHz, $CDCl_3$) δ 169.92 (2 signals), 152.96 (2 signals), 146.57 (2 signals), 141.43 (2 signals), 138.67 (2 signals), 135.89, 135.59, 130.43 (2 signals), 129.16, 129.04, 128.82, 127.93, 127.82, 125.84 (2 signals), 119.00 (2 signals), 112.06, 52.07, 47.34 (2 signals), 26.93 (2 signals), 19.42, 16.10, 13.74 (2 signals), −3.57 (2 signals) ppm; ^{19}F NMR (470 MHz, $CDCl_3$) δ −75.79 ppm; HRMS (ESI) *m/z*: $[M]^+$ calcd for $C_{30}H_{34}ClN_2Si$, 485.2174; found 485.2173.

_N_-(7-(Dimethylamino)-10-(_o_-tolyl)-3_H_-spiro[dibenzo[_b_,_e_]siline-5,1'-silolan]-3-ylidene)-_N_-methylmethanaminium (42).

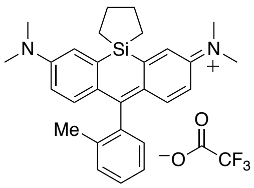

The same procedure was used as described above for compound **38**. A solution of **37** (0.30 g, 0.62 mmol) in anhydrous THF (15.0 mL) was treated with *s*-BuLi (1.4M in cyclohexane) (1.95 mL, 2.74 mmol) and methyl 2-methylbenzoate (0.20 mL, 1.37 mmol) in THF (10.0 mL). The resulting residue was re-dissolved in MeOH (15.0 mL), followed by treatment with AcOH (150 µL) to provide **42** (0.11 g, 41%) as a dark blue solid. ^{1}H NMR (500 MHz, $CDCl_3$) δ 7.46–7.41 (m, 1 H), 7.37–7.31 (m, 2 H), 7.12–7.05 (m, 5 H), 6.61 (dd, J = 10.0, 2.5 Hz, 2 H), 3.32 (s, 12 H), 2.06–2.00 (m, 4 H), 2.03 (s, 3 H, overlapping), 1.23–1.14 (m, 4 H) ppm; ^{13}C NMR (125 MHz, $CDCl_3$) δ 170.82, 154.16, 148.07, 141.92, 138.40, 135.84, 130.43, 129.17, 128.92, 128.34, 125.82, 120.73, 116.88, 114.59, 114.25, 40.91, 28.81, 28.74, 19.55, 16.11, 15.75 ppm; ^{19}F NMR (470 MHz, $CDCl_3$) δ −75.87 ppm; HRMS (ESI) *m/z*: $[M]^+$ calcd for $C_{28}H_{33}N_2Si$, 425.2408; found 425.2404.

_N_-(5-(3-Chloropropyl)-10-(2,6-dimethoxyphenyl)-7-(dimethylamino)-5-methyldibenzo[_b_,_e_]silin-3(5_H_)-ylidene)-_N_-methylmethanaminium (43).

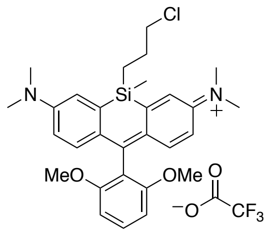

The same procedure was used as described above for compound **38**. A solution of **36b** (1.10 g, 2.12 mmol) in anhydrous THF (40.0 mL) was treated with *s*-BuLi (1.4M in cyclohexane) (6.06 mL, 8.48 mmol) and methyl 2,6-dimethoxybenzoate (0.92 g, 4.66 mmol) in THF (10.0 mL). The resulting residue was re-dissolved in MeOH (50.0 mL), followed by treatment with AcOH (1.0 mL) to provide **43** (0.80 g, 74%) as a dark blue

solid. ^{1}H NMR (500 MHz, $CDCl_3$) δ 7.48 (t, J = 8.5 Hz, 1 H), 7.25 (d, J = 9.5 Hz, 2 H), 7.10 (d, J = 3.0 Hz, 2 H), 6.71 (t, J = 8.5 Hz, 2 H), 6.62 (dd, J = 9.5, 3.0 Hz, 2 H), 3.67 (s, 3 H), 3.65 (s, 3 H), 3.39 (t, J = 6.5 Hz, 2 H), 3.32 (s, 12 H), 1.71–1.62 (m, 2 H), 1.15–1.08 (m, 2 H), 0.66 (s, 3 H) ppm; ^{13}C NMR (125 MHz, $CDCl_3$) δ 167.91, 157.48, 157.40, 154.30, 146.76, 141.40, 131.26, 128.96, 120.24, 116.89, 115.74, 114.58, 114.14, 104.08, 103.89, 56.19, 56.17, 47.23, 40.84, 26.82, 14.42, −3.95 ppm; ^{19}F NMR (470 MHz, $CDCl_3$) δ −75.82 ppm; HRMS (ESI) *m/z*: $[M]^+$ calcd for $C_{29}H_{36}ClN_2O_2Si$, 507.2229; found 507.2221.

1-(7-(Azetidin-1-yl)-5-(3-chloropropyl)-10-(2,6-dimethoxyphenyl)-5-methyldibenzo[*b*,*e*]silin-3(5*H*)-ylidene)azetidin-1-ium (44).

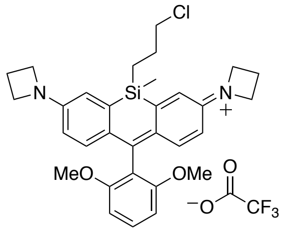

The same procedure was used as described above for compound **38**. A solution of **36a** (1.30 g, 2.39 mmol) in anhydrous THF (40.0 mL) was treated with *s*-BuLi (1.4M in cyclohexane) (6.84 mL, 9.56 mmol) and methyl 2,6-dimethoxybenzoate (1.04 g, 5.27 mmol) in THF (10.0 mL). The resulting residue was re-dissolved in MeOH (50.0 mL), followed by treatment with AcOH (1.0 mL) to provide **44** (1.0 g, 78%) as a dark blue solid. ^{1}H NMR (500 MHz, $CDCl_3$) δ 7.46 (t, J = 8.5 Hz, 1 H), 7.16 (d, J = 9.5 Hz, 2 H), 6.71 (d, J = 3.0 Hz, 2 H), 6.69 (t, J = 8.5 Hz, 2 H), 6.23 (dd, J = 9.0, 2.5 Hz, 2 H), 4.33 (t, J = 7.5 Hz, 8 H), 3.66 (s, 3 H), 3.64 (s, 3 H), 3.38 (t, J = 7.0 Hz, 2 H), 2.58 (p, J = 8.0 Hz, 4 H), 1.69–1.58 (m, 2 H), 1.11–1.03 (m, 2 H), 0.62 (s, 3 H) ppm; ^{13}C NMR (125 MHz, $CDCl_3$) δ 166.82, 157.47, 157.38, 153.11, 146.33, 140.91, 131.10, 128.82, 118.44, 116.07, 112.07, 104.09, 103.86, 56.19, 56.16, 51.99, 47.30, 26.85, 16.15, 14.45, −4.07 ppm; ^{19}F NMR (470 MHz, $CDCl_3$) δ −75.78 ppm; HRMS (ESI) *m/z*: $[M]^+$ calcd for $C_{31}H_{36}ClN_2O_2Si$, 531.2229; found 531.2226.

Synthesis of Si-Bridge spirolactones

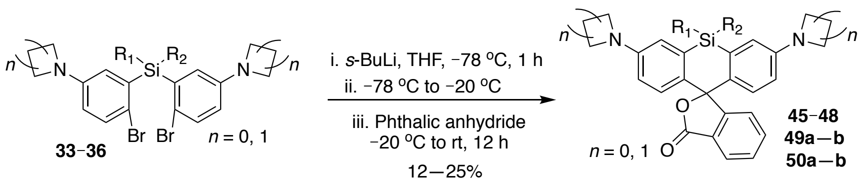

3,7-Bis(dimethylamino)-5,5-dimethyl-3'*H*,5*H*-spiro[dibenzo[*b*,*e*]siline-10,1'-isobenzofuran]-3'-one (45).

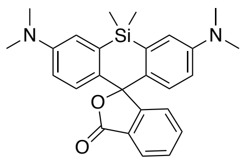

A degassed solution of **33b** (0.40 g, 0.88 mmol) in anhydrous THF (20.0 mL) under argon atmosphere was cooled to −78 °C in an acetone/dry ice bath. After 15 min, *s*-BuLi (1.4M in cyclohexane) (2.50 mL, 3.51 mmol) was added dropwise over 5 min. The resulting reaction mixture was stirred at −78 °C for additional 1 hour. It was then warmed to −20 °C, and a solution of phthalic anhydride (0.29 g, 1.93 mmol) in THF (10.0 mL) was added dropwise over 30 min. The reaction was allowed to warm to room temperature and stirred for overnight (18 h). It was subsequently quenched with saturated NH_4Cl (25.0 mL), diluted with water (25.0 mL), and then extracted with EtOAc (2 x 50.0 mL), the combined extracts were washed with saturated $NaHCO_3$ solution (25.0 mL), saturated NaCl solution (25.0 mL), dried (Na_2SO_4), filtered, and evaporated under reduced pressure. The resulting residue was purified by flash column chromatography (Silicycle column, 12 g, 0–25% EtOAc in 20% v/v DCM Hexanes, linear gradient for 20 min) to provide **45** (90.0 mg, 24%) as a light green solid. ^1H NMR (500 MHz, $CDCl_3$) δ 7.97 (d, J = 7.5 Hz, 1 H), 7.64 (td, J = 7.5, 1.5 Hz, 1 H), 7.54 (td, J = 7.5, 1.0 Hz, 1 H), 7.30 (d, J = 8.0 Hz, 1 H), 6.97 (d, J = 2.5 Hz, 2 H), 6.79 (d, J = 8.5 Hz, 2 H), 6.55 (dd, J = 9.0, 2.5 Hz, 2 H), 2.96 (s, 12 H), 0.65 (s, 3 H), 0.61 (s, 3 H) ppm; ^{13}C NMR (125 MHz, $CDCl_3$) δ 170.82, 154.55, 149.41, 137.10, 133.77, 132.06, 128.79, 128.29, 127.16, 125.76, 124.69, 116.72, 113.44, 91.97, 40.41, 0.56, −1.36 ppm; HRMS (ESI) *m/z*: $[M + H]^+$ calcd for $C_{26}H_{29}N_2O_2Si$, 429.1993; found 429.1989.

3,7-Di(azetidin-1-yl)-5,5-dimethyl-3'*H*,5*H*-spiro[dibenzo[*b*,*e*]siline-10,1'-isobenzofuran]-3'-one (46).

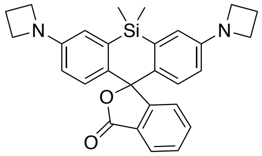

The same procedure was used as described above for compound **45**. A solution of **33a** (0.26 g, 0.54 mmol) in anhydrous THF (15.0 mL) was treated with *s*-BuLi (1.4M in cyclohexane) (1.55 mL, 2.17 mmol) and phthalic anhydride (0.18 g, 1.19 mmol) in THF (10.0 mL) to provide **46** (60.0 mg, 25%) as a light green solid. ^{1}H NMR (500 MHz, $CDCl_3$) δ 7.96 (d, J = 7.5 Hz, 1 H), 7.64 (td, J = 7.5, 1.0 Hz, 1 H), 7.54 (td, J = 7.5, 0.5 Hz, 1 H), 7.31 (d, J = 7.5 Hz, 1 H), 6.76 (d, J = 8.5 Hz, 2 H), 6.67 (d, J = 2.5 Hz, 2 H), 6.25 (dd, J = 8.5, 2.5 Hz, 2 H), 3.89 (t, J = 7.5 Hz, 8 H), 2,36 (p, J = 7.5 Hz, 4 H), 0.61 (s, 3 H), 0.59 (s, 3 H) ppm; ^{13}C NMR (125 MHz, $CDCl_3$) δ 170.72, 154.31, 151.00, 137.08, 133.71, 132.96, 128.84, 128.02, 127.19, 125.83, 124.83, 115.73, 112.28, 92.09, 52.40, 17.04, 0.53, −1.49 ppm; HRMS (ESI) *m/z*: $[M + H]^+$ calcd for $C_{28}H_{29}N_2O_2Si$, 453.1993; found 453.1988.

3,7-Di(azetidin-1-yl)-5,5-divinyl-3'*H*,5*H*-spiro[dibenzo[*b*,*e*]siline-10,1'-isobenzofuran]-3'-one (47).

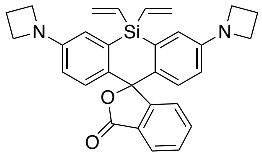

The same procedure was used as described above for compound **45**. A solution of **34** (0.25 g, 0.50 mmol) in anhydrous THF (15.0 mL) was treated with *s*-BuLi (1.4M in cyclohexane) (1.06 mL, 1.49 mmol) and phthalic anhydride (0.17 g, 1.09 mmol) in THF (10.0 mL) to provide **47** (30.0 mg, 12%) as a light green solid. ^{1}H NMR (500 MHz, $CDCl_3$) δ 7.91 (d, J = 7.5 Hz, 1 H), 7.48 (td, J = 7.5, 1.0 Hz, 1 H), 7.43 (td, J = 7.5, 1.0 Hz, 1 H), 7.20 (d, J = 8.0 Hz, 1 H), 6.94 (d, J = 8.5 Hz, 2 H), 6.63 (d, J = 3.0 Hz, 2 H), 6.51 (dd, J = 20.0, 14.5 Hz, 2 H), 6.42–6.26 (m, 4 H), 6.03 (dd, J = 19.5, 4.0 Hz, 1 H), 6.01 (dd, J = 20.0, 4.0 Hz, 1 H), 3.87 (t, J = 7.5 Hz, 8 H), 2,35 (p, J = 7.5 Hz, 4 H) ppm; ^{13}C NMR (125 MHz, $CDCl_3$)

δ 171.55, 156.52, 150.80, 137.04, 134.72, 134.31, 134.17, 133.51, 131.18, 128.49, 127.84, 125.71, 125.12, 123.90, 116.87, 113.34, 90.89, 52.41, 17.03 ppm; HRMS (ESI) *m/z*: [M + H]$^+$ calcd for $C_{30}H_{29}N_2O_2Si$, 477.1993; found 477.1991.

3,7-Di(azetidin-1-yl)-5,5-diphenyl-3'*H*,5*H*-spiro[dibenzo[*b*,*e*]siline-10,1'-isobenzofuran]-3'-one (48).

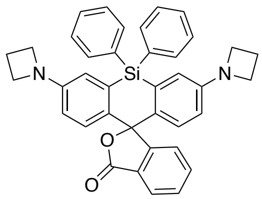

The same procedure was used as described above for compound **45**. A solution of **35** (0.20 g, 0.33 mmol) in anhydrous THF (10.0 mL) was treated with *s*-BuLi (1.4M in cyclohexane) (0.95 mL, 1.32 mmol) and phthalic anhydride (0.11 g, 0.73 mmol) in THF (10.0 mL) to provide **48** (30.0 mg, 16%) as a light green solid. ^{1}H NMR (500 MHz, $CDCl_3$) δ 7.87 (d, *J* = 7.5 Hz, 1 H), 7.77–7.72 (m, 2 H), 7.59–7.54 (m, 2 H), 7.50–7.45 (m, 1 H), 7.44–7.39 (m, 3 H), 7.37–7.32 (m, 2 H), 7.31 (td, *J* = 7.5, 0.5 Hz, 1 H), 7.17 (td, *J* = 8.0, 1.0 Hz, 1 H), 7.08 (d, *J* = 8.5 Hz, 2 H), 6.63 (d, *J* = 2.5 Hz, 2 H), 6.61 (d, *J* = 8.0 Hz, 1 H), 6.41 (dd, *J* = 8.5, 2.5 Hz, 2 H), 3.86–3.75 (m, 8 H), 2,30 (p, *J* = 7.0 Hz, 4 H) ppm; ^{13}C NMR (125 MHz, $CDCl_3$) δ 171.89, 157.44, 150.75, 136.54, 136.14, 135.07, 134.56, 134.37, 134.04, 130.74, 129.99, 129.96, 128.28, 128.24, 127.98, 127.67, 125.57, 124.17, 123.26, 117.42, 113.74, 90.43, 52.39, 16.96 ppm; HRMS (ESI) *m/z*: [M + H]$^+$ calcd for $C_{38}H_{33}N_2O_2Si$, 577.2306; found 577.2302.

5-(3-Chloropropyl)-3,7-bis(dimethylamino)-5-methyl-3'*H*,5*H*-spiro[dibenzo[*b*,*e*]siline-10,1'-isobenzofuran]-3'-one (49).

The same procedure was used as described above for compound **45**. A solution of **36b** (1.90 g, 3.66 mmol) in anhydrous THF (50.0 mL) was treated with *s*-BuLi (1.4M in cyclohexane) (10.5 mL, 14.6 mmol) and phthalic anhydride (1.19 g, 8.05 mmol) in THF (10.0 mL) to provide **49** as a separable mixture of isomers (0.45 g, 25%).

(5*s*,10*s*)-5-(3-Chloropropyl)-3,7-bis(dimethylamino)-5-methyl-3'*H*,5*H*-spiro[dibenzo[*b*,*e*]siline-10,1'-isobenzofuran]-3'-one (49a).

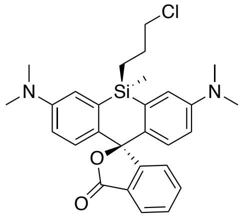

49a was obtained as a light green color solid. ^{1}H NMR (500 MHz, $CDCl_3$) δ 7.97 (dt, J = 7.5, 0.5 Hz, 1 H), 7.66 (td, J = 7.5, 1.5 Hz, 1 H), 7.56 (td, J = 7.5, 0.5 Hz, 1 H), 7.31 (d, J = 7.5 Hz, 1 H), 6.94 (d, J = 3.0 Hz, 2 H), 6.73 (d, J = 9.0 Hz, 2 H), 6.55 (dd, J = 9.0, 2.5 Hz, 2 H), 3.48 (t, J = 6.5 Hz, 2 H), 2,97 (s, 12 H), 1.91–1.83 (m, 2 H), 1.24–1.18 (m, 2 H), 0.65 (s, 3 H) ppm; ^{13}C NMR (125 MHz, $CDCl_3$) δ 170.62, 154.27, 149.40, 135.76, 133.80, 132.20, 128.92, 128.68, 127.56, 125.72, 124.85, 116.77, 113.52, 92.09, 48.09, 40.39, 27.75, 14.21, −3.50 ppm; HRMS (ESI) *m/z*: $[M + H]^+$ calcd for $C_{28}H_{32}ClN_2O_2Si$, 491.1916; found 491.1911.

(5*r*,10*r*)-5-(3-Chloropropyl)-3,7-bis(dimethylamino)-5-methyl-3'*H*,5*H*-spiro[dibenzo[*b*,*e*]siline-10,1'-isobenzofuran]-3'-one (49b).

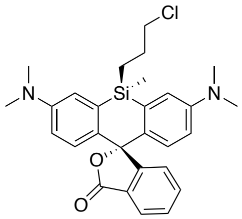

49b was obtained as a light green color solid. ^{1}H NMR (500 MHz, $CDCl_3$) δ 7.97 (dt, J = 8.0, 1.0 Hz, 1 H), 7.63 (td, J = 7.5, 1.5 Hz, 1 H), 7.54 (td, J = 7.5, 1.0 Hz, 1 H), 7.24 (d, J = 8.0 Hz, 1 H), 6.93 (d, J = 2.5 Hz, 2 H), 6.77 (d, J = 9.0 Hz, 2 H), 6.57 (dd, J = 9.0, 3.0 Hz, 2 H), 3.55 (t, J = 6.5 Hz, 2 H), 2,97 (s, 12 H), 1.94–1.86 (m, 2 H), 1.33–1.26 (m, 2 H), 0.60 (s, 3 H) ppm; ^{13}C NMR (125 MHz, $CDCl_3$) δ 170.86, 154.87, 149.36, 134.87, 133.98, 132.18, 128.51, 128.13, 127.10, 125.70, 124.44, 116.39, 113.78, 91.80, 48.22, 40.40, 27.86, 12.62, −1.18 ppm; HRMS (ESI) *m/z*: $[M + H]^+$ calcd for $C_{28}H_{32}ClN_2O_2Si$, 491.1916; found 491.1911.

3,7-Di(azetidin-1-yl)-5-(3-chloropropyl)-5-methyl-3'*H*,5*H*-spiro[dibenzo[*b*,*e*]siline-10,1'-isobenzofuran]-3'-one (50).

The same procedure was used as described above for compound **45**. A solution of **36a** (0.20 g, 0.37 mmol) in anhydrous THF (10.0 mL) was treated with *s*-BuLi (1.4M in cyclohexane) (0.80 mL, 1.10 mmol) and phthalic anhydride (0.12 g, 0.81 mmol) in THF (10.0 mL) to provide **50** as a separable mixture of isomers (40.0 mg, 21%).

(5*s*,10*s*)-3,7-Di(azetidin-1-yl)-5-(3-chloropropyl)-5-methyl-3'*H*,5*H*-spiro[dibenzo[*b*,*e*]siline-10,1'-isobenzofuran]-3'-one (50a).

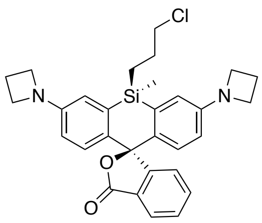

50a was obtained as a light green color solid. ^{1}H NMR (500 MHz, $CDCl_3$) δ 7.96 (d, J = 7.5 Hz, 1 H), 7.66 (t, J = 7.5 Hz, 1 H), 7.56 (t, J = 7.5 Hz, 1 H), 7.31 (d, J = 8.0 Hz, 1 H), 6.70 (d, J = 8.5 Hz, 2 H), 6.65 (d, J = 2.5 Hz, 2 H), 6.26 (dd, J = 8.5, 2.5 Hz, 2 H), 3.90 (t, J = 7.0 Hz, 8 H), 3.48 (t, J = 7.0 Hz, 2 H), 2,37 (p, J = 7.5 Hz, 4 H), 1.89–1.80 (m, 2 H), 1.23–1.16 (m, 2 H), 0.62 (s, 3 H) ppm; ^{13}C NMR (125 MHz, $CDCl_3$) δ 170.50, 153.99, 150.95, 135.76, 133.75, 133.15, 128.98, 128.41, 127.57, 125.80, 124.98, 115.86, 112.45, 92.18, 52.42, 48.06, 27.74, 17.03, 14.17, −3.64 ppm; HRMS (ESI) *m/z*: $[M + H]^+$ calcd for $C_{30}H_{32}ClN_2O_2Si$, 515.1916; found 515.1908.

(5*r*,10*r*)-3,7-Di(azetidin-1-yl)-5-(3-chloropropyl)-5-methyl-3'*H*,5*H*-spiro[dibenzo[*b*,*e*]siline-10,1'-isobenzofuran]-3'-one (50b)

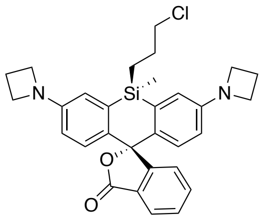

50b was obtained as an off-white color solid. ^{1}H NMR (500 MHz, $CDCl_3$) δ 7.96 (d, J = 7.5 Hz, 1 H), 7.63 (t, J = 7.5 Hz, 1 H), 7.54 (t, J = 7.5 Hz, 1 H), 7.25 (d, J = 7.5 Hz, 1 H), 6.74 (d, J = 9.0 Hz, 2 H), 6.64 (d, J = 2.5 Hz, 2 H), 6.28 (dd, J = 8.5, 2.5 Hz, 2 H), 3.90 (t, J = 7.5 Hz, 8 H), 3.54 (t, J = 6.5 Hz, 2 H), 2,37 (p, J = 7.0 Hz, 4 H), 1.91–1.81 (m, 2 H), 1.30–1.22 (m, 2 H), 0.58 (s, 3 H) ppm; ^{13}C NMR (125 MHz, $CDCl_3$) δ 170.77, 154.68, 150.92, 134.79, 133.95, 133.12, 128.86, 128.62, 127.08, 125.76, 124.53, 115.45, 112.71, 91.87, 52.40, 48.19, 27.78, 17.04, 12.50, −1.23 ppm; HRMS (ESI) *m/z*: $[M + H]^+$ calcd for $C_{30}H_{32}ClN_2O_2Si$, 515.1916; found 515.1907.

Unexpected tri-iodination of 44

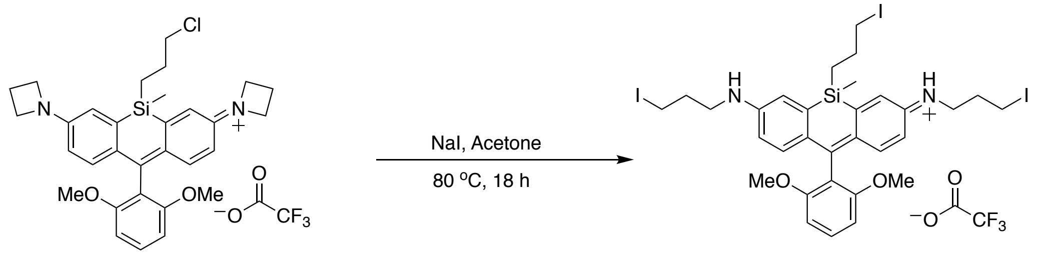

44 **Tri-iodination product**

A solution of **44** (0.10 g, 0.16 mmol) in anhydrous acetone (5.0 mL) under argon atmosphere was treated with NaI (93.0 mg, 0.62 mmol) at room temperature and reaction mixture was stirred at 80 °C for 18 h. After completion of the reaction, cooled to room temperature and solvent was evaporated under reduced pressure. The resulting residue was purified by flash column chromatography (Silicycle column, 25 g, 0–10% MeOH in 1% v/v TFA/DCM, linear gradient for 20 min) provided unexpected tri-iodinated product of **44** (0.13 g, 92%) in dark blue color solid. ^{1}H NMR (500 MHz, $CDCl_3$) δ 7.46 (t, J = 8.0 Hz, 1 H), 7.21 (d, J = 9.0 Hz, 2 H), 7.16 (br s, 2 H), 6.70 (d, J = 7.5 Hz, 1 H), 6.69 (d, J = 7.0 Hz, 1 H), 6.60 (d, J = 9.5 Hz, 2 H), 3.69 (s, 6 H), 3.55 (t, J = 6.5 Hz, 4 H), 3.28 (t, J = 6.5 Hz, 4 H), 3.08 (t, J = 7.0 Hz, 2 H), 2.20 (p, J = 6.5 Hz, 4 H), 1.77–1.69 (m, 2 H), 1.13–1.05 (m, 2 H), 0.61 (s, 3 H) ppm; ^{19}F NMR (470 MHz, $CDCl_3$) δ −75.66 ppm; HRMS (ESI) *m/z*: $[M]^+$ calcd for $C_{31}H_{39}I_3N_2O_2Si$, 878.9831; found 878.9822.

Introduction of handles

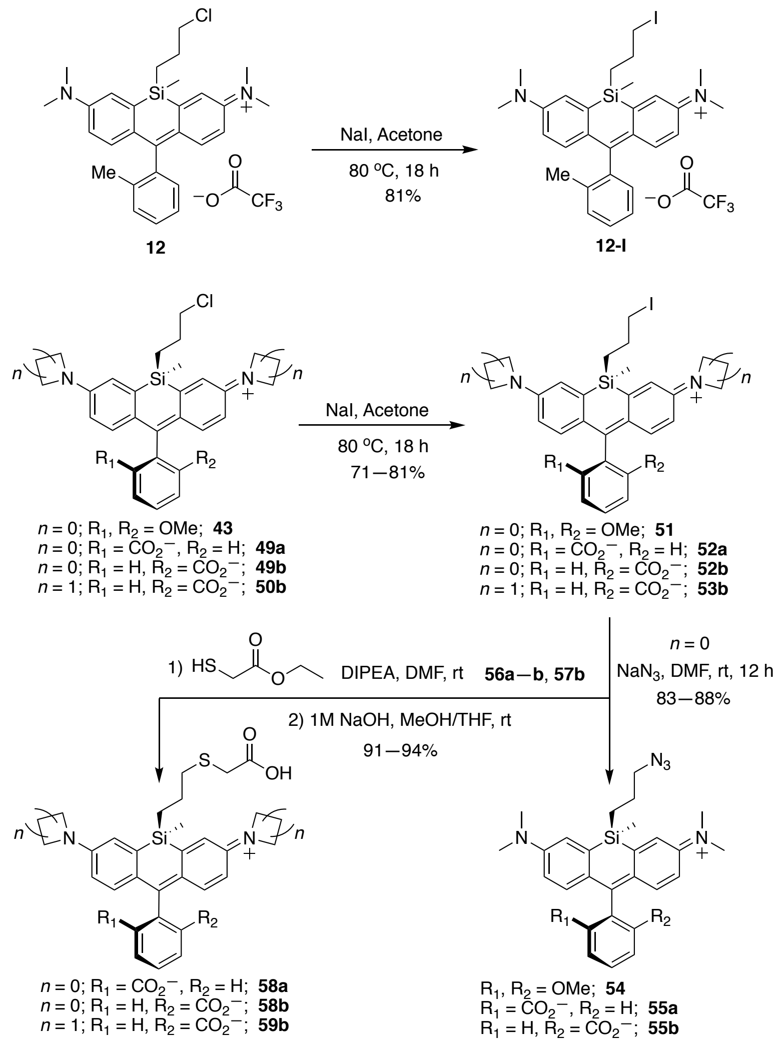

***N*-(7-(Dimethylamino)-5-(3-iodopropyl)-5-methyl-10-(*o*-tolyl)dibenzo[*b*,*e*]silin-3(5*H*)-ylidene)-*N*-methylmethanaminium (12-I)**.

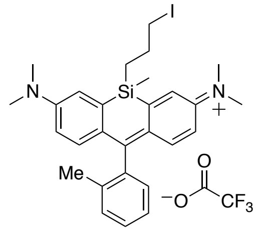

A solution of **12** (0.29 g, 0.63 mmol) in anhydrous acetone (10.0 mL) under argon atmosphere was treated with NaI (0.38 g, 2.51 mmol) at room temperature and reaction

mixture was stirred at 80 °C for 18 h. After completion of the reaction, cooled to room temperature and solvent was evaporated under reduced pressure. The resulting residue was purified by flash column chromatography (Silicycle column, 25 g, 0–10% MeOH in 1% v/v TFA/DCM, linear gradient for 20 min) to provide **12-I** as an inseparable mixture of isomers (0.28 g, 81%) in dark blue color solid. ^{1}H NMR (500 MHz, $CDCl_3$) δ 7.46–7.41 (m, 1 H), 7.37–7.31 (m, 2 H), 7.16 (t, J = 3.0 Hz, 2 H), 7.10 (d, J = 3.5 Hz, 1 H), 7.09–7.06 (m, 2 H), 6.61 (dd, J = 9.5, 2.5 Hz, 2 H), 3.34 (s, 12 H), 3.14 and 3.09 (2 x t, J = 7.0 Hz, 2 H), 2.04 and 2.01 (2 x s, 3 H), 1.80–1.66 (m, 2 H), 1.21–1.13 (m, 2 H), 0.66 and 0.64 (2 x s, 3 H) ppm; ^{13}C NMR (125 MHz, $CDCl_3$) δ 170.69 (2 signals), 154.24 (2 signals), 147.00 (2 signals), 141.87 (2 signals), 138.36 (2 signals), 135.88, 135.60, 130.45 (2 signals), 129.17, 129.15, 128.84, 128.09, 127.99, 125.83 (2 signals), 120.93 (2 signals), 119.20, 116.91, 114.61, 114.23, 112.32, 41.01, 28.07 (2 signals), 19.56 (2 signals), 17.80 (2 signals), 10.57 (2 signals), −3.35 (2 signals) ppm; ^{19}F NMR (470 MHz, $CDCl_3$) δ −75.80 ppm; HRMS (ESI) *m/z*: $[M]^+$ calcd for $C_{28}H_{34}IN_2Si$, 553.1530; found 553.1521.

***N*-(10-(2,6-Dimethoxyphenyl)-7-(dimethylamino)-5-(3-iodopropyl)-5-methyldibenzo[*b*,*e*]silin-3(5*H*)-ylidene)-*N*-methylmethanaminium (51).**

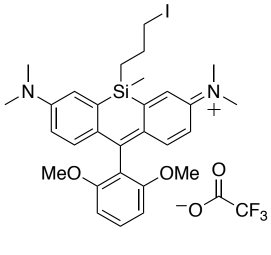

The same procedure was used as described above for compound **12-I**. A solution of **43** (0.40 g, 0.65 mmol) in anhydrous acetone (25.0 mL) was treated with NaI (0.39 g, 2.58 mmol) to provide **51** (0.35 g, 76%) as a dark blue color solid. ^{1}H NMR (500 MHz, $CDCl_3$) δ 7.47 (t, J = 8.0 Hz, 1 H), 7.23 (d, J = 9.5 Hz, 2 H), 7.20 (d, J = 3.0 Hz, 2 H), 6.71 (d, J = 6.5 Hz, 1 H), 6.69 (d, J = 6.0 Hz, 1 H), 6.64 (dd, J = 9.5, 2.5 Hz, 2 H), 3.68 (s, 6 H), 3.40 (s, 12 H), 3.14 (t, J = 7.5 Hz, 2 H), 1.79–1.70 (m, 2 H), 1.30–1.22 (m, 2 H), 0.73 (s, 3 H) ppm; ^{13}C NMR (125 MHz, $CDCl_3$) δ 167.14, 157.48, 157.44, 154.28, 146.79, 141.20, 131.15, 128.89, 120.75, 115.90, 114.24, 104.09, 103.91, 56.44, 56.27, 41.59, 28.06, 18.44,

11.23, −2.92 ppm; ^{19}F NMR (470 MHz, $CDCl_3$) δ −75.04 ppm; HRMS (ESI) *m/z*: $[M]^+$ calcd for $C_{29}H_{36}IN_2O_2Si$, 599.1585; found 599.1577.

(5*s*,10*s*)-3,7-Bis(dimethylamino)-5-(3-iodopropyl)-5-methyl-3'*H*,5*H*-spiro[dibenzo[*b*,*e*]siline-10,1'-isobenzofuran]-3'-one (52a).

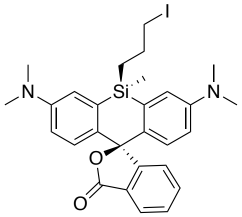

A solution of **49a** (0.23 g, 0.47 mmol) in anhydrous acetone (15.0 mL) under argon atmosphere was treated with NaI (0.28 g, 1.87 mmol) at room temperature and reaction mixture was stirred at 80 °C for 18 h. After completion of the reaction, cooled to room temperature and solvent was evaporated under reduced pressure. The resulting residue was purified by flash column chromatography (Silicycle column, 25 g, 0–25% EtOAc in 20% v/v DCM/Hexanes, linear gradient for 20 min) to provide **52a** (0.19 g, 73%) as a light green color solid. ^{1}H NMR (500 MHz, $CDCl_3$) δ 7.97 (dt, J = 7.5, 1.0 Hz, 1 H), 7.65 (td, J = 7.5, 1.0 Hz, 1 H), 7.56 (td, J = 7.5, 1.0 Hz, 1 H), 7.30 (d, J = 7.5 Hz, 1 H), 6.94 (br s, 2 H), 6.73 (d, J = 9.0 Hz, 2 H), 6.55 (d, J = 7.5 Hz, 2 H), 3.17 (t, J = 7.0 Hz, 2 H), 2,97 (s, 12 H), 1.95–1.86 (m, 2 H), 1.24–1.17 (m, 2 H), 0.65 (s, 3 H) ppm; ^{13}C NMR (125 MHz, $CDCl_3$) δ 170.61, 154.28, 149.39, 135.72, 133.81, 132.20, 128.92, 128.68, 127.52, 125.74, 124.82, 116.76, 113.57, 92.05, 40.43, 28.83, 18.40, 12.01, −3.45 ppm; HRMS (ESI) *m/z*: $[M + H]^+$ calcd for $C_{28}H_{32}IN_2O_2Si$, 583.1272; found 583.1260.

(5*r*,10*r*)-3,7-Bis(dimethylamino)-5-(3-iodopropyl)-5-methyl-3'*H*,5*H*-spiro[dibenzo[*b*,*e*]siline-10,1'-isobenzofuran]-3'-one (52b).

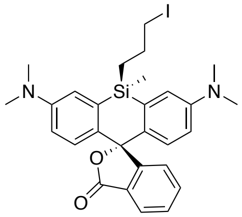

The same procedure was used as described above for compound **52a**. A solution of **49b** (0.20 g, 0.41 mmol) in anhydrous acetone (12.0 mL) was treated with NaI (0.25 g, 1.64 mmol) to provide **52b** (0.17 g, 71%) as a light green color solid. ^{1}H NMR (500 MHz, $CDCl_3$) δ 7.97 (dt, J = 7.5, 0.5 Hz, 1 H), 7.63 (td, J = 7.5, 1.0 Hz, 1 H), 7.53 (td, J = 7.5, 0.5 Hz, 1 H), 7.24 (d, J = 7.5 Hz, 1 H), 6.92 (br s, 2 H), 6.77 (d, J = 9.0 Hz, 2 H), 6.57 (d, J = 7.5 Hz, 2 H), 3.25 (t, J = 7.0 Hz, 2 H), 2,97 (s, 12 H), 1.97–1.88 (m, 2 H), 1.30–1.24 (m, 2 H), 0.60 (s, 3 H) ppm; ^{13}C NMR (125 MHz, $CDCl_3$) δ 170.88, 154.95, 149.36, 134.81, 134.03, 132.15, 128.88, 128.81, 127.03, 125.69, 124.41, 116.35, 113.85, 91.74, 40.45, 28.82, 16.84, 12.17, −1.16 ppm; HRMS (ESI) *m/z*: $[M + H]^+$ calcd for $C_{28}H_{32}IN_2O_2Si$, 583.1272; found 583.1263.

(5*r*,10*r*)-3,7-Di(azetidin-1-yl)-5-(3-iodopropyl)-5-methyl-3'*H*,5*H*-spiro[dibenzo[*b*,*e*]siline-10,1'-isobenzofuran]-3'-one (53b).

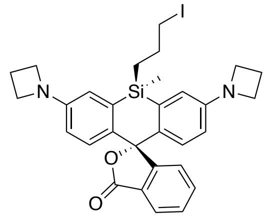

The same procedure was used as described above for compound **52a**. A solution of **50b** (50.0 mg, 0.097 mmol) in anhydrous acetone (5.0 mL) was treated with NaI (58.0 mg, 0.39 mmol) to provide **53b** (48.0 mg, 81%) as a light green color solid. ^{1}H NMR (500 MHz, $CDCl_3$) δ 7.96 (d, J = 8.0 Hz, 1 H), 7.64 (td, J = 7.5, 1.0 Hz, 1 H), 7.54 (td, J = 8.0, 0.5 Hz, 1 H), 7.25 (d, J = 8.0 Hz, 1 H), 6.74 (d, J = 8.5 Hz, 2 H), 6.63 (d, J = 2.0 Hz, 2 H), 6.28 (dd, J = 8.5, 2.5 Hz, 2 H), 3.90 (t, J = 7.0 Hz, 8 H), 3.24 (t, J = 6.5 Hz, 2 H), 2.37 (p, J = 7.0 Hz, 4 H), 1.92–1.83 (m, 2 H), 1.28–1.21 (m, 2 H), 0.58 (s, 3 H) ppm; ^{13}C NMR (125 MHz, $CDCl_3$) δ 170.80, 154.70, 150.91, 134.76, 133.99, 133.08, 128.86, 128.63, 127.04, 125.76, 124.53, 115.43, 112.74, 91.86, 52.43, 28.72, 17.04, 16.65, 12.14, −1.19 ppm; HRMS (ESI) *m/z*: $[M + H]^+$ calcd for $C_{30}H_{32}IN_2O_2Si$, 607.1272; found 607.1273.

***N*-(5-(3-Azidopropyl)-10-(2,6-dimethoxyphenyl)-7-(dimethylamino)-5-methyldibenzo[*b*,*e*]silin-3(5*H*)-ylidene)-*N*-methylmethanaminium (54).**

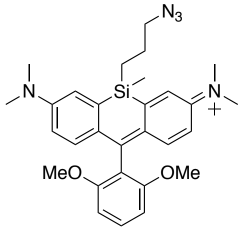

A solution of **51** (40.0 mg, 0.06 mmol) in anhydrous DMF (1.0 mL) under argon atmosphere was treated with NaN_3 (18.0 mg, 0.28 mmol) at room temperature and reaction mixture was stirred at room temperature for 12 h. After completion of the reaction, solvent was evaporated under reduced pressure. The resulting residue was purified by flash column chromatography (Biotage column, 10 g, 0–10% MeOH/DCM, linear gradient for 20 min) to provide **54** (25.0 mg, 83%) as a dark blue color solid. ^{1}H NMR (500 MHz, $CDCl_3$) δ 7.46 (t, J = 8.5 Hz, 1 H), 7.22 (d, J = 10.0 Hz, 2 H), 7.17 (d, J = 3.0 Hz, 2 H), 6.70 (t, J = 8.0 Hz, 2 H), 6.63 (dd, J = 9.5, 2.5 Hz, 2 H), 3.67 (s, 3 H), 3.64 (s, 3 H), 3.38 (s, 12 H), 3.18 (t, J = 7.0 Hz, 2 H), 1.56–1.47 (m, 2 H), 1.19–1.13 (m, 2 H), 0.72 (s, 3 H) ppm; ^{13}C NMR (125 MHz, $CDCl_3$) δ 167.23, 157.44, 157.35, 154.25, 146.82, 141.19, 131.15, 128.87, 120.54, 115.82, 114.21, 104.07, 103.90, 56.25, 56.19, 53.74, 41.51, 23.13, 14.07, −3.21 ppm; HRMS (ESI) *m/z*: $[M]^+$ calcd for $C_{29}H_{36}N_5O_2Si$, 514.2633; found 514.2626.

(5*s*,10*s*)-5-(3-Azidopropyl)-3,7-bis(dimethylamino)-5-methyl-3'*H*,5*H*-spiro[dibenzo[*b*,*e*]siline-10,1'-isobenzofuran]-3'-one (55a).

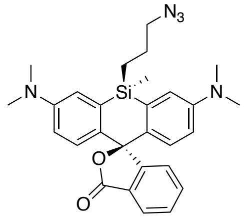

A solution of **52a** (30.0 mg, 0.05 mmol) in anhydrous DMF (1.0 mL) under argon atmosphere was treated with NaN_3 (17.0 mg, 0.26 mmol) at room temperature and reaction mixture was stirred at room temperature for 12 h. After completion of the reaction, solvent was evaporated under reduced pressure. The resulting residue was purified by flash column chromatography (Biotage column, 10 g, 0–50% EtOAc/DCM, linear gradient for 20 min)

to provide **55a** (22.0 mg, 85%) as a light green color solid. ^1H NMR (500 MHz, $CDCl_3$) δ 7.97 (d, J = 7.5 Hz, 1 H), 7.67 (td, J = 7.5, 1.0 Hz, 1 H), 7.57 (td, J = 7.0, 0.5 Hz, 1 H), 7.31 (d, J = 7.5 Hz, 1 H), 6.95 (br s, 2 H), 6.73 (d, J = 9.0 Hz, 2 H), 6.56 (d, J = 7.0 Hz, 2 H), 3.20 (t, J = 7.0 Hz, 2 H), 2,97 (s, 12 H), 1.75–1.66 (m, 2 H), 1.18–1.11 (m, 2 H), 0.66 (s, 3 H) ppm; ^{13}C NMR (125 MHz, $CDCl_3$) δ 170.55, 154.12, 149.37, 135.93, 133.80, 132.21, 128.97, 128.73, 127.65, 125.74, 124.91, 116.82, 113.57, 92.09, 54.24, 40.24, 23.87, 13.80, −3.59 ppm; HRMS (ESI) *m/z*: $[M + H]^+$ calcd for $C_{28}H_{32}N_5O_2Si$, 498.2320; found 498.2312.

(5*r*,10*r*)-5-(3-Azidopropyl)-3,7-bis(dimethylamino)-5-methyl-3'*H*,5*H*-spiro[dibenzo[*b*,*e*]siline-10,1'-isobenzofuran]-3'-one (55b).

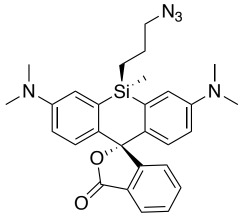

The same procedure was used as described above for compound **55a**. A solution of **52b** (50.0 mg, 0.09 mmol) in anhydrous DMF (1.0 mL) was treated with NaN_3 (28.0 mg, 0.43 mmol) to provide **55b** (38.0 mg, 88%) as a light green color solid. ^1H NMR (500 MHz, $CDCl_3$) δ 7.97 (d, J = 7.5 Hz, 1 H), 7.62 (td, J = 7.0, 0.5 Hz, 1 H), 7.53 (td, J = 7.0, 0.5 Hz, 1 H), 7.22 (d, J = 7.5 Hz, 1 H), 6.92 (br s, 2 H), 6.78 (d, J = 8.5 Hz, 2 H), 6.58 (d, J = 7.5 Hz, 2 H), 3.28 (t, J = 6.5 Hz, 2 H), 2,97 (s, 12 H), 1.79–1.69 (m, 2 H), 1.24–1.16 (m, 2 H), 0.60 (s, 3 H) ppm; ^{13}C NMR (125 MHz, $CDCl_3$) δ 170.89, 155.10, 149.34, 134.77, 134.07, 132.07, 128.93, 128.83, 126.95, 125.71, 124.31, 116.27, 113.92, 91.66, 54.40, 40.42, 24.14, 12.54, −1.29 ppm; HRMS (ESI) *m/z*: $[M + H]^+$ calcd for $C_{28}H_{32}N_5O_2Si$, 498.2320; found 498.2312.

Ethyl 2-((3-((5*s*,10*s*)-3,7-bis(dimethylamino)-5-methyl-3'-oxo-3'*H*,5*H*-spiro[dibenzo[*b*,*e*]siline-10,1'-isobenzofuran]-5-yl)propyl)thio)acetate (56a).

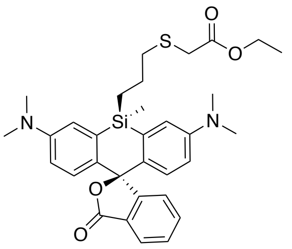

A solution of **52a** (0.10 g, 0.17 mmol) in anhydrous DMF (3.0 mL) under argon atmosphere was treated with DIPEA (90.0 μL, 0.52 mmol) and ethyl thioglycolate (31.0 μL, 0.26 mmol) at room temperature and reaction mixture was stirred at room temperature for 18 h. After completion of the reaction, solvent was evaporated under reduced pressure. The resulting residue was purified by flash column chromatography (Biotage column, 10 g, 0–50% EtOAc/DCM, linear gradient for 20 min) to provide **56a** (95.0 mg, 94%) as a light green color solid. ^{1}H NMR (500 MHz, $CDCl_3$) δ 7.97 (d, J = 8.0 Hz, 1 H), 7.65 (td, J = 7.5, 1.0 Hz, 1 H), 7.56 (td, J = 7.0, 0.5 Hz, 1 H), 7.29 (d, J = 7.5 Hz, 1 H), 6.95 (br s, 2 H), 6.74 (d, J = 9.0 Hz, 2 H), 6.56 (br s, 2 H), 4.13 (q, J = 7.0 Hz, 2 H), 3.15 (s, 2 H), 2,97 (s, 12 H), 2.62 (t, J = 7.5 Hz, 2 H), 1.75–1.66 (m, 2 H), 1.24 (t, J = 7.5 Hz, 3 H), 1.26–1.18 (m, 2 H, overlapping), 0.64 (s, 3 H) ppm; ^{13}C NMR (125 MHz, $CDCl_3$) δ 170.83, 170.62, 154.24, 149.30, 136.00, 133.82, 132.21, 128.92, 128.65, 127.46, 125.74, 124.82, 116.91, 113.54, 92.00, 61.38, 40.46, 36.19, 33.85, 23.90, 15.95, 14.30, −3.41 ppm; HRMS (ESI) *m/z*: $[M + H]^+$ calcd for $C_{32}H_{39}N_2O_4SSi$, 575.2394; found 575.2382.

Ethyl 2-((3-((5*r*,10*r*)-3,7-bis(dimethylamino)-5-methyl-3'-oxo-3'*H*,5*H*-spiro[dibenzo[*b*,*e*]siline-10,1'-isobenzofuran]-5-yl)propyl)thio)acetate (56b).

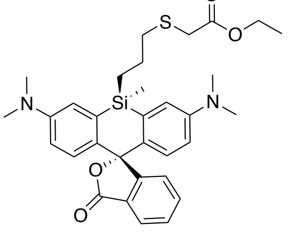

The same procedure was used as described above for compound **56a**. A solution of **52b** (0.10 g, 0.17 mmol) in anhydrous DMF (3.0 mL) was treated with DIPEA (90.0 μL, 0.52

mmol) and ethyl thioglycolate (31.0 μL, 0.26 mmol) to provide **56b** (92.0 mg, 93%) as a light green color solid. ^{1}H NMR (500 MHz, $CDCl_3$) δ 7.96 (d, J = 8.0 Hz, 1 H), 7.61 (td, J = 7.5, 1.0 Hz, 1 H), 7.52 (td, J = 7.0, 0.5 Hz, 1 H), 7.23 (d, J = 8.0 Hz, 1 H), 6.92 (br s, 2 H), 6.78 (d, J = 9.0 Hz, 2 H), 6.57 (d, J = 9.0 Hz, 2 H), 4.13 (q, J = 7.0 Hz, 2 H), 3.15 (s, 2 H), 2,96 (s, 12 H), 2.70 (t, J = 7.0 Hz, 2 H), 1.80–1.71 (m, 2 H), 1.29–1.23 (m, 2 H), 1.24 (t, J = 7.0 Hz, 3 H, overlapping), 0.58 (s, 3 H) ppm; ^{13}C NMR (125 MHz, $CDCl_3$) δ 170.99, 170.63, 155.24, 149.33, 134.92, 134.05, 132.09, 128.81, 128.74, 126.84, 125.66, 124.32, 116.41, 113.85, 91.70, 61.41, 40.42, 36.38, 33.80, 23.99, 14.71, 14.30, −1.28 ppm; HRMS (ESI) *m/z*: $[M + H]^+$ calcd for $C_{32}H_{39}N_2O_4SSi$, 575.2394; found 575.2382.

Ethyl 2-((3-((5*r*,10*r*)-3,7-di(azetidin-1-yl)-5-methyl-3'-oxo-3'*H*,5*H*-spiro[dibenzo[*b*,*e*]siline-10,1'-isobenzofuran]-5-yl)propyl)thio)acetate (57b).

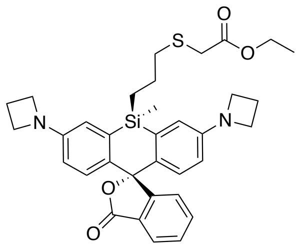

The same procedure was used as described above for compound **56a**. A solution of **53b** (45.0 mg, 0.074 mmol) in anhydrous DMF (1.0 mL) was treated with DIPEA (40.0 μL, 0.23 mmol) and ethyl thioglycolate (17.0 μL, 0.15 mmol) to provide **57b** (40.0 mg, 91%) as a light green color solid. ^{1}H NMR (500 MHz, $CDCl_3$) δ 7.96 (d, J = 7.5 Hz, 1 H), 7.63 (td, J = 8.0, 1.0 Hz, 1 H), 7.53 (td, J = 7.5, 0.5 Hz, 1 H), 7.23 (d, J = 7.5 Hz, 1 H), 6.78 (d, J = 9.0 Hz, 2 H), 6.70 (br s, 2 H), 6.35 (br s, 2 H), 4.14 (q, J = 7.0 Hz, 2 H), 3.94 (t, J = 7.0 Hz, 8 H), 3.16 (s, 2 H), 2.69 (t, J = 7.0 Hz, 2 H), 2.39 (p, J = 7.0 Hz, 4 H), 1.75–1.67 (m, 2 H), 1.28–1.21 (m, 2 H), 1.25 (t, J = 7.0 Hz, 3 H, overlapping), 0.57 (s, 3 H) ppm; HRMS (ESI) *m/z*: $[M + H]^+$ calcd for $C_{34}H_{39}N_2O_4SSi$, 599.2394; found 599.2399.

2-((3-((5*s*,10*s*)-3,7-Bis(dimethylamino)-5-methyl-3'-oxo-3'*H*,5*H*-spiro[dibenzo[*b*,*e*]siline-10,1'-isobenzofuran]-5-yl)propyl)thio)acetic acid (58a).

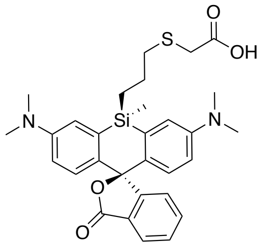

A solution of **56a** (90.0 mg, 0.16 mmol) in a mixture of anhydrous MeOH/THF (1:1, 4.0 mL) under argon atmosphere was treated with 1M NaOH (0.32 mL, 0.32 mmol). The reaction mixture was then stirred at room temperature for 2 h. Then the reaction mixture was acidified with 1M HCl (0.35 mL), diluted with H_2O (10.0 mL), and extracted with EtOAc (2 x 20.0 mL), the combined extracts were washed with saturated NaCl solution (25.0 mL), dried (Na_2SO_4), filtered, and evaporated under reduced pressure. The resulting residue was dried under high vacuum for four hours to provide acid **58a** (85.0 mg, 97%) as a blue color gum. This acid was used for next step without further purification. HRMS (ESI) *m/z*: $[M + H]^+$ calcd for $C_{30}H_{35}N_2O_4SSi$, 547.2081; found 547.2075.

2-((3-((5*r*,10*r*)-3,7-Bis(dimethylamino)-5-methyl-3'-oxo-3'*H*,5*H*-spiro[dibenzo[*b*,*e*]siline-10,1'-isobenzofuran]-5-yl)propyl)thio)acetic acid (58b).

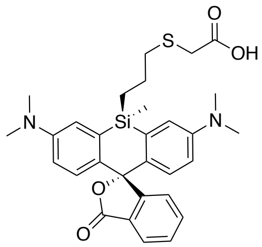

The same procedure was used as described above for compound **58a**. A solution of **56b** (90.0 mg, 0.16 mmol) in a mixture of anhydrous MeOH/THF (1:1, 4.0 mL) was treated with 1M NaOH (0.32 mL, 0.32 mmol) to provide acid **58b** (86.0 mg, 97%) as a blue color gum. HRMS (ESI) *m/z*: $[M + H]^+$ calcd for $C_{30}H_{35}N_2O_4SSi$, 547.2081; found 547.2073.

2-((3-((5*r*,10*r*)-3,7-Di(azetidin-1-yl)-5-methyl-3'-oxo-3'*H*,5*H*-spiro[dibenzo[*b*,*e*]siline-10,1'-isobenzofuran]-5-yl)propyl)thio)acetic acid (59b).

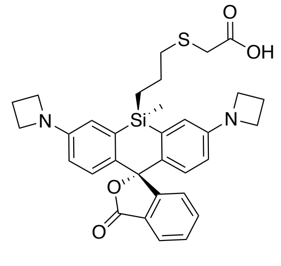

The same procedure was used as described above for compound **58a**. A solution of **57b** (32.0 mg, 0.054 mmol) in a mixture of anhydrous MeOH/THF (1:1, 2.0 mL) was treated with 1M NaOH (0.11 mL, 0.11 mmol) to provide acid **59b** (30.0 mg, 98%) as a blue color solid. HRMS (ESI) *m/z*: $[M + H]^+$ calcd for $C_{32}H_{35}N_2O_4SSi$, 571.2081; found 571.2092.

Applications of clickable dyes with methyltetrazine NHS ester and DBCO NHS ester/NHBoc reagents

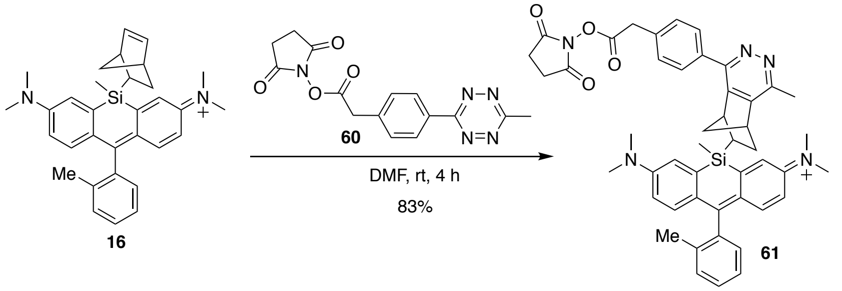

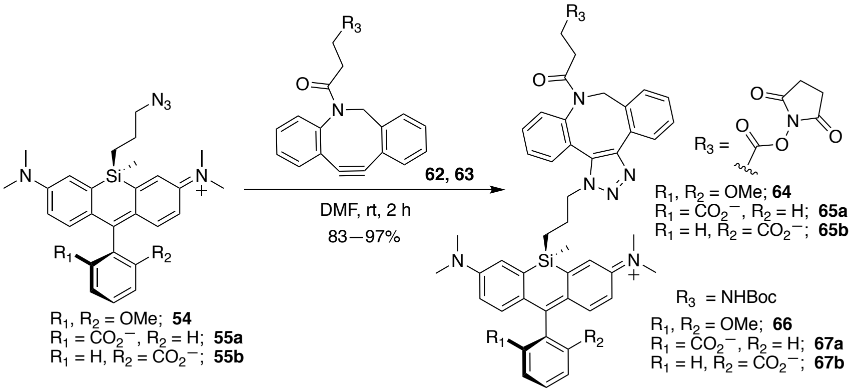

N-(7-(Dimethylamino)-5-((6S)-4-(4-(2-((2,5-dioxopyrrolidin-1-yl)oxy)-2-oxoethyl)phenyl)-1-methyl-5,6,7,8-tetrahydro-5,8-methanophthalazin-6-yl)-5-methyl-10-(o-tolyl)dibenzo[b,e]silin-3(5H)-ylidene)-N-methylmethanaminium (61).

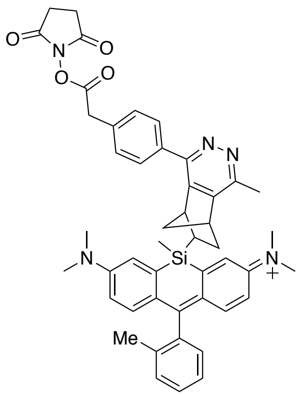

A solution of **16** (18.0 mg, 0.03 mmol) in anhydrous DMF (0.5 mL) under argon atmosphere was treated with methyltetrazine-NHS ester **60** (12.0 mg, 0.04 mmol) at room temperature and reaction mixture was stirred at room temperature for 4 h. After completion of the reaction, solvent was evaporated under reduced pressure. The resulting residue was purified by flash column chromatography (Biotage column, 10 g, 0–15% MeOH/DCM, linear gradient for 20 min) to provide **61** as an inseparable mixture of isomers (exo/endo and atropisomers) (20.0 mg, 83%) in dark blue color solid. Although the NMR spectra were not interpretable, HRMS analyses were consistent with the expected cycloaddition products mixture; HRMS (ESI) *m/z*: $[M]^+$ calcd for $C_{47}H_{48}N_5O_4Si$, 774.3470; found 774.3470.

_N_-(10-(2,6-Dimethoxyphenyl)-7-(dimethylamino)-5-(3-(8-(4-((2,5-dioxopyrrolidin-1-yl)oxy)-4-oxobutanoyl)-8,9-dihydro-3_H_-dibenzo[_b_,_f_][1,2,3]triazolo[4,5-_d_]azocin-3-yl)propyl)-5-methyldibenzo[_b_,_e_]silin-3(5_H_)-ylidene)-_N_-methylmethanaminium (64).

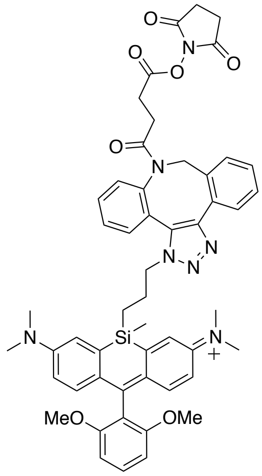

A solution of **54** (10.0 mg, 0.016 mmol) in anhydrous DMF (0.5 mL) under argon atmosphere was treated with DBCO-NHS-Ester **62** (8.0 mg, 0.019 mmol) at room temperature and reaction mixture was stirred at room temperature for 2 h. After completion of the reaction, solvent was evaporated under reduced pressure. The resulting residue was purified by flash column chromatography (Biotage column, 10 g, 0–15% MeOH/DCM, linear gradient for 20 min) to provide **64** as a mixture of regioisomers (13.5 mg, 94%) in dark blue color solid. Although the NMR spectra were not interpretable, HRMS analyses were consistent with the expected product mixture. HRMS (ESI) *m/z*: $[M]^+$ calcd for $C_{52}H_{54}N_7O_7Si$, 916.3849; found 916.3843.

2,5-Dioxopyrrolidin-1-yl 4-(3-(3-((5*s*,10*s*)-3,7-bis(dimethylamino)-5-methyl-3'-oxo-3'*H*,5*H*-spiro[dibenzo[*b*,*e*]siline-10,1'-isobenzofuran]-5-yl)propyl)-3,9-dihydro-8*H*-dibenzo[*b*,*f*][1,2,3]triazolo[4,5-*d*]azocin-8-yl)-4-oxobutanoate (65a)[3]**.**

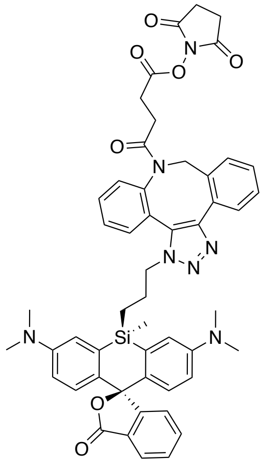

A solution of **55a** (10.0 mg, 0.02 mmol) in anhydrous DMF (0.5 mL) under argon atmosphere was treated with DBCO-NHS-Ester **62** (10.0 mg, 0.024 mmol) at room temperature and reaction mixture was stirred at room temperature for 2 h. After completion of the reaction, solvent was evaporated under reduced pressure. The resulting residue was purified by flash column chromatography (Biotage column, 10 g, 0–80% EtOAc/DCM, linear gradient for 20 min) to provide **65a** as a mixture of regioisomers (17.0 mg, 97%) in light green color solid. Although the NMR spectra were not interpretable, HRMS analyses were consistent with the expected product mixture. HRMS (ESI) *m/z*: $[M + H]^+$ calcd for $C_{51}H_{50}N_7O_7Si$, 900.3536; found 900.3528.

2,5-Dioxopyrrolidin-1-yl 4-(3-(3-((5*r*,10*r*)-3,7-bis(dimethylamino)-5-methyl-3'-oxo-3'*H*,5*H*-spiro[dibenzo[*b*,*e*]siline-10,1'-isobenzofuran]-5-yl)propyl)-3,9-dihydro-8*H*-dibenzo[*b*,*f*][1,2,3]triazolo[4,5-*d*]azocin-8-yl)-4-oxobutanoate (65b).

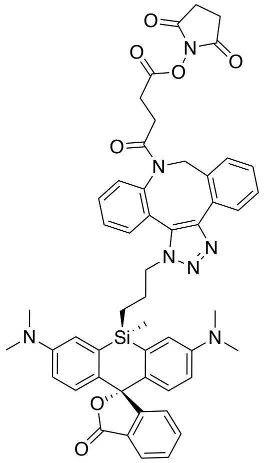

The same procedure was used as described above for compound **65a**. A solution of **55b** (25.0 mg, 0.05 mmol) in anhydrous DMF (1.0 mL) was treated with DBCO-NHS-Ester **62** (24.0 mg, 0.06 mmol) to provide **65b** as a mixture of regioisomers (42.0 mg, 96%) in light green color solid. Although the NMR spectra were not interpretable, HRMS analyses were consistent with the expected product mixture. HRMS (ESI) *m/z*: $[M + H]^+$ calcd for $C_{51}H_{50}N_7O_7Si$, 900.3536; found 900.3528.

***N*-(5-(3-(8-(3-((*tert*-butoxycarbonyl)amino)propanoyl)-8,9-dihydro-3*H*-dibenzo[*b*,*f*][1,2,3]triazolo[4,5-*d*]azocin-3-yl)propyl)-10-(2,6-dimethoxyphenyl)-7-(dimethylamino)-5-methyldibenzo[*b*,*e*]silin-3(5*H*)-ylidene)-*N*-methylmethanaminium (66).**

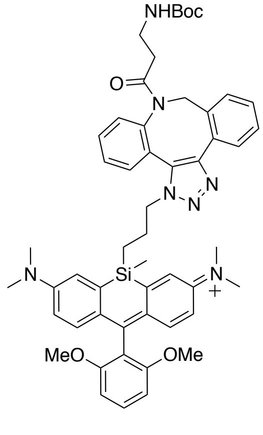

A solution of **54** (30.0 mg, 0.048 mmol) in anhydrous DMF (1.0 mL) under argon atmosphere was treated with DBCO-NH-Boc **63** (22.0 mg, 0.057 mmol) at room temperature and reaction mixture was stirred at room temperature for 2 h. After completion of the reaction, solvent was evaporated under reduced pressure. The resulting residue was purified by flash column chromatography (Biotage column, 10 g, 0–10% MeOH/DCM, linear gradient for 20 min) to provide **66** as a mixture of regioisomers (39.0 mg, 91%) in dark blue color solid. Although the NMR spectra were not interpretable, HRMS analyses were consistent with the expected product mixture. HRMS (ESI) *m/z*: $[M]^+$ calcd for $C_{52}H_{60}N_7O_5Si$, 890.4420; found 890.4427.

***tert*-Butyl (3-(3-(3-((5*s*,10*s*)-3,7-bis(dimethylamino)-5-methyl-3'-oxo-3'*H*,5*H*-spiro[dibenzo[*b*,*e*]siline-10,1'-isobenzofuran]-5-yl)propyl)-3,9-dihydro-8*H*-dibenzo[*b*,*f*][1,2,3]triazolo[4,5-*d*]azocin-8-yl)-3-oxopropyl)carbamate (67a).**

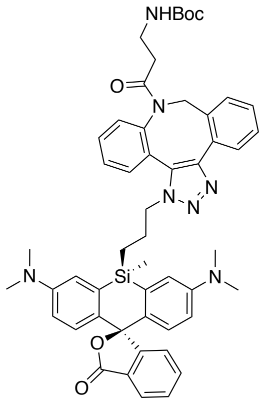

A solution of **55a** (20.0 mg, 0.04 mmol) in anhydrous DMF (1.0 mL) under argon atmosphere was treated with DBCO-NH-Boc **63** (18.0 mg, 0.05 mmol) at room temperature and reaction mixture was stirred at room temperature for 2 h. After completion of the reaction, solvent was evaporated under reduced pressure. The resulting residue was purified by flash column chromatography (Biotage column, 10 g, 0–80% EtOAc/DCM, linear gradient for 20 min) to provide **67a** as a mixture of regioisomers (33.0 mg, 95%) in light green color solid. Although the NMR spectra were not interpretable, HRMS analyses were consistent with the expected product mixture. HRMS (ESI) *m/z*: $[M + H]^+$ calcd for $C_{51}H_{56}N_7O_5Si$, 874.4107; found 874.4119.

***tert*-Butyl (3-(3-(3-((5*r*,10*r*)-3,7-bis(dimethylamino)-5-methyl-3'-oxo-3'*H*,5*H*-spiro[dibenzo[*b*,*e*]siline-10,1'-isobenzofuran]-5-yl)propyl)-3,9-dihydro-8*H*-dibenzo[*b*,*f*][1,2,3]triazolo[4,5-*d*]azocin-8-yl)-3-oxopropyl)carbamate (67b).**

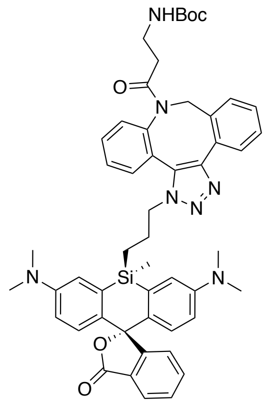

The same procedure was used as described above for compound **67a**. A solution of **55b** (20.0 mg, 0.04 mmol) in anhydrous DMF (1.0 mL) was treated with DBCO-NH-Boc **63** (18.0 mg, 0.048 mmol) to provide **67b** as a mixture of regioisomers (32.0 mg, 92%) in light green color solid. Although the NMR spectra were not interpretable, HRMS analyses were consistent with the expected product mixture. HRMS (ESI) *m/z*: $[M + H]^+$ calcd for $C_{51}H_{56}N_7O_5Si$, 874.4107; found 874.4120.

Synthesis of Si-Bridge Hoechst dyes

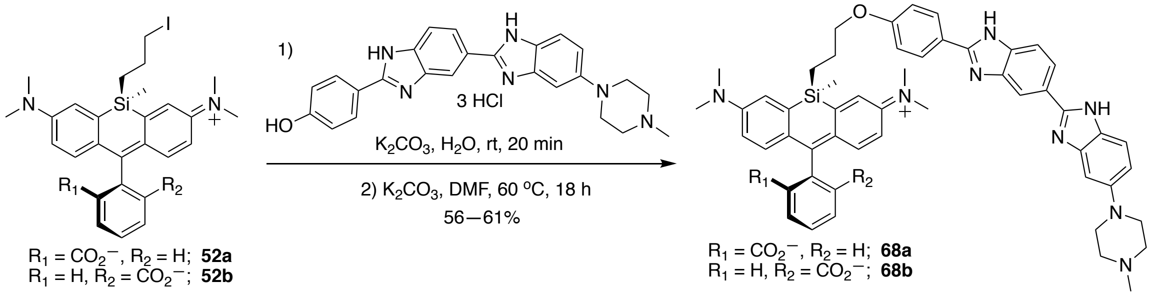

(5*s*,10*s*)-3,7-Bis(dimethylamino)-5-methyl-5-(3-(4-(5-(4-methylpiperazin-1-yl)-1*H*,1'*H*-[2,5'-bibenzo[*d*]imidazol]-2'-yl)phenoxy)propyl)-3'*H*,5*H*-spiro[dibenzo[*b*,*e*]siline-10,1'-isobenzofuran]-3'-one (68a).

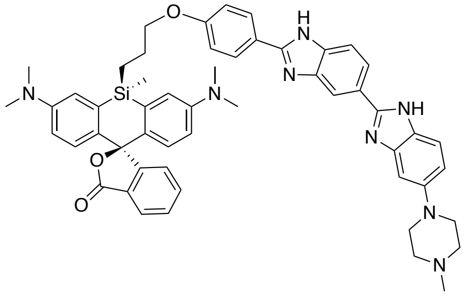

A solution of Hoechst 33258 trihydrochloride (0.15 g, 0.28 mmol) in H_2O (9.0 mL) under argon atmosphere was treated with a solution of K_2CO_3 (0.12 g, 0.84 mmol) in H_2O (3.0 mL). The reaction mixture was then stirred at room temperature for 20 min. The precipitate thus formed was isolated by filtration, washed with H_2O (5.0 mL) and high vacuumed for 24 hours to provide free base of Hoechst 33258 (0.11 g, 93%) as an off-white color solid. The Hoechst 33258 free base (40.0 mg, 0.094 mmol) in anhydrous DMF (2.0 mL) was treated with K_2CO_3 (39.0 mg, 0.28 mmol) and **52a** (66.0 mg, 0.11 mmol). After stirring the reaction at 60 °C for 18 h, solvent was evaporated under reduced pressure, the residue was dissolved in a mixture of MeOH/DCM (1:1 10.0 mL) and filtered through a small pad of celite to remove excess K_2CO_3. The resulting residue was purified by flash column chromatography (Biotage column, 10 g, 0–15% MeOH in 0.1% v/v TEA DCM, linear gradient for 20 min) to provide **68a** (50.0 mg, 61%) as a light green color solid. ^{1}H NMR

(500 MHz, CD_3OD) δ 8.23 (s, 1 H), 7.96 (d, J = 8.0 Hz, 2 H), 7.94 (d, J = 8.5 Hz, 2 H), 7.74 (td, J = 7.5, 1.0 Hz, 1 H), 7.70–7.63 (m, 1 H), 7.64 (td, J = 7.0, 0.5 Hz, 1 H, overlapping), 7.50 (d, J = 9.0 Hz, 1 H), 7.25 (d, J = 7.5 Hz, 1 H), 7.13 (s, 1 H), 7.03 (dd, J = 9.0, 2.0 Hz, 1 H), 7.00 (d, J = 2.5 Hz, 2 H), 6.87 (d, J = 8.5 Hz, 2 H), 6.69 (d, J = 9.0 Hz, 2 H), 6.60 (dd, J = 9.0, 3.0 Hz, 2 H), 3.85 (t, J = 6.5 Hz, 2 H), 3.22 (t, J = 4.5 Hz, 4 H), 2.90 (s, 12 H), 2.67 (t, J = 4.5 Hz, 4 H), 2.37 (s, 3 H), 1.90–1.81 (m, 2 H), 1.32–1.25 (m, 2 H), 0.65 (s, 3 H) ppm; ^{13}C NMR (125 MHz, CD_3OD) δ 173.05, 162.58, 156.53, 155.43, 151.00, 149.58, 141.60, 136.71, 135.57, 132.83, 130.24, 129.54, 129.41, 127.90, 126.25, 125.78, 125.68, 122.54, 117.78, 116.05, 114.90, 94.44, 70.79, 56.16, 51.80, 46.07, 40.39, 24.89, 13.01, −2.95 ppm; HRMS (ESI) *m/z*: $[M + H]^+$ calcd for $C_{53}H_{55}N_8O_3Si$, 879.4161; found 879.4155.

(5*r*,10*r*)-3,7-Bis(dimethylamino)-5-methyl-5-(3-(4-(5-(4-methylpiperazin-1-yl)-1*H*,1'*H*-[2,5'-bibenzo[*d*]imidazol]-2'-yl)phenoxy)propyl)-3'*H*,5*H*-spiro[dibenzo[*b*,*e*]siline-10,1'-isobenzofuran]-3'-one (68b).

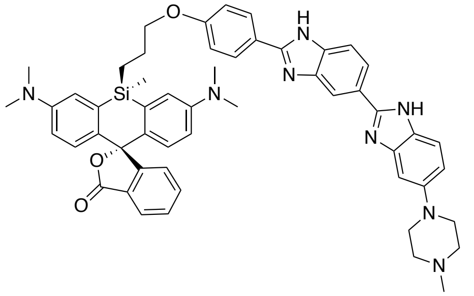

The same procedure was used as described above for compound **68a**. A solution of Hoechst 33258 free base (40.0 mg, 0.094 mmol) in anhydrous DMF (2.0 mL) was treated with K_2CO_3 (39.0 mg, 0.28 mmol) and **52b** (66.0 mg, 0.11 mmol) to provide **68b** (47.0 mg, 56%) as a light green color solid. ^{1}H NMR (500 MHz, CD_3OD) δ 8.25 (s, 1 H), 8.00 (d, J = 9.0 Hz, 2 H), 7.94 (d, J = 7.5 Hz, 2 H), 7.71 (td, J = 7.5, 1.0 Hz, 1 H), 7.70–7.66 (m, 1 H, overlapping), 7.61 (td, J = 7.5, 1.0 Hz, 1 H), 7.52 (d, J = 8.5 Hz, 1 H), 7.29 (d, J = 8.0 Hz, 1 H), 7.16 (s, 1 H), 7.04 (dd, J = 8.5, 2.0 Hz, 1 H), 7.01 (d, J = 3.0 Hz, 2 H), 6.94 (d, J = 9.0 Hz, 2 H), 6.69 (d, J = 9.0 Hz, 2 H), 6.60 (dd, J = 9.0, 3.0 Hz, 2 H), 3.98 (t, J = 6.5 Hz, 2 H), 3.29 (t, J = 4.5 Hz, 4 H), 2.94 (s, 12 H), 2.88 (t, J = 4.5 Hz, 4 H), 2.53 (s, 3 H), 1.97–1.88 (m, 2 H), 1.41–1.34 (m, 2 H), 0.59 (s, 3 H) ppm; ^{13}C NMR (125 MHz, CD_3OD)

δ 172.66, 161.96, 155.58, 154.93, 153.63, 150.44, 148.35, 136.11, 135.17, 132.36, 129.87, 129.51, 129.19, 127.70, 126.04, 125.45, 124.87, 122.47, 122.20, 117.29, 116.20, 115.73, 114.52, 94.24, 70.83, 55.34, 50.72, 45.05, 40.49, 24.80, 11.44, −1.02 ppm; HRMS (ESI) *m/z*: $[M + H]^+$ calcd for $C_{53}H_{55}N_8O_3Si$, 879.4161; found 879.4155.

Synthesis of thiol (O4) HaloTag ligand

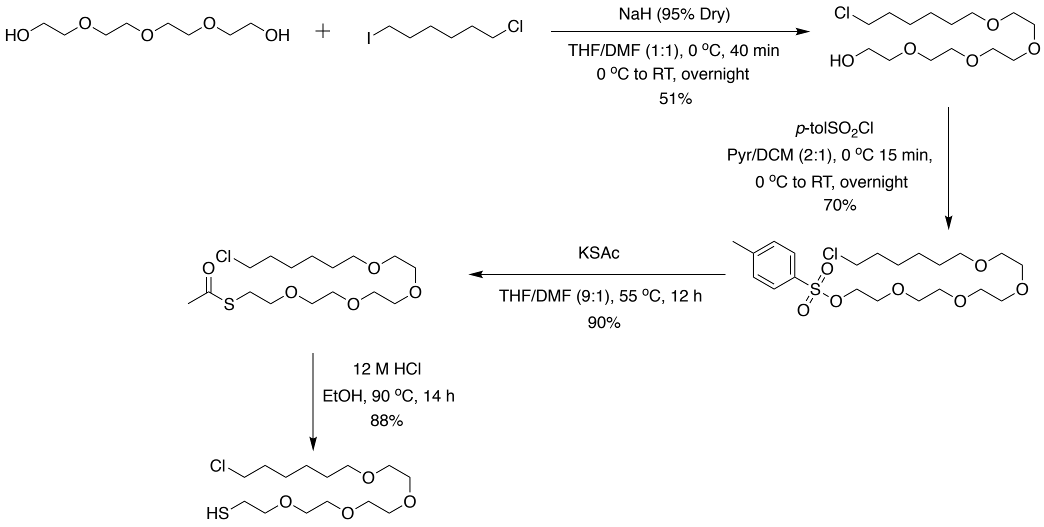

69

18-Chloro-3,6,9,12-tetraoxaoctadecan-1-ol.

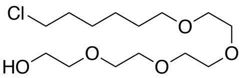

A solution of NaH (dry 95%) (0.49 g, 20.3 mmol) in a mixture of anhydrous THF/DMF (1:1, 80.0 mL) under argon atmosphere was cooled to 0 °C in an ice-water bath. After 10 min, tetraethylene glycol (7.04 mL, 40.6 mmol) was added dropwise. The resulting reaction mixture was stirred at 0 °C for 40 min. At the same temperature, 1-chloro-6-iodohexane (1.23 mL, 8.12 mmol) was added dropwise. The reaction mixture was then warmed to room temperature and stirred overnight. The reaction mixture was quenched with H_2O (10.0 mL), diluted with 1M HCl (50.0 mL) and extracted with $CHCl_3$ (2 x 100 mL), the combined extracts were washed with saturated NaCl solution (50.0 mL), dried (Na_2SO_4), filtered, and evaporated under reduced pressure. The resulting residue was purified by flash column chromatography (Silicycle column, 50 g, 25–100% EtOAc/Hexanes, linear gradient for 20

min) to provide 18-chloro-3,6,9,12-tetraoxaoctadecan-1-ol (1.30 g, 51%) as a color less oil. ^{1}H NMR (500 MHz, $CDCl_3$) δ 3.74–3.70 (m, 2 H), 3.69–3.63 (m, 10 H), 3.62–3.60 (m, 2 H), 3.59–3.56 (m, 2 H), 3.53 (t, J = 6.5 Hz, 2 H), 3.45 (t, J = 6.5 Hz, 2 H), 2,45 (br s, 1 H), 1.81–1.73 (m, 2 H), 1.63–1.55 (m, 2 H), 1.48–1.42 (m, 2 H), 1.40–1.33 (m, 2 H) ppm; ^{13}C NMR (125 MHz, $CDCl_3$) δ 72.62, 71.38, 70.77, 70.75, 70.73, 70.52, 70.24, 61.92, 45.19, 32.69, 29.58, 26.83, 25.56 ppm; HRMS (ESI) m/z: $[M + H]^+$ calcd for $C_{14}H_{30}ClO_5$, 313.1776; found 313.1772.

18-Chloro-3,6,9,12-tetraoxaoctadecyl 4-methylbenzenesulfonate.

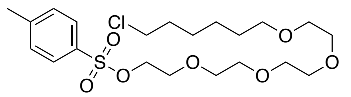

A solution of 18-chloro-3,6,9,12-tetraoxaoctadecan-1-ol (1.20 g, 3.84 mmol) in anhydrous pyridine (8.0 mL) under argon atmosphere was cooled to 0 °C in an ice-water bath. After 10 min, *p*-toluenesulfonyl chloride (1.83 mL, 9.60 mmol) in DCM (5.0 mL) was added dropwise. The reaction mixture was then warmed to room temperature and stirred overnight. Excess pyridine was evaporated under reduced pressure, diluted with 10% citric acid (25.0 mL) and extracted with DCM (2 x 50.0 mL), the combined extracts were washed with saturated NaCl solution (25.0 mL), dried (Na_2SO_4), filtered, and evaporated under reduced pressure. The resulting residue was purified by flash column chromatography (Silicycle column, 25 g, 20–80% EtOAc/Hexanes, linear gradient for 20 min) to provide 18-chloro-3,6,9,12-tetraoxaoctadecyl 4-methylbenzenesulfonate (1.25 g, 70%) as a color less oil. ^{1}H NMR (500 MHz, $CDCl_3$) δ 7.82–7.77 (m, 2 H), 7.36–7.31 (m, 2 H), 4.15 (t, J = 5.0 Hz, 2 H), 3.68 (t, J = 5.0 Hz, 2 H), 3.65–3.60 (m, 6 H), 3.59–3.55 (m, 6 H), 3.53 (t, J = 6.5 Hz, 2 H), 3.45 (t, J = 6.5 Hz, 2 H), 2,44 (s, 3 H), 1.81–1.73 (m, 2 H), 1.62–1.55 (m, 2 H), 1.48–1.41 (m, 2 H), 1.40–1.32 (m, 2 H) ppm; ^{13}C NMR (125 MHz, $CDCl_3$) δ 144.91, 133.17, 129.95, 128.13, 71.37, 70.90, 70.77, 70.73, 70.67, 70.24, 69.37, 68.82, 45.20, 32.68, 29.60, 26.83, 25.56, 21.78 ppm; HRMS (ESI) m/z: $[M + H]^+$ calcd for $C_{21}H_{36}ClO_7S$, 467.1865; found 467.1866.

***S*-(18-Chloro-3,6,9,12-tetraoxaoctadecyl) ethanethioate.**

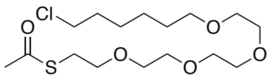

A solution of 18-chloro-3,6,9,12-tetraoxaoctadecyl 4-methylbenzenesulfonate (1.25 g, 2.68 mmol) in a mixture of anhydrous THF/DMF (9:1, 25.0 mL) under argon atmosphere was treated with potassium thioacetate (0.30 g, 2.63 mmol). The reaction mixture was then stirred at 55 °C for 12 hours. The reaction mixture was diluted with H_2O (25.0 mL) and extracted with EtOAc (2 x 50.0 mL), the combined extracts were washed with saturated NaCl solution (25.0 mL), dried (Na_2SO_4), filtered, and evaporated under reduced pressure. The resulting residue was purified by flash column chromatography (Silicycle column, 25 g, 20–80% EtOAc/Hexanes, linear gradient for 20 min) to provide *S*-(18-chloro-3,6,9,12-tetraoxaoctadecyl) ethanethioate (0.90 g, 90%) as a color less oil. ^{1}H NMR (500 MHz, $CDCl_3$) δ 3.67–3.56 (m, 14 H), 3.53 (t, J = 6.5 Hz, 2 H), 3.45 (t, J = 6.5 Hz, 2 H), 3.09 (t, J = 6.5 Hz, 2 H), 2.33 (s, 3 H), 1.81–1.73 (m, 2 H), 1.63–1.55 (m, 2 H), 1.49–1.41 (m, 2 H), 1.40–1.33 (m, 2 H) ppm; ^{13}C NMR (125 MHz, $CDCl_3$) δ 195.67, 71.38, 70.81, 70.78, 70.76, 70.67, 70.47, 70.26, 69.91, 45.20, 32.69, 30.71, 29.61, 28.99, 26.85, 25.58 ppm; HRMS (ESI) *m/z*: $[M + H]^+$ calcd for $C_{16}H_{32}ClO_5S$, 371.1653; found 371.1655.

18-Chloro-3,6,9,12-tetraoxaoctadecane-1-thiol (69).

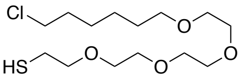

A solution of *S*-(18-chloro-3,6,9,12-tetraoxaoctadecyl) ethanethioate (0.90 g, 2.43 mmol) in anhydrous EtOH (12.5 mL) under argon atmosphere was treated with 12M HCl (0.65 mL). The reaction mixture was then stirred at 90 °C for 14 hours. The reaction mixture was concentrated to ~ 2.0 mL and then poured into H_2O (10.0 mL) and extracted with EtOAc (3 x 25.0 mL), the combined extracts were washed with saturated NH_4Cl solution (40.0 mL), dried (Na_2SO_4), filtered, and evaporated under reduced pressure. The resulting residue was purified by flash column chromatography (Silicycle column, 25 g, 20–80% EtOAc/Hexanes, linear gradient for 20 min) to provide **69** (0.70 g, 88%) as a color less oil. ^{1}H NMR (500 MHz, $CDCl_3$) δ 3.67–3.60 (m, 12 H), 3.59–3.56 (m, 2 H), 3.53 (t, J = 6.5

Hz, 2 H), 3.45 (t, J = 6.5 Hz, 2 H), 2.69 (dt, J = 8.5, 6.0 Hz, 2 H), 1.82–1.73 (m, 2 H), 1.63–1.55 (m, 3 H), 1.48–1.41 (m, 2 H), 1.40–1.33 (m, 2 H) ppm; ^{13}C NMR (125 MHz, $CDCl_3$) δ 73.03, 71.38, 70.81, 70.79, 70.78, 70.69, 70.39, 70.26, 45.20, 32.69, 29.61, 26.84, 25.57, 24.42 ppm; HRMS (ESI) *m/z*: $[M + H]^+$ calcd for $C_{14}H_{30}ClO_4S$, 329.1548; found 329.1545.

Synthesis of Si-Bridge thiol (O4) HaloTags

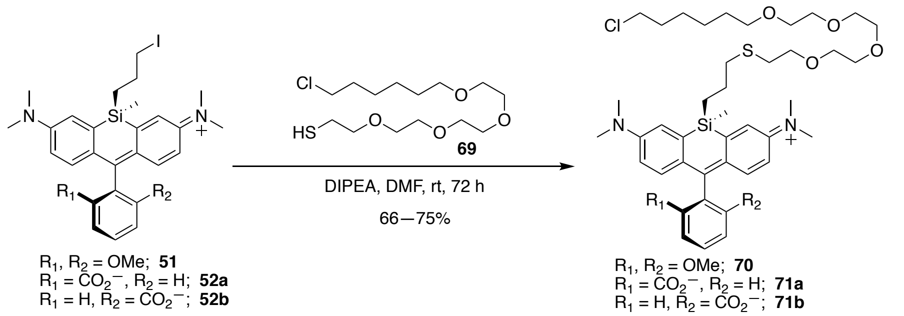

***N*-(5-(22-Chloro-7,10,13,16-tetraoxa-4-thiadocosyl)-10-(2,6-dimethoxyphenyl)-7-(dimethylamino)-5-methyldibenzo[*b*,*e*]silin-3(5*H*)-ylidene)-*N*-methylmethanaminium (70).**

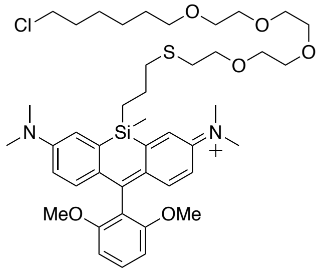

A solution of **51** (80.0 mg, 0.11 mmol) in anhydrous DMF (2.0 mL) under argon atmosphere was treated with DIPEA (59.0 μL, 0.34 mmol) and thiol (O4) HaloTag ligand **69** (55.0 μL, 0.17 mmol) at room temperature and reaction mixture was stirred at room temperature for 72 h. After completion of the reaction, solvent was evaporated under reduced pressure. The resulting residue was purified by flash column chromatography (Biotage column, 10 g, 0–15% MeOH/DCM, linear gradient for 20 min) to provide **70** (60.0 mg, 68%) as a dark blue color solid. ^{1}H NMR (500 MHz, $CDCl_3$) δ 7.46 (t, J = 8.5 Hz, 1 H), 7.23 (d, J = 9.5 Hz, 2 H), 7.13 (d, J = 3.0 Hz, 2 H), 6.71 (d, J = 6.0 Hz, 1 H), 6.69

(d, J = 6.5 Hz, 1 H), 6.63 (dd, J = 9.5, 2.5 Hz, 2 H), 3.67 (s, 3 H), 3.66 (s, 3 H), 3.66–3.61 (m, 8 H), 3.60–3.54 (m, 6 H), 3.52 (t, J = 6.5 Hz, 2 H), 3.46 (t, J = 6.5 Hz, 2 H), 3.35 (s, 12 H), 2.60 (t, J = 6.5 Hz, 2 H), 2.46 (t, J = 6.5 Hz, 2 H), 1.80–1.72 (m, 2 H), 1.62–1.55 (m, 2 H), 1.54–1.47 (m, 2 H), 1.46–1.40 (m, 2 H), 1.39–1.32 (m, 2 H), 1.16–1.10 (m, 2 H), 0.65 (s, 3 H) ppm; ^{13}C NMR (125 MHz, $CDCl_3$) δ 167.35, 157.50, 157.45, 154.26, 147.13, 141.25, 131.14, 128.93, 120.42, 115.91, 114.16, 104.09, 103.88, 71.39, 70.99, 70.68, 70.63, 70.61, 70.56, 70.28, 70.12, 56.26, 56.21, 45.24, 41.01, 35.51, 32.68, 31.29, 29.48, 26.84, 25.51, 23.66, 16.09, −3.53 ppm; HRMS (ESI) *m/z*: $[M]^+$ calcd for $C_{43}H_{64}ClN_2O_6SSi$, 799.3937; found 799.3926.

(5*s*,10*s*)-5-(22-Chloro-7,10,13,16-tetraoxa-4-thiadocosyl)-3,7-bis(dimethylamino)-5-methyl-3'*H*,5*H*-spiro[dibenzo[*b*,*e*]siline-10,1'-isobenzofuran]-3'-one (71a).

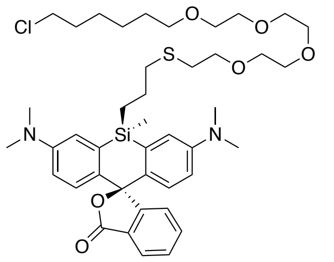

A solution of **52a** (50.0 mg, 0.09 mmol) in anhydrous DMF (2.0 mL) under argon atmosphere was treated with DIPEA (45.0 µL, 0.26 mmol) and thiol (O4) HaloTag ligand **69** (42.0 µL, 0.13 mmol) at room temperature and reaction mixture was stirred at room temperature for 72 h. After completion of the reaction, solvent was evaporated under reduced pressure. The resulting residue was purified by flash column chromatography (Biotage column, 10 g, 0–80% EtOAc/DCM, linear gradient for 20 min) to provide **71a** (50.0 mg, 75%) as a light green color solid. ^{1}H NMR (500 MHz, CD_3OD) δ 7.94 (d, J = 7.5 Hz, 1 H), 7.74 (td, J = 7.5, 1.0 Hz, 1 H), 7.63 (td, J = 7.5, 1.0 Hz, 1 H), 7.25 (d, J = 7.5 Hz, 1 H), 7.02 (d, J = 2.5 Hz, 2 H), 6.69 (d, J = 9.0 Hz, 2 H), 6.64 (dd, J = 9.0, 3.0 Hz, 2 H), 3.60–3.47 (m, 16 H), 3.43 (t, J = 6.5 Hz, 2 H), 2.97 (s, 12 H), 2.53 (t, J = 6.5 Hz, 2 H), 2.48 (t, J = 7.0 Hz, 2 H), 1.77–1.70 (m, 2 H), 1.66–1.59 (m, 2 H), 1.58–1.51 (m, 2 H), 1.47–1.40 (m, 2 H), 1.39–1.32 (m, 2 H), 1.23–1.17 (m, 2 H), 0.63 (s, 3 H) ppm; ^{13}C NMR (125 MHz, CD_3OD) δ 156.50, 151.05, 136.88, 135.50, 132.98, 130.21, 129.48, 127.84, 126.30, 125.80, 117.92, 114.94, 72.15, 71.57, 71.55, 71.54, 71.20, 71.18, 45.72, 40.51, 36.35,

33.76, 31.99, 30.56, 27.73, 26.50, 25.61, 16.26, −2.94 ppm; HRMS (ESI) *m/z*: $[M + H]^+$ calcd for $C_{42}H_{60}ClN_2O_6SSi$, 783.3624; found 783.3612.

(5*r*,10*r*)-5-(22-Chloro-7,10,13,16-tetraoxa-4-thiadocosyl)-3,7-bis(dimethylamino)-5-methyl-3'*H*,5*H*-spiro[dibenzo[*b*,*e*]siline-10,1'-isobenzofuran]-3'-one (71b).

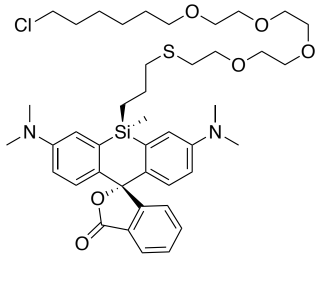

The same procedure was used as described above for compound **71a**. A solution of **52b** (50.0 mg, 0.09 mmol) in anhydrous DMF (2.0 mL) under argon atmosphere was treated with DIPEA (45.0 µL, 0.26 mmol) and thiol (O4) HaloTag ligand **69** (42.0 µL, 0.13 mmol) to provide **71b** (45.0 mg, 66%) as a light green color solid. ^1H NMR (500 MHz, CD_3OD) δ 7.95 (d, J = 7.5 Hz, 1 H), 7.75 (td, J = 7.5, 1.0 Hz, 1 H), 7.64 (td, J = 7.5, 1.0 Hz, 1 H), 7.29 (d, J = 7.5 Hz, 1 H), 7.02 (d, J = 3.0 Hz, 2 H), 6.69 (d, J = 9.0 Hz, 2 H), 6.64 (dd, J = 9.0, 2.5 Hz, 2 H), 3.58–3.47 (m, 16 H), 3.42 (t, J = 7.0 Hz, 2 H), 2.97 (s, 12 H), 2.60–2.53 (m, 4 H), 1.77–1.62 (m, 4 H), 1.58–1.50 (m, 2 H), 1.47–1.28 (m, 6 H), 0.55 (s, 3 H) ppm; ^{13}C NMR (125 MHz, CD_3OD) δ 172.99, 156.50, 151.00, 136.34, 135.63, 132.93, 130.24, 129.64, 127.88, 126.27, 125.74, 117.71, 114.97, 94.17, 72.19, 72.14, 71.59, 71.57, 71.56, 71.25, 71.19, 45.71, 40.52, 36.65, 33.75, 32.17, 30.56, 27.73, 26.50, 25.74, 14.72, −1.14 ppm; HRMS (ESI) *m/z*: $[M + H]^+$ calcd for $C_{42}H_{60}ClN_2O_6SSi$, 783.3624; found 783.3617.

Synthesis of Si-Bridge NHS esters

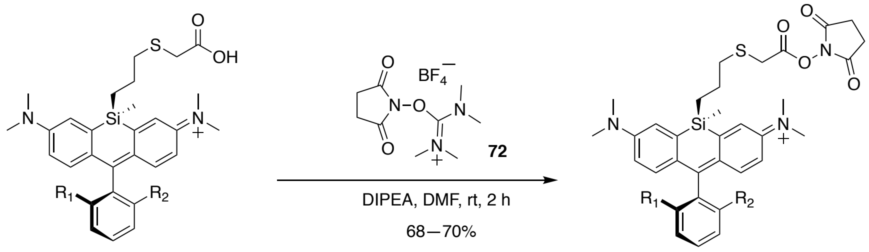

2,5-Dioxopyrrolidin-1-yl 2-((3-((5*s*,10*s*)-3,7-bis(dimethylamino)-5-methyl-3'-oxo-3'*H*,5*H*-spiro[dibenzo[*b*,*e*]siline-10,1'-isobenzofuran]-5-yl)propyl)thio)acetate (73a).

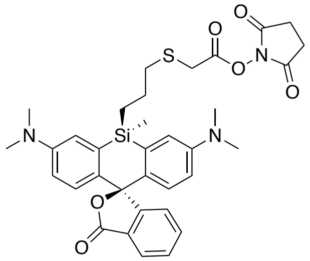

A solution of **58a** (60.0 mg, 0.11 mmol) in anhydrous DMF (2.0 mL) under argon atmosphere was treated with TSTU **72** (50.0 mg, 0.17 mmol) and DIPEA (58.0 µL, 0.33 mmol). After stirring the reaction at room temperature for 2 h, the reaction mixture was diluted with 10% w/v citric acid (5.0 mL) and extracted with EtOAc (2 x 10.0 mL). The combined extracts were washed with saturated NaCl solution (10.0 mL), dried (Na_2SO_4), filtered, and evaporated under reduced pressure. The resulting residue was purified by flash column chromatography (Silicycle column, 10 g, 0–100% EtOAc/DCM, linear gradient for 20 min) to provide **73a** (50.0 mg, 70%) as a light blue color solid. ^{1}H NMR (500 MHz, $CDCl_3$) δ 7.96 (d, J = 7.5 Hz, 1 H), 7.64 (td, J = 7.5, 1.0 Hz, 1 H), 7.55 (td, J = 7.0, 0.5 Hz, 1 H), 7.28 (d, J = 7.5 Hz, 1 H), 6.98 (br s, 2 H), 6.75 (d, J = 8.5 Hz, 2 H), 6.58 (br s, 2 H), 3.38 (s, 2 H), 2,97 (s, 12 H), 2,84 (s, 4 H), 2.71 (t, J = 7.5 Hz, 2 H), 1.78–1.69 (m, 2 H), 1.26–1.19 (m, 2 H), 0.64 (s, 3 H) ppm; HRMS (ESI) *m/z*: $[M + H]^+$ calcd for $C_{34}H_{38}N_3O_6SSi$, 644.2245; found 644.2248.

2,5-Dioxopyrrolidin-1-yl 2-((3-((5*r*,10*r*)-3,7-bis(dimethylamino)-5-methyl-3'-oxo-3'*H*,5*H*-spiro[dibenzo[*b*,*e*]siline-10,1'-isobenzofuran]-5-yl)propyl)thio)acetate (73b).

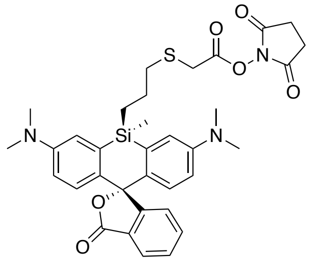

The same procedure was used as described above for compound **73a**. A solution of **58b** (60.0 mg, 0.11 mmol) in anhydrous DMF (2.0 mL) was treated with TSTU **72** (50.0 mg, 0.17 mmol) and DIPEA (58.0 μL, 0.33 mmol) to provide **73b** (48.0 mg, 68%) as a light blue color solid. ^{1}H NMR (500 MHz, $CDCl_3$) δ 7.96 (d, J = 7.5 Hz, 1 H), 7.62 (td, J = 7.0, 1.0 Hz, 1 H), 7.53 (td, J = 7.0, 0.5 Hz, 1 H), 7.24 (d, J = 8.0 Hz, 1 H), 6.96 (br s, 2 H), 6.79 (d, J = 9.0 Hz, 2 H), 6.60 (d, J = 6.0 Hz, 2 H), 3.41 (s, 2 H), 2,97 (s, 12 H), 2,83 (s, 4 H), 2.79 (t, J = 7.0 Hz, 2 H), 1.82–1.74 (m, 2 H), 1.32–1.26 (m, 2 H), 0.59 (s, 3 H) ppm; HRMS (ESI) *m/z*: $[M + H]^+$ calcd for $C_{34}H_{38}N_3O_6SSi$, 644.2245; found 644.2261.

Synthesis of amine (O2) HaloTag ligand

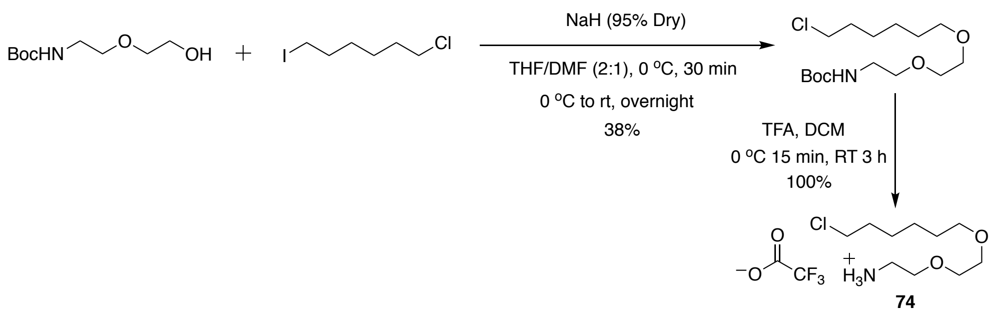

***tert*-Butyl (2-(2-((6-chlorohexyl)oxy)ethoxy)ethyl)carbamate.**

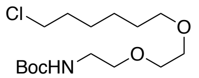

A solution of *tert*-butyl (2-(2-hydroxyethoxy)ethyl)carbamate (2.50 g, 12.2 mmol) in a mixture of anhydrous THF/DMF (2:1, 30.0 mL) under argon atmosphere was cooled to 0

°C in an ice-water bath. After 10 min, NaH (dry 95%) (0.35 g, 14.6 mmol) was added. The resulting reaction mixture was stirred at 0 °C for 30 min. At the same temperature, 1-chloro-6-iodohexane (2.80 mL, 18.3 mmol) was added dropwise. The reaction mixture was then warmed to room temperature and stirred overnight. The reaction mixture was then cooled to ~ 5 °C and quenched by addition of NH_4Cl (50.0 mL) and extracted with EtOAc (2 x 100 mL), the combined extracts were washed with saturated NaCl solution (50.0 mL), dried (Na_2SO_4), filtered, and evaporated under reduced pressure. The resulting residue was purified by flash column chromatography (Silicycle column, 50 g, 0–50% EtOAc/Hexanes, linear gradient for 20 min) to provide *tert*-Butyl (2-(2-((6-chlorohexyl)oxy)ethoxy)ethyl) carbamate (1.50 g, 38%) as a color less oil. ^1H NMR (500 MHz, $CDCl_3$) δ 4.99 (br s, 1 H), 3.62–3.58 (m, 2 H), 3.57–3.52 (m, 4 H), 3.53 (t, J = 7.0 Hz, 2 H, overlapping), 3.46 (t, J = 7.0 Hz, 2 H), 3.35–3.26 (m, 2 H), 1.81–1.73 (m, 2 H), 1.64–1.55 (m, 2 H), 1.50–1.41 (m, 2 H), 1.44 (s, 9 H, overlapping), 1.40–1.33 (m, 2 H) ppm; ^{13}C NMR (125 MHz, $CDCl_3$) δ 156.11, 79.30, 71.41, 70.41, 70.35, 70.17, 45.17, 40.48, 32.67, 29.57, 28.55, 26.82, 25.55 ppm; HRMS (ESI) *m/z*: $[M + H]^+$ calcd for $C_{15}H_{31}ClNO_4$, 324.1936; found 324.1930.

2-(2-((6-Chlorohexyl)oxy)ethoxy)ethan-1-amine (74).

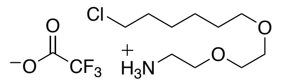

A solution of *tert*-Butyl (2-(2-((6-chlorohexyl)oxy)ethoxy)ethyl) carbamate (1.50 g, 4.63 mmol) in anhydrous DCM (30.0 mL) under argon atmosphere was cooled to 0 °C in an ice-water bath. After 10 min, TFA (5.0 mL) was added. The resulting reaction mixture was stirred at room temperature for 4 hours, and solvents were evaporated under reduced pressure. The resulting residue was purified by flash column chromatography (Silicycle column, 25 g, 0–15% MeOH/DCM, linear gradient for 20 min) to provide TFA salt of Amine (O2) HaloTag **74** (1.50 g, 100%) as a color less oil. ^1H NMR (500 MHz, $CDCl_3$) δ 8.10 (br s, 2 H), 3.75 (t, J = 4.5 Hz, 2 H), 3.68–3.64 (m, 2 H), 3.59–3.55 (m, 2 H), 3.53 (t, J = 7.0 Hz, 2 H), 3.46 (t, J = 7.0 Hz, 2 H), 3.16 (t, J = 4.5 Hz, 2 H), 1.81–1.73 (m, 2 H), 1.62–1.54 (m, 2 H), 1.49–1.41 (m, 2 H), 1.38–1.30 (m, 2 H) ppm; ^{13}C NMR (125 MHz, $CDCl_3$) δ 71.40, 70.50, 69.92, 66.73, 45.17, 39.77, 32.59, 29.37, 26.75, 25.41 ppm; ^{19}F

NMR (470 MHz, $CDCl_3$) δ −75.84 ppm; HRMS (ESI) *m/z*: $[M + H]^+$ calcd for $C_{10}H_{23}ClNO_2$, 224.1412; found 224.1408.

Synthesis of Si-Bridge amine (O2) HaloTags

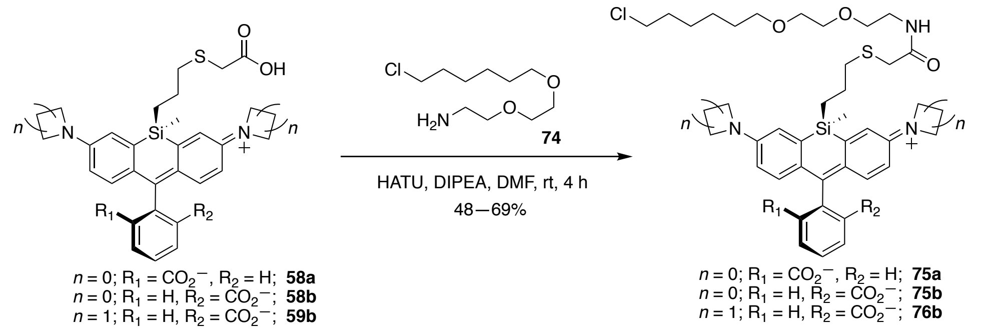

2-((3-((5*s*,10*s*)-3,7-Bis(dimethylamino)-5-methyl-3'-oxo-3'*H*,5*H*-spiro[dibenzo[*b*,*e*]siline-10,1'-isobenzofuran]-5-yl)propyl)thio)-*N*-(2-(2-((6-chlorohexyl)oxy)ethoxy)ethyl)acetamide (75a).

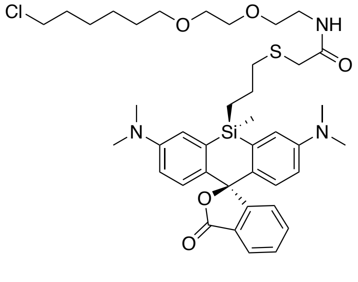

A solution of **58a** (60.0 mg, 0.11 mmol) in anhydrous DMF (2.0 mL) under argon atmosphere was combined with amine (O2) HaloTag ligand **74** (55.0 mg, 0.16 mmol), and treated with HATU (71.0 mg, 0.19 mmol) and DIPEA (96.0 μL, 0.55 mmol). After stirring the reaction at room temperature for 4 h, the reaction mixture was diluted with 0.25M HCl (10.0 mL) and extracted with EtOAc (2 x 20.0 mL). The combined extracts were washed with saturated $NaHCO_3$ (15.0 mL), saturated NaCl solution (15.0 mL), dried (Na_2SO_4), filtered, and evaporated under reduced pressure. The resulting residue was purified by flash column chromatography (Silicycle column, 10 g, 25–100% EtOAc/DCM, linear gradient for 20 min) to provide **75a** (40.0 mg, 48%) as a light green color solid. ^1H NMR (500 MHz,

CD_3OD) δ 7.95 (d, J = 7.5 Hz, 1 H), 7.75 (td, J = 7.5, 1.0 Hz, 1 H), 7.64 (td, J = 7.0, 0.5 Hz, 1 H), 7.27 (d, J = 7.5 Hz, 1 H), 7.01 (d, J = 3.0 Hz, 2 H), 6.68 (d, J = 8.5 Hz, 2 H), 6.62 (dd, J = 9.0, 3.0 Hz, 2 H), 3.53–3.45 (m, 6 H), 3.43 (t, J = 5.5 Hz, 2 H), 3.38 (t, J = 6.5 Hz, 2 H), 3.28 (t, J = 5.5 Hz, 2 H), 3.08 (s, 2 H), 2.96 (s, 12 H), 2.53 (t, J = 7.5 Hz, 2 H), 1.75–1.68 (m, 2 H), 1.67–1.61 (m, 2 H), 1.55–1.48 (m, 2 H), 1.45–1.37 (m, 2 H), 1.36–1.28 (m, 2 H), 1.22–1.16 (m, 2 H), 0.63 (s, 3 H) ppm; ^{13}C NMR (125 MHz, CD_3OD) δ 172.83, 172.61, 156.28, 151.03, 136.80, 135.50, 133.00, 130.27, 129.42, 127.95, 126.27, 125.87, 117.92, 114.83, 94.15, 72.17, 71.24, 71.15, 70.33, 45.72, 40.55, 36.99, 36.40, 33.74, 30.51, 27.73, 26.46, 25.29, 16.60, −3.07 ppm; HRMS (ESI) *m/z*: $[M + H]^+$ calcd for $C_{40}H_{55}ClN_3O_5SSi$, 752.3315; found 752.3299.

2-((3-((5*r*,10*r*)-3,7-Bis(dimethylamino)-5-methyl-3'-oxo-3'*H*,5*H*-spiro[dibenzo[*b*,*e*]siline-10,1'-isobenzofuran]-5-yl)propyl)thio)-*N*-(2-(2-((6-chlorohexyl)oxy)ethoxy)ethyl)acetamide (75b).

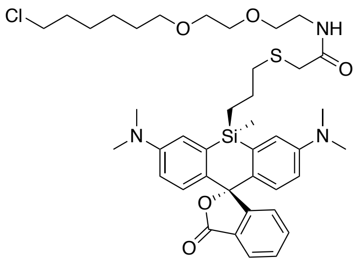

The same procedure was used as described above for compound **75a**. A solution of **58b** (55.0 mg, 0.10 mmol) in anhydrous DMF (2.0 mL) was combined with amine (O2) HaloTag ligand **74** (51.0 mg, 0.15 mmol), treated with HATU (66.0 mg, 0.17 mmol) and DIPEA (88.0 µL, 0.51 mmol) to provide **75b** (38.0 mg, 50%) as a light green color solid. ^{1}H NMR (500 MHz, CD_3OD) δ 7.95 (d, J = 7.5 Hz, 1 H), 7.76 (td, J = 7.5, 1.0 Hz, 1 H), 7.64 (td, J = 7.0, 0.5 Hz, 1 H), 7.28 (d, J = 7.5 Hz, 1 H), 7.02 (d, J = 2.5 Hz, 2 H), 6.69 (d, J = 9.0 Hz, 2 H), 6.64 (dd, J = 9.0, 2.5 Hz, 2 H), 3.53–3.46 (m, 6 H), 3.43 (t, J = 5.5 Hz, 2 H), 3.39 (t, J = 6.5 Hz, 2 H), 3.27 (t, J = 5.0 Hz, 2 H), 3.10 (s, 2 H), 2.97 (s, 12 H), 2.63 (t, J = 7.0 Hz, 2 H), 1.75–1.63 (m, 4 H), 1.56–1.49 (m, 2 H), 1.45–1.37 (m, 2 H), 1.36–1.28 (m, 4 H), 0.55 (s, 3 H) ppm; ^{13}C NMR (125 MHz, CD_3OD) δ 172.96, 172.54, 156.38, 151.02, 136.29, 135.62, 132.94, 130.25, 129.66, 127.91, 126.29, 125.77, 117.69, 114.92,

94.19, 72.20, 71.22, 71.18, 70.35, 45.70, 40.52, 37.15, 36.38, 33.72, 30.52, 27.72, 26.47, 25.41, 14.94, −1.13 ppm; HRMS (ESI) *m/z*: $[M + H]^+$ calcd for $C_{40}H_{55}ClN_3O_5SSi$, 752.3315; found 752.3300.

***N*-(2-(2-((6-Chlorohexyl)oxy)ethoxy)ethyl)-2-((3-((5*r*,10*r*)-3,7-di(azetidin-1-yl)-5-methyl-3'-oxo-3'*H*,5*H*-spiro[dibenzo[*b*,*e*]siline-10,1'-isobenzofuran]-5-yl)propyl)thio)acetamide (76b).**

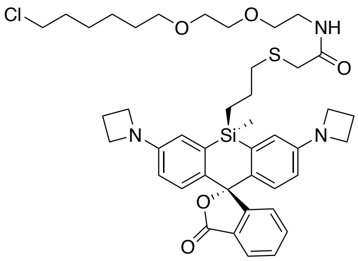

The same procedure was used as described above for compound **75a**. A solution of **59b** (32.0 mg, 0.056 mmol) in anhydrous DMF (1.0 mL) was combined with amine (O2) HaloTag ligand **74** (29.0 mg, 0.084 mmol), treated with HATU (37.0 mg, 0.095 mmol) and DIPEA (50.0 µL, 0.28 mmol) to provide **76b** (30.0 mg, 69%) as a light green color solid. ^{1}H NMR (500 MHz, $CDCl_3$) δ 7.96 (d, J = 7.5 Hz, 1 H), 7.63 (t, J = 7.5 Hz, 1 H), 7.53 (t, J = 7.0 Hz, 1 H), 7.23 (d, J = 8.0 Hz, 1 H), 7.12 (t, J = 5.5 Hz, 1 H), 6.74 (d, J = 8.5 Hz, 2 H), 6.61 (d, J = 2.5 Hz, 2 H), 6.27 (dd, J = 8.5, 2.5 Hz, 2 H), 3.90 (t, J = 7.0 Hz, 8 H), 3.59–3.50 (m, 8 H), 3.48–3.42 (m, 4 H), 3.17 (s, 2 H), 2.58 (t, J = 7.0 Hz, 2 H), 2.37 (p, J = 7.0 Hz, 4 H), 1.80–1.73 (m, 2 H), 1.72–1.65 (m, 2 H), 1.62–1.55 (m, 2 H), 1.49–1.41 (m, 2 H), 1.39–1.33 (m, 2 H), 1.23–1.18 (m, 2 H), 0.56 (s, 3 H) ppm; ^{13}C NMR (125 MHz, $CDCl_3$) δ 170.80, 168.96, 150.95, 134.95, 133.99, 132.92, 128.82, 128.74, 126.95, 125.81, 124.51, 115.40, 112.71, 92.00, 71.43, 70.58, 70.17, 69.85, 52.38, 45.18, 39.58, 38.75, 36.58, 36.19, 32.65, 29.61, 26.82, 25.56, 24.08, 17.04, 14.56, −1.33 ppm; HRMS (ESI) *m/z*: $[M + H]^+$ calcd for $C_{42}H_{55}ClN_3O_5SSi$, 776.3315; found 776.3328.

Synthesis of Si-Bridge SNAP-Tags

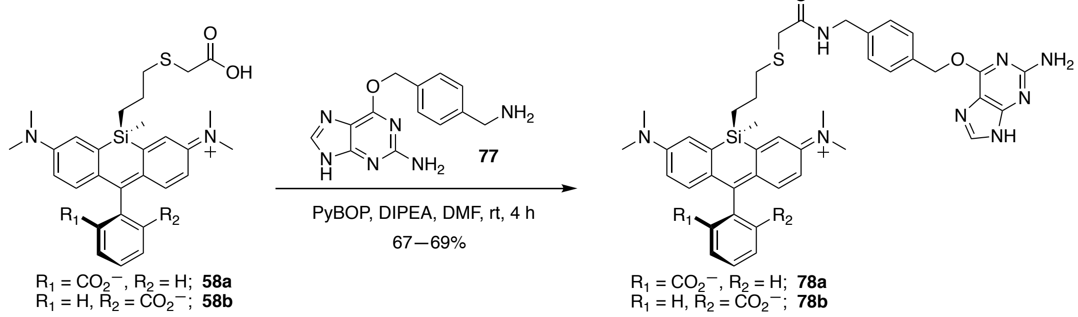

***N*-(4-(((2-Amino-9*H*-purin-6-yl)oxy)methyl)benzyl)-2-((3-((5*s*,10*s*)-3,7-bis(dimethylamino)-5-methyl-3'-oxo-3'*H*,5*H*-spiro[dibenzo[*b*,*e*]siline-10,1'-isobenzofuran]-5-yl)propyl)thio)acetamide (78a).**

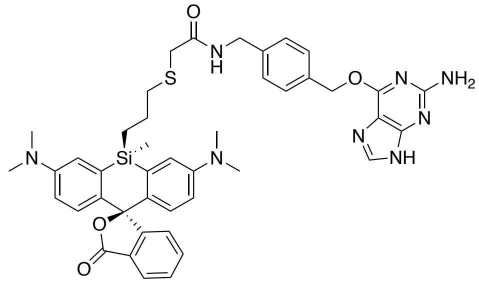

A solution of **58a** (50.0 mg, 0.092 mmol) in anhydrous DMF (1.5 mL) under argon atmosphere was combined with SNAP-Tag ligand **77** (37.0 mg, 0.14 mmol), and treated with PyBOP (71.0 mg, 0.14 mmol) and DIPEA (80.0 µL, 0.46 mmol). After stirring the reaction at room temperature for 4 h, solvent was evaporated under reduced pressure. The resulting residue was purified by flash column chromatography (Silicycle column, 10 g, 0–15% MeOH in 0.1% v/v TEA/DCM, linear gradient for 20 min) to provide **78a** (49.0 mg, 67%) as a light green color solid. ^{1}H NMR (500 MHz, $CDCl_3$) δ 8.08 (t, J = 5.0 Hz, 1 H), 7.95 (d, J = 7.5 Hz, 1 H), 7.68 (t, J = 7.5 Hz, 1 H), 7.57 (t, J = 7.5 Hz, 1 H), 7.33 (d, J = 7.5 Hz, 1 H), 7.28 (d, J = 8.0 Hz, 2 H), 7.21 (d, J = 8.0 Hz, 2 H), 6.93 (d, J = 3.0 Hz, 2 H), 6.68 (d, J = 8.5 Hz, 2 H), 6.50 (dd, J = 9.0, 3.0 Hz, 2 H), 5.31 (s, 2 H), 4.38 (d, J = 5.5 Hz, 2 H), 3.31 (s, 2 H), 2.95 (s, 12 H), 2.50 (t, J = 7.0 Hz, 2 H), 1.75–1.66 (m, 2 H), 1.19–1.13 (m, 2 H), 0.62 (s, 3 H) ppm; ^{13}C NMR (125 MHz, $CDCl_3$) δ 170.97, 170.35, 159.21, 154.09,

149.45, 139.12, 136.41, 134.92, 133.95, 131.84, 129.68, 129.07, 128.71, 128.10, 127.70, 125.68, 125.10, 116.85, 113.32, 92.91, 68.70, 43.87, 40.34, 36.11, 36.01, 24.22, 16.00, −3.39 ppm; HRMS (ESI) *m/z*: $[M + H]^+$ calcd for $C_{43}H_{47}N_8O_4SSi$, 799.3205; found 799.3216.

***N*-(4-(((2-Amino-9*H*-purin-6-yl)oxy)methyl)benzyl)-2-((3-((5*r*,10*r*)-3,7-bis(dimethylamino)-5-methyl-3'-oxo-3'*H*,5*H*-spiro[dibenzo[*b*,*e*]siline-10,1'-isobenzofuran]-5-yl)propyl)thio)acetamide (78b).**

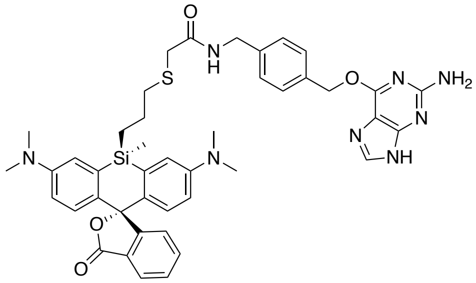

The same procedure was used as described above for compound **78a**. A solution of **58b** (50.0 mg, 0.092 mmol) in anhydrous DMF (1.5 mL) was combined with SNAP-Tag ligand **77** (37.0 mg, 0.14 mmol), treated with PyBOP (71.0 mg, 0.14 mmol) and DIPEA (80.0 μL, 0.46 mmol) to provide **78b** (51.0 mg, 69%) as a light green color solid. ^{1}H NMR (500 MHz, $CDCl_3$) δ 7.94 (d, J = 7.5 Hz, 1 H), 7.62 (t, J = 5.0 Hz, 1 H), 7.59 (t, J = 7.5 Hz, 1 H), 7.50 (t, J = 7.0 Hz, 1 H), 7.35 (d, J = 8.0 Hz, 2 H), 7.24 (d, J = 8.0 Hz, 2 H), 7.20 (d, J = 8.0 Hz, 1 H), 6.90 (d, J = 3.0 Hz, 2 H), 6.76 (d, J = 9.0 Hz, 2 H), 6.54 (dd, J = 8.5, 2.5 Hz, 2 H), 5.36 (s, 2 H), 4.41 (d, J = 5.5 Hz, 2 H), 3.29 (s, 2 H), 2.93 (s, 12 H), 2.65 (t, J = 7.0 Hz, 2 H), 1.79–1.71 (m, 2 H), 1.26–1.19 (m, 2 H), 0.54 (s, 3 H) ppm; ^{13}C NMR (125 MHz, $CDCl_3$) δ 171.06, 169.97, 159.20, 154.97, 149.37, 138.76, 135.10, 135.04, 134.11, 132.00, 129.63, 128.81, 127.89, 126.88, 125.68, 124.40, 116.44, 113.79, 92.19, 68.62, 43.77, 40.39, 36.84, 36.23, 24.26, 14.61, −1.30 ppm; HRMS (ESI) *m/z*: $[M + H]^+$ calcd for $C_{43}H_{47}N_8O_4SSi$, 799.3205; found 799.3209.

References

1. Hanwell, M.D.; Curtis, D.E.; Lonie, D.C.; Vandermeersch, T.; Zurek, E.; Hutchison, G.R. Avogadro: An advanced semantic chemical editor, visualization, and analysis platform. *J. Cheminform.* **2012**, *4*, 17.
2. Bucevičius, J.; Keller-Findeisen, J.; Gilat, T.; W. Hell, S.; Lukinavičius, G. Rhodamine–Hoechst Positional Isomers for Highly Efficient Staining of Heterochromatin. *Chemical Science* **2019**, *10* (7), 1962–1970.
3. Grimm, J. B.; Tkachuk, A. N.; Xie, L.; Choi, H.; Mohar, B.; Falco, N.; Schaefer, K.; Patel, R.; Zheng, Q.; Liu, Z.; Lippincott-Schwartz, J.; Brown, T. A.; Lavis, L. D. *Nat. Methods* **2020**, *17*, 815–821.
4. Zhou, X.; Lai, R.; Beck, J. R.; Li, H.; Stains, C. I. Nebraska Red: A Phosphinate-Based near-Infrared Fluorophore Scaffold for Chemical Biology Applications. *Chem. Commun.* **2016**, *52*, 12290–12293.
5. Koide, Y.; Urano, Y.; Hanaoka, K.; Piao, W.; Kusakabe, M.; Saito, N.; Terai, T.; Okabe, T.; Nagano, T. Development of NIR Fluorescent Dyes Based on Si–Rhodamine for in Vivo Imaging. *J. Am. Chem. Soc.* **2012**, *134*, 5029–5031
6. Grimm, J. B.; Brown, T. A.; Tkachuk, A. N.; Lavis, L. D. *ACS Central Science*. **2017**, *3*, 975–985.

Supporting Information – NMR Spectra

Si-Bridge Dyes: Silicon Substitution Expands the Repertoire of Si-Rhodamine Fluorescent Probes

Desaboini Nageswara Rao, Xincai Ji, and Stephen C. Miller*

Department of Biochemistry and Molecular Biotechnology, University of Massachusetts Chan Medical School, Worcester, Massachusetts 01605, United States

*Corresponding author e-mail: stephen.miller@umassmed.edu

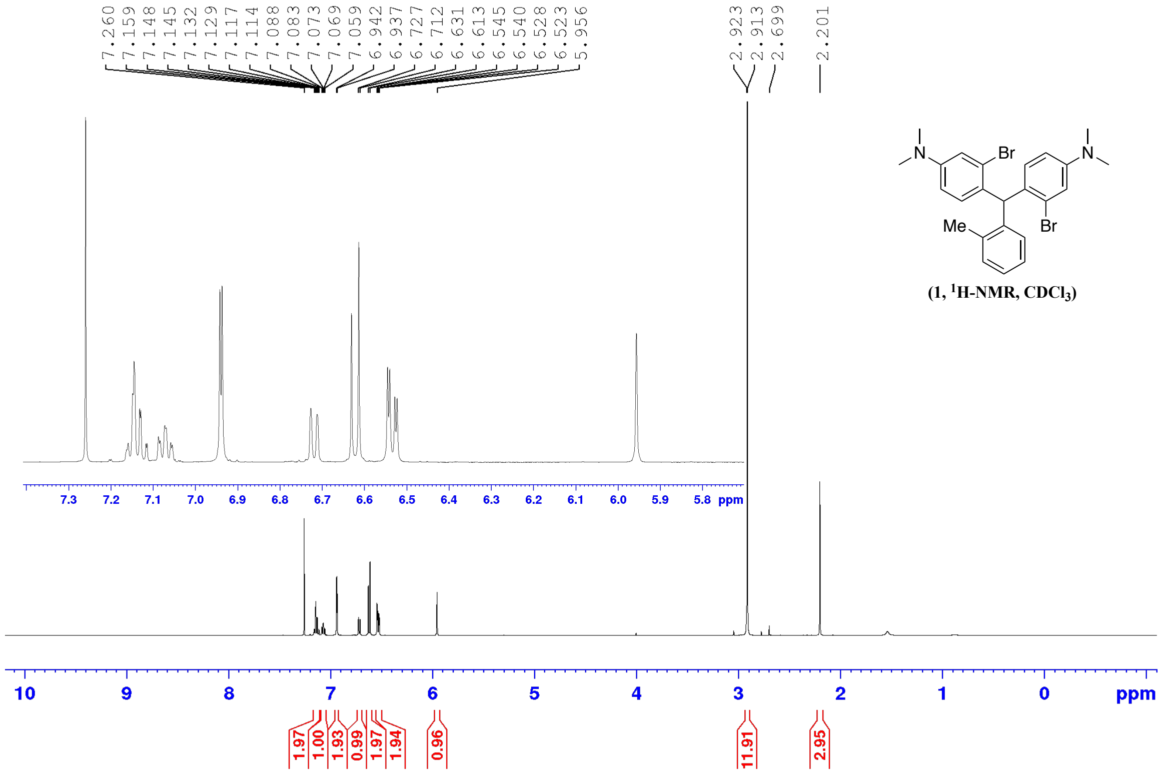
7.260 7.159 7.148 7.145 7.132 7.129 7.117 7.114 7.088 7.083 7.073 7.069 7.059 6.942 6.937 6.727 6.712 6.631 6.613 6.545 6.540 6.528 6.523 5.956
2.923 2.913 2.699 2.201
Br
N
Me
(1, ¹H-NMR, CDCl₃)
1.97 1.00 1.93 0.99 1.97 1.94 0.96 11.91 2.95
ppm

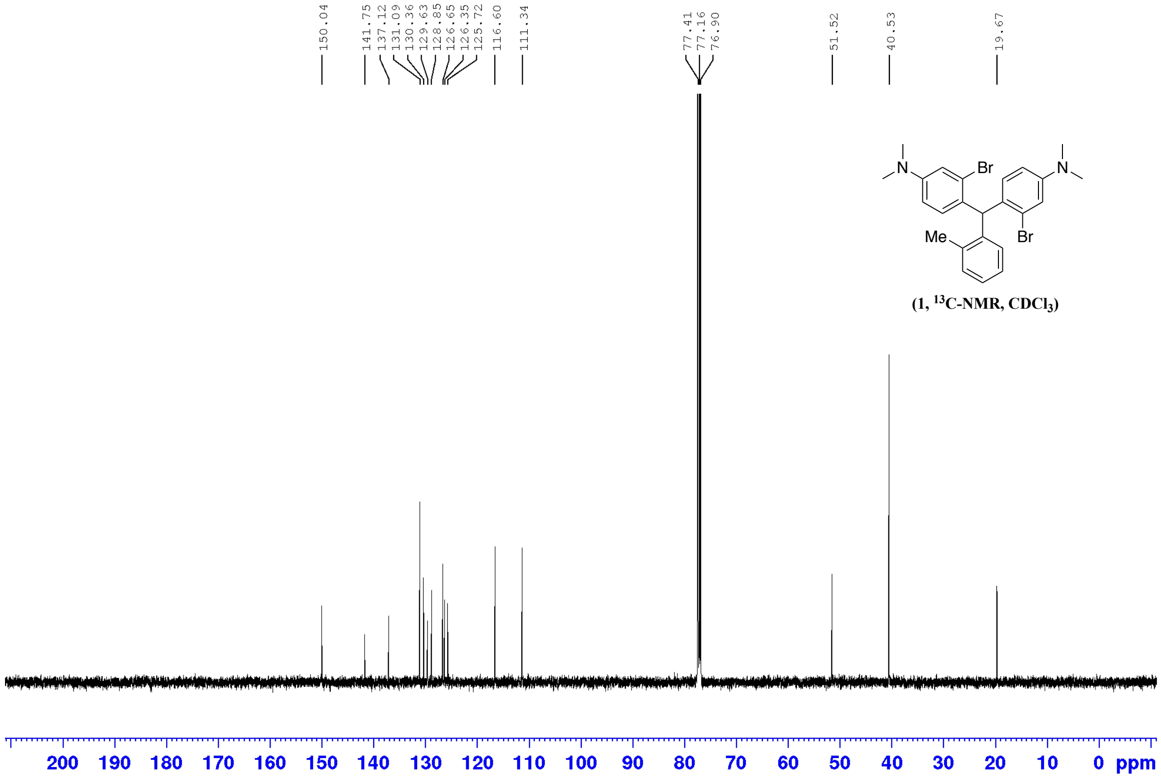
150.04 141.75 137.12 131.09 130.36 129.63 128.85 126.65 126.35 125.72 116.60 111.34
77.41 77.16 76.90 51.52 40.53 19.67
Br
N
Me
(1, ¹³C-NMR, CDCl₃)
ppm

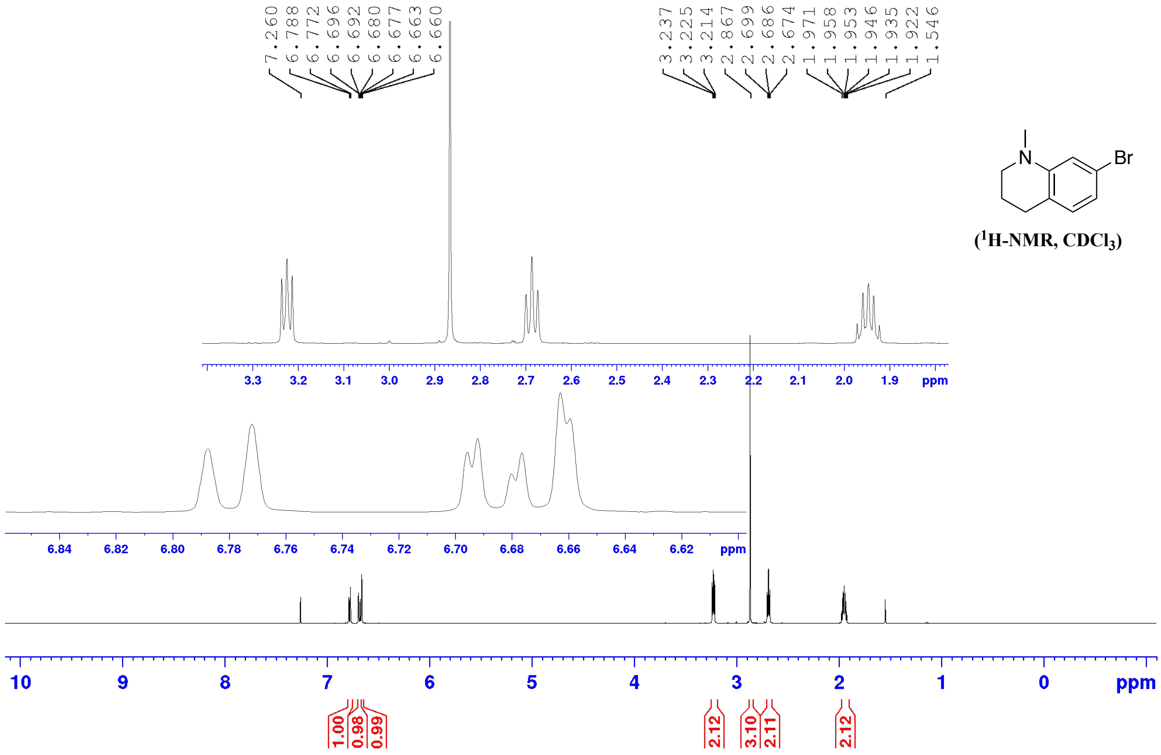
7.260
6.788
6.772
6.696
6.692
6.680
6.677
6.663
6.660
3.237
3.225
3.214
2.867
2.699
2.686
2.674
1.971
1.958
1.953
1.946
1.935
1.922
1.546
N
Br
(1H-NMR, CDCl3)
1.00
0.98
0.99
2.12
3.10
2.11
2.12
ppm

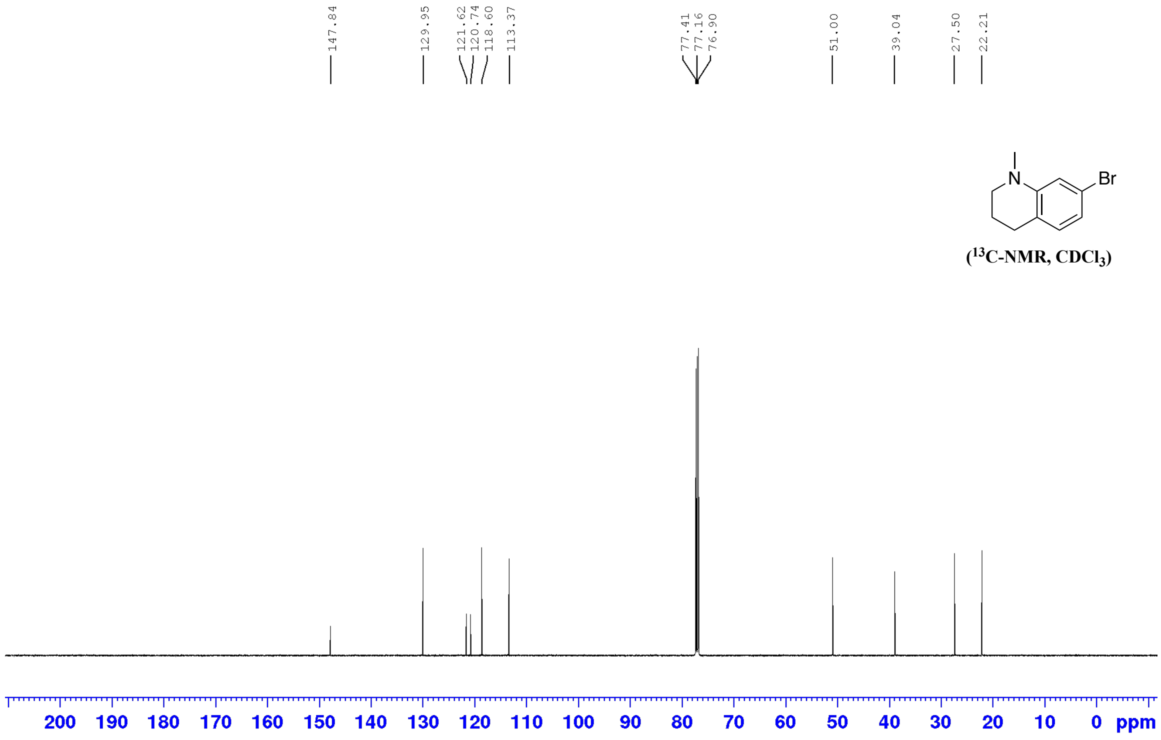
147.84
129.95
121.62
120.74
118.60
113.37
77.41
77.16
76.90
51.00
39.04
27.50
22.21
N
Br
(13C-NMR, CDCl3)
ppm

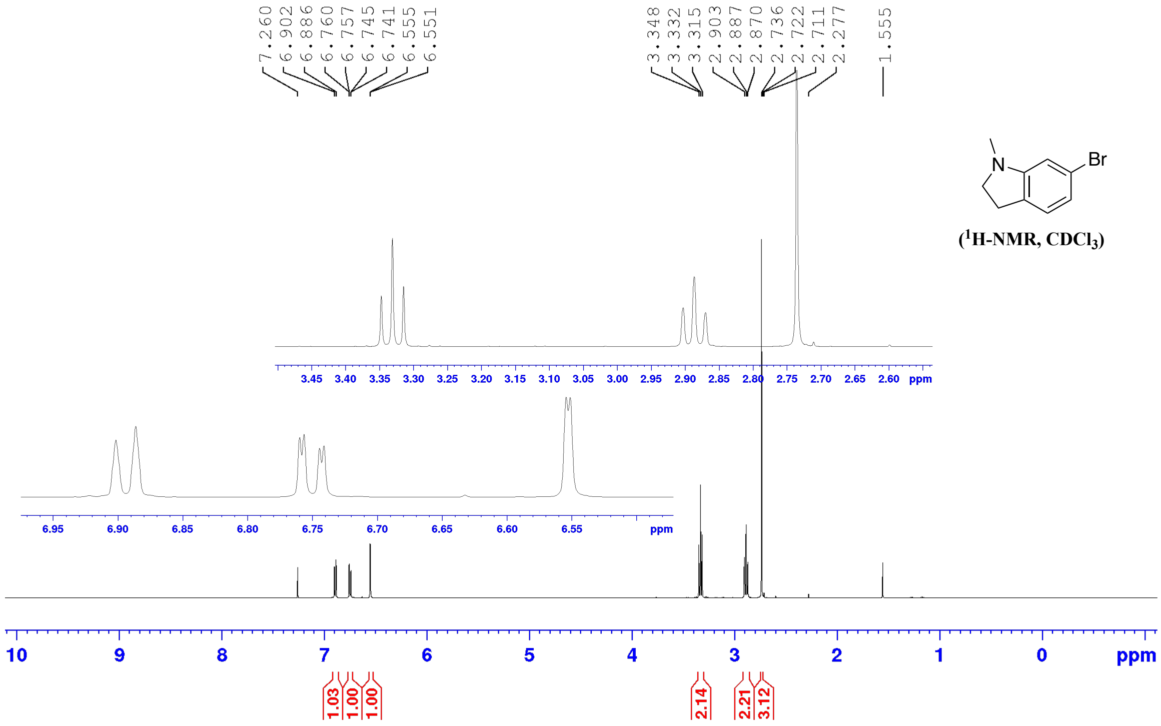
7.260
6.902
6.886
6.760
6.757
6.745
6.741
6.555
6.551
3.348
3.332
3.315
2.903
2.887
2.870
2.736
2.722
2.711
2.277
1.555
N
Br
(^{1}H-NMR, CDCl$_3$)
1.03
1.00
1.00
2.14
2.21
3.12
ppm

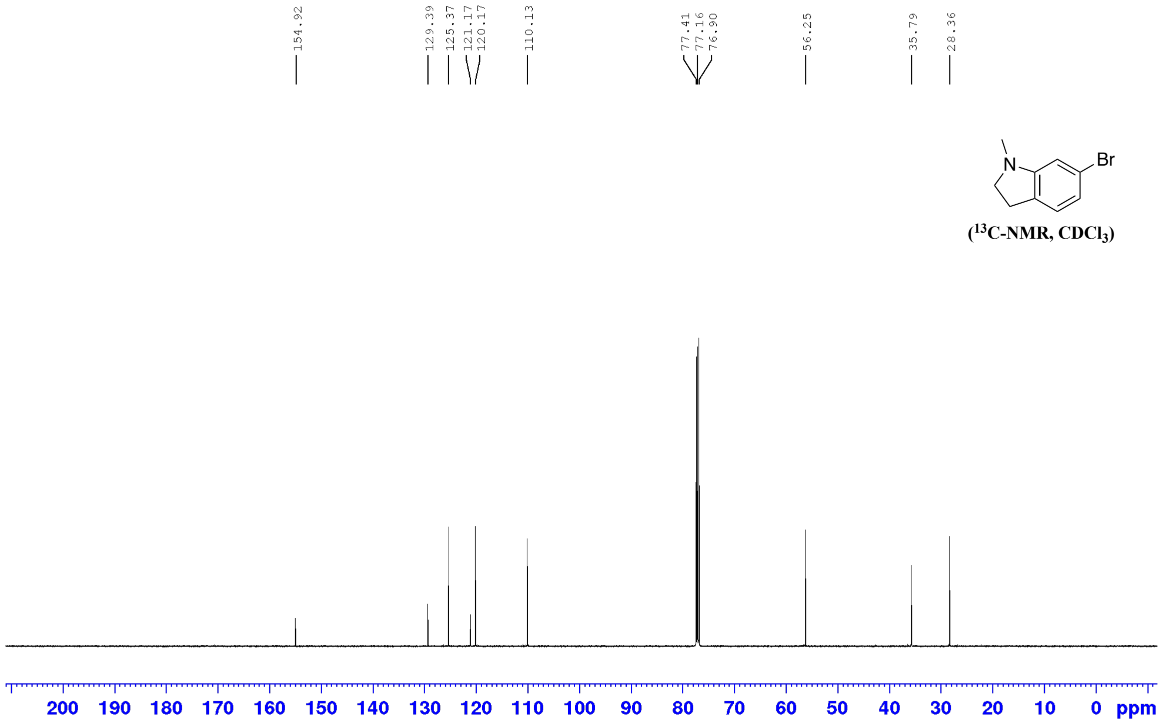
154.92
129.39
125.37
121.17
120.17
110.13
77.41
77.16
76.90
56.25
35.79
28.36
N
Br
(^{13}C-NMR, CDCl$_3$)
ppm

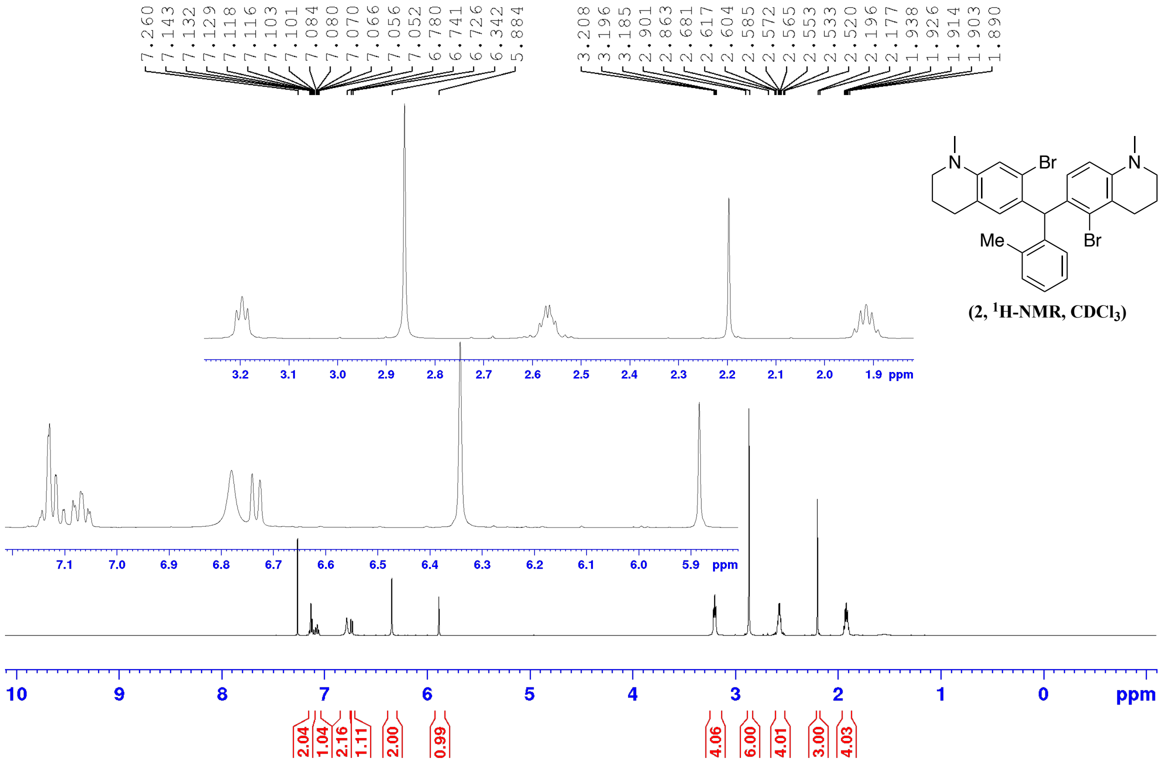

(2, ^{1}H-NMR, $CDCl_3$)

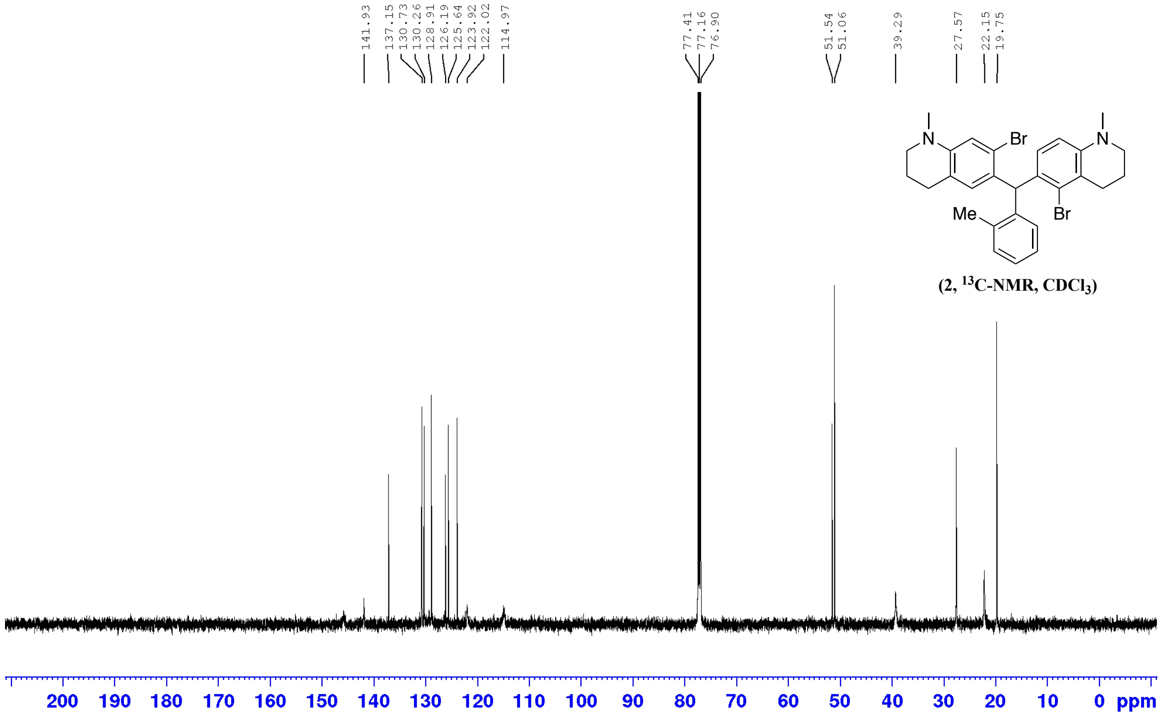

(2, ^{13}C-NMR, $CDCl_3$)

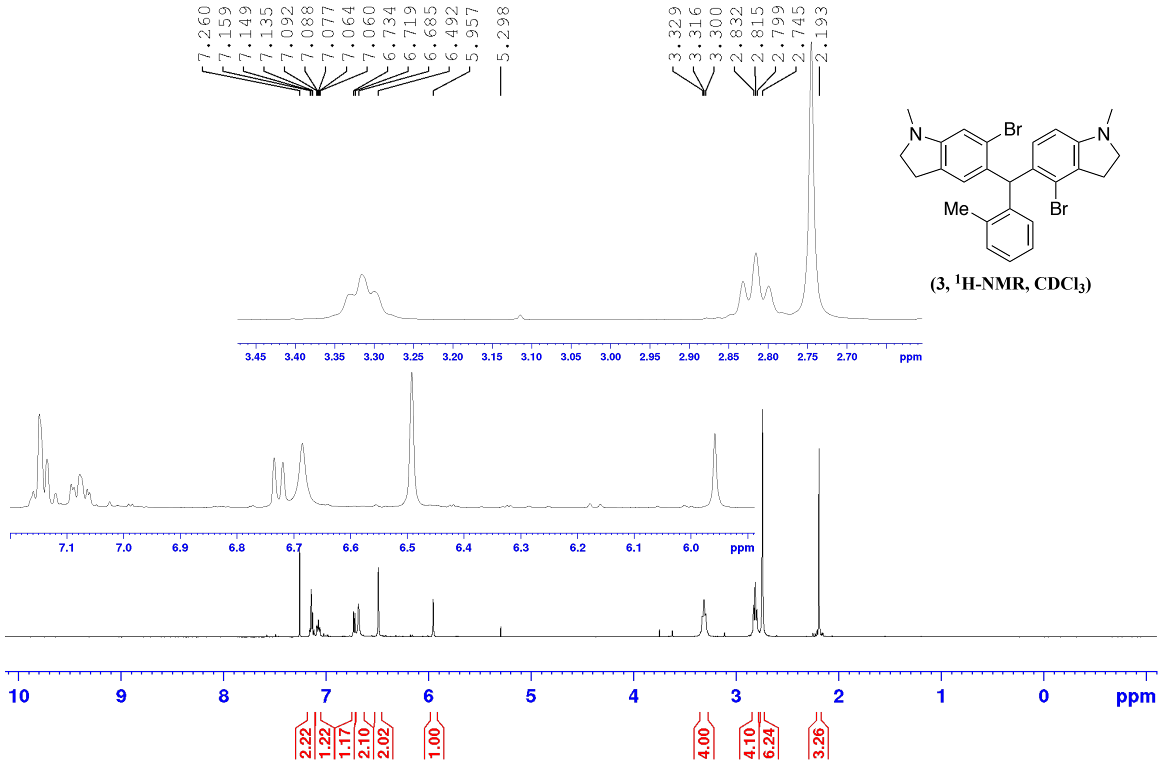

(3, ^{1}H-NMR, $CDCl_3$)

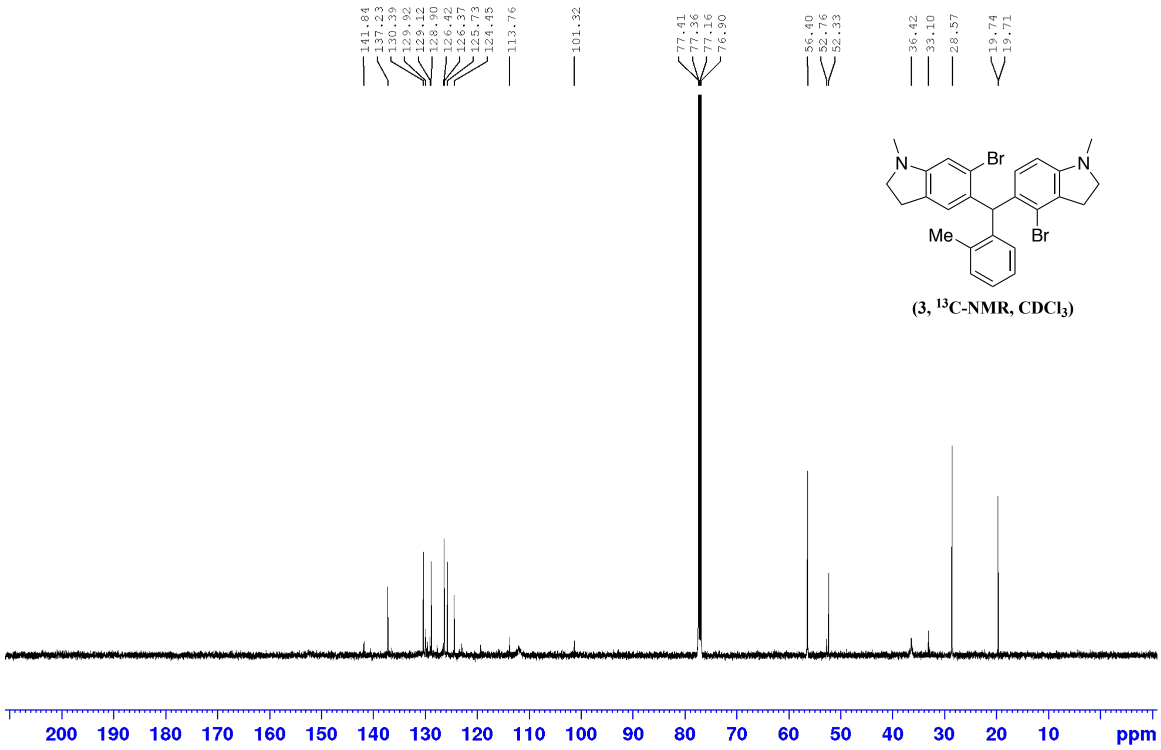

(3, ^{13}C-NMR, $CDCl_3$)

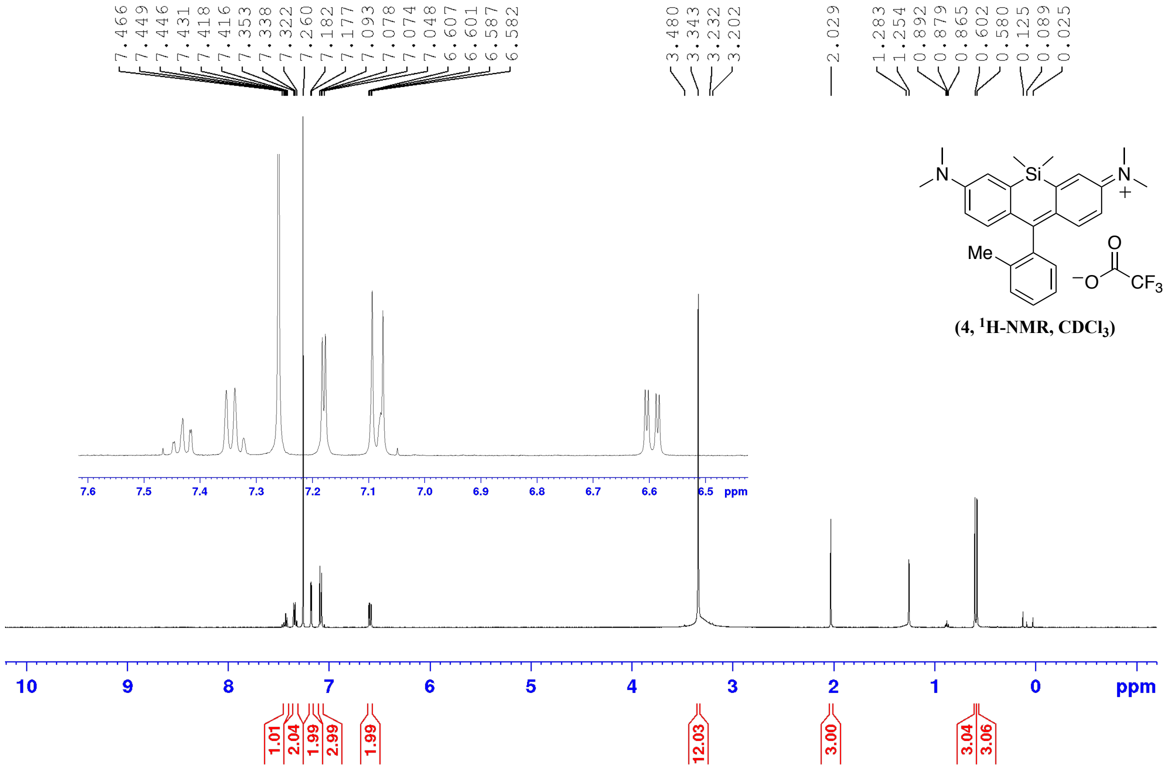
7.466
7.449
7.446
7.431
7.418
7.416
7.353
7.338
7.322
7.260
7.182
7.177
7.093
7.078
7.074
7.048
6.607
6.601
6.587
6.582
3.480
3.343
3.232
3.202
2.029
1.283
1.254
0.892
0.879
0.865
0.602
0.580
0.125
0.089
0.025
Si
N
N
+
Me
O
−O
CF3
(4, 1H-NMR, CDCl3)
1.01
2.04
1.99
2.99
1.99
12.03
3.00
3.04
3.06
ppm

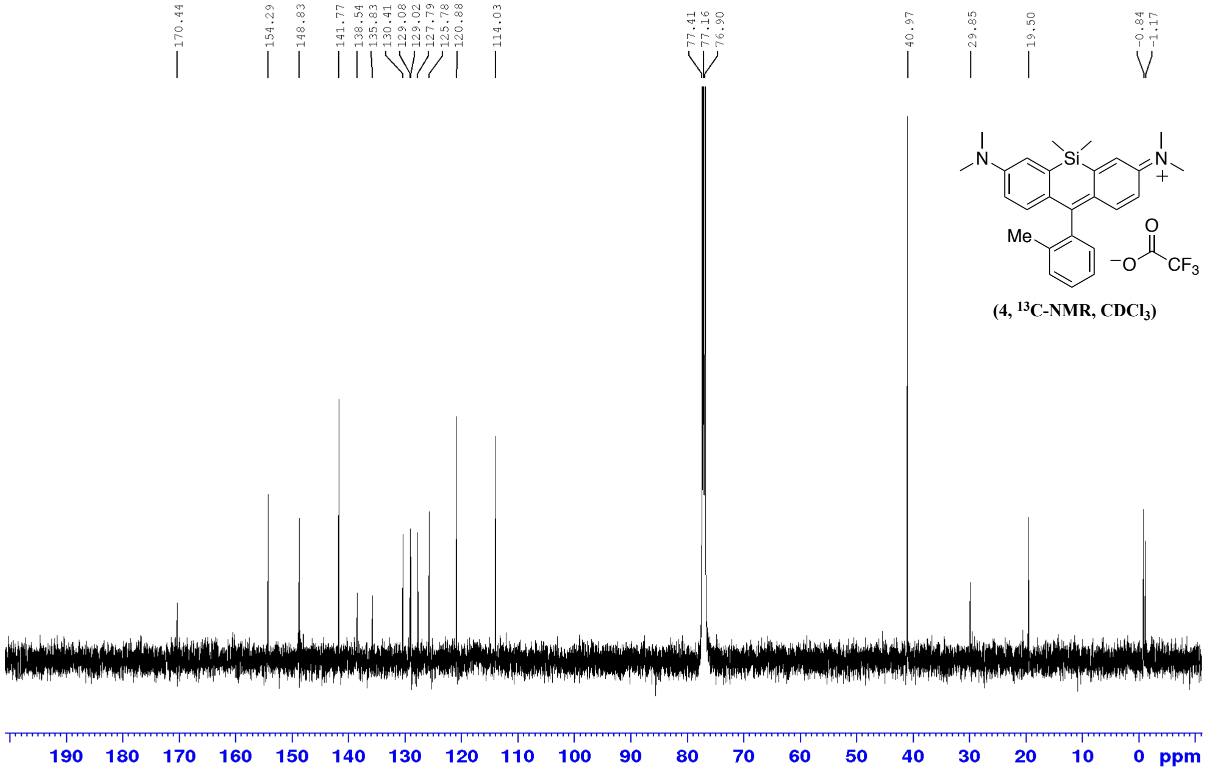
170.44
154.29
148.83
141.77
138.54
135.83
130.41
129.08
129.02
127.79
125.78
120.88
114.03
77.41
77.16
76.90
40.97
29.85
19.50
-0.84
-1.17
Si
N
N
+
Me
O
−O
CF3
(4, 13C-NMR, CDCl3)
ppm

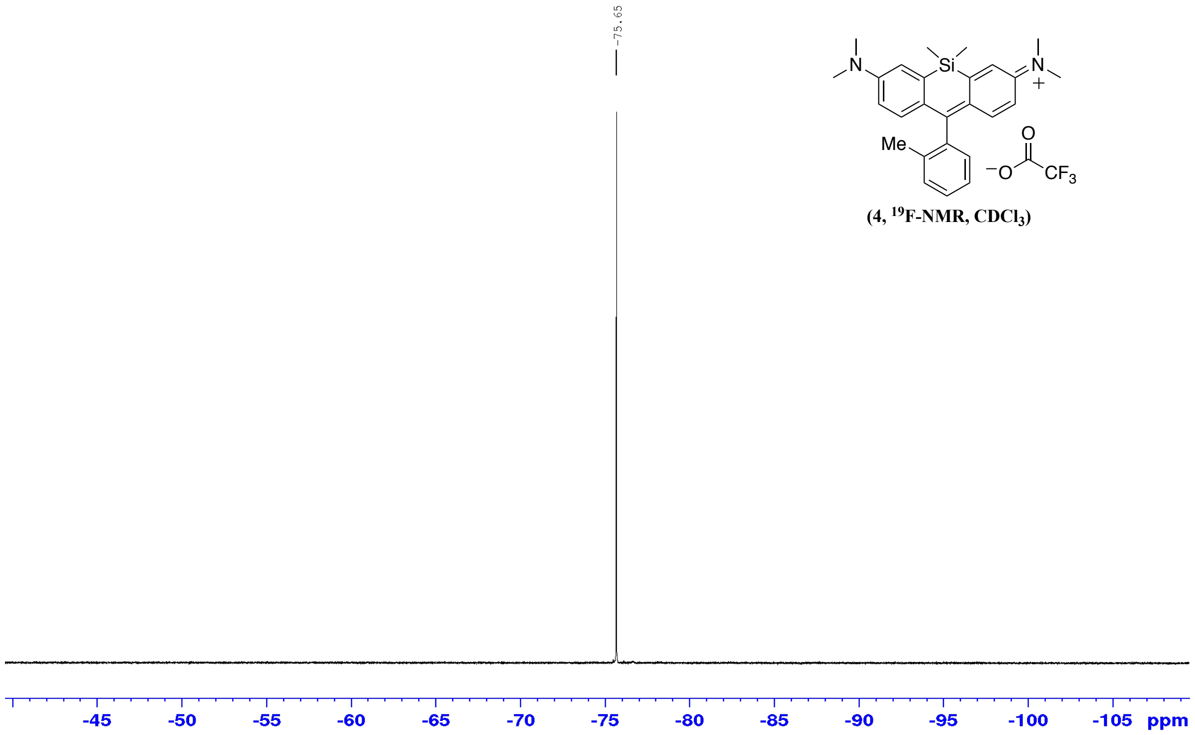

(4, ¹⁹F-NMR, CDCl$_3$)

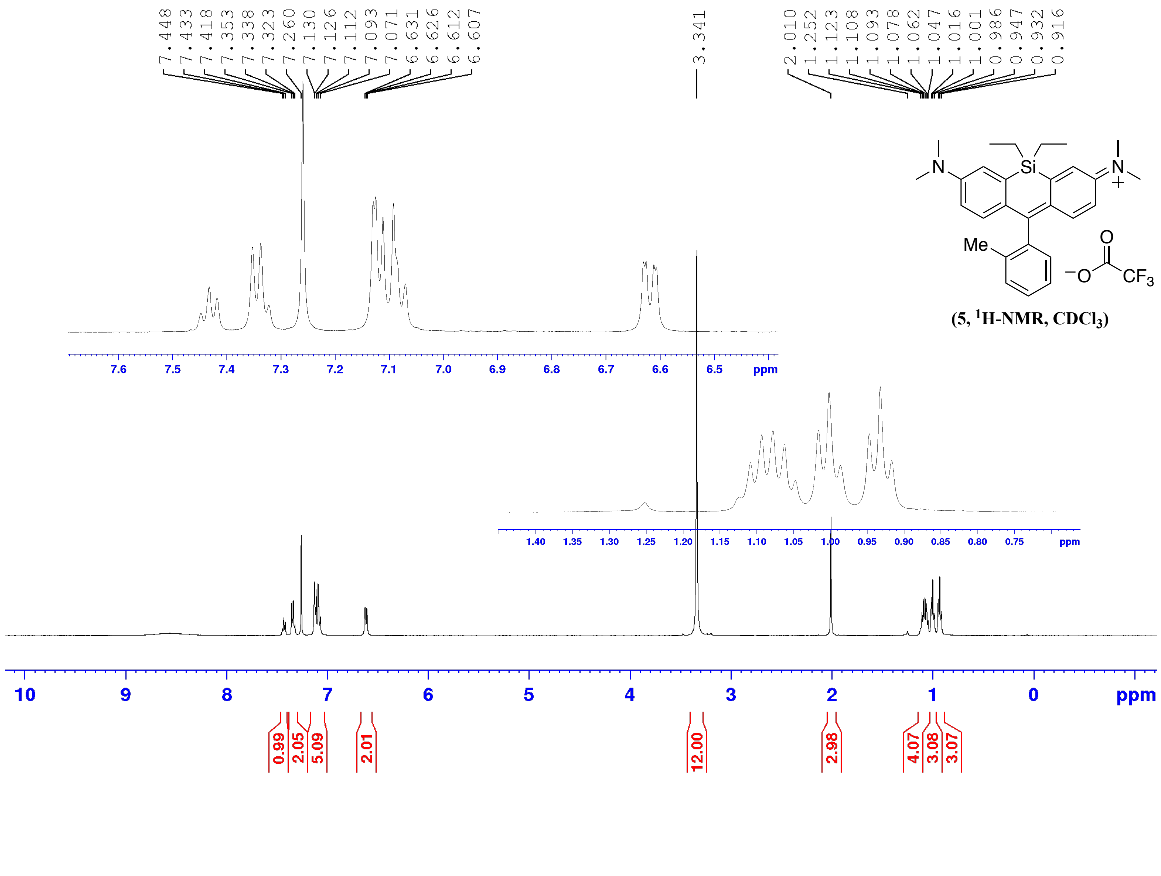

(5, ¹H-NMR, CDCl$_3$)

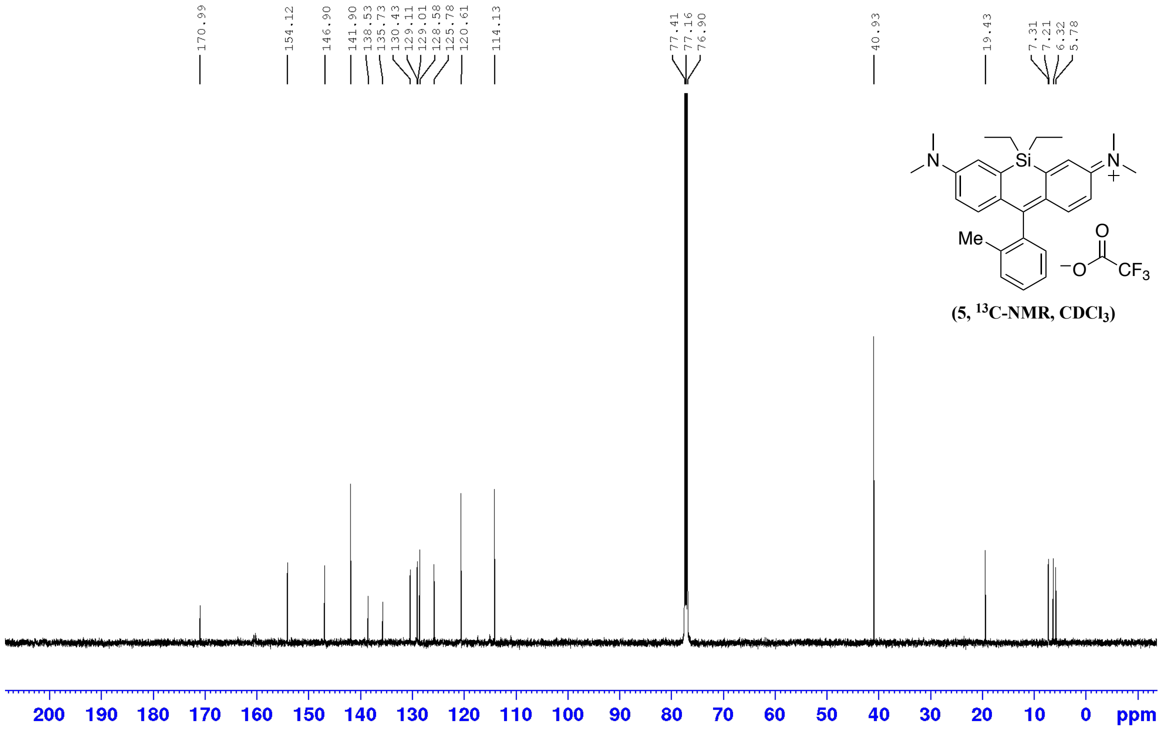

(5, ^{13}C-NMR, $CDCl_3$)

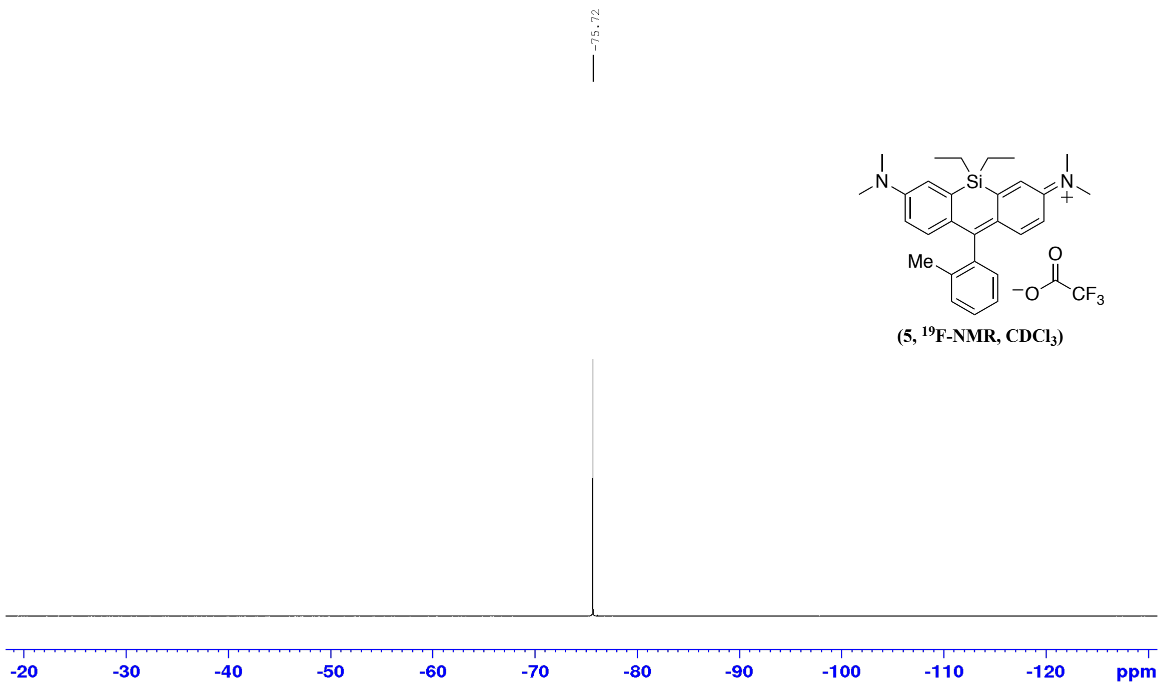

(5, ^{19}F-NMR, $CDCl_3$)

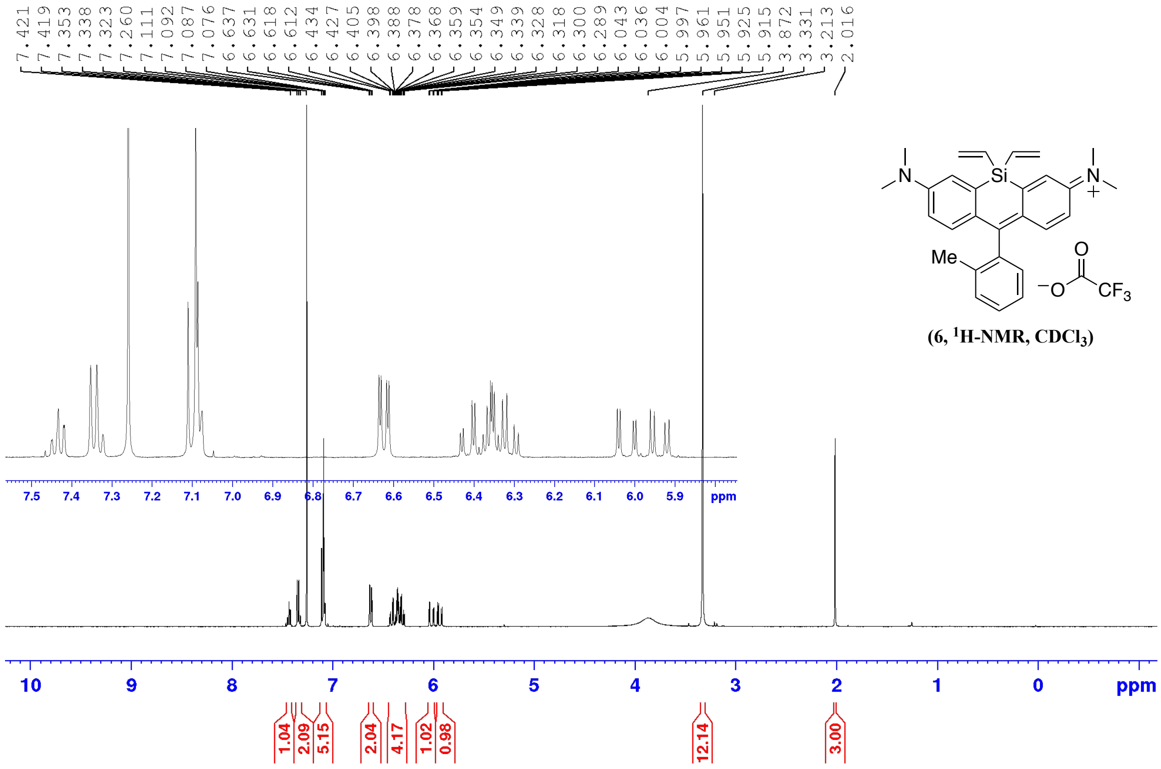

(6, ^{1}H-NMR, $CDCl_3$)

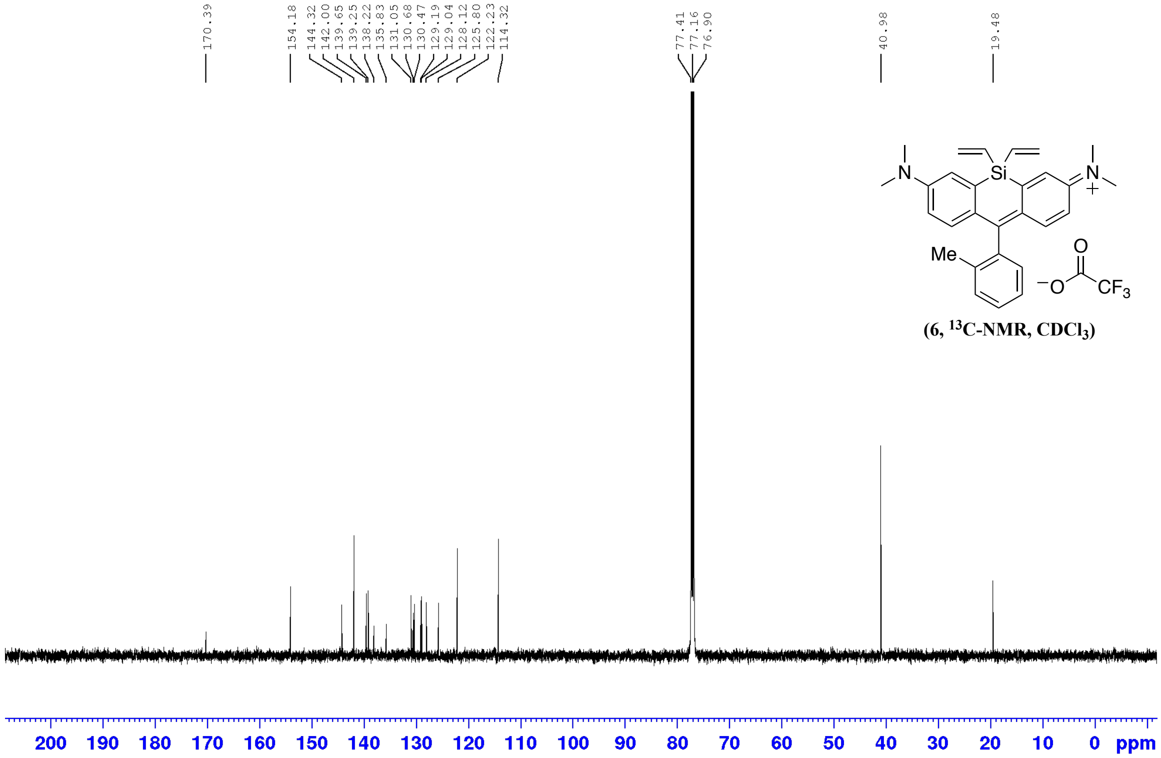

(6, ^{13}C-NMR, $CDCl_3$)

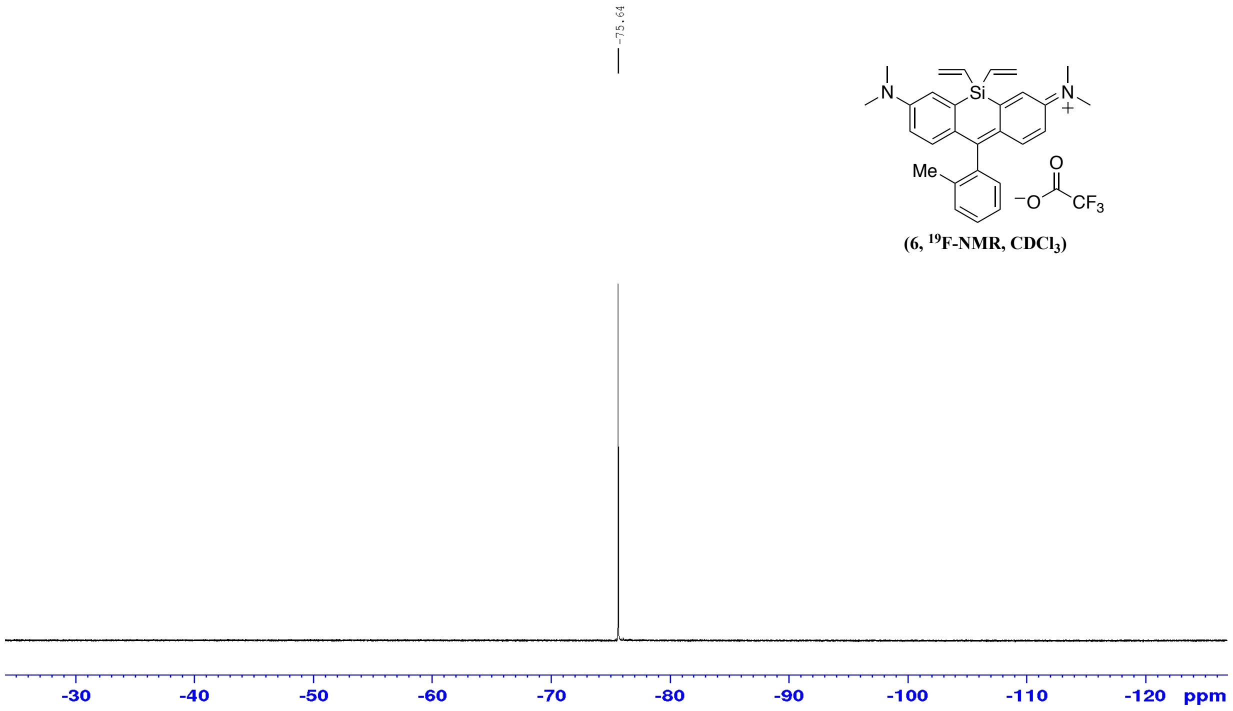

(6, ^{19}F-NMR, $CDCl_3$)

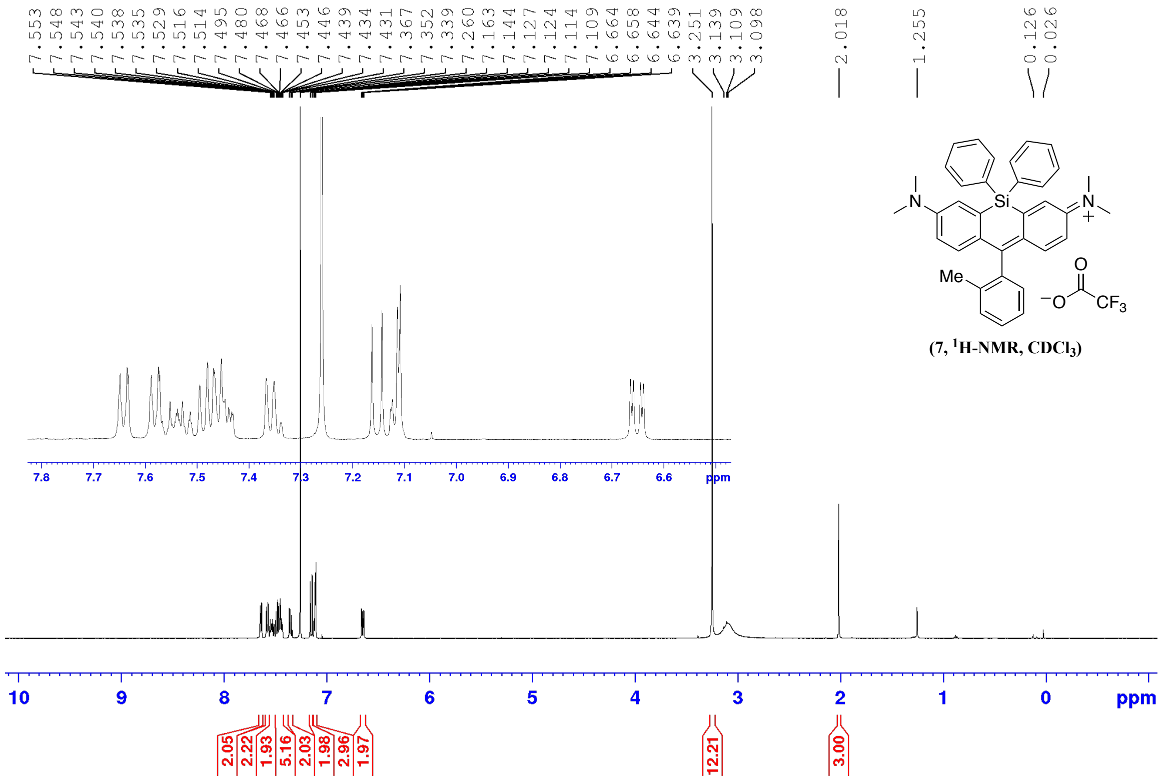

(7, ^{1}H-NMR, $CDCl_3$)

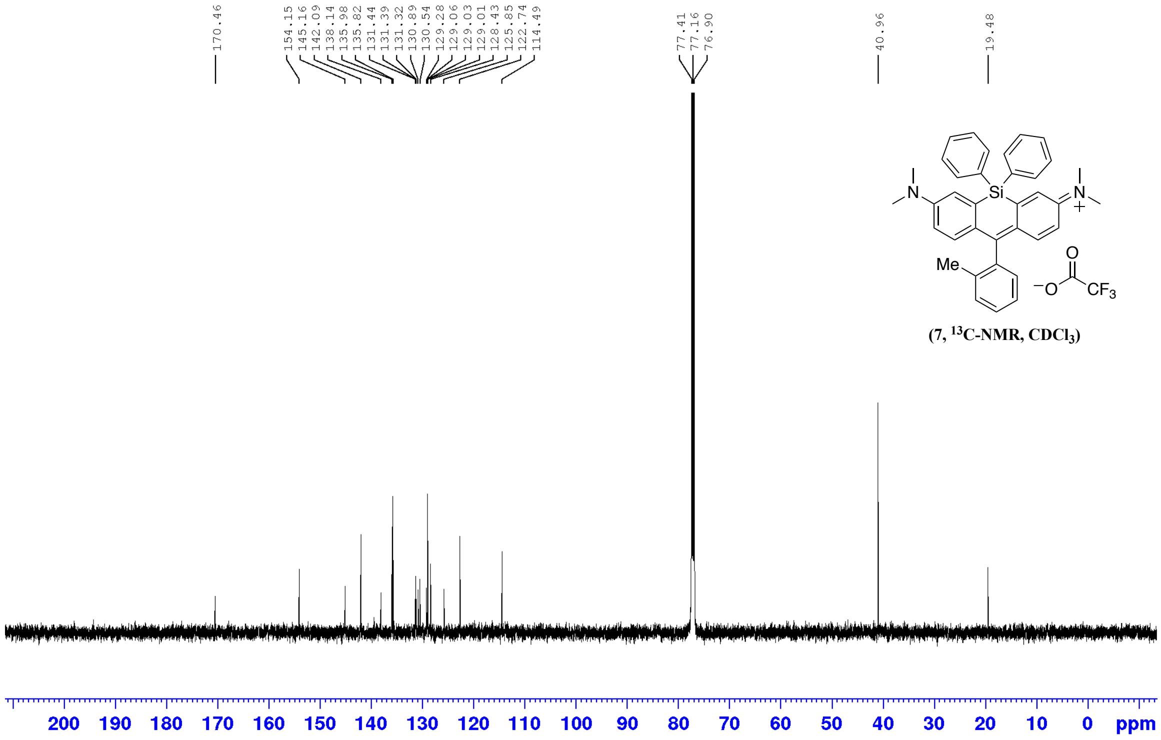
170.46
154.15
145.16
142.09
138.14
135.98
135.82
131.44
131.39
131.32
130.89
130.54
129.28
129.06
129.03
129.01
128.43
125.85
122.74
114.49
77.41
77.16
76.90
40.96
19.48
Si
N
N
+
Me
O
−O
CF3
(7, 13C-NMR, CDCl3)
200 190 180 170 160 150 140 130 120 110 100 90 80 70 60 50 40 30 20 10 0 ppm

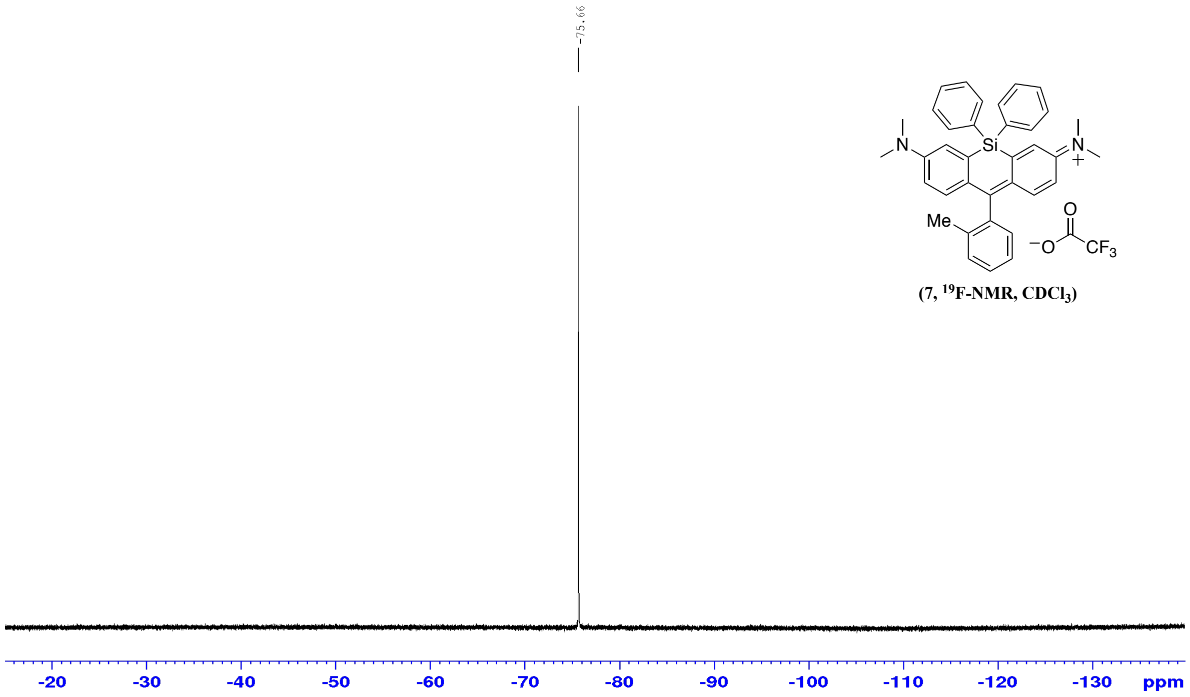
-75.66
Si
N
N
+
Me
O
−O
CF3
(7, 19F-NMR, CDCl3)
-20 -30 -40 -50 -60 -70 -80 -90 -100 -110 -120 -130 ppm

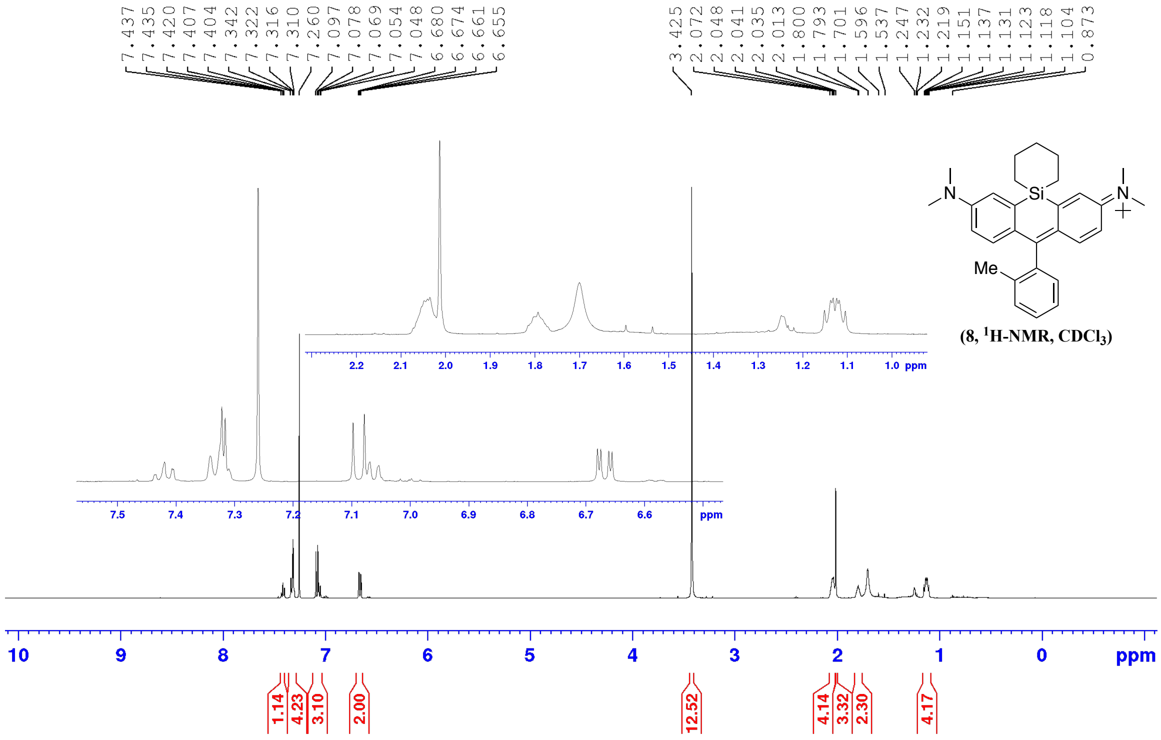

7.437
7.435
7.420
7.407
7.404
7.342
7.322
7.316
7.310
7.260
7.097
7.078
7.069
7.054
7.048
6.680
6.674
6.661
6.655
3.425
2.072
2.048
2.041
2.035
2.013
1.800
1.793
1.701
1.596
1.537
1.247
1.232
1.219
1.151
1.137
1.131
1.123
1.118
1.104
0.873
Si
N
Me
(8, 1H-NMR, CDCl3)
ppm
1.14
4.23
3.10
2.00
12.52
4.14
3.32
2.30
4.17

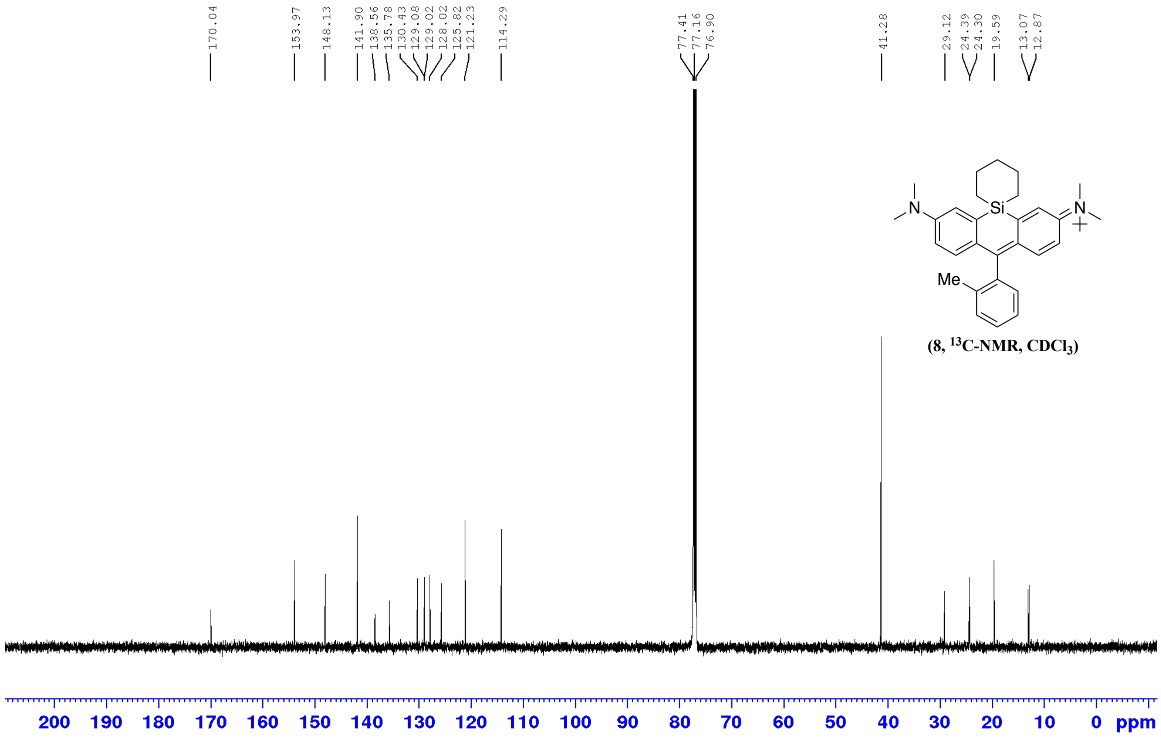

170.04
153.97
148.13
141.90
138.56
135.78
130.43
129.08
129.02
128.02
125.82
121.23
114.29
77.41
77.16
76.90
41.28
29.12
24.39
24.30
19.59
13.07
12.87
Si
N
Me
(8, 13C-NMR, CDCl3)
ppm

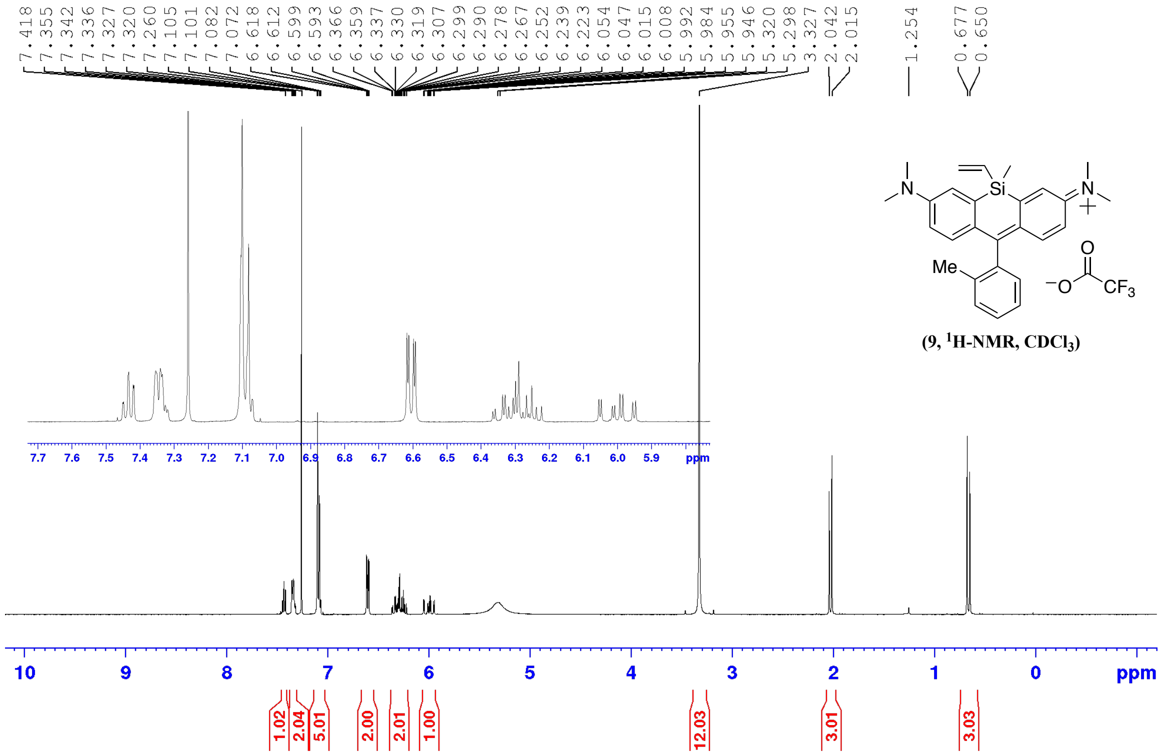

(9, ^{1}H-NMR, $CDCl_3$)

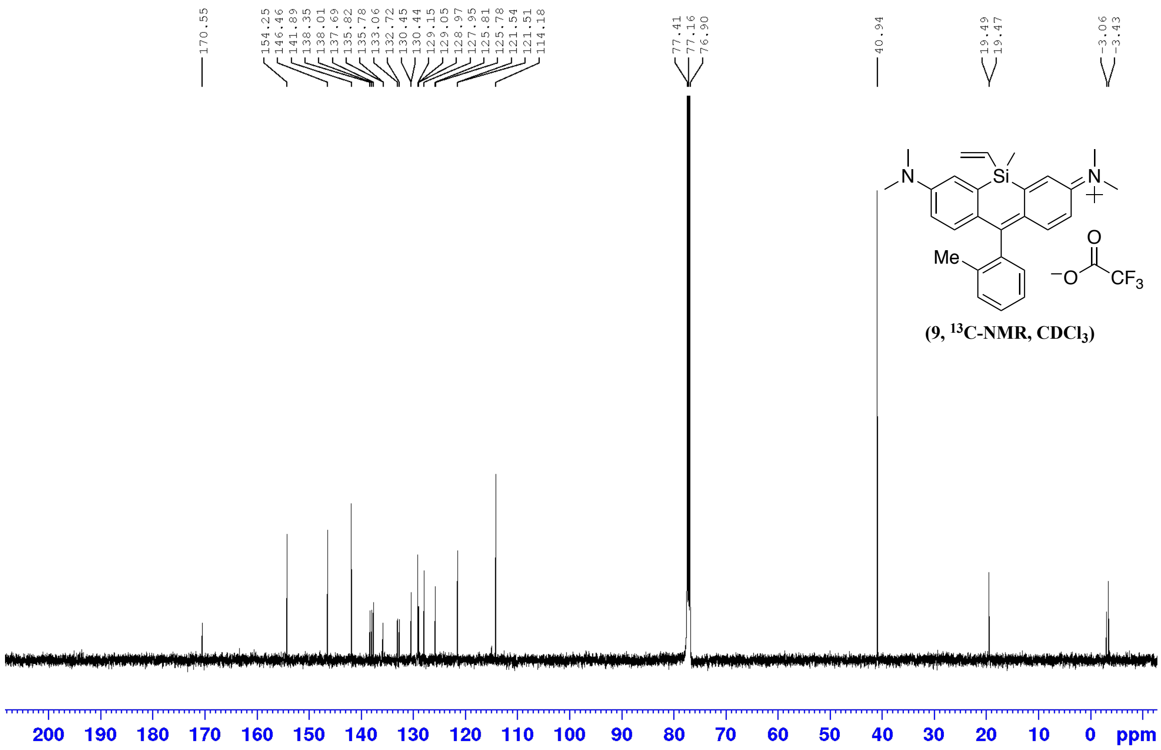

(9, ^{13}C-NMR, $CDCl_3$)

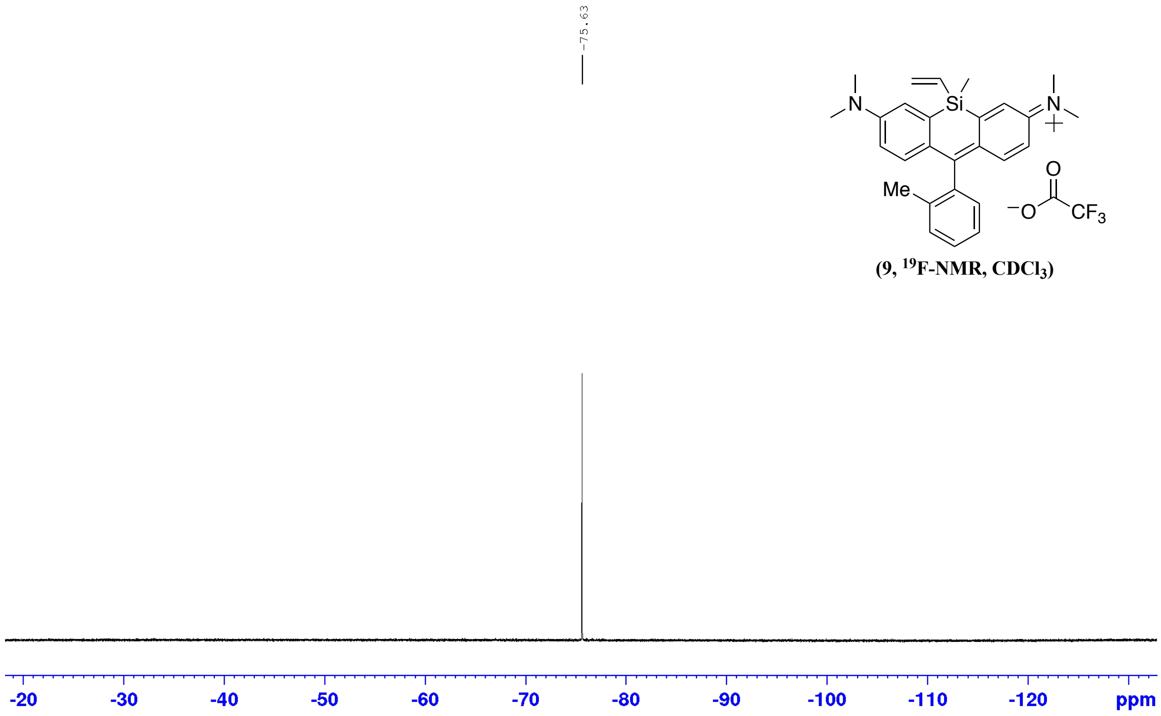

(9, ^{19}F-NMR, $CDCl_3$)

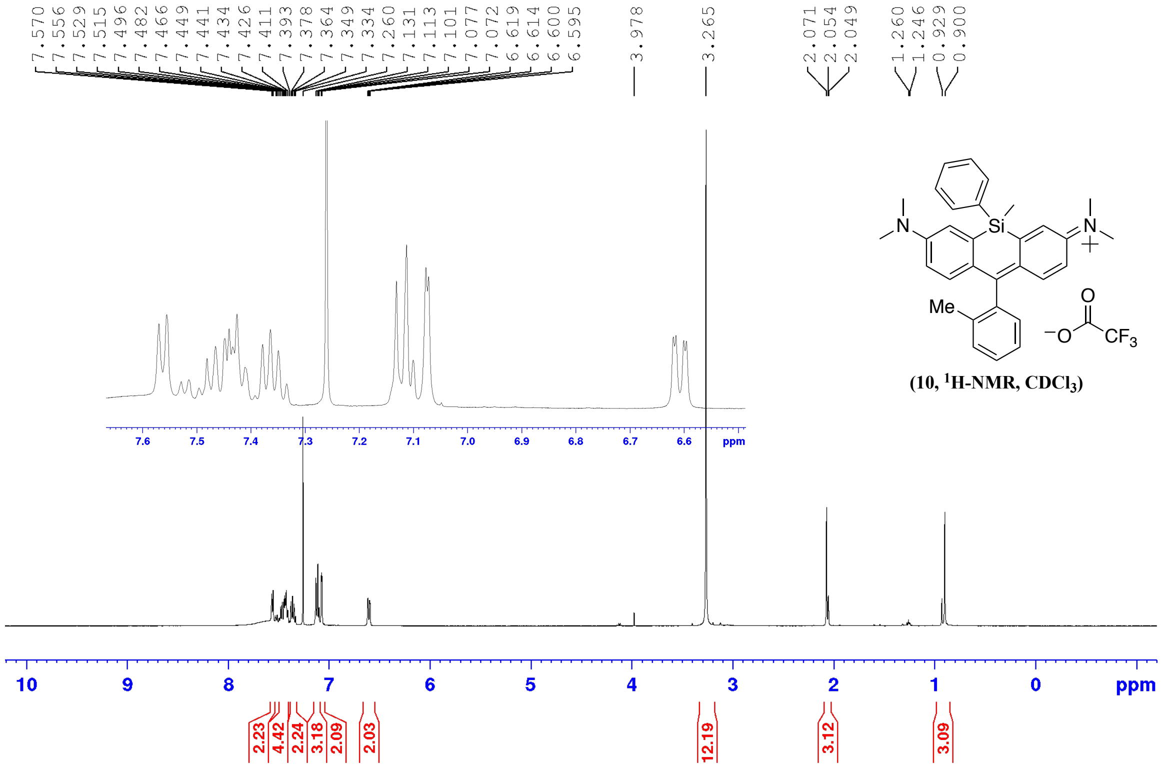

(10, ^{1}H-NMR, $CDCl_3$)

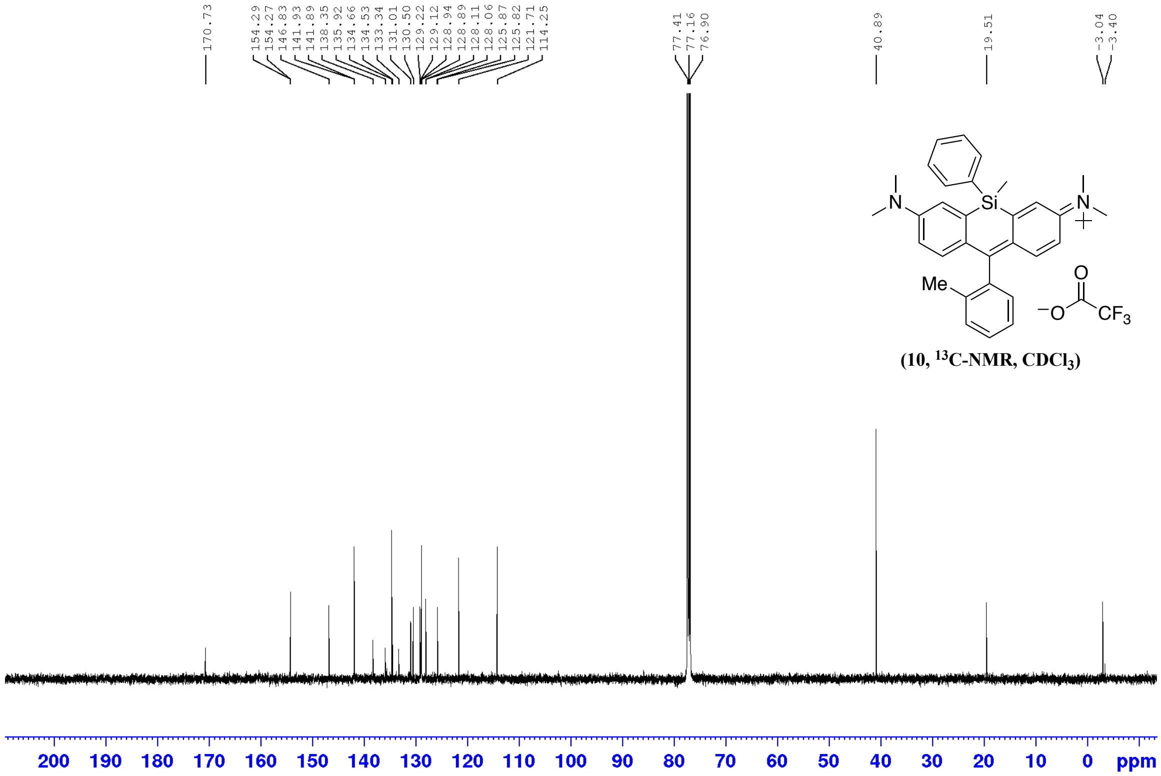

(10, ^{13}C-NMR, $CDCl_3$)

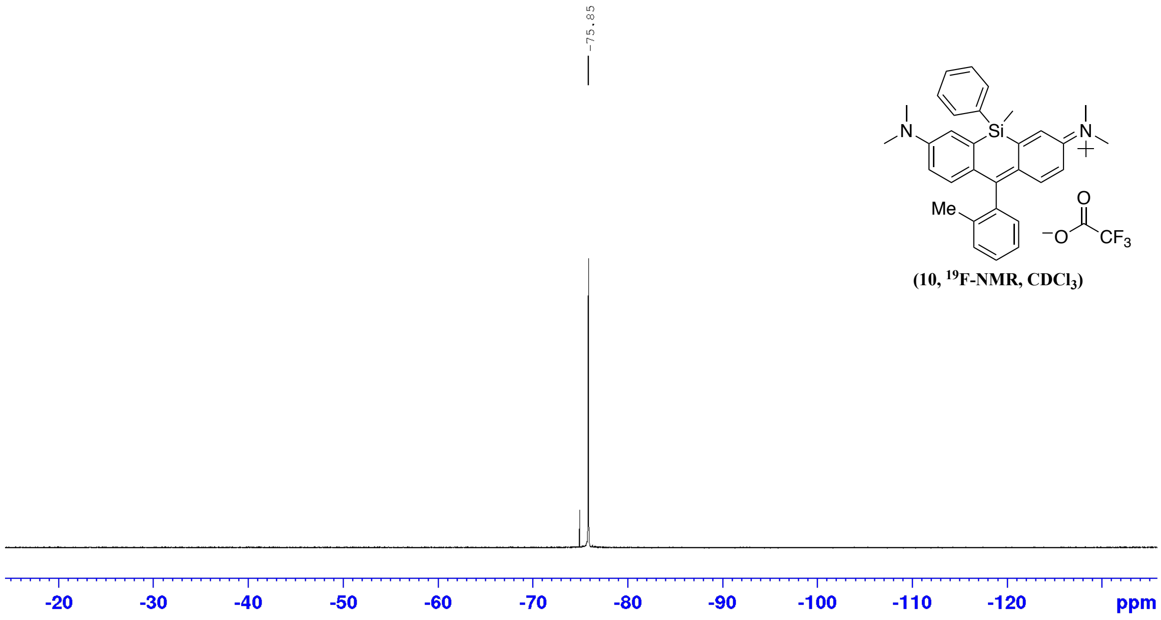

(10, ^{19}F-NMR, $CDCl_3$)

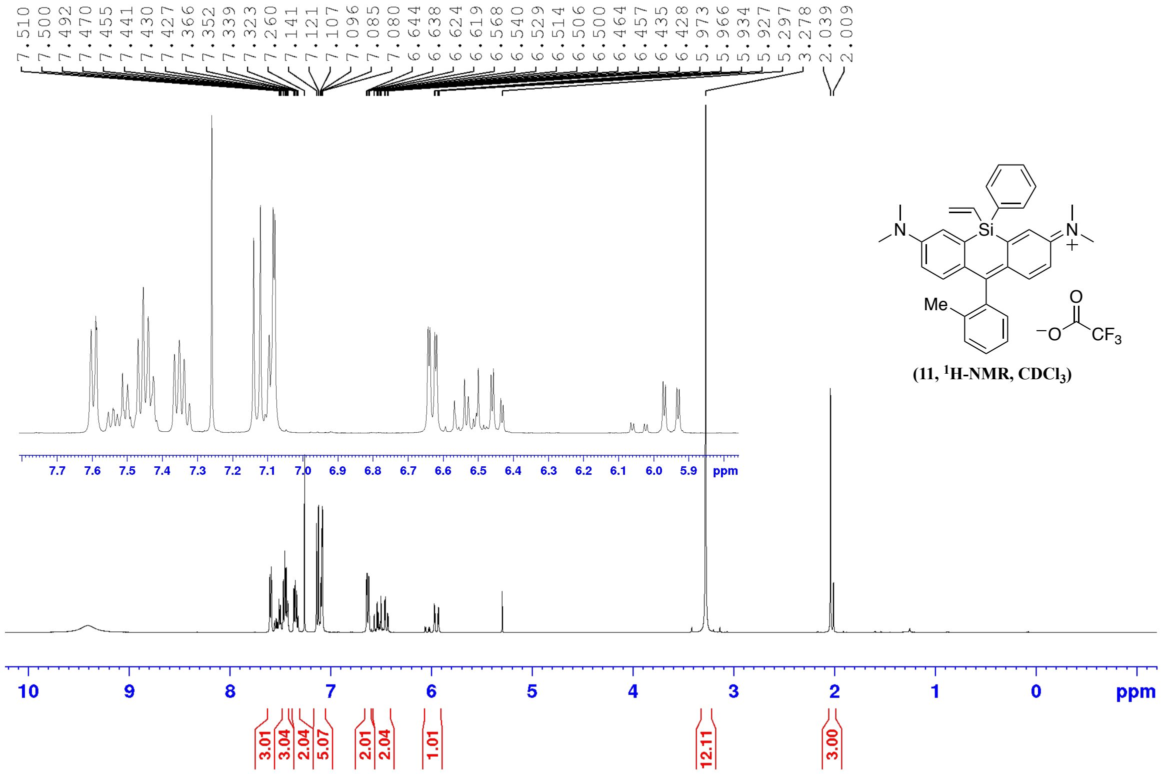

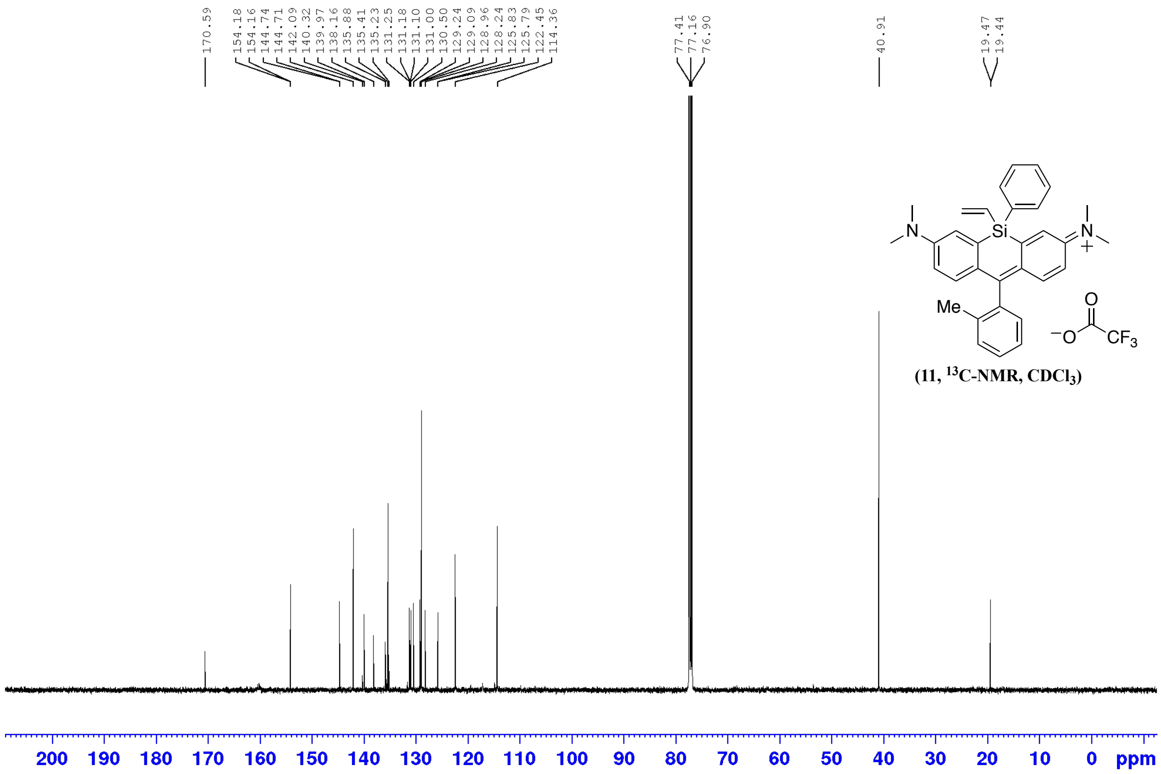

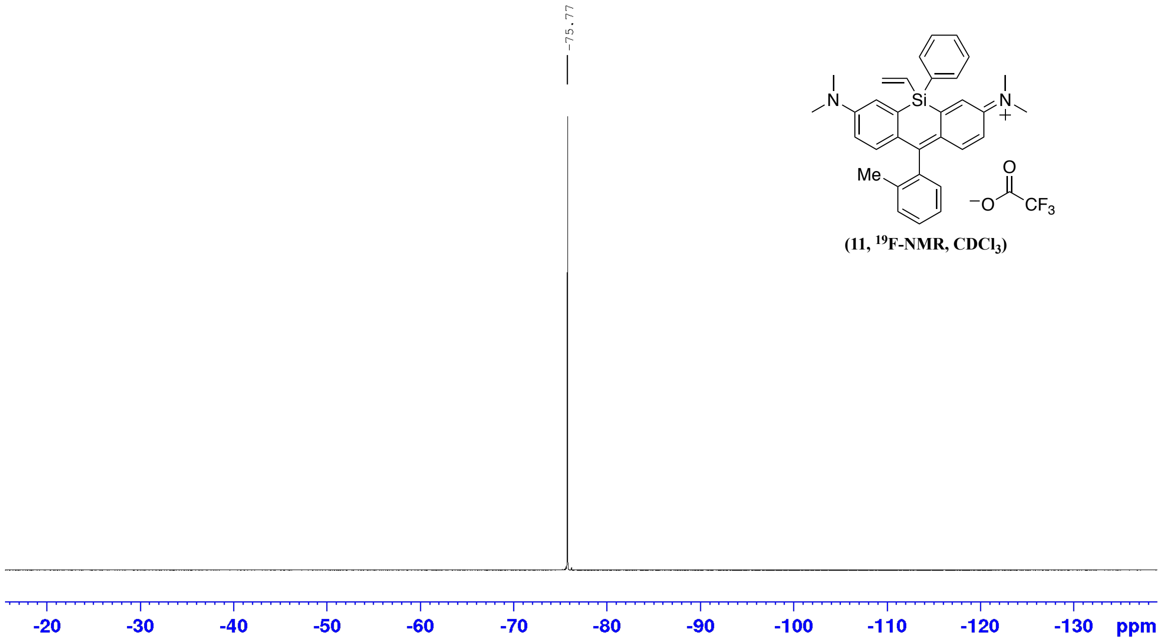

(11, ^{19}F-NMR, $CDCl_3$)

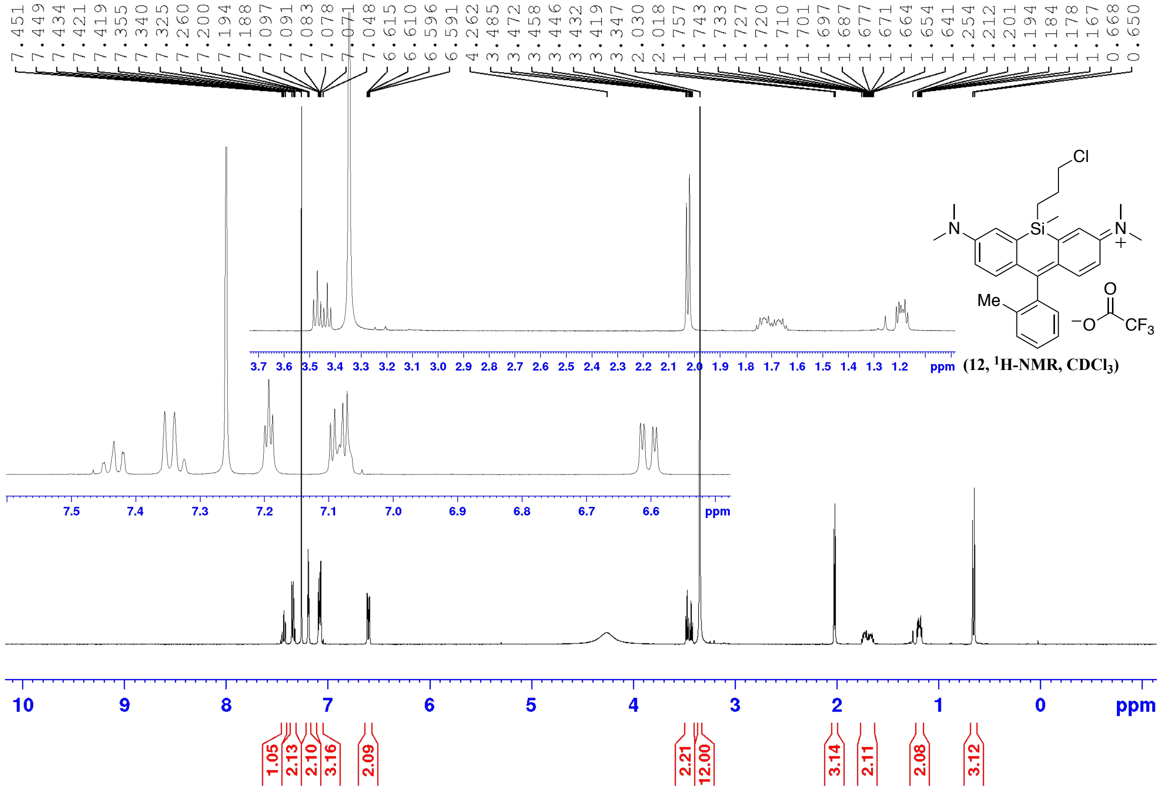

(12, ^{1}H-NMR, $CDCl_3$)

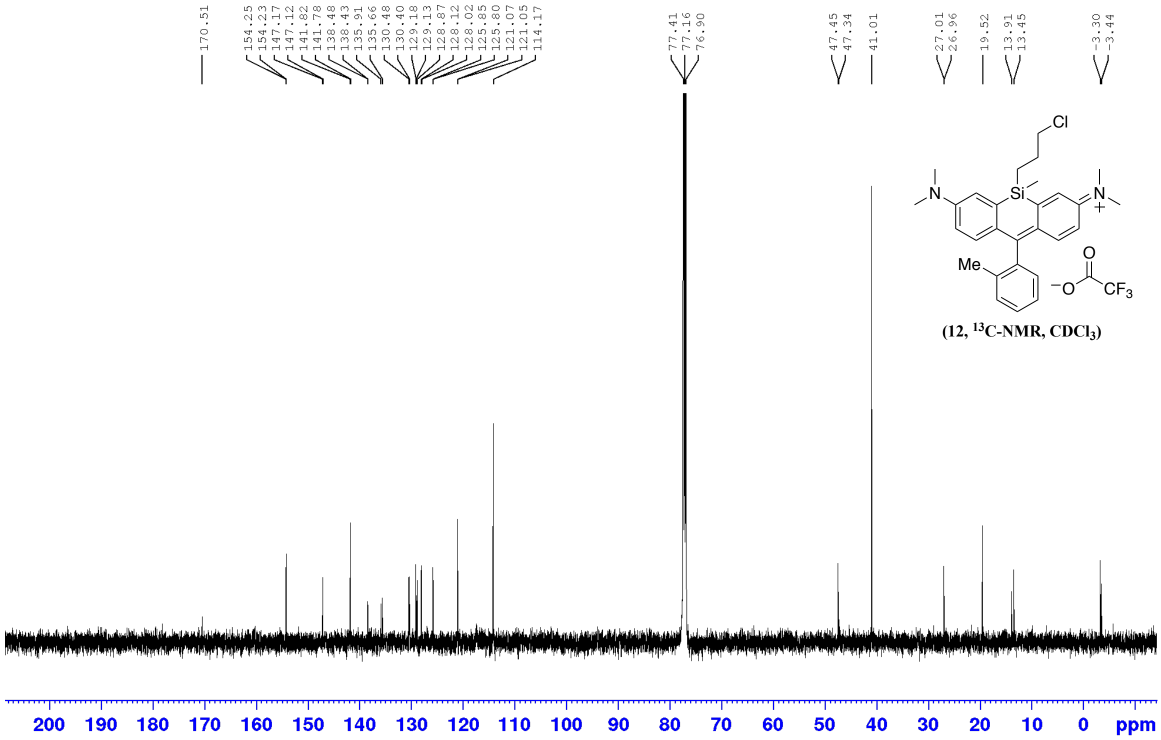

(12, ^{13}C-NMR, $CDCl_3$)

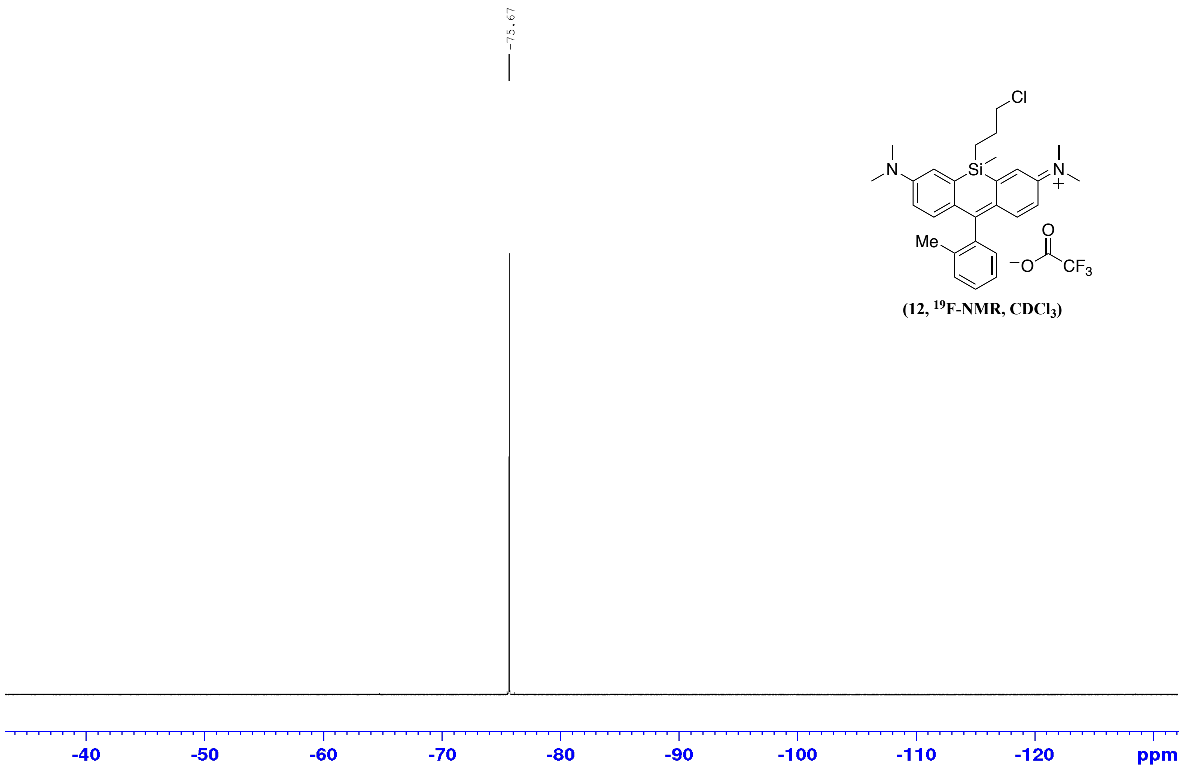

(12, ^{19}F-NMR, $CDCl_3$)

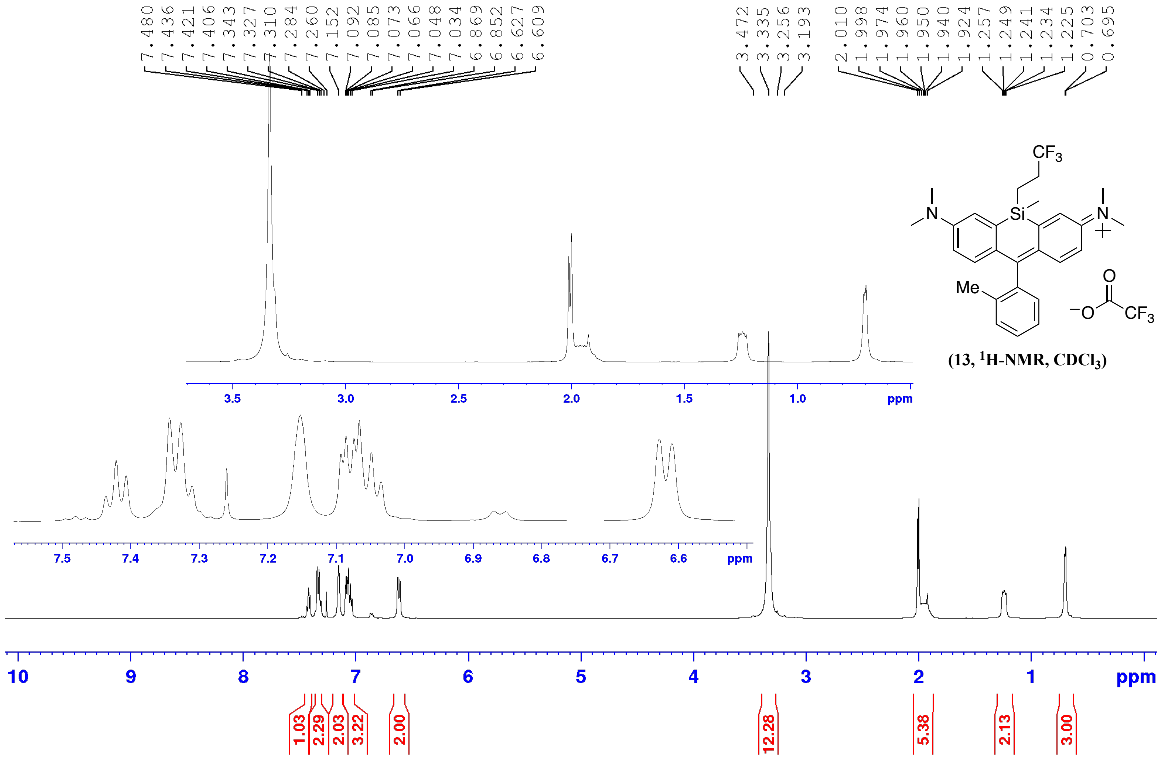

(13, ¹H-NMR, $CDCl_3$)

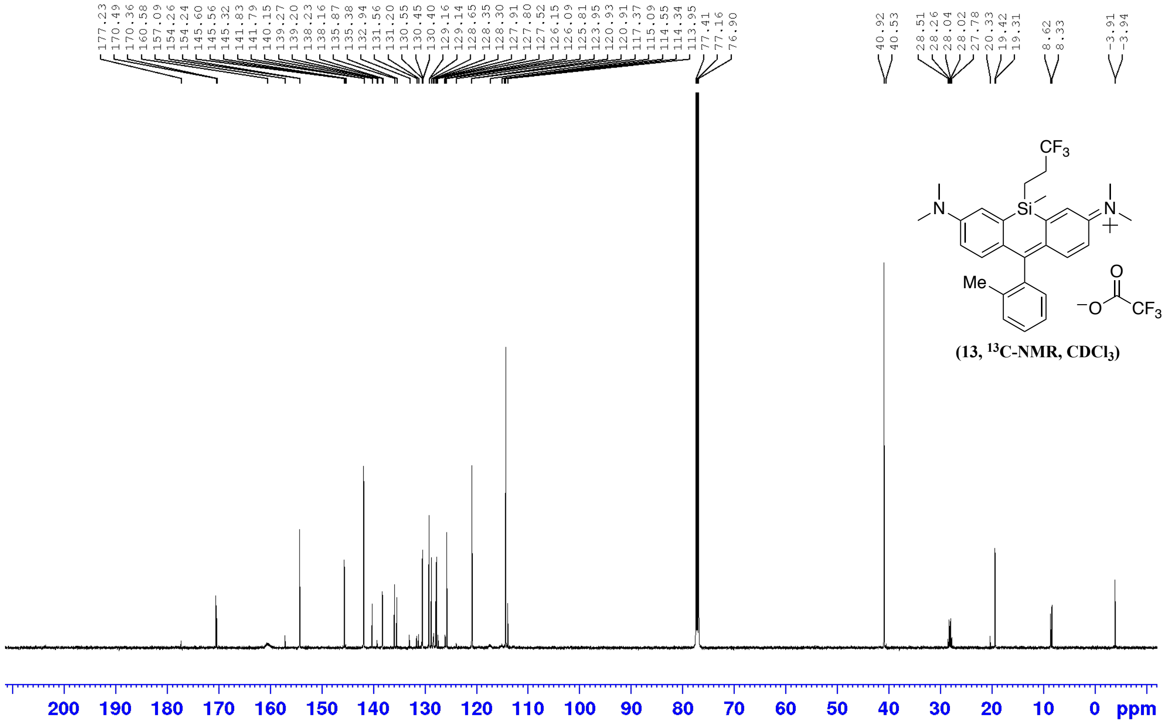

(13, ¹³C-NMR, $CDCl_3$)

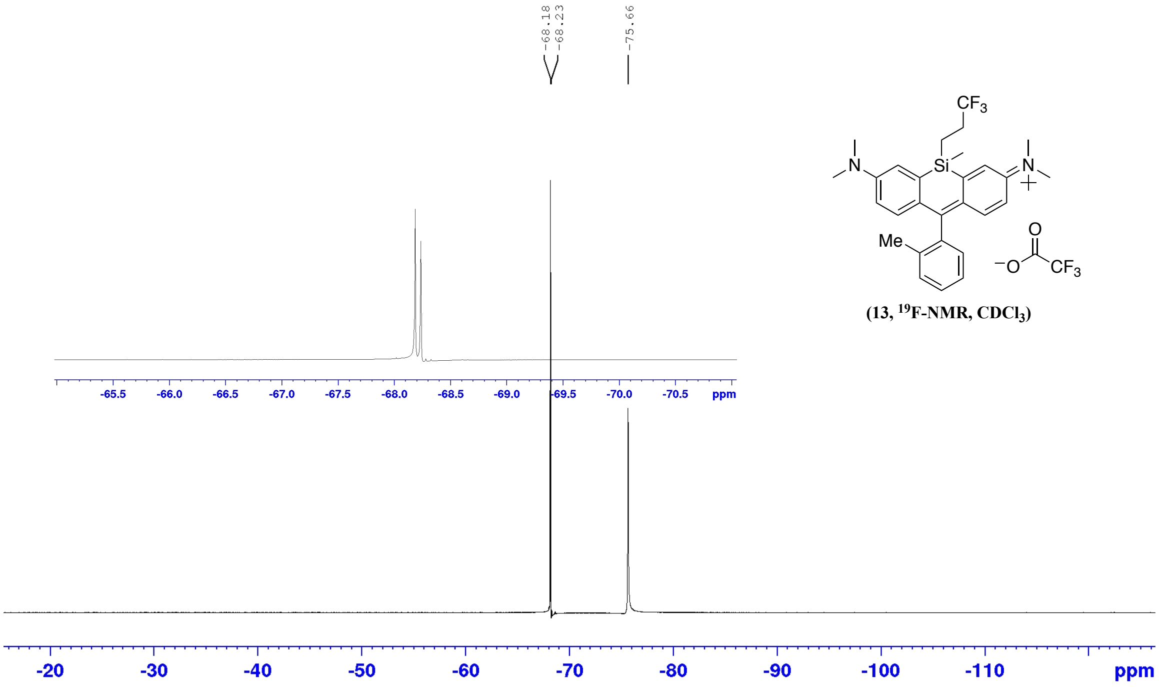

(13, ^{19}F-NMR, $CDCl_3$)

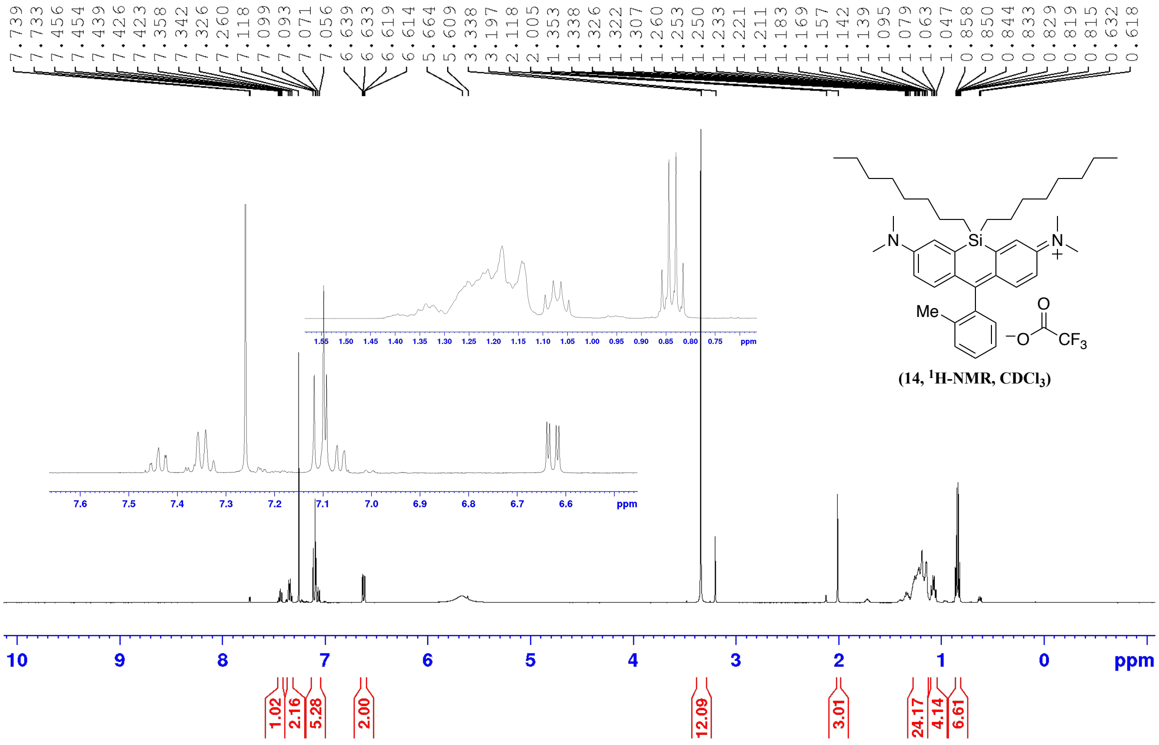

(14, ^{1}H-NMR, $CDCl_3$)

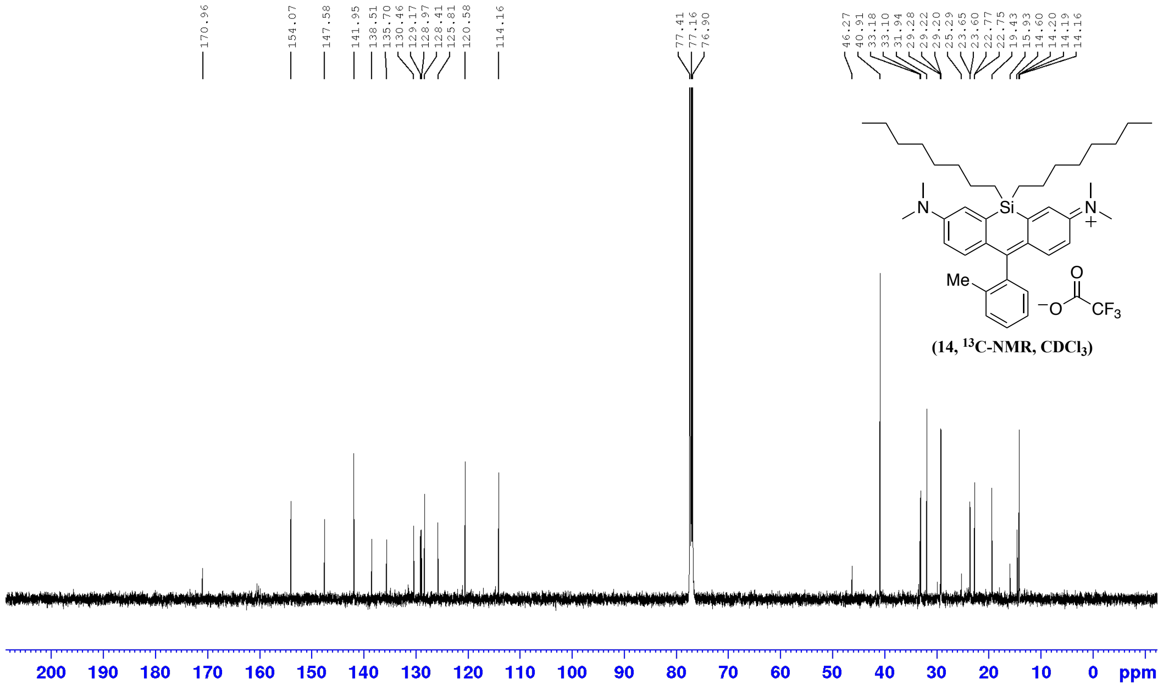

(14, ^{13}C-NMR, $CDCl_3$)

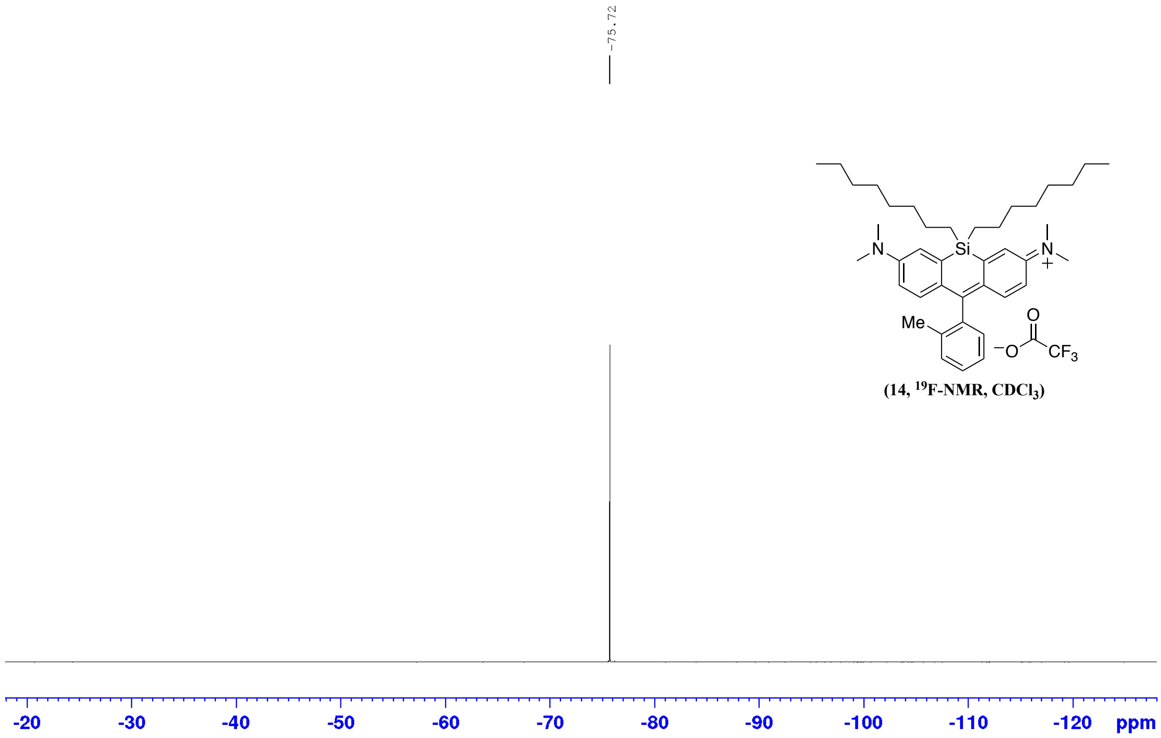

(14, ^{19}F-NMR, $CDCl_3$)

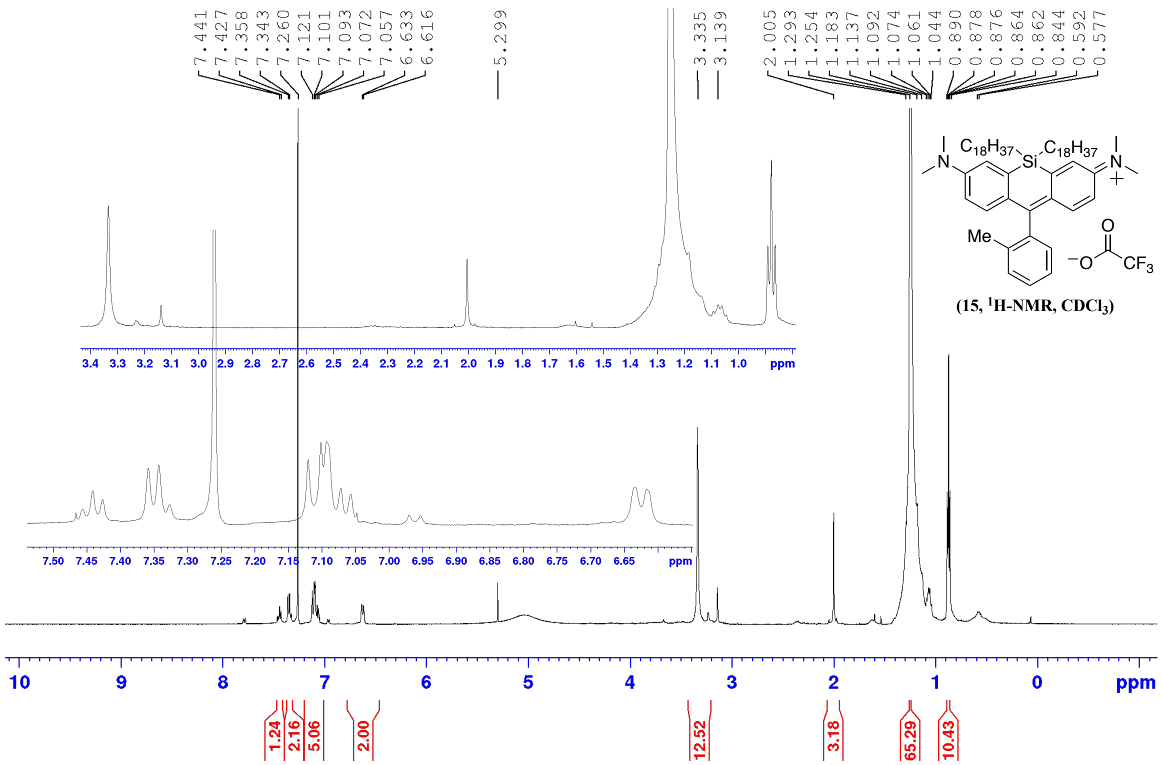
(15, ¹H-NMR, CDCl₃)
Me
C18H37
Si
N
O
CF3
7.441 7.427 7.358 7.343 7.260 7.121 7.101 7.093 7.072 7.057 6.633 6.616
5.299
3.335 3.139
2.005 1.293 1.254 1.183 1.137 1.092 1.074 1.061 1.044 0.890 0.878 0.876 0.864 0.862 0.844 0.592 0.577
1.24 2.16 5.06 2.00 12.52 3.18 65.29 10.43
ppm

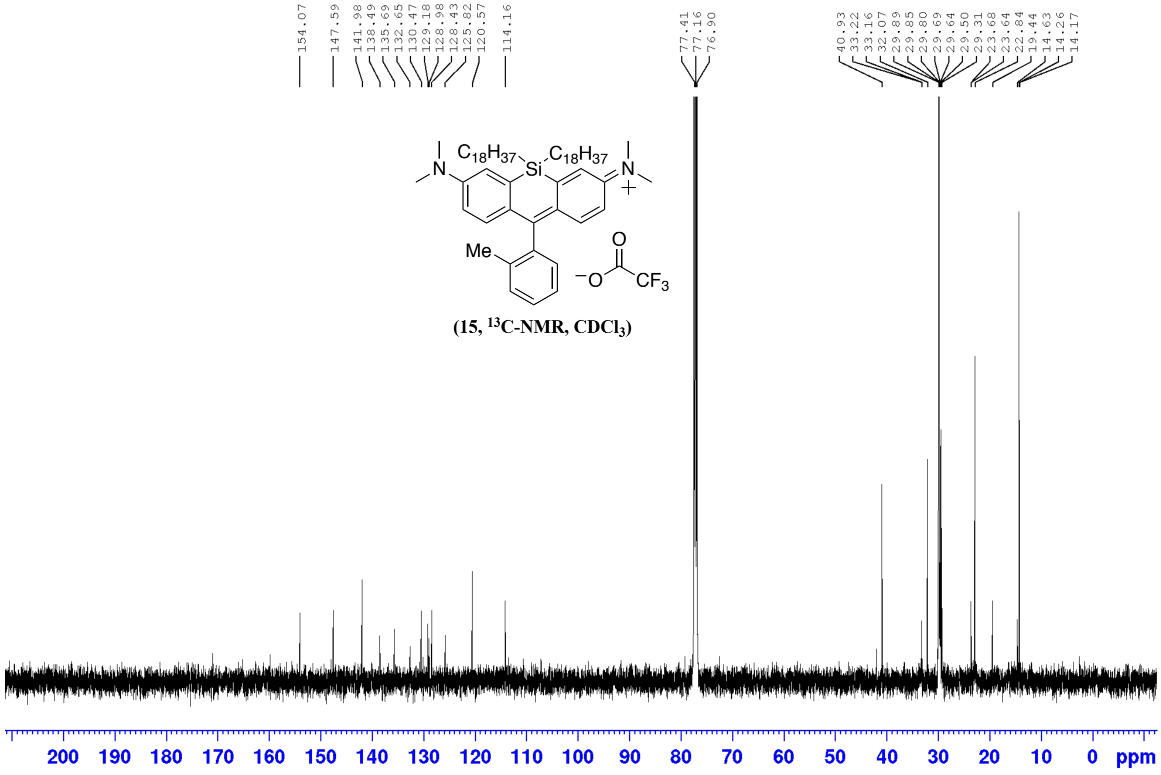
(15, ¹³C-NMR, CDCl₃)
Me
C18H37
Si
N
O
CF3
154.07 147.59 141.98 138.49 135.69 132.65 130.47 129.18 128.98 128.43 125.82 120.57 114.16
77.41 77.16 76.90
40.93 33.22 33.16 32.07 29.89 29.85 29.80 29.69 29.64 29.50 29.31 23.68 23.64 22.84 19.44 14.63 14.26 14.17
ppm

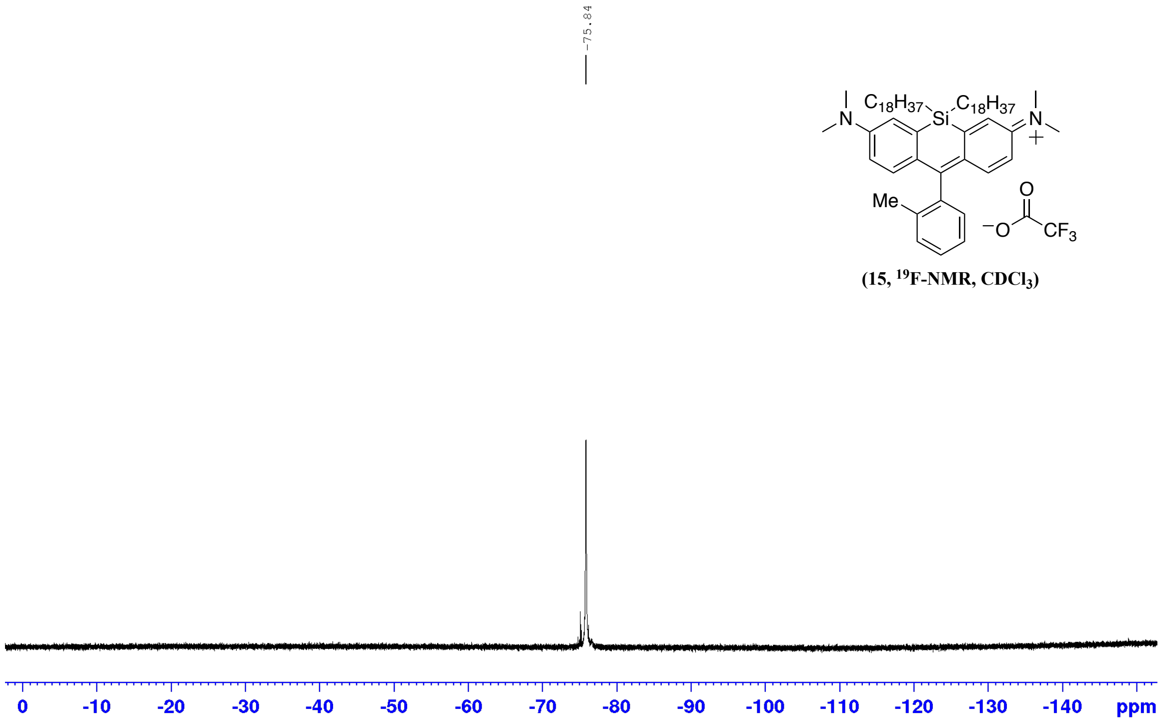

(15, ^{19}F-NMR, $CDCl_3$)

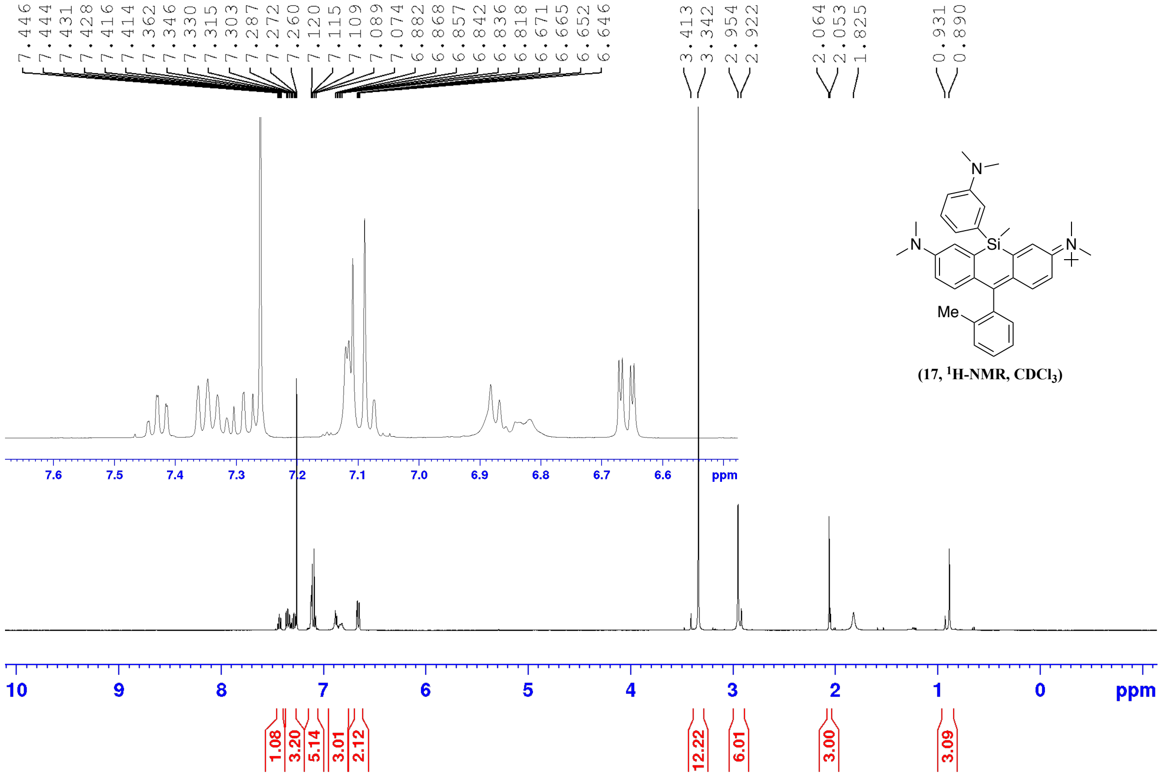

(17, ^{1}H-NMR, $CDCl_3$)

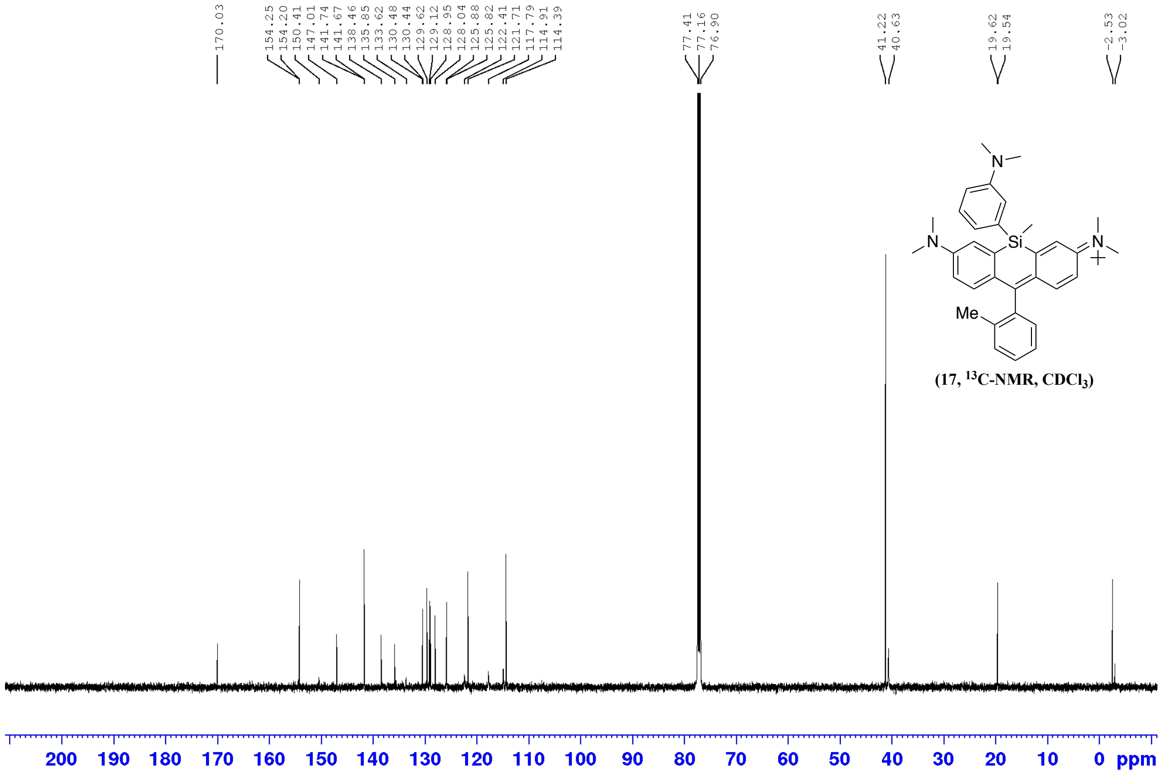

(17, ^{13}C-NMR, $CDCl_3$)

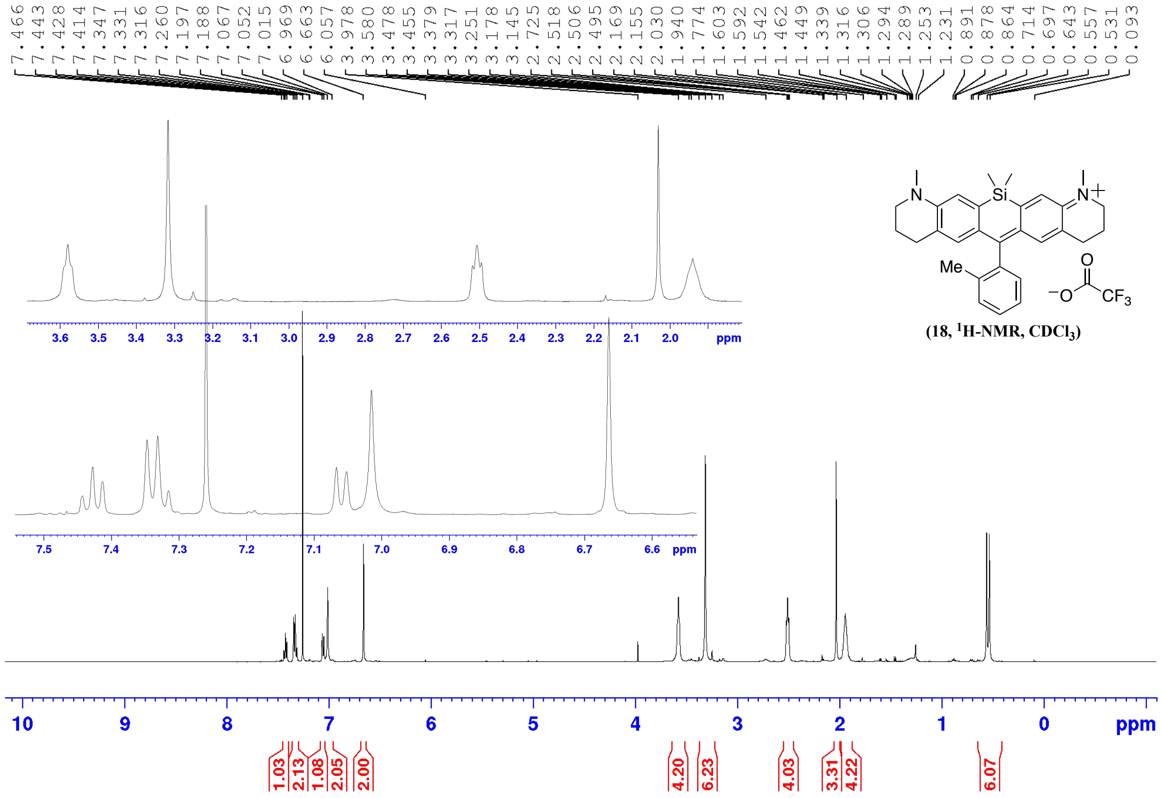

(18, ^{1}H-NMR, $CDCl_3$)

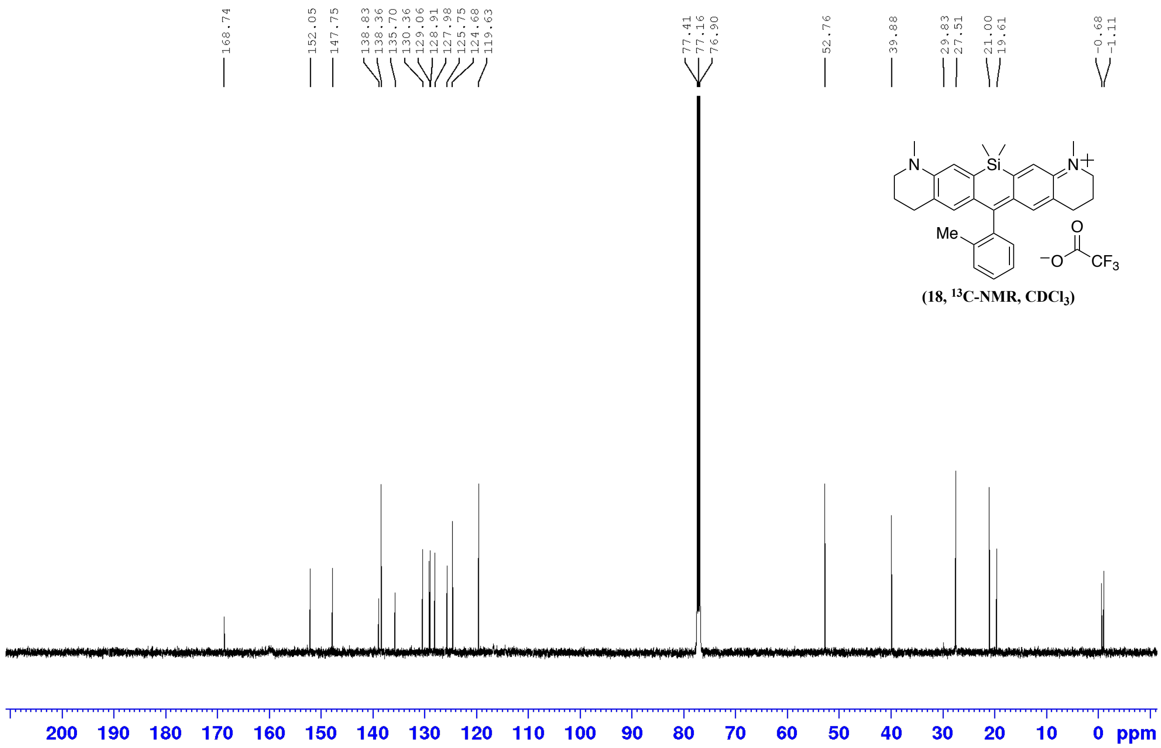
168.74
152.05
147.75
138.83
138.36
135.70
130.36
129.06
128.91
127.98
125.75
124.68
119.63
77.41
77.16
76.90
52.76
39.88
29.83
27.51
21.00
19.61
-0.68
-1.11
Si
N
N+
Me
O
−O
CF3
(18, 13C-NMR, CDCl3)
200 190 180 170 160 150 140 130 120 110 100 90 80 70 60 50 40 30 20 10 0 ppm

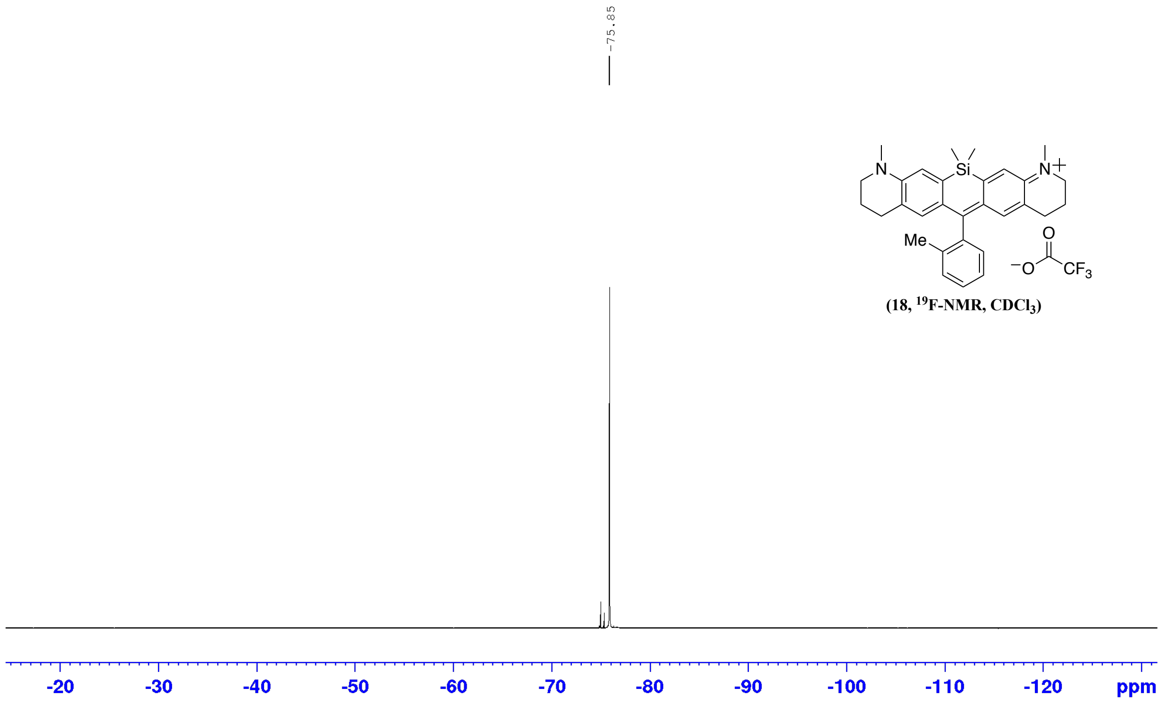
-75.85
Si
N
N+
Me
O
−O
CF3
(18, 19F-NMR, CDCl3)
-20 -30 -40 -50 -60 -70 -80 -90 -100 -110 -120 ppm

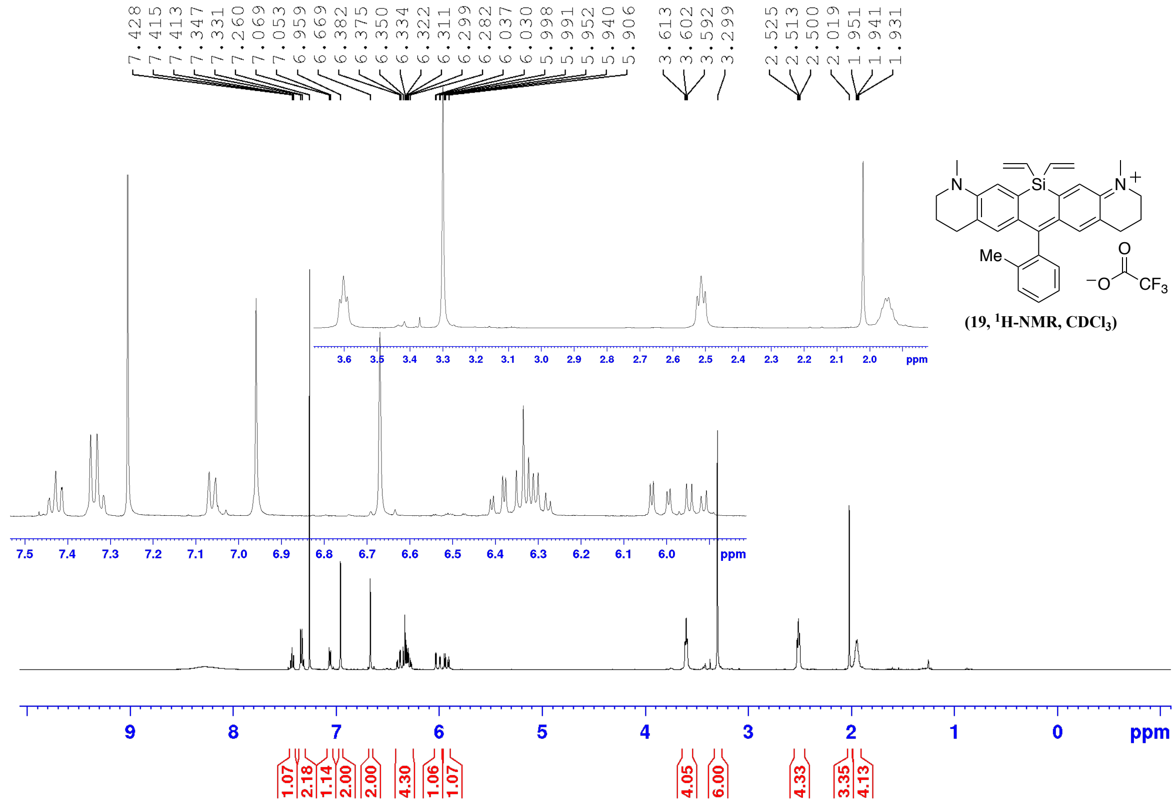

(19, ¹H-NMR, CDCl$_3$)

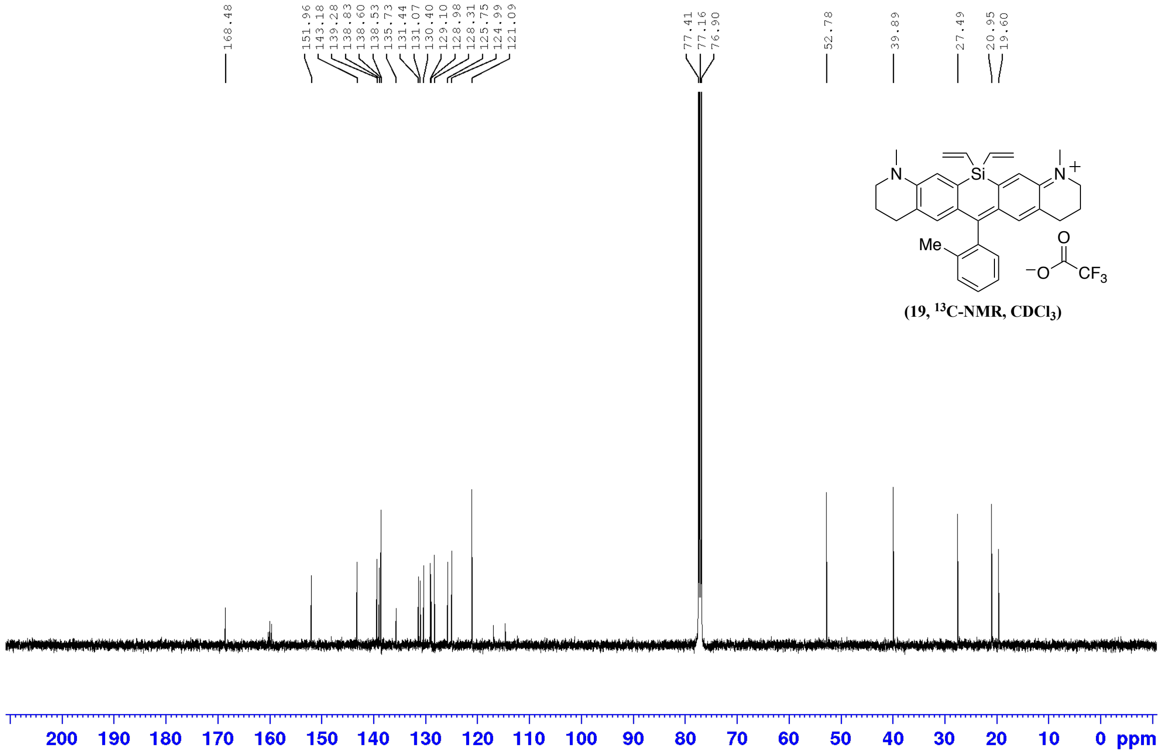

(19, ¹³C-NMR, CDCl$_3$)

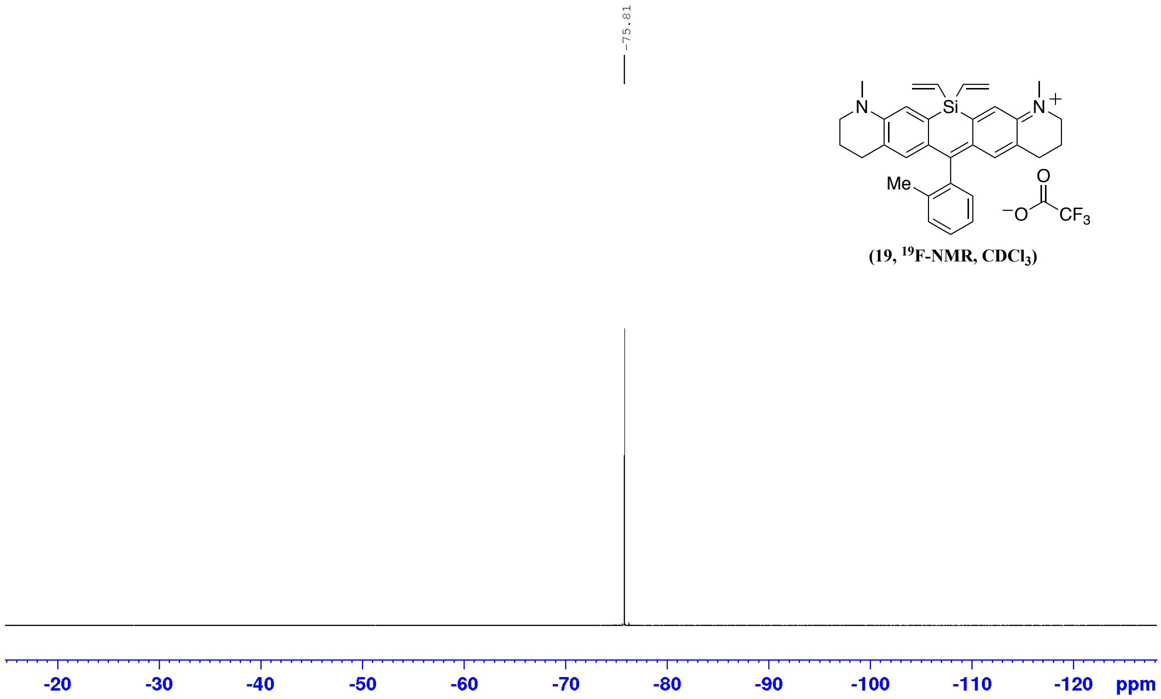

(19, ^{19}F-NMR, CDCl$_3$)

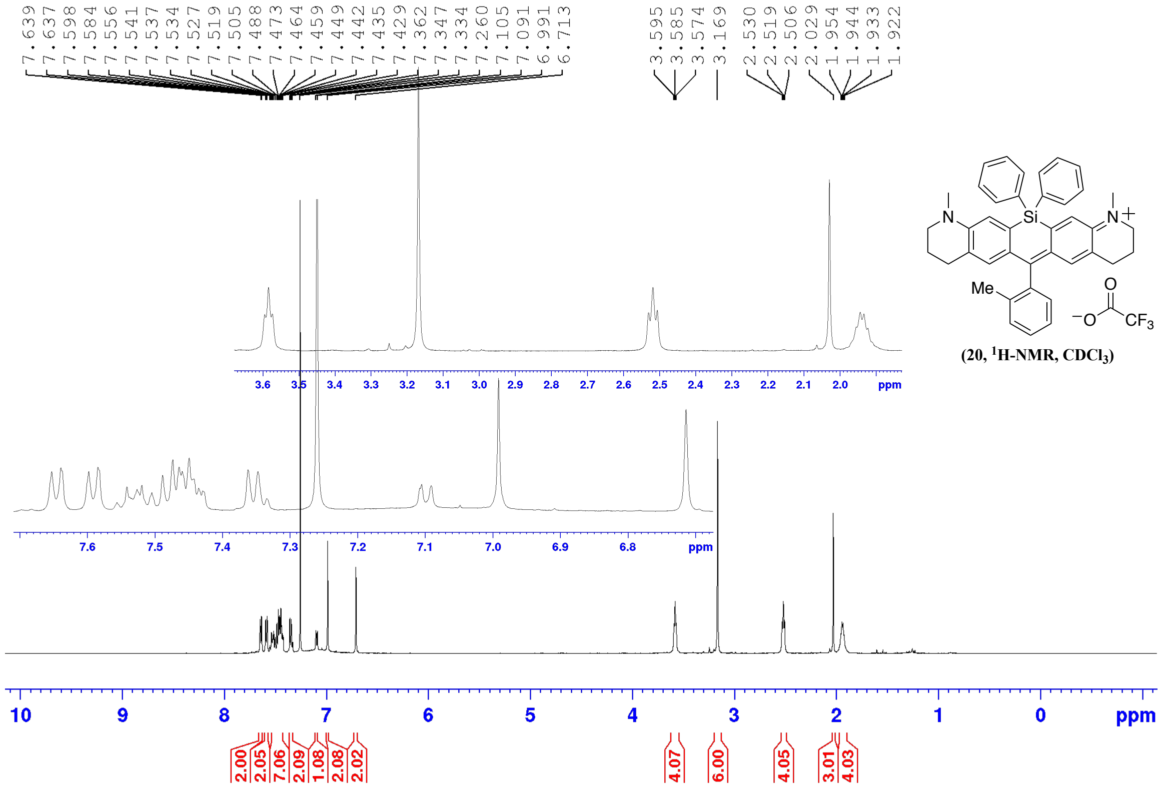

(20, ^{1}H-NMR, CDCl$_3$)

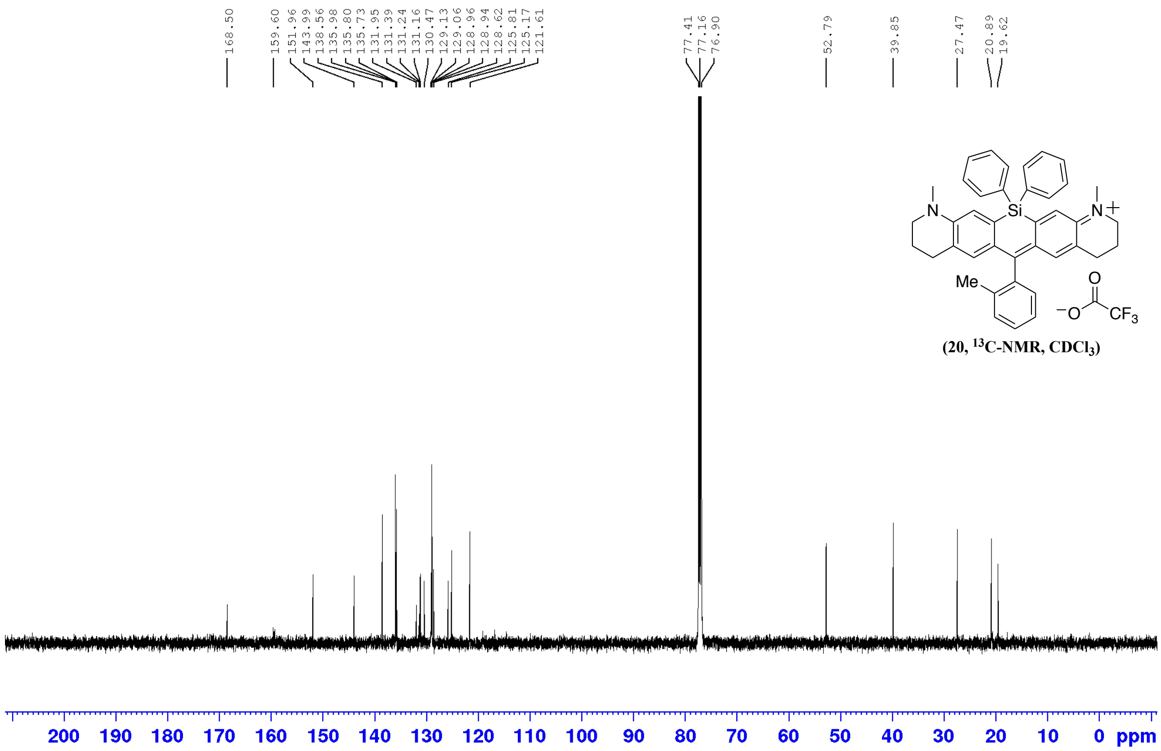

(20, ^{13}C-NMR, CDCl$_3$)

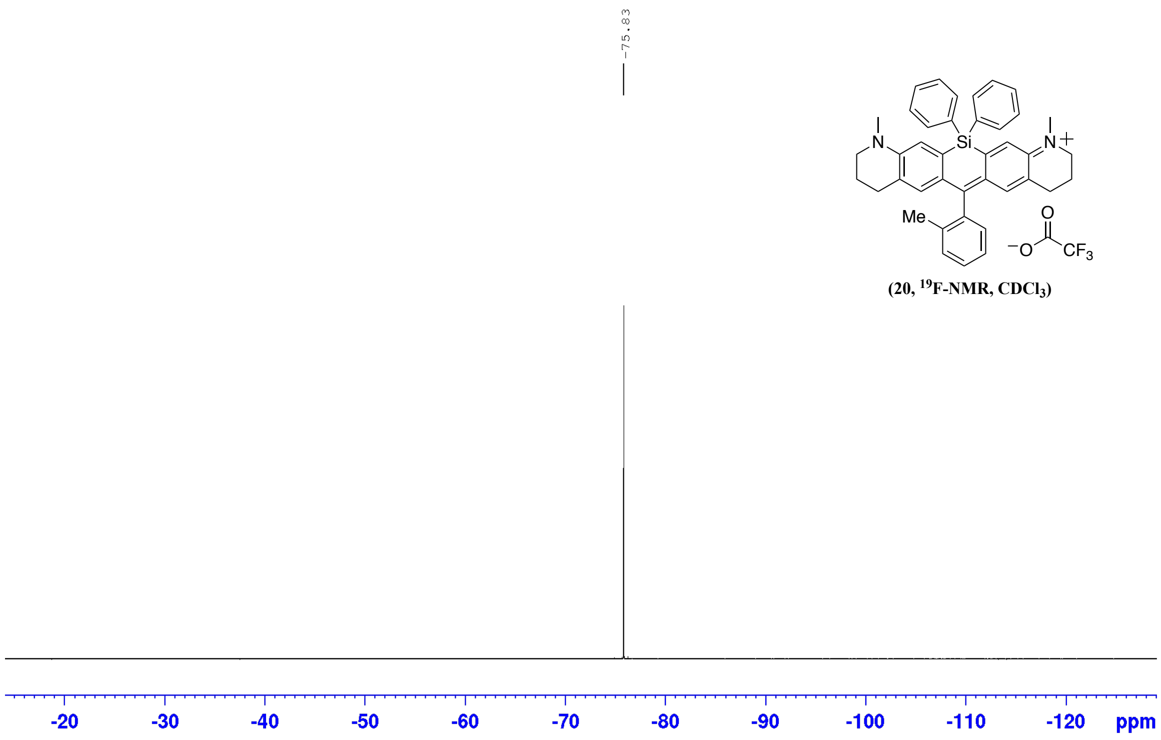

(20, ^{19}F-NMR, CDCl$_3$)

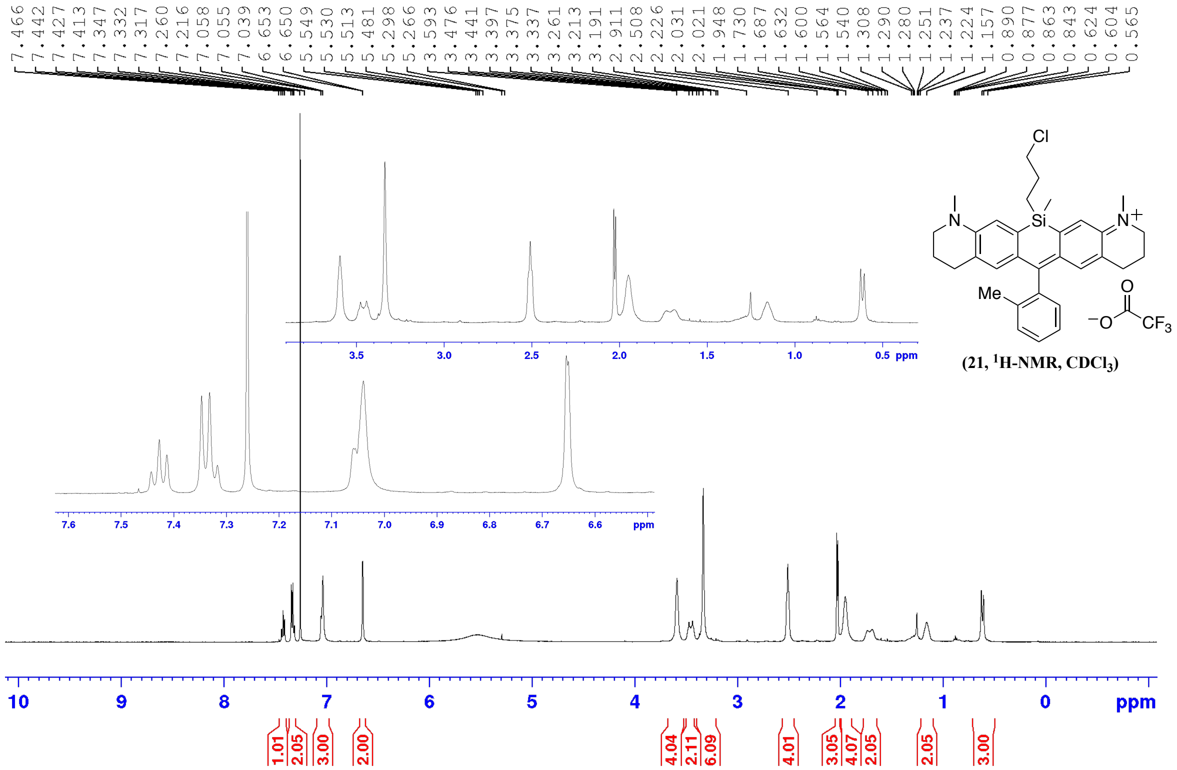
(21, ^{1}H-NMR, $CDCl_3$)

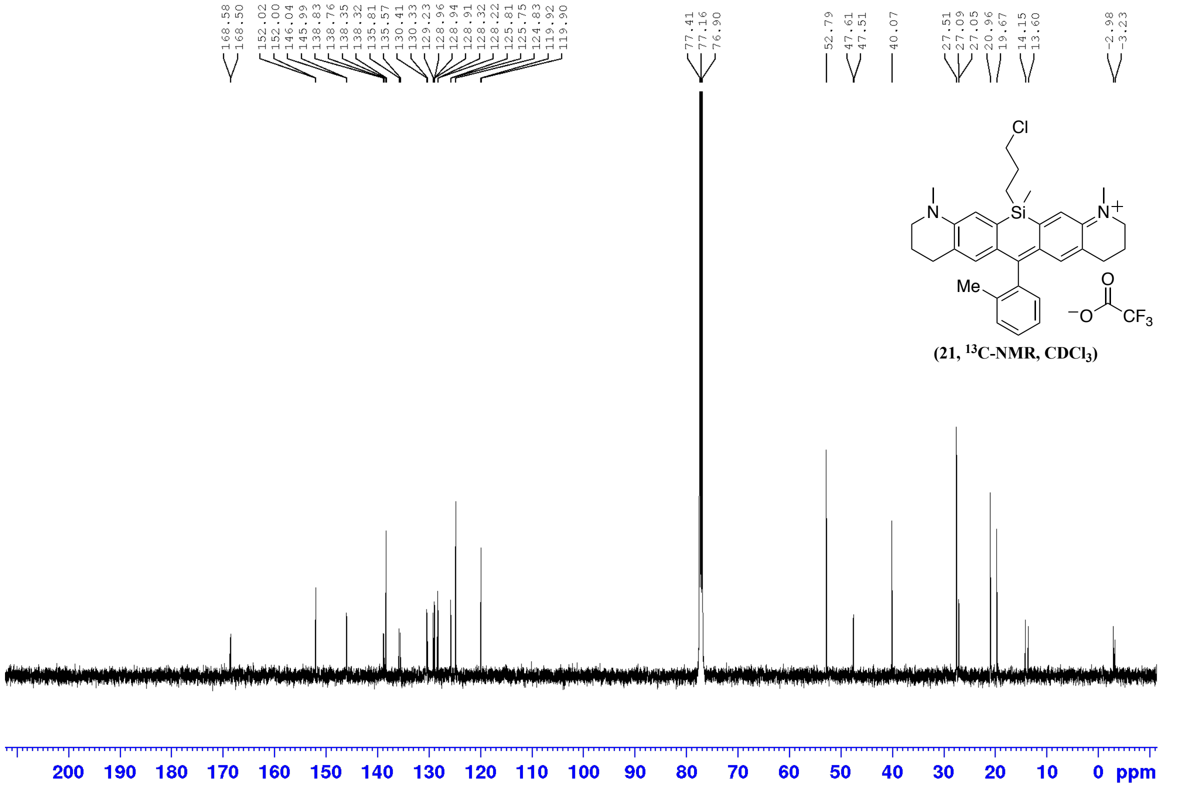
(21, ^{13}C-NMR, $CDCl_3$)

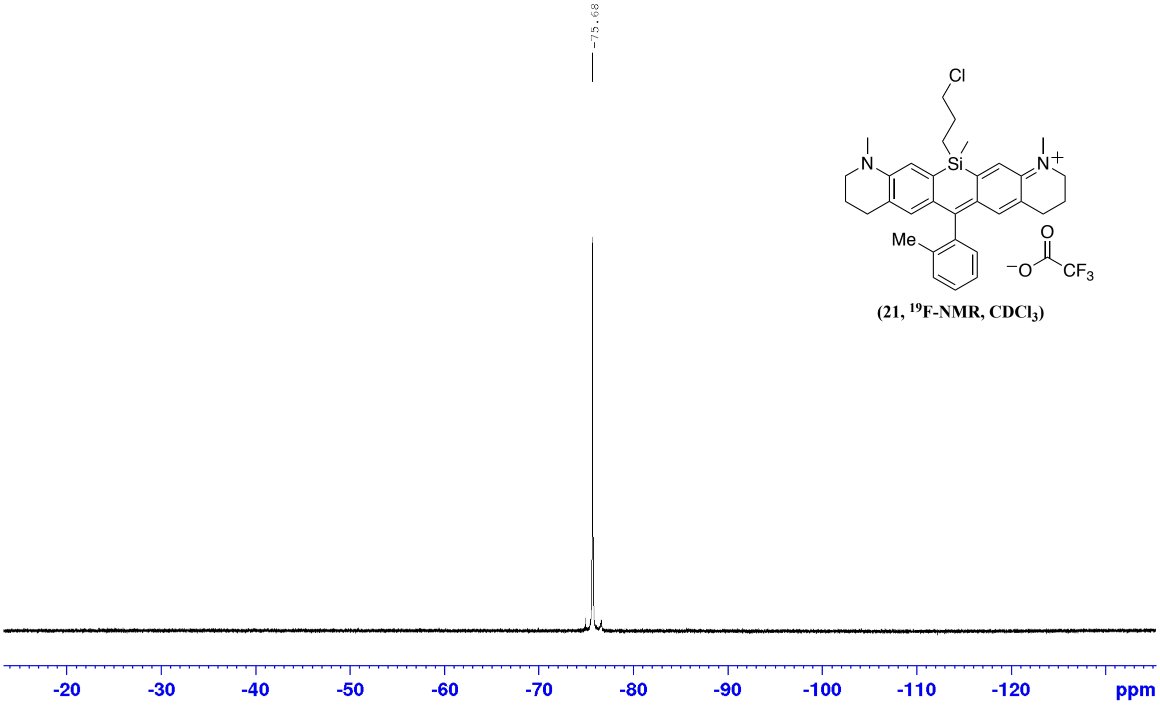

(21, ^{19}F-NMR, $CDCl_3$)

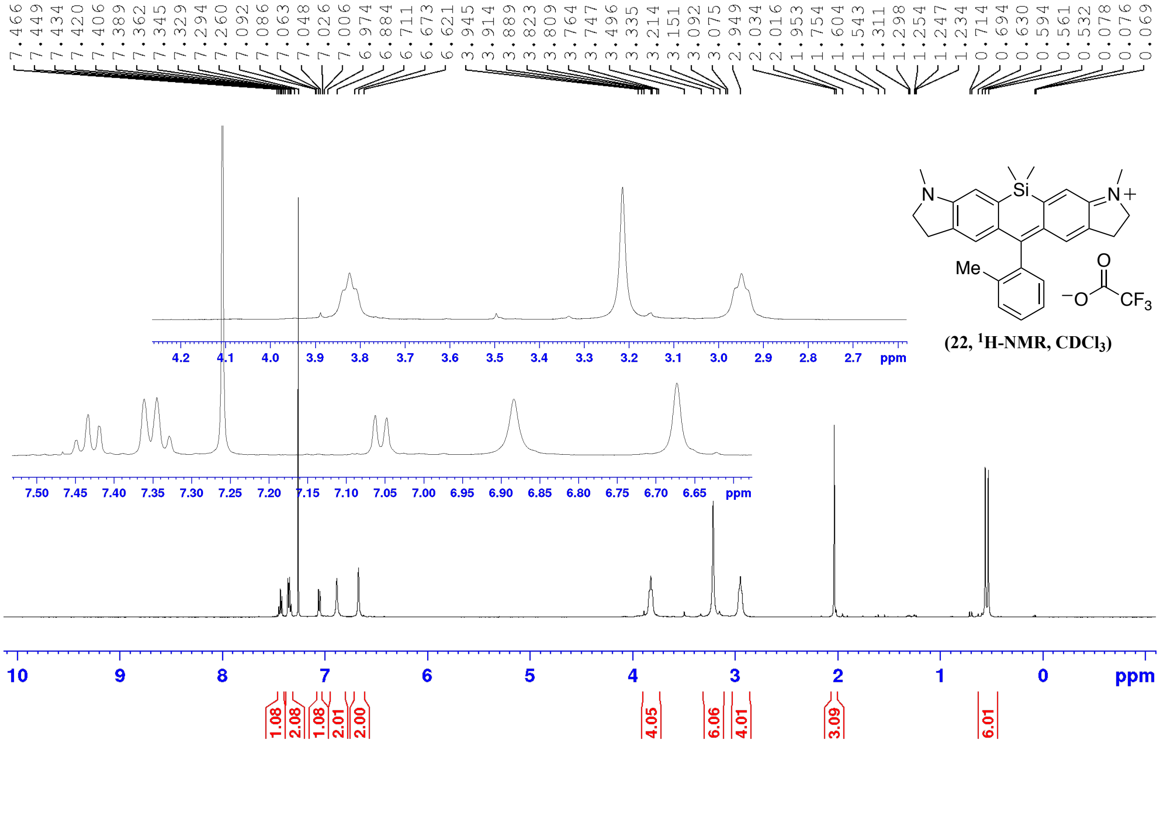

(22, ^{1}H-NMR, $CDCl_3$)

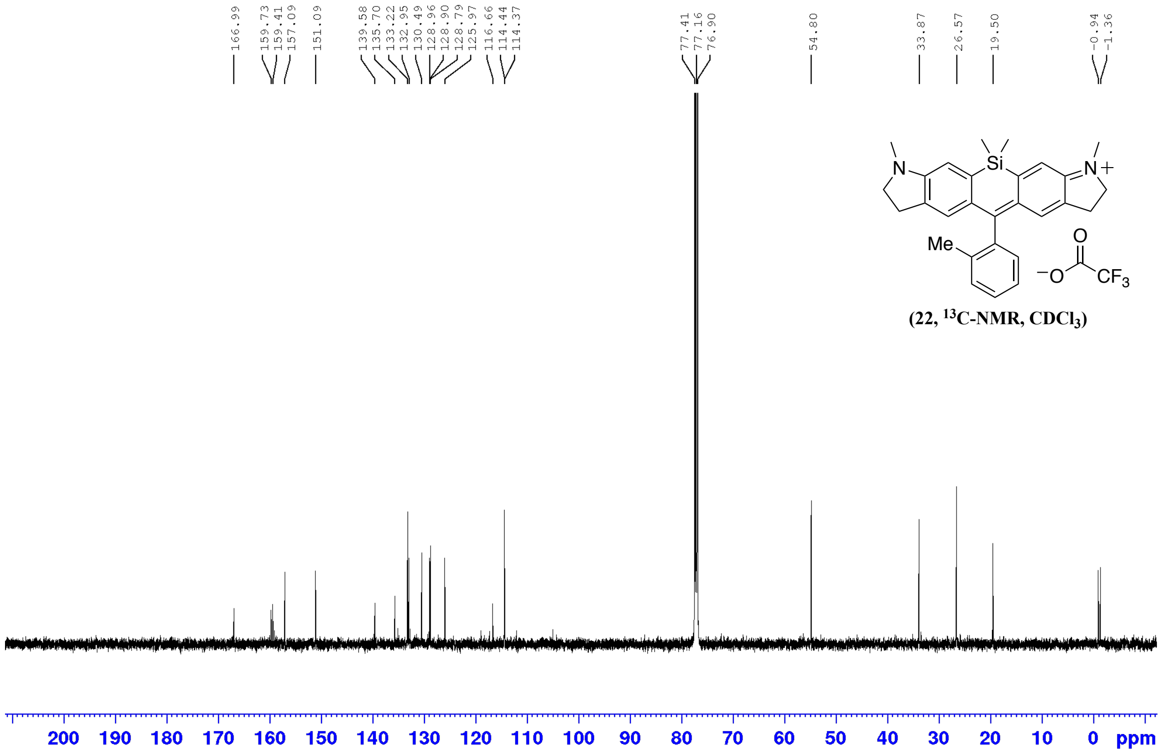

(22, ^{13}C-NMR, $CDCl_3$)

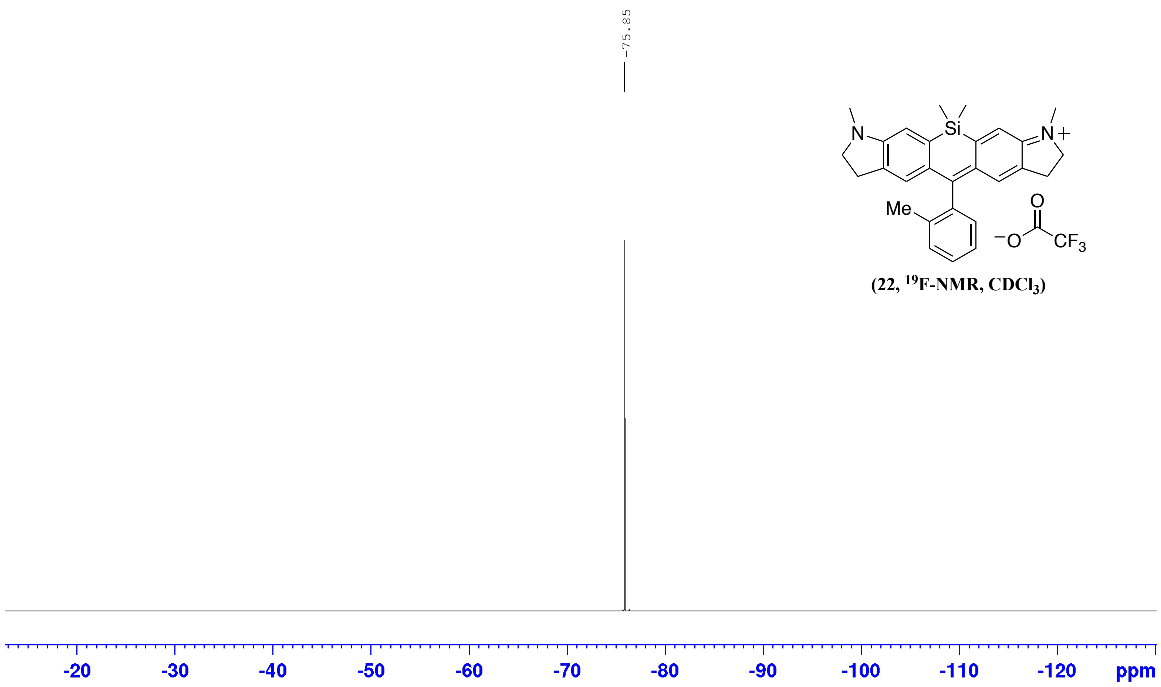

(22, ^{19}F-NMR, $CDCl_3$)

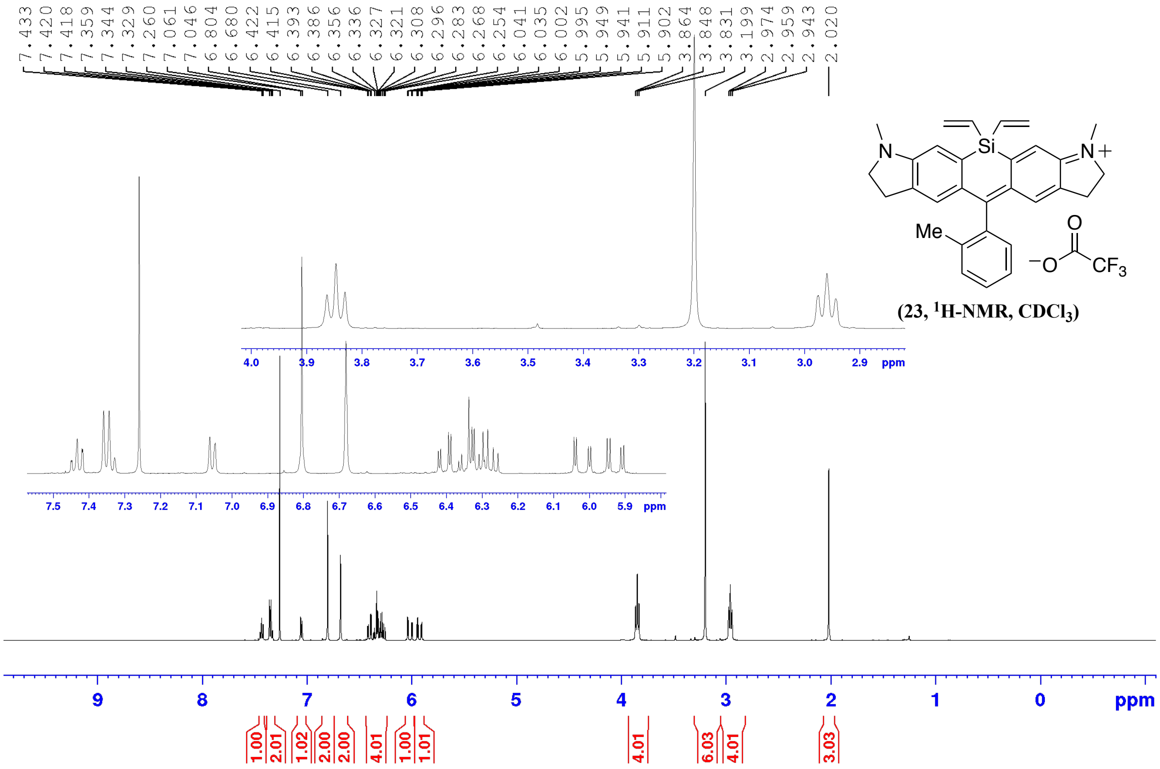

(23, ^{1}H-NMR, $CDCl_3$)

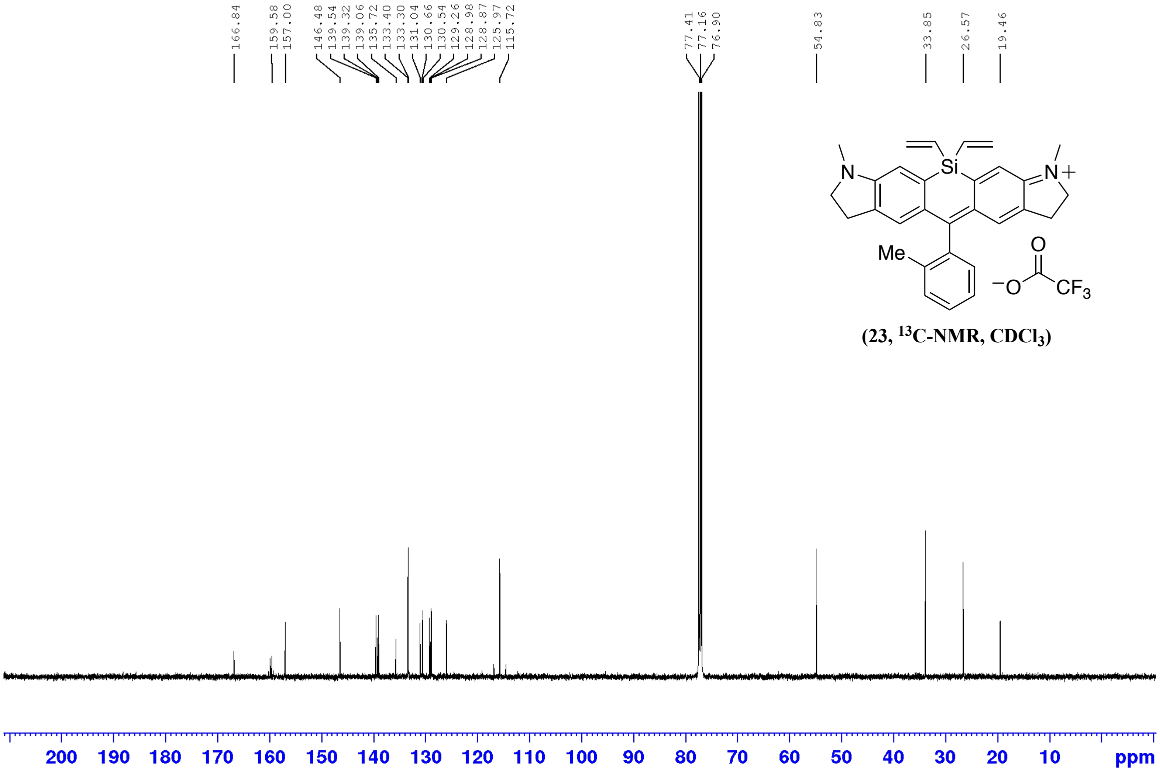

(23, ^{13}C-NMR, $CDCl_3$)

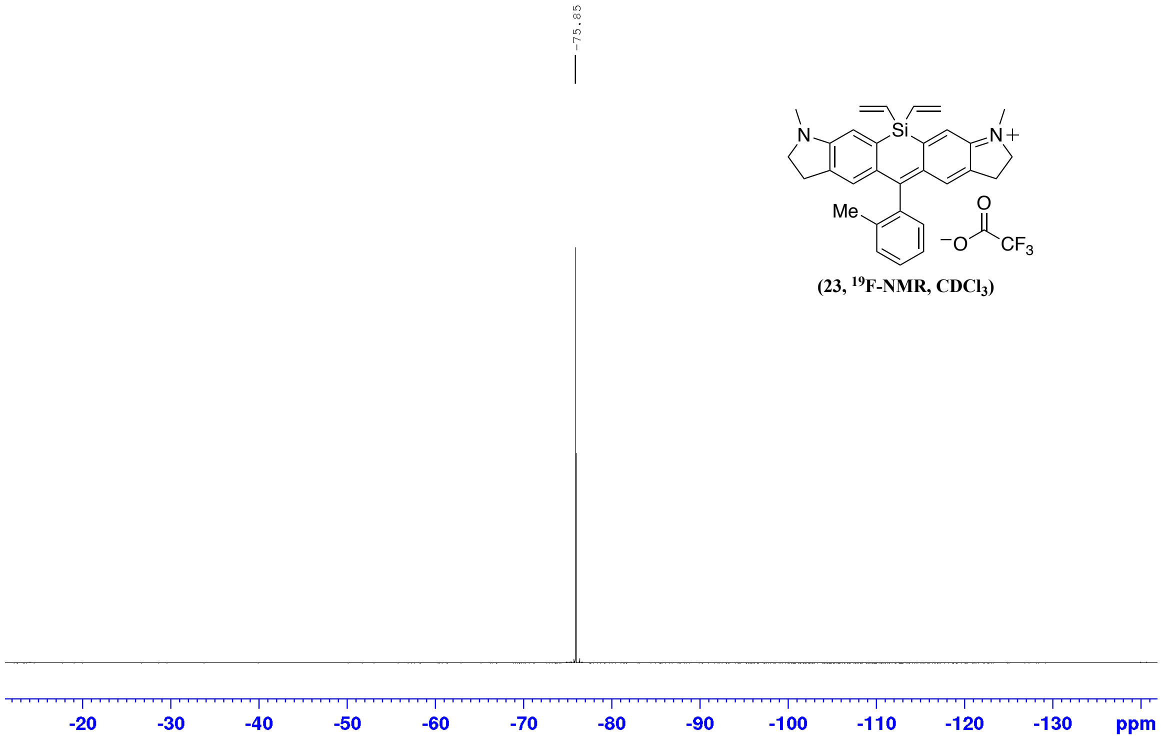
-75.85
Si
N
N+
Me
O
−O
CF3
(23, 19F-NMR, CDCl3)
-20
-30
-40
-50
-60
-70
-80
-90
-100
-110
-120
-130
ppm

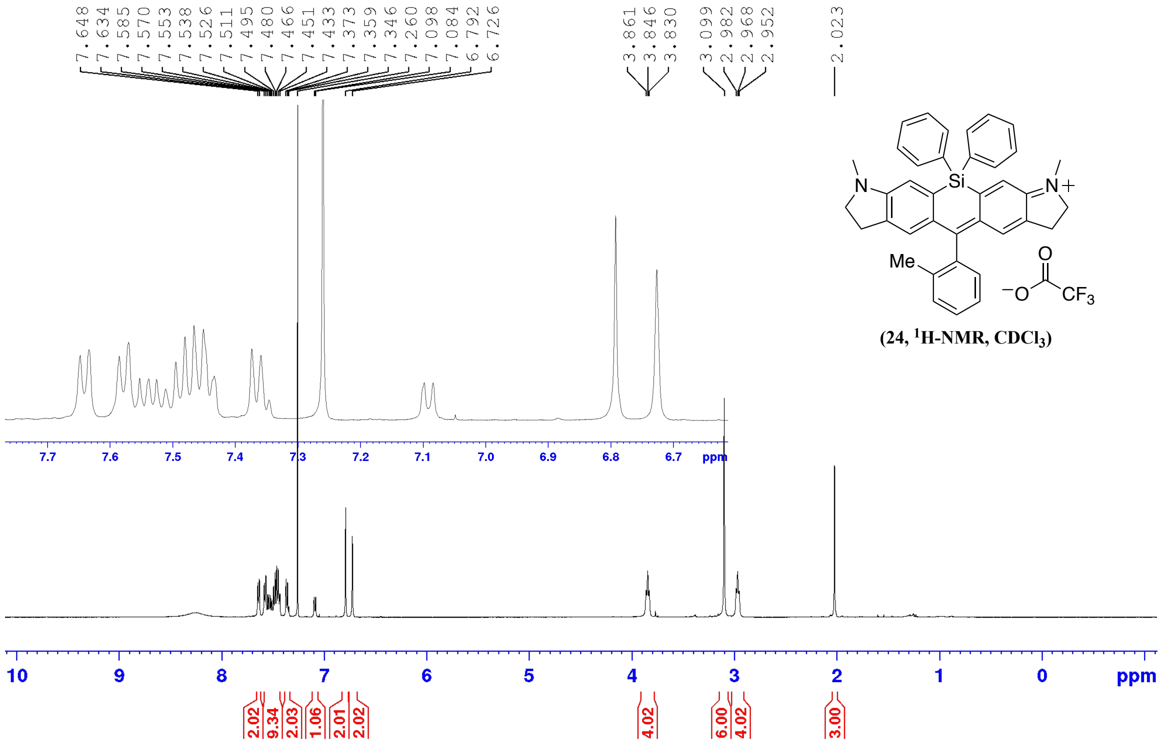
7.648
7.634
7.585
7.570
7.553
7.538
7.526
7.511
7.495
7.480
7.466
7.451
7.433
7.373
7.359
7.346
7.260
7.098
7.084
6.792
6.726
3.861
3.846
3.830
3.099
2.982
2.968
2.952
2.023
Si
N
N+
Me
O
−O
CF3
(24, 1H-NMR, CDCl3)
7.7
7.6
7.5
7.4
7.3
7.2
7.1
7.0
6.9
6.8
6.7
ppm
10
9
8
7
6
5
4
3
2
1
0
ppm
2.02
9.34
2.03
1.06
2.01
2.02
4.02
6.00
4.02
3.00

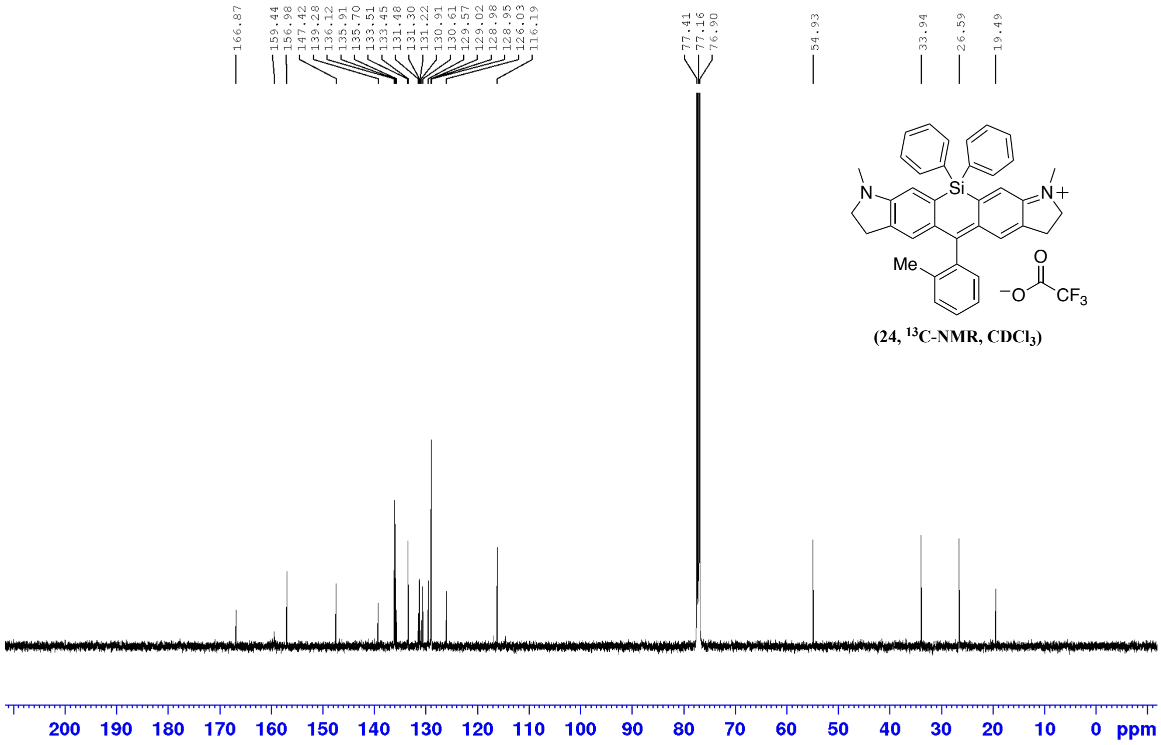
166.87
159.44
156.98
147.42
139.28
136.12
135.91
135.70
133.51
133.45
131.48
131.30
131.22
130.91
130.61
129.57
129.02
128.98
128.95
126.03
116.19
77.41
77.16
76.90
54.93
33.94
26.59
19.49
Si
N
N+
Me
O
−O
CF3
(24, 13C-NMR, CDCl3)
200 190 180 170 160 150 140 130 120 110 100 90 80 70 60 50 40 30 20 10 0 ppm

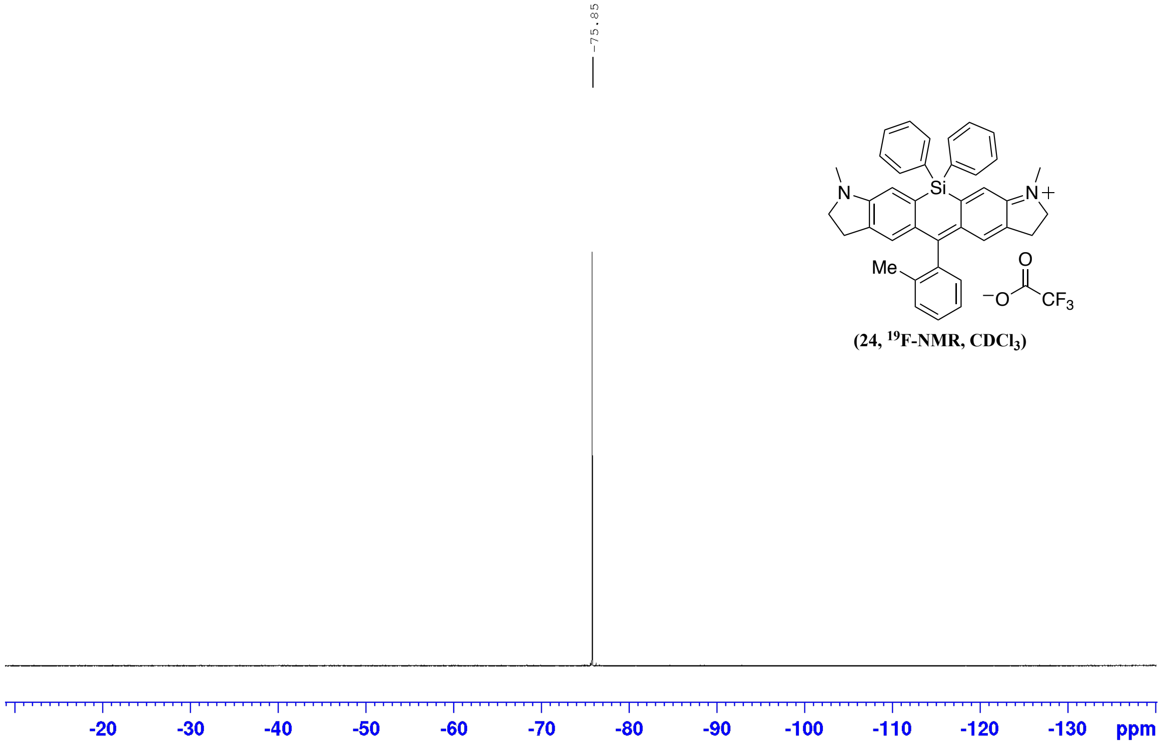
-75.85
Si
N
N+
Me
O
−O
CF3
(24, 19F-NMR, CDCl3)
-20 -30 -40 -50 -60 -70 -80 -90 -100 -110 -120 -130 ppm

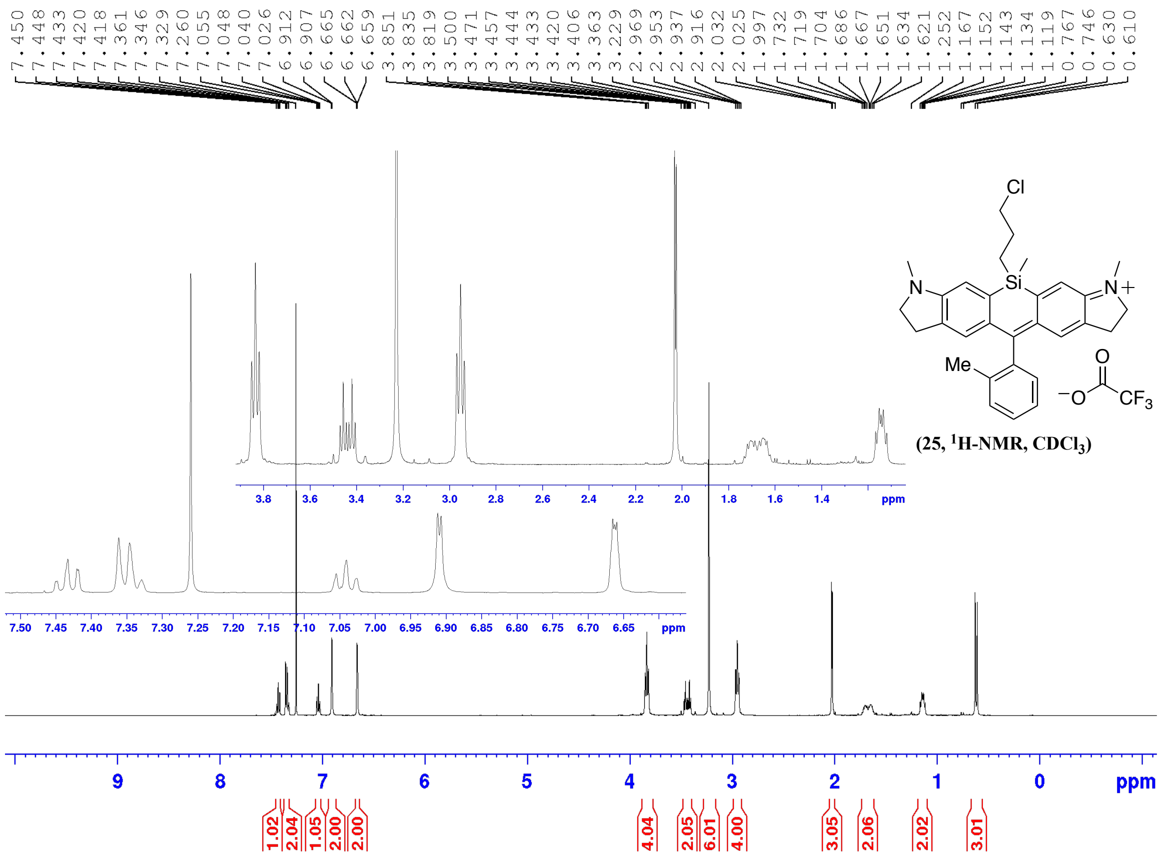
7.450 7.448 7.433 7.420 7.418 7.361 7.346 7.329 7.260 7.055 7.048 7.040 7.026 6.912 6.907 6.665 6.662 6.659 3.851 3.835 3.819 3.500 3.471 3.457 3.444 3.433 3.420 3.406 3.363 3.229 2.969 2.953 2.937 2.916 2.032 2.025 1.997 1.732 1.719 1.704 1.686 1.667 1.651 1.634 1.621 1.252 1.167 1.152 1.143 1.134 1.119 0.767 0.746 0.630 0.610
1.02 2.04 1.05 2.00 2.00 4.04 2.05 6.01 4.00 3.05 2.06 2.02 3.01
ppm
(25, ¹H-NMR, CDCl₃)

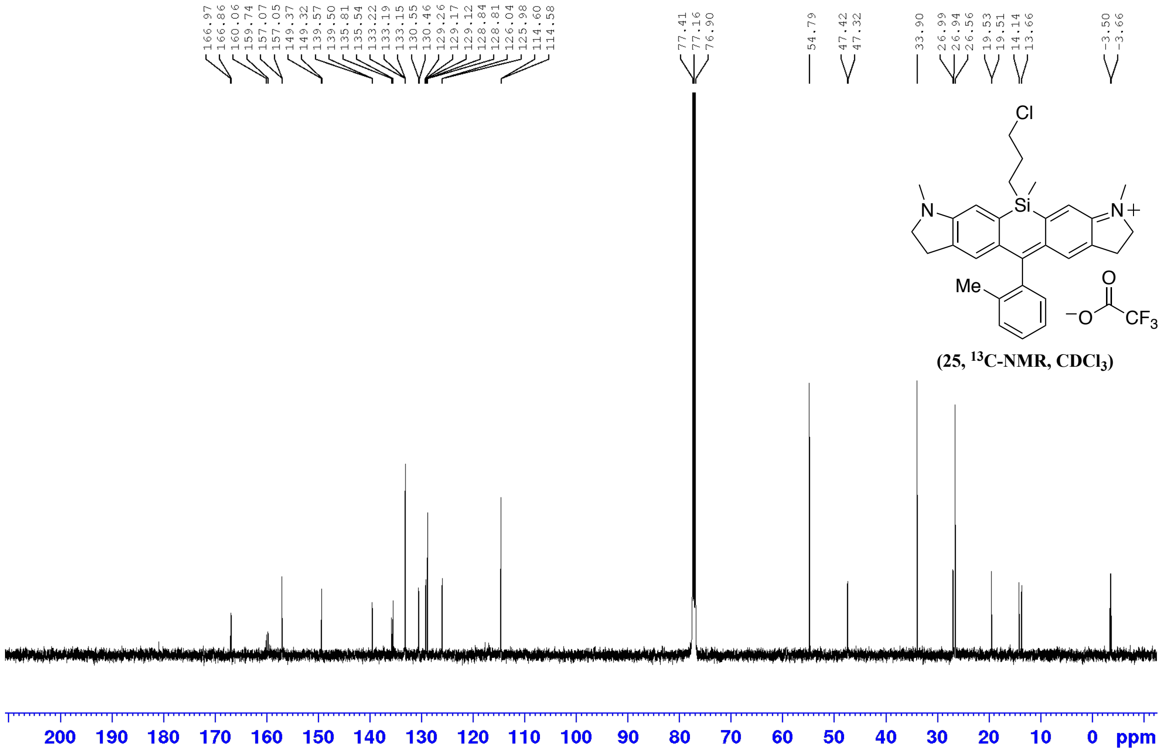
166.97 166.86 160.06 159.74 157.07 157.05 149.37 149.32 139.57 139.50 135.81 135.54 133.22 133.19 133.15 130.55 130.46 129.26 129.17 129.12 128.84 128.81 126.04 125.98 114.60 114.58 77.41 77.16 76.90 54.79 47.42 47.32 33.90 26.99 26.94 26.56 19.53 19.51 14.14 13.66 -3.50 -3.66
ppm
(25, ¹³C-NMR, CDCl₃)

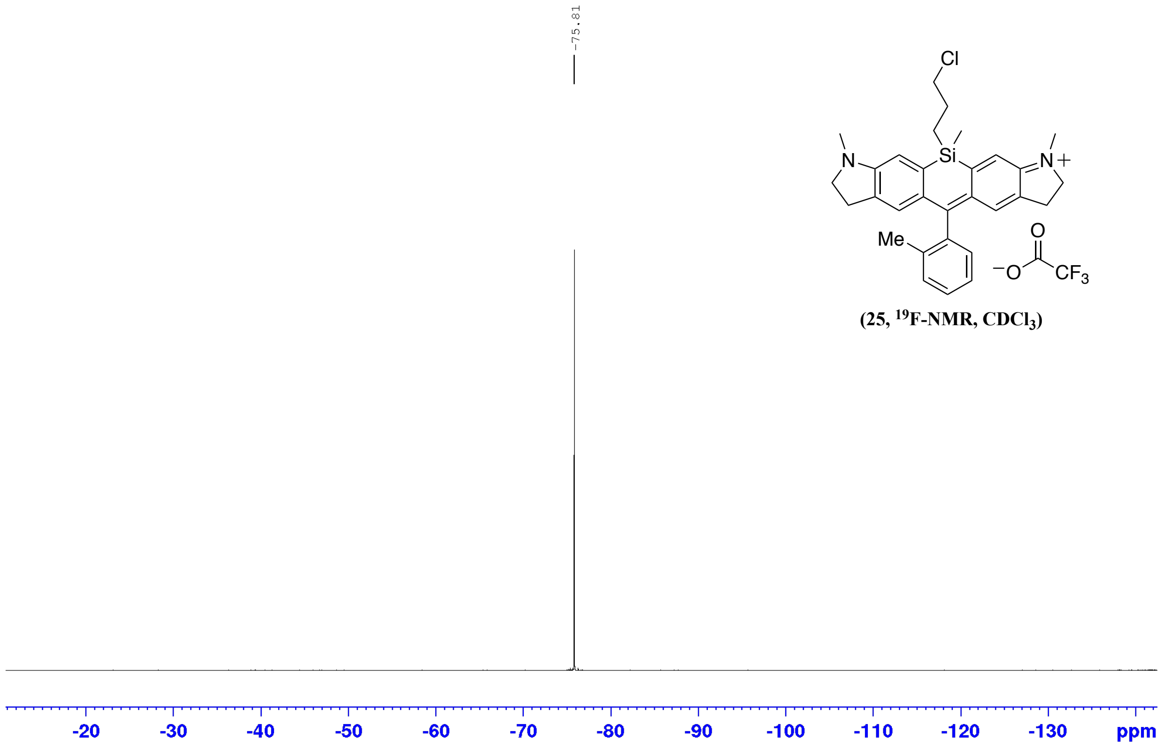

(25, ^{19}F-NMR, $CDCl_3$)

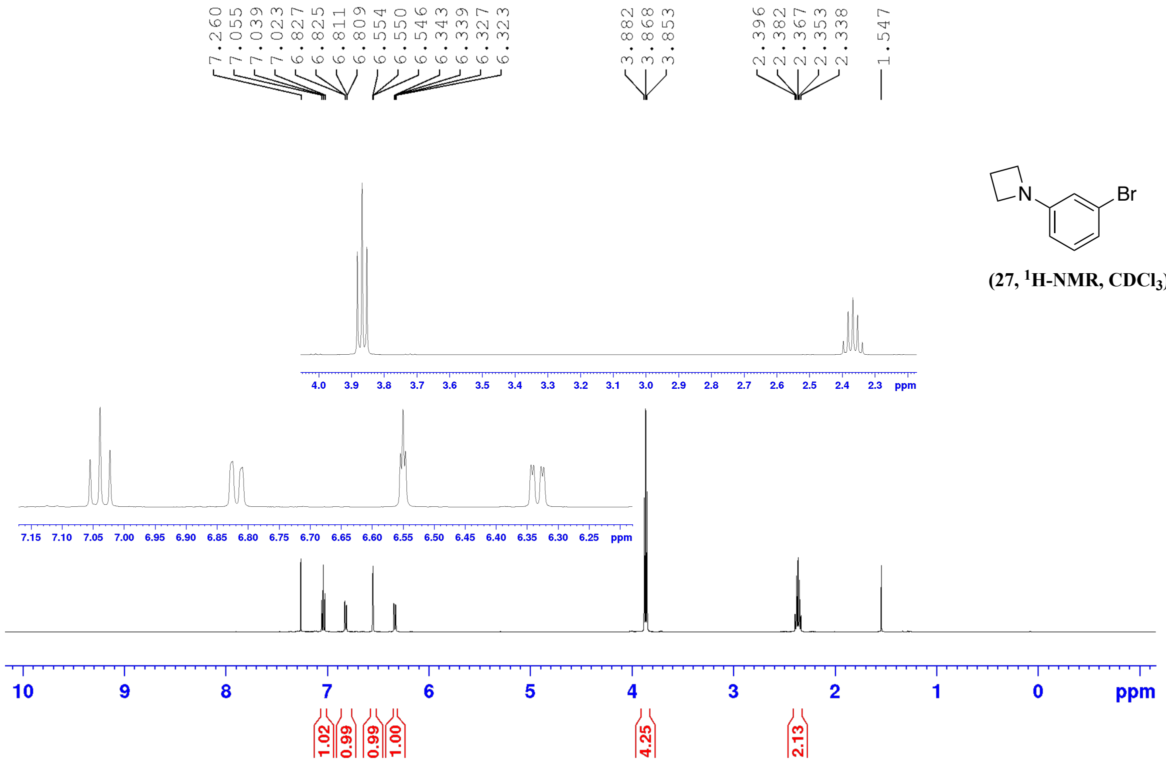

(27, ^{1}H-NMR, $CDCl_3$)

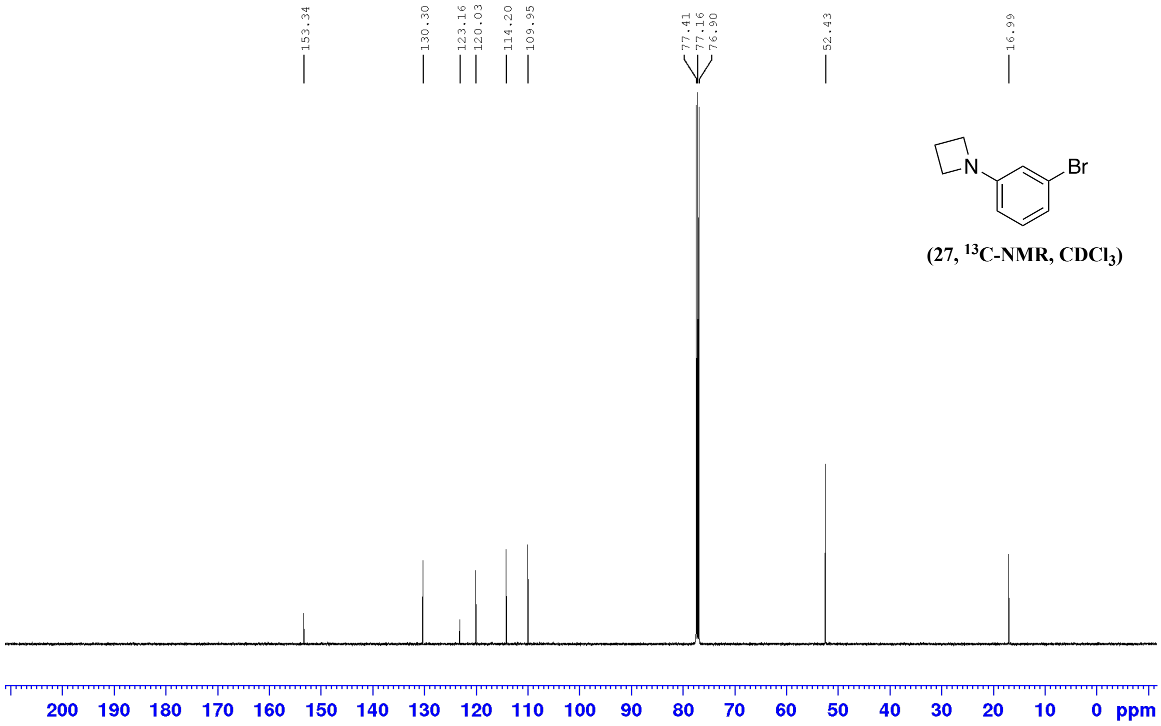

(27, ^{13}C-NMR, $CDCl_3$)

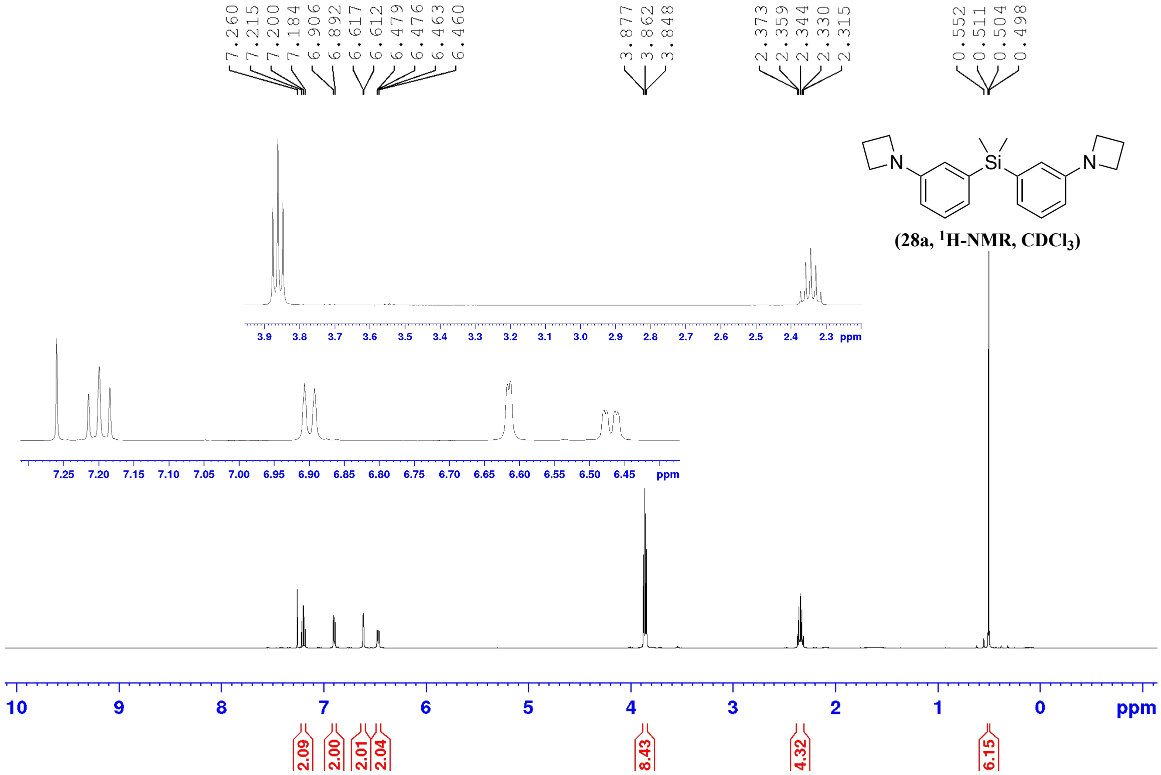

(28a, ^{1}H-NMR, $CDCl_3$)

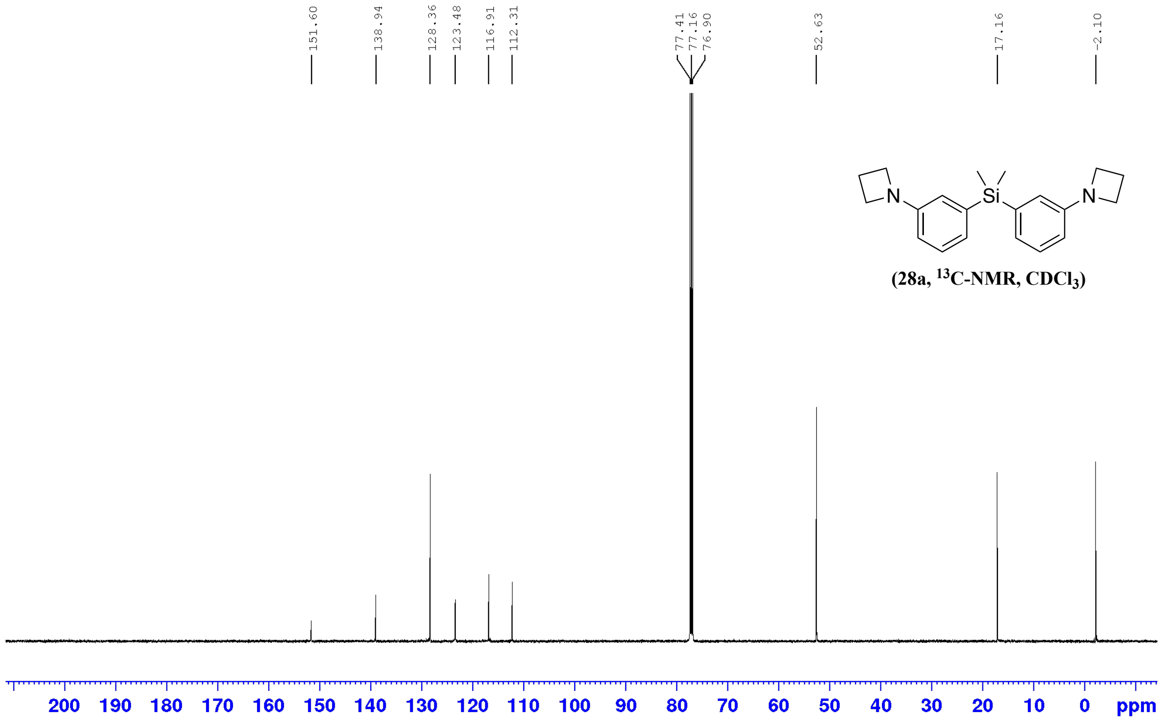
151.60
138.94
128.36
123.48
116.91
112.31
77.41
77.16
76.90
52.63
17.16
-2.10
(28a, ^{13}C-NMR, CDCl$_3$)
200 190 180 170 160 150 140 130 120 110 100 90 80 70 60 50 40 30 20 10 0 ppm

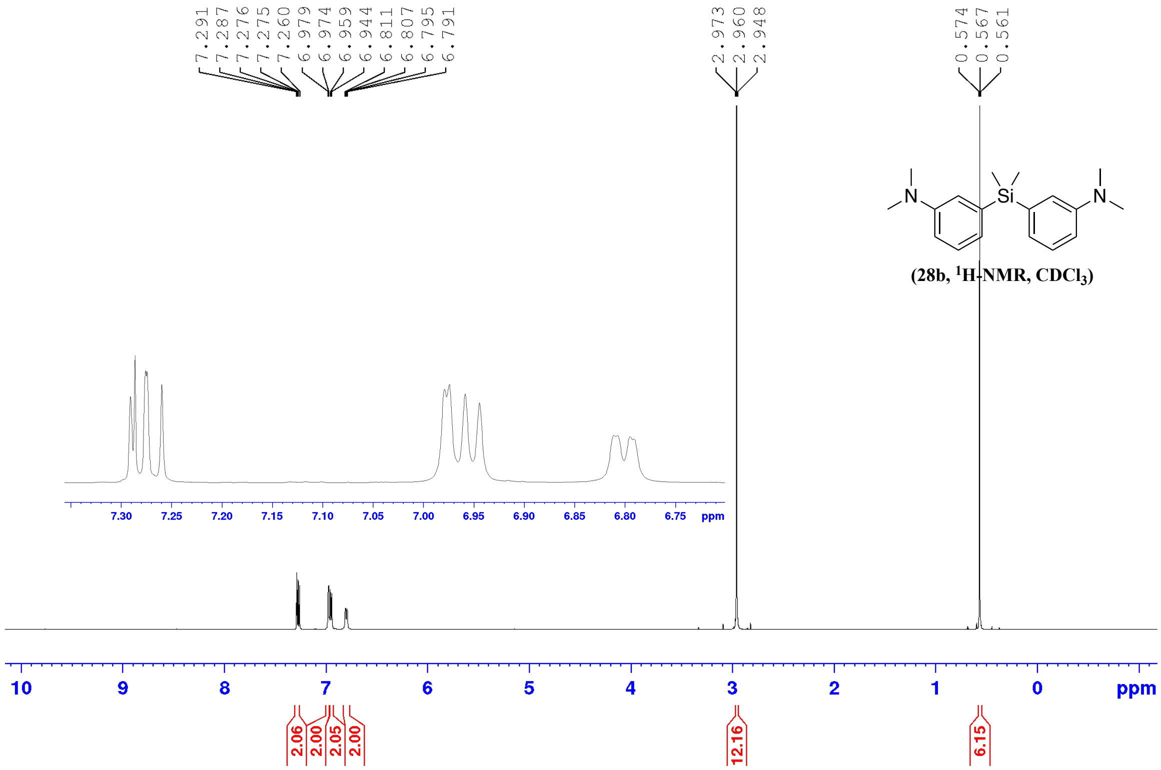
7.291
7.287
7.276
7.275
7.260
6.979
6.974
6.959
6.944
6.811
6.807
6.795
6.791
2.973
2.960
2.948
0.574
0.567
0.561
(28b, ^{1}H-NMR, CDCl$_3$)
7.30 7.25 7.20 7.15 7.10 7.05 7.00 6.95 6.90 6.85 6.80 6.75 ppm
10 9 8 7 6 5 4 3 2 1 0 ppm
2.06
2.00
2.05
2.00
12.16
6.15

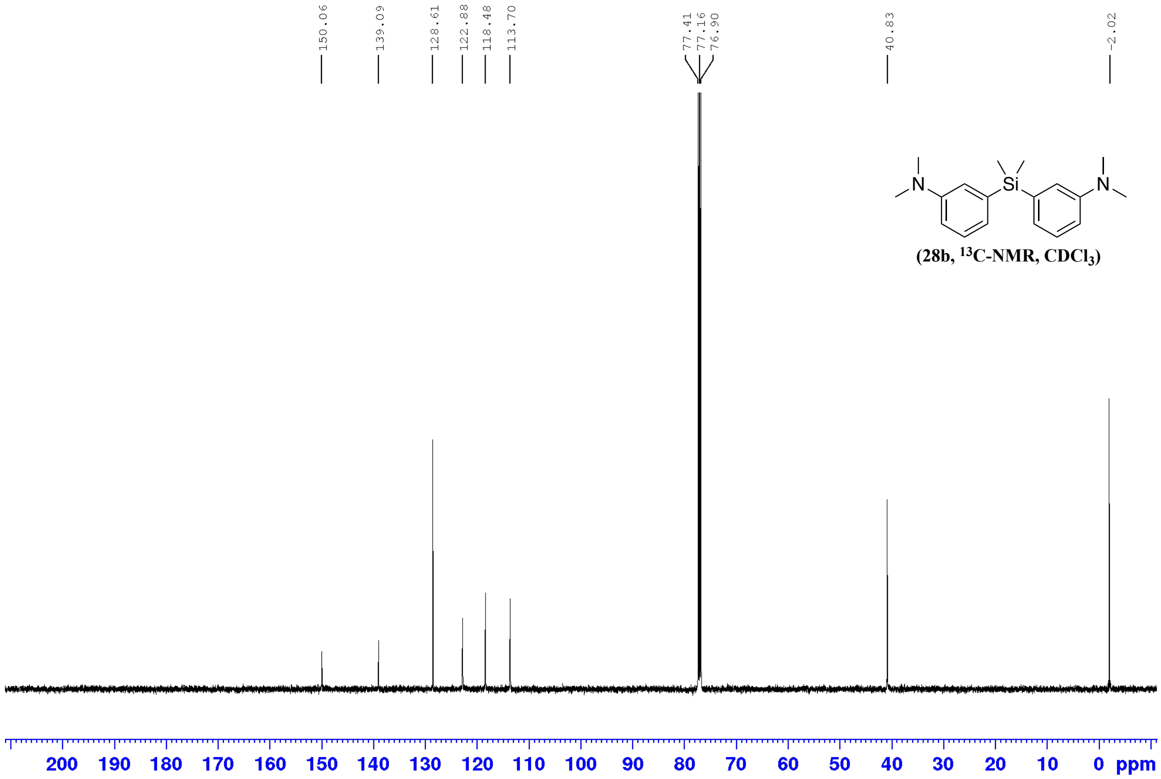
150.06
139.09
128.61
122.88
118.48
113.70
77.41
77.16
76.90
40.83
-2.02
(28b, 13C-NMR, CDCl3)
ppm

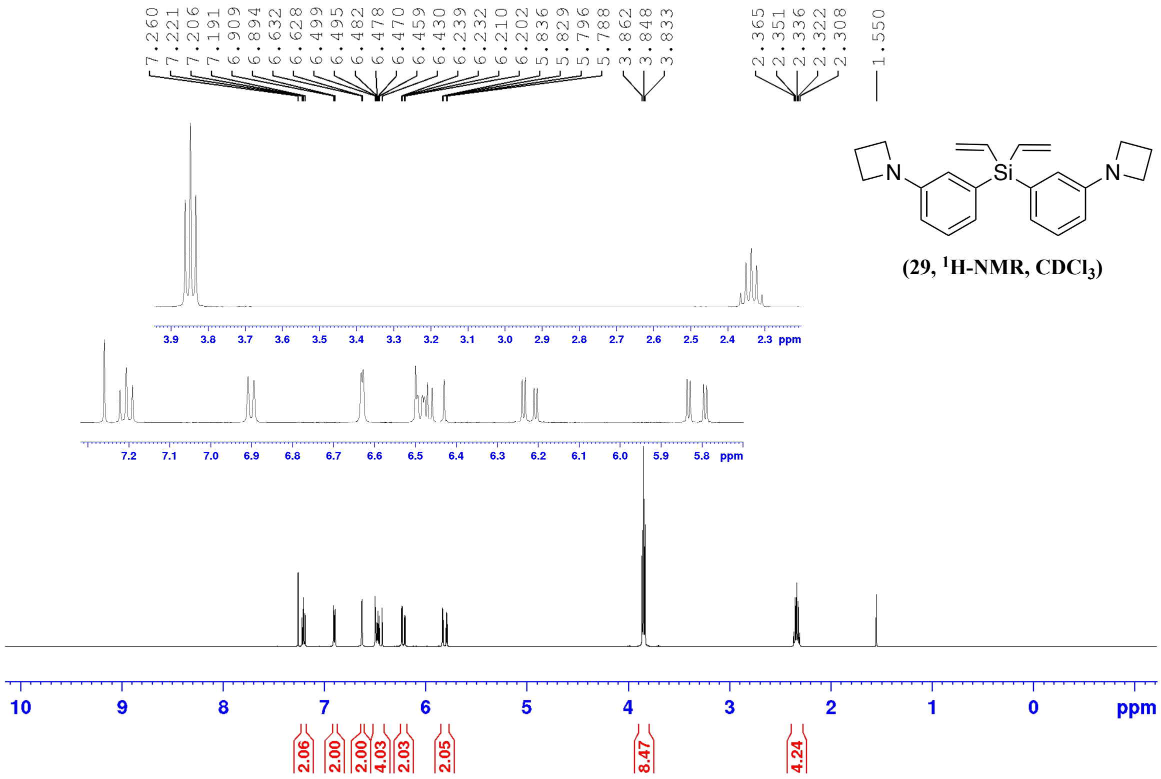
7.260
7.221
7.206
7.191
6.909
6.894
6.632
6.628
6.499
6.495
6.482
6.478
6.470
6.459
6.430
6.239
6.232
6.210
6.202
5.836
5.829
5.796
5.788
3.862
3.848
3.833
2.365
2.351
2.336
2.322
2.308
1.550
(29, 1H-NMR, CDCl3)
2.06
2.00
2.00
4.03
2.03
2.05
8.47
4.24
ppm

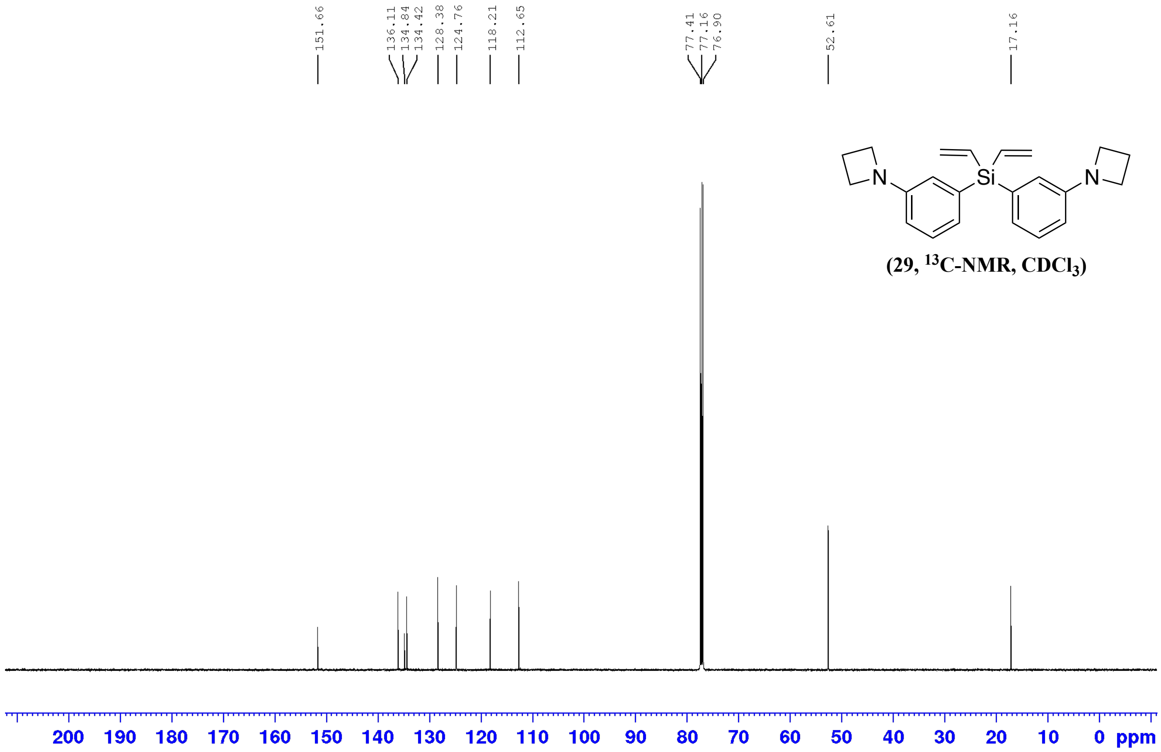

(29, ^{13}C-NMR, $CDCl_3$)

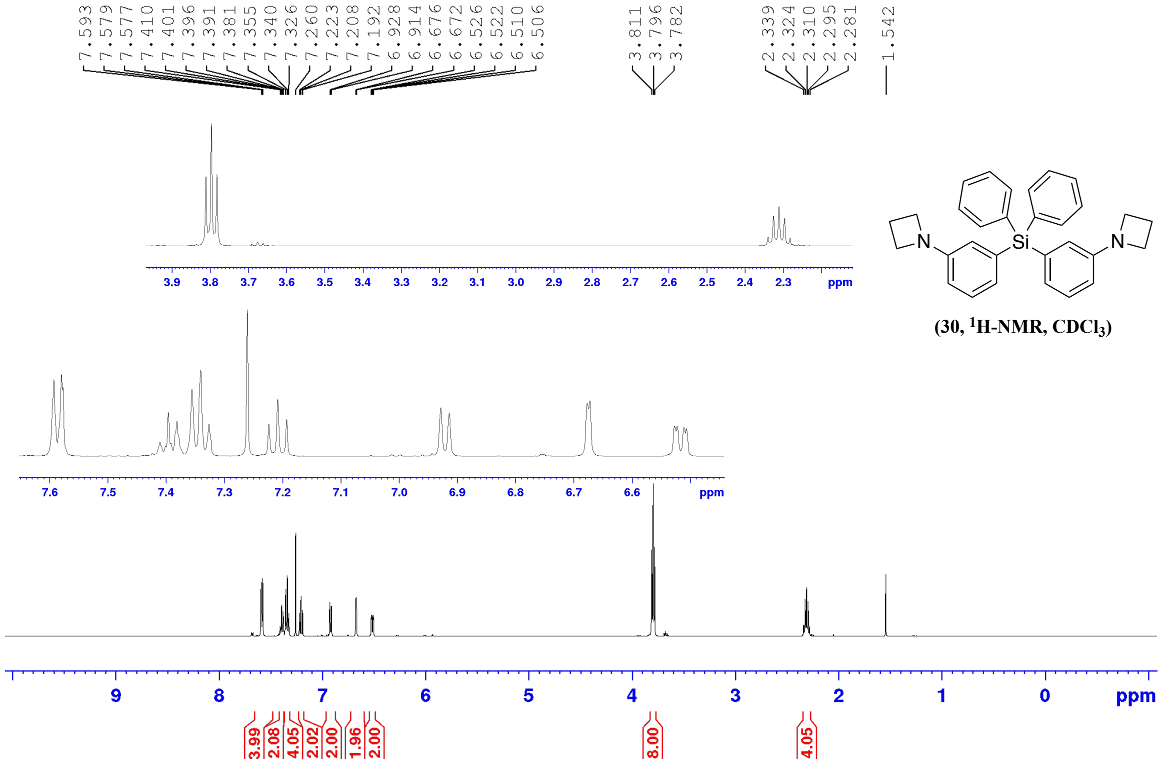

(30, ^{1}H-NMR, $CDCl_3$)

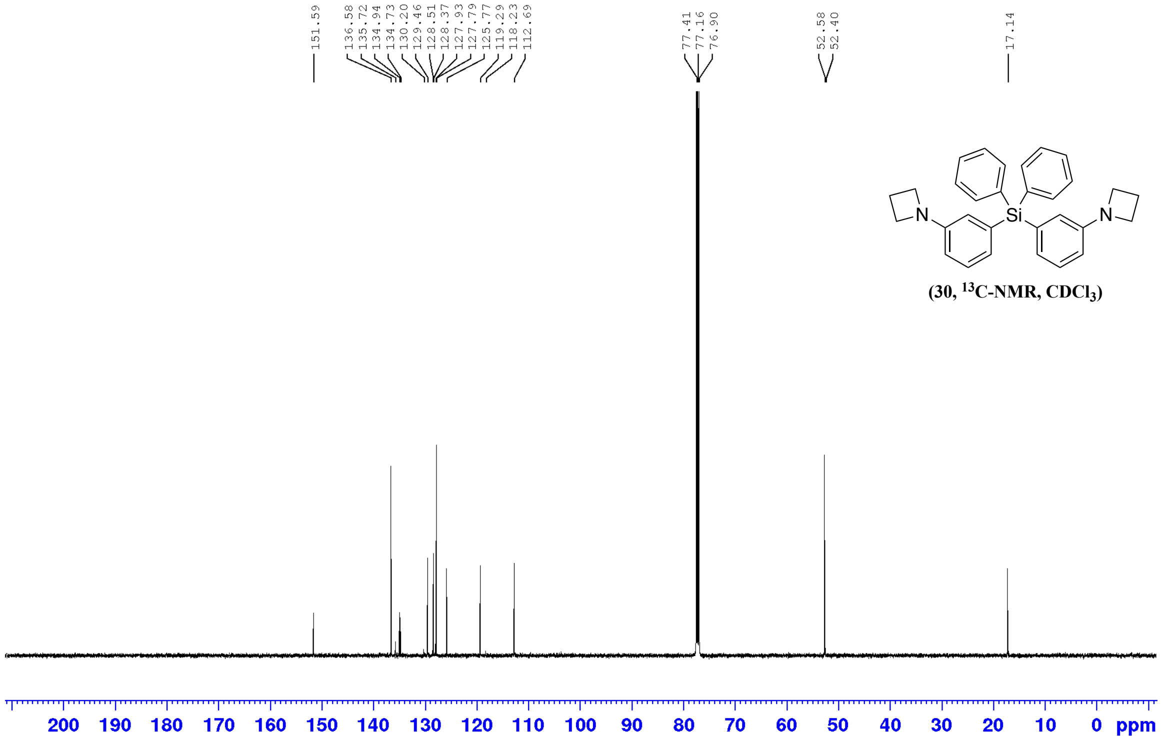

(30, ^{13}C-NMR, $CDCl_3$)

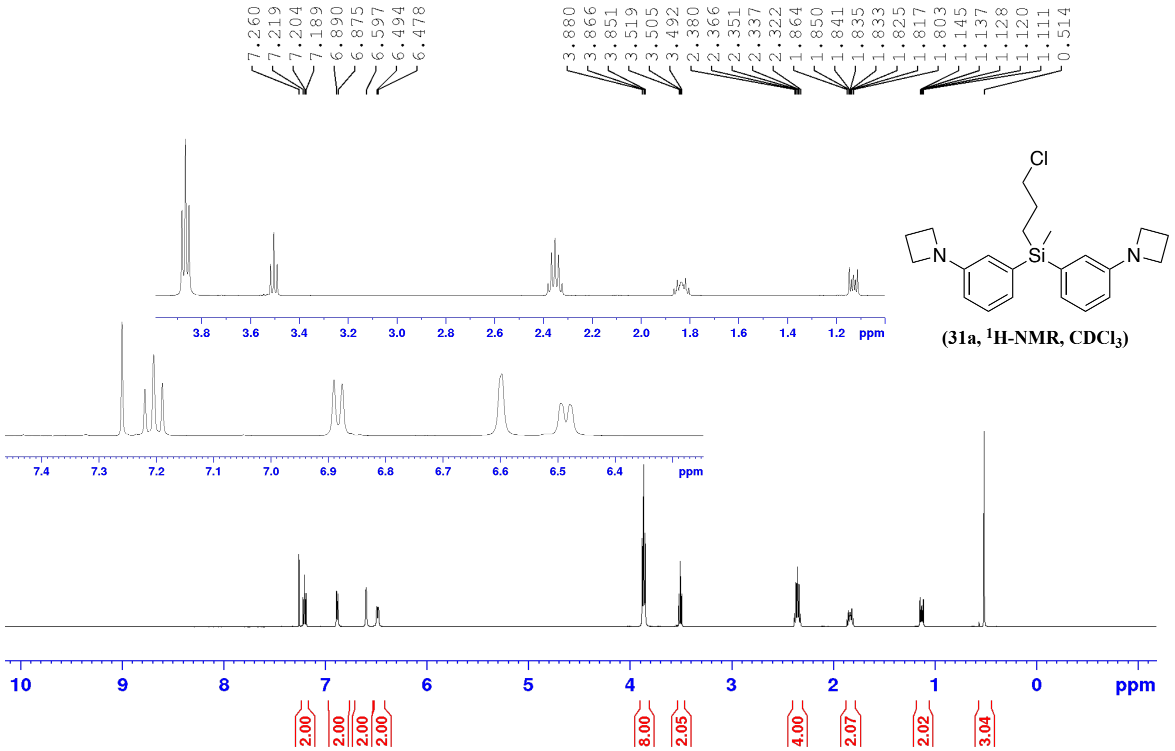

(31a, ^{1}H-NMR, $CDCl_3$)

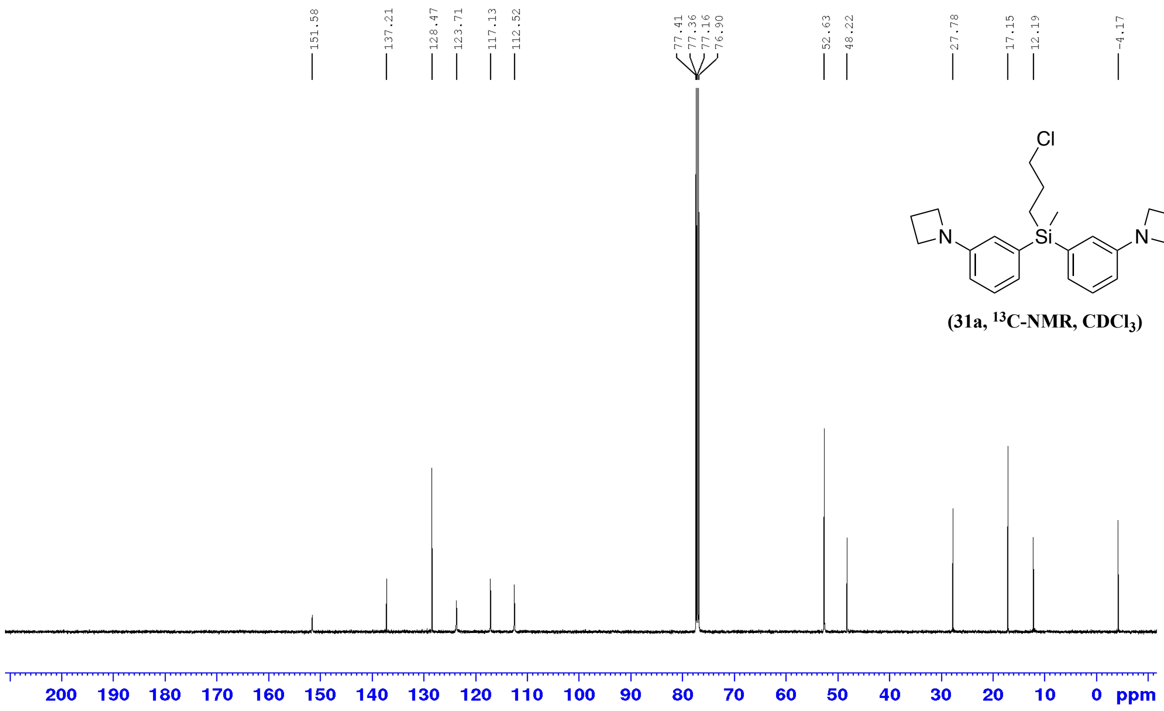

(31a, ^{13}C-NMR, $CDCl_3$)

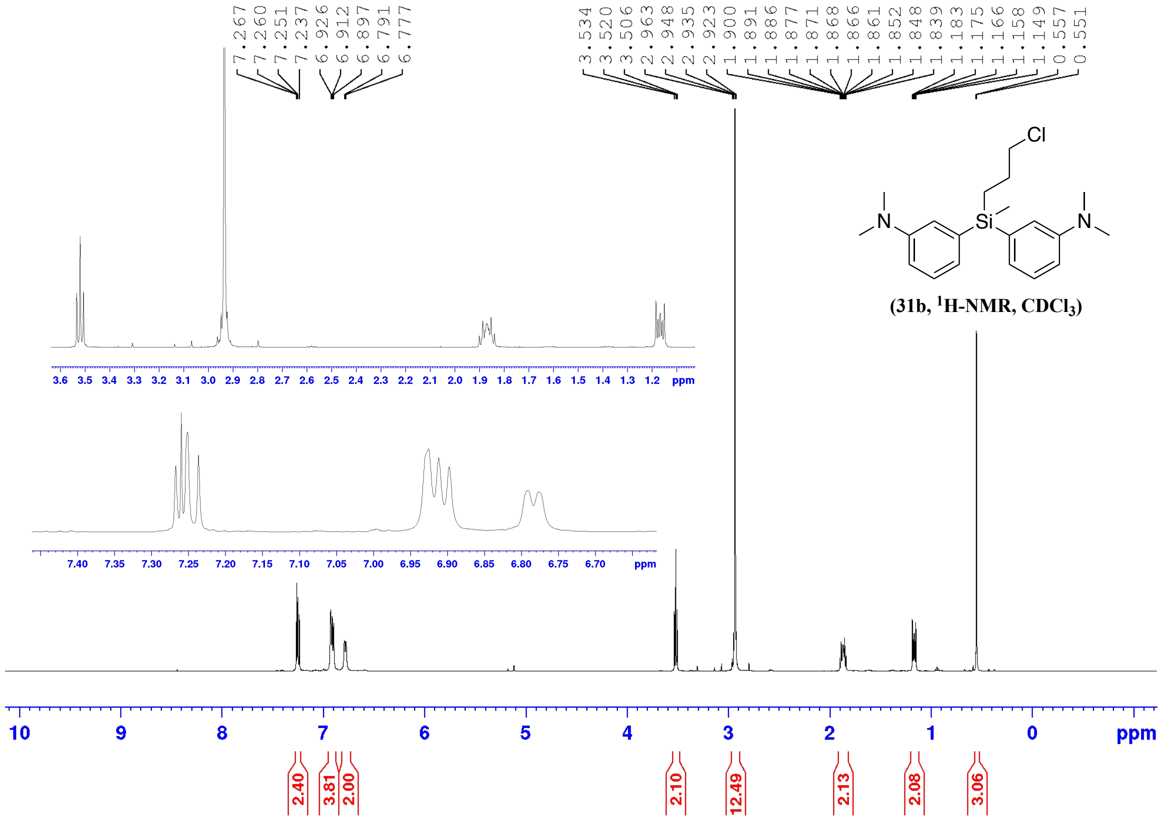

(31b, ^{1}H-NMR, $CDCl_3$)

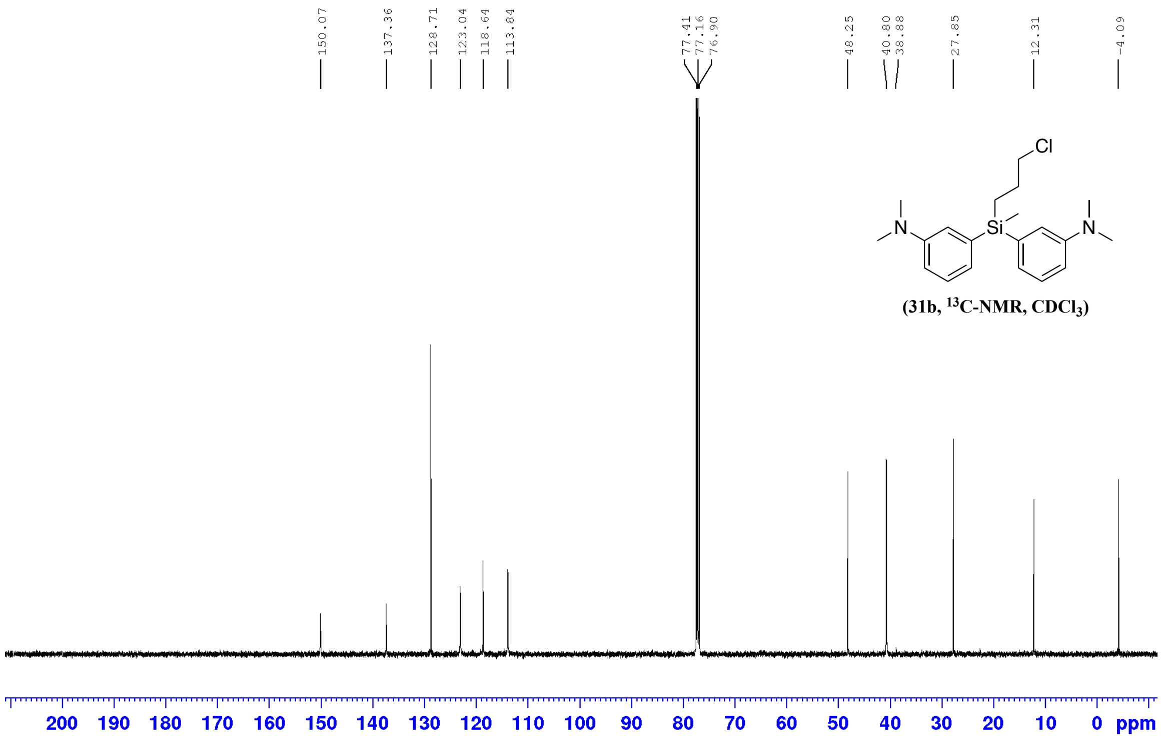

(31b, ^{13}C-NMR, $CDCl_3$)

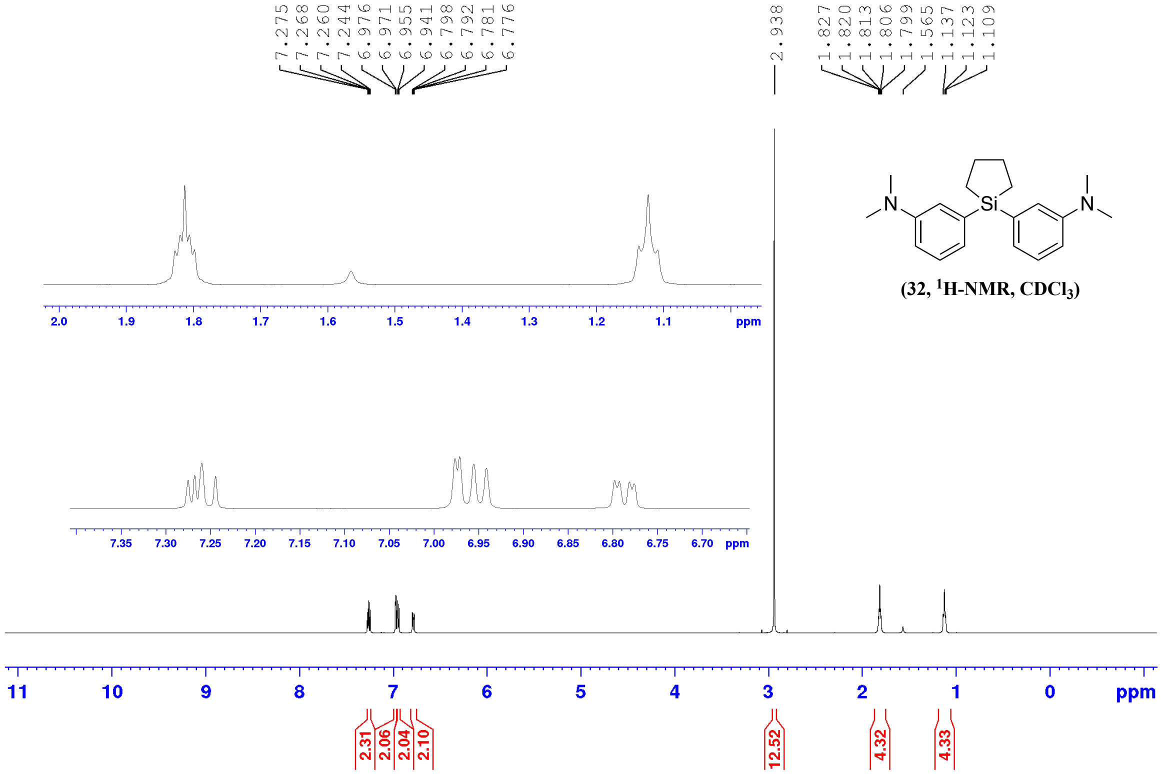

(32, ^{1}H-NMR, $CDCl_3$)

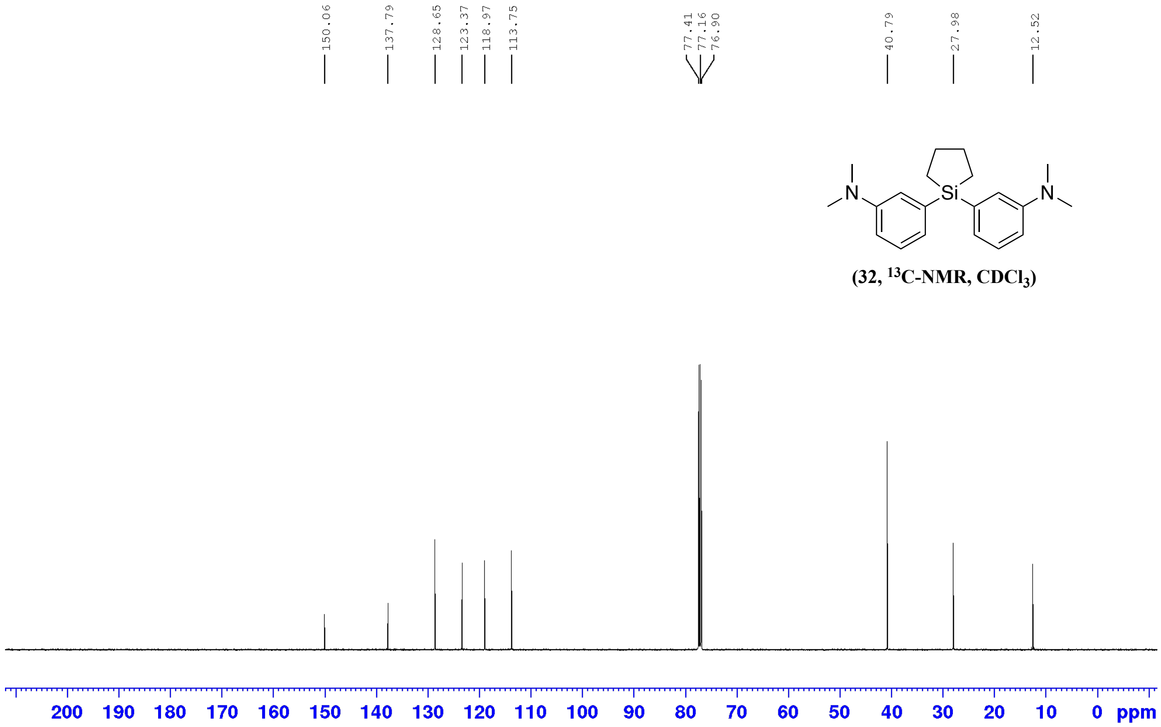

(32, ^{13}C-NMR, $CDCl_3$)

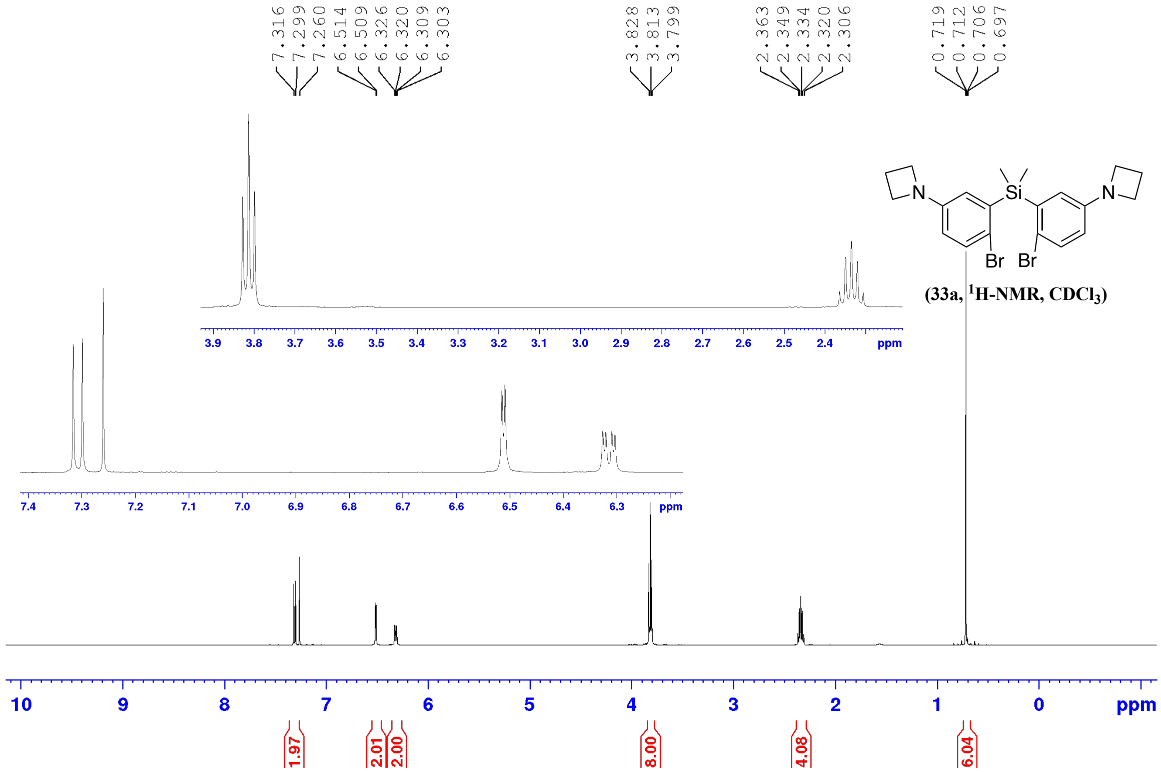

(33a, ^{1}H-NMR, $CDCl_3$)

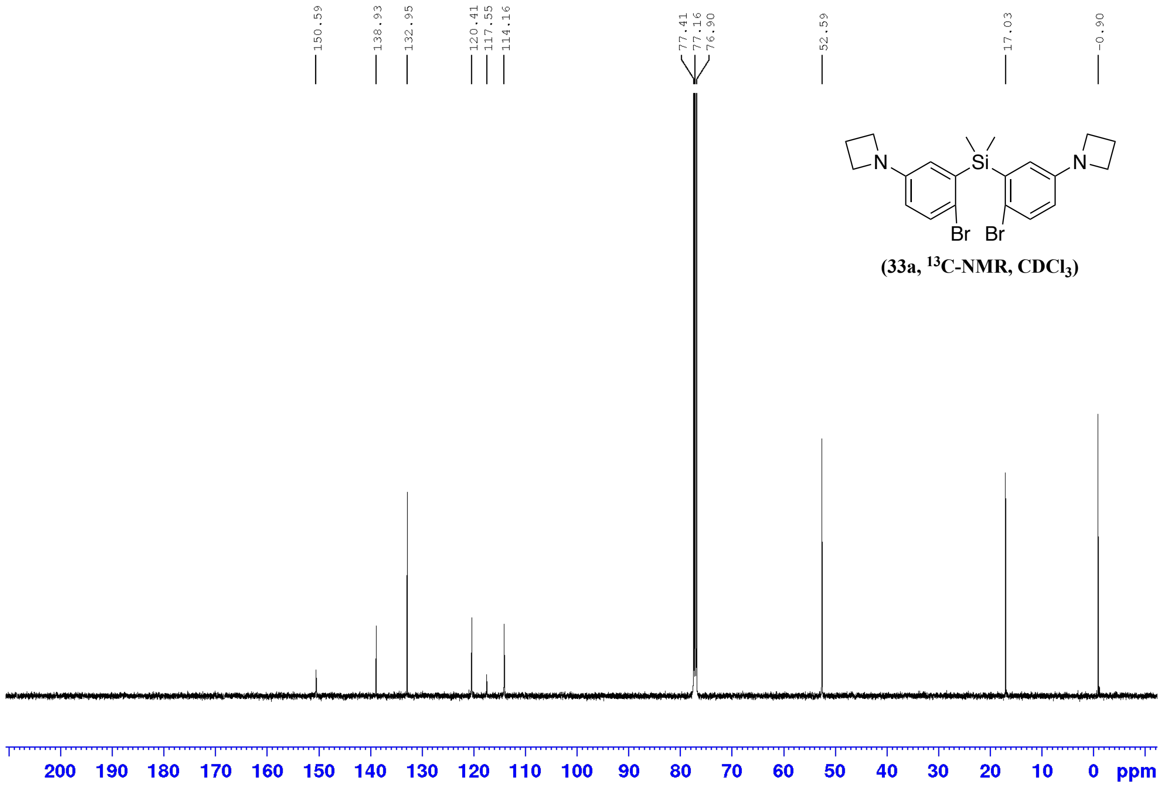
150.59
138.93
132.95
120.41
117.55
114.16
77.41
77.16
76.90
52.59
17.03
-0.90
Si
N
N
Br Br
(33a, ^{13}C-NMR, $CDCl_3$)
200 190 180 170 160 150 140 130 120 110 100 90 80 70 60 50 40 30 20 10 0 ppm

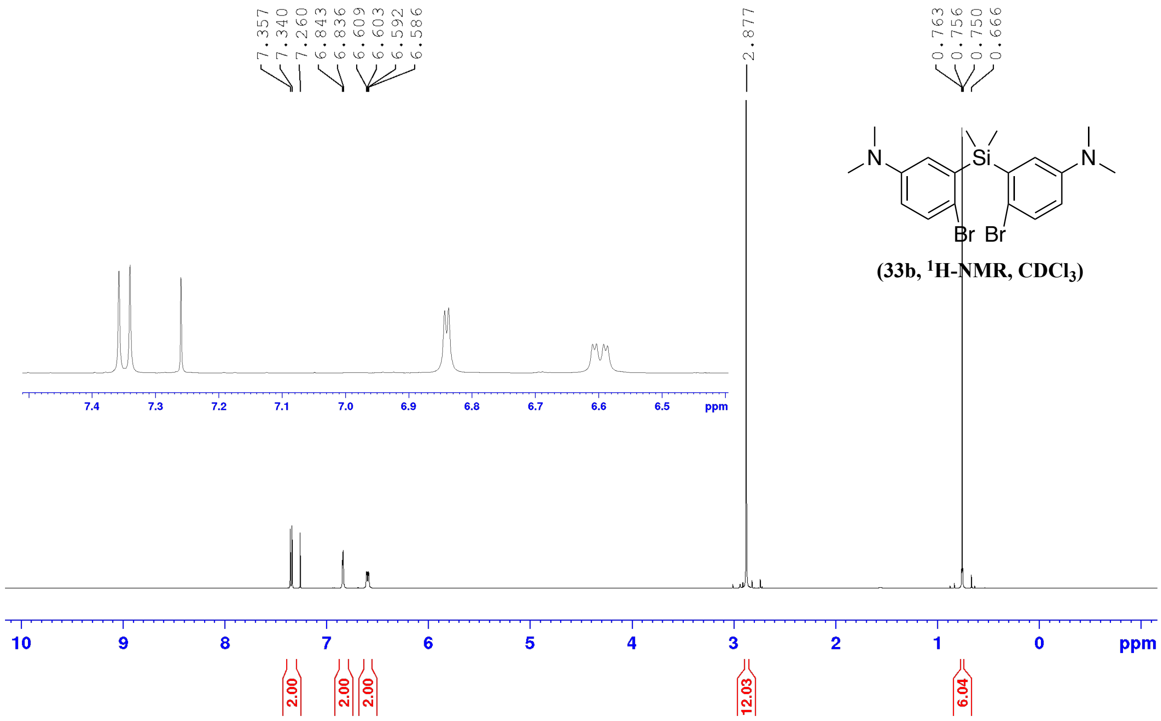
7.357
7.340
7.260
6.843
6.836
6.609
6.603
6.592
6.586
2.877
0.763
0.756
0.750
0.666
Si
N
N
Br Br
(33b, ^{1}H-NMR, $CDCl_3$)
7.4 7.3 7.2 7.1 7.0 6.9 6.8 6.7 6.6 6.5 ppm
10 9 8 7 6 5 4 3 2 1 0 ppm
2.00
2.00
2.00
12.03
6.04

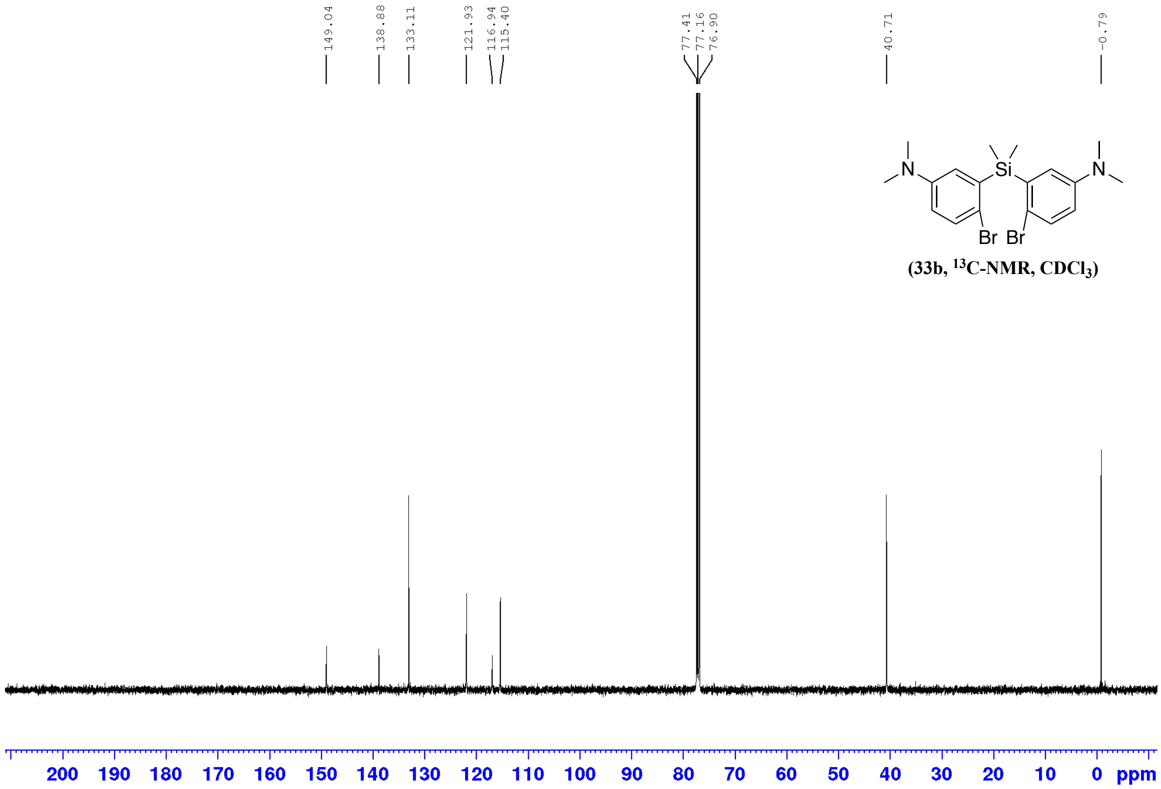

(33b, ^{13}C-NMR, $CDCl_3$)

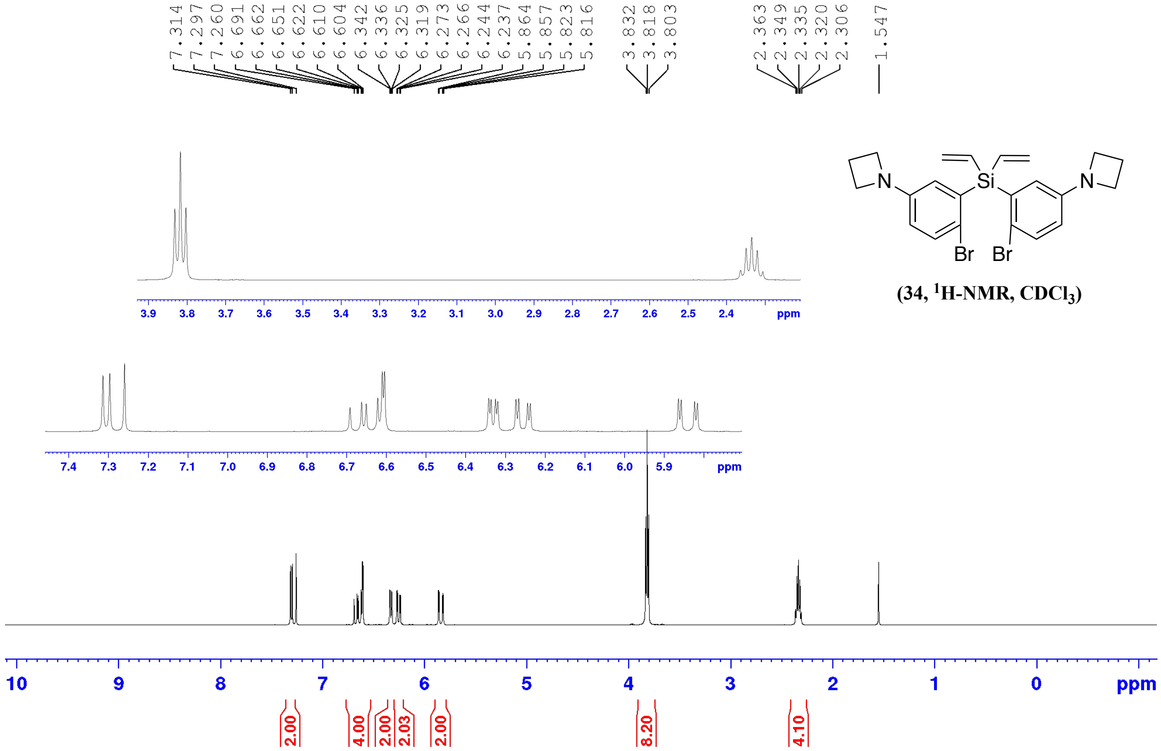

(34, ^{1}H-NMR, $CDCl_3$)

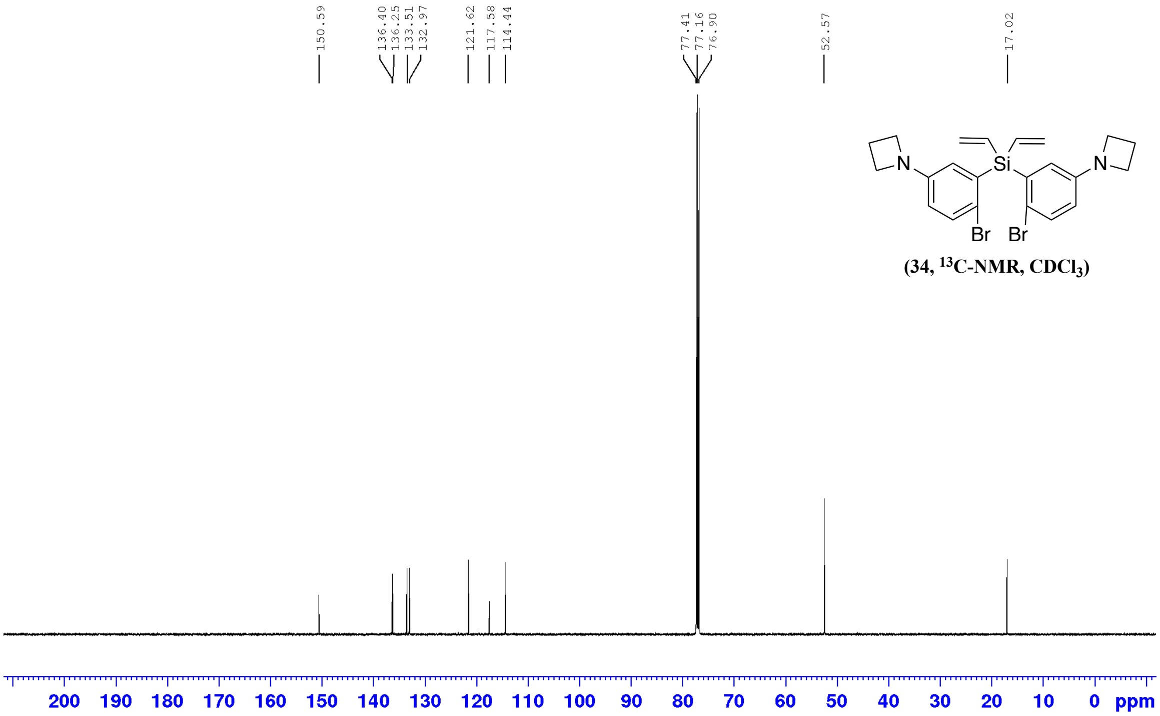

(34, ^{13}C-NMR, $CDCl_3$)

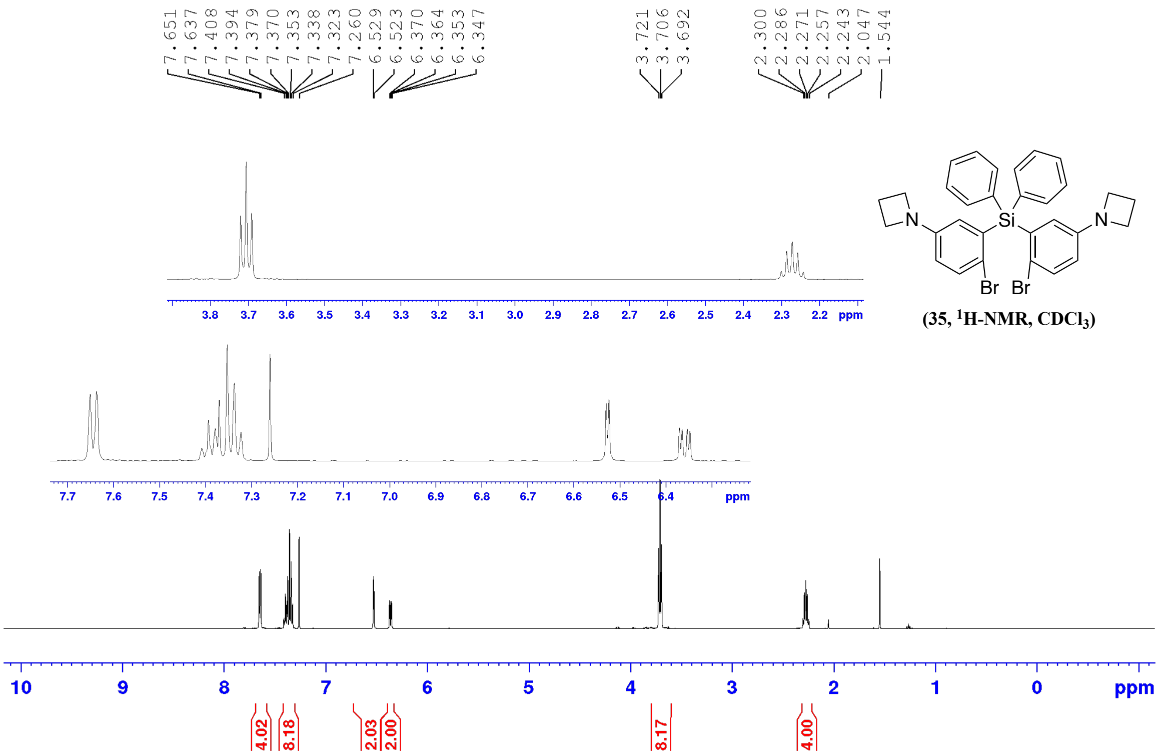

(35, ^{1}H-NMR, $CDCl_3$)

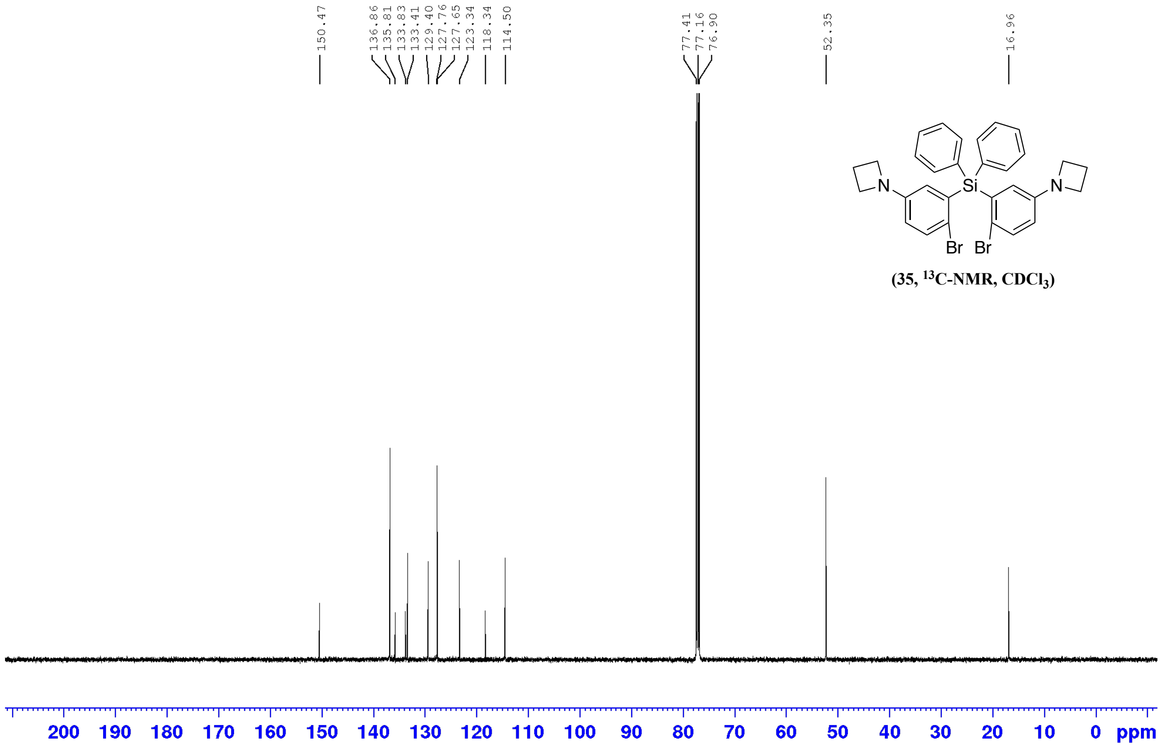

(35, ^{13}C-NMR, $CDCl_3$)

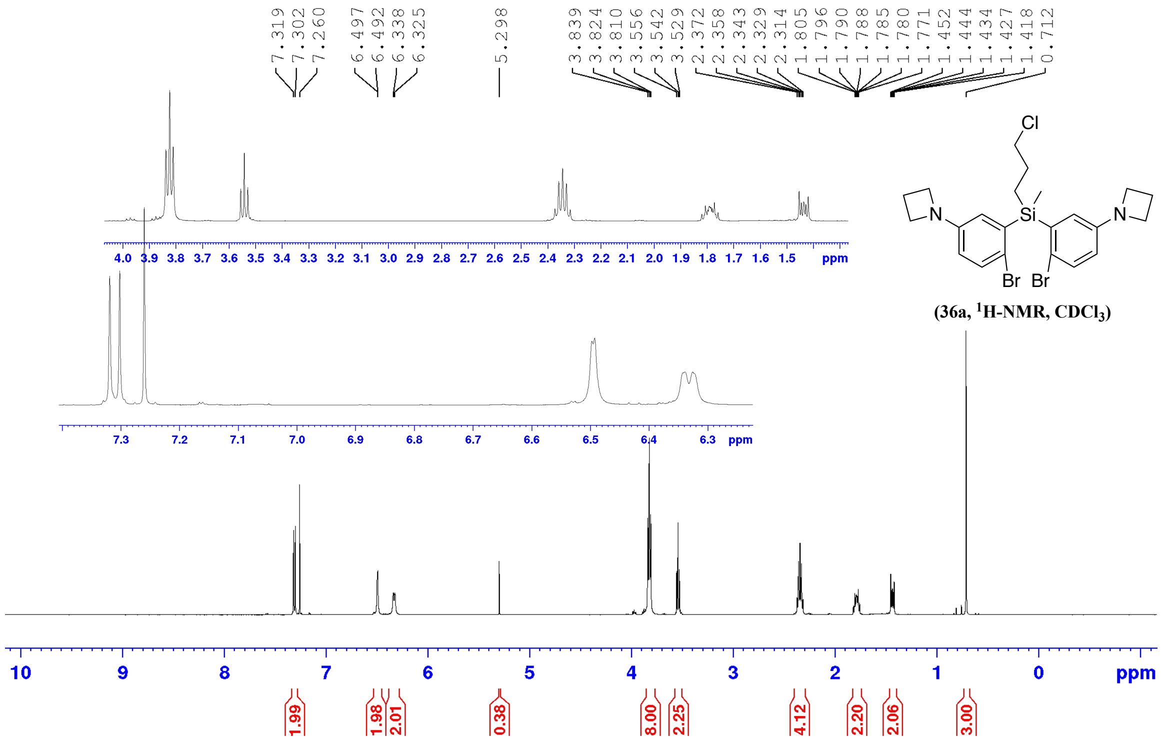

(36a, ^{1}H-NMR, $CDCl_3$)

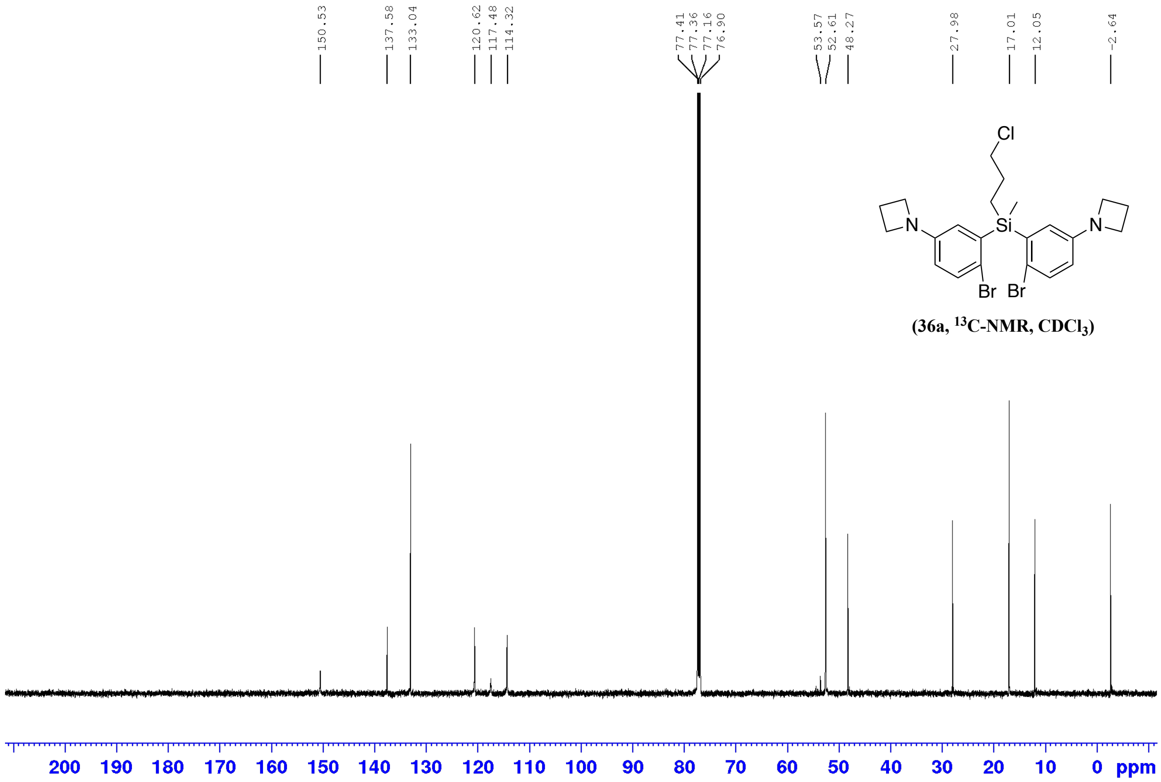

(36a, ^{13}C-NMR, $CDCl_3$)

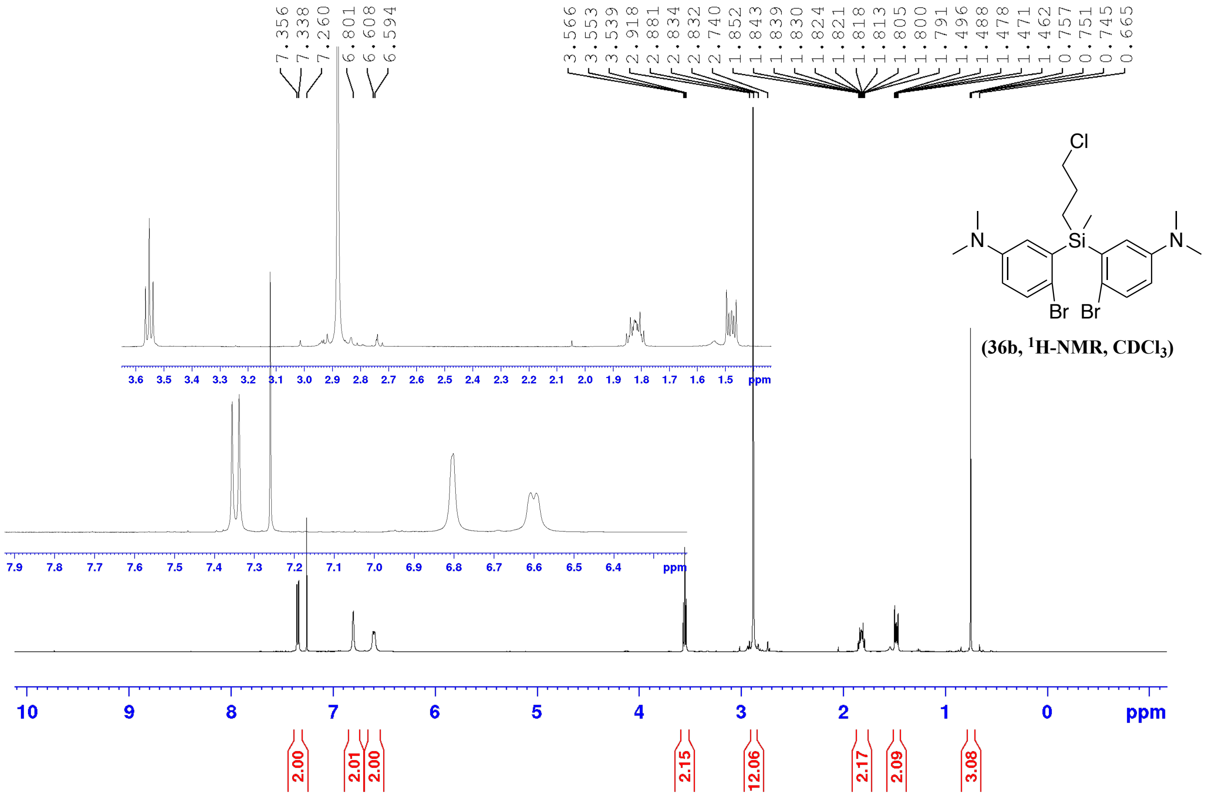

(36b, ^{1}H-NMR, $CDCl_3$)

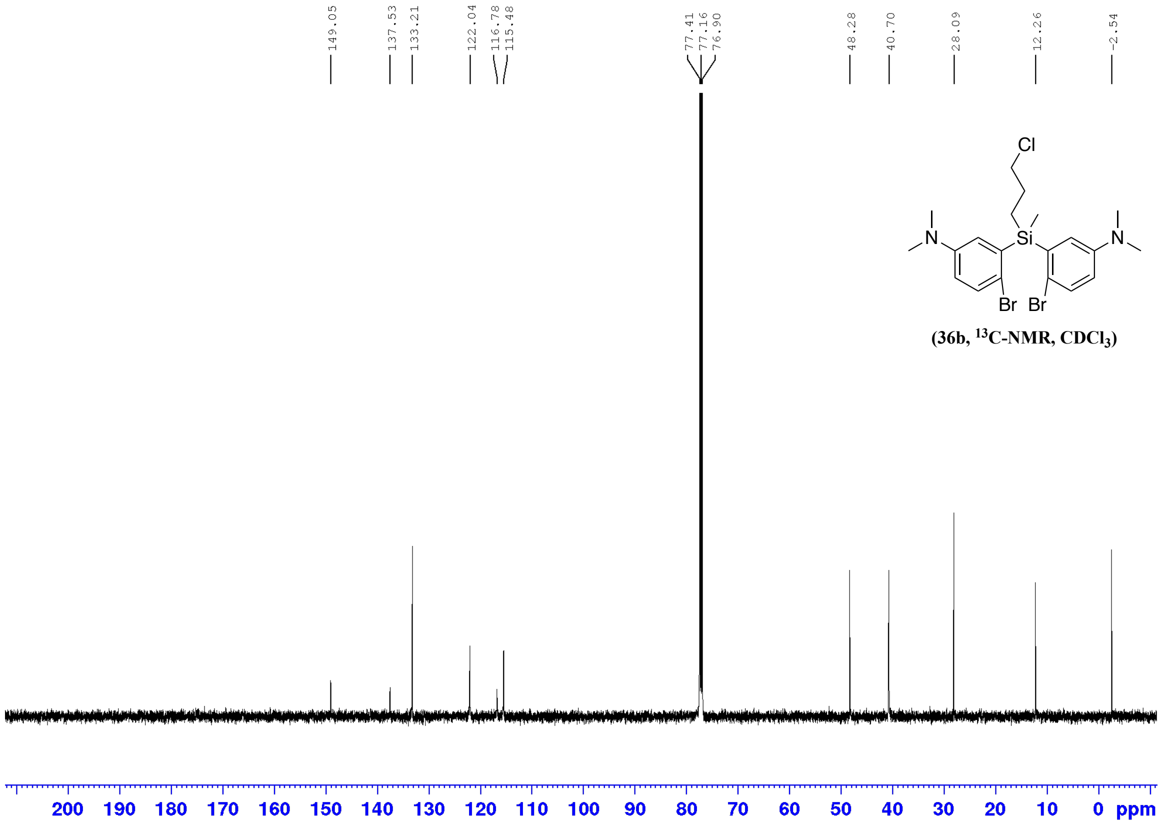

(36b, ^{13}C-NMR, $CDCl_3$)

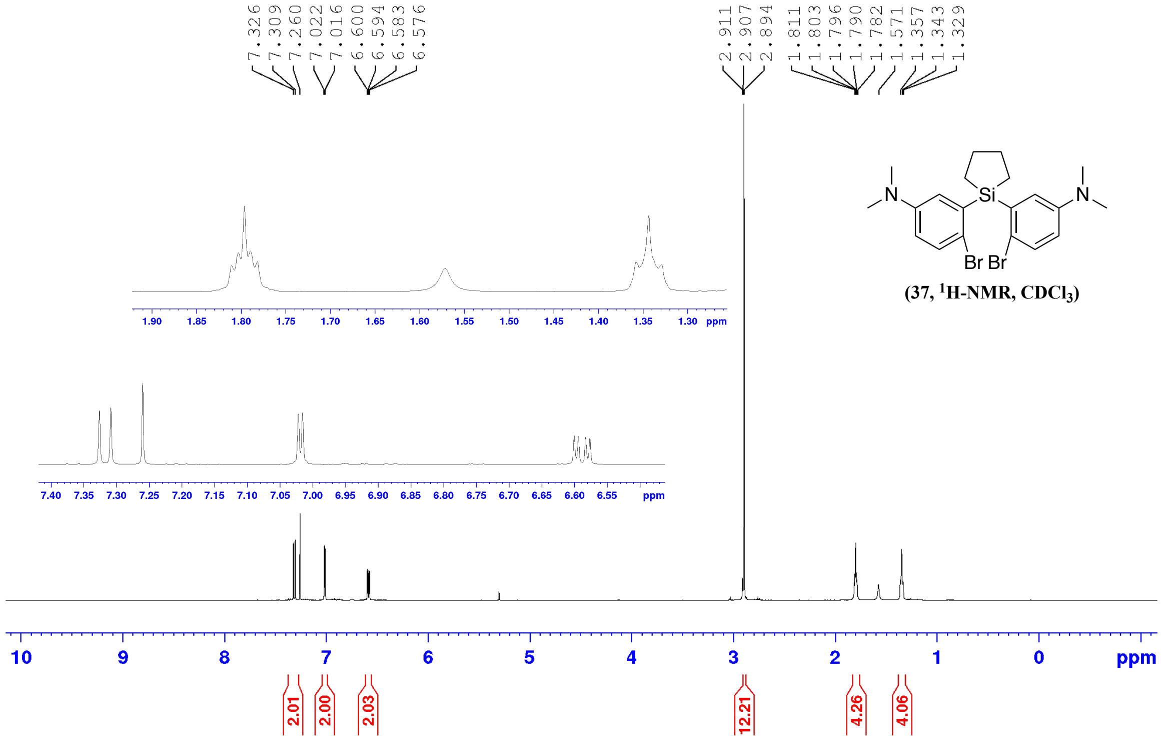

(37, ^{1}H-NMR, $CDCl_3$)

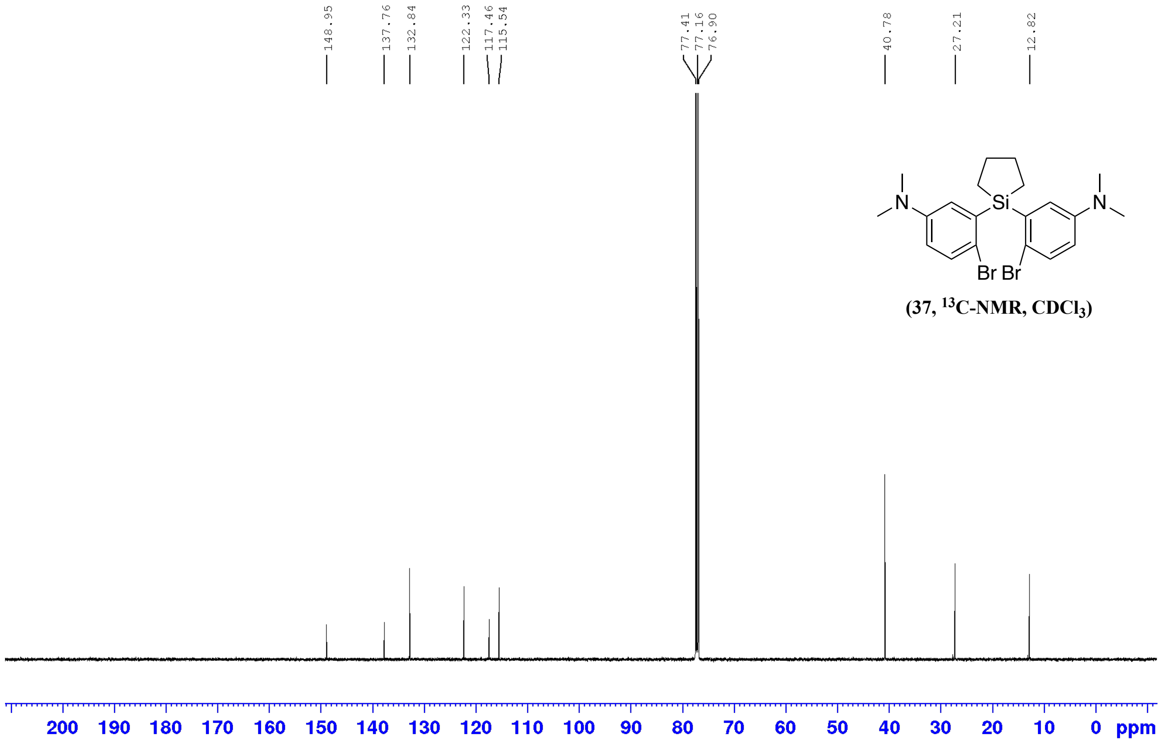

(37, ^{13}C-NMR, $CDCl_3$)

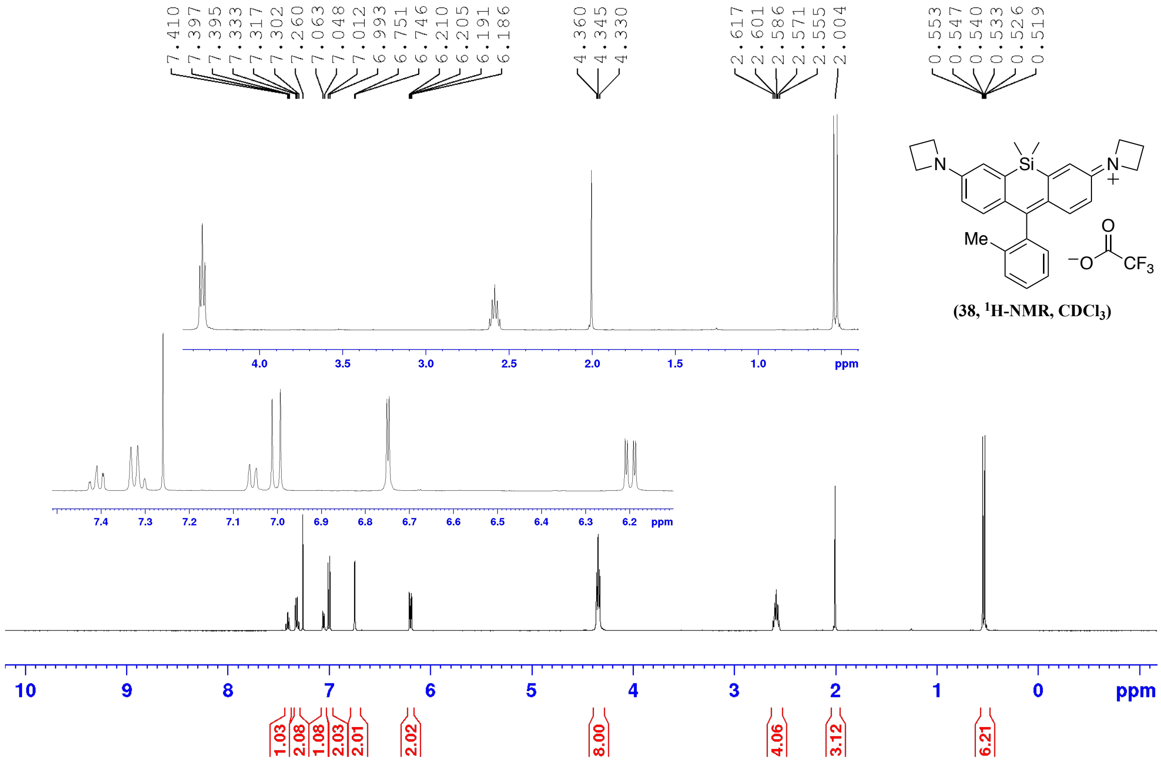

(38, ^{1}H-NMR, $CDCl_3$)

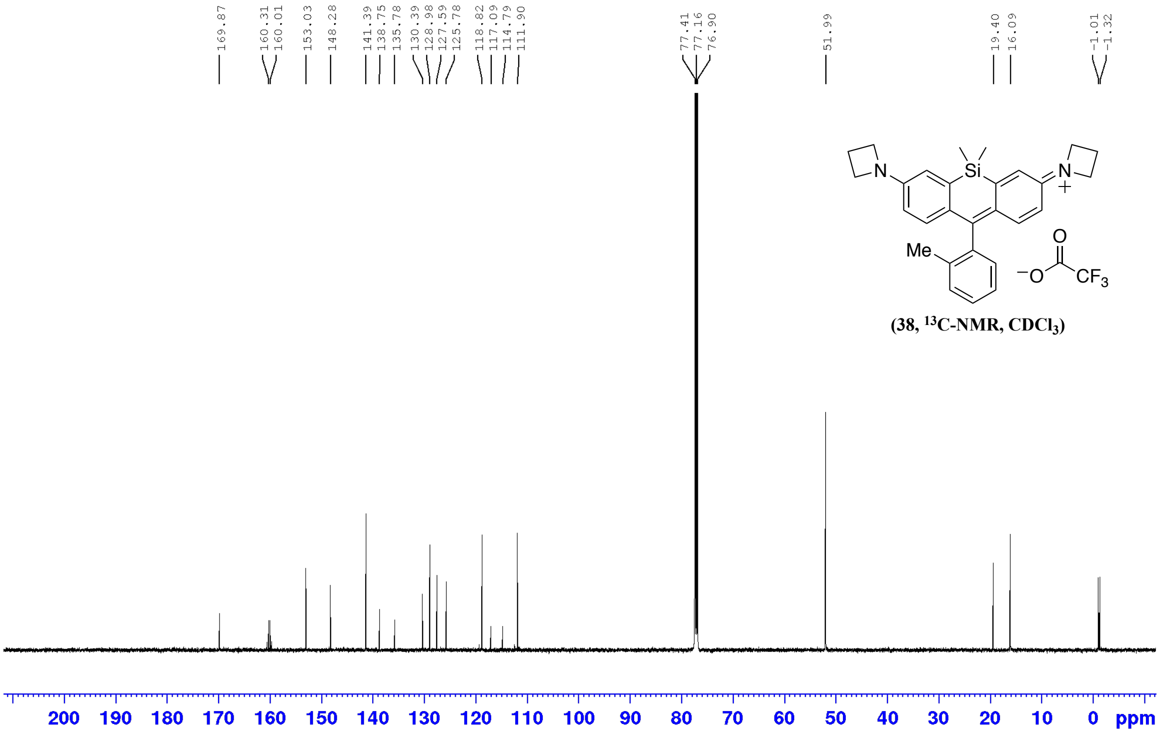

(38, ^{13}C-NMR, $CDCl_3$)

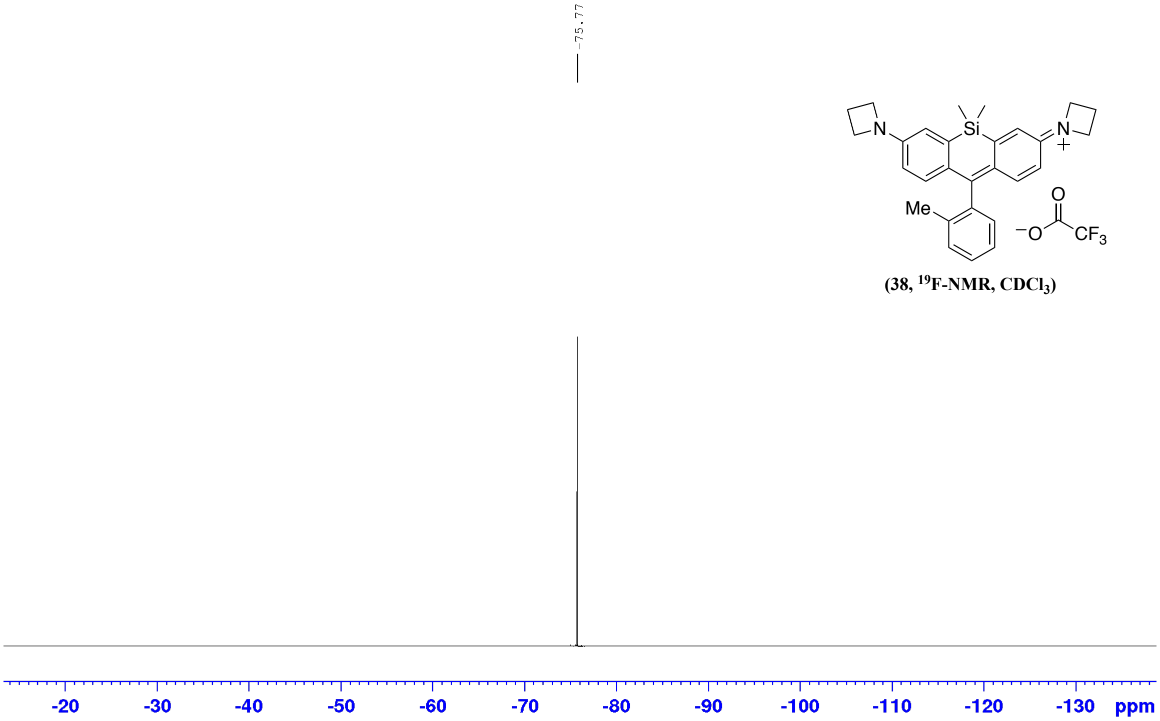

(38, ^{19}F-NMR, $CDCl_3$)

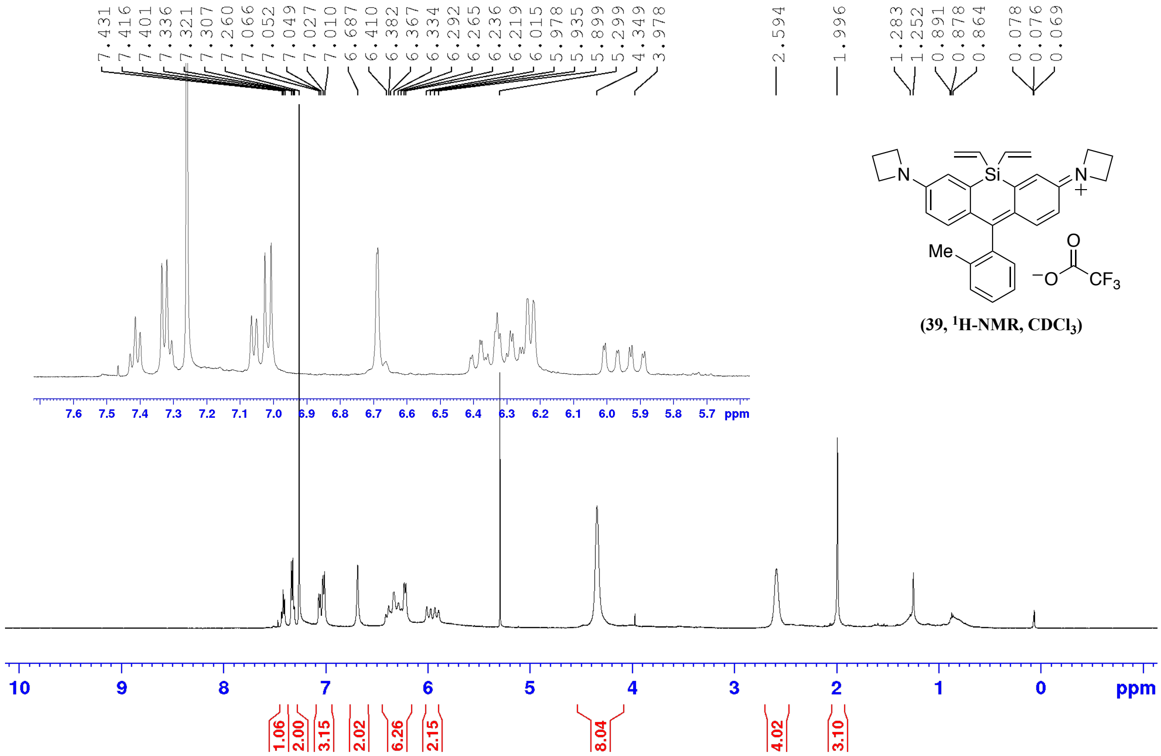

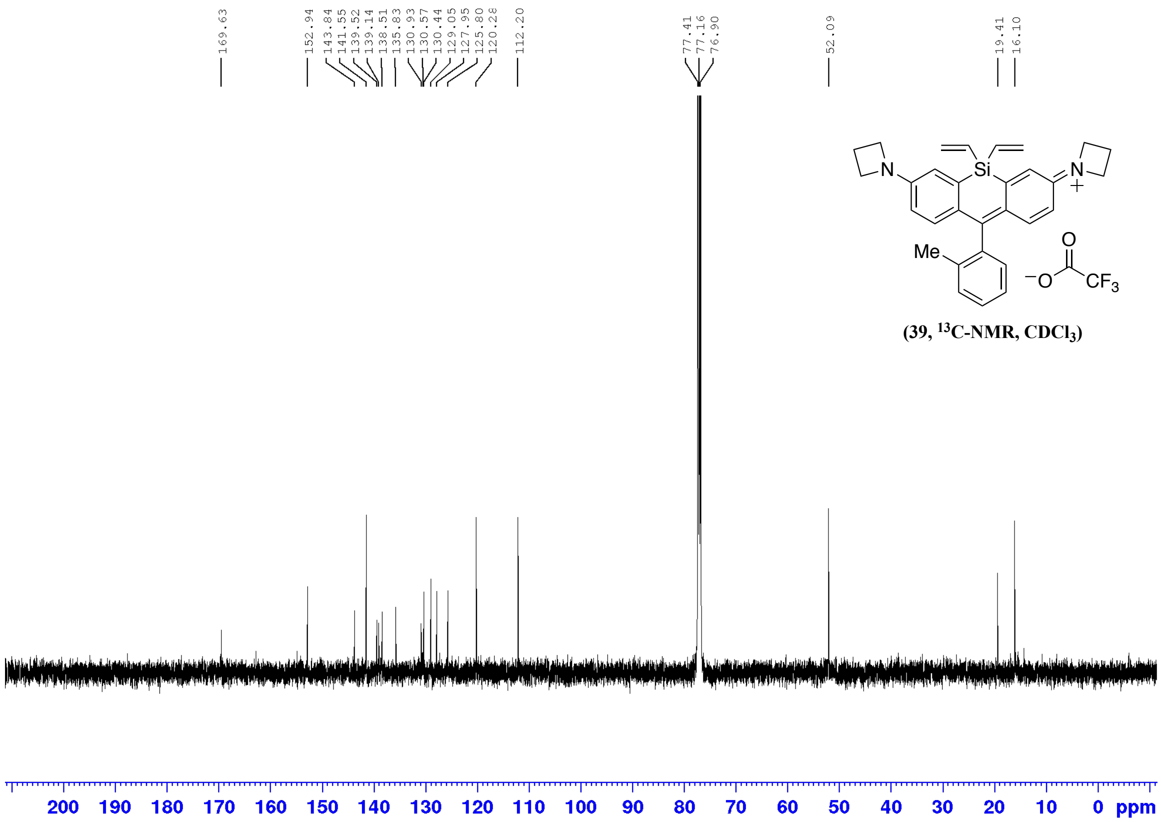

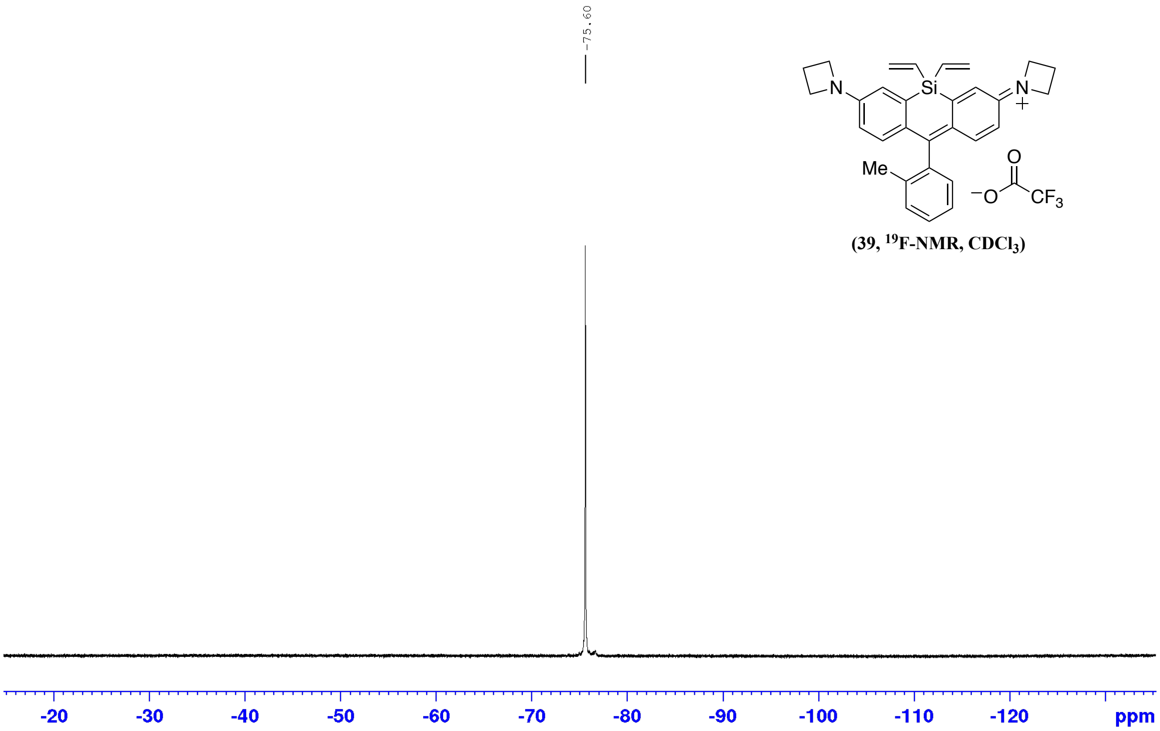

(39, ^{19}F-NMR, $CDCl_3$)

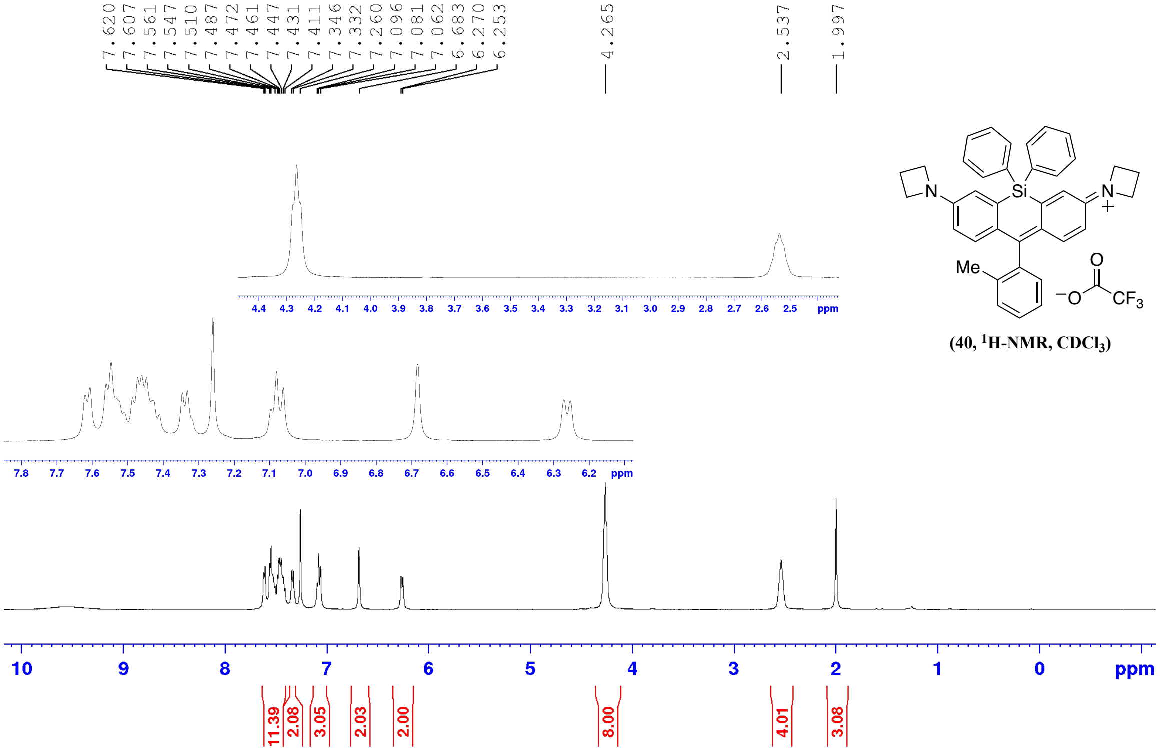

(40, ^{1}H-NMR, $CDCl_3$)

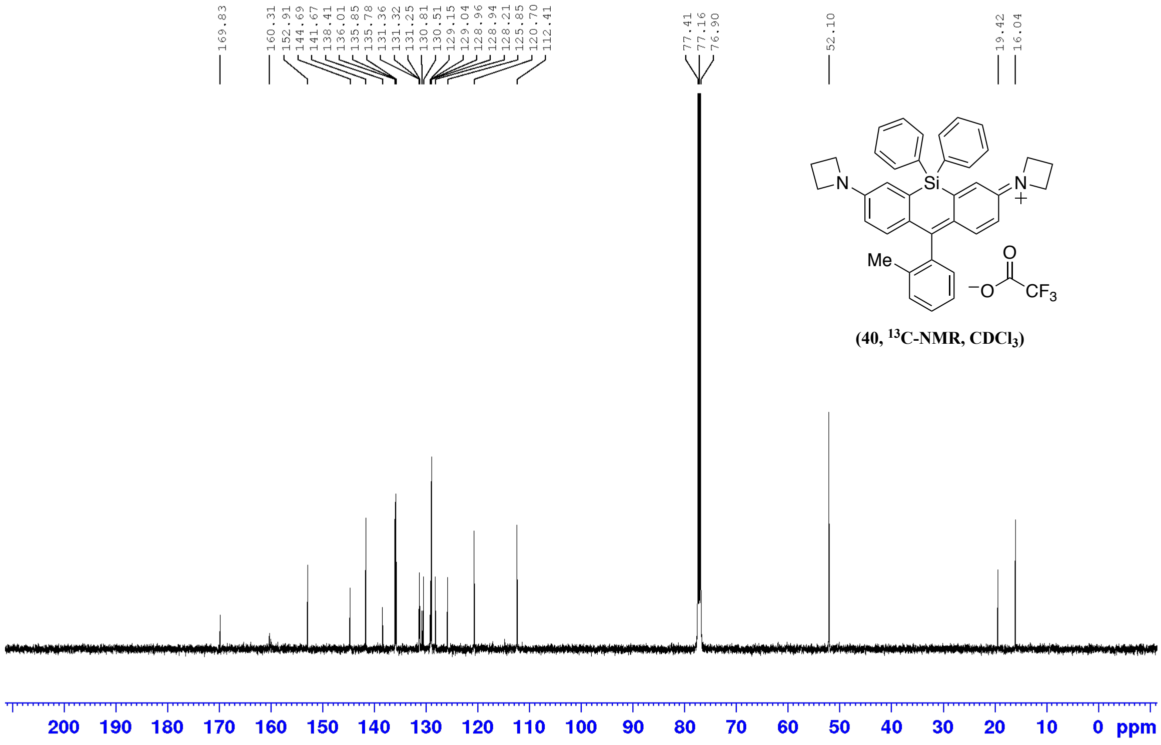

(40, ^{13}C-NMR, $CDCl_3$)

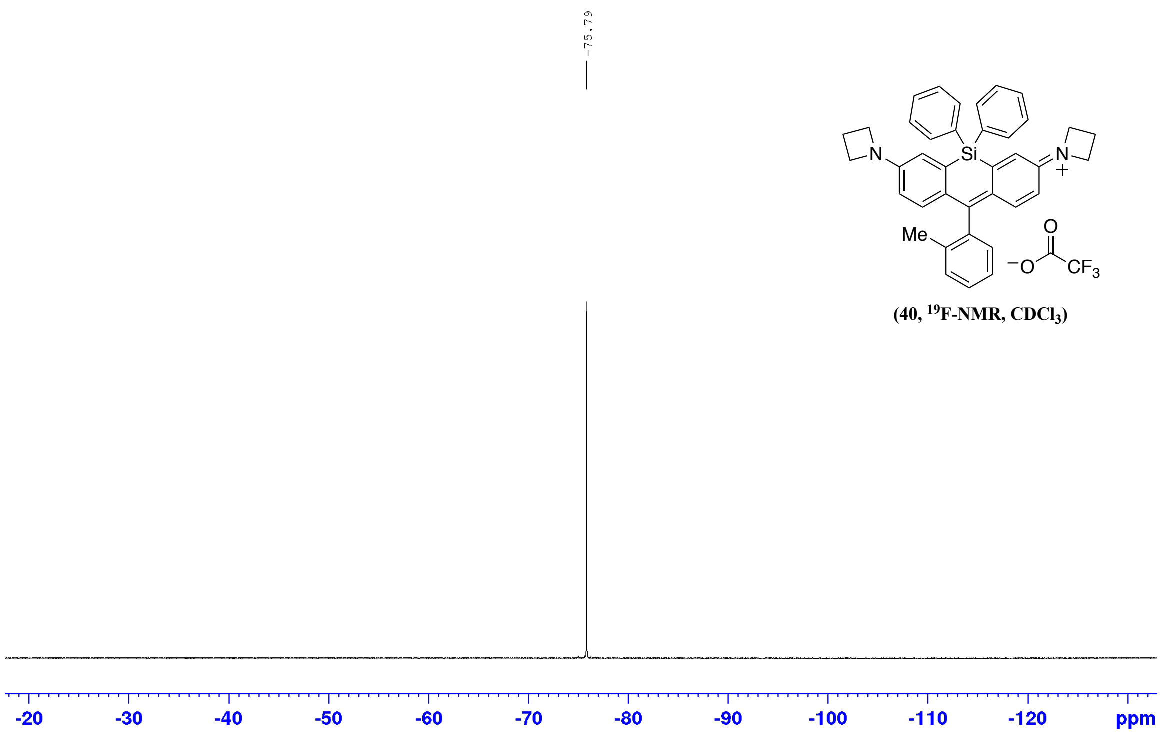

(40, ^{19}F-NMR, $CDCl_3$)

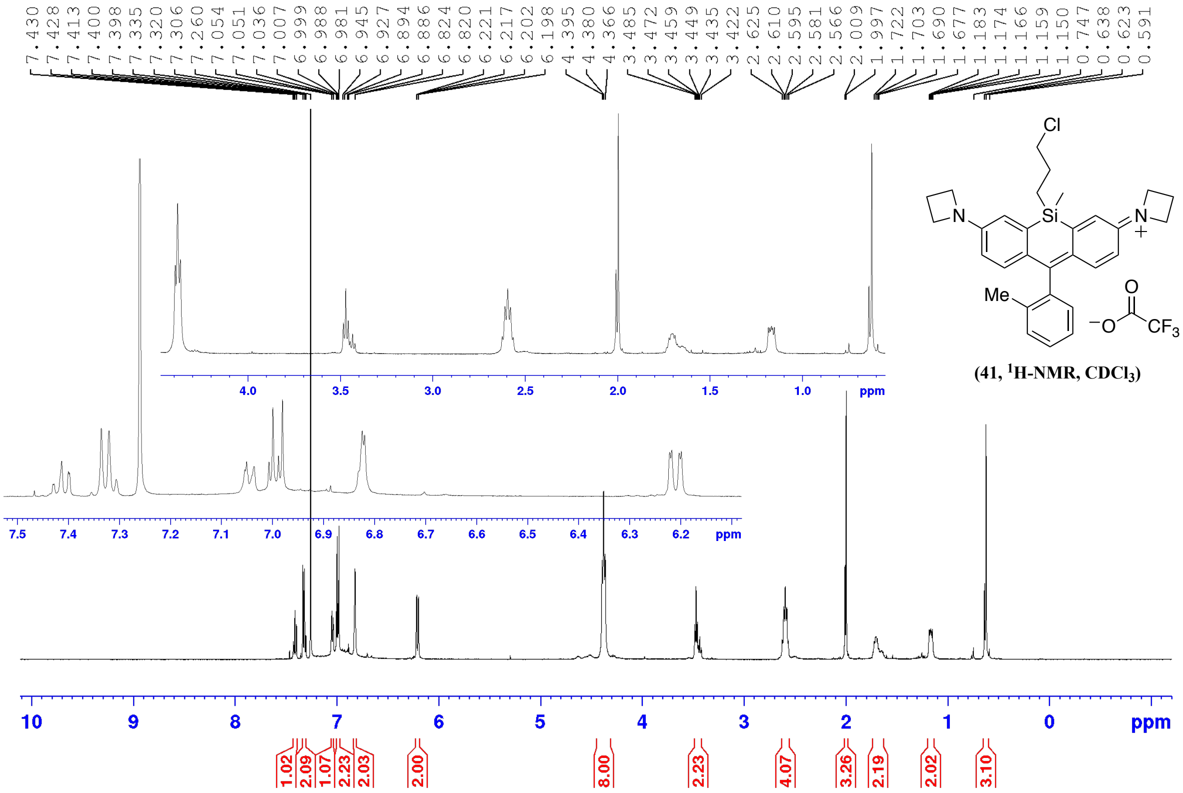

(41, ^{1}H-NMR, $CDCl_3$)

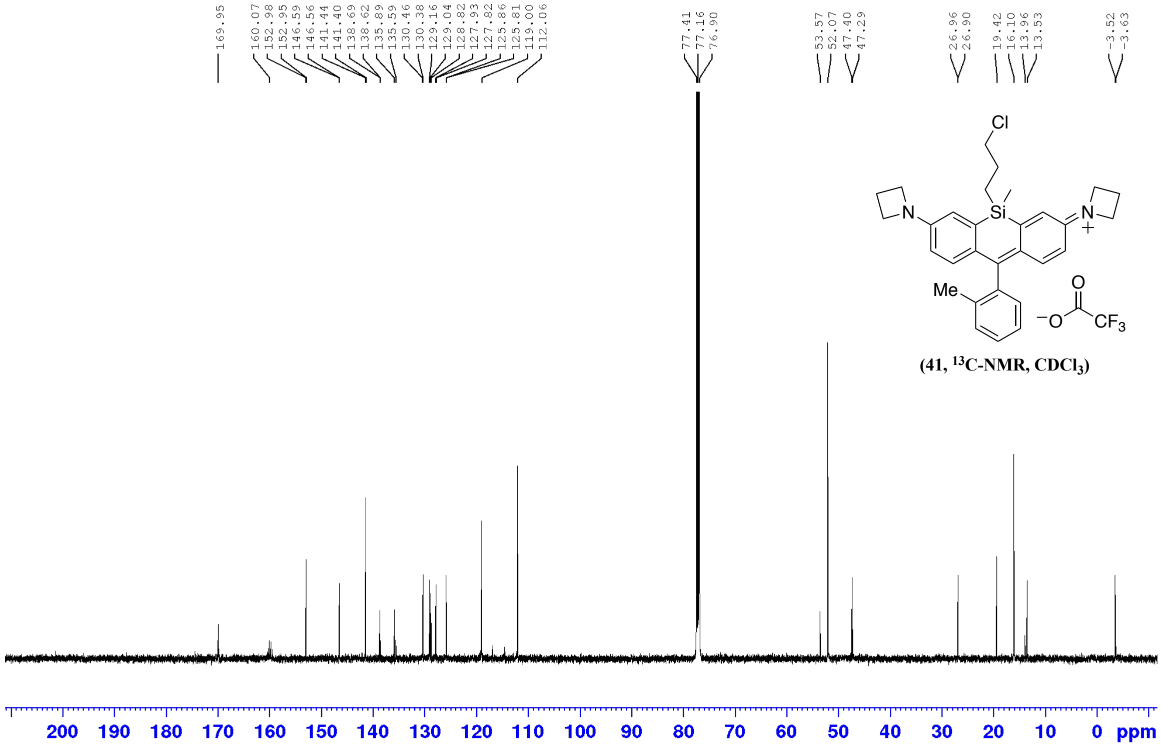

(41, ^{13}C-NMR, $CDCl_3$)

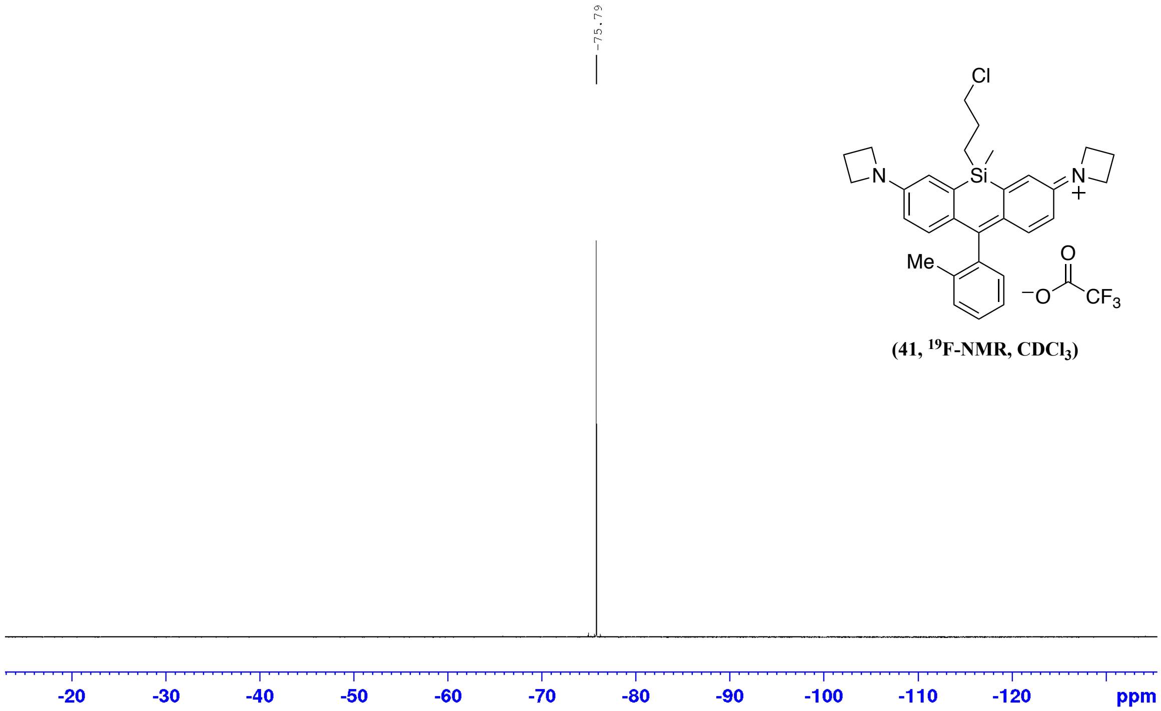

(41, ^{19}F-NMR, $CDCl_3$)

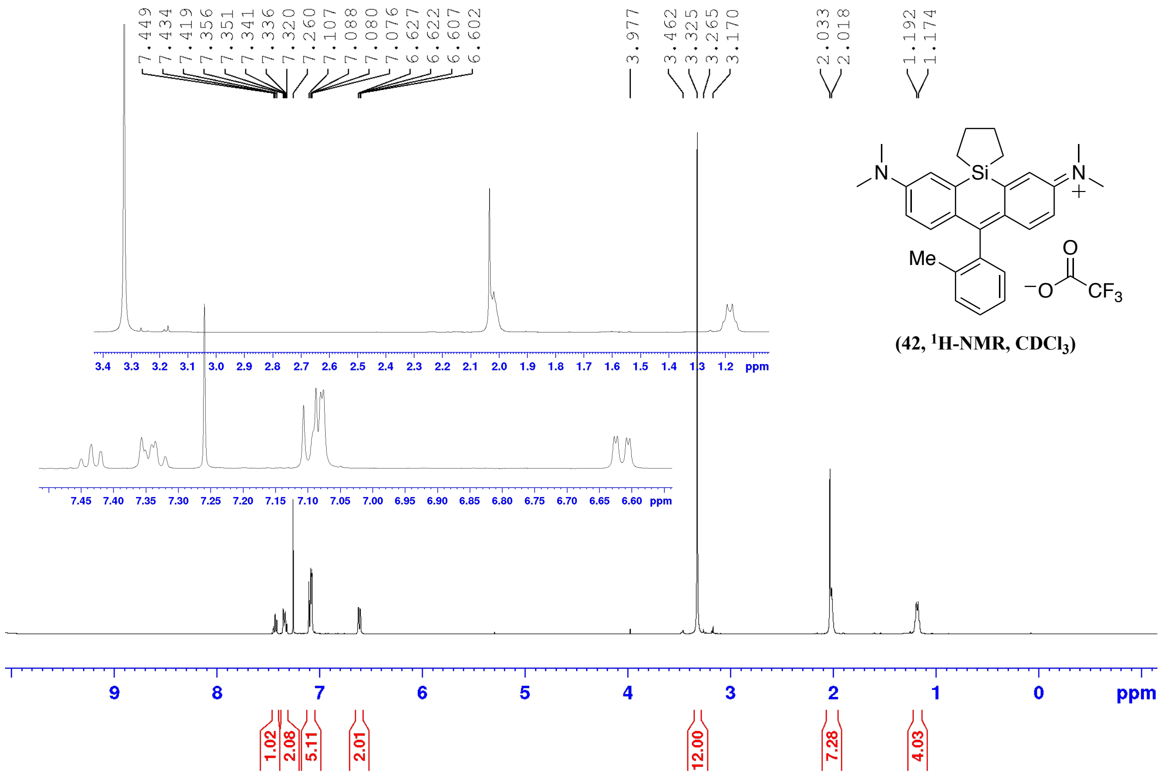

(42, ^{1}H-NMR, $CDCl_3$)

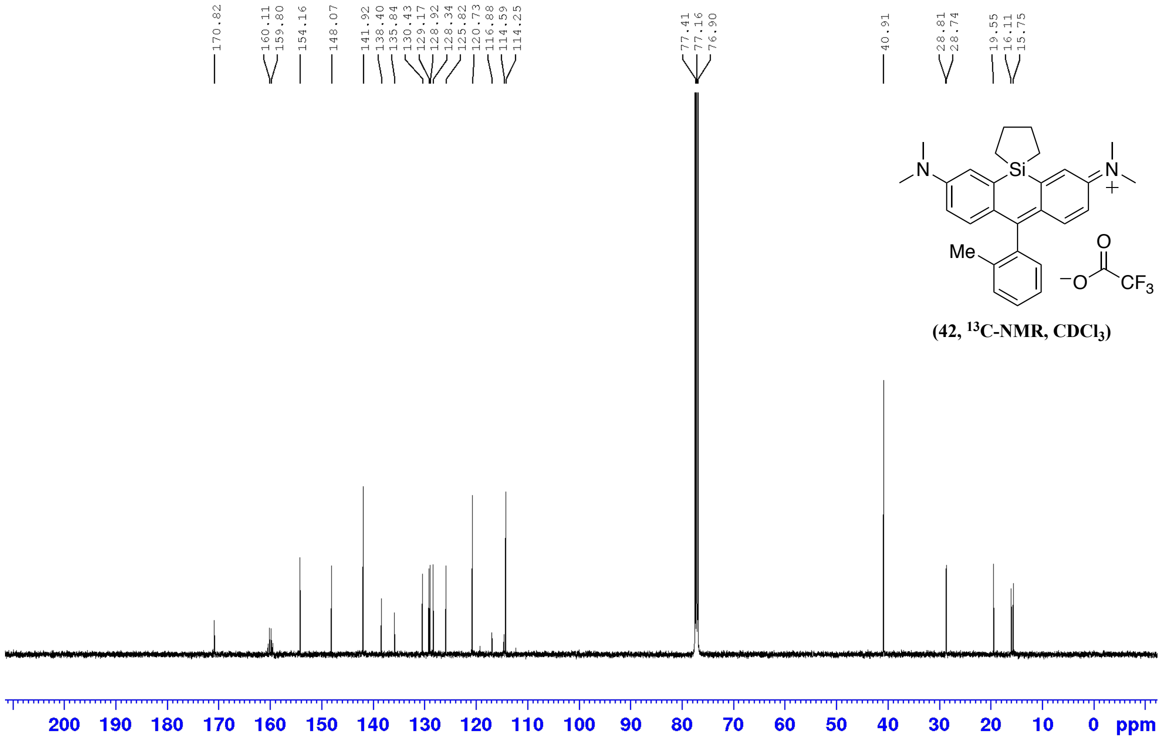

(42, ^{13}C-NMR, $CDCl_3$)

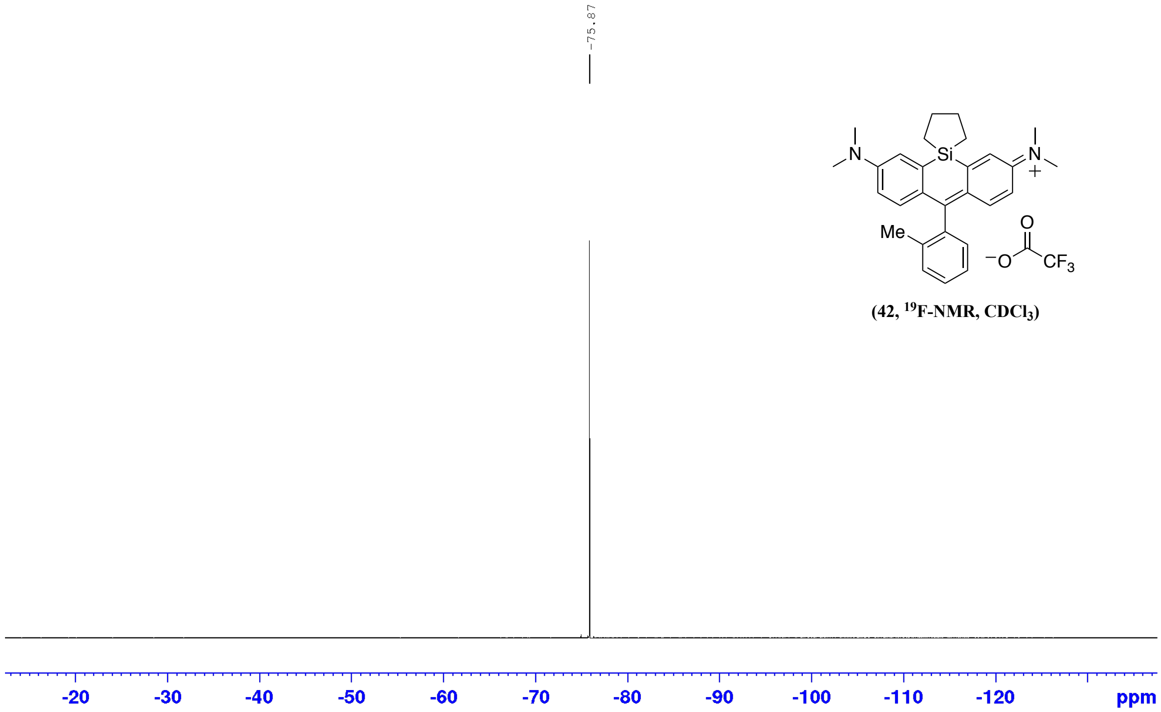

(42, ^{19}F-NMR, $CDCl_3$)

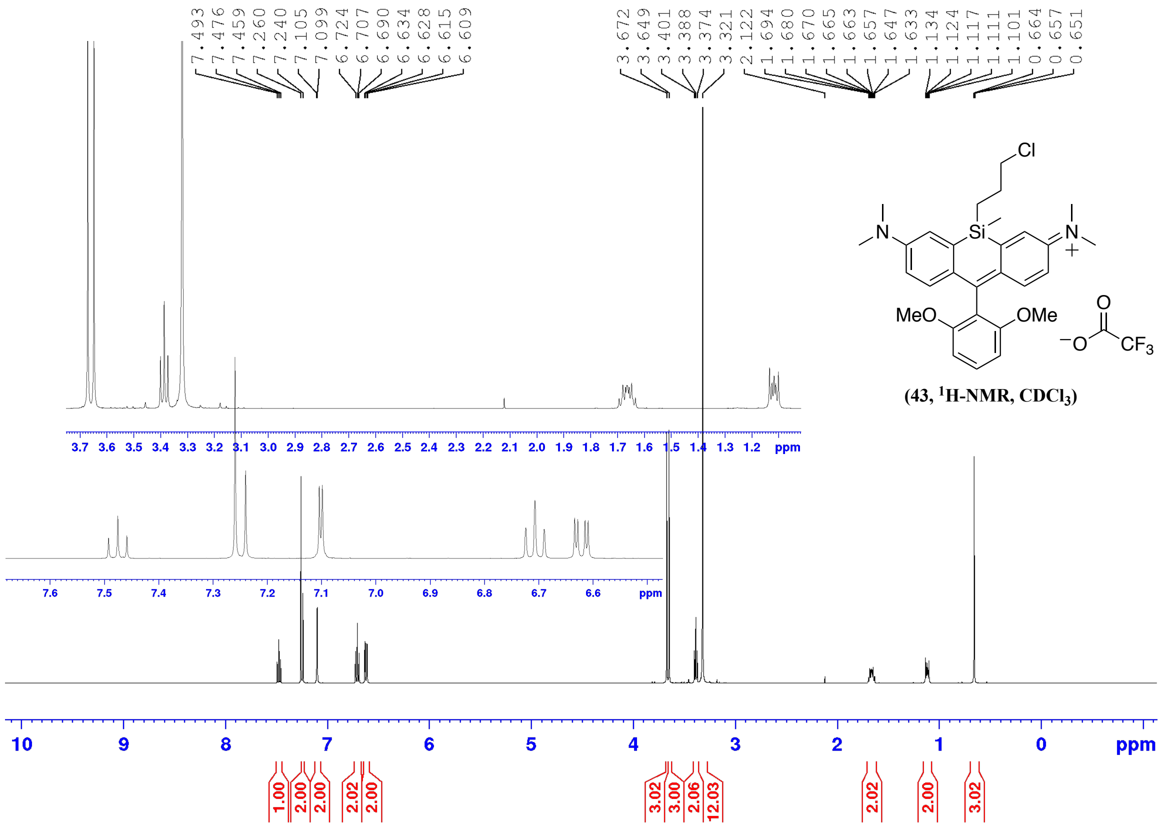

(43, ^{1}H-NMR, $CDCl_3$)

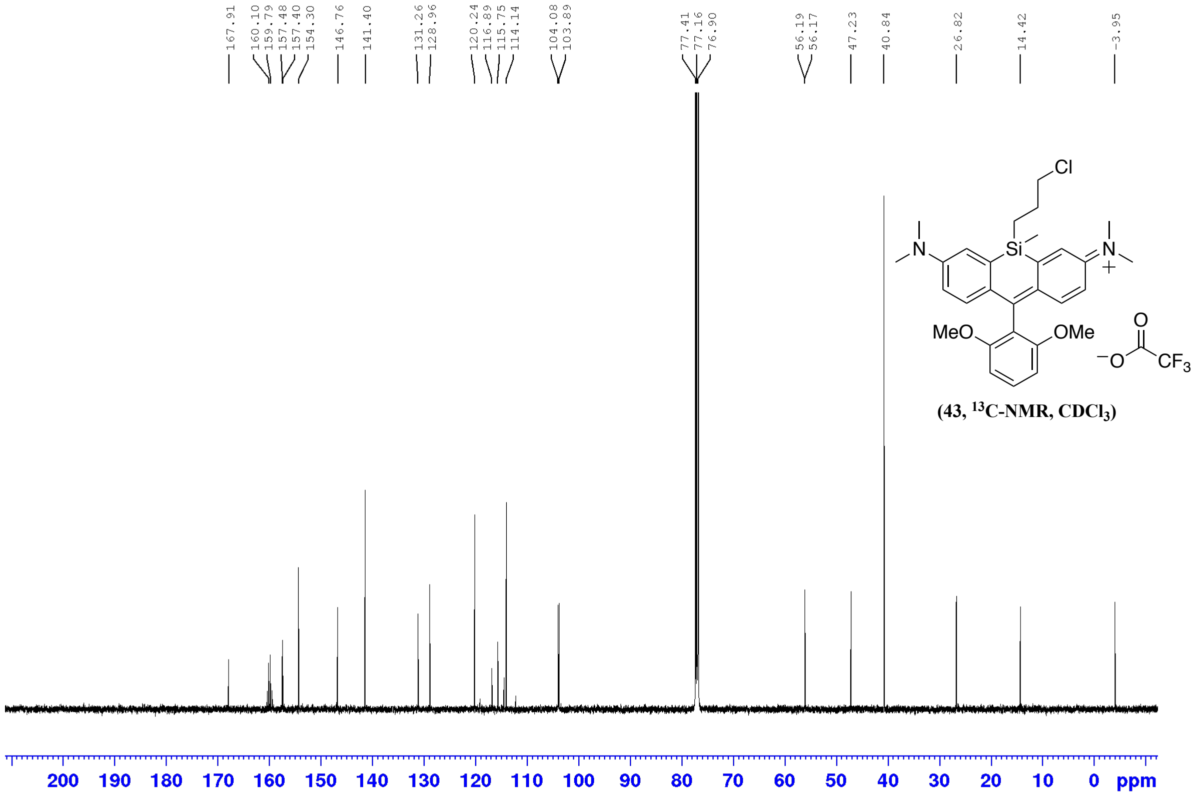

(43, ^{13}C-NMR, $CDCl_3$)

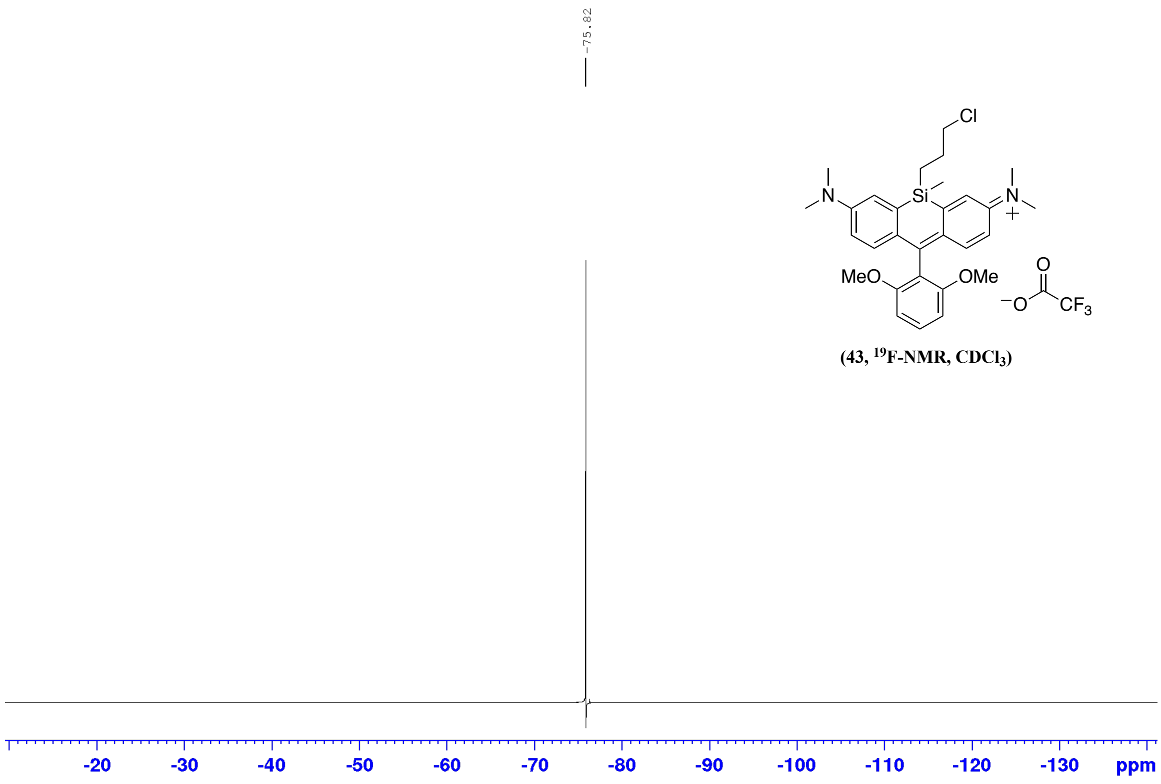

(43, ^{19}F-NMR, $CDCl_3$)

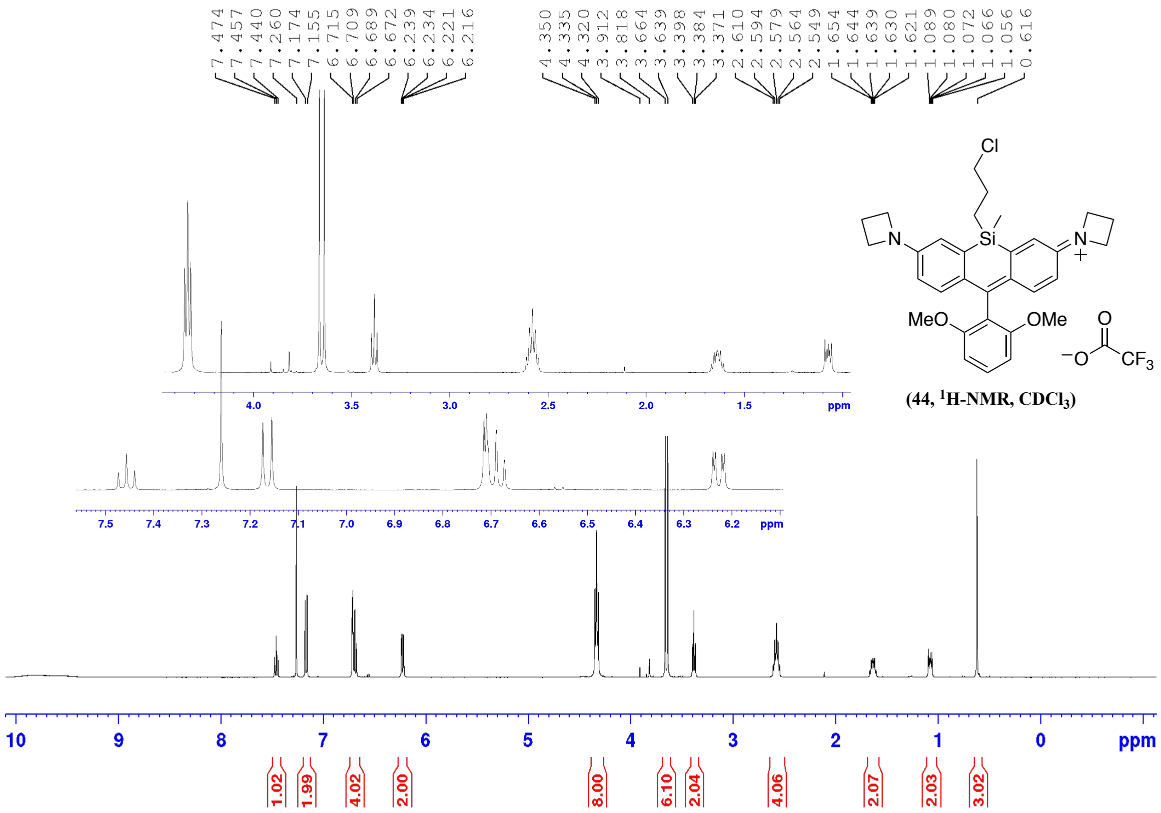

(44, ^{1}H-NMR, $CDCl_3$)

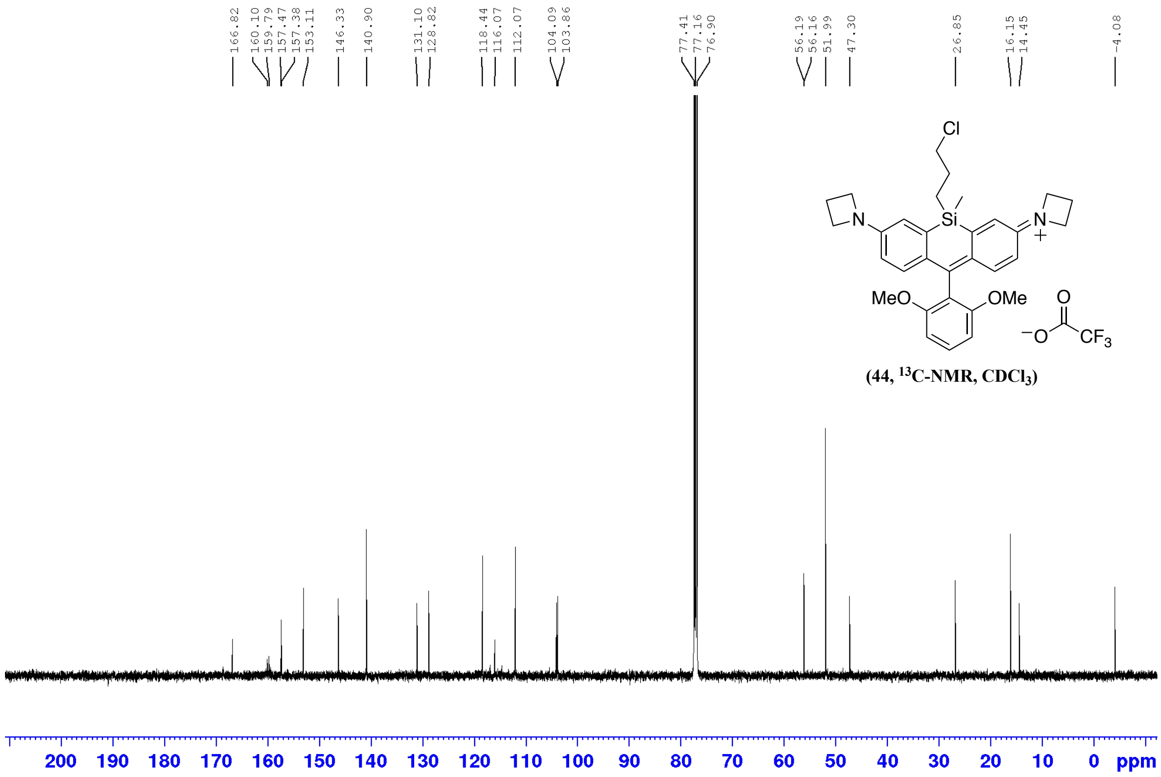

(44, ^{13}C-NMR, $CDCl_3$)

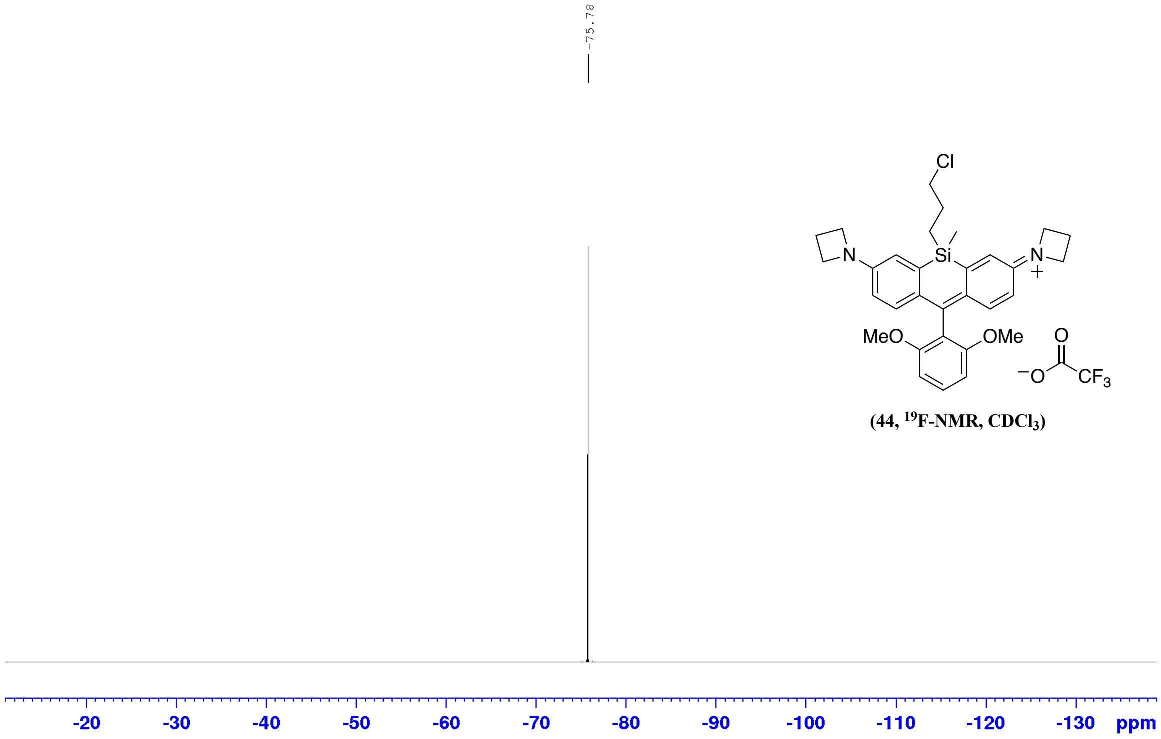

(44, ^{19}F-NMR, $CDCl_3$)

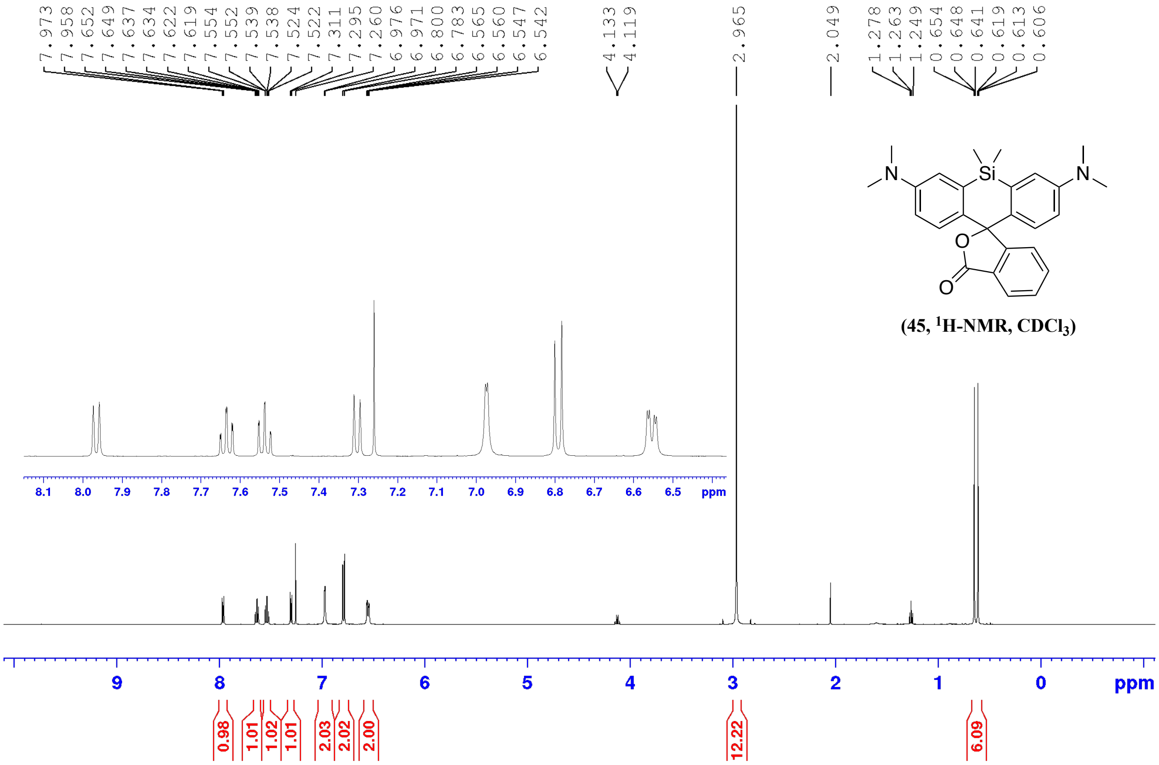
(45, ^{1}H-NMR, $CDCl_3$)

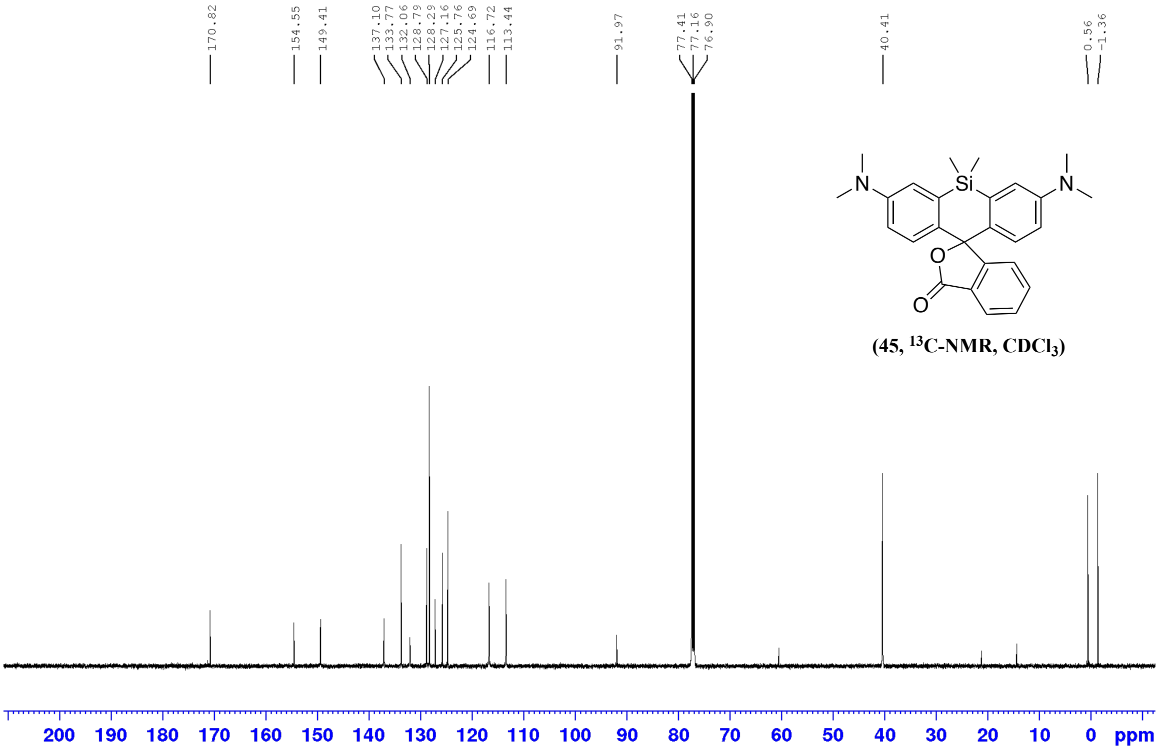
(45, ^{13}C-NMR, $CDCl_3$)

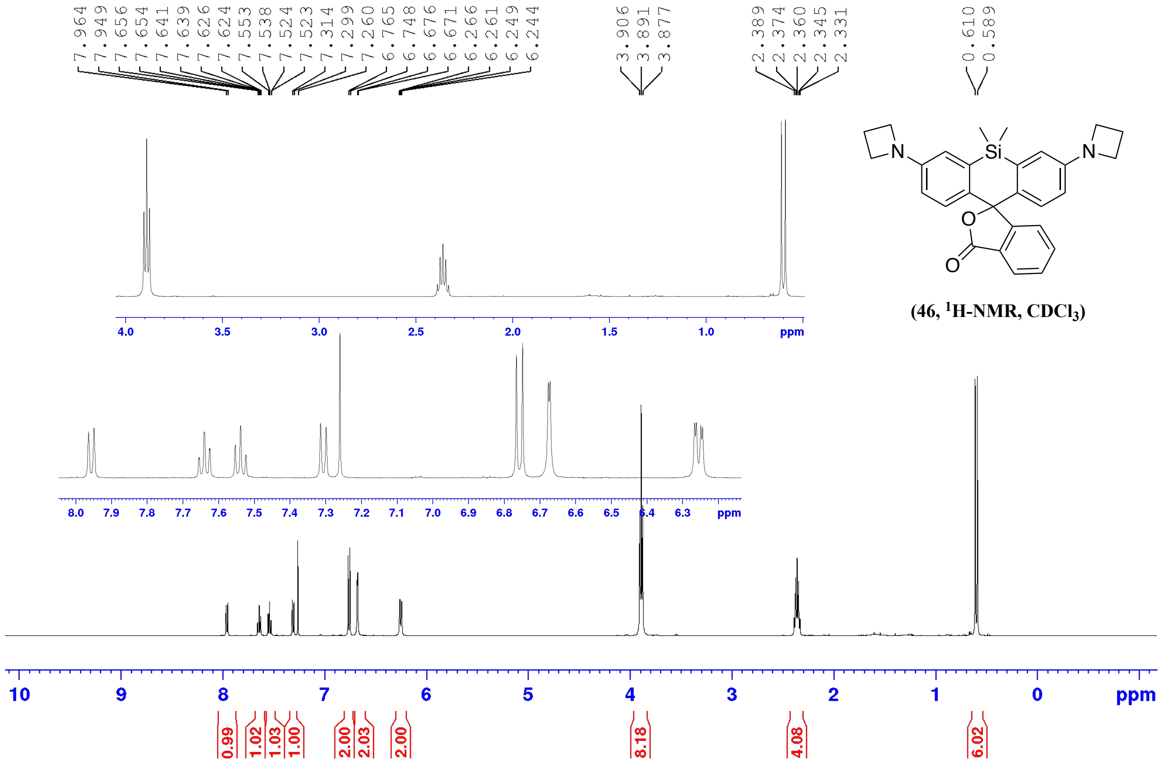

(46, ^{1}H-NMR, $CDCl_3$)

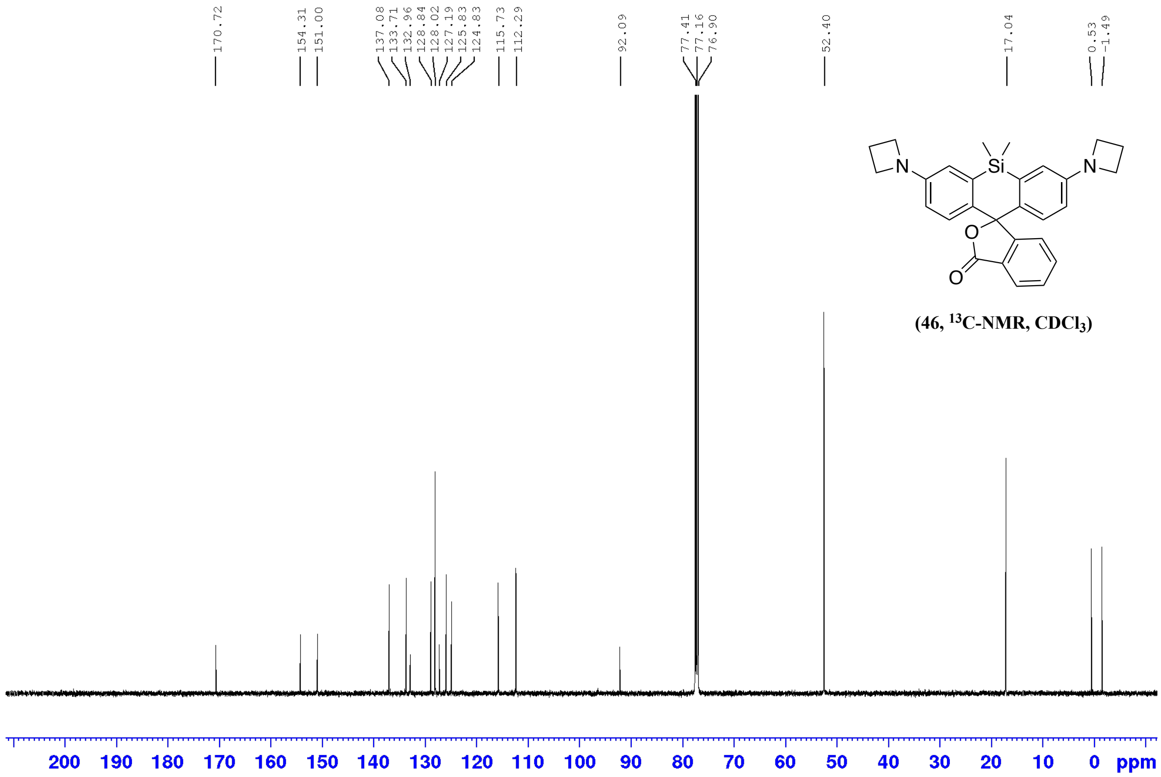

(46, ^{13}C-NMR, $CDCl_3$)

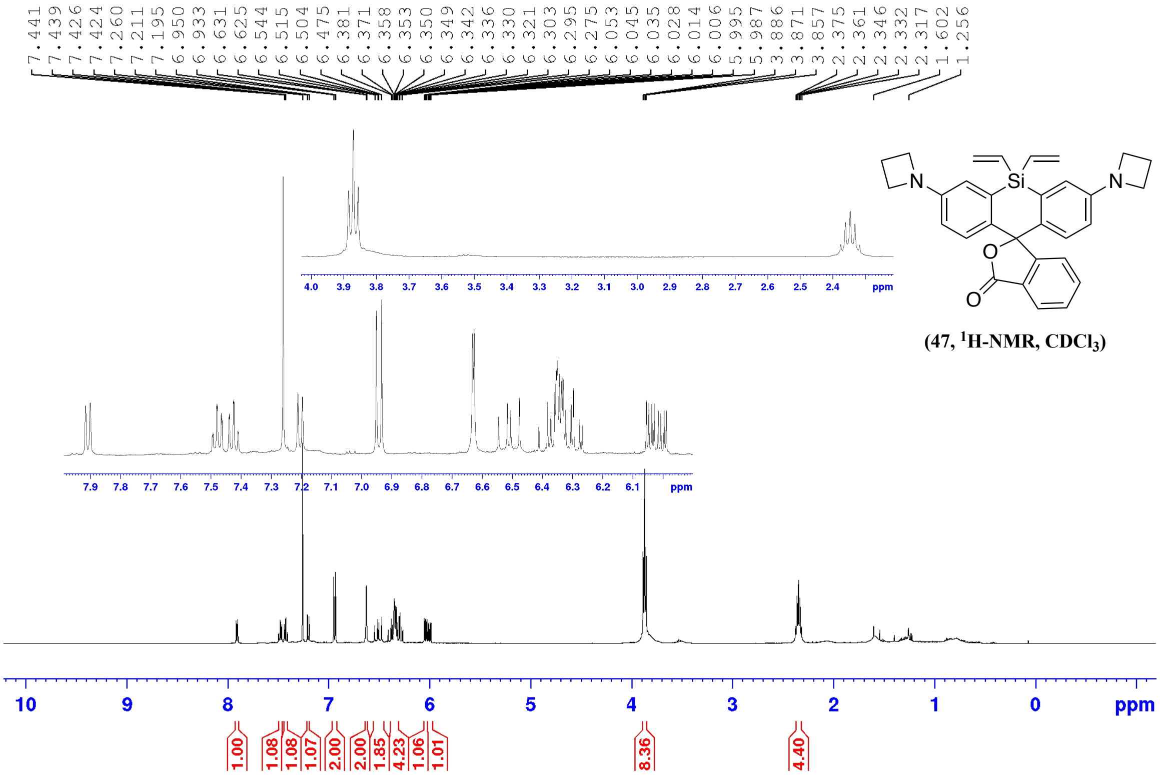

(47, ^{1}H-NMR, $CDCl_3$)

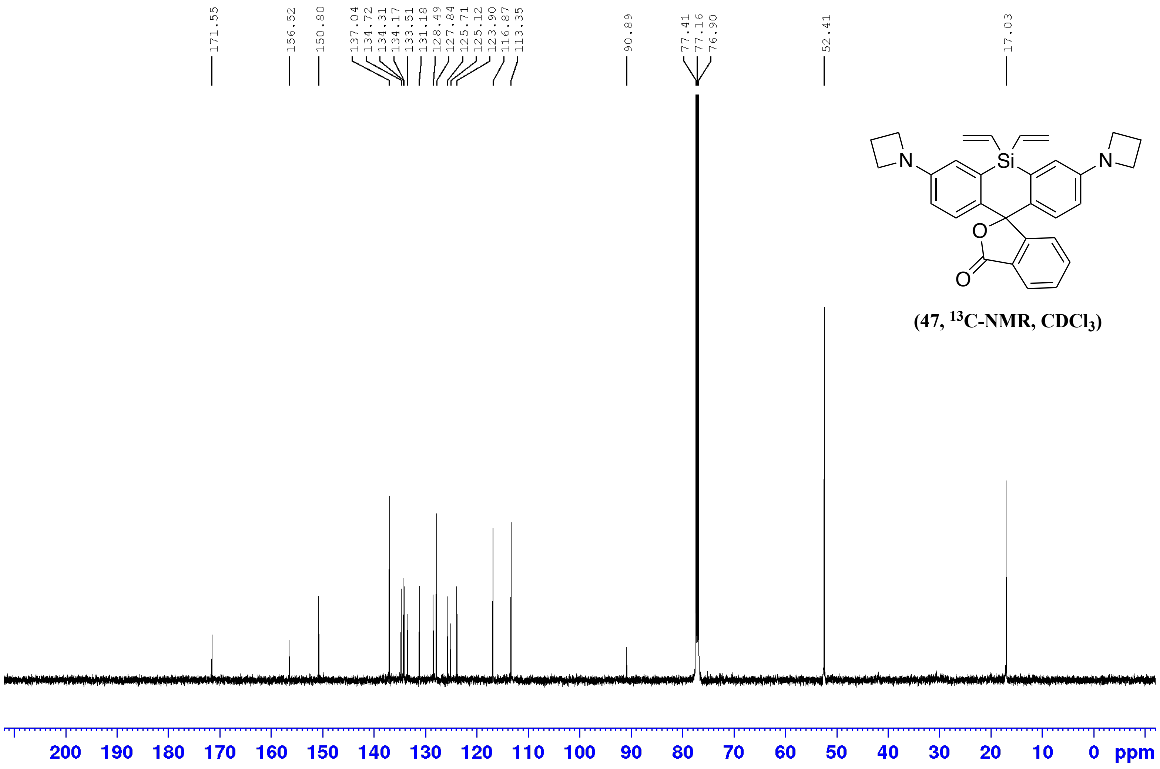

(47, ^{13}C-NMR, $CDCl_3$)

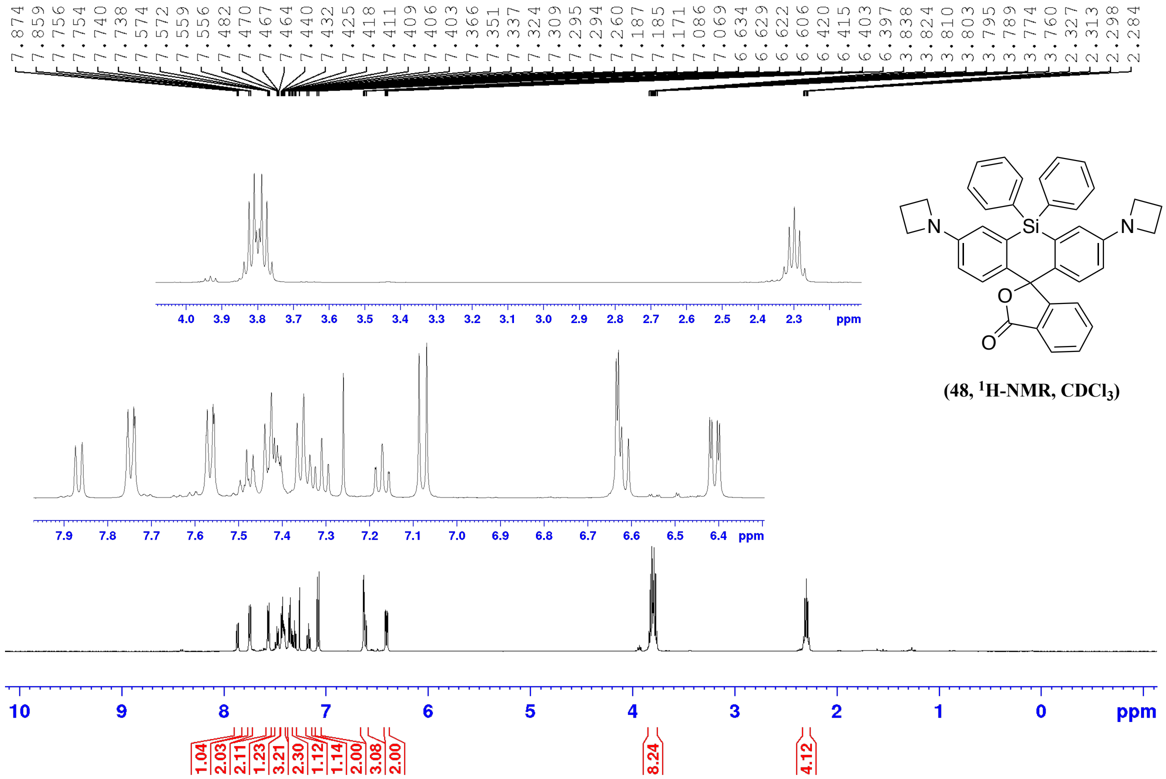

(48, ^{1}H-NMR, $CDCl_3$)

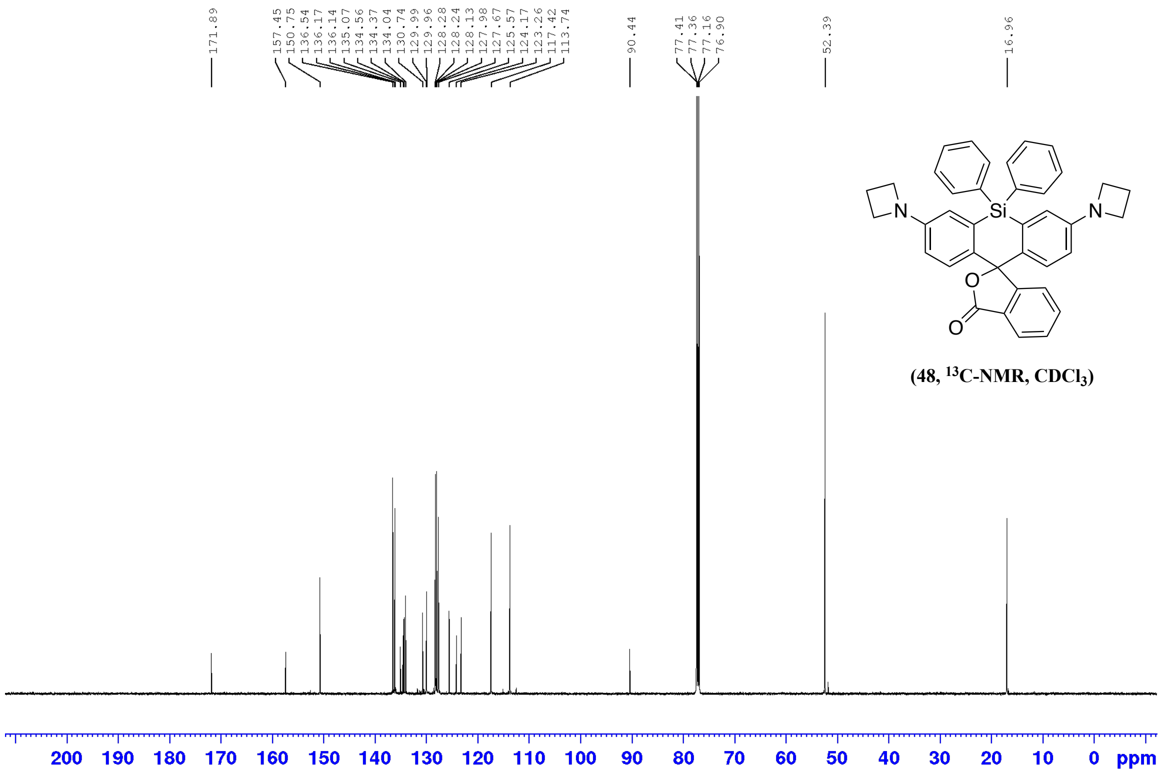

(48, ^{13}C-NMR, $CDCl_3$)

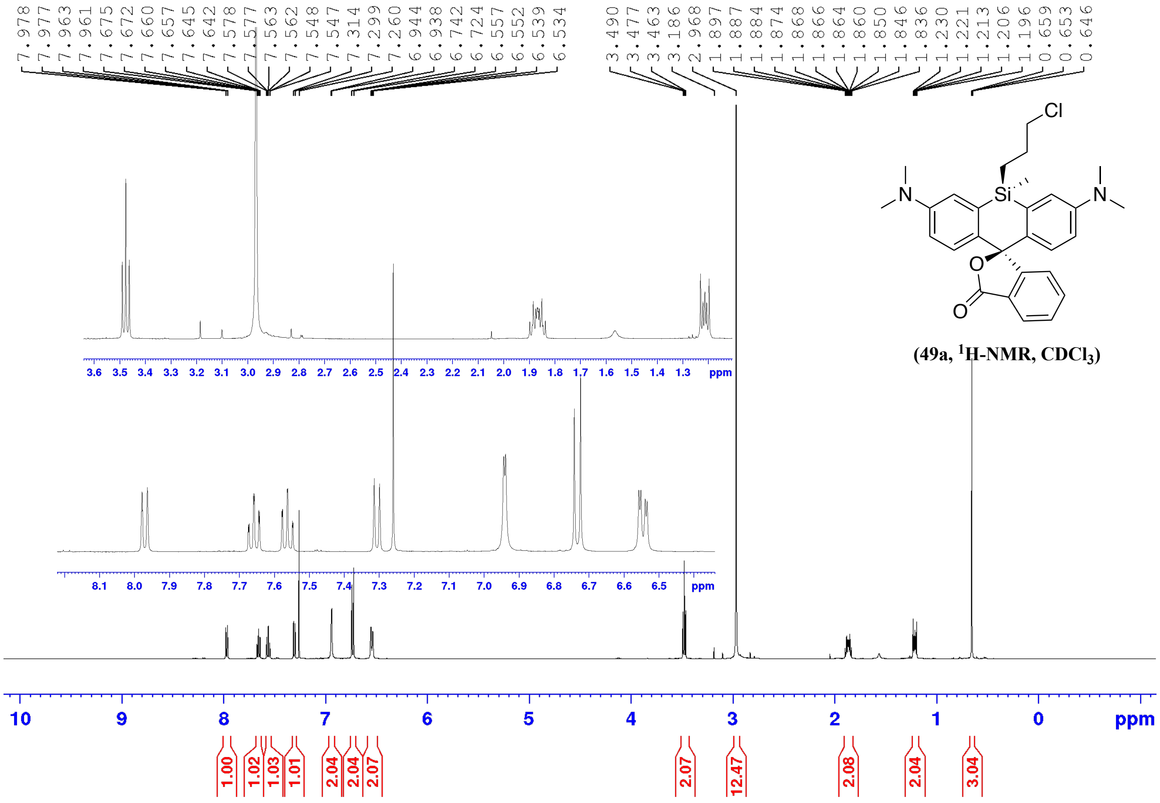
(49a, ¹H-NMR, CDCl₃)

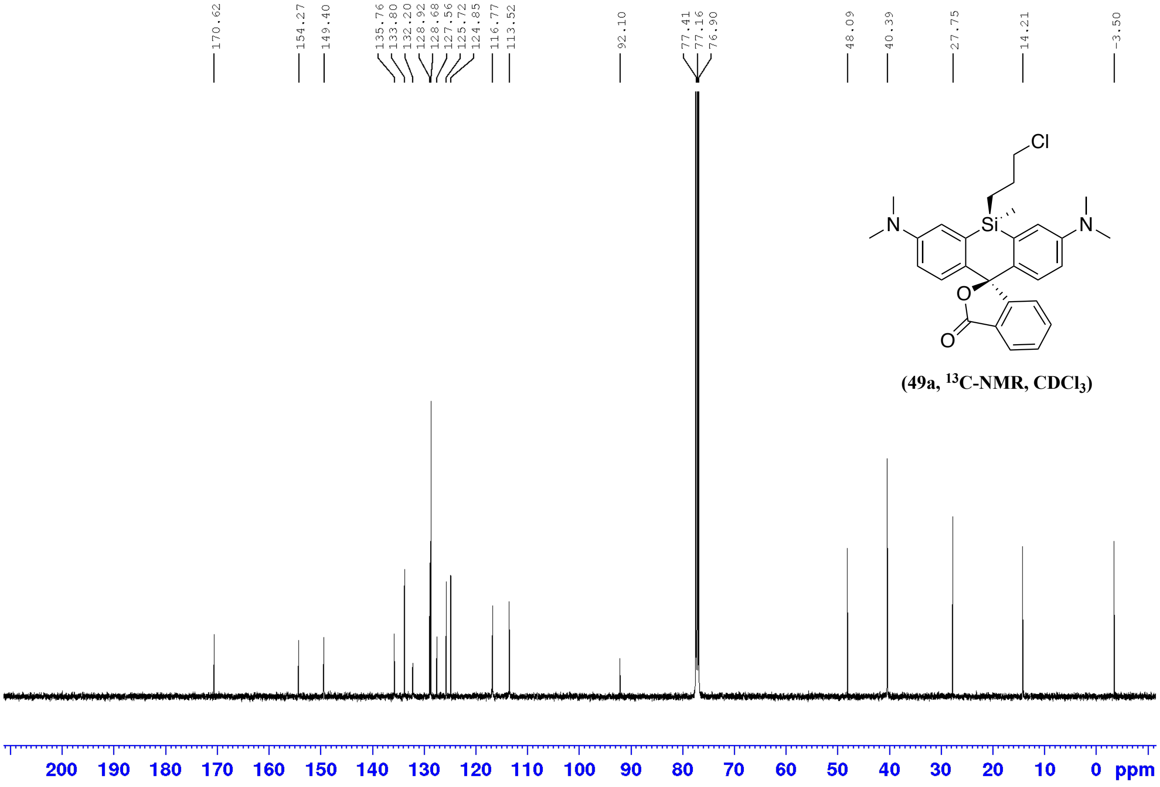
(49a, ¹³C-NMR, CDCl₃)

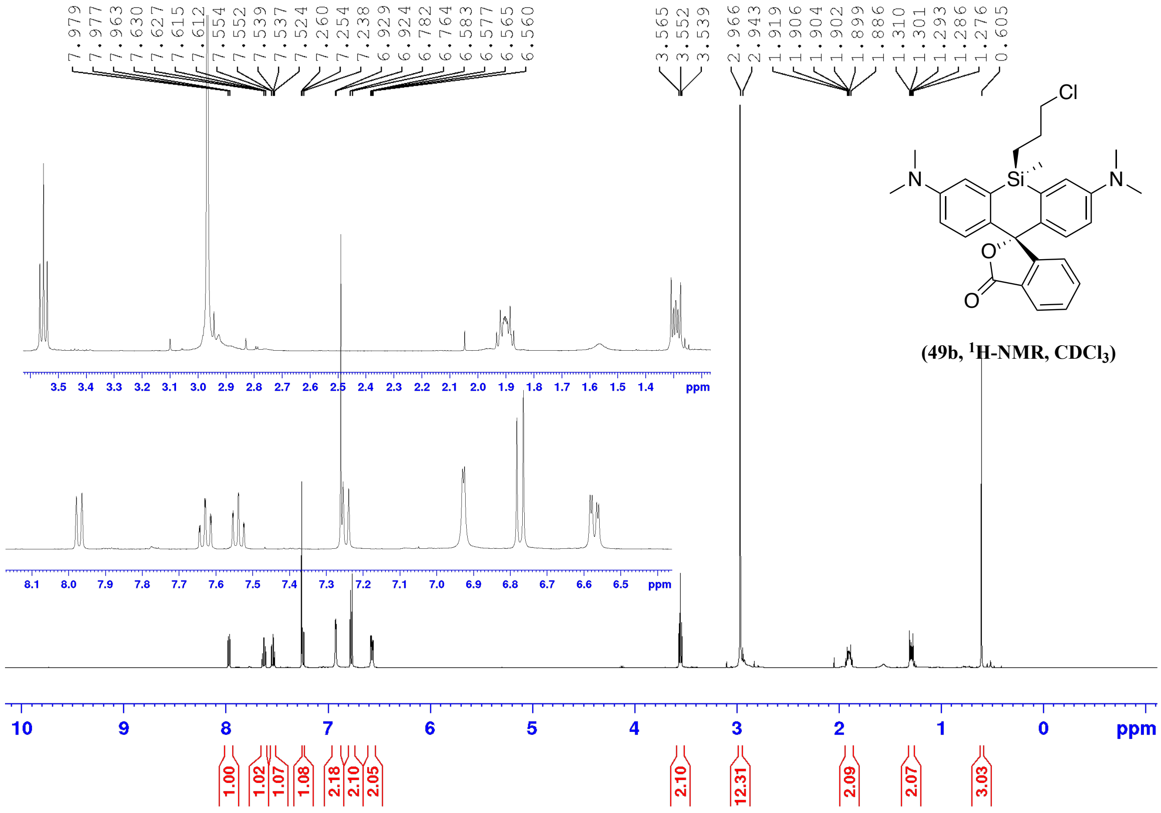

(49b, ^{1}H-NMR, $CDCl_3$)

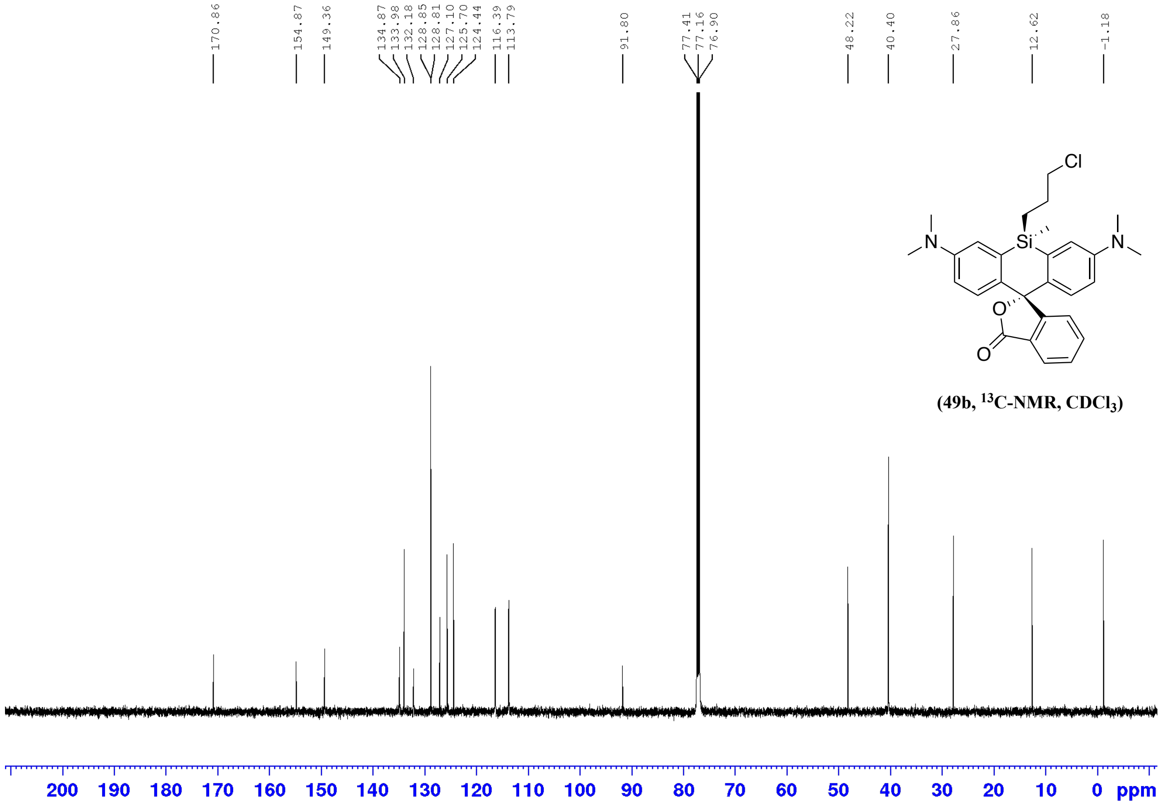

(49b, ^{13}C-NMR, $CDCl_3$)

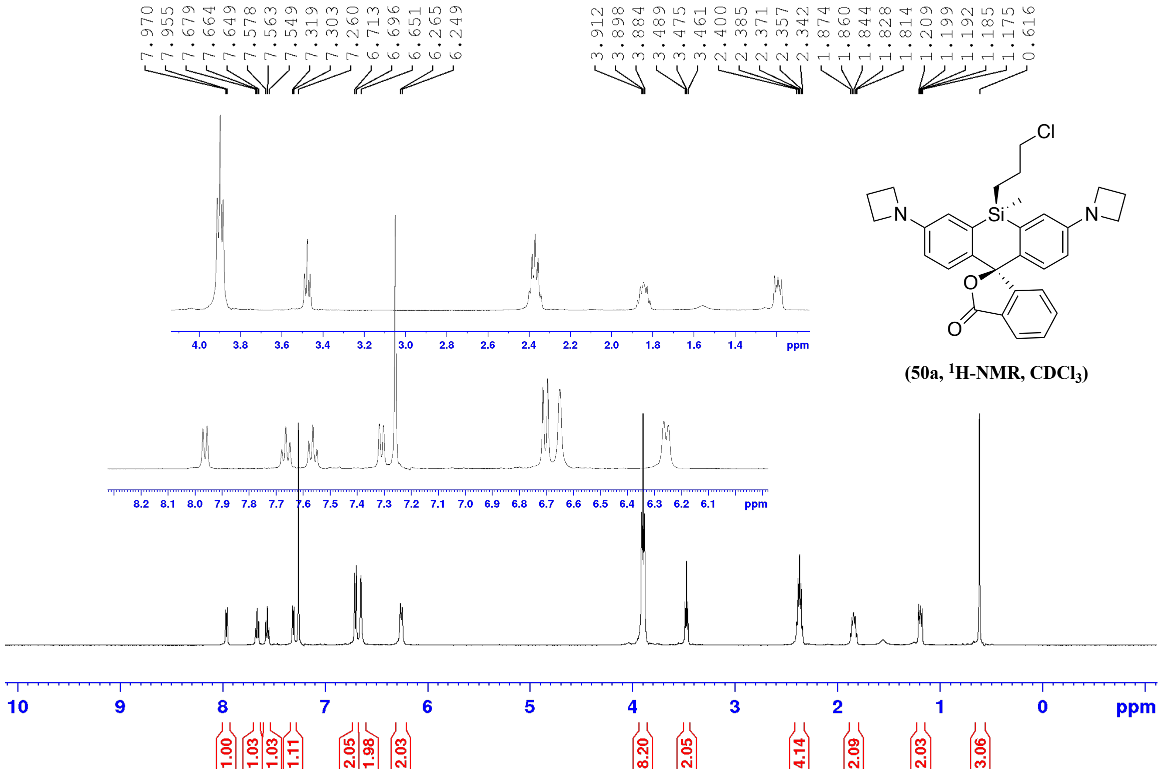
(50a, ¹H-NMR, CDCl3)

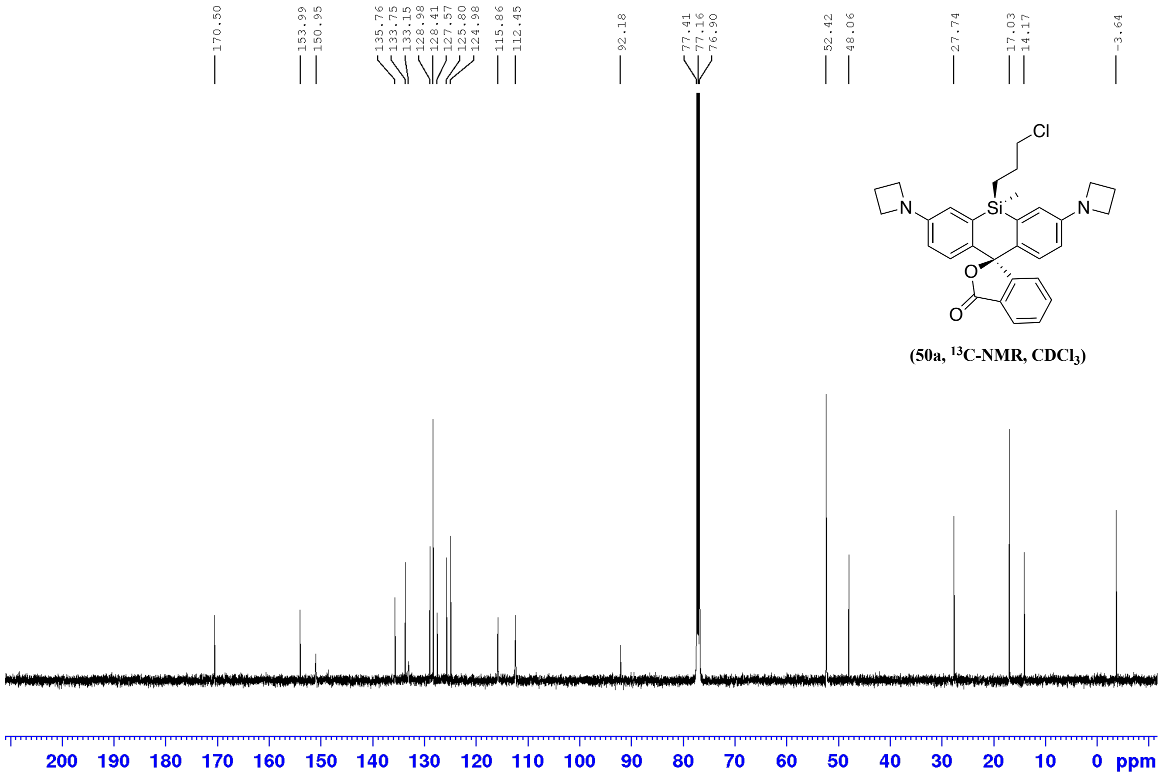
(50a, ¹³C-NMR, CDCl3)

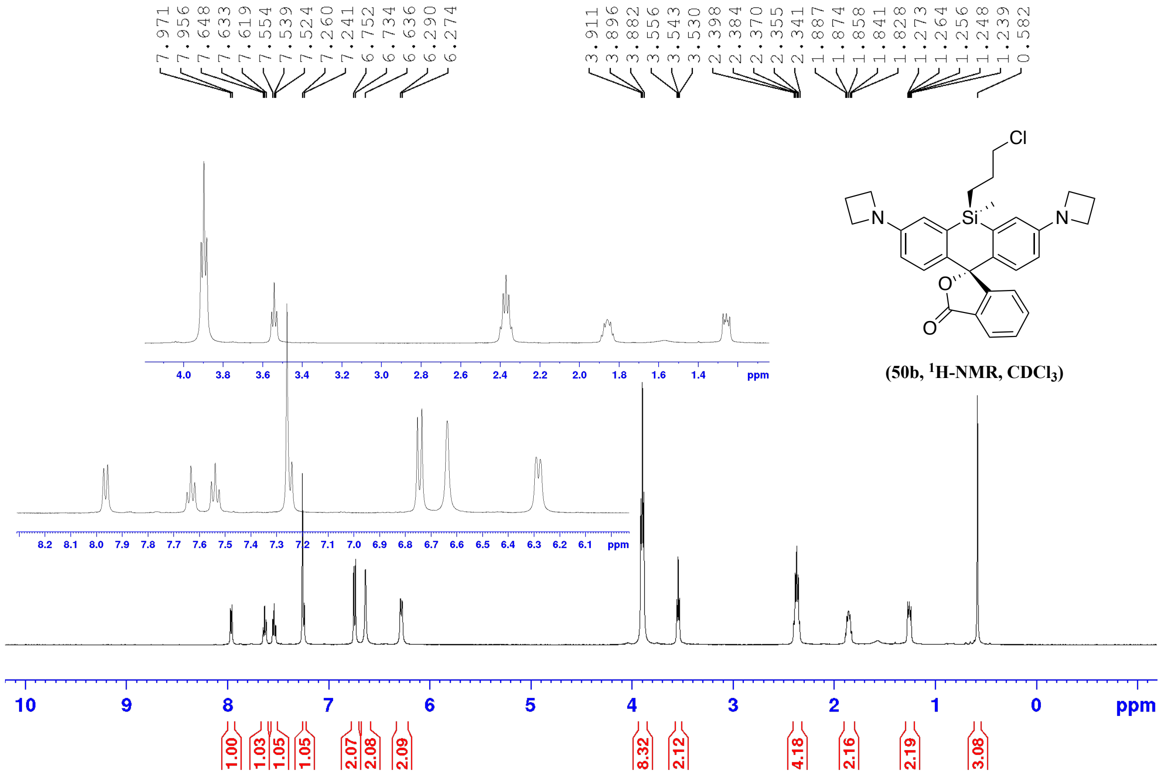

(50b, ^{1}H-NMR, $CDCl_3$)

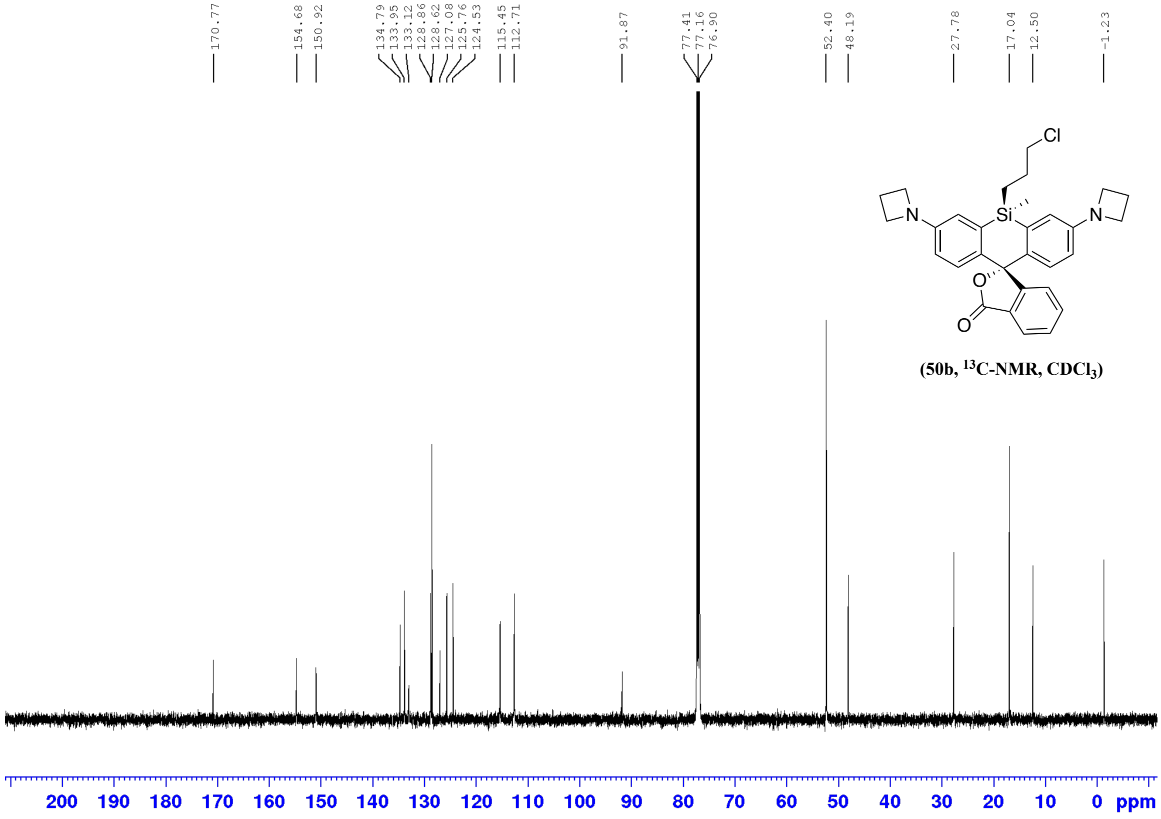

(50b, ^{13}C-NMR, $CDCl_3$)

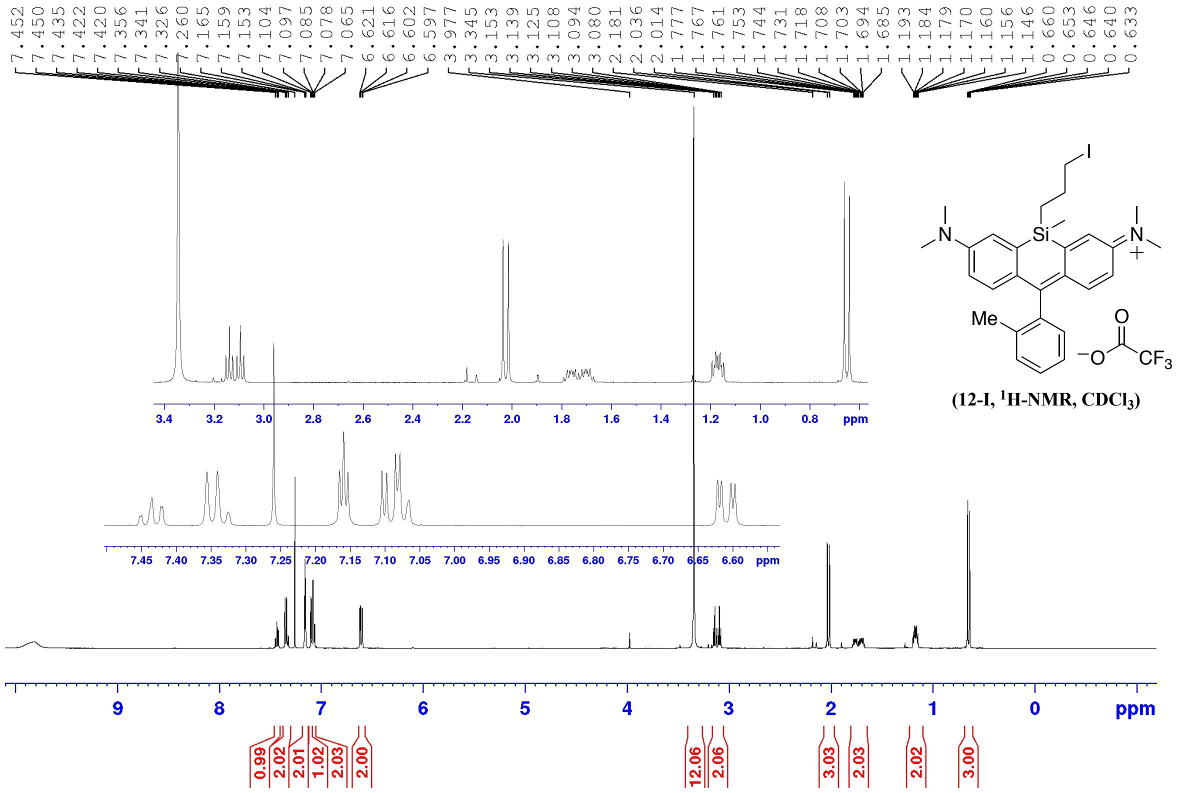

(12-I, ^{1}H-NMR, $CDCl_3$)

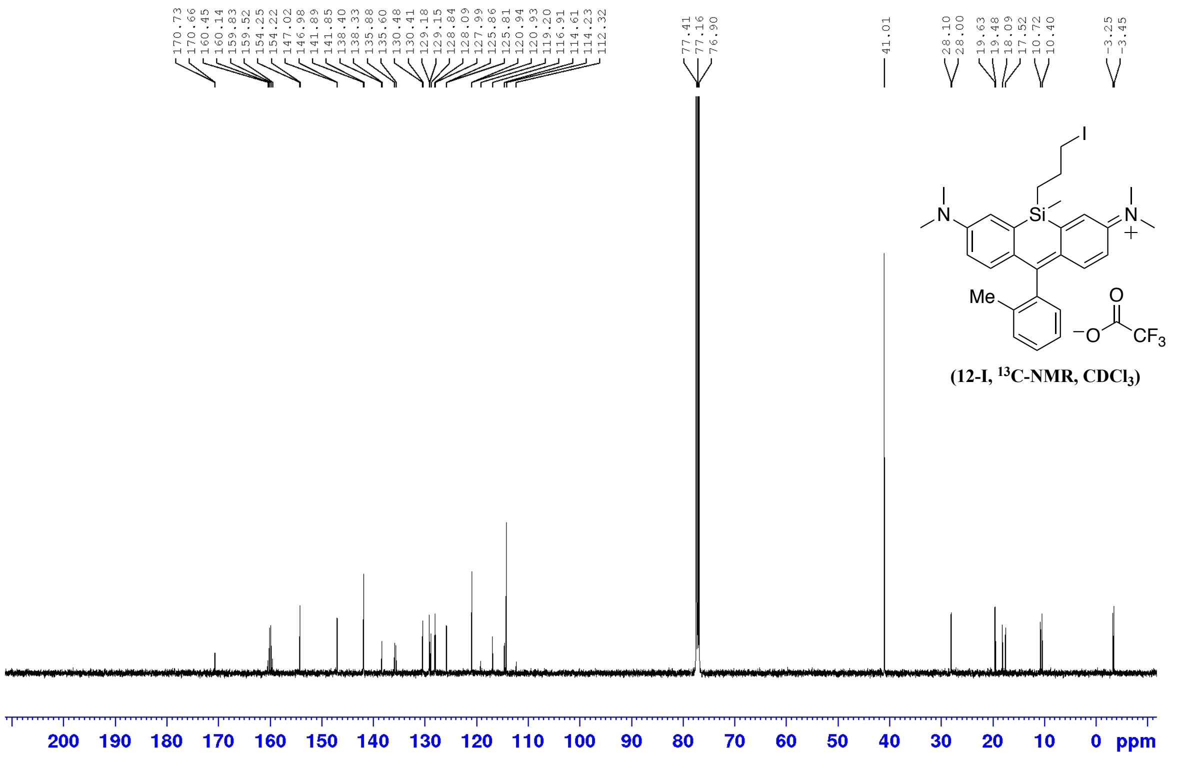

(12-I, ^{13}C-NMR, $CDCl_3$)

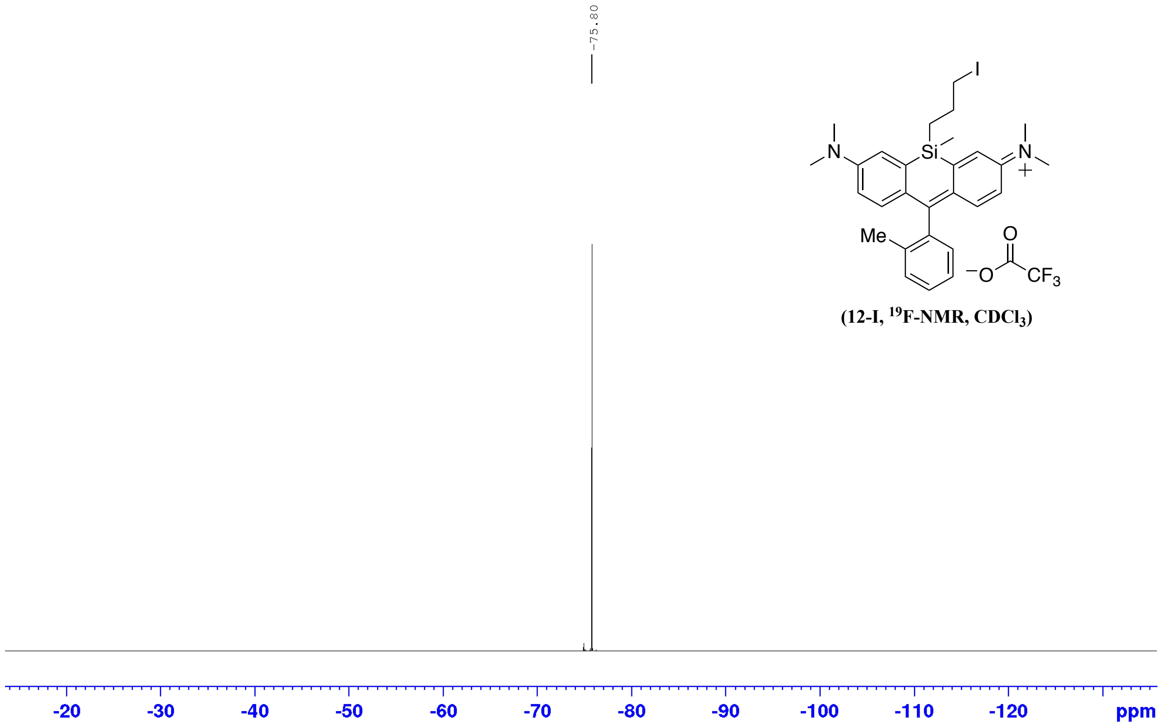

(12-I, ^{19}F-NMR, $CDCl_3$)

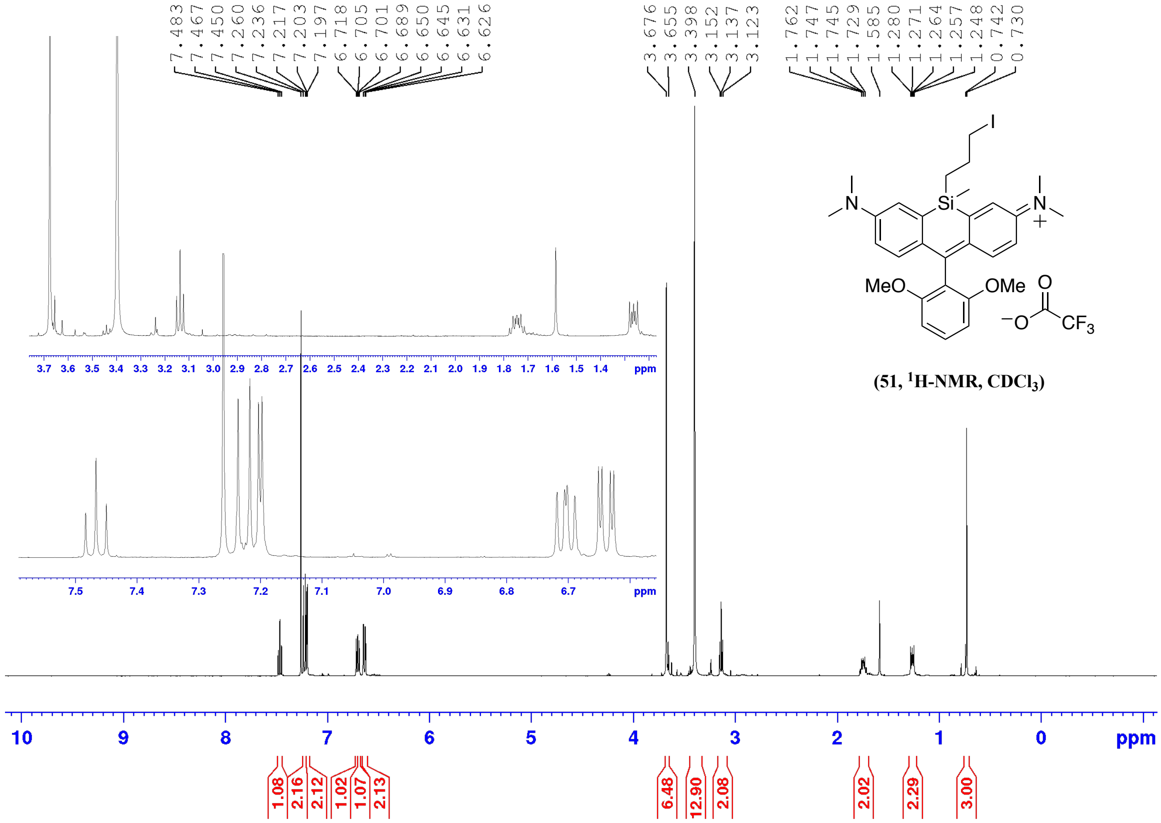

(51, ^{1}H-NMR, $CDCl_3$)

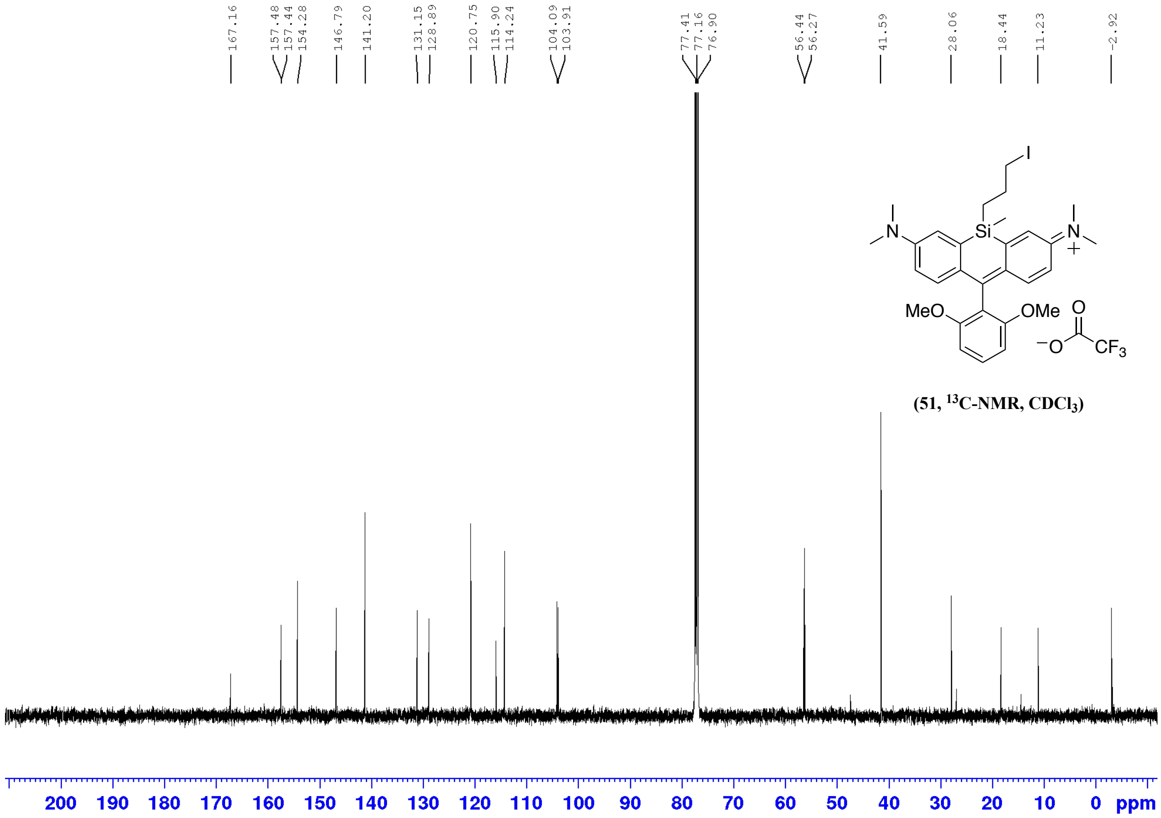

(51, ^{13}C-NMR, $CDCl_3$)

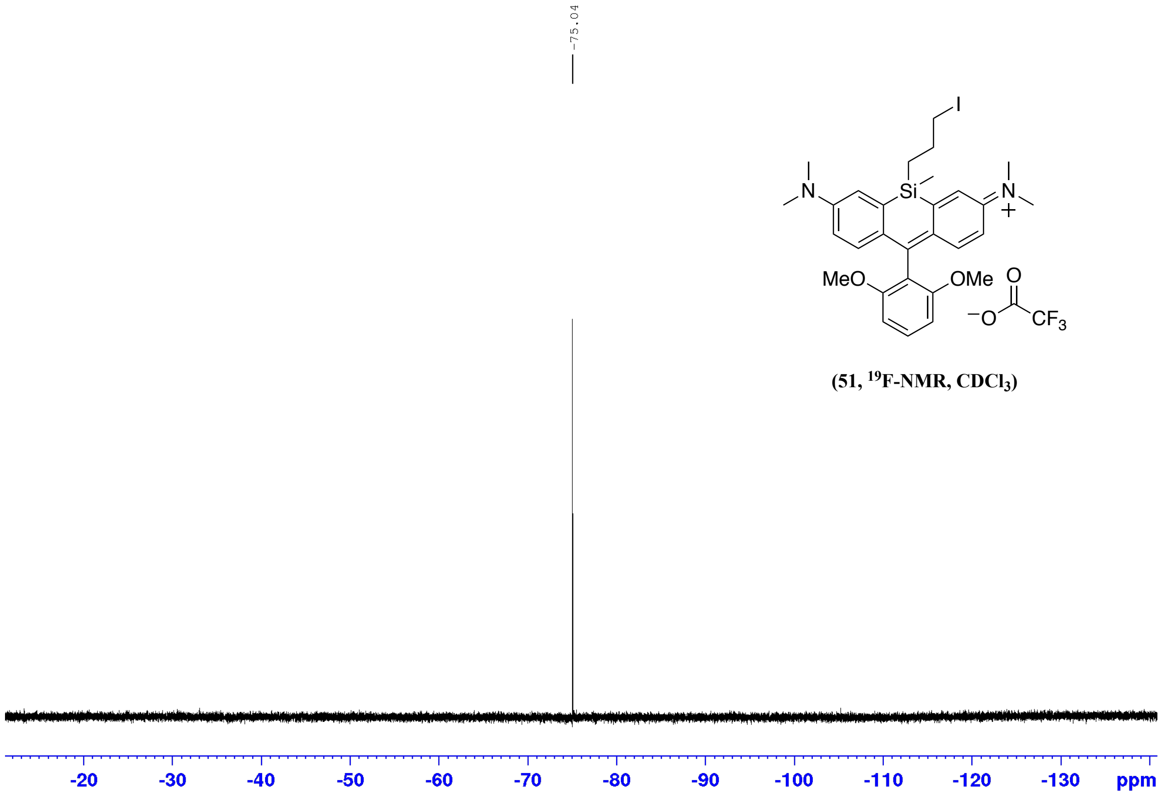

(51, ^{19}F-NMR, $CDCl_3$)

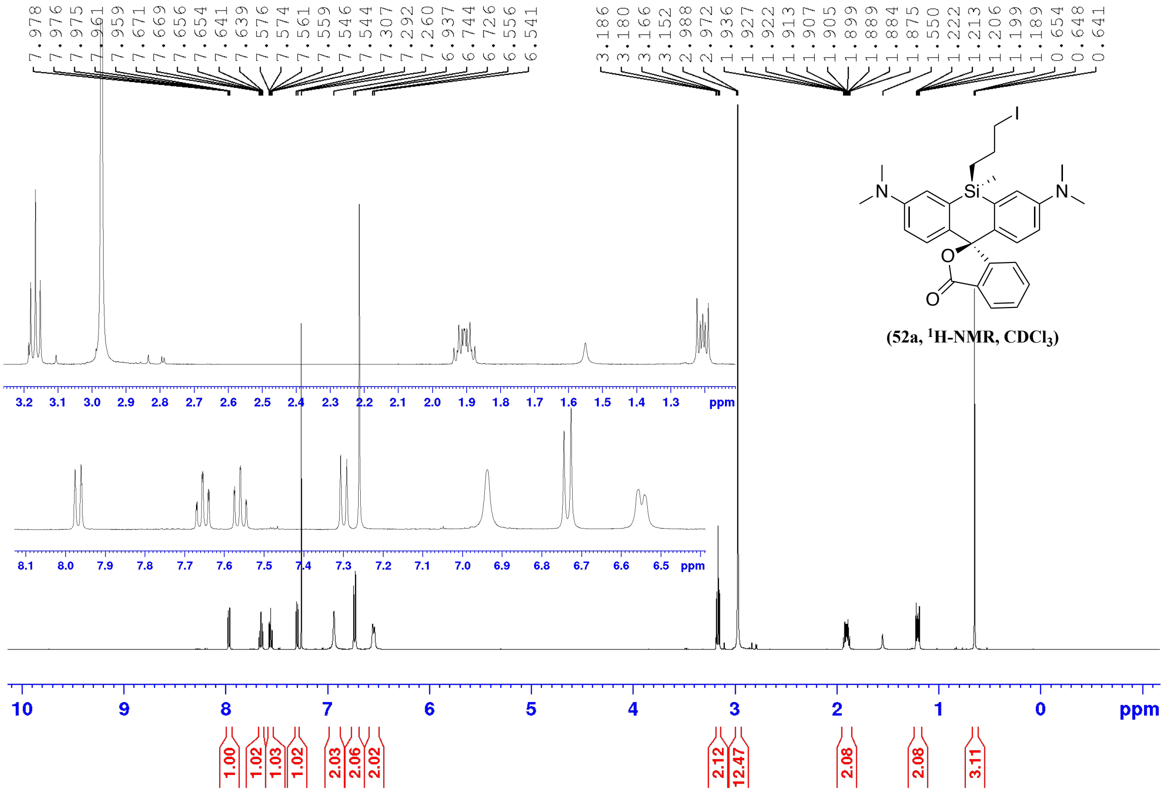

(52a, ^{1}H-NMR, $CDCl_3$)

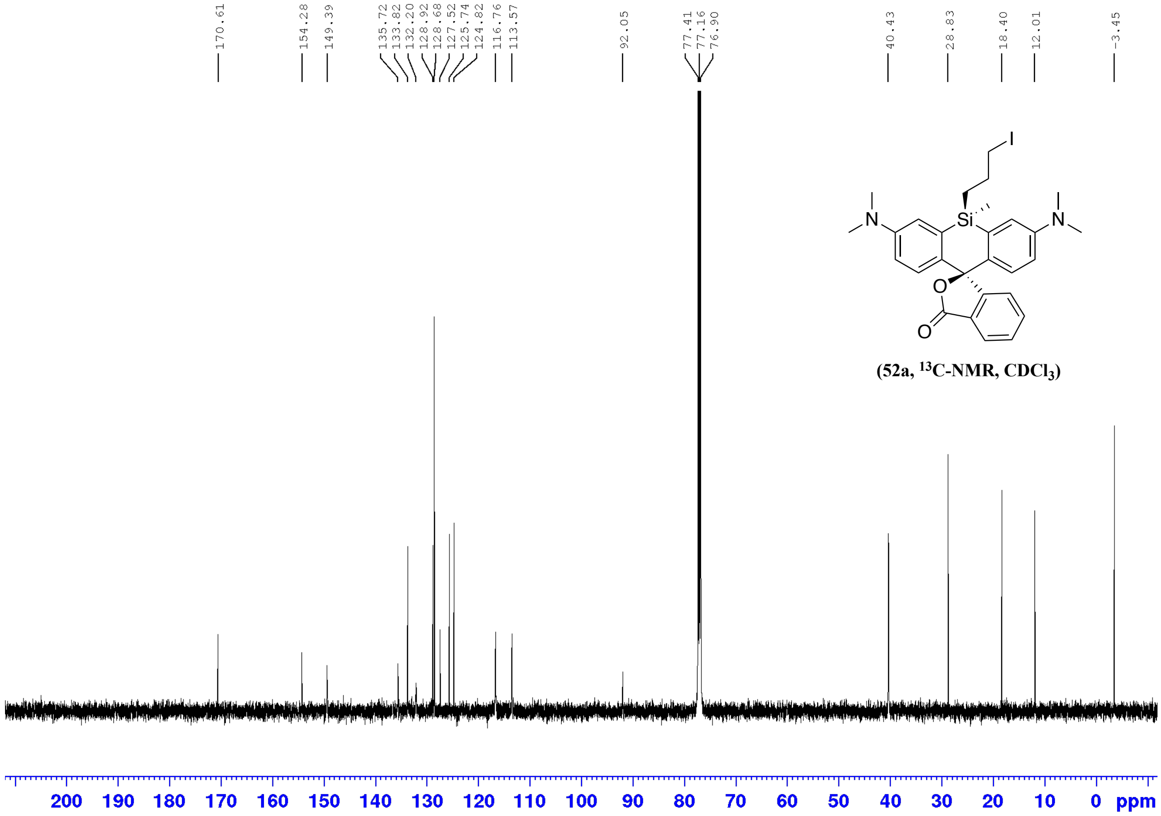

(52a, ^{13}C-NMR, $CDCl_3$)

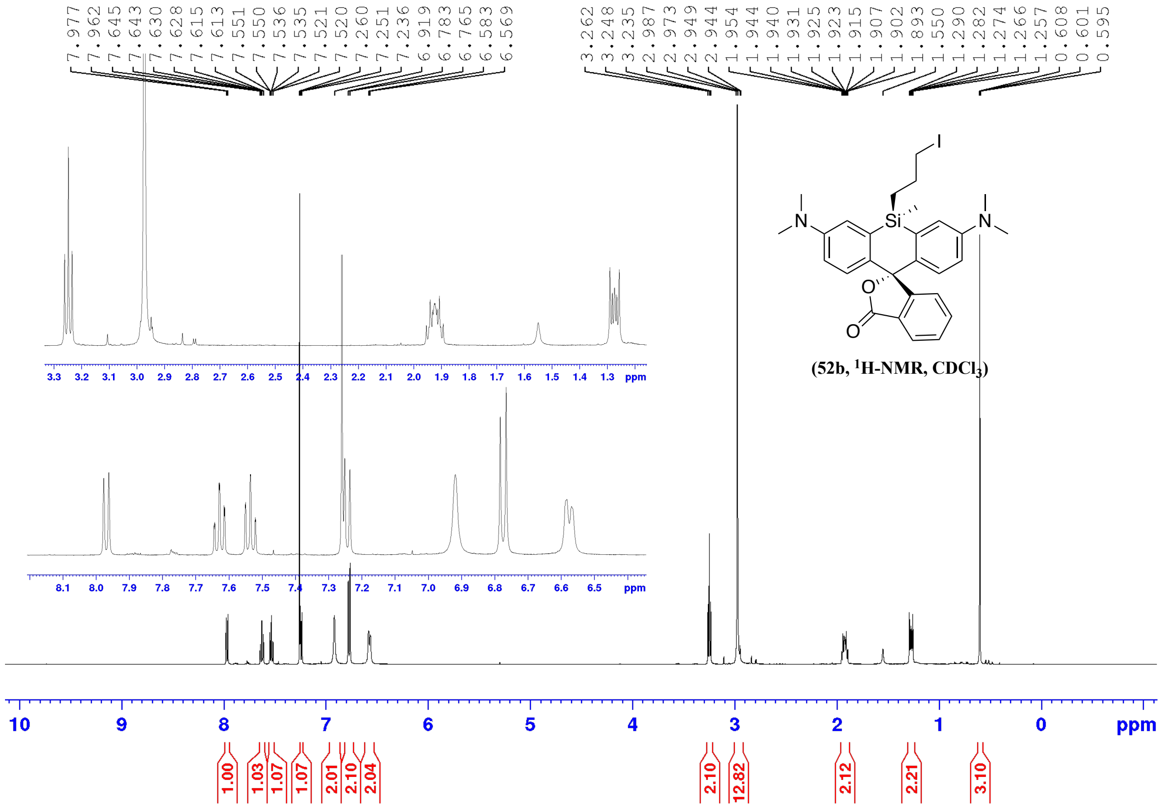

(52b, ^{1}H-NMR, $CDCl_3$)

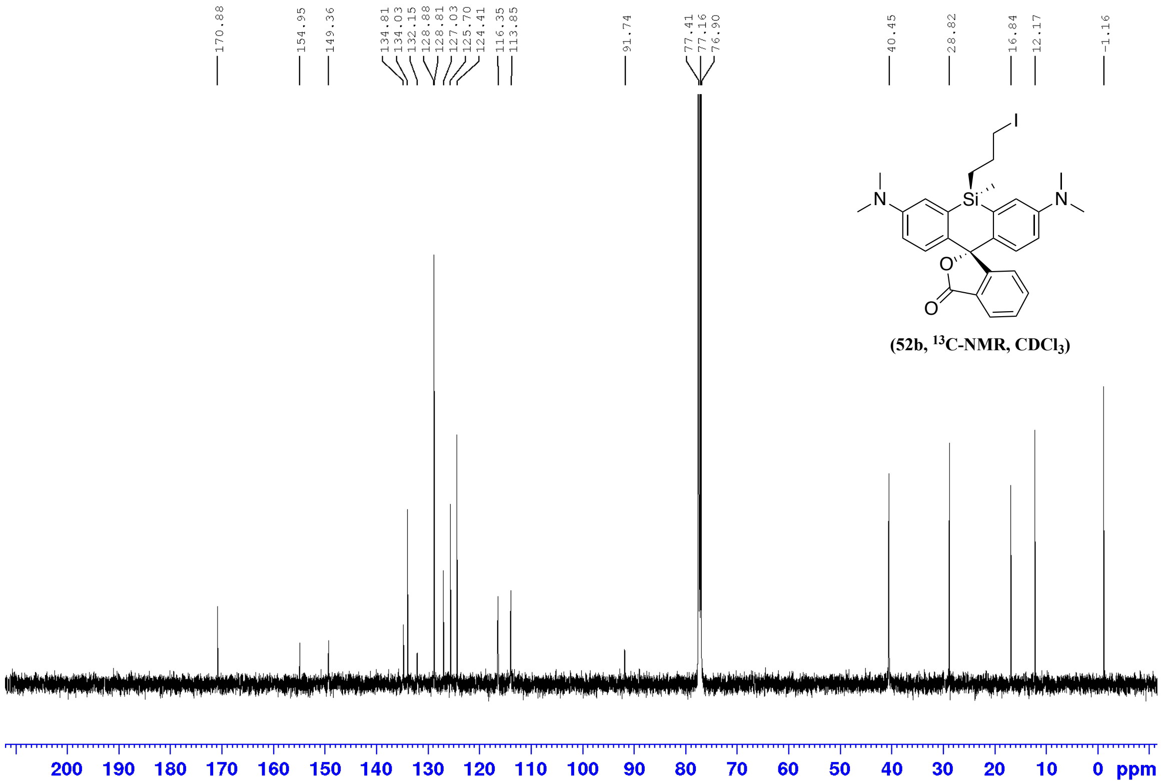

(52b, ^{13}C-NMR, $CDCl_3$)

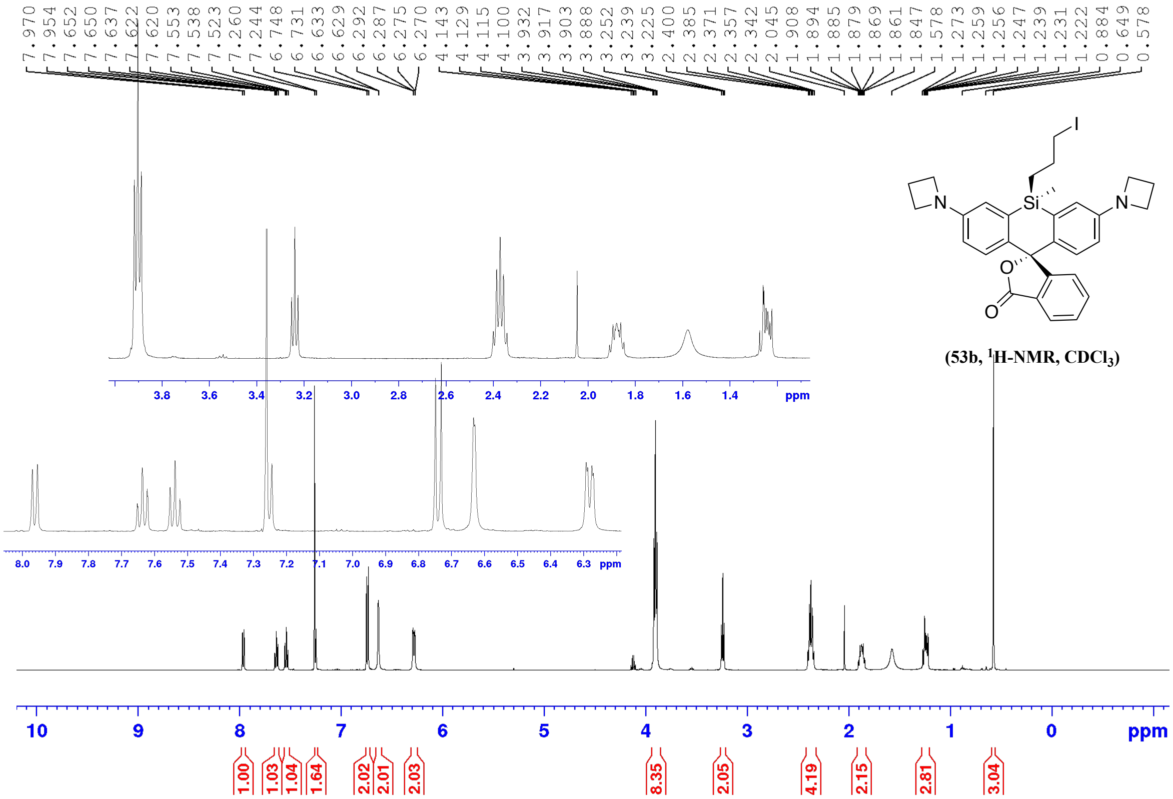

(53b, ^{1}H-NMR, $CDCl_3$)

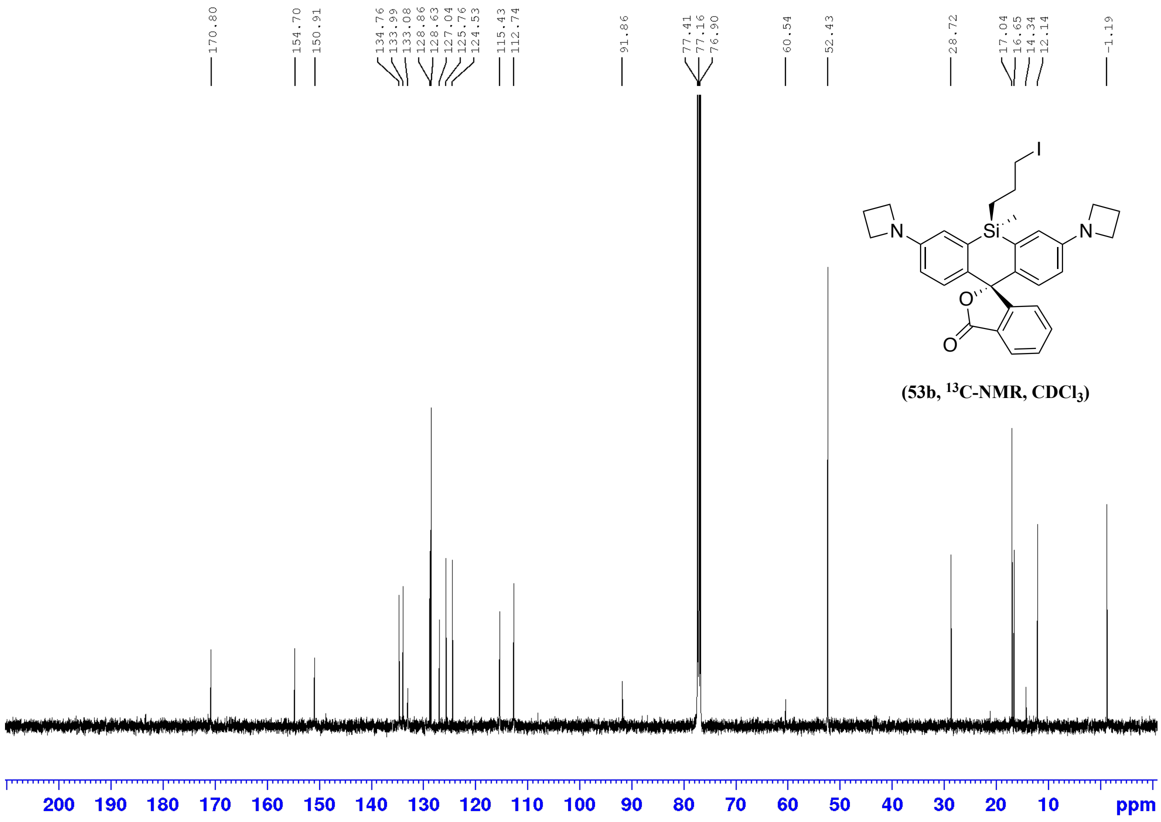

(53b, ^{13}C-NMR, $CDCl_3$)

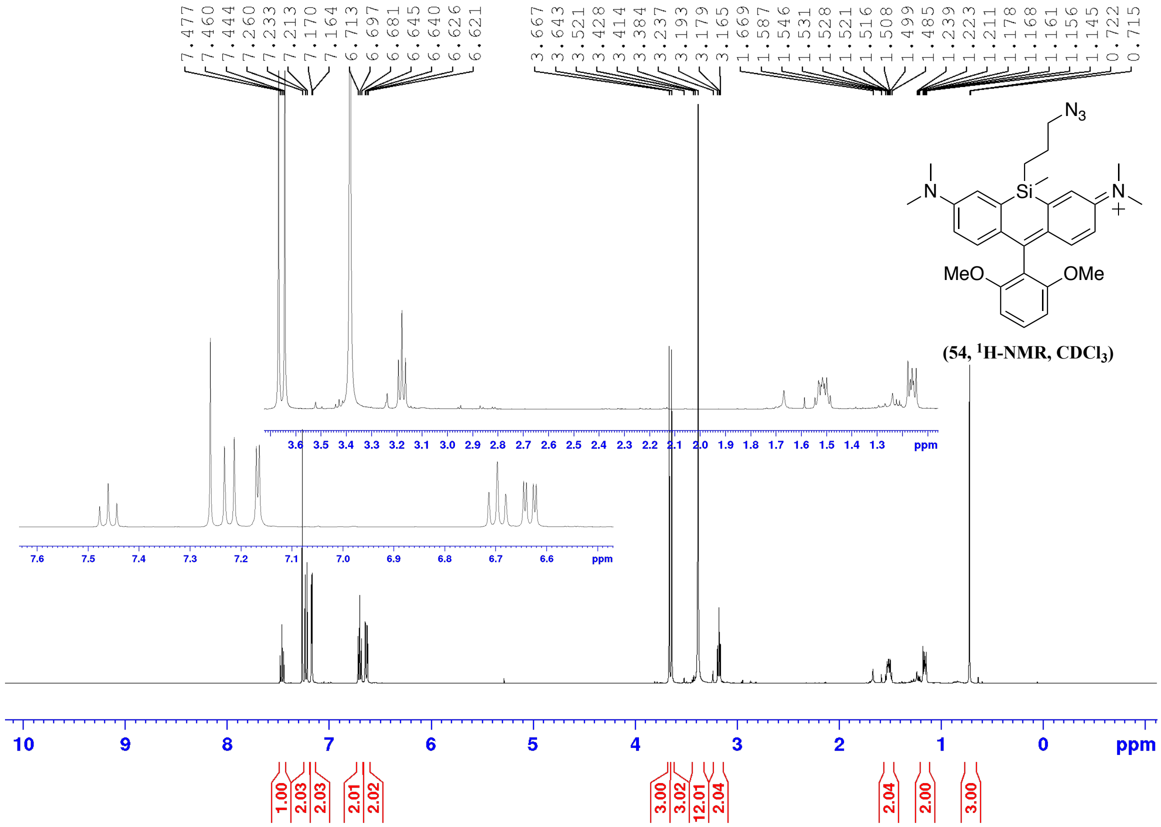
(54, ^{1}H-NMR, CDCl$_3$)

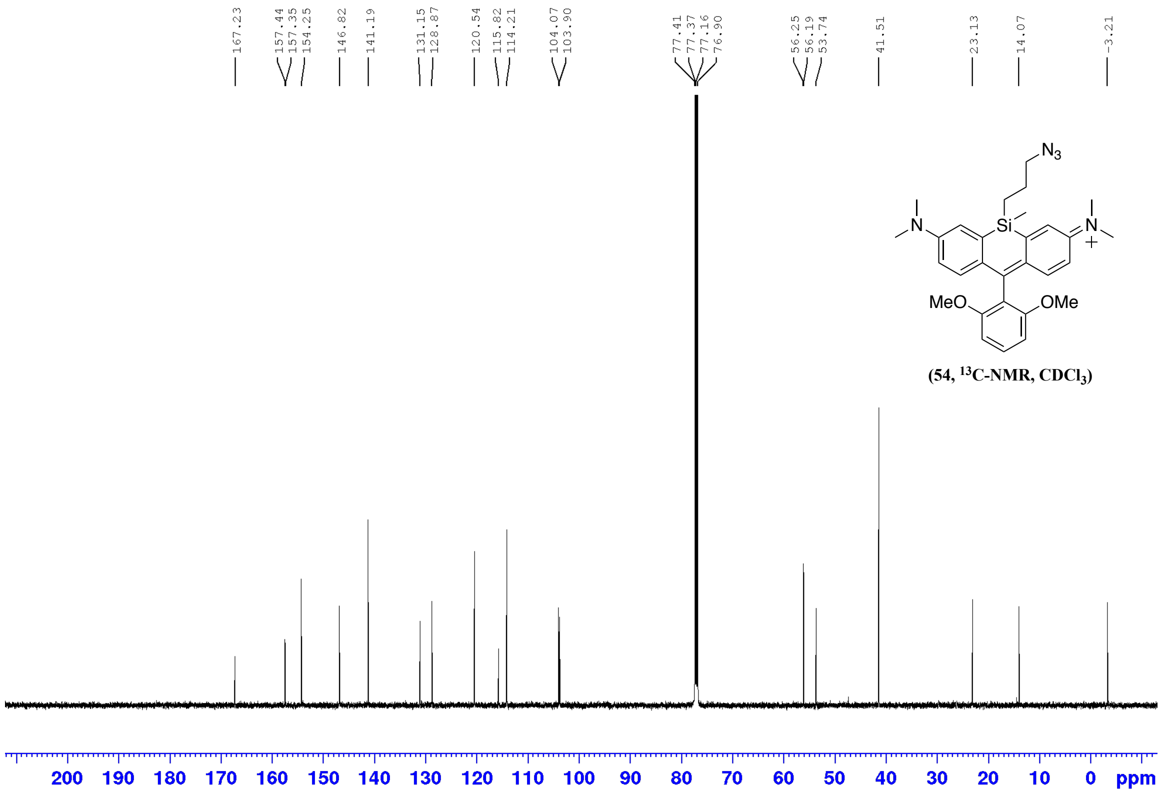
(54, ^{13}C-NMR, CDCl$_3$)

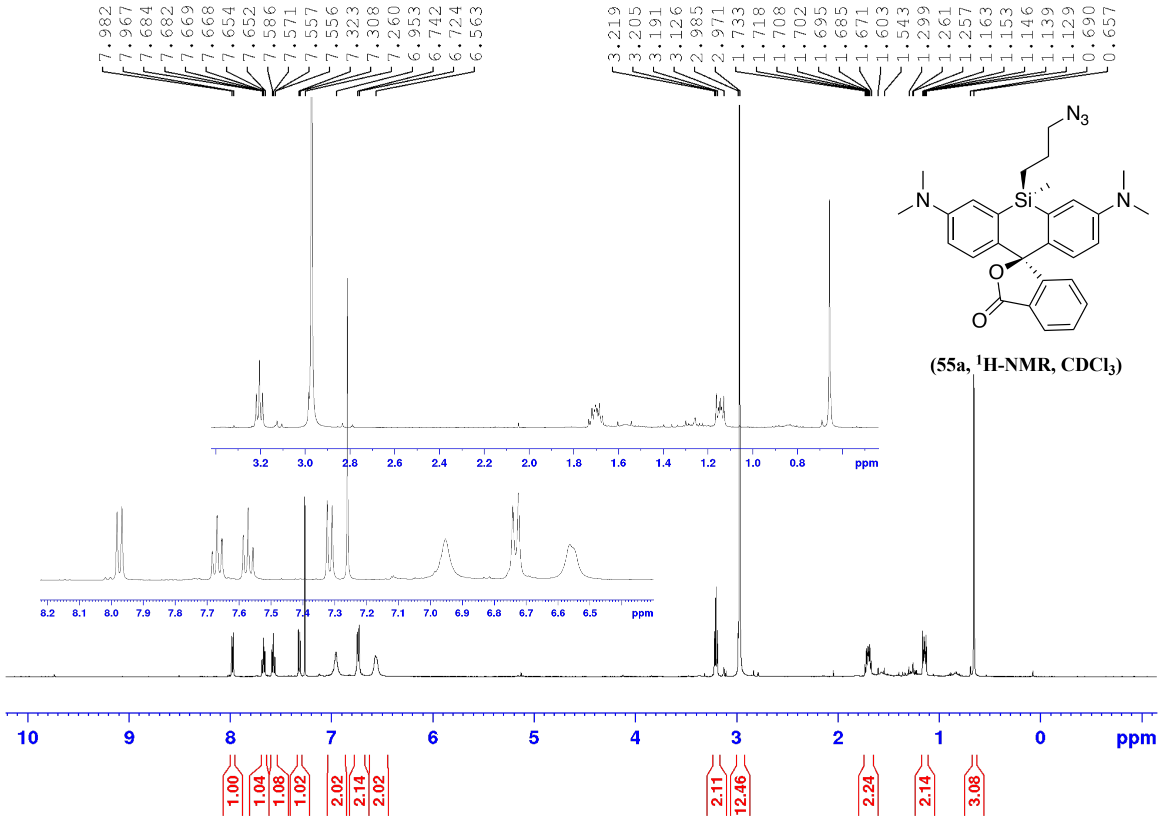
7.982 7.967 7.684 7.682 7.669 7.668 7.654 7.652 7.586 7.571 7.557 7.556 7.323 7.308 7.260 6.953 6.742 6.724 6.563
3.219 3.205 3.191 3.126 2.985 2.971 1.733 1.718 1.708 1.702 1.695 1.685 1.671 1.603 1.543 1.299 1.261 1.257 1.163 1.153 1.146 1.139 1.129 0.690 0.657
1.00 1.04 1.08 1.02 2.02 2.14 2.02 2.11 12.46 2.24 2.14 3.08
ppm
N3
N
Si
O
(55a, 1H-NMR, CDCl3)

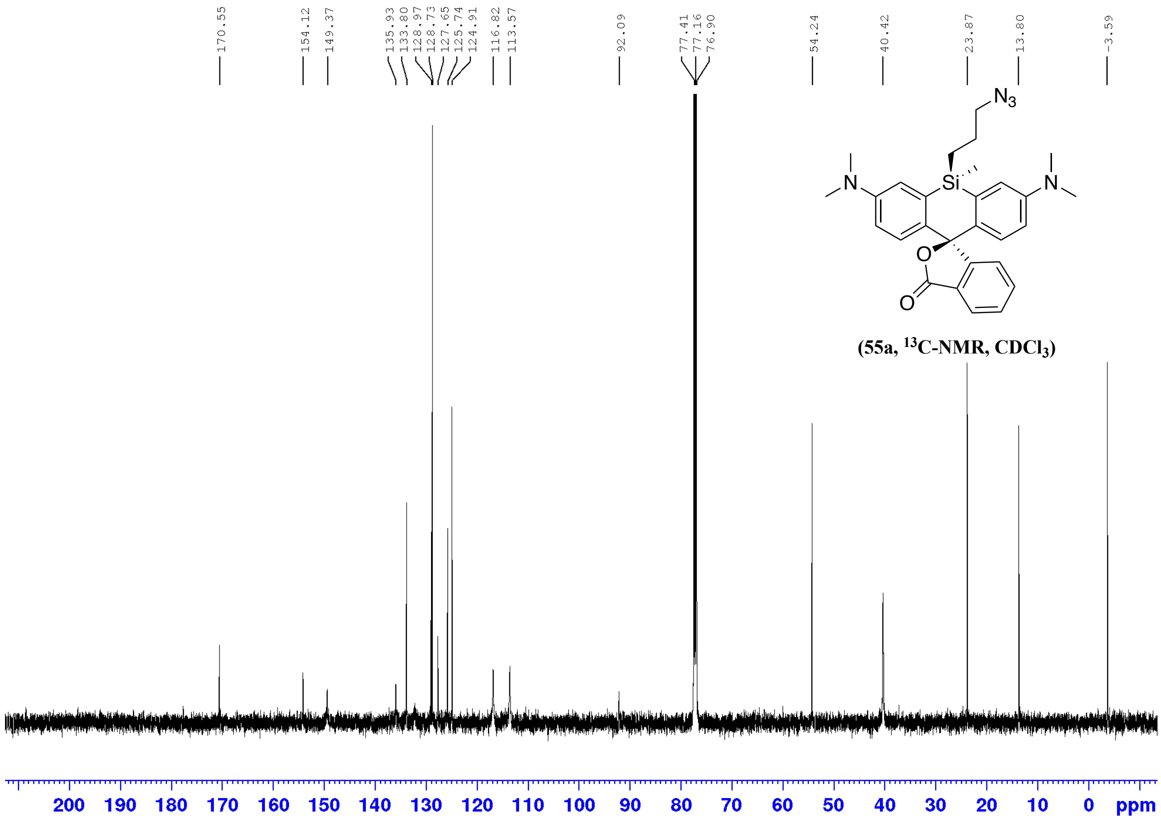
170.55 154.12 149.37 135.93 133.80 128.97 128.73 127.65 125.74 124.91 116.82 113.57 92.09 77.41 77.16 76.90 54.24 40.42 23.87 13.80 -3.59
ppm
N3
N
Si
O
(55a, 13C-NMR, CDCl3)

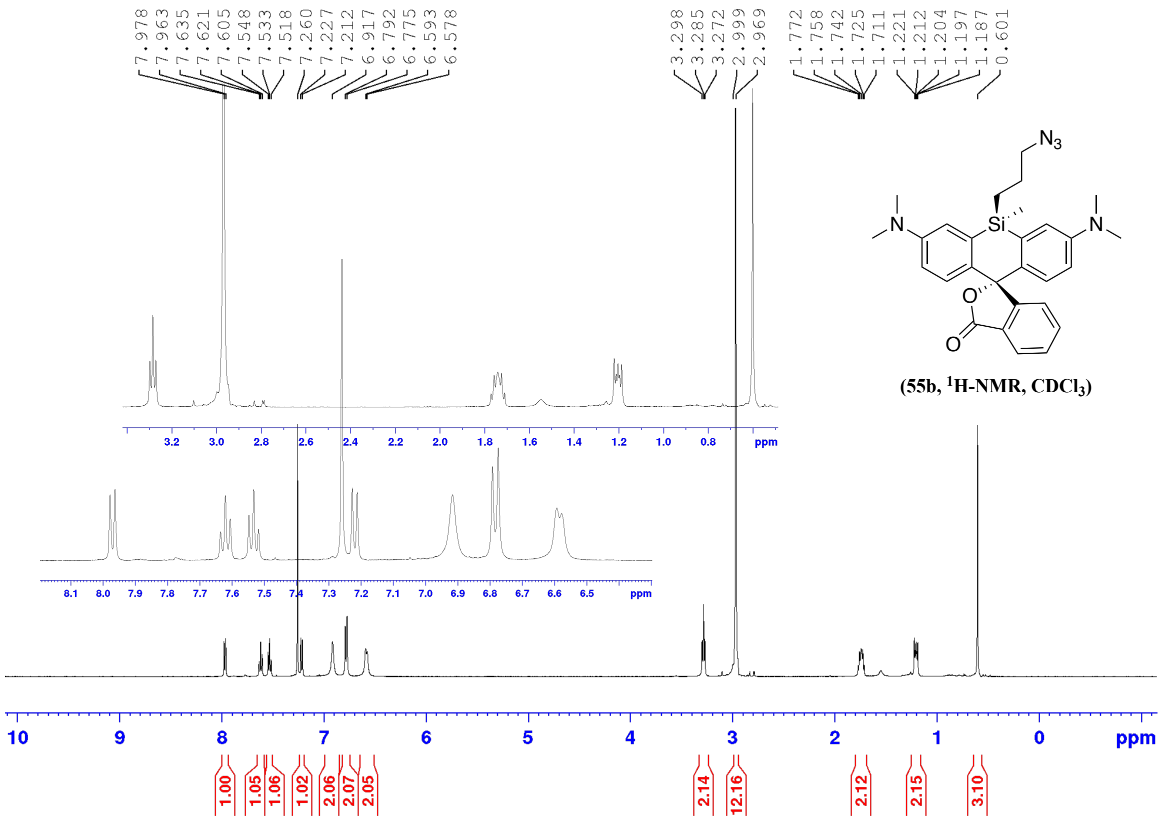
7.978 7.963 7.635 7.621 7.605 7.548 7.533 7.518 7.260 7.227 7.212 6.917 6.792 6.775 6.593 6.578
3.298 3.285 3.272 2.999 2.969
1.772 1.758 1.742 1.725 1.711 1.221 1.212 1.204 1.197 1.187 0.601
N3
Si
N
O
(55b, ¹H-NMR, CDCl3)
1.00 1.05 1.06 1.02 2.06 2.07 2.05 2.14 12.16 2.12 2.15 3.10
ppm

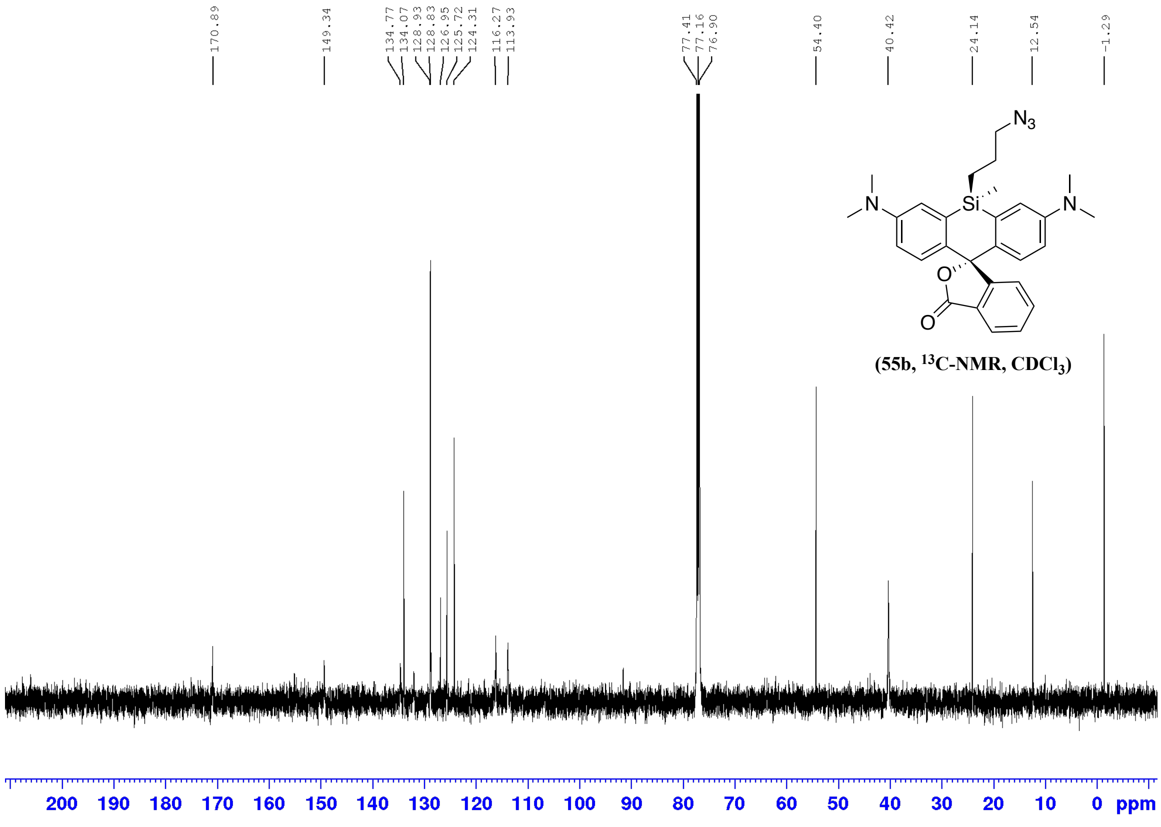
170.89
149.34
134.77 134.07 128.93 128.83 126.95 125.72 124.31
116.27 113.93
77.41 77.16 76.90
54.40
40.42
24.14
12.54
-1.29
N3
Si
N
O
(55b, ¹³C-NMR, CDCl3)
ppm

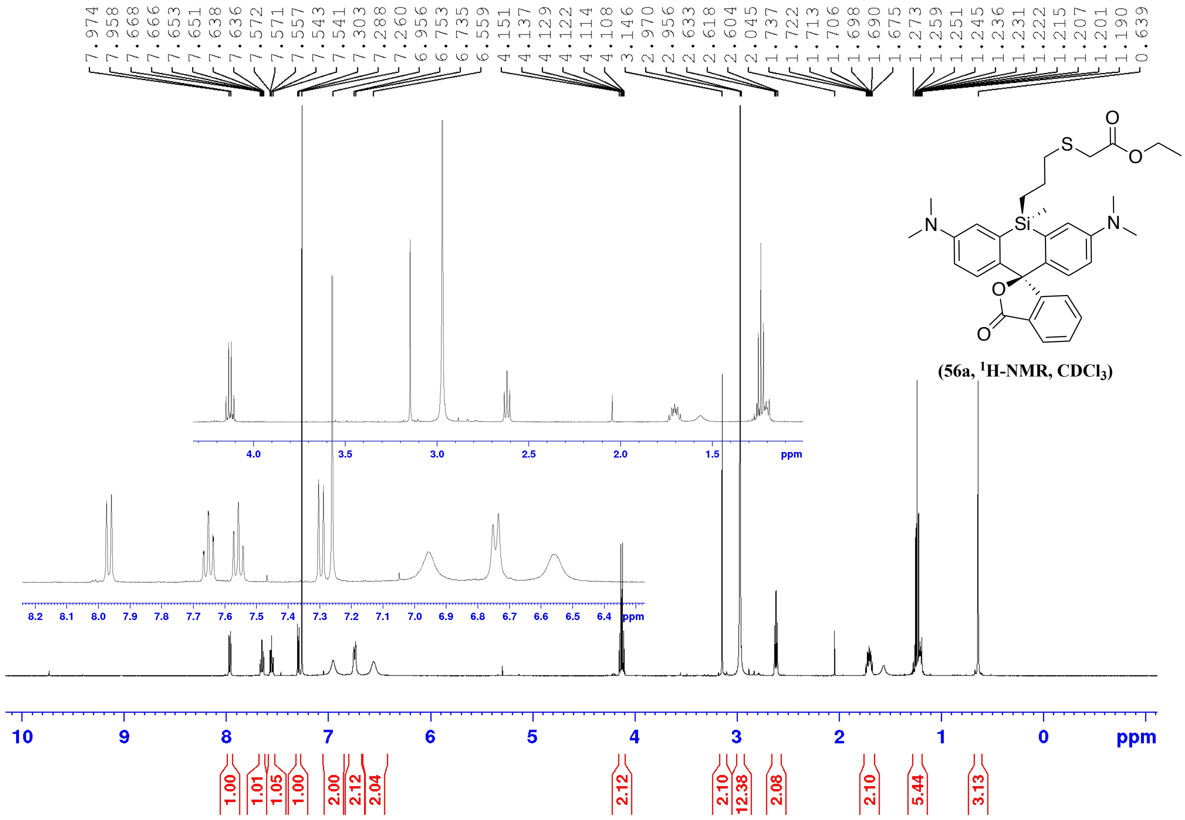
7.974 7.958 7.668 7.666 7.653 7.651 7.638 7.636 7.572 7.571 7.557 7.543 7.541 7.303 7.288 7.260 6.956 6.753 6.735 6.559 4.151 4.137 4.129 4.122 4.114 4.108 4.146 3.140 2.970 2.956 2.633 2.618 2.604 2.045 1.737 1.722 1.713 1.706 1.698 1.690 1.675 1.273 1.259 1.251 1.245 1.236 1.231 1.222 1.215 1.207 1.201 1.190 0.639
(56a, 1H-NMR, CDCl3)
1.00 1.01 1.05 1.00 2.00 2.12 2.04 2.12 2.10 12.38 2.08 2.10 5.44 3.13
ppm

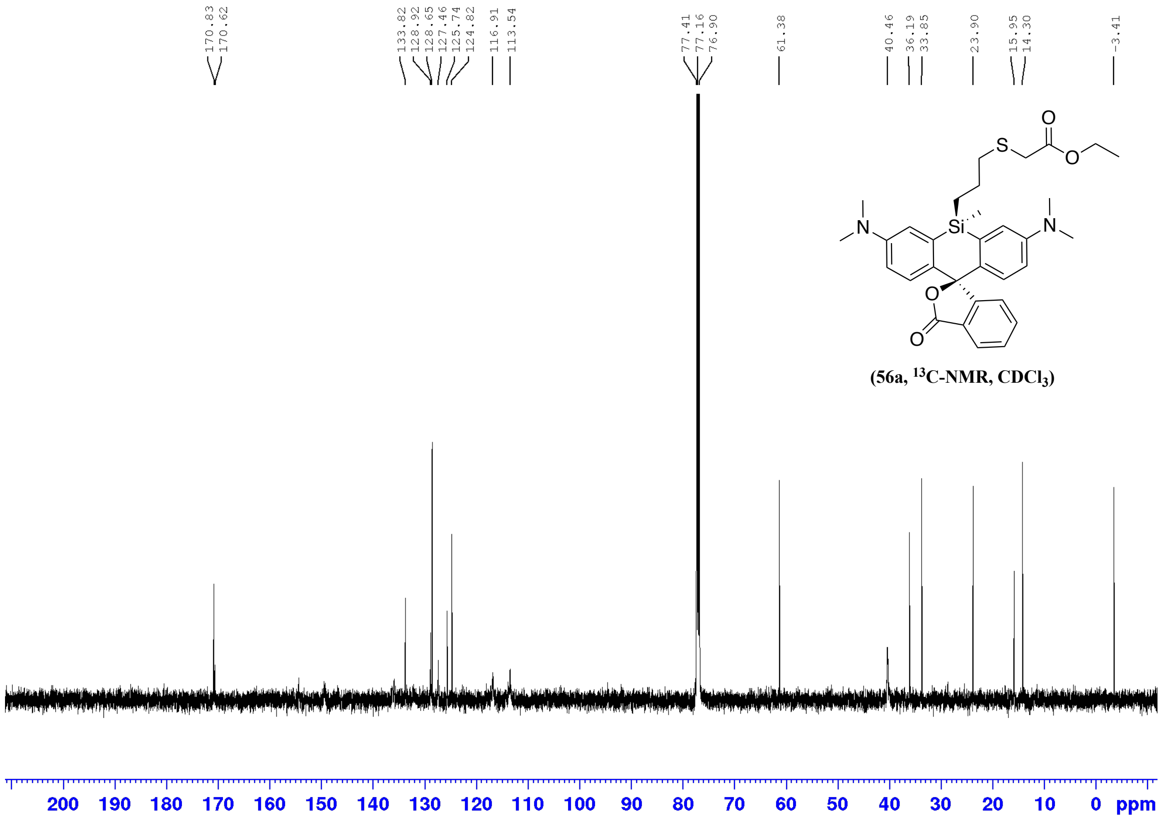
170.83 170.62 133.82 128.92 128.65 127.46 125.74 124.82 116.91 113.54 77.41 77.16 76.90 61.38 40.46 36.19 33.85 23.90 15.95 14.30 -3.41
(56a, 13C-NMR, CDCl3)
ppm

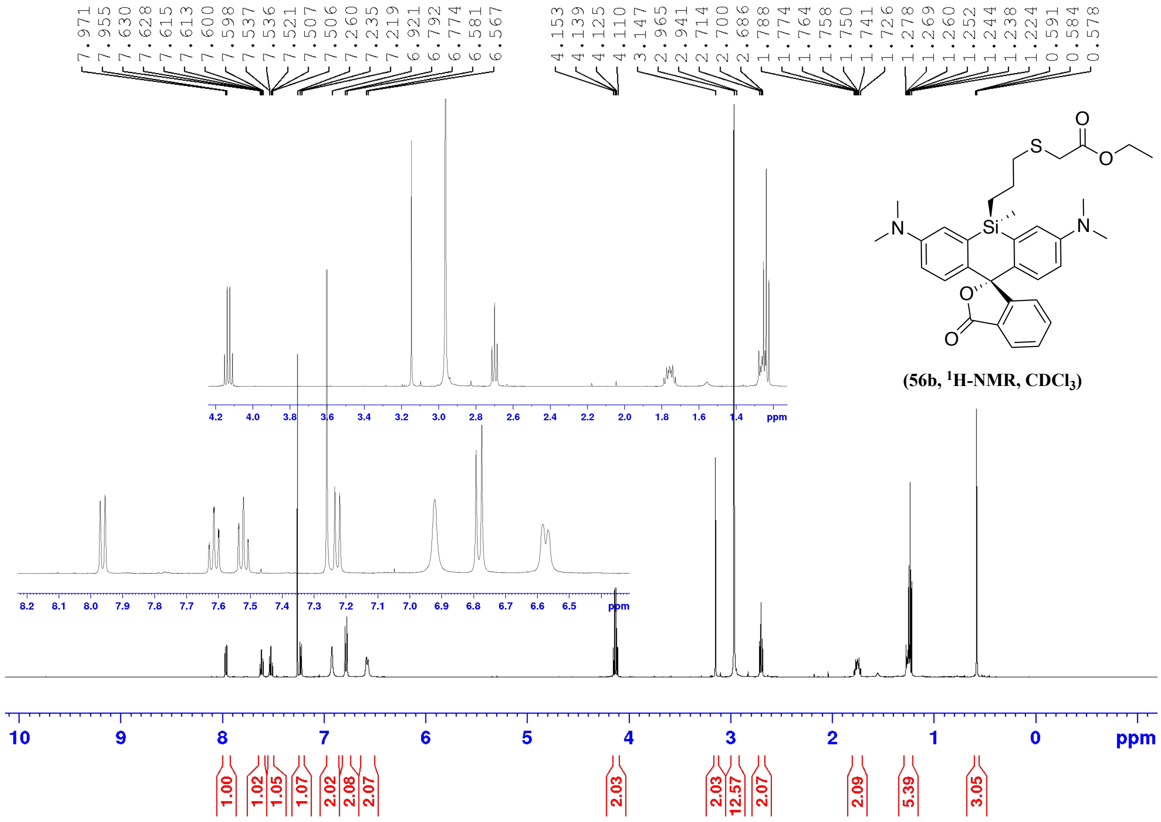

(56b, ^{1}H-NMR, $CDCl_3$)

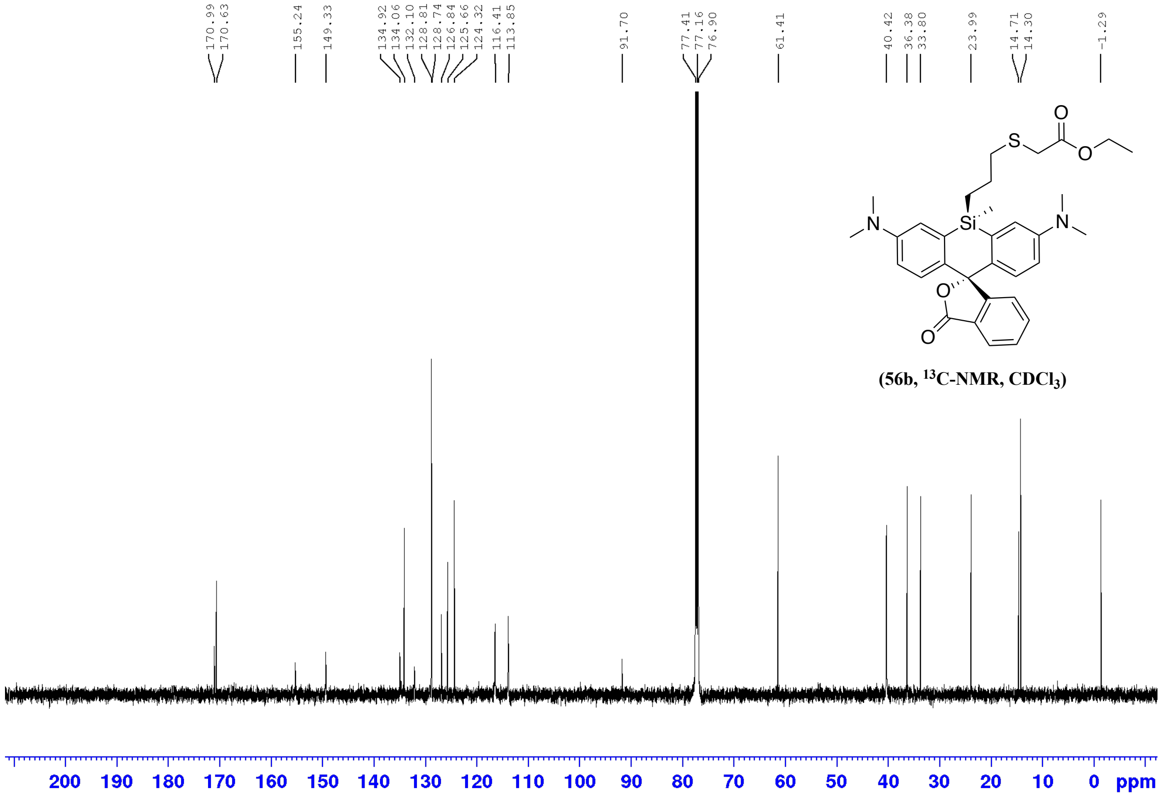

(56b, ^{13}C-NMR, $CDCl_3$)

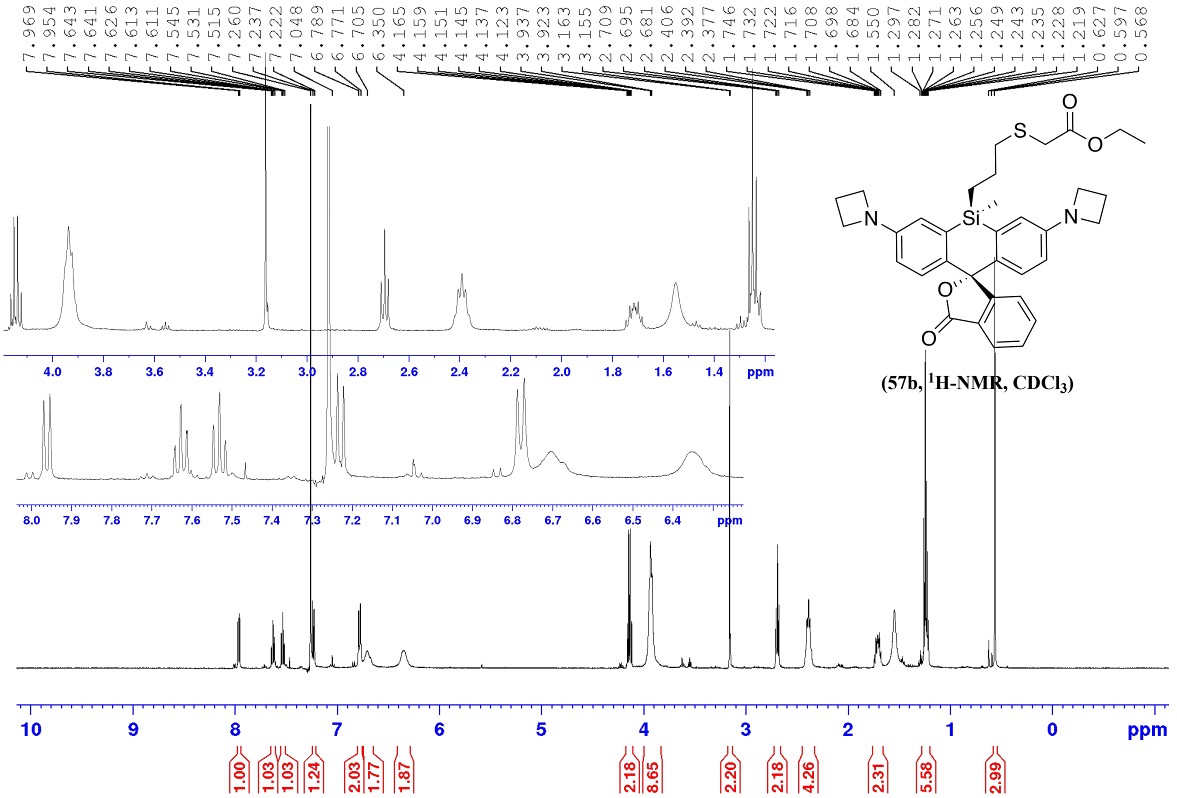

(57b, ^{1}H-NMR, $CDCl_3$)

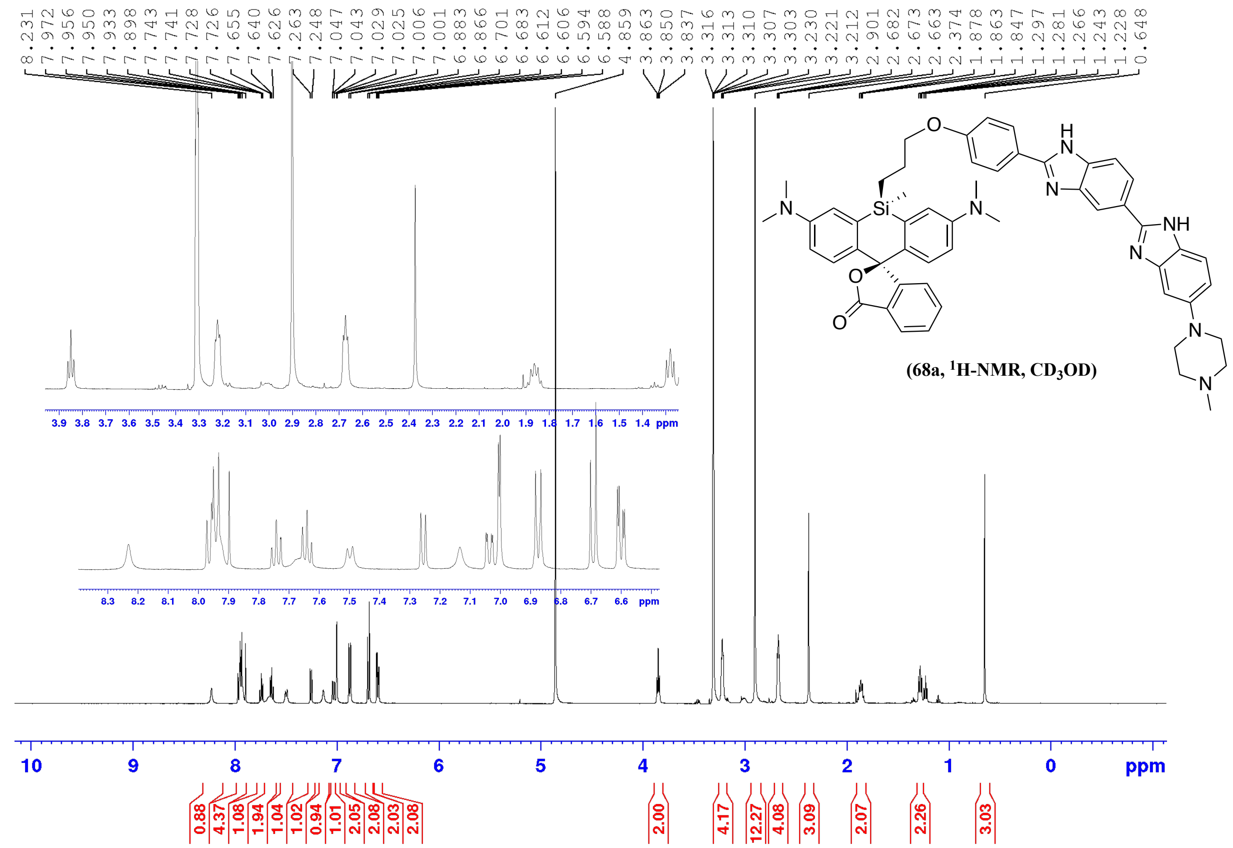

(68a, ^{1}H-NMR, CD_3OD)

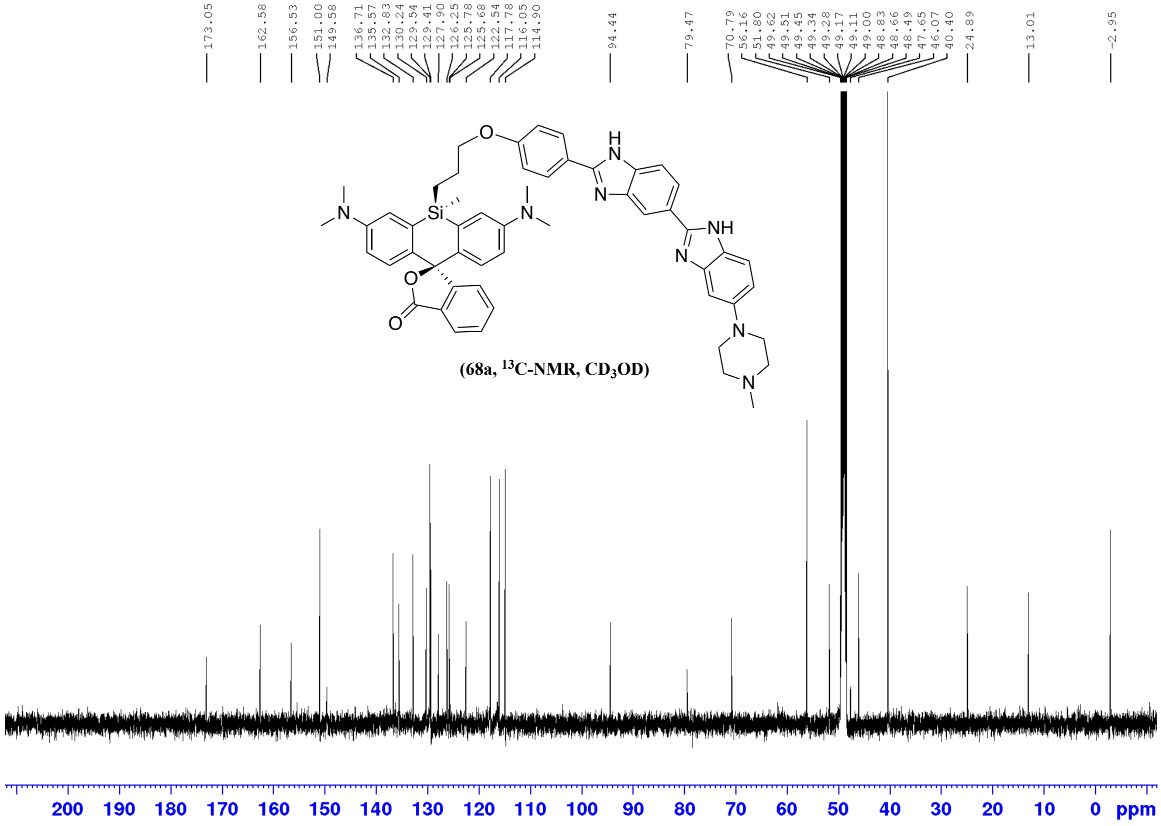

(68a, ^{13}C-NMR, CD_3OD)

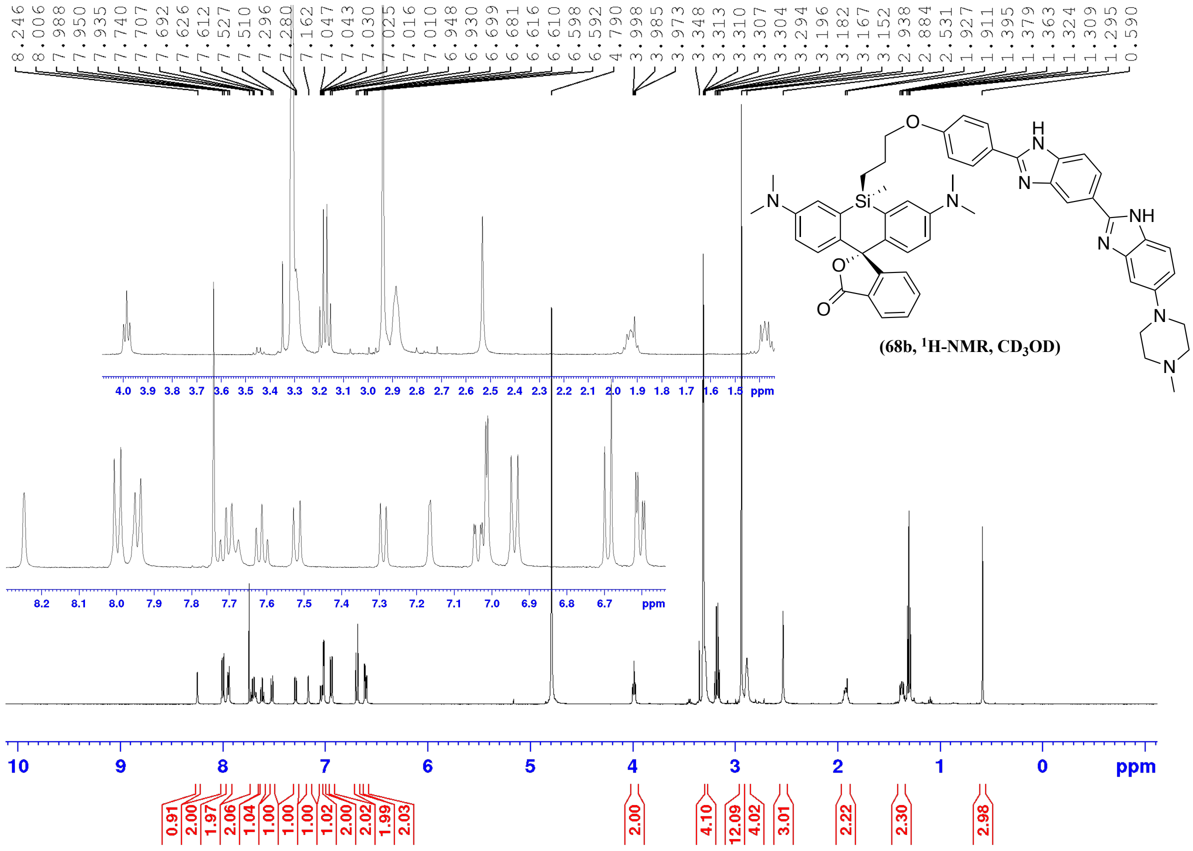

(68b, ^{1}H-NMR, CD_3OD)

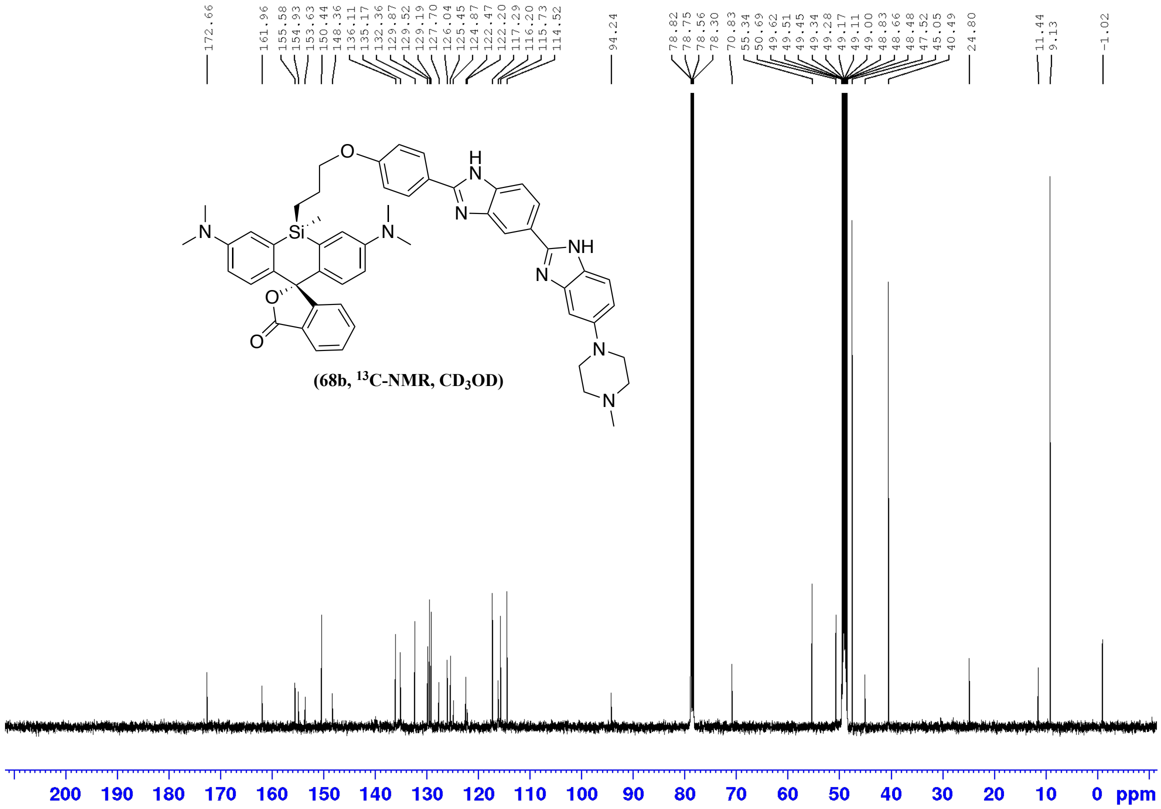

172.66
161.96
155.58
154.93
153.63
150.44
148.36
136.11
135.17
132.36
129.87
129.52
129.19
127.70
126.04
125.45
124.87
122.47
122.20
117.29
116.20
115.73
114.52
94.24
78.82
78.75
78.56
78.30
70.83
55.34
50.69
49.62
49.51
49.45
49.34
49.28
49.17
49.11
49.00
48.83
48.66
48.48
47.52
45.05
40.49
24.80
11.44
9.13
-1.02
(68b, 13C-NMR, CD3OD)
200 190 180 170 160 150 140 130 120 110 100 90 80 70 60 50 40 30 20 10 0 ppm

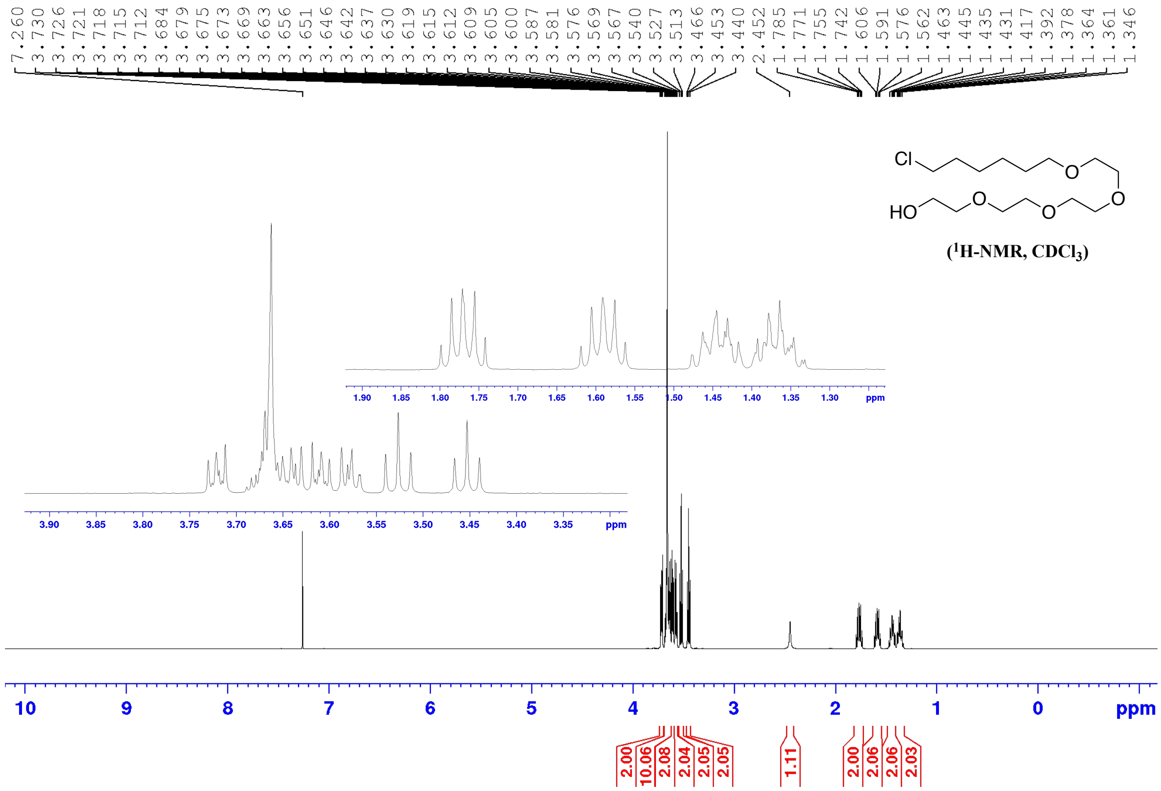

7.260
3.730
3.726
3.721
3.718
3.715
3.712
3.684
3.679
3.675
3.673
3.669
3.663
3.656
3.651
3.646
3.642
3.637
3.630
3.619
3.615
3.612
3.609
3.605
3.600
3.587
3.581
3.576
3.569
3.567
3.540
3.527
3.513
3.466
3.453
3.440
2.452
1.785
1.771
1.755
1.742
1.606
1.591
1.576
1.562
1.463
1.445
1.435
1.431
1.417
1.392
1.378
1.364
1.361
1.346
Cl
O
O
HO
O
O
(1H-NMR, CDCl3)
1.90 1.85 1.80 1.75 1.70 1.65 1.60 1.55 1.50 1.45 1.40 1.35 1.30 ppm
3.90 3.85 3.80 3.75 3.70 3.65 3.60 3.55 3.50 3.45 3.40 3.35 ppm
10 9 8 7 6 5 4 3 2 1 0 ppm
2.00
10.06
2.08
2.04
2.05
2.05
1.11
2.00
2.06
2.06
2.03

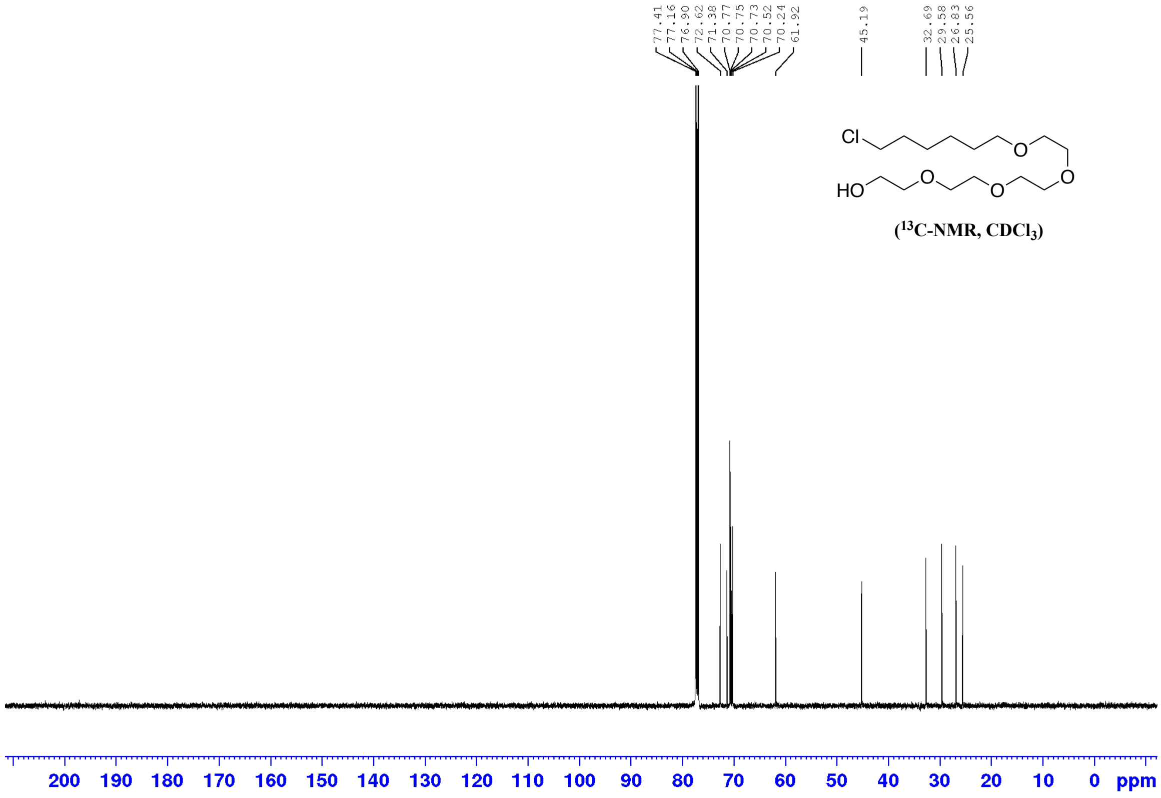
(13C-NMR, CDCl3)

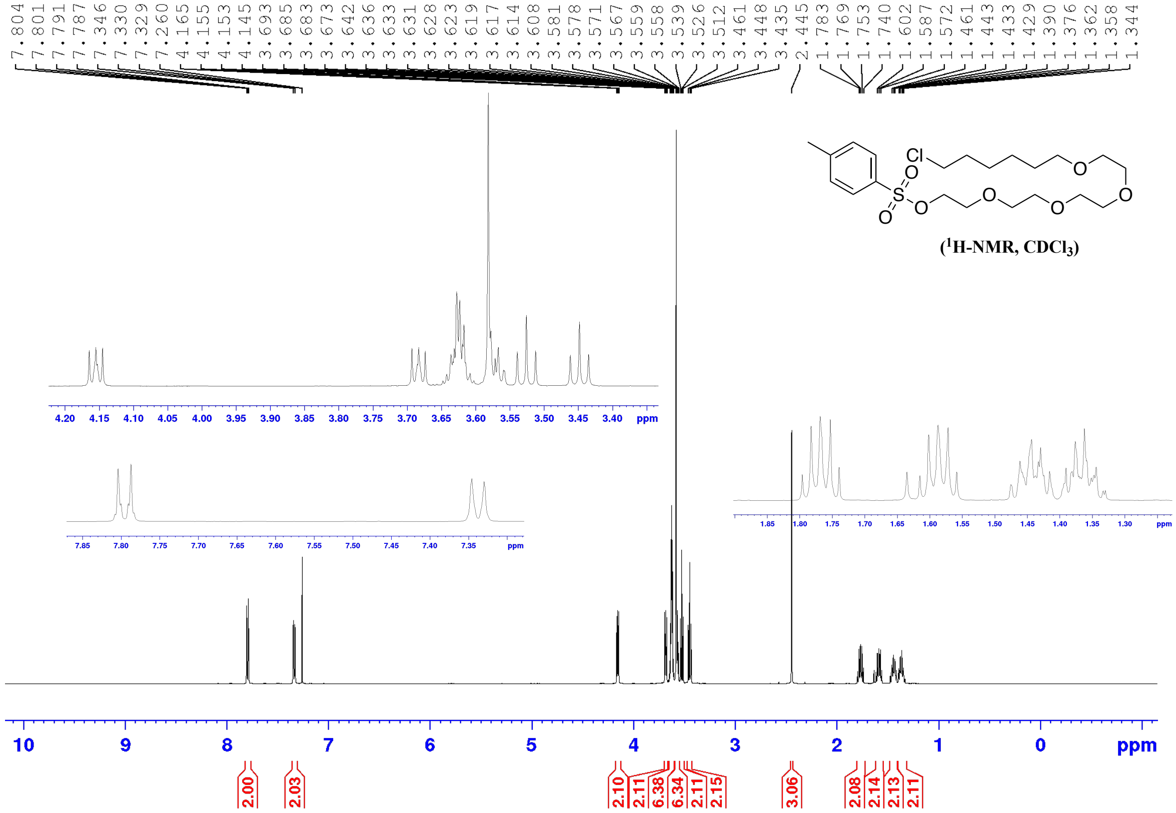
(1H-NMR, CDCl3)

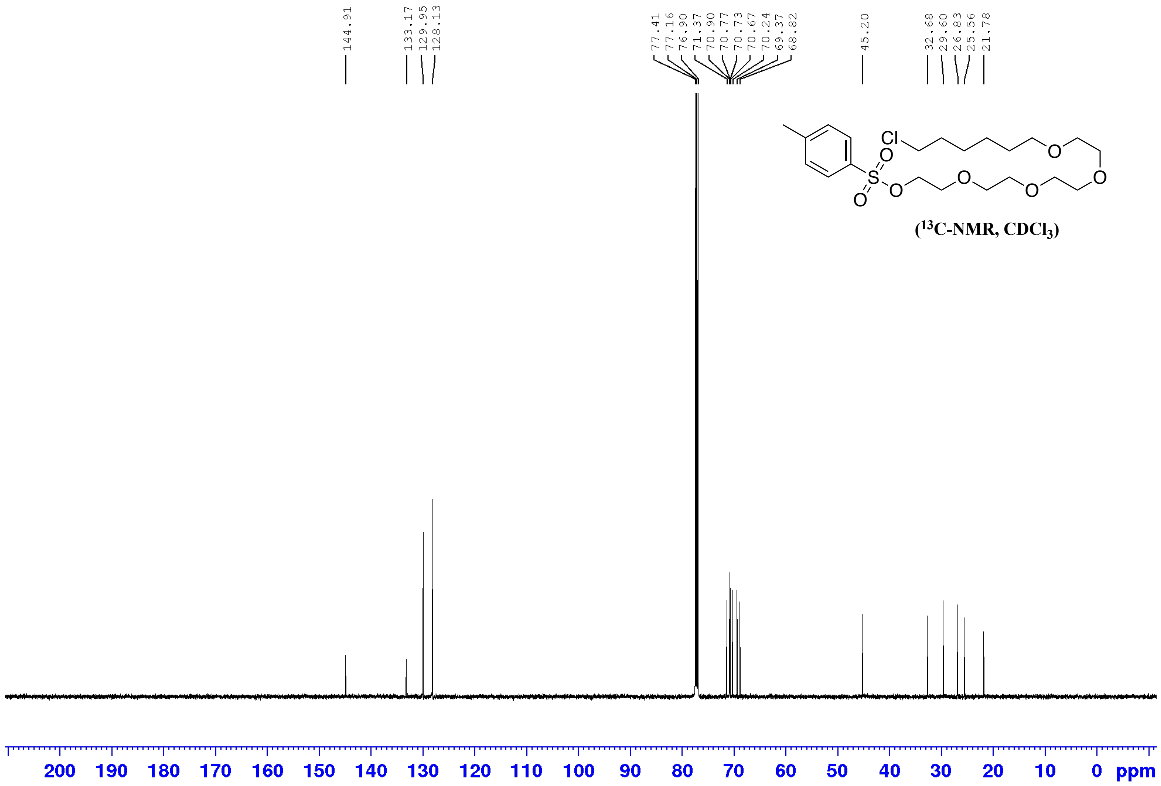

(^{13}C-NMR, $CDCl_3$)

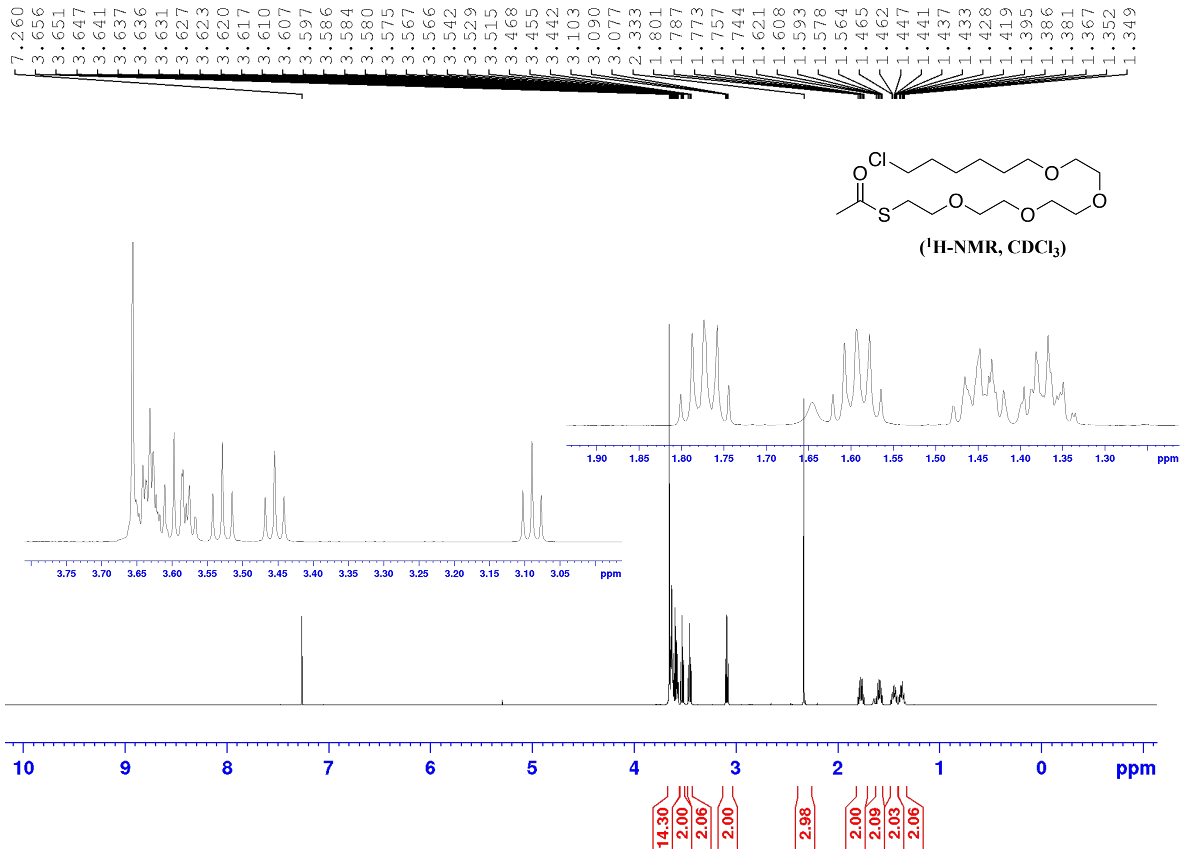

(^{1}H-NMR, $CDCl_3$)

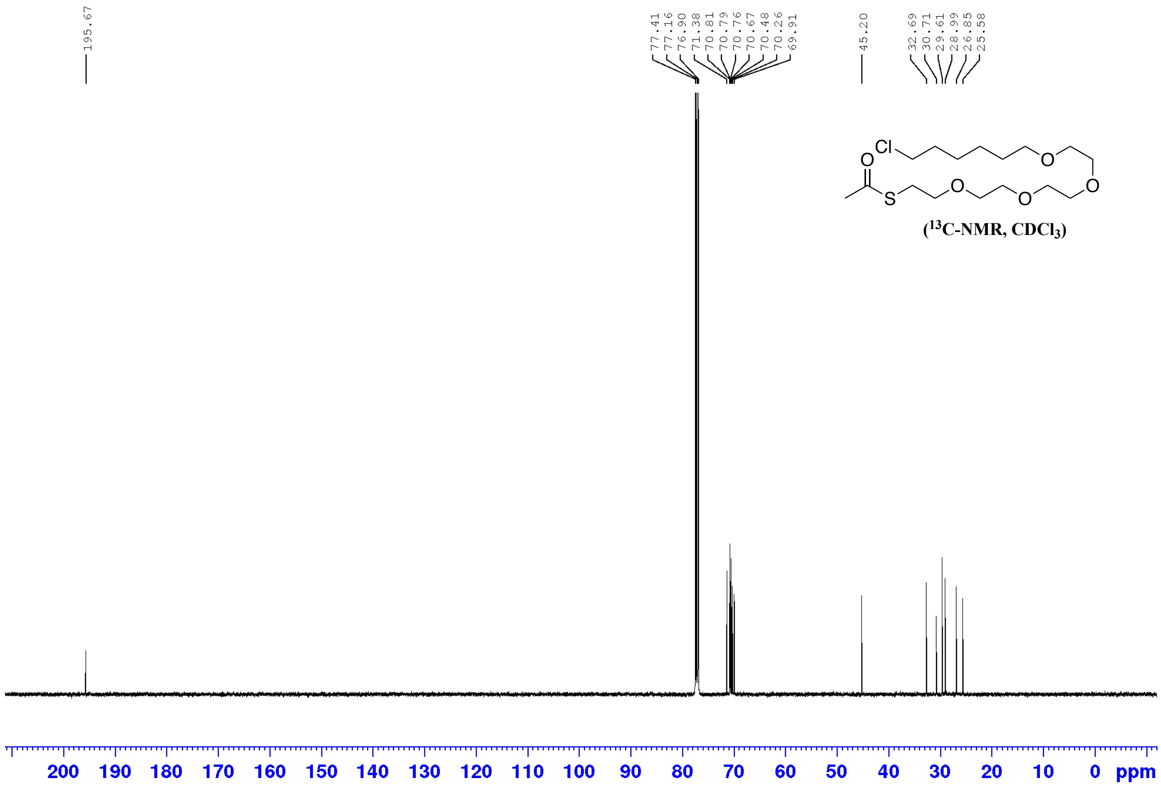
(13C-NMR, CDCl3)

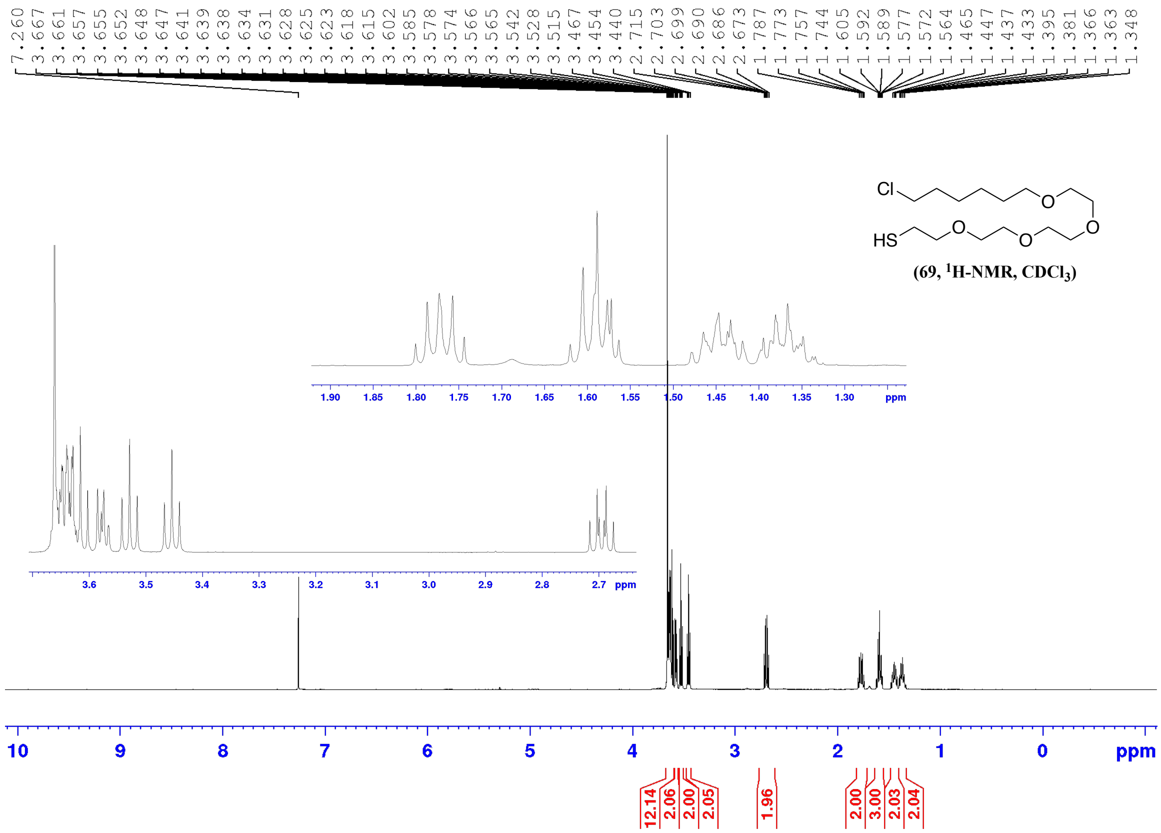
(69, 1H-NMR, CDCl3)

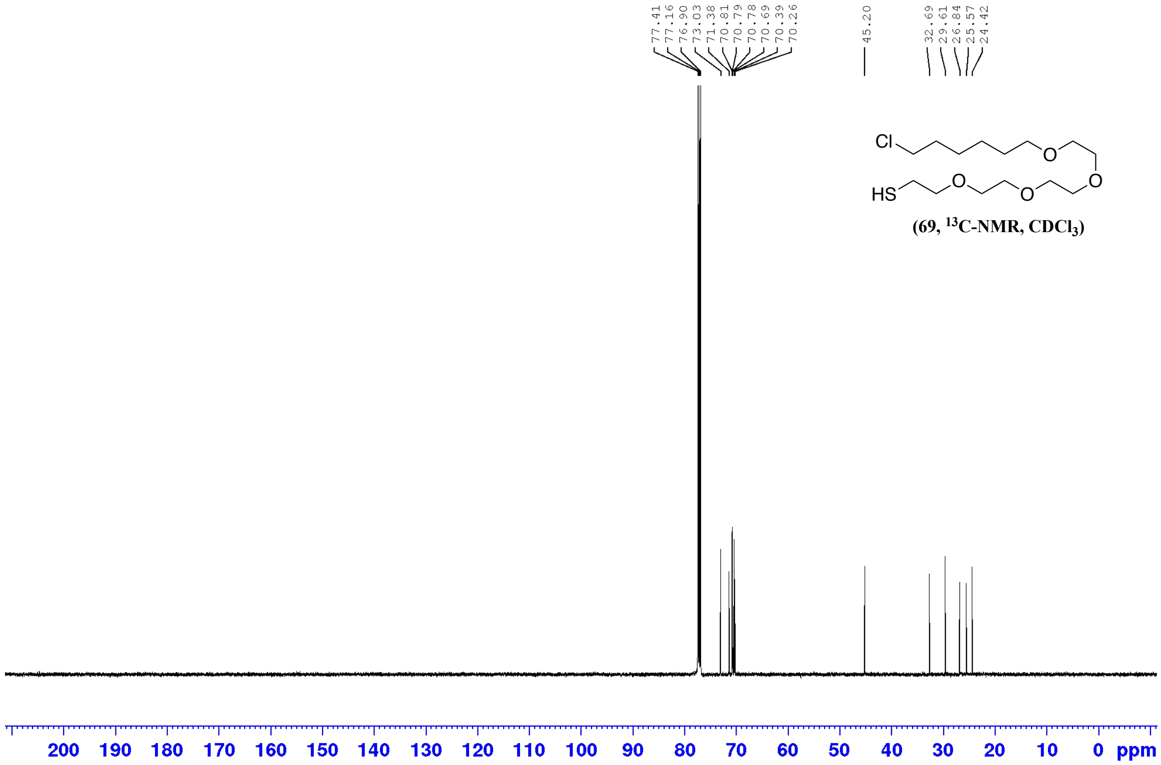

(69, ^{13}C-NMR, $CDCl_3$)

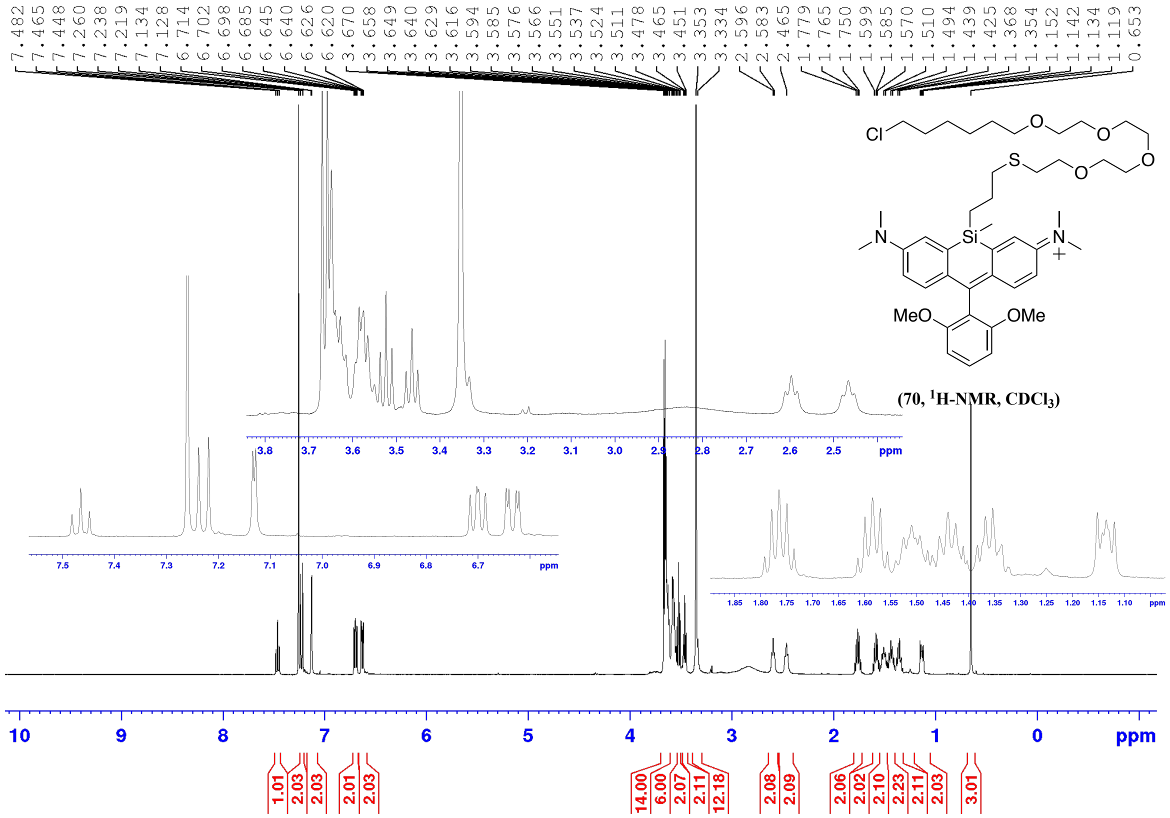

(70, ^{1}H-NMR, $CDCl_3$)

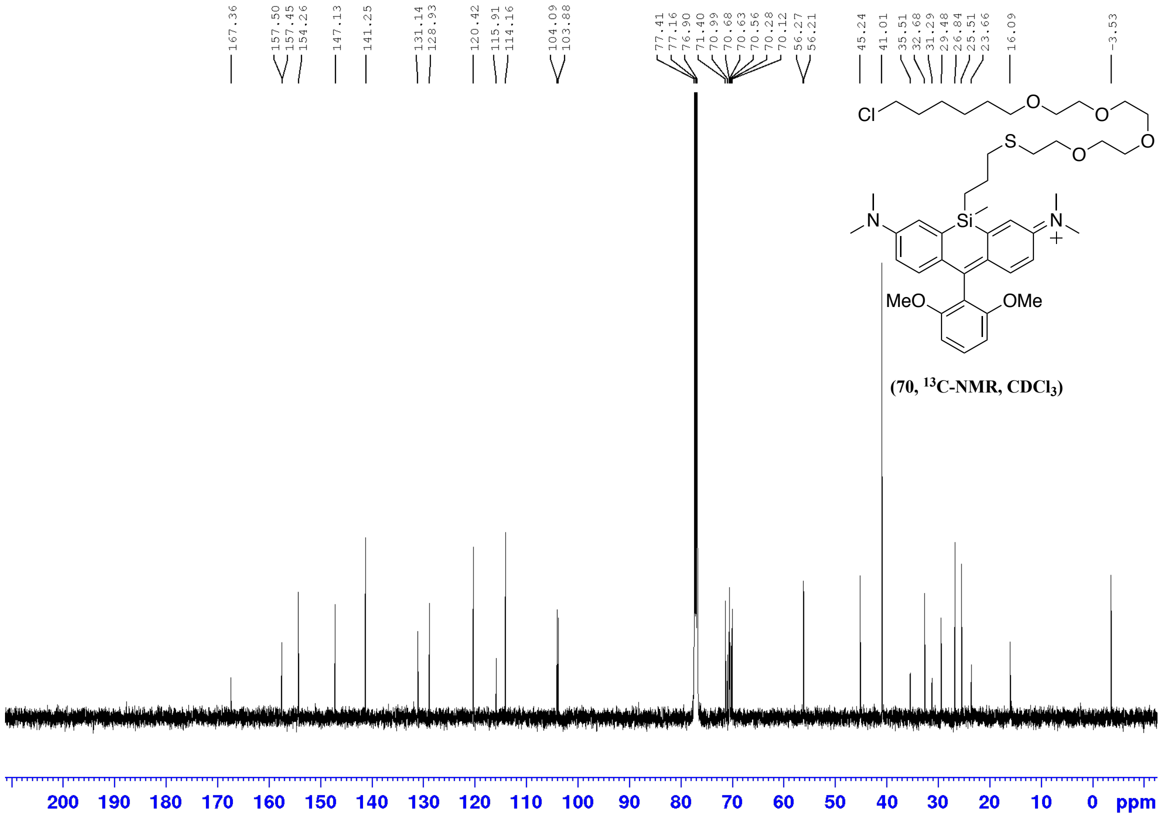

(70, ^{13}C-NMR, $CDCl_3$)

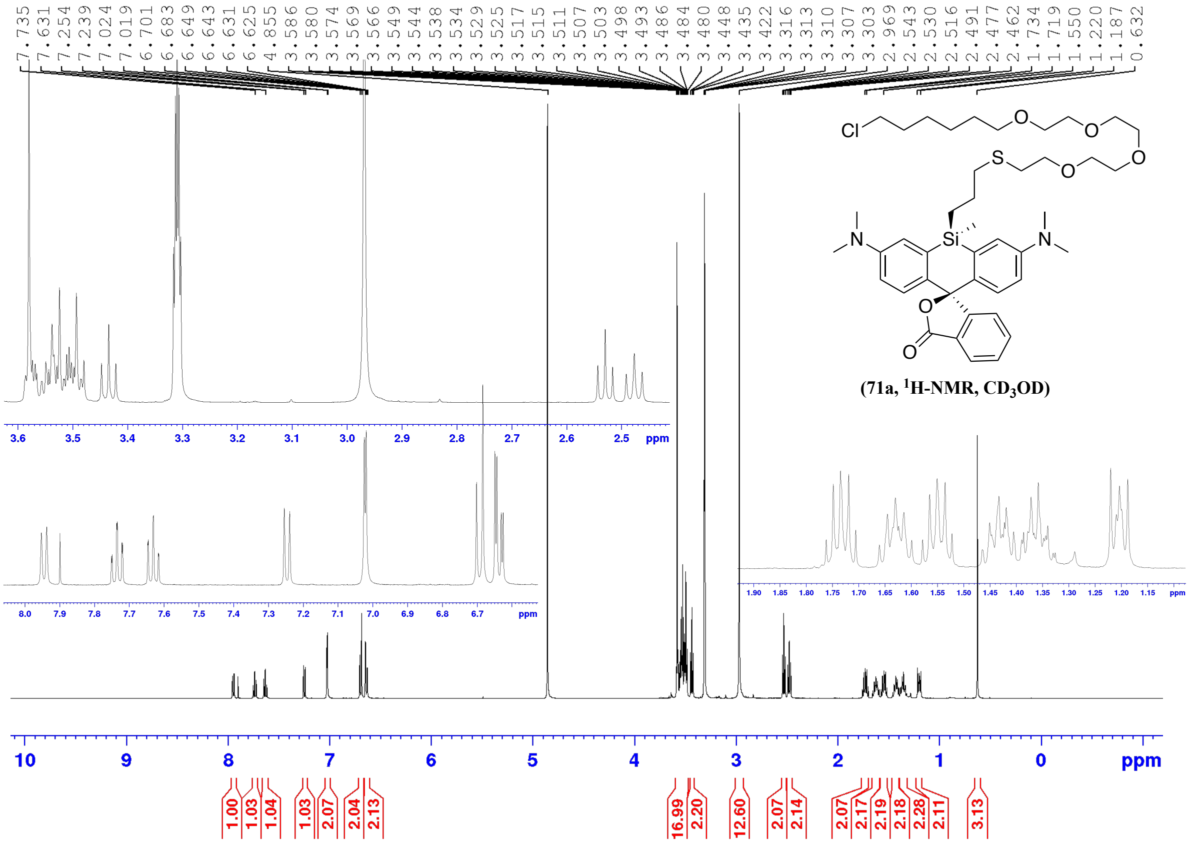

(71a, ^{1}H-NMR, CD_3OD)

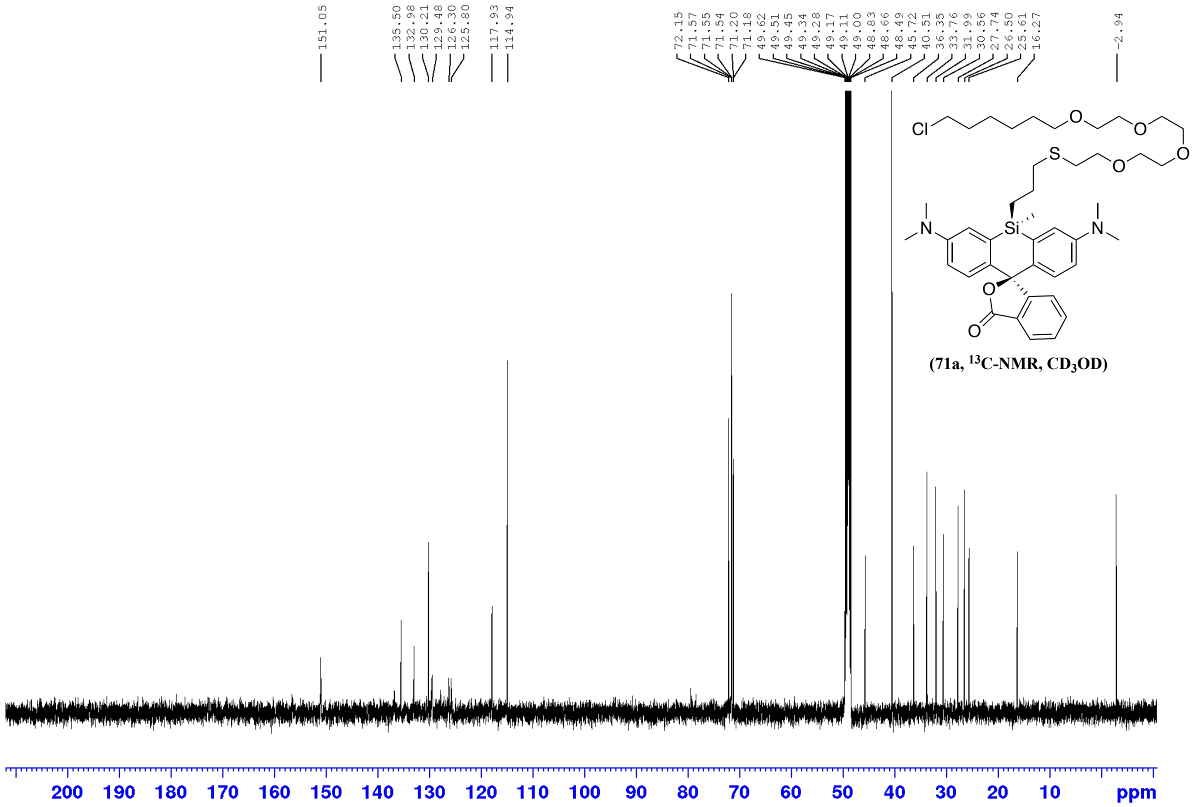

(71a, ^{13}C-NMR, CD_3OD)

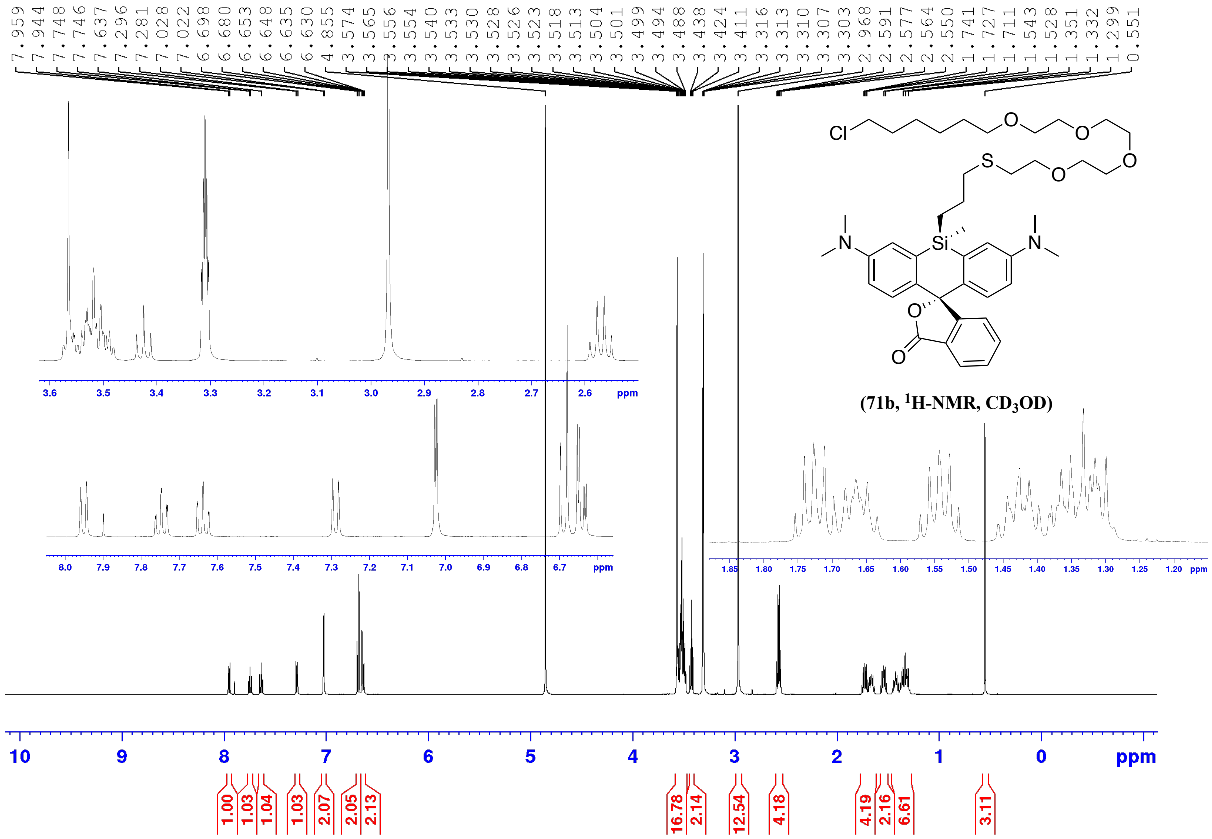

(71b, ^{1}H-NMR, CD_3OD)

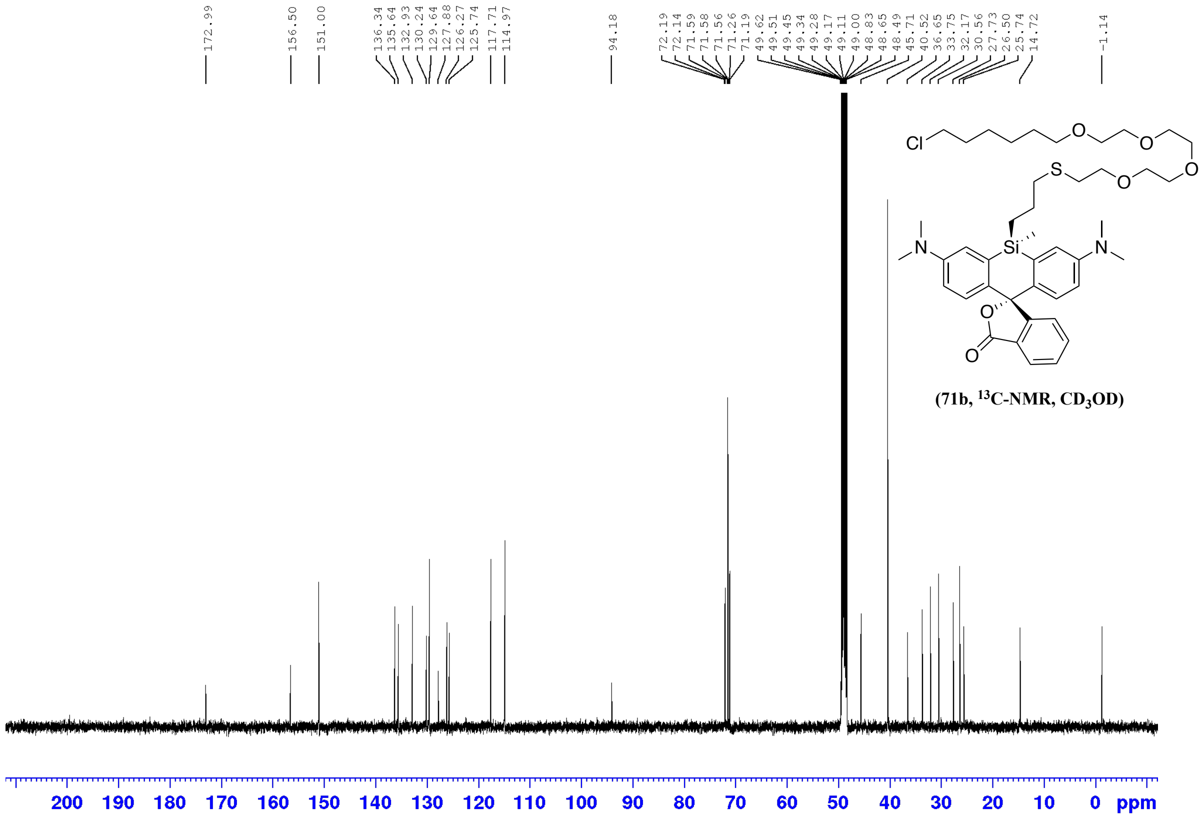
(71b, ¹³C-NMR, CD₃OD)

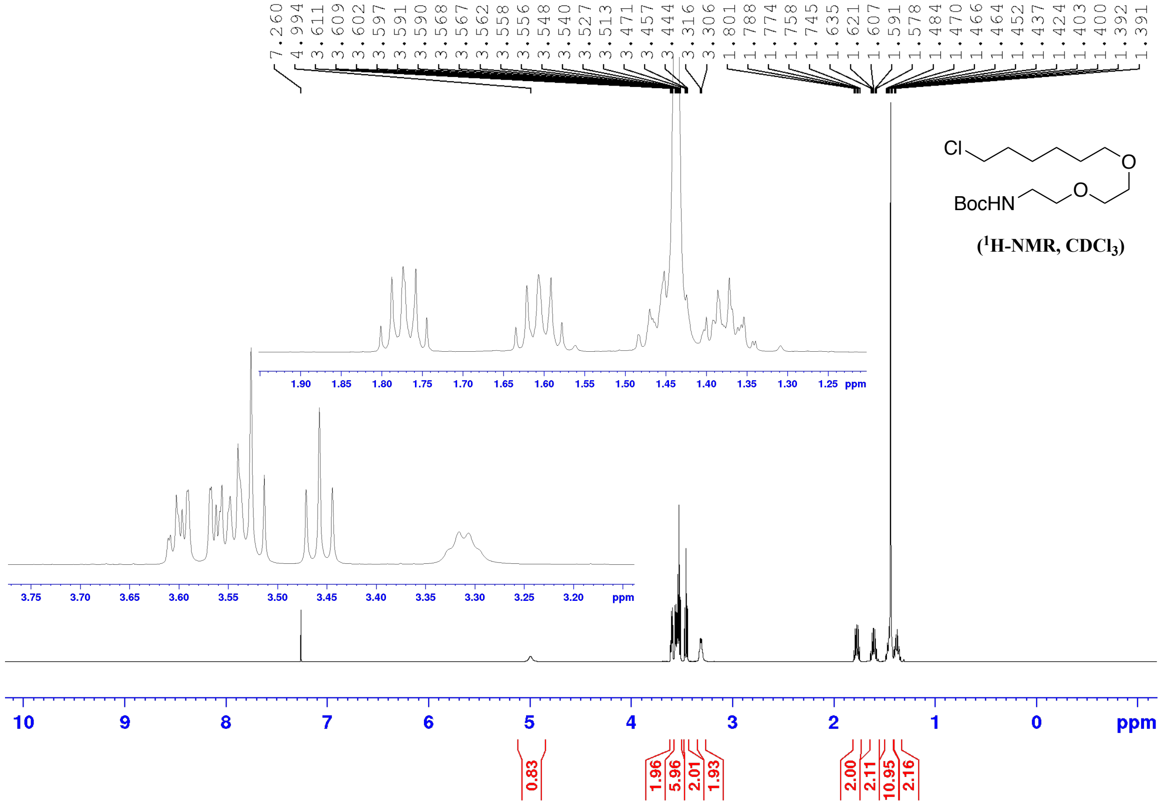
BocHN
(¹H-NMR, CDCl₃)

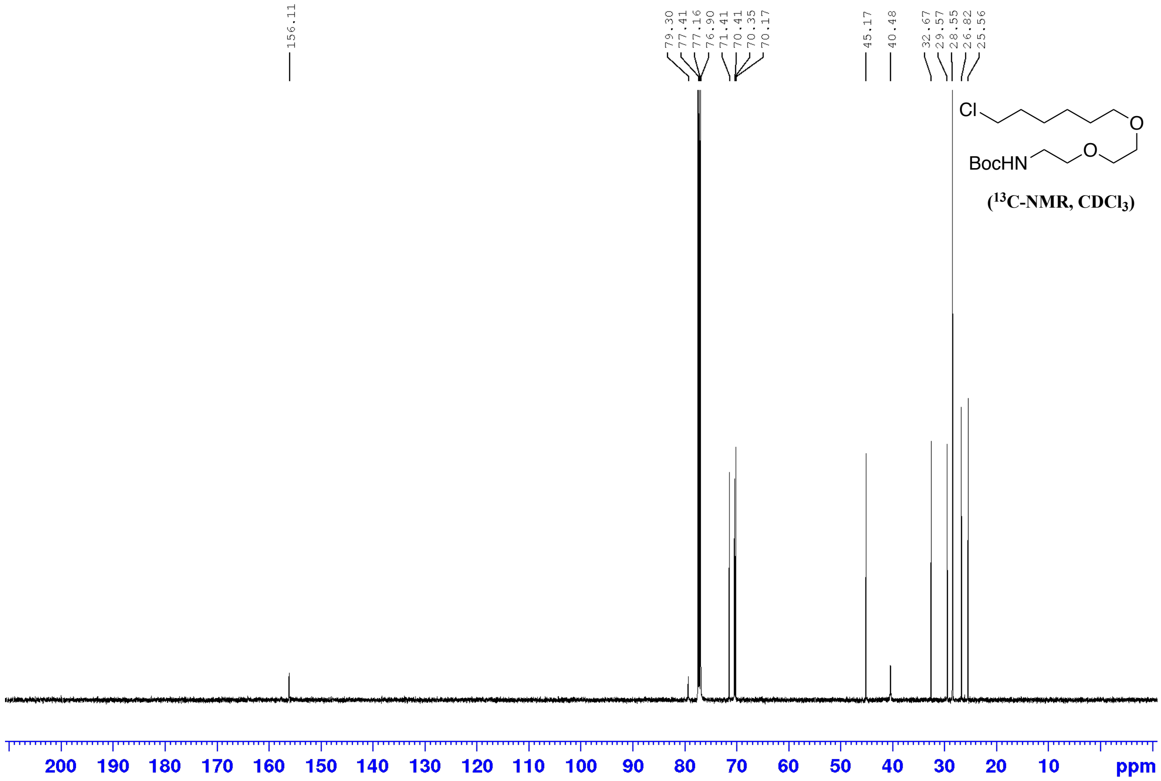

(^{13}C-NMR, $CDCl_3$)

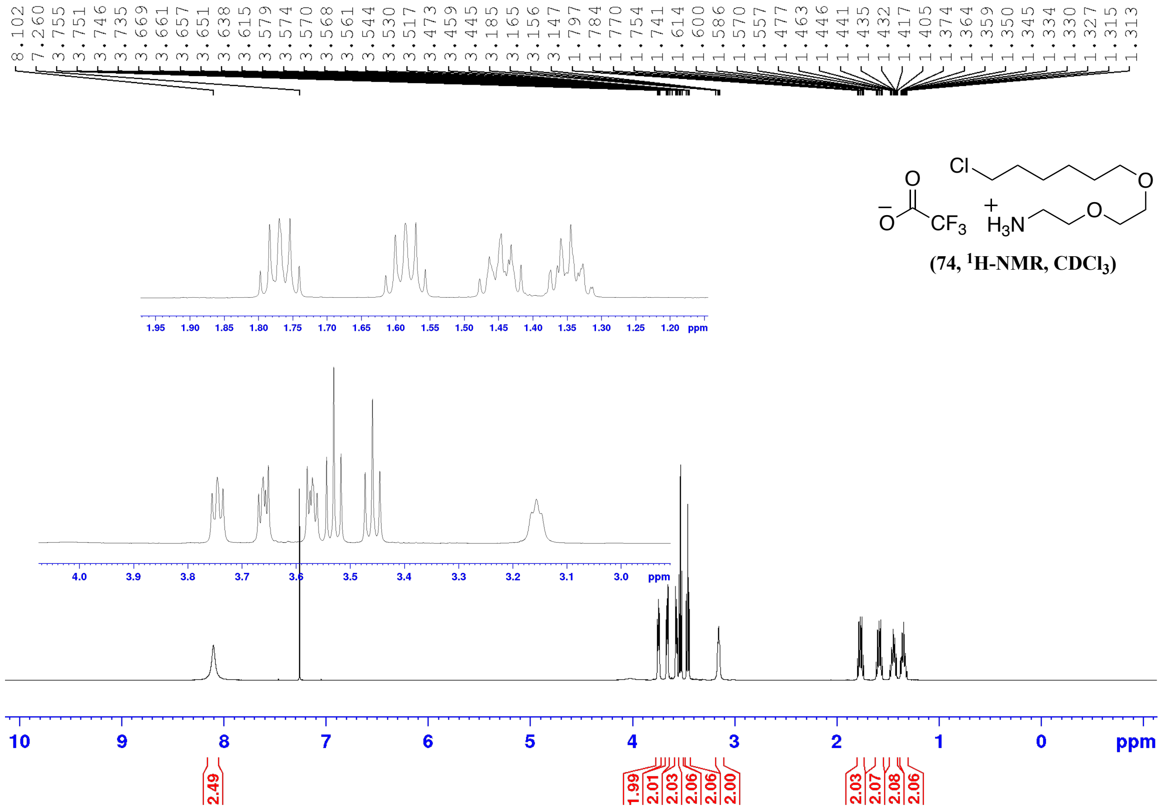

(74, ^{1}H-NMR, $CDCl_3$)

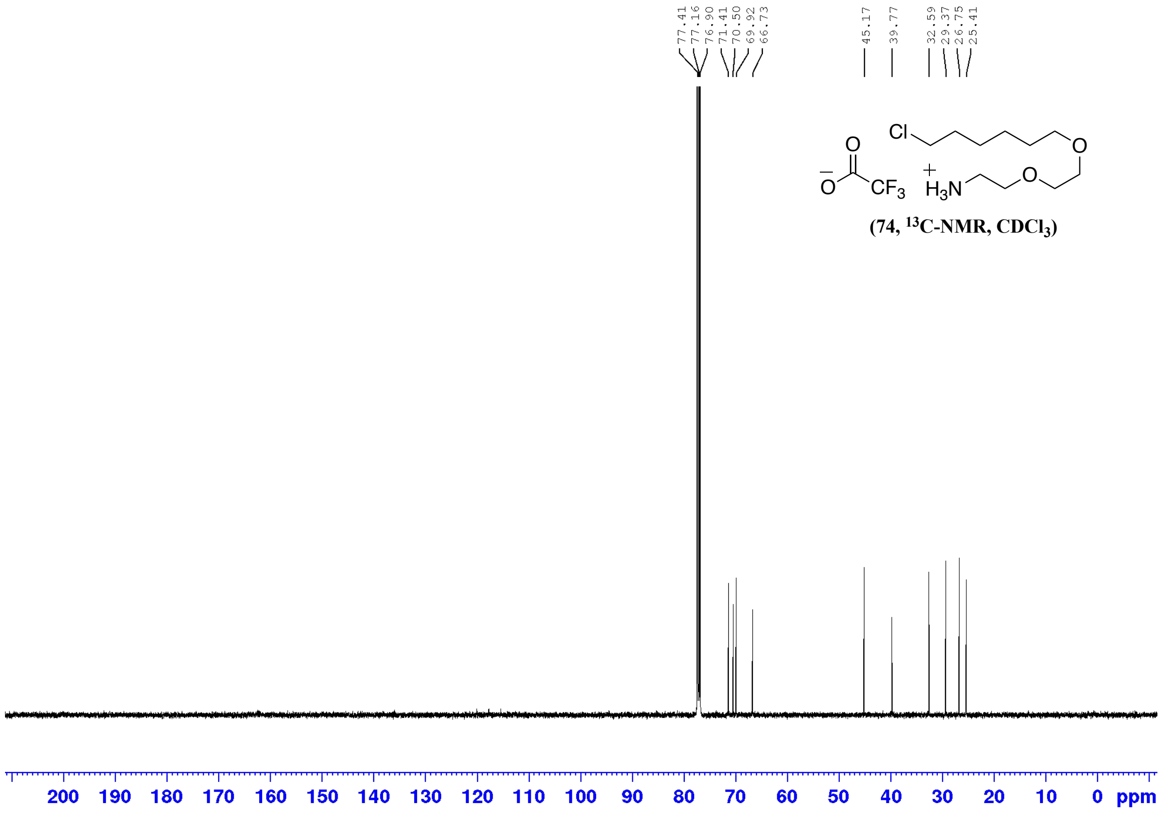

(74, ^{13}C-NMR, $CDCl_3$)

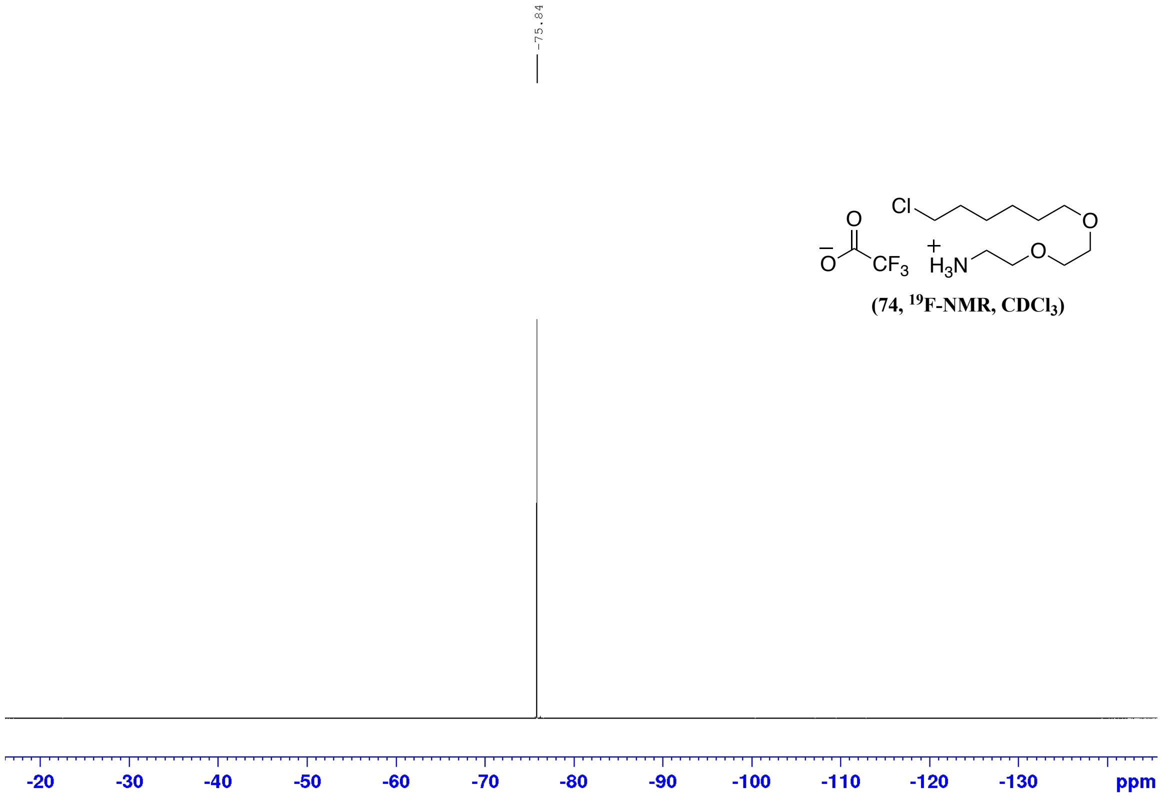

(74, ^{19}F-NMR, $CDCl_3$)

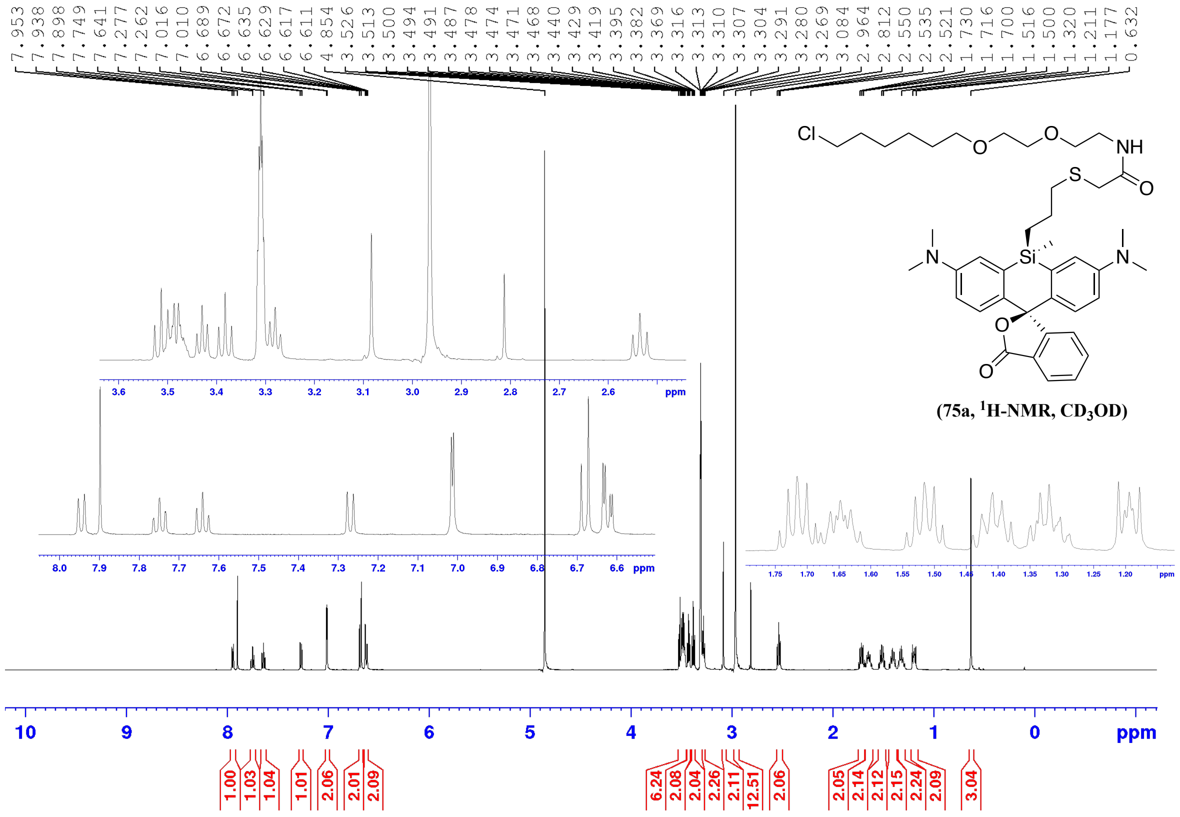

(75a, ^{1}H-NMR, CD_3OD)

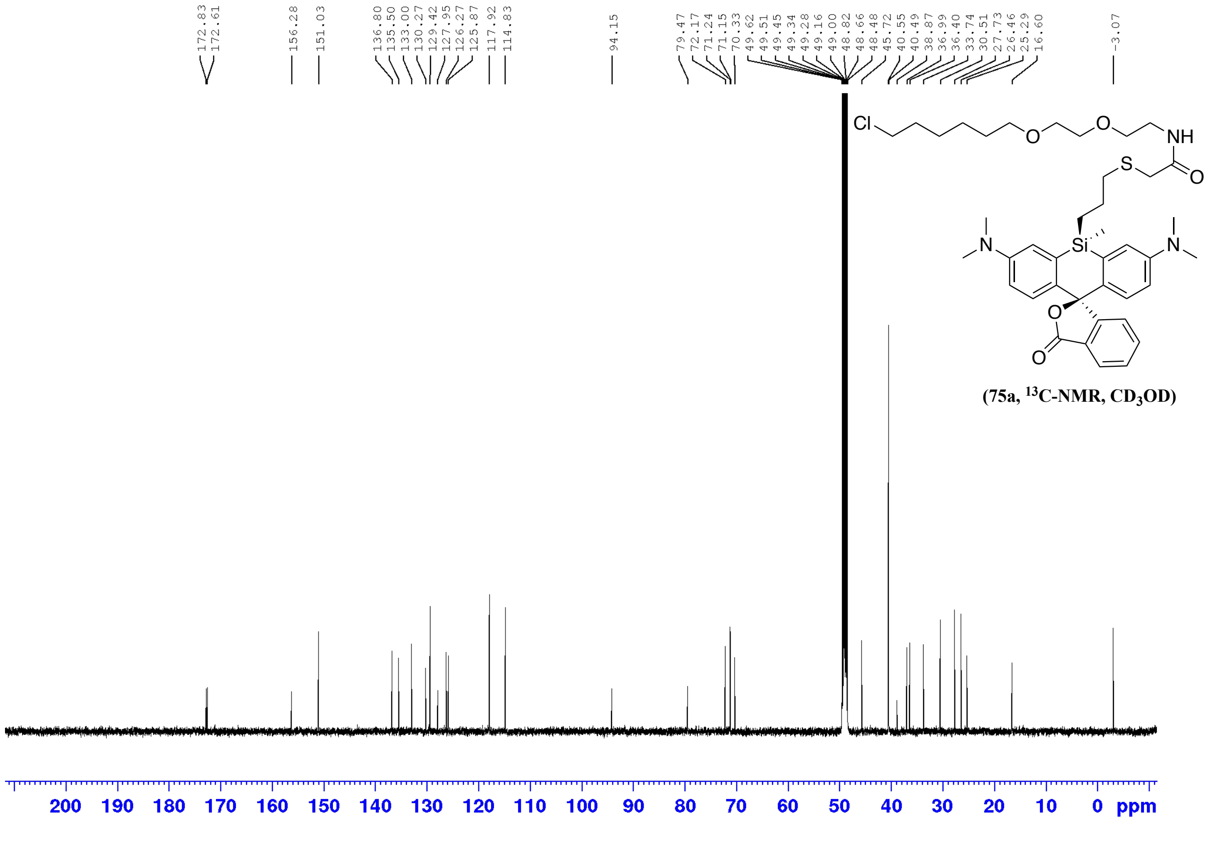

(75a, ^{13}C-NMR, CD_3OD)

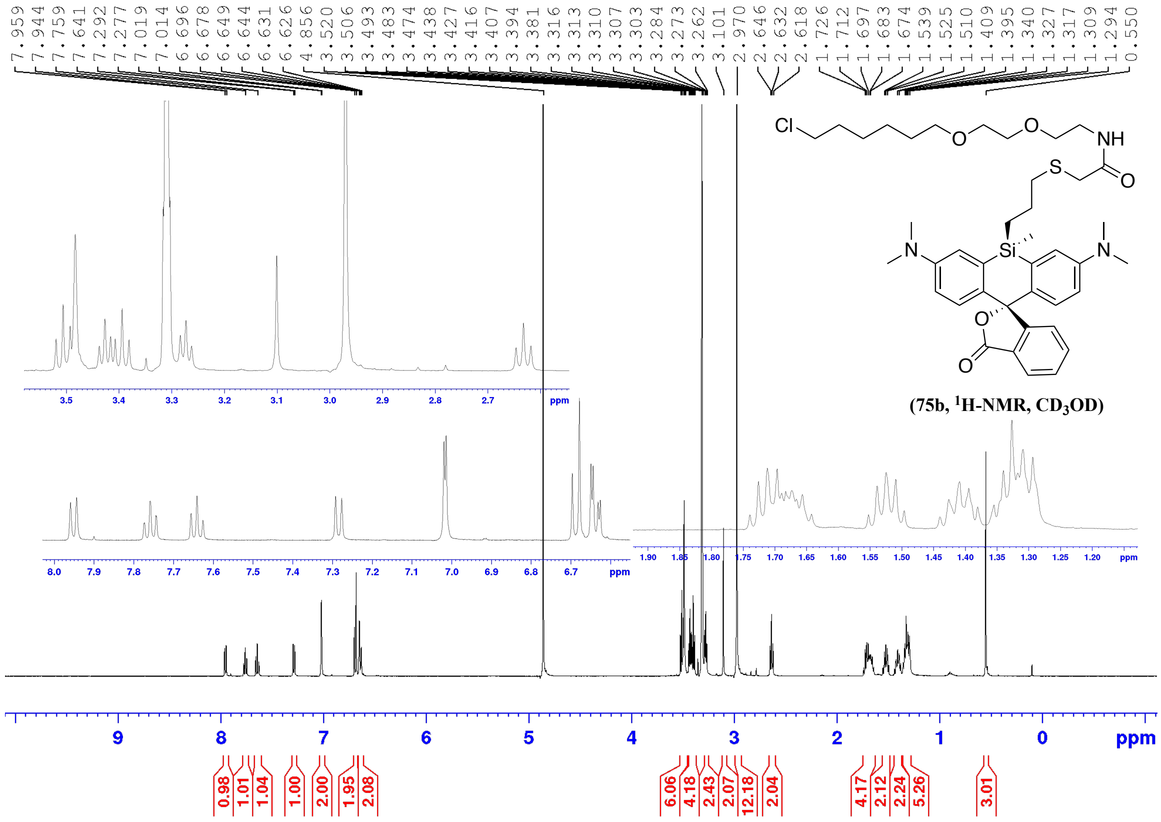

(75b, ^{1}H-NMR, CD_3OD)

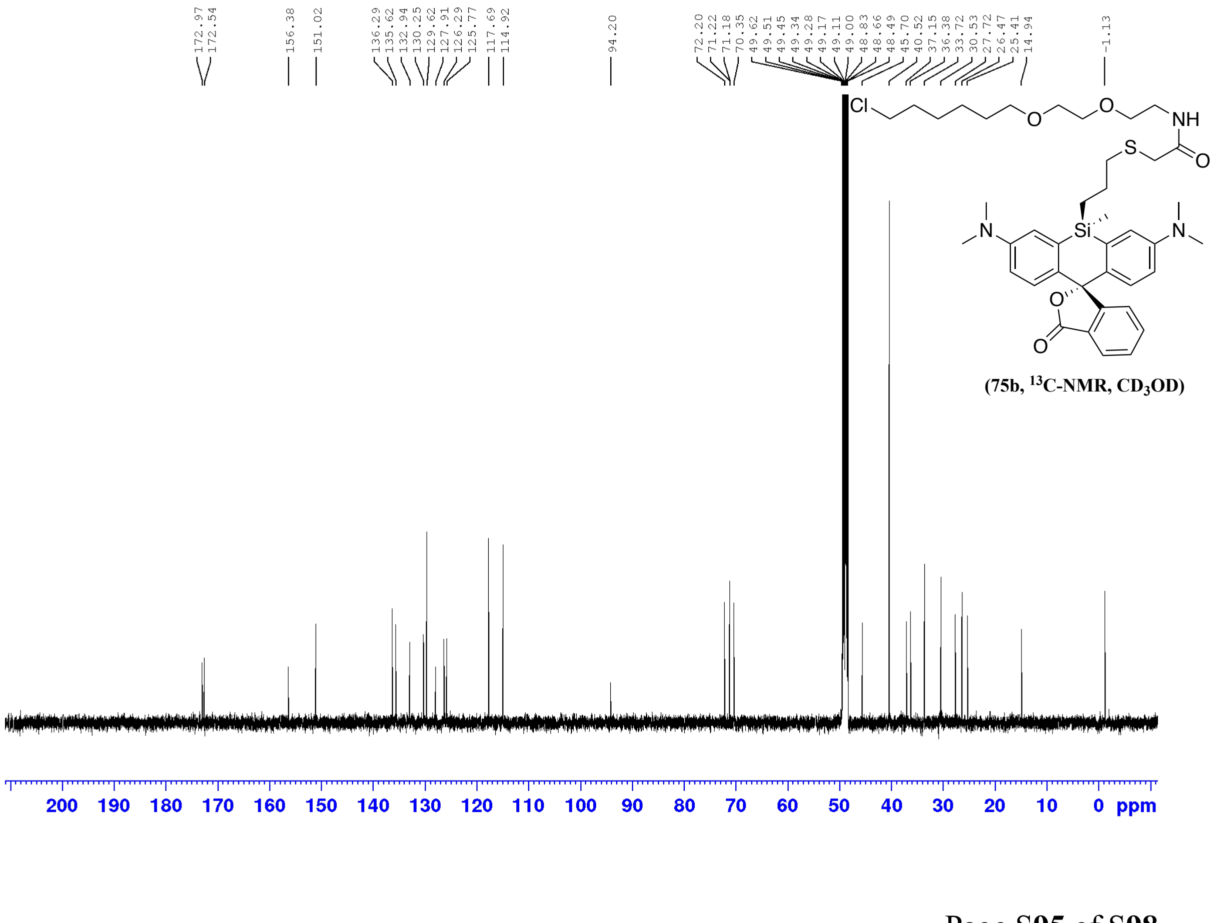

(75b, ^{13}C-NMR, CD_3OD)

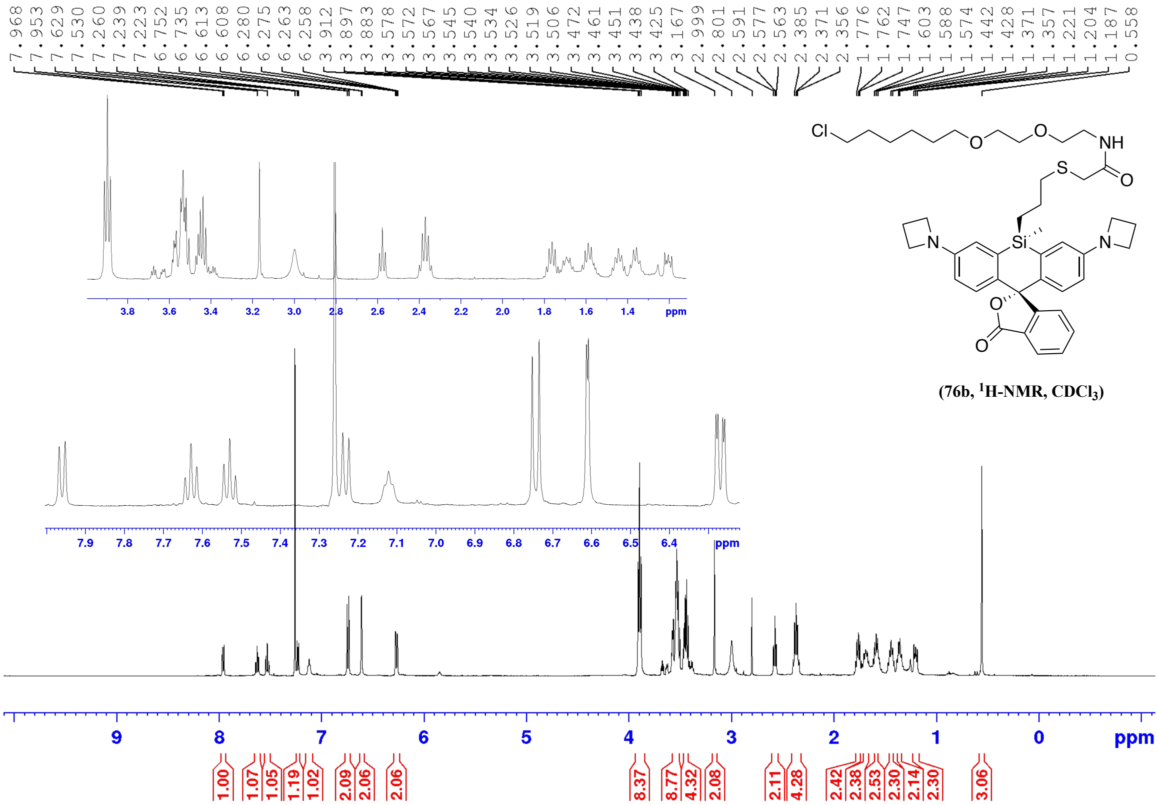

(76b, ^{1}H-NMR, $CDCl_3$)

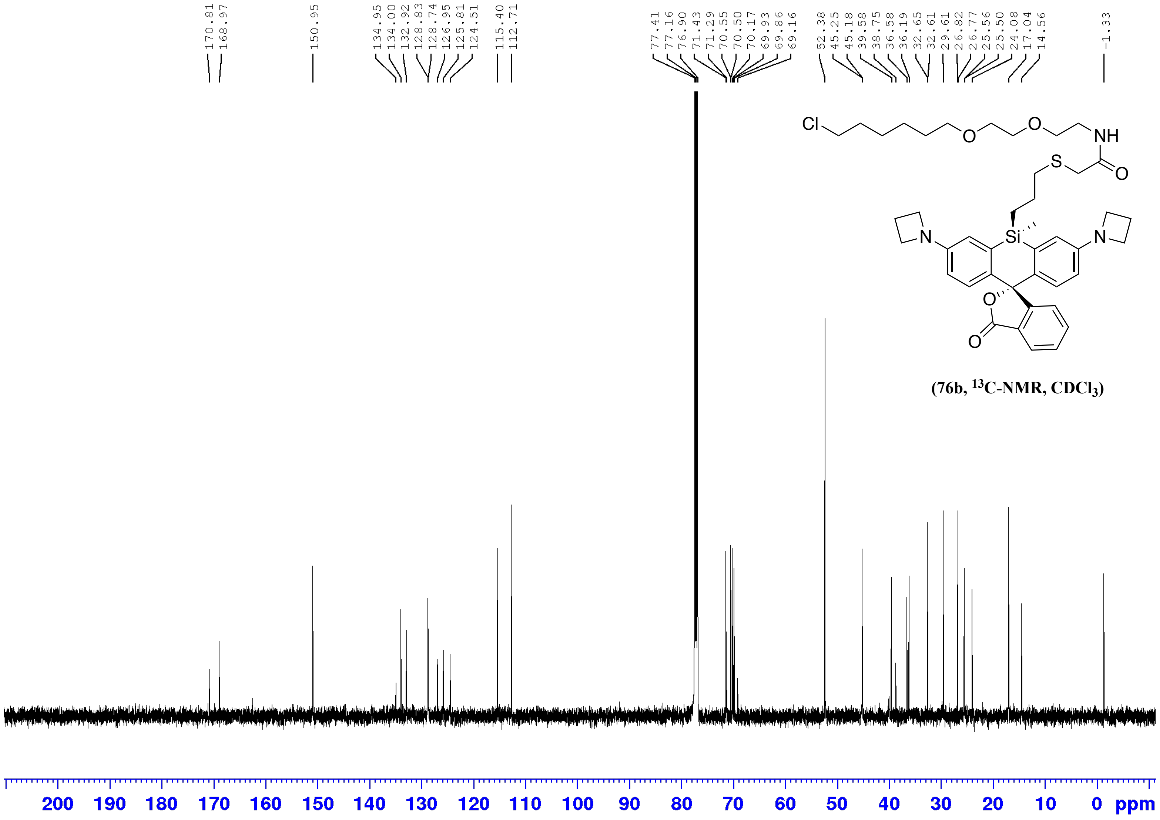

(76b, ^{13}C-NMR, $CDCl_3$)

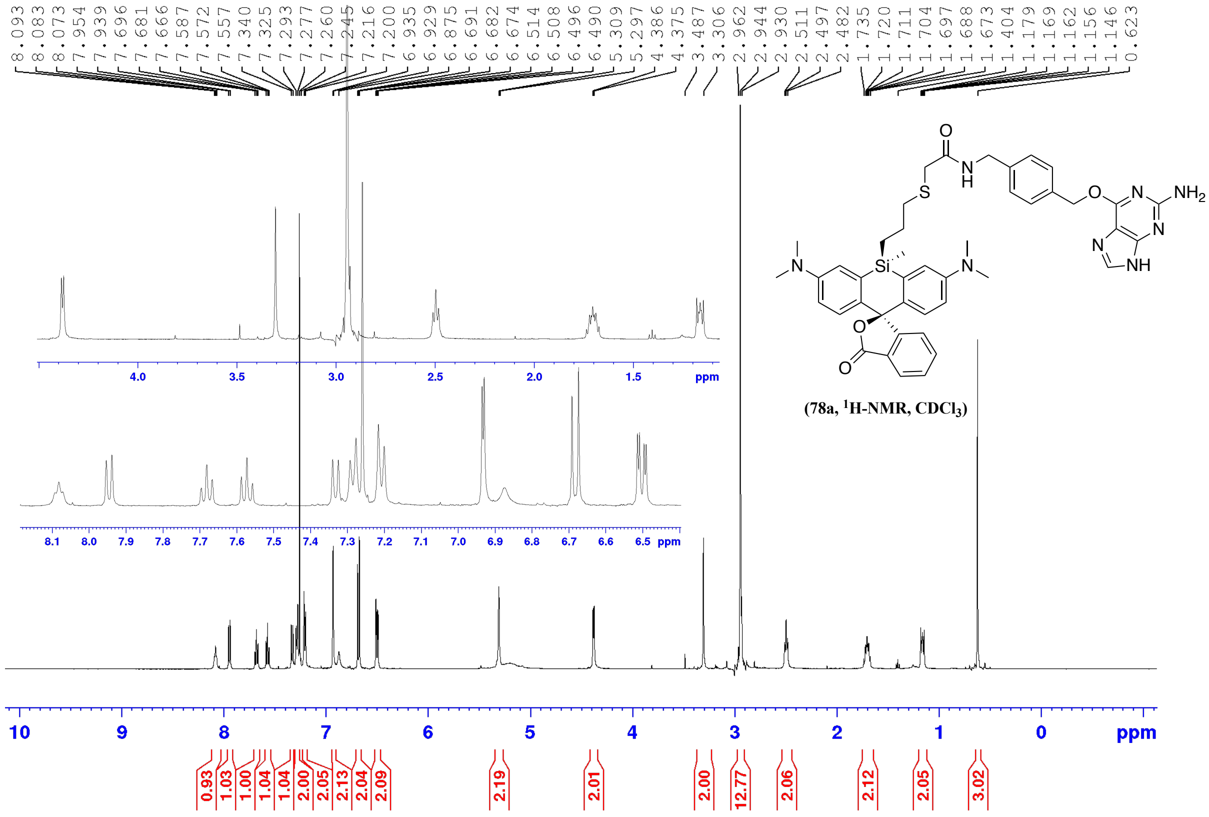

(78a, ^{1}H-NMR, $CDCl_3$)

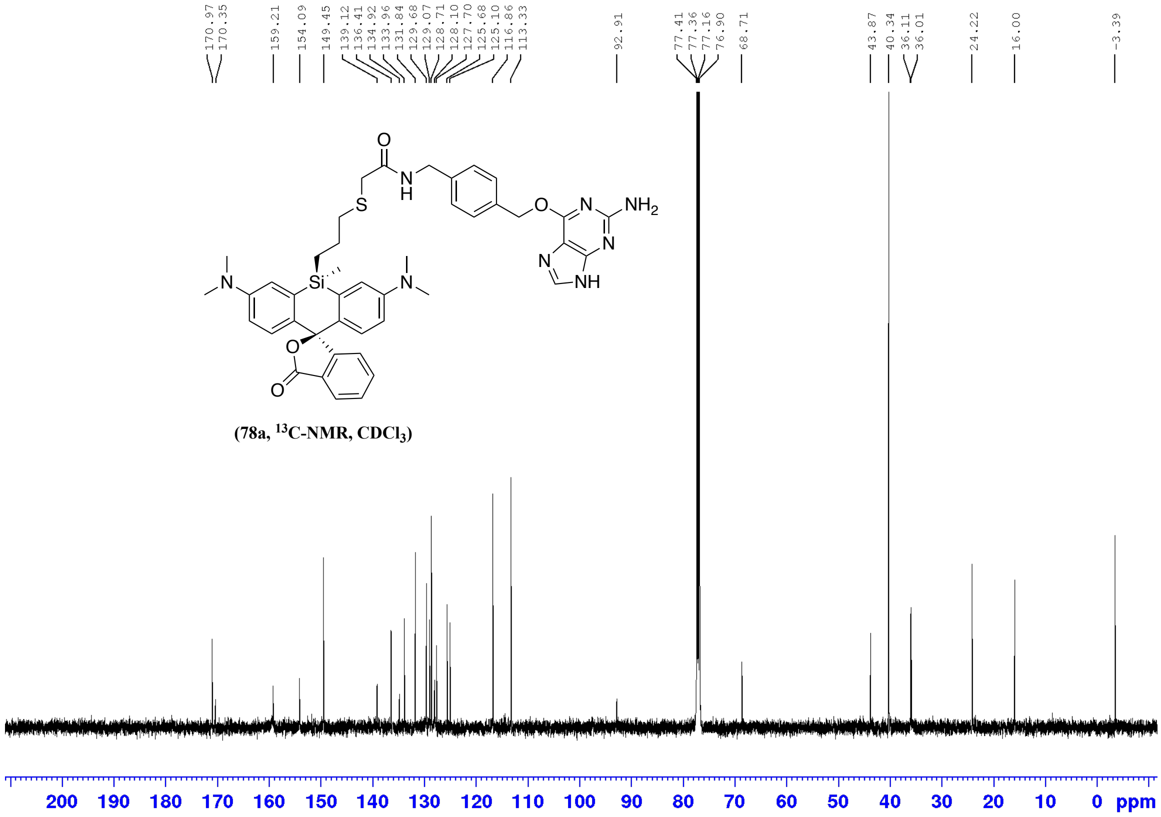

(78a, ^{13}C-NMR, $CDCl_3$)

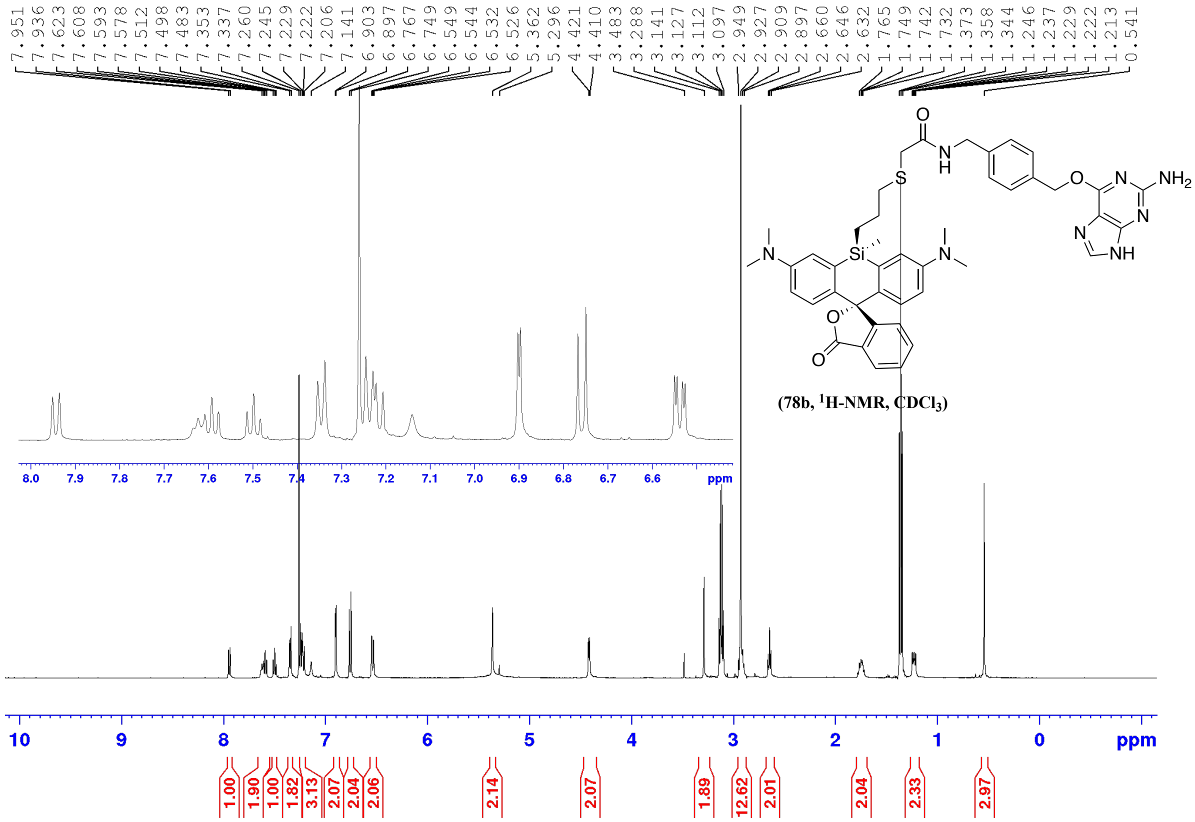

(78b, ^{1}H-NMR, $CDCl_3$)

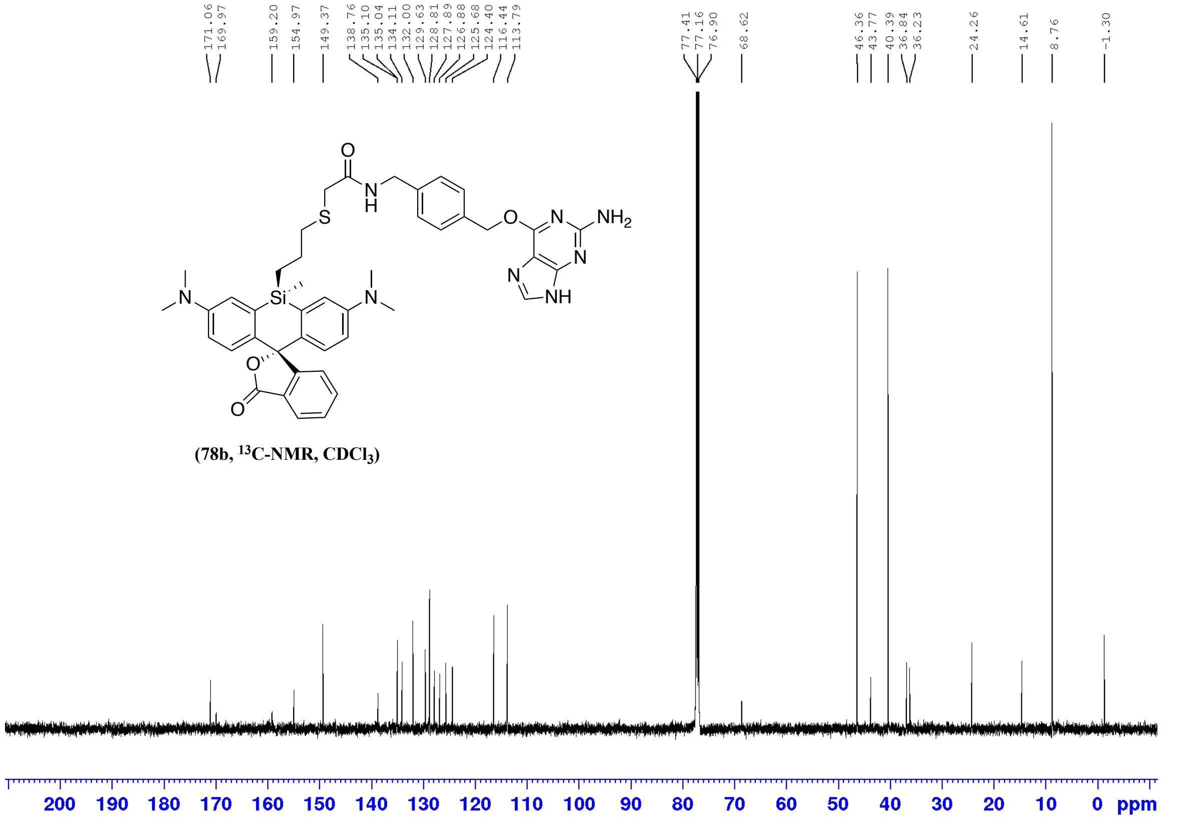

(78b, ^{13}C-NMR, $CDCl_3$)
